# USP5 acts as a proteolytic switch to control SDE2 function via UBL-directed cleavage

**DOI:** 10.1038/s41467-026-76751-6

**Published:** 2026-08-14

**Authors:** Liam T. Hales, Paul M. Tammiste, Adam J. Walker, Rebecca Bryce, Andrew Sugden, Axel Knebel, Rachel Toth, Frédéric Lamoliatte, Marian Péteri, Oliwia Curry, Glenn R. Masson, Virginia De Cesare, Chiara Maniaci

**Affiliations:** 1 MRC Protein Phosphorylation and Ubiquitylation Unit, Sir James Black Centre, Faculty of Life Sciences, University of Dundee, Dundee, UK; 2 School of Natural and Environmental Sciences, Newcastle University, Newcastle Upon Tyne, UK; 3 Division of Cancer Research, Faculty of Health, University of Dundee, Ninewells Hospital, Dundee, UK

**Keywords:** Deubiquitylating enzymes, Ubiquitylation, Enzyme mechanisms, Chemical tools

## Abstract

Proteolytic processing is a fundamental regulatory mechanism in eukaryotic cells, yet the molecular identities and mechanisms underlying such events are often poorly defined. Silencing Defective 2 (SDE2), an essential human protein, plays important roles in mRNA splicing, DNA repair and ribosomal biogenesis. Cleavage of SDE2 downstream to its N-terminal ubiquitin-like domain (SDE2_UBL_) releases the biologically functional C-terminal domain (SDE2_CT_), highlighting the importance of this proteolytic event. However, the protease responsible for this cleavage in human cells has remained undefined. Here, we identify deubiquitinating enzyme, ubiquitin-specific protease 5 (USP5), as the selectively primary effector of SDE2 cleavage both in vitro and in cell. Biophysical and structural analysis suggests that SDE2_UBL_ engages with USP5 through a two-site interaction that mirrors key features of ubiquitin recognition, supporting a mechanism of substrate mimicry. Functionally, depletion of USP5 increases intron retention in previously reported SDE2-dependent transcripts, linking the removal of SDE2_UBL_ domain by USP5 to the role of SDE2 in mRNA splicing. Together, these findings reveal a non-canonical proteolytic function of USP5, uncovering a previously unrecognised regulatory axis linking deubiquitinating enzymes to protein maturation, expanding the substrate repertoire for USP5 and providing a framework for identifying protease–substrate relationships in post-translational regulation.

## Abstract (web summary)

Deubiquitinases (DUBs) are canonically defined by their ability to process ubiquitin, but this may represent only part of their biological function. Here, the authors identify USP5 as the protease that cleaves SDE2, revealing an unexpected non-canonical activity of DUBs in RNA splicing regulation.

## Introduction

Precise regulation of protein abundance and function via insertion or removal of post-translational modifications (PTMs) lies at the heart of cellular homoeostasis^1^. Ubiquitin (Ub) and ubiquitin-like proteins (UBLs) play a key regulatory role by functioning as covalent modifiers that modulate protein activity and direct proteins towards diverse cellular fates, including signalling for degradation, mediating protein-protein interactions, modulating protein activity, and altering protein localisation^2,3^. These modifications are orchestrated by a sophisticated array of enzymes capable of writing, reading and erasing Ub and UBLs, resulting in a dynamic regulatory system interwoven in nearly every aspect of cell signalling, and therefore often disrupted during pathogenesis^4,5^. At the centre of this system lies a cascade of enzymatic reactions culminating in the covalent attachment of Ub/UBL to a target protein by an E3 ligase^6,7^. Antagonising this process are proteases, including deubiquitinating enzymes (DUBs), capable of proteolytically cleaving Ub and UBLs, typically downstream to a conserved C-terminal Gly-Gly motif^8,9^.

While UBLs share high structural similarity with Ub and are often regulated by overlapping enzymatic machinery, many have evolved distinct biological functions through an additional layer of selective interactions with their respective conjugation and deconjugation machinery. For instance, a cascade of interferon induced enzymes, UBEL1-UBE2L6-HECT5, specifically conjugate interferon-stimulated gene 15 (ISG15), rather than Ub, playing an important role in immune response^10–12^. Similarly, the misleadingly named ubiquitin-specific protein 18 (USP18) selectively cleaves ISG15 from tagged proteins, with no activity toward Ub^13,14^. These selective interactions facilitate nuanced activation and suppression of Ub and UBL signalling cascades in response to diverse stimuli, and are therefore crucial for effective cellular functioning.

Ub-like domains (ULDs) offer an additional layer of Ub-mimetic regulation without requiring post-translational conjugation^15^. While Ub acts as a molecular tag by canonically modifying the ε-amino groups of lysine residues, ULDs structurally resemble the Ub fold but are encoded intrinsically within protein sequences^16^. ULD-bearing proteins often participate in Ub-regulated processes, using the ULD to interact with Ub-binding partners, enabling fine-tuned cross-talk between distinct signalling circuits^15^. Whilst many ULDs are stable features of a protein’s structure, some contain conserved protease-recognition motifs that permit proteolytic cleavage and irreversible removal of such domains. Proteolysis of such domains resembles the DUB-mediated processing of immature Ub precursors from poly Ub chains (UBB and UBC) or from the ribosomal fusion proteins encoded by the UBA52 and UBA80 genes^17^. In such cases, these cleavable ULDs act as internally encoded “tags”, with their removal rewiring protein fate and function.

**Fig. 1:**
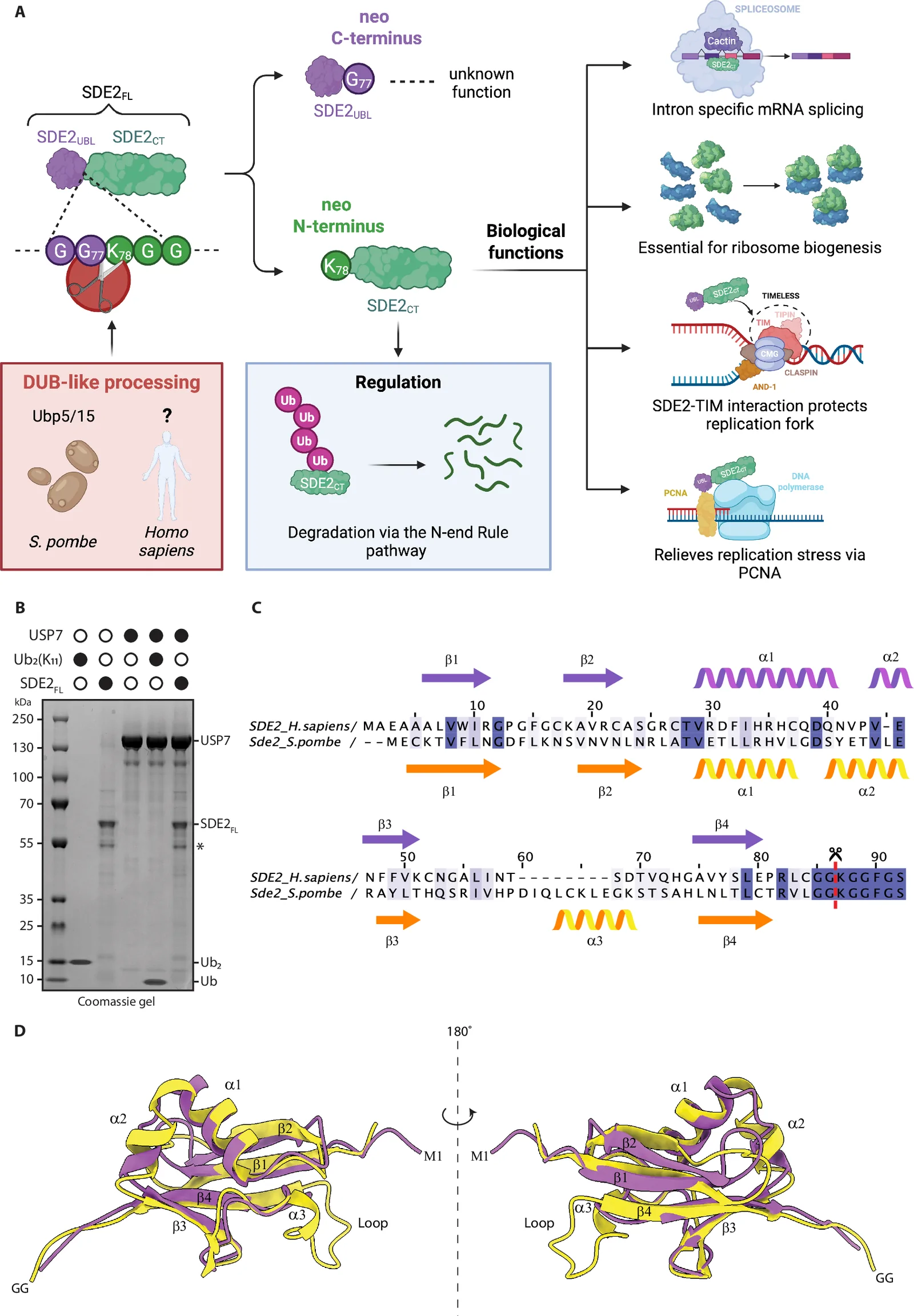
Overview of SDE2 biology and regulation. **A** Schematic overview of SDE2 processing, regulation and biological functions. Created in BioRender. Maniaci, C. (2026) https://BioRender.com/2gomvtv. **B** USP7 was screened as potential candidate protease for human SDE2 based on Protein-BLAST analysis of Ubp5/15. Recombinant USP7 was incubated with either Ub_2_ (K11) or SDE2_FL_ at 37 °C for 1 h and analysed by SDS-PAGE and Coomassie staining. * indicates a protein contaminant. Ub_2_(K11) represents K11-linked diubiquitin. **C** Sequence alignment, conservation, and predicted secondary structures of the Ub fold of *H. sapiens* SDE2 (purple) and *S. pombe* Sde2 (yellow/orange). Fully conserved residues are highlighted in dark-blue and semi-conserved residues in light-blue. **D** Structural comparison of *S. pombe* (yellow) and *H. sapiens* (purple) SDE2 ubiquitin-like fold. Structures were predicted using AlphaFold 3^30^ and visualised in ChimeraX^80^. Secondary structures are highlighted and annotated corresponding to  (**C**).

First identified in yeast, Silencing Defective 2 (*H. sapiens*: SDE2, *S. pombe:* Sde2) is one such ULD-containing protein whose proteolytic processing is essential for its biological roles, exemplifying this intriguing mode of regulation^18,19^. SDE2 is a highly conserved and essential gene required for genome maintenance which coordinates essential processes, ranging from pre-mRNA splicing and ribosome biogenesis to DNA repair and replication stress response^18,20,21^. It is expressed as a full-length precursor (SDE2_FL,_ 49.7 kDa) bearing an N-terminal ULD, which is proteolytically cleaved to generate two protein fragments, SDE2_UBL_ (8.35 kDa) and the larger C-terminal domain (SDE2_CT_, 41.4 kDa) (Fig. 1A). Akin to canonical Ub cleavage, this proteolytic event occurs downstream to a conserved Gly-Gly motif, which becomes exposed following ULD removal, forming the de novo C-terminus of SDE2_UBL_^22^.

The proteolytic cleavage of SDE2 represents a crucial regulatory step that underpins its diverse roles. This cleavage is central to SDE2 function and is required for two major functional outcomes. First, the removal of the sterically occluding SDE2_UBL_ domain permits the liberated SDE2_CT_ to be incorporated into spliceosomes where it promotes the efficient excision of specific introns from a subset of pre-mRNAs^20,23^. This activity is linked to the spliceosomal association of Cactin/Cay1, with genetic and biochemical evidence supporting a functional interplay between SDE2 and Cactin^20^. Second, the cleaved C-terminal domain, but not full-length SDE2, negatively regulates damage-inducible monoubiquitination of PCNA, a key modification required for translation DNA synthesis^22^. Timely proteasomal degradation of SDE2 is necessary to eliminate this inhibitory activity, thereby allowing S-phase progression and replication fork recovery following DNA damage^24–26^. Failure to cleave or degrade SDE2 leads to its pathological accumulation, which disrupts the finely tuned balance of PCNA ubiquitination. This dysregulation results in impaired S-phase progression, stalled replication forks, elevated DNA damage, and increased cell death^20,22^. Importantly, SDE2 homoeostasis itself is governed by proteolysis. Removal of the UBL domain exposes a destabilising N-terminal lysine (K78) on SDE2_CT_ and initiates its degradation via the N-end rule pathway^26^. Thus, SDE2 cleavage serves as a molecular switch that coordinates replication stress responses and RNA processing to safeguard cell proliferation and genome integrity.

Although the regulatory importance of SDE2_UBL_ removal is well established, the enzyme responsible for the cleavage of human SDE2 remains unknown. Given that SDE2 is an essential protein, identifying the protease responsible for this processing event is critical. Moreover, defining the mechanisms that control SDE2 cleavage is key to revealing the intricacies of SDE2 regulation and its associated biological functions. To date, SDE2 dysfunction and the importance of its proteolytic activation has been implicated in the development and progression of oncological conditions such as multiple myeloma and prostate cancer, highlighting the importance in understanding SDE2 regulation^27,28^.

In this study, we report the identification of the protease responsible for SDE2 processing, resolving a previously uncharacterised mechanism of post-translational regulation. Using a targeted strategy combining biochemical fractionation of cell lysate with mass spectrometry-based proteomic analysis, we have identified USP5 as the protease responsible for the cleavage of human SDE2. This finding reveals a previously unrecognised cross-reactive activity of this DUB that extends beyond its canonical function. Using complementary approaches, including development of an activity-based SDE2_UBL_ probe and a live-cell NanoBRET^®^ reporter assay, we confirm that USP5 selectively cleaves SDE2 both in vitro and in cell. Through biophysical characterisation and mutagenesis studies, we further define key structural features of USP5 required for this non-canonical substrate recognition event. Finally, we confirm the impact of USP5 catalysed SDE2 cleavage on spliceosome activity. Together, these findings expand the functional landscape of USP5 and establish an additional layer of UBL-driven proteolytic regulation within the cell.

## Results

### Divergence in SDE2_UBL_ processing suggests a distinct human protease

Studies conducted in *S. pombe* identified deubiquitinating enzymes Ubp5 and Ubp15 as the proteases responsible for Sde2_UBL_ cleavage, confirming the role of DUBs in this processing^20^. This suggested that a related DUB might perform this function in humans. Several human homologues for Ubp5 and Ubp15 have been reported, including ubiquitin-specific proteases (USPs) USP8, USP50, and USP7. We first performed a Protein BLAST analysis^29^ using Ubp5 and Ubp15 as query sequences. The search identified USP7 as the top human match for both yeast enzymes. Despite a modest 34% sequence identity, USP7 shares up to 50% identity with the catalytic domain of Ubp5/15, further supporting its candidacy. To test this hypothesis, we performed an in vitro cleavage assay by incubating recombinant USP7 with recombinantly expressed SDE2_FL_. However, while USP7 actively processed Ub_2_ (K11), no processing of recombinant human SDE2 was observed (Fig. 1B). These findings prompted us to more closely compare full length human SDE2 (451 amino acids) and *S. pombe* Sde2 (263 amino acids) to understand potential structural divergence. Despite a 39% sequence identity, conservation is concentrated around the GGKGG cleavage motif (Fig. 1C and Supplementary Fig. 1), suggesting a preserved recognition element. Secondary structure prediction of SDE2_UBL_ and Sde2_UBL_ revealed strong alignment between the two UBL domains, with the major structural difference being the presence of an additional alpha helix (α3) (amino acids D59-E65) in the yeast protein that is absent in the human SDE2 (Fig. 1C). Structural modelling using AlphaFold 3^30^ confirmed the overall structural similarity of the two UBL folds (RMSD = 1.574). However, the model also highlighted divergent structural elements including the alpha helix (α3) together with an additional loop positioned near the N-terminal tail, distant from the conserved C-terminal cleavage site, present in Sde2_UBL_ but not in SDE2_UBL_ (Fig. 1D). These observations indicate that, despite the structural conservation around the cleavage region, species specific structural differences may influence protease recognition. The failure of USP7 to cleave SDE2_UBL_ in vitro suggests that the human protease responsible may differ substantially from those in yeast, either in sequence or in mechanism of substrate engagement. As such, identifying the human protease responsible for SDE2_UBL_ cleavage is not only critical to understanding how SDE2 is regulated, but it may also reveal additional principles of substrate recognition by DUBs.

### Integrated biochemical and proteomic strategy implicates USP5 and USP13 in SDE2 proteolysis

**Fig. 2:**
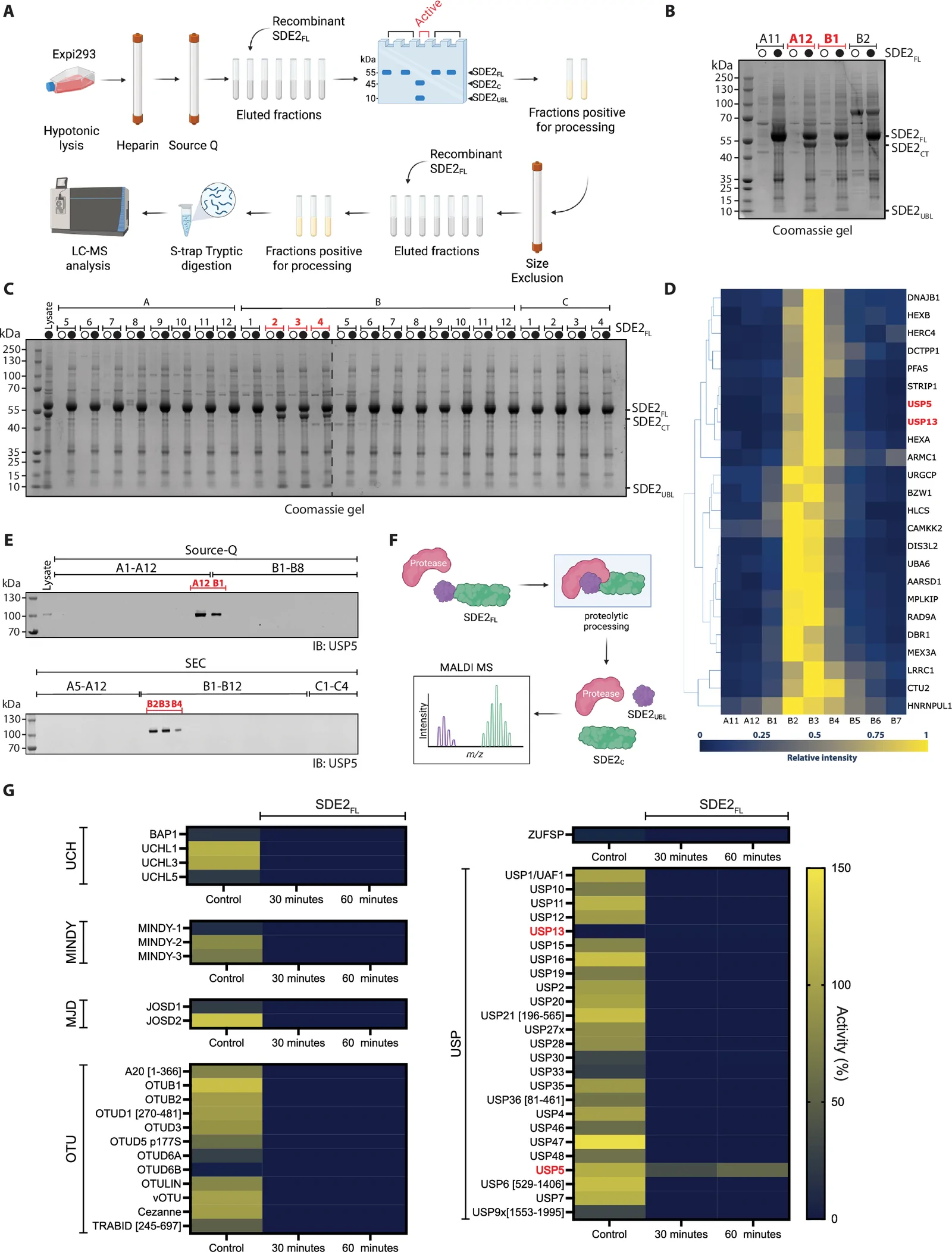
Screening for SDE2 protease identified USP5 and USP13 as candidate proteases. **A** Schematic overview of the lysate screening process. Expi293 cells were lysed in hypotonic conditions and fractionated sequentially over Heparin and Source-Q columns. Eluted fractions were screened for activity by incubation with recombinant SDE2_FL_. Fractions showing activity were processed by size exclusion chromatography (SEC) and collected fractions were screened again for processing. Active fractions were subjected to tryptic digestion over S-trap column and analysed by mass spectrometry. Created in BioRender. Maniaci, C. (2026) https://BioRender.com/j93q834. **B** Representative screening results for fractions collected after Source-Q ion-exchange chromatography. Eluted fractions were incubated with SDE2_FL_ for 30 min at 37 °C and analysed by SDS-PAGE and Coomassie staining. Protein cleavage activity was detected in fractions A12 and B1 (in red). **C** Active Source-Q fractions (A12 and B1) were fractionated by SEC and the fractions screened for activity against SDE2_FL_. Eluted fractions were incubated with SDE2_FL_ for 30 min at 37 °C and analysed by SDS-PAGE and Coomassie staining. Protein cleavage activity was detected in fractions B2-B4 (in red). **D** Identity of proteins detected in all active SEC fractions as determined by proteomic mass spectrometry. **E** Immunoblot analysis of endogenous USP5 in fractions from Source-Q and SEC. Fractions that showed activity are highlighted in red. **F** Schematic overview of the high-throughput MALDI-TOF SDE2 cleavage assay. Recombinant proteases were incubated with SDE2_FL_ and the amount of SDE2_UBL_ formed by this reaction was determined by MALDI-TOF MS by the presence of peak for SDE2_UBL_ at *m/z* = 8348.9. Created in BioRender. Maniaci, C. (2026) https://BioRender.com/pfe981m. **G** Protease enzymes including known DUBs, pseudo-DUBs and proteases were incubated with their respective specific substrates (control) or with SDE2_FL_ for 30 or 60 min at 30 °C and analysed by the MALDI-TOF MS. The amount of SDE2_UBL_ formed by this reaction was determined by MALDI-TOF MS and quantified against ^15^N-Ub internal standard. Activity for the individual tested enzymes is reported in a gradient of blue (0%) to yellow (150%). Source data are provided as Source Data file.

To uncover the identity of the protease responsible for processing SDE2 in human cells, we first turned to a biochemical strategy using recombinant SDE2_FL_ as substrate and Expi293 cell lysate as the enzyme source (Fig. 2A). Expi293 cells were chosen for their higher protein yields and high-density growth. Recognising that protease activity can be sensitive to buffer conditions, we lysed Expi293 cells in hypotonic conditions specifically chosen to preserve endogenous enzymatic function. The crude lysate was then subjected to a two-step column chromatography workflow (Fig. 2A). In the first step, we applied the lysate to a heparin column to remove nucleic acid-binding and regulatory proteins. The heparin column flowthrough was then subjected to ion-exchange chromatography using a Source-Q column to separate proteins based on charge. This approach resolved the lysate into discrete fractions while maintaining enzymatic integrity. Next, each collected fraction was incubated with recombinant SDE2_FL_ and proteolytic activity assessed by monitoring the formation of the expected cleavage products (SDE2_CT_ and SDE2_UBL_) via SDS-PAGE. Proteolytic activity was highly restricted to two adjacent Source-Q column fractions, A12 and B1, indicating a successful enrichment of candidate protease (Fig. 2B). To further isolate the active enzyme, active fractions A12 and B1 were individually purified further using size exclusion chromatography (SEC), reasoning that this additional resolution step would help distinguish true proteolytic activity from co-eluting contaminants. SEC fractions were once again tested for their ability to cleave recombinant SDE2_FL_, revealing that activity was concentrated in fractions B2 through B4 (Fig. 2C and Supplementary Fig. 2A). These fractions, together with adjacent fractions (A11-B1 and B5-B7), were subjected to tryptic digestion followed by mass spectrometry. Overall, proteomic analysis identified 4339 protein groups across all fractions. To pinpoint candidate enzymes associated with SDE2-cleaving activity, we focused on proteins enriched specifically within the active Source-Q fractions (53 for A12 and 172 for B1) (Supplementary Fig. 2D). Among the identified proteins, 24 were common to both active fractions and of these, only two enzymes, DUBs USP5 and USP13, were annotated with known or predicted protease activity (Fig. 2D and Supplementary Fig. 2D). This finding aligned with the precedent reports of DUB-mediated Sde2 processing in *S.pombe*^20^. Western blot analysis confirmed the presence of both USP5 and USP13 across the active fractions from both Source-Q and SEC chromatography steps, strengthening the case for their involvement (Fig. 2E). To confirm whether these DUBs could cleave SDE2, we performed an orthogonal cleavage assay in which USP5 or USP13 was incubated individually with recombinant SDE2_FL_. Cleavage activity was assessed by MALDI-TOF/MS, using the detection of SDE2_UBL_ fragment as the primary readout (Fig. 2F and Supplementary Fig. 3). To evaluate specificity and rule out alternative proteases, we extended this assay to a panel of 70 recombinant human and viral proteases, including 65 DUBs, some of which have no known substrates. (Fig. 2G and Supplementary Fig. 3). Among the entire panel, only USP5 and USP13 consistently cleaved SDE2_FL_ across biological replicates. Notably, USP5 exhibited the highest level of activity cleaving approximately 50% of the input substrate under the assay conditions (Fig. 2G). Together, these results implicate USP5 and USP13 as leading candidates for the processing of human SDE2, with USP5 emerging as the dominant enzyme in recombinant systems.

### An SDE2_UBL_-derived activity-based probe reacts with USP5, but not USP13, in a catalytically dependent manner

**Fig. 3:**
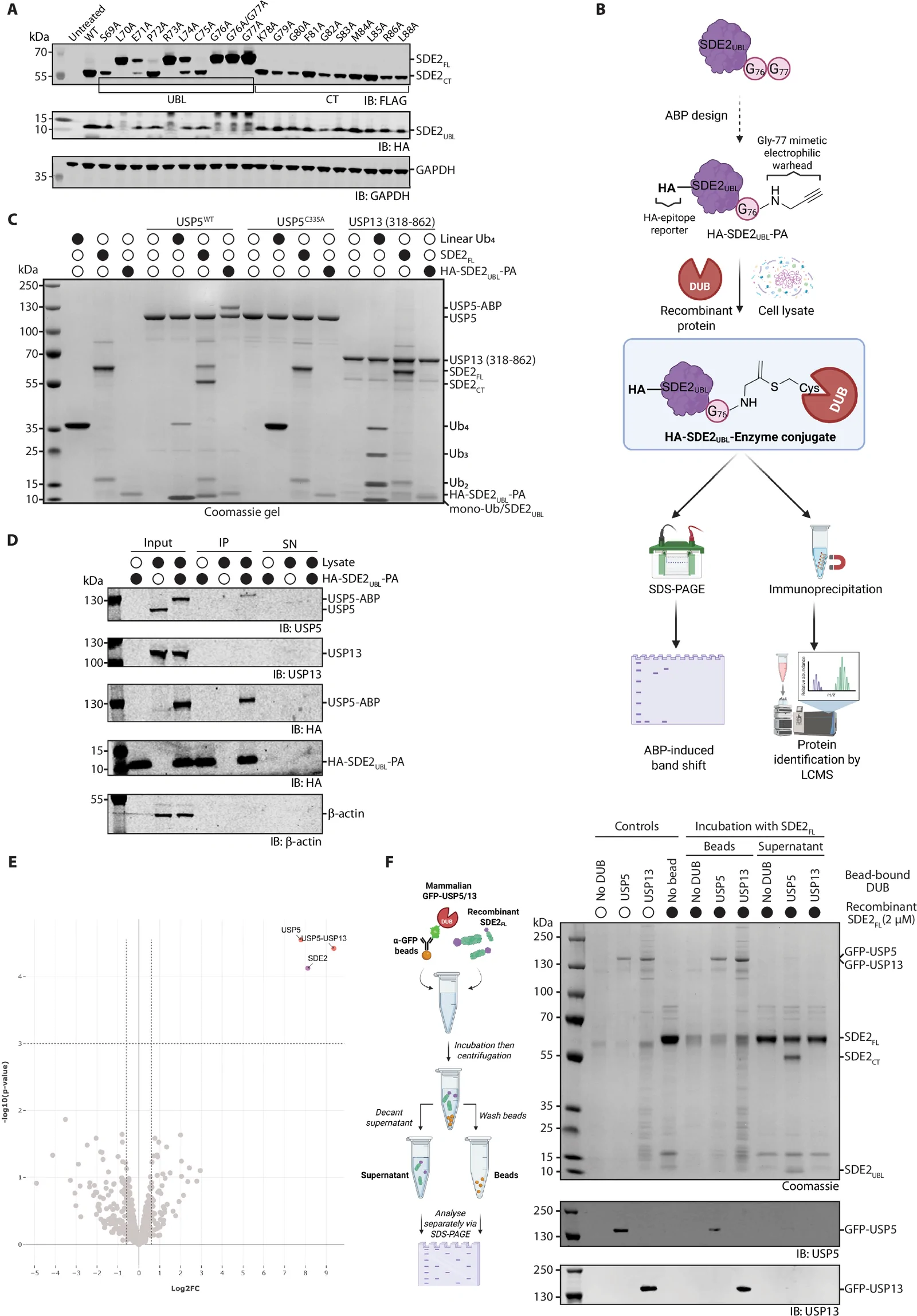
HA-SDE2_UBL_-PA selectively reacts with USP5, in in vitro and cell lysate contexts. **A** Representative immunoblotting results from alanine scanning of the residues adjacent to the site of cleavage. HEK293T cells overexpressing HA-SDE2-FLAG^WT^ or indicated mutants were lysed and immunoblotted for the HA and FLAG epitopes to determine the effect of each mutation on SDE2_FL_ cleavage. The location of the residues is annotated as either the UBL or C-terminal domain (CT). **B** An ABP derived from SDE2_UBL_ was designed and prepared semi-synthetically. Gly77 is replaced with a propargyl electrophilic warhead to mimic the scissile amide bond. An N-terminal HA epitope tag facilitates recognition by immunoblotting. This ABP can be used to detect catalytic-dependent interactions with enzymes, either by incubation with recombinant protein (visualisation by SDS-PAGE), or with cell lysate followed by HA-mediated immunoprecipitation and analysis by immunoblotting and mass spectrometry. Created in BioRender. Maniaci, C. (2026) https://BioRender.com/irimq8w. **C** Assessment of USP5^WT^/USP5^C335A^ and USP13 (318-862) reactivity towards various substrates. DUBs (1 µM) were incubated with HA-SDE2_UBL_-PA (2 µM), SDE2_FL_ (2 µM) or linear tetraubiquitin (Ub_4_, 1 µM) for 1 h at 37 °C and analysed by SDS-PAGE. **D** Immunoblot analysis following immunoprecipitation of HEK293T lysate treated with HA-SDE2_UBL_-PA. HEK293T cell lysate was incubated with HA-SDE2_UBL_-PA (1 µM) for 45 min at 37 °C and immunoprecipitated with anti-HA beads. **E** Volcano plot analysis of enriched HEK293T cell lysate following HA-SDE2_UBL_-PA treatment. ABP immunoprecipitated samples (triplicate) were subject to trypsin digestion and proteomic analysis, including comparison to the UniProt Swiss-Prot human database to identify the parent protein. Samples were compared to a ‘no-probe’ negative control. Two-tailed differential expression analysis was carried out using an empirical Bayes-moderated linear model and *P* values were corrected using Benjamini-Hochberg multiple hypothesis correction. **F** On-bead DUB cleavage assay where α-GFP beads conjugated to Expi293 recombinantly expressed GFP-USP5 or GFP-USP13 were incubated with recombinant SDE2_FL_ (2 µM) for 1 h at 37 °C. The beads and reaction supernatant were analysed separately via SDS-PAGE followed by staining with Coomassie blue or immunoblotting. Created in BioRender. Maniaci, C. (2026) https://BioRender.com/xymvuv8.

Having identified USP5 and USP13 as the candidate proteases responsible for cleavage of SDE2_UBL_, we next sought to define the amino acids residues required for the SDE2_FL_ cleavage event. For Ub, key residues involved in recognition by DUBs are primarily located within the C-terminal tail, including the highly conserved LRGG motif and residue R72^31^. We therefore hypothesised that the interaction between SDE2_FL_ and the identified DUBs may similarly be mediated by the same C-terminal region (L70-G77) of SDE2_UBL_. To test this, we focused our analysis on the 10 amino acid segment spanning S69 and K78, which encompasses the previously reported cleavage site motif (GGK)^20,22^. In addition, we analysed the downstream region between G79 and L88 to account for potential contribution from residues immediately following the cleavage site. To functionally interrogate the residues within these regions, we employed an alanine scanning approach. A library of SDE2 expression plasmids was generated in which each amino acid within the selected region was individually mutated to alanine. An N-terminal hemagglutinin (HA) tag and a C-terminal FLAG tag were included in the construct to allow for detection of both fragments. As controls, we also included a plasmid expressing SDE2_FL_ wild type (WT) and a G76A G77A double mutant, previously reported to abrogate processing^20,22^. HEK293T cells were transiently transfected with each plasmid, and SDE2 processing was evaluated by immunoblotting. Interestingly, the mutations that impaired SDE2 processing were all localised in the region of UBL domain. Indeed, while none of the tested mutations in the region corresponding to the start of SDE2_CT_ affected SDE2 cleavage, several mutations in the SDE2_UBL_ region seem to reduce or abrogate SDE2 processing to different degrees. As expected, mutagenesis of the diglycine (GG) motif, both single and double mutations, resulted in impairment of SDE2 processing (Fig. 3A). Mutations L70A, and R73A also completely prevented SDE2 cleavage. Notably, these residues correspond to positions that are also conserved in Ub and are known to contribute to recognition by DUBs, suggesting that SDE2 cleavage relies on similar structural determinants. In contrast, mutations of amino acids E71 and L74 resulted in approximately 50% cleavage of SDE2, indicating that these amino acids play a supporting role in the cleavage process.

To further interrogate these interactions, we used AlphaFold 3 to model SDE2_UBL_ or Ub bound to USP5 (Supplementary Fig. 4). L70^SDE2^ is predicted to form part of the hydrophobic core (including I33, V49 and F47) of SDE2_UBL_. Mutation of equivalent Ub residue (L69^Ub^) has been reported to disrupt key interactions which maintain the structural integrity of Ub^32^. Therefore, in the case of SDE2_UBL_ mutation may result in similar structural changes, preventing molecular recognition and cleavage of SDE2_FL_. Similarities are also evident for conserved residue R73^SDE2^ which is predicted to form electrostatic interactions with E427^USP5^ and E438^USP5^ analogous to those formed by R72^Ub^. Likewise, L74^SDE2^ is positioned within the same hydrophobic pocket of USP5 occupied by L73^Ub^^33^. A notable point of divergence between SDE2_UBL_ and Ub is the predicted hydrogen-bond formed between E71^SDE2^ and Q488^USP5^. Interestingly, the equivalent residue (V70^Ub^) is unable to form a similar interaction, suggesting that E71^SDE2^ may specifically contribute to SDE2 recognition. Together, these mutagenesis and modelling data highlight the importance of specific SDE2_UBL_ C-terminal tail residues for efficient SDE2 processing. While several of these determinants mirror those required for Ub recognition, others appear to unique to SDE2_UBL_. These results further suggest that the interaction between SDE2_UBL_ and USP5/USP13 may represent a crucial driving force for this proteolytic event.

Given the importance of the UBL domain sequence for SDE2_FL_ cleavage_,_ we next sought to interrogate the SDE2_UBL_-USP5/USP13 interaction through the development of an SDE2_UBL_ activity-based probe (ABP) designed to selectively react with enzymes capable of recognising SDE2_UBL_. We developed a HA-tagged SDE2_UBL_ probe using a semi-synthetic strategy. In this design, SDE2_UBL_ serves as a recognition element, while the N-terminal HA tag functions both as an enrichment handle and reporter tag for detection via immunoblotting, enabling profiling of protease enzymatic activity in both recombinant and complex biological mixtures (Fig. 3B). Many UBLs have been converted into ABPs through the installation of electrophilic warheads which mimic the C-terminal scissile amide bond. In most cases, this is achieved by replacing the terminal glycine with an electrophilic glycine mimetic. This strategy has been successfully applied in activity-based profiling campaigns to identify novel DUBs^34^, or to uncover previously unrecognised UBLs substrates for various DUBs^35^. Following this principle, a propargylamine warhead was installed at the C-terminus to act as covalent trap for catalytically active SDE2-interacting cysteine proteases.

To generate a functional SDE2_UBL_ probe, we employed a intein-mediated ligation based method^36^. An N-terminally HA-tagged SDE2_UBL_ truncated at Gly77 (HA-SDE2_UBL_ΔG77) was expressed as a fusion with a tandem self-cleaving intein and chitin-binding domain, enabling efficient purification using chitin affinity resin. Cleavage from the resin was induced by addition of 2-mercaptoethane sulfonate (MESNA), generating a reactive C-terminus thioester (HA-SDE2_UBL_-MESNA). The final ABP (HA-SDE2_UBL_-PA) was produced by incubating HA-SDE2_UBL_-MESNA with excess propargylamine, which initiates the nucleophilic substitution to install the electrophilic warhead, as previously reported^34^.

A propargyl warhead was selected due to its unique electrophilic characteristics which prevent it from reacting with untargeted cysteines (Cys), minimizing non-specific interactions with the Cys present in SDE2_UBL_ sequence^37,38^. Indeed, unlike other UBLs which either lack Cys or contain a single Cys residue which can be easily mutated, SDE2_UBL_ contains six Cys in its sequence. As a result, attempts to utilise more reactive electrophiles were unsuccessful. Notably, mass spectrometry revealed evidence of MESNA conjugation to these native cysteines following cleavage from the chitin resin, indicated by mass shifts consistent with one or more MESNA adducts (Supplementary Fig. 5). These adducts were successfully removed by DTT treatment following propargylamine conjugation, restoring the expected ABP mass.

To assess the probe reactivity, we incubated HA-SDE2_UBL_-ABP with recombinant wild-type USP5 (USP5^WT^), catalytically inactive USP5 (USP5^C335A^) and USP13 (318-862). A truncated USP13 construct was utilised in place of full-length protein, as recombinantly expressed full-length USP13 is reported to be inactive towards ubiquitin substrates, whereas the truncated USP13 (318-862) variant retains its catalytic activity^39^. HA-SDE2_UBL_-ABP readily reacted with USP5^WT^, evidenced by the formation of a higher molecular weight band by SDS-PAGE analysis, corresponding to the covalently linked ABP-USP5 adduct (Fig. 3C). The catalytically inactive USP5^C335A^ mutant neither reacted with the ABP nor cleaved SDE2_FL_, indicating that both activities are dependent on the cysteine mediated protease activity of USP5. In contrast, USP13 showed no evidence of reactivity with HA-SDE2_UBL_-ABP or SDE2_FL_ processing, suggesting that the cleavage of SDE2_FL_ observed in the active SEC fractions was primarily driven by USP5.

The reactivity of HA-SDE2_UBL_-PA was further assessed against a broader panel of DUBs including the archetypal DUB USP7, as well as DUBs which, like USP5, have been reported to react with both ISG15 and Ub derived ABPs (Supplementary Fig. 6A)^40,41^. While Ub-PA and ISG15-PA reacted with multiple DUBs in this panel, HA-SDE2_UBL_-PA displayed a high degree of selectivity, reacting exclusively with USP5. Both Ub-PA and HA-SDE2_UBL_-PA exhibited rapid labelling kinetics (Supplementary Fig. 6B), with detectable USP5 modification observed after 30 s and near-complete labelling observed within 10 − 30 min. In contrast, ISG15-PA labelled USP5 much more slowly, with a substantial fraction of unmodified USP5 remaining even after 60 min incubation. This reduced reactivity may be explained by the truncated nature of ISG15-PA, which contains a single ubiquitin-like lobe compared to native ISG15 which instead contains two lobes^42^. Despite its slower labelling kinetics, ISG15-PA formed a more thermally stable complex with USP5, as indicated by a thermal shift assay. The melting temperature (T_m_) of the USP5-ISG15-PA complex was higher T_m_ than that of USP5-SDE2_UBL_-PA complex (57.6 °C ± 0.2 vs 56.4 °C ± 0.2, respectively, Supplementary Fig. 6C). In comparison, the USP5-Ub-PA complex formed the most stable complex (58.4 °C ± 0.2), potentially reflecting USP5’s canonical role of processing Ub chains.

We next performed activity-based profiling in HEK293 cell lysate to determine whether the lack of reactivity observed for certain DUBs, such as USP13, could reflect the requirement for additional components, such as cofactors, that facilitate reaction with the probe but are absent under the in vitro assay conditions. Incubation of the lysate with the HA-SDE2_UBL_-ABP followed by HA immunoprecipitation enabled the isolation of covalently labelled proteins (Fig. 3D). Immunoblotting with a specific α-USP5 antibody revealed a distinct band shift upon ABP treatment, indicating near complete reaction with endogenous USP5. The shifted band was also detected with α-HA antibody, confirming the formation of the HA-SDE2_UBL_-USP5 complex. No labelling of USP13 was observed, consistent with our recombinant protein data and previously reported literature using Ub and UBL derived ABPs^43^.

To further validate the identity of the probe-bound proteins, we performed a tryptic digestion and mass spectrometry analysis on the ABP-enriched material. Proteomics revealed strong enrichment of peptides specific for USP5 and SDE2, confirming the formation of a covalent HA-SDE2_UBL_-USP5 complex (Fig. 3E). Although some USP13-related peptides were detected, these corresponded to regions of shared homology with USP5. Critically, no USP13-specific peptides were identified, reinforcing the conclusion that USP5—but not USP13—directly interacts with SDE2_UBL_ in cells. Moreover, the proteomic analysis did not reveal binding of the probe to other DUBs, indicating a high level of selectivity for USP5.

Although an indispensable tool, HA-SDE2_UBL_-PA does not fully recapitulate the structure of SDE2_FL_. Therefore, to directly determine if full-length USP13 can cleave SDE2_FL_, we transiently over-expressed N-terminally Green Fluorescent Protein (GFP) tagged USP13 (GFP-USP13) and USP5 (GFP-USP5) in Expi293 mammalian cells. The enzymes were isolated from cell lysate using α-GFP immunoaffinity beads and their activity was assessed using an on-bead cleavage assay. Here, bead-bound DUBs were incubated with recombinant SDE2_FL_, then centrifuged to separate the beads from the supernatant which contains the cleavage product. The beads and supernatant were subsequently analysed by SDS-PAGE and immunoblotting. GFP-USP13 failed to cleave recombinant SDE2_FL_, whereas GFP-USP5 retained the activity observed for bacterially expressed USP5 (Fig. 3F). Furthermore, while both enzymes could process K63-linked diUb, only GFP-USP5 reacted with an SDE2_UBL_-derived ABP (Supplementary Fig. 7). Together with the IP-MS results, these data support the conclusion that mammalian USP13 is inactive against SDE2_FL_ and related ABP.

When considered together, these results provide strong biochemical and cellular evidence that USP5 is the primary protease responsible for SDE2_UBL_ cleavage, and they establish HA-SDE2_UBL_-PA as a selective and effective probe for USP5 activity.

### Knockdown cell assay confirms that SDE2 is selectively processed by USP5

**Fig. 4:**
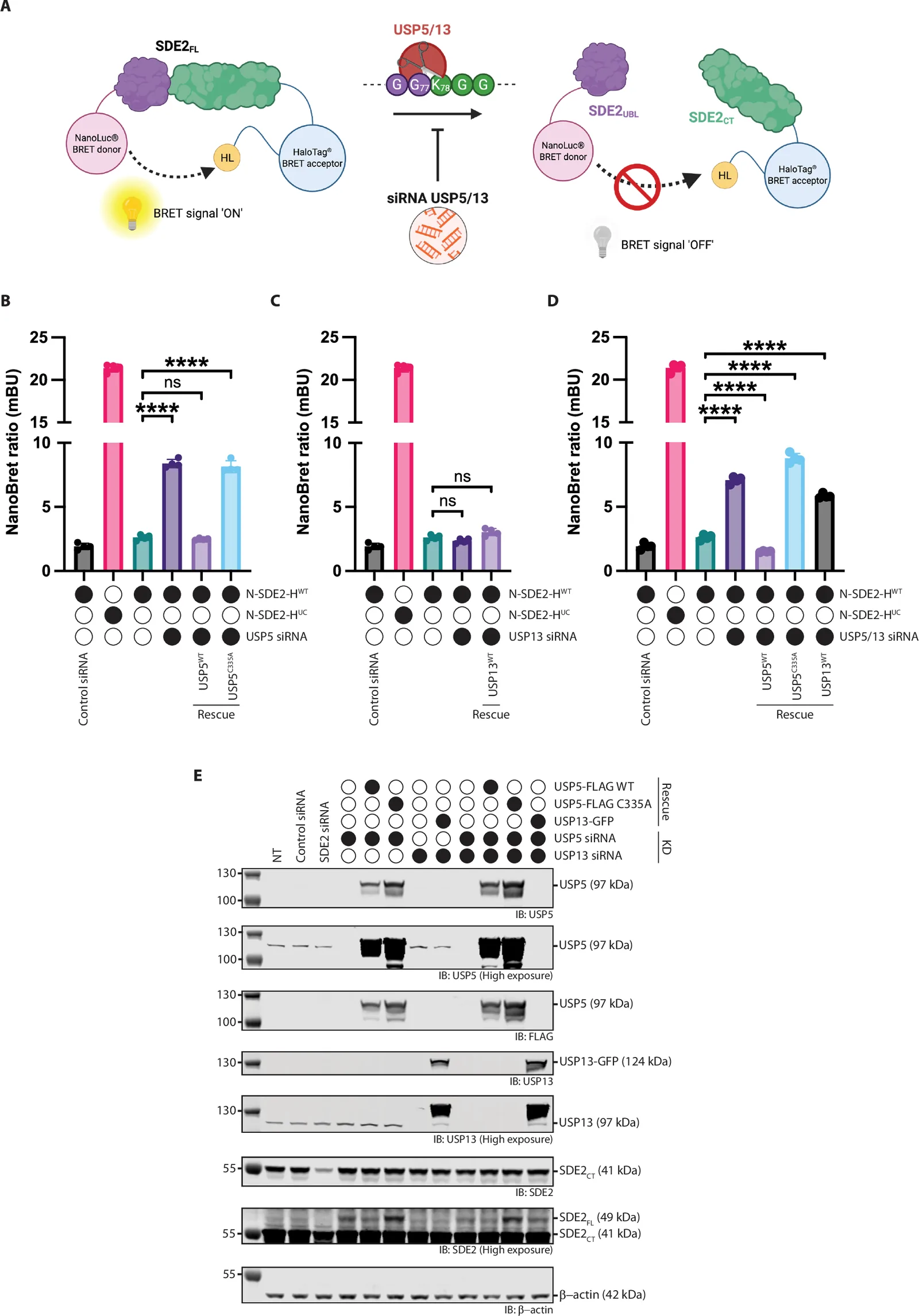
siRNA knockdown confirms USP5 is solely responsible for SDE2 processing at both overexpression and endogenous levels. **A** Schematic representation of a NanoBRET^®^ intracellular SDE2_FL_ cleavage assay. SDE2_FL_ is tagged with an N-terminal NanoLuc^®^ (N-SDE2, donor) and a C-terminal HaloTag^®^ (SDE2-H, acceptor). Upon addition of HaloTag^®^ 618 Ligand, if N-SDE2-H is processed, the N-SDE2_UBL_ and SDE2_CT_-H fragments will separate, and no BRET signal will be detected. If the processing is impaired, the donor and acceptor tags will be in close proximity, so the dipole-dipole non-radiative energy transfer from the luciferase energy donor to the acceptor fluorophore will lead to emission of a light signal (618 nm). Created in BioRender. Bryce, R. (2026) https://BioRender.com/hwy1v2m. Calculated NanoBRET^®^ ratio in experimental samples of HEK293 cells treated with USP5 siRNA (**B**), USP13 siRNA (**C**) or combination of both siRNA (**D**) and relative rescues. WT wild-type, UC uncleavable. Graph depicts the mean calculated NanoBRET^®^ ratios (dots) + SEM of three independent biological replicates (bar). One way ANOVA was performed to calculate *P* values, and levels of significance are denoted as follows: ****P < 0.0001, ns = not significant. **E** HEK293 cells were treated with siRNA targeting USP5, USP13, SDE2, or control siRNA for 48 h. A not treated control (NT) was also included. Rescue experiments were also conducted by exogenously expressing USP5^WT^, USP5^C335A^ or USP13. The data is a representative example of experiments performed as three biological replicates. KD knockdown. Source data are provided as a Source Data file.

To determine whether the proteolytic selectivity observed in vitro extends to a cellular context, we developed a sensitive, real-time assay to monitor SDE2 processing dynamics in live cells using the bioluminescence resonance energy transfer (NanoBRET^®^) technology^44^. In this system, SDE2_FL_ was overexpressed as a fusion protein with an N-terminal NanoLuc^®^ luciferase tag, acting as an energy donor, and a C-terminal HaloTag^®^ as an energy acceptor (N-SDE2-H^WT^). When intact, for example in the case of impaired processing, upon treatment with a fluorophore-containing chloroalkene ligand, the proximity of the donor and acceptor allows for efficient dipole-dipole energy transfer from the luciferase (NanoLuc^®^) to a fluorescently labelled HaloTag^®^, resulting in a detectable BRET signal. Upon proteolytic cleavage of N-SDE2-H^WT^, the donor and acceptor are separated, leading to a measurable loss of energy transfer and thus a decrease in BRET signal (Fig. 4A).

To validate the system and provide a positive control, we engineered an uncleavable variant (N-SDE2-H^UC^) in which the conserved cleavage -GGK- motif was mutated to -AAK- ensuring that the donor and acceptor remain in close proximity and the BRET signal remains constant and high.

Using this assay, we tested the contribution of USP5 and USP13 to N-SDE2-H^WT^ processing via siRNA-mediated knock down (Fig. 4B–D and Supplementary Fig. 8). HEK293 cells were used due to their high transfection efficiency and compatibility with NanoBRET^®^ and RNA interference approaches, allowing efficient perturbation and rescue of candidate proteases. Depletion of USP5 resulted in a significant increase in BRET signal (****P < 0.0001) consistent with reduced N-SDE2-H^WT^ processing. Cleavage was restored upon exogenous reintroduction of wild type USP5 (USP5^WT^), but not by a catalytically inactive USP5^C335A^ mutant (Fig. 4B). In contrast, USP13 knockdown alone had no significant effect, suggesting that USP13 does not play a major role in SDE2 processing under these conditions. Consistently, overexpression of wild type USP13 (USP13^WT^) following either USP5 or USP13 depletion did not decrease the NanoBRET^®^ signal (Fig. 4C). Simultaneous knockdown of both USP5 and USP13 resulted in an increase of NanoBRET^®^ signal; however, cleavage was rescued only by reintroduction of USP5^WT^ and not by USP5^C335A^ or USP13^WT^ (Fig. 4D). Similar experiments were also performed in HeLa and MCF-7 cells, which have been widely used in previous studies of SDE2, DUBs and related pathways^18,43^, yielding similar results (Supplementary Fig. 9A-C). Together, these findings demonstrate that USP5 is the primary protease responsible for SDE2_UBL_ cleavage in cells and that this activity occurs independently of USP13.

To ensure that the observed effects were not an artefact of SDE2 overexpression, we analysed the processing of endogenous SDE2_FL_ by immunoblotting in HEK293 cells treated with siRNA targeting USP5, USP13 or both (Fig. 4E). Importantly, these cells express high levels of endogenous SDE2 and relevant DUBs, enabling assessment of SDE2 cleavage dynamics in a cellular context. Consistent with the NanoBRET^®^ assay, knockdown of USP5 consistently impaired SDE2_FL_ processing, as indicated by the accumulation of SDE2_FL_. Reintroduction of USP5^WT^ restored cleavage and reduced SDE2_FL_, whereas USP5^C335A^ failed to do so. In contrast, USP13 knockdown did not affect SDE2 cleavage, and no change was observed upon reintroduction of USP13. Cells subjected to combined USP5/USP13 knockdown also displayed accumulation of SDE2_FL_; however, this phenotype was rescued only by reintroduction of USP5^WT^ and not by USP5^C335A^ or USP13^WT^. For comparison, we also performed the same experiment in U2OS, which have previously been used as model in related assay, yielding consistent results. A similar phenotype was observed in HeLa and MCF-7 cells (Supplementary Fig. 9D-E). These results further confirming that USP5 is solely responsible for SDE2_FL_ cleavage across multiple cell lines.

### SDE2_UBL_ mimics ubiquitin to engage two binding sites on USP5

**Fig. 5:**
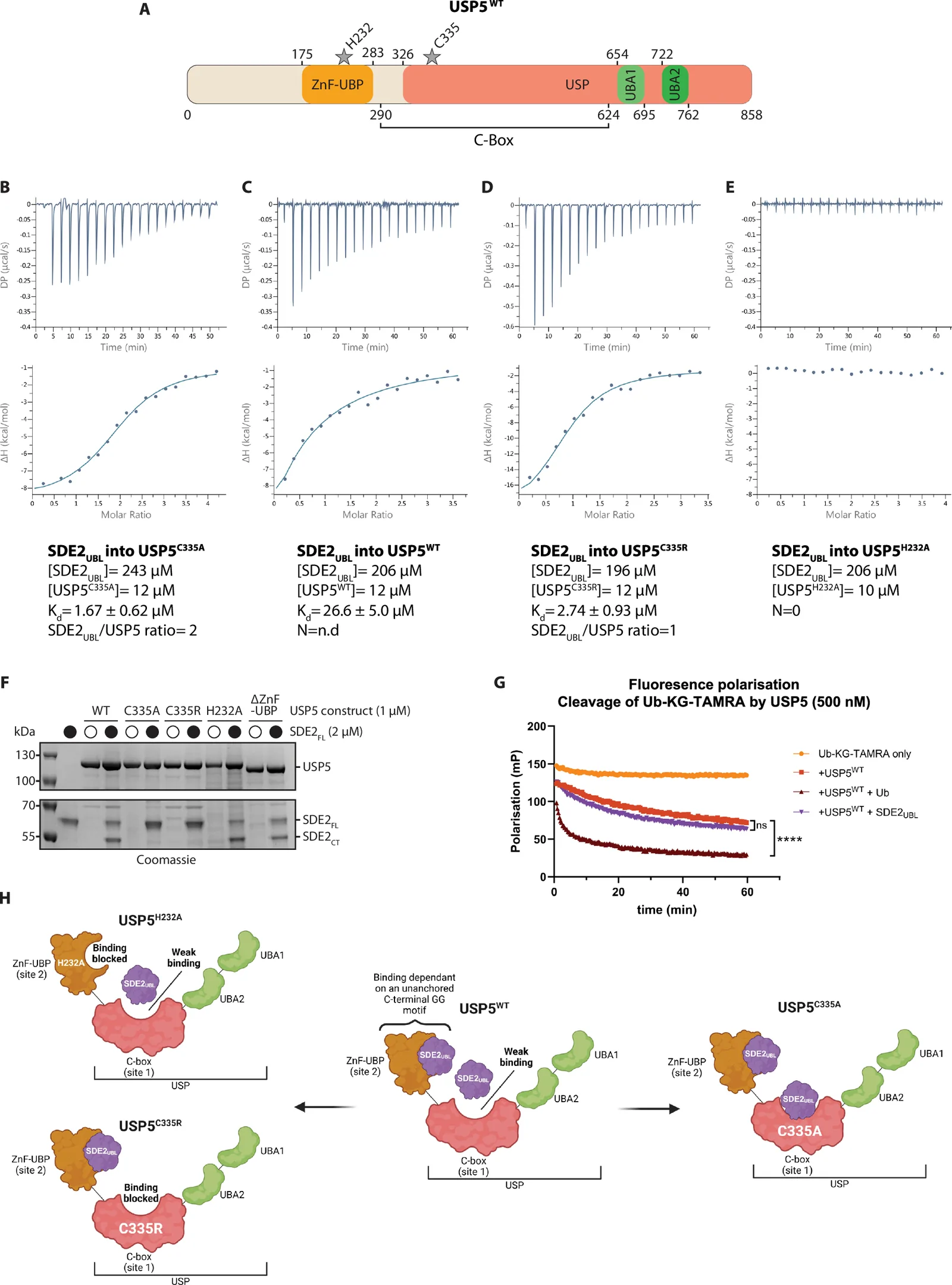
Biophysical, biochemical and structural assessment of the interaction between USP5 and SDE2 confirms that SDE2_UBL_ drives the interaction and can bind at two sites on USP5. **A** Domain structure of USP5^WT^ including amino acid range of zinc-finger ubiquitin binding domain (ZnF-UBP), ubiquitin specific protease (USP) domain, and two ubiquitin associated (UBA) domains. **B–E** ITC analysis of binding between USP5 constructs and SDE2_UBL_. SDE2_UBL_ was titrated into (B) USP5^C335A^, (C) USP5^WT^, (D) USP5^C335R^ or (E) USP5^H232A^. Isotherm curves are representative of duplicate experiments. The *K*_d_ is presented as the mean + SEM of the two experiments. Stoichiometry was inferred from N value, see Supplementary Table 1. **F** Assessment of SDE2_FL_ cleavage by USP5 constructs. USP5 variants (1 µM) were incubated with SDE2_FL_ (2 µM) for 15 min at 37 °C and analysed by SDS-PAGE and Coomassie staining. **G** Fluorescence polarisation cleavage assay demonstrates that Ub, but not SDE2_UBL_, activates USP5^WT^. Ub-KG-TAMRA (100 nM) was incubated with USP5^WT^ (500 nM) for 1 h at 37 °C, either alone or in the presence of Ub or SDE2_UBL_ (500 nM). One way ANOVA was performed between final polarisation values to calculate *P* values, and levels of significance are denoted as follows: ****P < 0.0001, ns not significant. Source data are provided as a Source Data file. **H** Proposed model of SDE2_UBL_ binding to USP5. SDE2_UBL_ can bind either site 1 within the C-box of the USP domain, or at site 2 formed by the ZnF-UBP. The affinity of these sites can be altered by single residue mutagenesis (C335 for site 1 and H232 for site 2) and binding at site 2 is dependent on SDE2_UBL_ containing an unanchored GG motif at the C-terminus. Created in BioRender. Maniaci, C. (2026) https://BioRender.com/3jvkwf1.

Canonically, USP5 is known for its role in processing unanchored polyubiquitin chains, a function that is critical for maintaining the pool of free Ub and ensuring proper Ub signalling dynamics^45,46^. Recognition of polyubiquitin chains is facilitated by a complex and flexible domain structure (Fig. 5A, Supplementary Fig. 10), consisting of a N-terminal ubiquitin-binding zinc-finger (ZnF-UBP) followed by a large USP catalytic domain^33,47^. The USP domain contains the catalytic Cys335 within the conserved C-box motif, as well as two ubiquitin associated (UBA) domains that contribute to the recognition of tri- and tetraubiquitin chains^46^. Whilst the structural and biophysical basis of Ub recognition by USP5 has been extensively characterised, comparatively little is known about its interaction with other substrates^33,47^.

We hypothesised that SDE2 is recognised by USP5 through a mechanism of substrate mimicry mediated by its UBL domain. In an in vitro cleavage assay, we observed that USP5 processed recombinant Ub substrates more rapidly than SDE2_FL_ (Supplementary Fig. 11), achieving near complete processing of di- and tetraubiquitin chains within 120 s. In contrast, cleavage of SDE2_FL_ proceeded more slowly and remained incomplete even after two hours incubation, suggesting distinct substrate engagement dynamics. Notably, processing of recombinant SDE2_FL_ appeared to plateau after 30 min, in contrast to the complete processing observed for cellular SDE2_FL_ (Fig. 3A).

To investigate whether these differences reflected altered substrate recognition and to test the potential role of Ub mimicry, we examined the interaction between SDE2 and USP5 using isothermal titration calorimetry (ITC). The results are summarised in Supplementary Table 1. In the first instance, a catalytically inactive USP5^C335A^ was used to prevent cleavage of SDE2_FL_ during the experiment and to enable direct comparison of binding between USP5 and the individual domains, SDE2_UBL_ and SDE2_CT_. Among the constructs tested, SDE2_UBL_ bound to USP5^C335A^ with the highest affinity (*K*_d_ = 1.67 ± 0.62 µM, Fig. 5B), consistent with the presence of a specific recognition interface. In contrast, SDE2_FL_ showed only weak, non-quantifiable binding while titration of the SDE2_CT_ produced no detectable interaction, indicating that this region does not contribute to binding (Supplementary Fig. 12). In comparison, Ub binds to USP5 with a low nanomolar *K*_d_ (11.7 ± 9.1 nM, Supplementary Fig. 13), in line with previously reported values^47^. In an orthogonal assay, we used time resolved fluorescence energy transfer (TR-FRET) assay where Cy5 labelled SDE2 or Ub constructs were titrated into His-USP5^C335A^ in presence of a europium (Eu) labelled α-His antibody, leveraging a proximity dependent FRET interaction between Cy5 and Eu to detect binding. Consistent with the ITC data, SDE2_UBL_ showed robust binding to USP5^C335A^ while SDE2_FL_ and SDE2_CT_ exhibited no appreciable interaction (Supplementary Fig. 14).

Given that SDE2_UBL_ was the only construct that exhibited quantifiable binding, we next focused on defining the molecular and structural determinates governing SDE2_UBL_ recognition by USP5, particularly in relation to established Ub-binding features. We first compared USP5^WT^ and USP5^C335A^, as mutation of the catalytic Cys to an Ala has been previously shown to increase the affinity of several DUBs for Ub^48^. We therefore asked whether a similar change in affinity would be observed for USP5. ITC measurements revealed that SDE2_UBL_ bound to USP5^WT^ with modest affinity (Fig. 5C, *K*_d_ = 26.6 ± 5.0 µM), whereas affinity to USP5^C335A^ was ~16-fold greater (Fig. 5B, *K*_d_ = 1.67 ± 0.62 µM, *n* = 2). Additionally, the ITC results indicated a stoichiometry of two SDE2_UBL_ molecules per USP5^C335A^ molecule. A comparable enhancement in binding affinity was observed when Ub was titrated into USP5^WT^ and USP5^C335A^ using ITC (Supplementary Fig. 13) and TR-FRET (Supplementary Fig. 14E), consistent with previous reports^48^. In contrast, titration of SDE2_UBL_ into USP5^C335R^ retained overall binding affinity (Fig. 5D, *K*_d_ = 2.15 ± 0.55 µM, *n* = 1), but altered the binding stoichiometry to a 1:1 interaction. This suggests that the bulky Arg substitution disrupts SDE2_UBL_ binding, likely at the C-box binding site as previously described for Ub^48^. These parallels indicate that SDE2_UBL_ and Ub share a common interaction mode and likely occupy similar binding pockets on USP5.

We next considered the importance of the USP5 ZnF-UBP domain for the recognition and processing of SDE2. Previous studies have shown that this domain is essential for binding polyubiquitin chains by recognising the proximal Ub monomer. This interaction facilitates the catalytic activation of USP5 toward these polyubiquitin substrates via engagement unanchored C-terminal GG motifs, either present within these polyubiquitin chains, or contained on separate molecules of monoubiquitin. The structure and function of the ZnF-UBP is reliant on the coordination of a Zn^2+^ ion by four key residues (C199, C202, C219 and H232). Consequently, a H232A mutation is expected to abrogate protein-protein interactions with the ZnF-UBP, as reported for Ub^47^. Accordingly, ITC measurements revealed a complete loss of detectable SDE2_UBL_ binding for the USP5^H232A^ mutant (Fig. 5E) and for a truncated USP5 variant lacking the ZnF-UBP entirely (USP5^ΔZnF^), consistent with previous observations for Ub^47^. Surprisingly, both USP5^H232A^ and USP5^ΔZnF^ successfully cleaved SDE2_FL_ in vitro (Fig. 5F) despite the lack of detectable affinity for SDE2_UBL_. This was recapitulated in cells using the NanoBRET^®^ assay described earlier. Following USP5 knockdown, the transfection of truncated USP5 constructs lacking either the ZnF-UBP domain (ΔZnF), or both UBA domains (ΔUBA1/2) rescued SDE2 processing (Supplementary Fig. 15). These findings indicate that high affinity interaction with the SDE2_UBL_ domain is not strictly required for catalytic cleavage. Interestingly, removal of terminal Gly77 from SDE2_UBL_ reduced binding affinity towards both USP5^WT^ and USP5^C335A^ (Supplementary Table 1), indicating that recognition by the ZnF-UBP depends on the recognition of the GG motif. Despite these similarities, we observed no evidence that the presence of SDE2_UBL_ activates USP5 catalytic activity, in contrast to Ub which enhances USP5 catalytic activity against Ub substrates (Fig. 5G)^45,47^. Together, these results suggest that the ZnF-UBP domain provides a higher affinity binding site for SDE2_UBL_ than the catalytic C-box site, but does not stimulate catalytic activation in the same manner as Ub.

As noted above, we consistently observe incomplete cleavage of recombinant SDE2_FL_ in our in vitro experiments (Fig. 5F and Supplementary Fig. 11). Furthermore, in cellular assays proteosomal inhibition using MG132 resulted in accumulation of SDE2_UBL_ and a reduction of SDE2_FL_ cleavage, suggesting a potential mechanism of product inhibition (Supplementary Fig. 16 A). We therefore considered whether the ZnF-UBP domain may contribute to a mechanism of product inhibition via SDE2_UBL_ binding. To test this hypothesis in vitro, we performed SDE2_FL_ cleavage assays in the presence of excess Ub or SDE2_UBL_. Comparison of the initial reaction rates and maximal reaction progression revealed no significant differences between conditions (Supplementary Fig. 16 B-C) indicating that neither product inhibition nor catalytic activation accounts for the reduced processing observed in the recombinant system^47^.

Finally, to obtain structural insight into the native SDE2_UBL_-USP5^WT^ binding interaction and to compare this to Ub-USP5^WT^ binding, we employed hydrogen-deuterium exchange mass-spectrometry (HDX-MS). Deuterium exchange profiles for *apo* USP5^WT^ were compared with those obtained in the presence of either Ub or SDE2_UBL_. A sequence coverage of 85% was obtained (Supplementary Fig. 17), with minor coverage gaps within the catalytic domain (C335-Q342 and S374-381) and larger gaps within domain linker regions (A702-T729 and S761-D773). Peptides observed to have significant changes in solvent exchange rate upon complex formation were mapped onto the AlphaFold 3 model of USP5^WT^ bound to two molecules of either Ub or SDE2_UBL_ (Supplementary Fig. 18, Supplementary Table 2), consistent with the apparent stoichiometry observed by ITC^30^. In both cases, the affected peptides largely overlapped, supporting a shared binding mode. Binding of either SDE2_UBL_ and Ub reduced exchange within peptides C219-E235 (located within the ZnF-UBP and containing two of the three key Zn chelating residues) as well as S156-W169 (which flank the ZnF-UBP region). These data are consistent with ligand engagement at the ZnF-UBP site. Peptide F439-N445 within the C-box exhibited an increase in HDX in the presence of both Ub and SDE2_UBL_, suggesting possible structural changes in the C-box binding region associated with binding. Interestingly, reduced exchange was also observed within the UBA2 domain (I730-A752), potentially reflecting an interaction between UBA2 and an SDE2_UBL_/Ub molecule bound at the C-box binding site, in agreement with previous structural studies of USP5^33^.

Combining these biophysical, biochemical and structural data with AlphaFold 3 modelling, we propose that USP5 contains two binding sites capable of recognising free SDE2_UBL_ in a manner analogous to Ub (Fig. 5H). The first site corresponds to the C-box within the USP domain (site 1), where mutation of C335 modulates an intrinsically low affinity interaction with SDE2_UBL_. The second site is the ZnF-UBP (site 2), which forms a higher-affinity interaction dependent on Zn^2+^ coordination and the recognition of terminal GG motif of SDE2_UBL._ We therefore speculate that these two sites play unique roles in the processing of SDE2_FL_.

### USP5-catalysed SDE2_FL_ processing modulates RNA splicing patterns

Upon proteolytic removal of its N-terminal UBL domain, the C-terminal fragment of SDE2 becomes incorporated into the spliceosome, where it contributes to the efficient removal of specific introns from a subset of pre-mRNAs^18^. This activity is thought to be mediated through interactions with spliceosomal components, including SF3B1 and U2AF within the U2 small nuclear ribonucleoprotein (snRNP) complex, as well as Cactin/Cay1 complex, which is critical for docking to the 3’ splice site^18^. This function is particularly important for the expression of genes involved in DNA replication and cell cycle progression. Consequently, proteolytic processing of SDE2 represents a key regulatory step linking replication stress responses with spliceosome function. Consistent with this mode, siRNA-mediated knock down of SDE2 has been shown to impair the excision of selected introns from a subset of pre-mRNAs in both mammalian cells and *Schizosaccharomyces pombe*. Moreover, structural analysis of the human post-catalytic spliceosome revealed that the N-terminal ubiquitin domain of unprocessed SDE2 would sterically clash with the branch helix, suggesting that SDE2_UBL_ removal is required for proper spliceosome assembly and function^23^.

**Fig. 6:**
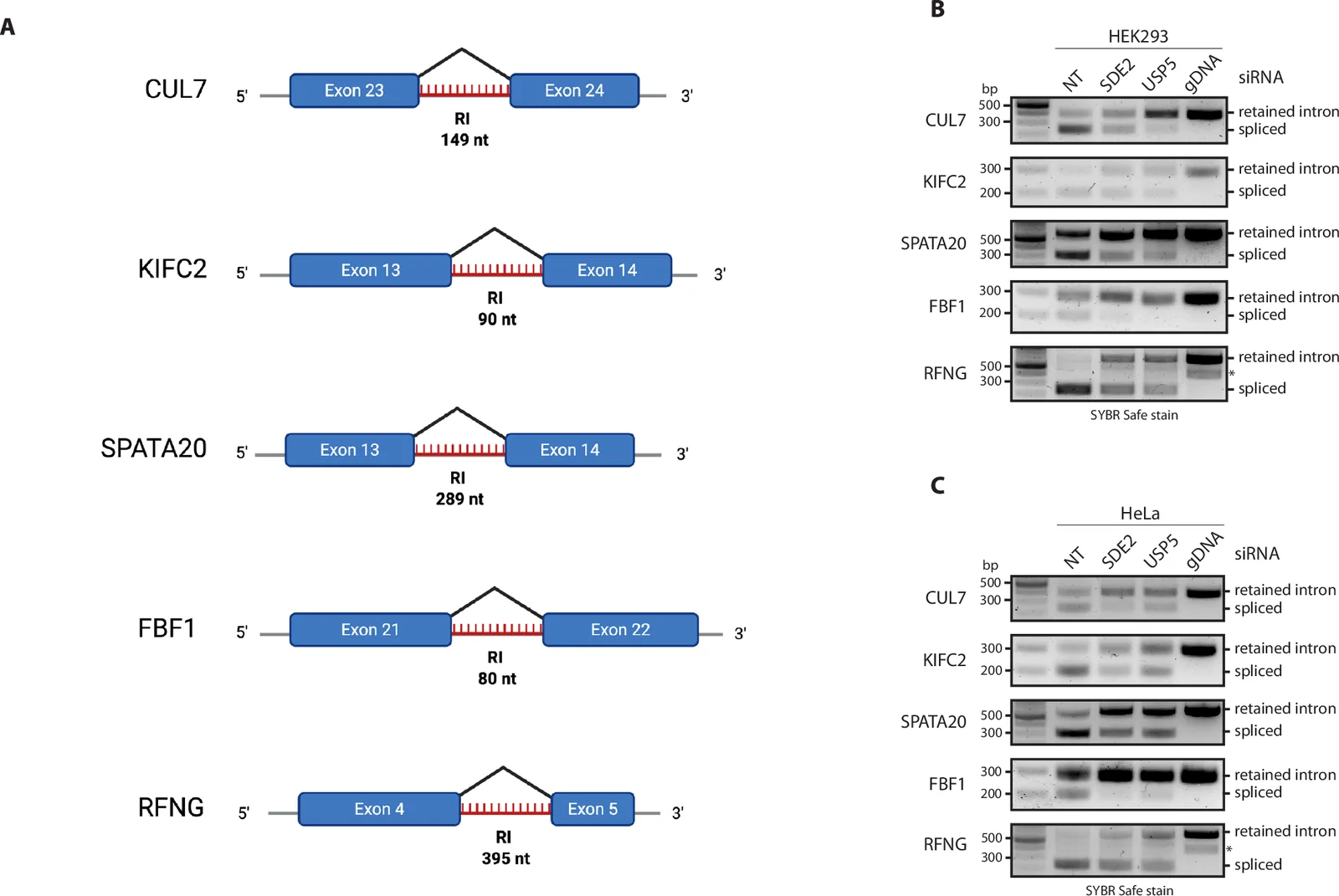
USP5-catalysed SDE2_FL_ processing modulates SDE2 biology. **A** Schematic representation of intron retention upon SDE2 depletion. Exons are visualised in blue and retained introns in red with the expected length in nucleotides annotated. RI= retained intron. Created in BioRender. Maniaci, C. (2026) https://BioRender.com/pg22rc6. Agarose gel analysis of intron retention events following RT-PCR. RNA was extracted from either HEK293 (**B**) and HeLa (**C**) following siRNA-mediated depletion of either SDE2 or USP5. mRNAs for target genes were reverse transcribed and the resulting cDNAs were amplified by PCR. PCR products were visualised using SYBR™ Safe DNA stain. RI events and spliced events are indicated. Lane legend: NT non-treated, SDE2 SDE2 siRNA treated, USP5 USP5 siRNA treated, gDNA genomic DNA from wild-type cells.

We therefore hypothesised that USP5 plays a key regulatory role in pre-mRNA splicing by processing SDE2_FL_ to generate the active spliceosomal fragment SDE2_CT_. Because SDE2_UBL_ inhibits SDE2 incorporation into the spliceosome, we further predicted that impaired processing resulting from USP5 knockdown would lead to a defect in intron excision similar to that observed upon SDE2_FL_ depletion. To test this, we treated HEK293 cells with siRNA targeting either SDE2 or USP5 and monitored the changes in intron retention in previously identified SDE2 dependent genes (CUL7, KIFC2, SPATA20, FBF1 and RFNG) by RT-PCR (Fig. 6A)^18^. Efficient knockdown of SDE2 and USP5 was confirmed by immunoblot (Supplementary Fig. 19) and all tested genes exhibited robust increase in intron retention at the reported loci following depletion of either SDE2 or USP5, linking the role of SDE2 in spliceosome function to the requirement for the removal of SDE2_UBL_ domain by USP5 (Fig. 6B). To ensure these events were not cell line specific, we repeated the knockdown and analysis in HeLa cells (Fig. 6C). Consistent with the data in HEK293 cells, impaired SDE2 processing in HeLa also led to a significant increase in intron retention in the tested genes. Together, these results demonstrate that efficient spliceosome function requires proteolytic removal of the SDE2_UBL_ domain by USP5.

## Discussion

The biological function of proteins is regulated by an intricate network of post-translational chemical and enzymatic modifications, including phosphorylation and ubiquitination^1^. Among these, proteolysis represent a unique and irreversible regulatory mechanism. Historically, it has largely been viewed through the lens of degradation, serving to remove misfolded or damaged proteins or to maintain cellular homoeostasis through pathways such as the ubiquitin-proteasome and caspase-mediated apoptosis^49^. However, recent advances in degradomics have fundamentally reshaped this perspective. These studies reveal that proteolysis also function as a highly selective and instructive regulatory mechanism, modulating protein activity, localisation and interaction across a wide range of biological pathways, from signal transduction to organelle biogenesis^50^.

Within this broader regulatory framework, the nuclear protein SDE2 presents a compelling case study. Given its involvement in fundamental processes, the activity of SDE2 must be stringently regulated. A key regulatory step lies in its proteolytic processing whereby removal of the N-terminal UBL domain is required to liberate the functional C-terminal fragment, which mediates the downstream biological function of SDE2. At the same time, this processing event also regulates SDE2 homoeostasis, as cleavage exposes an N-terminal lysine that targets SDE2_CT_ for proteasomal degradation through the N-end rule pathway. Yet, despite the importance of this cleavage event, the identity of the responsible protease and proteolytic mechanism in humans has remained unresolved.

In this study, we identify USP5, a protease previously considered to predominantly process Ub substrates, as the principal cysteine-dependent protease responsible for the site-specific cleavage of SDE2_FL_ immediately downstream to its UBL domain. Through a combination of activity-based probe profiling, quantitative binding analysis, and cellular assays, we demonstrate that SDE2 is exclusively cleaved by USP5 both in vitro and in cell through a specific interaction mediated by SDE2_UBL_ domain. Impaired SDE2 processing following USP5 knockdown confirmed the specificity of this mechanism and its dependence on USP5 catalytic activity. Indeed, only reintroduction of catalytically active USP5 rescued SDE2 cleavage in USP5 depleted cells, whereas no other DUBs tested displayed detectable activity towards SDE2_FL_. The observation of a similar phenotypes across multiple cell lines further supports the generality of this mechanism.

Except for the structural similarities between the UBL domain and Ub, SDE2 differs substantially from the other known USP5 substrates. Therefore, our finding that USP5 can also cleave SDE2 expands the substrate repertoire of USP5 and raises important questions regarding the mechanistic, structural and biological basis of this previously unrecognised activity.

Like other USP family members, USP5 conventionally catalyses the hydrolysis of isopeptide bonds between Ub chains and substrate proteins. However, unlike most DUBs, USP5 plays a specialised role in maintaining the cellular pool of free Ub by preferentially disassembling unanchored polyubiquitin chains into monomeric Ub^33,47^. Comparative assays showed that while Ub remains the preferred substrate, reflected by the observed rapid and complete cleavage of Ub₄, SDE2 is also efficiently processed by USP5, with approximately 50% cleavage observed within 5 minutes. Moreover, an SDE2 derived probe, HA-SDE2_UBL_-PA, displayed labelling kinetics comparable to an equivalent Ub-ABP and significantly higher than an ISG15-derived probe, another ubiquitin-like modifier known to act as non-canonical USP5 substrate^40^. These findings highlight a previously underappreciated substrate plasticity of USP5, demonstrating its ability to process distant UBLs with less than 20% sequence identity. This suggests that the substrate repertoire of USP5 may extend well beyond classical Ub chains under certain conditions.

Interestingly, we observed differences in the efficiency of SDE2_FL_ processing between recombinant and cellular contexts. While cleavage of recombinant SDE2_FL_ by USP5 and labelling of USP5 by HA-SDE2_UBL_-PA plateaued at approximately 50% across multiple assays conditions, complete processing and labelling were observed in cell lysate. Treatment of cells with proteasome inhibitor MG132 resulted in accumulation of SDE2_UBL_ together with reduced SDE2_FL_ cleavage, suggesting a possible mechanism of product inhibition. However, this phenomenon was not reproduced in vitro when cleavage assays were performed in presence of excess SDE2_UBL_ or Ub, arguing against a simple product inhibition mechanism. It has been previously reported that certain DUBs require cofactors to achieve full catalytic activity^51,52^. It is therefore possible that, unlike for Ub processing, efficient cleavage of SDE2_FL_ by USP5 may depend on an additional cellular cofactor or binding partner that is present in cell lysates but absent in the recombinant system. Such a mechanism could provide a feedback loop regulating SDE2 cleavage in response to cellular conditions, although further studies will be required to elucidate the molecular basis of this regulation.

USP5 activity is enabled by its unique modular domain architecture, which orchestrates substrate engagement, positioning, and catalysis, allowing for the critical recognition of substrates^33,47^. Key elements include a ZnF-UBP domain that recognises the free C-terminal diglycine motif of Ub, and multiple UBA domains that contribute to polyubiquitin chain recognition. The inherent flexibility of the UBA domains, tethered to the catalytic core via disordered linkers, enables USP5 to accommodate and process a wide range of Ub chain architectures, including K48-, K63-, K11-, and M1 linked chains^45,53^. Notably, although USP5 efficiently processes unanchored polyubiquitin chains, there are relatively few reports describing the deubiquitination of polyubiquitinated substrates^54–57^ with only one example confirmed in vitro^58^. Moreover, in vitro screening of USP5 against ubiquitinated peptides has revealed inactivity against isopeptidic linkages between ubiquitin and its substrate^59^. The ability of USP5 to recognise and cleave the peptidic linkage between SDE2_UBL_ and SDE2_CT_ therefore highlights a previously unappreciated functional versatility of this enzyme^45,53^. Using orthogonal biophysical techniques including isothermal titration calorimetry (ITC) and TR-FRET, we investigated the interaction between USP5 and various SDE2 domains. These experiments revealed minimal binding affinity for SDE2_FL_ and SDE2_CT_, whereas SDE2_UBL_ exhibited a binding behaviour closely resembling the interaction between USP5 and monoubiquitin. Mutational analysis, domain truncation and HDX-MS further highlighted the similarities between SDE2_UBL_ and Ub. HDX-MS achieved only partial sequence coverage of USP5^WT^ (85%), however the observed deuterium exchange profiles were found to be similar in the presence of either SDE2_UBL_ or Ub. Although further work is required to understand the molecular determinants of binding, our data suggest that SDE2_UBL_ engages USP5 at two sites in a comparable manner to Ub, with the first of these sites within the C-box (site 1), which contains the catalytic cysteine C335^33,46^. The second site would correspond to the ZnF-UBP domain (site 2), which recognises the free C-terminal GG motif, aligning with its canonical function for Ub binding. Although removal of the ZnF-UBP of USP5 reduced SDE2_UBL_ binding to below detection limits, this did not abolish SDE2 processing. This indicates that while an intact and flexible ZnF-UBP domain is important to accommodate a large array of possible substrates, binding at this site is not essential for catalytic activity. Additionally, we have observed that the interaction between SDE2_UBL_ and the ZnF-UBP does not enhance USP5 catalytic activity in the same way as Ub despite the similarities in binding, indicating that SDE2 acts as a unique pseudo-substrate. Based on these observations, we propose a model (Fig. 5H) in which two USP5 binding sites fulfil distinct roles. Site 1 mediates substrate positioning and cleavage by orienting the UBL domain of SDE2_FL_ with its GG motif adjacent to C335, whereas site 2 recognises cleaved SDE2_UBL_ molecules containing a terminal GG motif. Interestingly, site 1 does not appear to rely upon high affinity recognition of either SDE2_FL_ or SDE2_UBL_ alone. In contrast, site 2 may be important for appropriate enzymatic regulation. Other ZnF-containing DUBs have been shown to rely on ZnF-UBP domains to relieve product inhibition, facilitate protein-protein interactions, or, as is the case for USP5, modulate catalytic activation^60,61^. It is therefore likely that SDE2_UBL_ binding to the ZnF-UBP plays an important role in regulating cleavage, although the precise functional consequences remain to be determined.

Alongside USP5, we also examined close paralog USP13, as a candidate for SDE2 processing. Despite sharing high sequence similarity (67.3% at the nucleotide level, 54.8% at the amino acid level) and near identical domain structure, USP13 expressed in either bacterial and mammalian systems showed minimal catalytic activity toward SDE2 in recombinant and cellular assays. HA-SDE2_UBL_-PA covalently labelled USP5 but not USP13, and knockdown of USP5, but not USP13, impaired SDE2 processing in cells. These results support a functional divergence between USP5 and USP13 and reinforce the substrate specificity of USP5 toward SDE2_UBL_. It has been reported that USP13 might exist in a constitutive self-inhibition state possibly supported by the interaction of UBA with its ZnF domain^39,62^. It has been hypothesised that this self-inhibition status may be released by recruitment of other proteins or modification, such as phosphorylation. Therefore, we cannot exclude the possibility that under certain stimuli, USP13 may process SDE2, however our data strongly suggests that USP5 is the primary effector of SDE2 processing.

Functionally, we have demonstrated that USP5-mediated processing of SDE2_FL_ plays a key regulatory role in pre-mRNA splicing by removing the sterically occlusive SDE2_UBL_ and enabling SDE2_CT_ to be incorporated into the spliceosome. Consistent with this, impairment of SDE2 processing following USP5 knockdown resulted in increased intron retention and altered pre-mRNA splicing patterns. Genes co-expressed with USP5 in pan-cancer are known to be strongly associated with spliceosome and RNA splicing regulation, suggesting a potential functional convergence with SDE2-associated cellular pathways^63^. Furthermore, although we could not directly demonstrate an effect of USP5 depletion on the DNA damage response functions of SDE2, USP5 has previously been implicated in homologous recombination and other DNA damage response pathways, which is consistent with the previously reported roles of SDE2 in genome stability^64,65^.

The identification of USP5-SDE2 interaction axis adds to the pool of research which highlights how the cleavage of UBLs is mediated by a suite of proteases with varied substrate specificity and domain organization. UBLs such as SUMO and NEDD8 are deconjugated by distinct families of UBL-specific proteases, including sentrin-specific proteases (SENPs), which display minimal cross-reactivity with Ub^66,67^. Similarly, ISG15 is canonically deISGylated by USP18, a USP family member with no known activity toward Ub^13,68^. However, more recent studies have revealed context-dependent cross-reactivity among DUBs and UBL proteases: USP16 and USP24, for instance, have been shown to deISGylate substrates in specific cellular environments^40,69^. USP16 also cleaves Fubi, a UBL with moderate sequence similarity to both Ub and ISG15 ( ~ 36–38%)^70^, further demonstrating that deubiquitinases can accommodate structurally related yet distinct modifiers. Therefore, our findings that USP5, a protease with canonically ubiquitin-specific activity, proteolytically removes SDE2_UBL_ from full-length SDE2, aligns with a growing body of evidence that DUB-UBL specificity is more nuanced than previously appreciated.

Collectively these findings expand the known substrate repertoire of USP5 to include SDE2 and suggests broader implications for how UBLs may be regulated by non-canonical DUB function. Further investigation into the structural determinants of this specificity will deepen our understanding of both USP5 biology and SDE2-regulated pathways.

## Methods

### Reagents used in this study

A list of plasmids used in this study can be found in Supplementary Table 3. Further information and requests for reagents should be directed to C.M. All complementary DNA constructs in this study were generated by C.M., P.M.T., L.T.H. and the cloning team at the Medical Research Council Protein Phosphorylation and Ubiquitylation Unit (MRC PPU) Reagents and Services. All plasmids were deposited with the MRC PPU Reagents and Services along with sequencing data and are available upon request at https://mrcppureagents.dundee.ac.uk/. ISG15-PA and the pTXB1_His-3C-Ub (1-75) plasmid used in this study were a kind gift from Rafael Salazar Claros of the Swatek lab.

### Cell culture

HEK293 cells (RRID:CVCL_0045), HEK293T (RRID:CVCL_0063), U2OS (RRID:CVCL_0042), HeLa (RRID:CVCL_0030) and MCF-7 (RRID:CVCL_0031) were obtained from the ATCC. HEK293, U2OS and HeLa cells were cultured in high-glucose Dulbecco’s modified Eagle’s medium (DMEM, 4.5 g/L glucose), while MCF-7 cells were cultured in Roswell Park Memorial Institute (RPMI) 1640 medium. Both mediums were supplemented with 10% foetal bovine serum (FBS), L-glutamine, and Penicillin and Streptomycin (Gibco). Cells were maintained at 37 °C in a 5% CO_2_ atmosphere and grown for no more than 30 passages. All cell lines were routinely tested for mycoplasma contamination using MycoAlert kit from Lonza.

*Expi293 culture and protein expression:* Expi293 cells were seeded in complete growth medium (Expi293 Expression Medium (Gibco)) into conical flasks at a density of 1 × 10^6^ cells/well to achieve a density of 2 × 10^6^ cells/well on day of transfection. Cells were culture at 37 °C in a 5% CO_2_ atmosphere in suspension under continuous agitation. For a 250 mL culture, a solution of 250 µg of plasmid was prepared in 10 mL of OptiMEM^®^, and 875 µg of Polyethylenimine (PEI) transfection reagent was added. The resulting solution was mixed carefully and incubated for 15 min at room temperature. Transfection complex was added directly to the media, before incubation for 48 h at 37 °C under continuous agitation.

### Cell lysis

Cells were lysed in RIPA buffer (50 mM Tris pH 8, 150 mM NaCl, 1 mM EDTA, 1% SDS, 1% sodium deoxycholate, 1% NP-40, 50 mM NaF, 5 mM sodium pyrophosphate) supplemented with cOmplete EDTA free protease inhibitor cocktail (Roche). The Petri dishes were placed on ice and the media aspirated. Tissue layer was washed twice with ice-cold phosphate saline buffer (PBS) before adding the lysis buffer. Cells were scraped from the surface and cell extract was collected. Insoluble fraction was removed by centrifugation at 4 °C (25 min, 20,800 × *g*). Protein concentration was measured using Pierce™ Coomassie (Bradford) Protein Assay Kit.

### SDS-PAGE and immunoblotting

Samples for SDS-PAGE and subsequent immunoblotting were heated at 95 °C for ~ 5 min in NuPAGE 1×LDS Sample Buffer (Life Technologies) with DTT (100 mM). SDS-PAGE was performed on 4–12% Tris-Acetate NuPAGE^®^ Novex^®^ (Life Technologies) polyacrylamide gels with 3-(*N*-morpholino) propanesulfonic acid (MOPS) running buffer (ThermoFisher Scientific) at 130 V.

Samples for immunoblotting were transferred to a nitrocellulose membrane using wet transfer (20% methanol, 80 V for 80 min). Membranes were blocked with 5% w/v Bovine serum albumin (BSA) in Tris-buffered saline (TBS) with 0.1% w/v Tween-20. The following antibodies were used in this study in the given concentrations: USP5 (AB154170, Abcam, 1:1000), USP13 (12577S, Cell Signalling, 1:1000), NanoLuc (N700A, Promega, 1:1000), HaloTag (G921A, Promega, 1:1000), SDE2 (HPA031255, 1:1000), HA (3724S, Cell Signalling, 1:1000), Flag (F7425, Sigma, 1:2000), B-actin (4970S, Cell Signalling, 1:1000), GAPDH (2118S, Cell Signalling, 1:1000), GFP (3H9, ChromoTek, 1:2000), IRDye^®^ 800CW Goat anti-Rabbit Secondary Antibody (926-32211, Licor, 1:25,000), IRDye^®^ 800CW Donkey anti-Mouse Secondary Antibody (926-32212, Licor, 1:25,000), Goat anti-Rat IgG (H + L) horseradish peroxidase (HRP) Secondary Antibody (31470, Invitrogen, 1:10,000). Fluorescent detection was performed with a Li-Cor Odyssey CLx scanner. Chemiluminescence was detected with Amersham™ ECL™ Prime Western Blotting Detection Reagents (RPN2232, Cytiva) and performed in ChemiDoc™ MP Imaging System (Bio-Rad). Uncropped blots are presented within the accompanying Source Data file.

### Protein expression

Competent *Escherichia coli* BL21 (DE3) cells were transformed and then used to inoculate a starter culture of Terrific Broth (TB) media (200 mL) supplemented with ampicillin (final concentration 100 µg/mL) which was incubated at 37 °C with shaking (180 rpm) for ~18 h. Expression cultures of TB (1 L, 100 µg/mL ampicillin) were inoculated with starter culture (15 mL), grown at 37 °C 180 rpm in 2.5 L high expression flasks containing 1 L of TB and 100 µg/mL ampicillin to an optical density of 0.6 at 600 nm (OD600). When the OD600 = 0.6 was reached ( ~ 4 h), cells were induced with 100 µM isopropyl-1-thio-β-d-galactopyranoside (IPTG) and left incubating at 18˚C and 240 rpm shaking for 24 h. Cells were pelleted by centrifuging at 4900 × *g* for 30 min at 4 °C, then supernatant decanted and cells resuspended in 50 mL (per 1 L of culture) lysis buffer (50 mM HEPES pH 7.5, 150 mM NaCl, 20 mM imidazole, 18 µM leupectin hemisulfate, 250 µM AEBSF, 1 mM DTT), after which the resuspended pellets were stored at −80 °C.

### Protein purification

Resuspended pellets were lysed using an ultrasonic processor for 5 min (10 s on/ 15 s off, 60% amp) at 4°C. Supernatant was collected by centrifuging at 36,300 × *g* for 30 min at 4 °C. Collected lysate was filtered through a 0.2 µm filter. From there on purification differed for different constructs.

*Expression of USP5*^*WT*^ *and its mutant construct:* Lysate was incubated with Ni-NTA agarose beads (5 mL per 1 L of culture lysate) for 2 h at 4°C on a roller. Loaded beads were captured in chromatography column, washed 10 column volumes (CV) with wash buffer A (50 mM HEPES pH 7.5, 150 mM NaCl, 20 mM imidazole) and eluted with 10 CV of elution buffer A (50 mM HEPES pH 7.5, 150 mM NaCl, 200 mM imidazole). Elution was concentrated down (Amicon^®^ Ultra Centrifugal Filter) to injection volume ( < 5 mL) for size exclusion chromatography (SEC) by gel filtration on a HiLoad 16/600 Superdex 200 pg column (Cytiva) using gel filtration buffer (50 mM HEPES pH 7.5, 150 mM NaCl, 5% glycerol, 1 mM DTT). Fractions containing the USP5 were pooled and concentrated by ultrafiltration. Structural integrity of select mutants was assessed using circular dichroism (Supplementary Fig. 25).

*Expression of SUMO-tagged constructs:* In the case of constructs containing SUMO tag [His-SUMO-SDE2_FL_(1-451), His-SUMO-SDE2_CT_(78–451) and His-SUMO-SDE2_UBL_(1–77)], expressed protein was captured with Ni-NTA agarose beads (2 h, 4˚C), washed in chromatography column with wash buffer A (10 CV), eluted with elution buffer A (10 CV) and set up for overnight dialysis into no imidazole buffer (50 mM HEPES pH 7.5, 150 mM NaCl, 1 mM DTT) containing His-tagged SUMO-protease (1 µg protease per 2 mg of protein). Dialysed solution was passed through fresh Ni-NTA agarose beads to capture SUMO-protease and uncleaved protein. Flowthrough containing the protein of interest was concentrated down to injection volume for gel filtration. SDE2_FL_ and SDE2_CT_ were further purified on HiLoad 16/600 Superdex 200 pg column (Cytiva), while SDE2_UBL_ was purified on HiLoad 16/600 Superdex p75 column (Cytiva) (using gel filtration buffer). Fractions containing the desired construct were pooled, concentrated, flash-frozen, and stored at −80°C.

For HA-SDE2_UBL_(1-77), lysate was incubated with chitin agarose beads (New England) for 3 h at 4˚C on a roller. Loaded resin was washed with 10 CV of wash buffer B (50 mM HEPES pH 7.5, 150 mM NaCl) and then incubated in a cleavage buffer (50 mM HEPES pH 7.5, 150 mM NaCl, 50 mM DTT) over 72 h at 4˚ C. The elution was collected and the resin was further washed with one CV of wash buffer B. Elution was concentrated and gel filtrated with a HiLoad 16/600 Superdex p75 column (Cytiva). Fractions containing POI were pooled, concentrated, flash-frozen, and stored at −80 ˚°C.

SUMO protease was expressed and purified by Ni-NTA affinity as previously described^71^. In short, lysate was incubated with Ni-NTA agarose beads for 2 h at 4 ˚°C, captured on affinity column, washed wash buffer A (10 CV), eluted with elution buffer A (10 CV), concentrated down, and stored in −80 ˚°C.

### Circular dichroism (CD) spectroscopy

CD spectra were collected using a MOS-500 circular dichroism spectrometer (BioLogic) in a 1 mm path length quartz cuvette (Hellma^®^ Analytics) under continuous nitrogen flushing. Measurements were obtained over a wavelength range of 190–350 nm with a spectral resolution of 0.2 nm and acquisition period of 0.5 s, at 20 °C following 5-min equilibration period. Protein samples were first dialysed in 20 mM potassium phosphate buffer (pH 8.0) using D-Tube Dialyzers (Merck, MWCO 11–12 kDa) and diluted to final concentrations of 4.2–10.6 µM. The molar ellipticity ([θ], deg×cm^2^×dmol^−1^) was calculated using $\Delta \varepsilon=\frac{{{{\rm{m}}}}^\circ \times {10}^{-3}}{32980\times {{{\rm{c}}}}\times {{{\rm{l}}}}}$ where the raw CD data is expressed in millidegrees m°, then converted to mean molar ellipticity (Δε), with c being the protein concentration in mol/L, and l the cuvette path length in cm. The secondary structure content of the protein and mutants was estimated using the ChiraKit platform which utilises either the SELCON or SESCA algorithm^72^.

### Lysate fractionation and screening

*Lysis, Heparin and SourceQ fractionation:* Expi293 cells were grown in 100 mL suspension in A14351-01 media until a confluency of 5 × 10^6^ cell/mL. Cells were washed in PBS and resuspended in 5 volumes of hypotonic buffer (50 mM Tris pH 7.5, 7 mM 2-BME, 1 mM TCEP, 1 mM AEBSF, 10 µg/mL Leupeptin), incubated on ice for 20 min before lysis by mechanical stress through a Dounce homogenizer ( > 20 sequential passes through 21-23-Gauge needles). Resulting lysate was cleared by centrifugation (20 min, 36,300 × *g*) and soluble fraction was filtered through 1 Whatman syringe filter (PVDF 0.45 µM). The lysate was buffer exchanged to MOPS-LSB (30 mM MOPS pH 7.0, 10% glycerol, 7 mM 2-BME, 1 mM TCEP, 0.015% Brij35) using a 20 mL Sephadex G25 fine column (XK16/10) using an ÄKTA Pure™ Fast Protein Liquid Chromatography (FPLC) device. The lysate was applied to a 1 mL Heparin HiTrap^™^ HP column (Cytiva) and proteins were eluted with an increased gradient of a high salt buffer (30 mM MOPS pH 7.0, 1.2 M NaCl, 5% glycerol, 1 mM DTT, 0.015% Brij 35) and collected as 1 mL fractions. The pH of the flowthrough from the Heparin chromatography was adjusted to pH 8.0, by addition of 300 µL (1% v/v) of unbuffered 1 M Tris-Base and it was passed through a 1 mL Source^™^ 15Q column (HR10/10, Cytiva) and eluted on a salt gradient (30 mM Tris pH 8.0, 10% glycerol, 7 mM 2-BME, 1 mM TCEP, 0.015% Brij35, 1 M NaCl). SourceQ fractions (1 mL volume) were eluted into a 96 well block at 1 mL intervals. Heparin and SourceQ binding fractions were immediately snap-frozen in liquid nitrogen and stored at –80 °C until use.

*Fraction screening against SDE2*_*FL*_*:* Lysate, fractions from Heparin and SourceQ, and flowthrough (3 μL) were diluted with 10 μL of assay buffer (50 mM Tris pH 7.5, 1 mM DTT) and treated with either 2 μL of a solution (22.8 mg/ml) of recombinant SDE2_FL_ or 2 μL of buffer. Samples were incubated at 30 °C for 30 min before quenching with 4×SDS loading dye. Samples were boiled at 95 °C for 5 min and resolved by SDS-PAGE on 4–12% Tris-Acetate NuPage^®^ Novex^®^ (Life Technologies) polyacrylamide gels with 3-(*N*-morpholino)propanesulfonic acid (MOPS) running buffer (ThermoFisher Scientific) at 130 V. Gels were stained with Coomassie Instant Blue before imaging on ChemiDoc (Bio-Rad).

*Chromatography over Superdex 200:* The active fractions A12 and B1 were found to contain the relevant enzymatic activity. Fractions were concentrated to 0.4–0.5 mL and individually purified on a Superdex 200 Increase 10/300 GL column (Cytiva) equilibrated in 30 mM Tris pH 7.5, 10% glycerol, 150 mM NaCl, 7 mM 2-BME, 1 mM TCEP, 0.015% Brij35. Fractions (1 mL) were collected and subjected to screening as described above. Fractions showing processing of SDE2_FL_ (B2, B3 and B4) together with fractions A11, A12, B1 and B5, B6, B7 were processed for mass spectrometry analysis.

### High-throughput MALDI-TOF/MS SDE2 cleavage assay

A panel of recombinantly expressed DUBs were tested for their ability to cleave SDE2_UBL_ from SDE2_FL_. The screening was performed and analysed similarly to previously reported^73^. Briefly, recombinantly expressed DUBs were diluted in 40 mM Tris-HCl pH 7.5, 0.01% Bovine Serum Albumin (BSA) and 1 mM TCEP to a final concentration of 50–200 ng/µL^73^. The reaction was started by adding either positive control substrates (Ub dimers, trimers of different linkages or other pseudo-substrates as previously reported^59,73^) at the final concentration of 1.2–2.4 µM or SDE2_FL_ at final concentration of 3 µM. The reaction was stopped after 30 and 60 min incubation time using a mixture of 2% TFA and 0.8 µM ^15^N Ub (as internal standard). Positive control activity was reported as ratio of Ub/^15^N Ub (integrated areas under the peak of 8565.76 and 8669.47 *m/z*) while activity toward SDE2_UBL_ was assessed as ratio of SDE2_UBL_/^15^N Ub (SDE2_UBL_ 8348.9 observed *m/z*). Samples were mixed 1:1 with 2′,6′-Dihydroxyacetophenone matrix (DHAP), spotted on a 1536 AnchorChip MALDI-target using a Mosquito^®^ nanoliter pipetting system and analysed using a rapifleX MALDI Tissuetyper MS System as previously reported^73^.

### Alanine scanning

HEK293T cells were seeded in complete growth medium (2 mL of DMEM + 10% Foetal Bovine Serum, 2 mM L-glutamine, 2% Pen/strep) into 6 well plates at a density of 0.4 ×  10^6^ cells/well to achieve 70% density on day of transfection. 155 µL of 0.020 µg/µL plasmid solution was prepared in OptiMEM^®^ in an Eppendorf tube for each plasmid, and 9.8 µL of FuGENE^®^ HD reagent added. Tubes were mixed carefully and incubated for 15 minutes at room temperature. 150 µL of each transfection complex was added to individual wells of the seeded plates, before incubation for 48 h at 37 °C.

### Preparation of activity-based probes

HA-SDE2_UBL_-PA was generated through C-terminal functionalisation via an intein approach as previously described for other UBLs^35,74^. An activated C-terminal MESNA thioester, HA-SDE2_UBL_-MESNA, was utilised as a key intermediate.

An N-terminally tagged HA-tagged human SDE2(1-76) insert was cloned into a pTXB1 vector using NedI and SapI restriction enzymes to generate pTXB1_SDE2(1-76). Protein expression was carried out according to the general procedure described above. To generate HA-SDE2_UBL_-MESNA, cells pellets were resuspended in lysis buffer (50 mM HEPES pH 7.5, 150 mM NaCl, 0.5 mM TCEP) and sonicated on ice (5 min, 10 s on/ 15 s off, 60% amp). Lysate was clarified by centrifugation (72,000 × *g*, 30 min at 4 °C), followed by filtration (0.22 µm syringe filter). Clarified lysate was incubated with chitin beads (#S6651, NEB) for 4 h at 4 °C on a rotatory platform. After incubation, beads were collected in a reusable column and washed with lysis buffer (10 column volumes). Three column volumes of cleavage buffer (50 mM HEPES pH 7.0, 150 mM NaCl, 0.5 mM TCEP, 100 mM MESNA) was added to the beads, followed by incubation for ~62 h at 4 °C on a rotatory platform. The protein was then eluted from the resin and concentrated using a centrifugal filter (Amicon^®^ Ultra, 15 mL, 3 kDa MWCO). The HA-SDE2_UBL_-MESNA product was then either purified by RP-HPLC for long-term storage at –80 °C or directly conjugated with propargylamine.

To generate HA-SDE2_UBL_-PA, a solution of HA-SDE2_UBL_-MESNA was treated with propargylamine (final concentration of 225 mM). The solution was incubated at 25 °C for 2 h and reaction completion monitored by intact LCMS. The final product was isolated by RP-HPLC and lyophilised to give a white powder ( ~ 2.5 mg/L of culture). The ABP was renatured by dissolving the powder in minimal DMSO, followed by the slow addition of buffer (50 mM HEPES, 150 mM NaCl, 0.5 mM TCEP) to a final DMSO concentration of 5%. Using a centrifugal filter (Amicon^®^ Ultra, 0.5 mL, 3 kDa MWCO), the refolded probe was concentrated, diluted with buffer, and concentrated again to remove DMSO. Final concentration was determined using A280 absorbance (NanoDrop One, ε = 7365 M^–1^cm^–1^).

His-Ub-PA was obtained using the same method described for HA-SDE2_UBL_-PA.

His-SDE2_UBL_-PA probe was generated in an analogous manner to HA-SDE2_UBL_-PA with a modified purification method. Following MESNA-mediated cleavage from chitin beads, flowthrough containing His-SDE2_UBL_-MESNA was concentrated to 1–2 mL (Amicon^®^ Ultra, 0.5 mL, 3 kDa MWCO) and incubated with a solution of propargylamine (PA) (final concentration 225 mM) for 1 h at r.t. His-SDE2_UBL_-PA product was buffer exchanged into lysis buffer (50 mM HEPES pH 7.5, 150 mM NaCl, 1 mM DTT) to remove unreacted PA, then purified via gel filtration using a HiLoad 16/600 Superdex p75 column (Cytiva) into SEC buffer (50 mM HEPES 7.5 pH, 150 mM NaCl, 5% glycerol, 1 mM DTT). Fractions containing the probe were pooled, concentrated, flash-frozen, and stored at -80˚C.

ABP characterisation can be found in the Supplementary information file (Supplementary Figs. 21–23). Additionally, a representative concentration dependent labelling experiment for HA-SDE2_UBL_-PA is provided (Supplementary Fig. 24).

### RP-HPLC

Reverse-phase HPLC purification was performed on a ThermoFisher UltiMate 3000 HPLC system coupled to a Phenomex Jupiter 5 µm C4 300 Å LC column (250 × 10 mm). Proteins were concentrated to a maximum injection volume of 3.5 mL and subject to a gradient of 10–80% MeCN (0.1% TFA) in H_2_O (0.1% TFA) over 30 min with absorbance monitored at 214 and 280 nm. Data was analysed using Chromeleon 6.80. Intact LCMS was used to identify fractions containing the desired product, which were pooled and lyophilized.

### Intact LCMS

Intact LCMS was performed using a Zorbax 300SB-C3 5 µm (150 × 2.1 mm) column coupled to an Agilent system composed of a 1260 Bin Pump VL and Degasser, a 1290 Thermostatted Column Compartment and a 6130 Quadropole LC/MS. Sample injection volumes ranges between 1 and 5 µL and subject to a gradient of 10–75% MeCN (0.05% TFA) in H_2_O (0.05% TFA) over 20 min. Data analysis, including deconvolution of mass ions, was performed using Agilent LCMSD ChemStation (Rev. B.04.03-SP1)

### SDE2 cleavage and ABP labelling assays

Both SDE2 cleavage and ABP labelling assays were performed in 0.5 or 2 mL Eppendorf tubes by diluting purified DUBs (1 µM final concentration) and ABPs or SDE2_FL_ (2 µM final concentration) in reaction buffer (50 mM Tris pH 7.5, 1 mM DTT). The reactions were incubated at 37 °C for the indicated time then quenched with 4× LDS/DTT (final DTT concentration 100 mM) and analysed by SDS-PAGE.

*Quantified time course assay:* USP5 (0.1 µM final concentration) in the presence or absence of either Ub or SDE2_UBL_ (30 µM final concentration) was prepared in reaction buffer (50 mM Tris pH 7.5, 1 mM DTT). SDE2_FL_ (3 µM final concentration) was then added to each condition and incubated at 25 °C. Samples were taken and quenched into 4× LDS/DTT at indicated time points and analysed by SDS-PAGE and immunoblotting. The appearance of a band corresponding to SDE2_CT_ was quantified using Image Studio 6.1, values normalised relative to the highest and lowest band intensities, then plotted and statistically analysed using GraphPad Prism 10.3.1. A one-phase non-linear association was conducted to obtain the rate constant (K) for each treatment group. An extra sum-of-squares F-test was performed to assess compare kinetics of USP5 processing of SDE2_FL_ between treatment groups. Full blots are provided within the Supplementary information file (Supplementary Figs. 28-29).

### On-bead GFP-DUB assay

Expi293 cells from a 25 mL culture were suspended in 2 mL lysis buffer (50 mM Tris pH 7.5, 20 mM MgCl_2_, 0.5% NP-40) supplemented with cOmplete EDTA free protease inhibitor cocktail (Roche). The cell suspension was snap-frozen, defrosted and gently vortexed (3×), then the insoluble fraction removed by centrifugation at 4 °C (54,000 × *g*, 25 min). Protein concentration was determined by Bradford assay before use in on-bead assay.

Prior to the addition of lysate (300 µg protein, 200 µL total volume), anti-GFP Sepharose beads (120 µL slurry, DU61915, prepared by University of Dundee Reagents and Services^75^) were equilibrated in lysis buffer. Next, beads were incubated with lysate for 1 h at 4 °C, then supernatant removed by centrifugation (1500 × *g*, 1 min). Beads were washed with lysis buffer (3×) and resuspended in 600 µL assay buffer (50 mM Tris, 20 mM MgCl_2_, pH 7.5). To fresh tubes, 150 µL bead suspension was added, followed by either SDE2_FL_, K63-Ub_2_ or His-SDE2_UBL_-PA at the indicated concentration. Reactions were incubated at 37 °C for 1 h, centrifuged (1500 × *g*, 1 min) to separate the supernatant, and the beads washed with reaction buffer containing 0.5% NP-40 (3×). The supernatant and beads were treated with 4× LDS/DTT and analysed by SDS-PAGE.

### Activity-based DUB profiling

*Cell lysis and probe labelling:* HEK293 cells were cultured in 15 cm dishes until ~70–80% confluent. The cells were detached by rinsing the plate with ice-cold PBS, collected into a centrifuge tube and pelleted by centrifugation at 4 °C (3 min, 350 × *g*). The resulting pellet was washed twice with PBS before being flash-frozen in liquid nitrogen and stored at –80 °C until lysis. Frozen cells were resuspended in lysis buffer (50 mM Tris pH 7.5, 5 mM MgCl_2_, 0.5 mM EDTA, 250 mM sucrose, 1 mM DTT). One volume of acid-washed glass beads (Sigma Aldrich, G4649) per 3 volumes of ice-cold glass bead buffer was added to the tube containing the resuspended cells followed by 10 vortexing/cooling cycles (30 s bursts followed by incubation on ice for 2 min). The lysate was cleared by centrifugation (14,000 × *g*, 4 °C, 25 min). The protein concentration was determined by Bradford assay. Cell extract (500 µg) was diluted to a final volume of 300 µL using lysis buffer and incubated with 1 µM of probe for 45 min at 37 °C and 300 rpm. The reaction was quenched by adding 0.4% SDS and 0.5% NP-40. To immunoprecipitated DUB–ABP complexes, 50 µL of anti-HA magnetic beads slurry (Thermo Scientific), previously washed four times with NP-40 lysis buffer (50 mM Tris pH 7.5, 20 mM MgCl_2_, 0.5% NP-40), was added to 105 µL samples diluted with 210 µL of NP-40 lysis buffer and incubated on a rotator overnight at 4 °C. Beads were pelleted by placing the sample on a magnetic rack, and the supernatant was removed. Beads were washed four times with 300 µL of NP-40 lysis buffer. Protein complexes were eluted by boiling beads in 80 µL of 2× LDS sample buffer at 75 °C for 15 min and 10% were analysed by immunoblotting (Supplementary Fig. 20). 25 µL of the DUB-ABP immunoprecipitated samples were processed for proteomic analysis as described below.

### Mass spectrometry proteomics experiments

#### Lysate fractionation experiment

*Sample preparation:* For each fraction 50 µL was diluted in 2× S-trap lysis buffer and digested using the S-trap micro column protocol (ProtiFi) according to manufacturer’s recommendations.

*LCMS analysis:* Samples were reconstituted in HPLC grade water supplemented with 5% formic acid. Peptides were injected onto an UltiMate 3000 RSLCnano System operating in trap and elute mode coupled to an Orbitrap Fusion Lumos Tribrid Mass Spectrometer (Thermo Fisher Scientific). Peptides were loaded onto an Acclaim Pepmap trap column (Thermo Fisher Scientific #164750) and analysed on a PepMap RSLC C18 analytical column (Thermo Fisher Scientific #ES903) with a 120 min linear gradient from 3 to 35% Buffer B (Buffer A: 0.1% formic acid in water, Buffer B: 0.08% formic acid in 80:20 acetonitrile:water (v:v)). Eluted peptides were then analysed by the mass spectrometer operating in data independent acquisition mode. Samples were injected in instrumental triplicate.

*Data Analysis:* Peptide were searched against the Uniprot Swissprot Human database (released on 05/10/2021) using DiaNN (v1.8)^76^ operating in library free mode. Analysis was carried out using Python and packages pandas, numpy, sklearn, scipy, Plotnine and Plotly. Protein groups identified with a less than 2 proteotypic peptides were excluded. Protein group intensities were then compared to the quantified gel bands using Pearson coefficient. Protein groups with a Pearson coefficient greater or equal to 0.9 were considered as a potential SDE2 deubiquitinase. The mass spectrometry proteomics data have been deposited to the ProteomeXchange Consortium via the PRIDE^77^ partner repository with the dataset identifier PXD064153.

#### IP-MS

*Sample preparation*: Samples (25 µL) were reduced with 5 mM Bond-Breaker TCEP solution for 15 min at 55 °C. Samples were alkylated with 20 mM iodoacetamide for 10 min at room temperature, followed by acidification (final concentration 2.5% phosphoric acid). Sample were diluted by adding 165 mL of binding buffer (100 mM TEAB in 90% methanol). Samples were applied to S-trap micro column (ProtiFi) and spun down at 4000 × *g* for 30 s to trap proteins. Spin columns were washed 3 times with 150 mL of binding buffer and centrifuged in between each wash (4000 × *g* for 30 s) followed by a last centrifugation for 1 min to dry the spin column membrane from residual washing buffer. Digestion buffer (20 µL, 50 mM TEAB) containing trypsin (5 mg) was added to the top of the S-Trap column and incubated overnight at 37 °C. Digested peptides were eluted in three subsequent steps using buffer 1 (50 mM TEAB in water), buffer 2 (0.2% formic acid in water), and buffer 3 (50% acetonitrile in water). Eluted peptides were combined and dried down in a SpeedVac.

*LC-MS Analysis:* Samples were dissolved in HPLC-grade water containing 0.1% formic acid and 0.015% n-Dodecyl β-D-maltoside. Peptide separation was performed on a Vanquish Neo UHPLC system in trap-and-elute mode, interfaced with an Orbitrap Astral Mass Spectrometer (Thermo Fisher Scientific). Peptides were initially captured on a PepMap Neo Trap Cartridge (Thermo Fisher Scientific, #174500) and subsequently separated using a C18 EASY-Spray HPLC Column (Thermo Fisher Scientific, #ES906). The elution gradient spanned 11.8 minutes, increasing from 1 to 55% buffer B (Buffer A: 0.1% formic acid in water; Buffer B: 0.08% formic acid in 80:20 acetonitrile:water (v:v)) with the following profile: 0.7 min at 1.8 µL/min from 1% to 4% B, 0.3 min at 1.8 µL/min from 4 to 8% B, 6.7 min at 1.8 µL/min from 8 to 22.5% B, 3.7 min at 1.8 µL/min from 22.5 to 35% B, and a final step of 0.4 min at 2.5 µL/min from 35% to 55% B. Peptides were analysed via data-independent acquisition (DIA) on the mass spectrometer.

*Data Processing and Statistical Analysis:* Peptide identification was carried out against the UniProt Swiss-Prot human database (release date: 02/01/2023) using DiaNN (v1.9)^76^ in library-free mode, with cross-run normalization disabled. Thread number was set to 64, output was filtered at 0.01 FDR, N-terminal methionine excision was enabled. Minimum and maximum peptide length were set to 7 and 30 respectively. Minimum and maximum precursor m/z set to 300 and 1800 respectively. Minimum and maximum precursor charge were set to 1 and 4 respectively. Protease cleavage sites were at Lys and Arg. Maximum number of missed cleavages was set to 2 and cysteine carbamidomethylation was enabled as a fixed modification. Data analysis was performed using Python (packages: pandas, numpy, sklearn, scipy, rpy2, Plotnine, and Plotly) and R (Limma package^78^). Protein groups were retained only if at least two peptides were identified and quantified in a minimum of two replicates. Missing values were imputed using a Gaussian distribution centred on the median, applying a downshift of 1.8 and a width of 0.3 (relative to the standard deviation). Differential protein regulation was assessed using LIMMA eBayes, with multiple hypothesis correction via the Benjamini-Hochberg method. Proteins were considered significantly regulated if the adjusted *p*-value was below 0.05 and the fold change exceeded 1.5 or was lower than 1/1.5. The mass spectrometry proteomics data have been deposited to the ProteomeXchange Consortium via the PRIDE^77^ partner repository with the dataset identifier PXD064155.

### HDX-MS experiments

*Sample preparation:* USP5^WT^ (6 µM) was incubated with or without ubiquitin/SDE2_UBL_ (200 µM) for 30 min in Protein Dilution Buffer (20 mM HEPES pH 7.5, 150 mM NaCl, 1 mM DTT). 5 µL of this sample was diluted with 45 µL of Deuteration Buffer (20 mM HEPES pH 7.5, 150 mM NaCl, 1 mM DTT, 92% D_2_O) (final D_2_O concentration = 82.8%) for timepoints 3/30/300/3000 s, before being quenched with 20 µL of ice-cold Quench Solution (4 M Urea, 2% Formic Acid), and being snap frozen in liquid nitrogen and stored at –70 °C. A further time point, 0.3 s, was achieved by incubating both the sample and deuteration buffer on ice and then conducting a 3 s exchange reaction. Each exchange reaction was conducted independently four times.

*Data acquisition:* Samples were rapidly thawed at room temperature and then injected into automated HDX-MS fluidics and UPLC manager system (Waters). Samples were loaded into a 50 µL loop and subsequently digested using a Waters Enzymate BEH Pepsin Column (Part No. 186007233) in a 0.1% Formic Acid solution with a flow rate of 200 µL/min at 20 °C. Peptic peptides then flowed onto a Waters ACQUITY UPLC BEH C18 VanGuard Pre-Column at 1 °C (Part No. 186003975). After digestion, the flow path was changed to elute the peptides via a Waters ACQUITY UPLC BEH C18 1.7 µm 1.0 × 100 mm reverse phase column (Part No. 186002346). A gradient from 0-85% 0.1% Formic Acid/Acetonitrile, conducted at 1 °C with a 40 µL/min flowrate, was used to elute deuterated peptides which were then ionised using an ESI source. Mass spectrometry data were collected using a Waters Select Series cIMS instrument, from a 50–2000 *m/z* range with the instrument in HDMSe mode. A single pass of the cyclic ion mobility separator (with a cycle time of 47 ms) was conducted. A blank sample of protein dilution buffer with quench was run between samples, and carry-over was routinely checked to be <1% intensity of the prior sample.

*Data analysis:* Peptide sequence identification was conducted using Protein Lynx Global Server (Waters). Minimum inclusion criteria were a minimum intensity of 5000 counts, minimum sequence length 5, maximum sequence length 35, a minimum of three fragment ions, a minimum of 0.1 products per amino acid, a minimum score of 5.0, a maximum MH+Error of 10 ppm. Subsequent analysis and determination of deuteration values conducted using HDExaminer (Trajan Scientific). Experimental design, data acquisition, analysis and reporting are in line with the community agreed recommendations^79^. HDX-MS uptake plots are available as a Supplementary file.

### Endogenous siRNA

HEK293, U2OS, HeLa and MCF-7 cells were seeded at a density of 0.4 × 10^6^ cells/well into 6-well plates to achieve 70% density on the day of transfection. To perform knockdown experiments, USP5 SMARTPool (cat. L-006095-00-0005, Dharmacon), SDE2 SMARTPool (cat. L-016456-02-0005, Dharmacon) and control siRNA (cat. D-001820-10-05, Dharmacon) were used at a final concentration of 40 nM per well. 9 µL of prepared siRNA stock [10 µM] was diluted in 141 µL of Opti-MEM^®^ (31985062, Thermo Fisher) and 9 µL of RNAiMAX^®^ (13778100, Thermo Fisher) in 141 µL Opti-MEM^®^. The two solutions were carefully mixed, incubated for 5 min at RT, and 300 µL of RNAiMAX:siRNA complex was added dropwise to the cells and the plate was swirled to ensure even distribution. Cells were incubated for 72 h before harvesting. Any rescue overexpression transfection of USP5 or USP13 to the cells was done 24 h after siRNA treatment using the method described above and incubated 48 h before harvest.

### NanoBRET assay

Wild-type full length SDE2 and uncleavable mutant (G76A G77A) were cloned into pF-NH Flexi Vector (Promega) with an N-terminal Nanoluc^®^ and C-terminal Halotag^®^. HEK293T cells were seeded into 6 well plates in complete growth medium (2 mL of DMEM + 10% Foetal Bovine Serum, 2 mM L-glutamine, 2% Pen/strep) at a density of 0.5  × 10^6^ cells/well to achieve 70% density on day of transfection. 24 h after seeding, cells were treated with siRNA as described in beforementioned method (*Endogenous siRNA*). A day later (24 h) NanoBRET^®^ constructs (N-SDE2-H^WT^ or N-SDE2-H^UC^) were transfected into cells: 1.5 µg of DNA was mixed with 9.8 µL of FuGENE^®^ HD transfection reagent (E2311, Promega) in a total of 155 µL of Opti-MEM^®^. In the case of rescue samples, 1.5 µg of rescue DNA construct was combined with 1.5 µg of NanoBRET^®^ DNA construct and diluted in 155 µL Opti-MEM^®^ and 9.8 µL of FuGENE^®^. DNA:FuGENE^®^ mixture was incubated 20 min at r.t and then 150 µL was added to each well dropwise and gently mixed for even distribution. Next day (24 h later) cells were replated onto Nunc™ MicroWell™ 96-well flat-bottom microplate (136101, Thermo Fisher) according to Promega NanoBRET protocol. Briefly, cells were washed with PBS (×2), detached using trypsin-EDTA (25300054, Thermo Fisher), neutralised with 2 mL DMEM and centrifuged (125× *g*, 5 min). Neutralisation media was aspirated and cells were resuspended in Opti-MEM™ I Reduced Serum Medium, no phenol red (11058021, Gibco) containing 4% FBS to achieve a concentration of 2 × 10^5^ cells/ml. Cells were divided into two pools, to be treated with either HaloTag^®^ NanoBRET™ 618 Ligand or DMSO vehicle control. For experimental samples, 500 µL of cells suspension from the above pools were treated with either 0.5 µL of 618 Ligand (100 nM final concentration) or vehicle control (0.1% DMSO final concentration). Treated cells were then seeded as triplicate (100 µL per well) and incubated overnight (18 h) at 37 ˚C 5% CO_2_. Excess cells remaining in conical tubes were washed and lysed for immunoblotting to verify protein expression. A 5× solution of NanoBRET™ Nano-Glo^®^ Substrate was prepared in Opti-MEM^®^ Reduced Serum Medium, no phenol red at 1:100 ratio (v:v) of NanoBRET™ Nano-Glo® substrate to Opti-MEM® Reduced Serum Medium, no phenol red. 25 µL of substrate was added to each well containing cells using a multidispenser. The plate was placed into a Promega GloMax^®^ plate reader and BRET signal measured using a BRET: NanoBRET 618 protocol with prior shaking for 30 s. Raw NanoBRET™ units were converted into milliBRET units (mBU) by multiplying by 1000. The mean NanoBRET™ ratio was then determined and corrected mBU was calculated as follows: Mean mBU experimental—Mean mBU no-ligand control = Mean corrected mBU. All graphs created using GraphPad Prism. Standard error of the mean calculated and displayed as error bars, where appropriate.

### Intron retention PCR

HEK293 and HeLa cells were grown on a 6-well plate and treated with siRNA for 72 h as described in “*Endogenous siRNA*” method. Cells were washed with PBS (2×) and then lysed. For RNA, Qiagen RNA extraction kit (74104, RNeasy kit) was used for lysis and obtaining of RNA samples. RNA was reverse-transcribed into cDNA using iScript™ cDNA Synthesis Kit (1708891, Bio-Rad). For DNA, GeneJET Genomic DNA Purification Kit (K0721, Thermo Scientific) was used according to manufacturer’s protocol. PCR primers were ordered from Merck (Supplementary Table 5) and PCR was performed using PrimeSTAR^®^ GXL DNA Polymerase (R050A, Takara Bio). PCR reaction mixes were prepared according to Takara Bio recommendations. 3-step PCR conditions used were: 10 s 98˚C, 15 s 60˚ C, 30 s 68 ˚C, 35 cycles. 5 µL of PCR product was mixed with 1 µL of 6×Gel Loading Dye purple (B7024, NEB). Samples were run on 2% agarose gel containing SYBR™ Safe DNA gel stain (S33102, Invitrogen) and run at 100 V for 25 min. Gel was visualised using a ChemiDoc™ MP Imaging System (Bio-Rad).

### Thermal shift assay

Components used in thermal shift assays were prepared in reaction buffer (PBS, 1 mM DTT) and the assays performed in a white BioRad PCR plate, sealed with optically clear plate sealer (BioRad). USP5 (6 µL, 4 µM) was mixed with ABP (SDE2_UBL_-PA, ISG15-PA, Ub-PA, 6 µL, 8 µM), or buffer and incubated at 37 °C for 1 h. To the protein solution was added SYPRO orange (8×, 12 µL) and plate thermocycled using a Bio-Rad CFX Opus 384 RT-PCR (10-95 °C, 0.5 °C increase every 30 s) and Bio-Rad CFX Maestro 2.3, with the fluorescence detected using the FRET channel. RFU was plotted against temperature and the first derivative (dRFU/dT) was calculated using GraphPad Prism 9. Melting temperature (T_m_) is calculated as the temperature which gives the maximum dRFU/dT value. Experiments were performed in triplicate and replicates used to calculate a mean T_m_ ± SEM. Full data is presented in Supplementary Table 6.

### ITC

Experiments were performed on an ITC200 micro-calorimeter (GE Healthcare) at 298 K. To prepare samples, protein stock solutions were dialysed overnight in ITC buffer (2 L, 50 mM HEPES, 100 mM NaCl, 1 mM TCEP, pH 7.5) using D-Tube Dialyzers (Merck, MWCO 3.5 kDa or 11–12 kDa). The dialysis buffer was filtered and degassed then used to dilute proteins to the indicated concentration. Titrations were performed with a single injection of 0.4 µL (data point discarded) followed by 19 injections of 2 µL at 120 or 180 s intervals (see individual experiments). Data was processed using the MicroCal ORIGIN software package. Experiments were performed in duplicate (Supplementary Figs. 26–27).

### TR-FRET

TR-FRET assay buffer (20 mM HEPES, 150 mM NaCl, 1 mM TCEP, 0.5% DMSO and 0.05% Tween-20, pH 7.5) was used to dilute all samples to 4 × final concentration. To each well of a 384-well white OptiPlate (Perkin Elmer) was added His-tagged donor protein (4 µL), LANCE Eu-W1024 Anti-His_6_ antibody (4 µL, final concentration 2 nM, Revvity AD0401), Cy-5 labelled acceptor protein (4 µL), and additional TR-FRET assay buffer (4 µL). Following 1 h incubation at room temperature the FRET ratio was measured at λ_exc_ 337 nm, λ_em_ = 620 nm and 665 nm using a PHERAstar FXS plate reader (BMG Labtech). All experiments were performed in triplicate. The emission values were converted to a ratio of the emission at 665 nm to 620 nm (FRET ratio = [665 nm/620 nm]*10000) and a mean value for each condition was calculated. A Cy5 labelled acceptor only control was used to determine background signal and subtracted from final readings. The one site-specific binding equation was used on Prism 10.3.1 to calculate the B_max_ and *K*_d_ for each condition.

### Fluorescence polarisation (FP)

FP assays were performed in 384-well black plates (Greiner) using FP assay buffer (50 mM HEPES, 150 mM NaCl, 1 mM DTT). Stock USP5^WT^, Ub and SDE2_UBL_ were diluted in assay buffer to 1.5 µM (3× final concentration) and Ub-KG-TAMRA (UbiQ, UbiQ-012) was diluted to 1 µM 10× final concentration. To assay buffer (4 µL) was added 3× USP5^WT^ (5 µL) and either 5 µL of 3× Ub, 3× SDE2_UBL_, or buffer. A blank (buffer only) and a negative control (14 µL buffer + 1 µL 10× Ub-KG-TAMRA) were also prepared. The reaction was initiated by the addition of 10× Ub-KG-TAMRA (1 µL) to each of the test wells. Using a ClarioStarPlus plate reader, fluorescence polarisation was measured at ambient temperate every 20 s for 60 min (Ex: 540, Em: 590, target mP= 150, blank corrected). Experiments were performed in triplicate. Data was analysed and visualised on Prism 10.3.1 and final polarisation values were compared using a one-way ANOVA test.

### AlphaFold modelling

For SDE2_UBL_ modelling depicted in Fig. 1, *Homo sapiens* (Q6IQ49, residues 1-77) and *Schizosaccharomyces pombe* (O14113; residues 1-84) N-terminal UBL sequences were modelled in a human USP5 bound state using AlphaFold 3^30^ (*H. sapiens* SDE2: ipTM=0.9 pTM=0.67; *S. pombe* Sde2: ipTM=0.26 pTM=0.61). SDE2_UBL_ structure was extracted and superimposed onto Ub (*H. sapiens* RMSD = 2.029; *S. pombe* RMSD = 1.574) in a solved USP5-Ub crystal structure (PDB: 3IHP). Predicted alignment error plots are shown in Supplementary Fig. 29. For comparison of C-terminal interactions and general binding modes (Supplementary Fig. 4 and Supplementary Fig. 10) one molecule of SDE2 1-77 (ipTM = 0.9, pTM = 0.67) or Ub 1-76 (ipTM = 0.95, pTM = 0.76) were modelled bound to USP5 using AlphaFold 3. For HDX-MS modelling (Supplementary Fig. 18) two molecules of SDE2 1-77 (ipTM = 0.59, pTM = 0.65) or Ub 1-76 (ipTM = 0.61, pTM = 0.68) were modelled bound to USP5 using AlphaFold 3.either one or two molecules of SDE2 1-77 1×: ipTM = 0.61, pTM = 0.68, 2× or Ub (1-76) were modelled bound to USP5 using AlphaFold 3. Structures were visualised using either PyMOL Molecular Graphics System, Version 3.0.4, Schrödinger, LLC or ChimeraX 1.9^80^.

Sequences used:

USP5 (*H.sapiens*): UniProt P45974-1 full length (1-858) with no modifications. SDE2_UBL_ (*H.sapiens*): UniProt Q6IQ49-1 UBL sequence (1-77) with no modifications. SDE2_UBL_ (*S.pombe*): UniProt O14113 UBL sequence (1-84) with no modifications. Ub (*H.sapiens*):UniProt P0CG48 sequence (1-76) with no modifications.

### Statistics and reproducibility

SDS-PAGE gel and western blot images in Figs. 1B, 2B−E, 3 A,  C, D,  F, 4E, 5 F, 6B,  C are representative of data from three separate experiments. Exact *p* values for hits in the ABP IP-MS in Fig. 3E are: -log_10_ *p* value (SDE2) = 2.287271; -log_10_ *p* value (USP5) = 2.682357; -log_10_ *p* value (USP13/5) = 2.682357. In Figs. 4B−D, 5 G exact *p* value when *p* < 0.0001 (****) is not available. Adjusted *p* values available for the non-significant values are as follows: 4B, columns C-E = 0.9359; 4 C, columns C-D = 0.4031; 4 C, columns C-E = 0.1172. Other parameters of the Šídák’s multiple comparisons test are all available in the Source Data file. No data has been excluded from the analysis.

### Reporting summary

Further information on research design is available in the Nature Portfolio Reporting Summary linked to this article.

## Supplementary information

Supplementary Information

Reporting Summary

Transparent Peer Review file

## Source data

Source Data

## Data Availability

The mass spectrometry proteomics data generated in this study have been deposited to the ProteomeXchange Consortium via the PRIDE partner repository database under accession codes PXD064153 and PXD064155 [http://proteomecentral.proteomexchange.org/cgi/GetDataset?ID = PXD64155]. The USP5 HDX-MS data generated in this study have been deposited to the ProteomeXchange Consortium via the PRIDE partner repository database under accession codes PXD072107. All other generated in this study are provided in the Supplementary Information and Source Data files. Source data are provided with this paper.
