# Supplementary material for: USP5 acts as a proteolytic switch to control SDE2 function via UBL-directed cleavage
Source: Nat Commun. 2026 Aug 14;17:9775. doi: 10.1038/s41467-026-76751-6 (PMC13572447; doi:10.1038/s41467-026-76751-6)
Supplement: Supplementary file 1 [file 41467_2026_76751_MOESM1_ESM.pdf]

Supplementary Figures

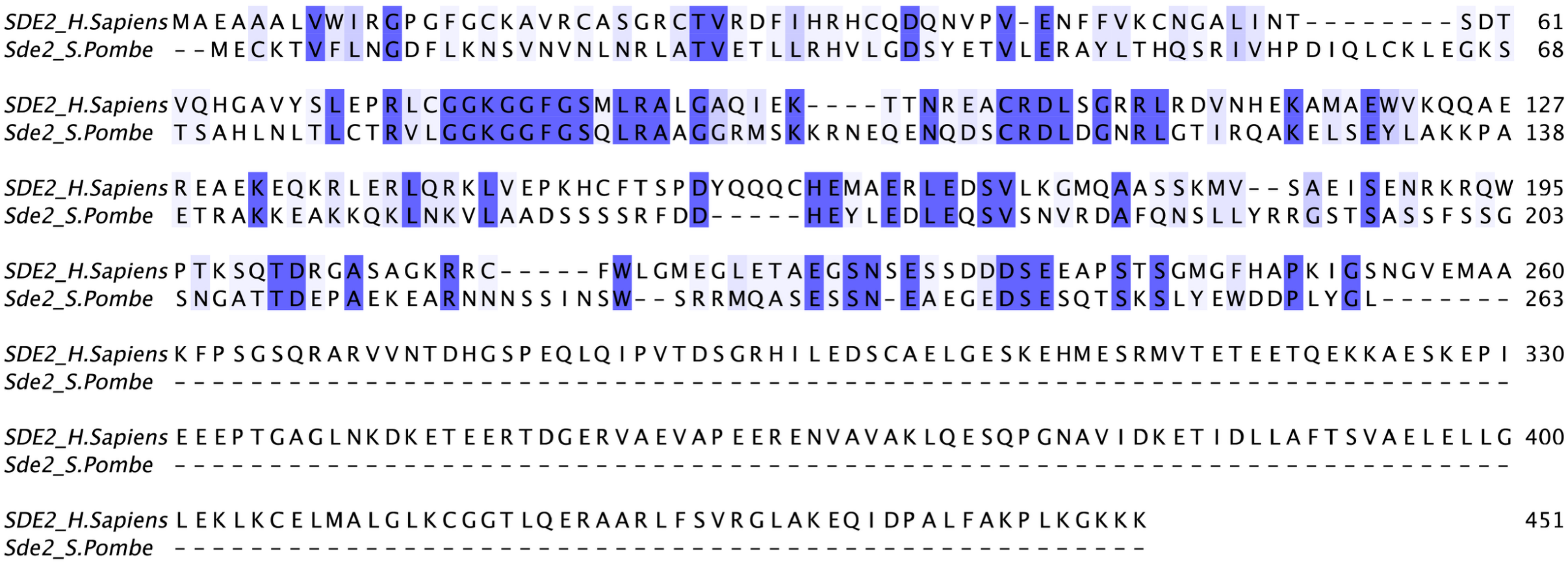

Supplementary Figure 1 | Sequence alignment of *H. sapiens* SDE2 with *S. pombe* Sde2. Sequences were aligned with Clustal Omega[1] and conserved residues were annotated in Jalview[2] by conservatism. Dark blue indicates fully conserved, while light blue shows semi-conserved residues.

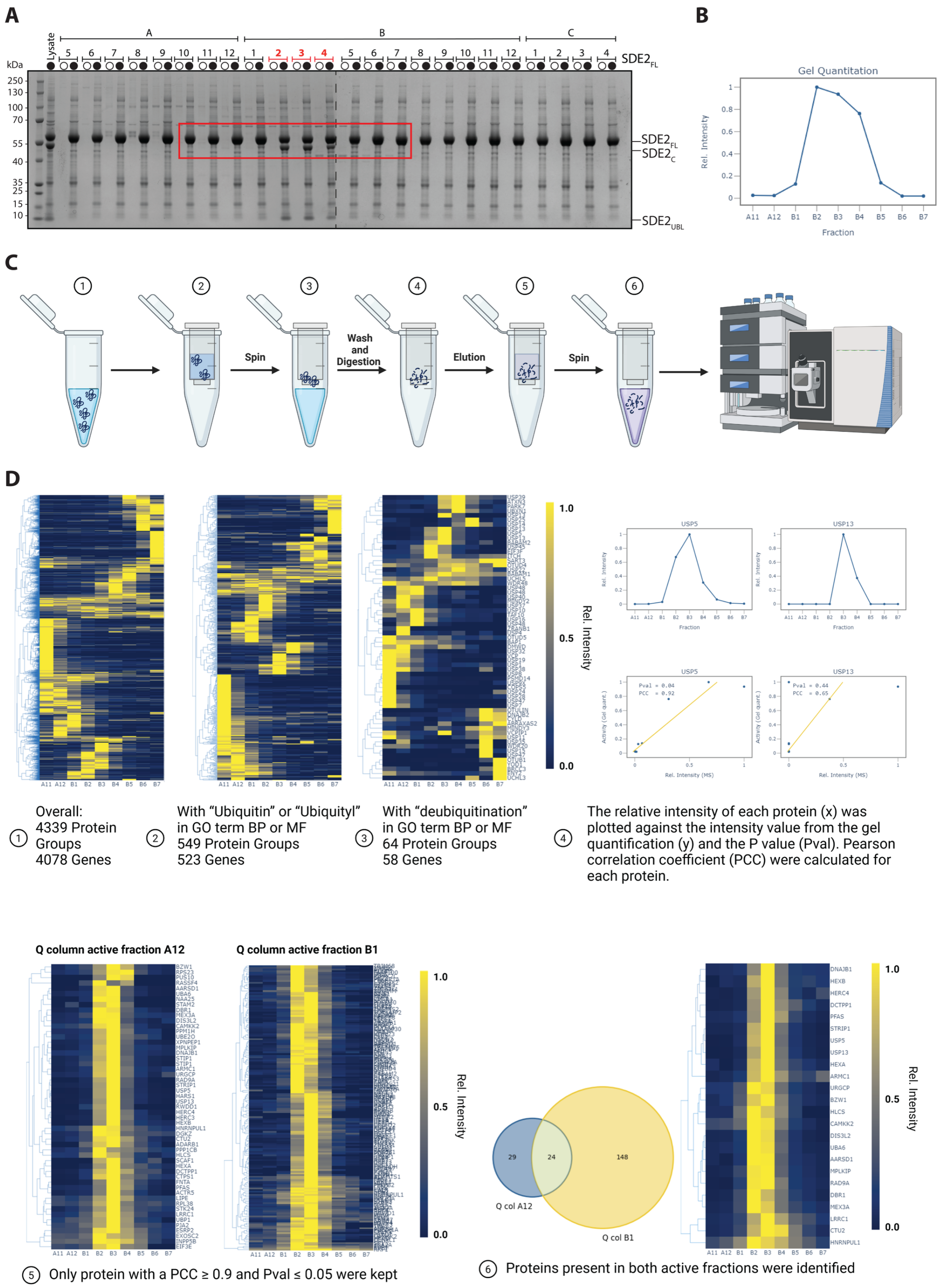

Supplementary Figure 2 | Cellular fractionation experimental data and processing. (A) Source-Q fractions with activity against SDE2 (A12 and B1) were individually further fractionated by SEC. Collected fractions were incubated with $SDE2_{FL}$ (30 min, 30 °C) and analysed by SDS-PAGE and Coomassie staining. Cleavage activity was detected in fractions B2-B4 (in red). **(B)** Cleavage was quantified by measuring the intensity of the $SDE2_{CT}$ band for each active fraction, together with adjacent fractions (A11-B1 and B5-B7). **(C)** Sample preparation workflow for mass spectrometry analysis using S-trap (Profiti). **(D)** Workflow used for proteomic analysis and relative candidate identification. Created in BioRender. Maniaci, C. (2026) https://BioRender.com/kuc96z2

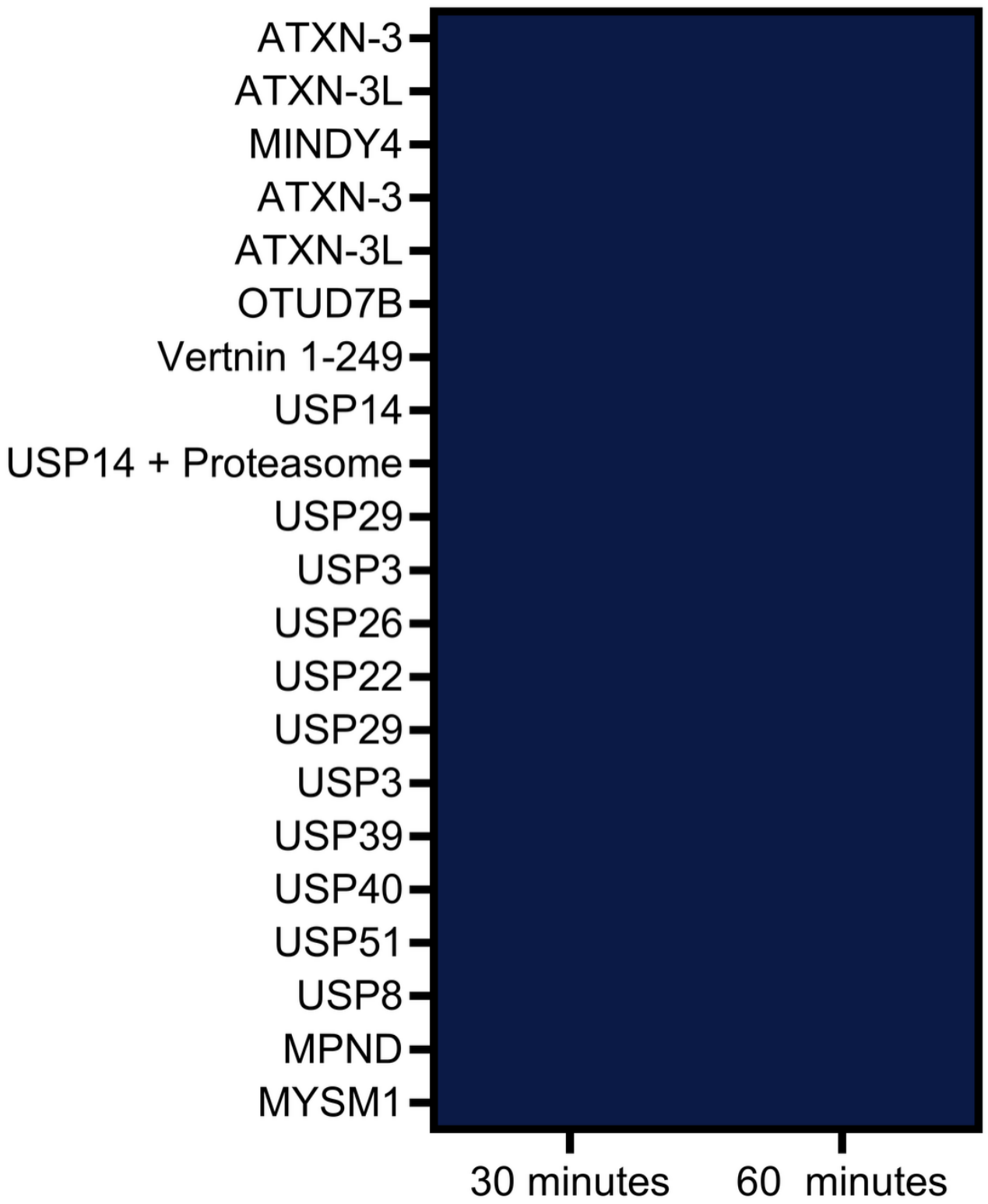

Supplementary Figure 3 | Extended panel of enzymes which lack known substrates tested for activity against recombinant SDE2$_{FL}$. Enzymes were incubated with SDE2$_{FL}$ for 30 or 60 min at 30 °C and analysed by the MALDI-TOF MS. No reaction was observed with SDE2$_{FL}$.

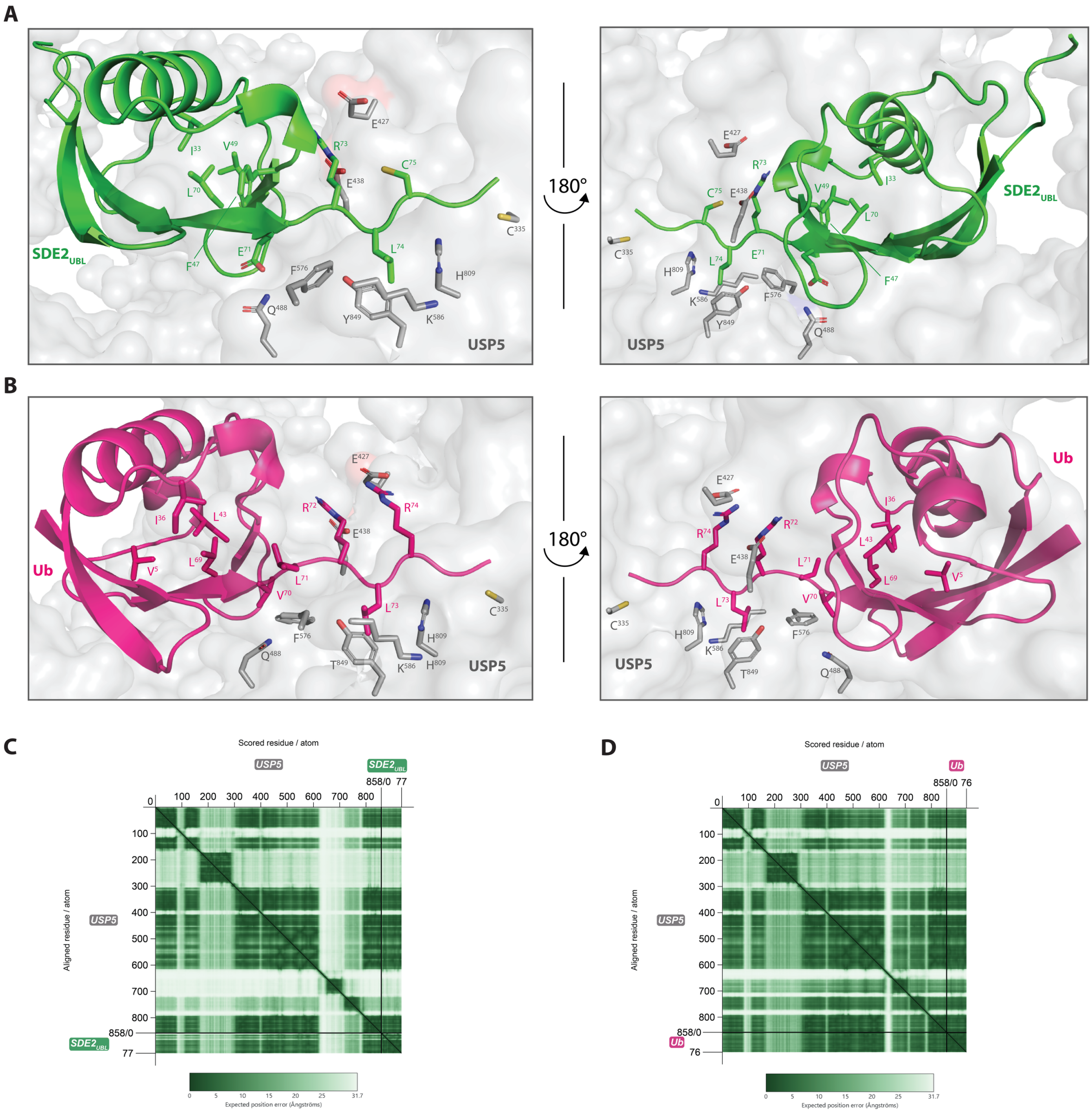

Supplementary Figure 4 | Comparison of predicted interactions between USP5 and the C-terminal tail of SDE2$_{UBL}$ and Ub. AlphaFold 3[3] was used to predict the binding of USP5 (grey) to SDE2$_{UBL}$ (**A**, green) or Ub (**B**, magenta). Key residues within the C-terminal tail, the residues with which they interact, and the catalytic cysteine (C335) are annotated. PAE plots corresponding to the AlphaFold 3 predictions of USP5 bound to SDE2$_{UBL}$ (**C**) and Ub (**D**).

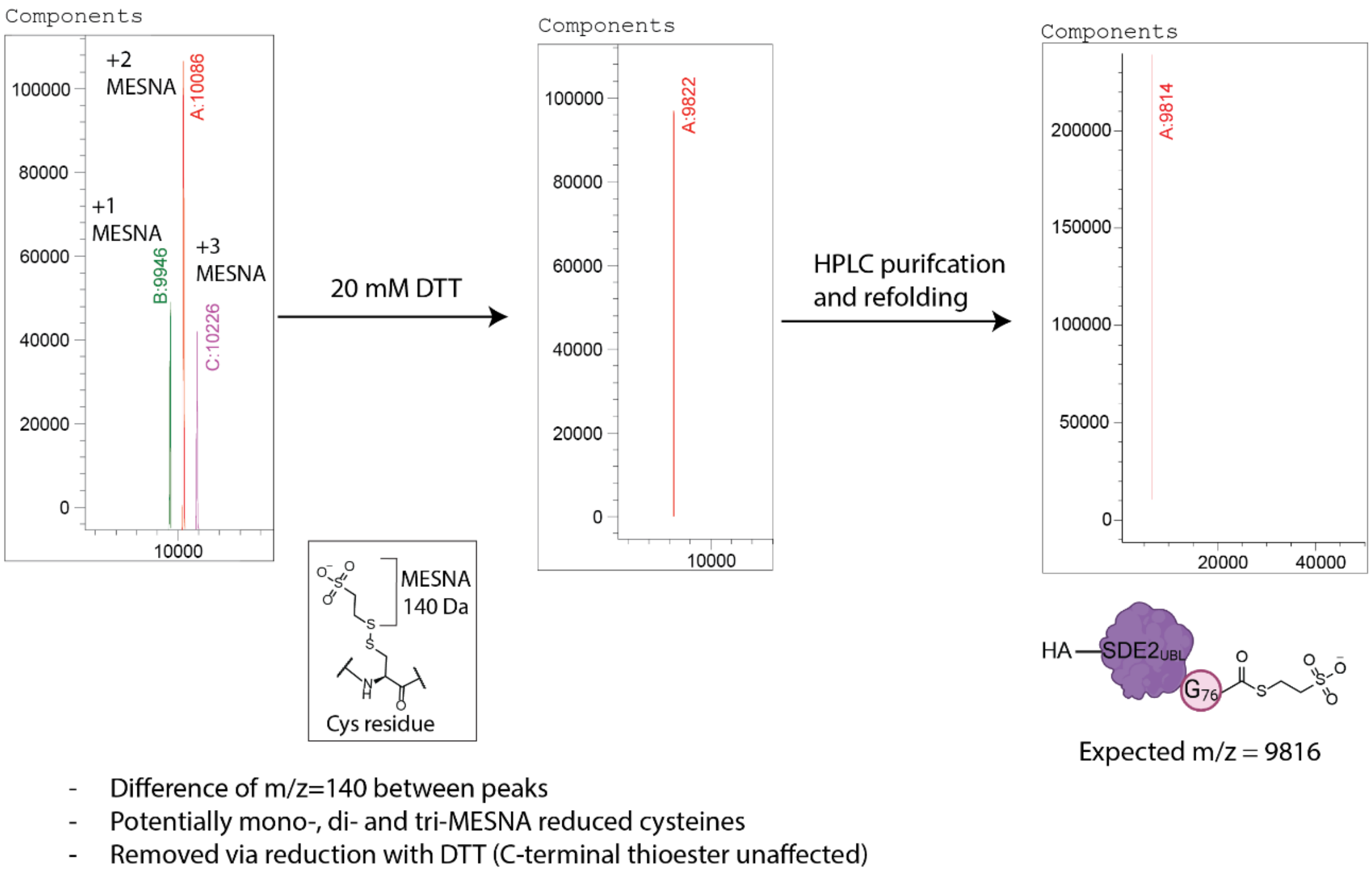

Supplementary Figure 5 | Intact LCMS of HA-SDE2$_{UBL}$-MESNA following the MESNA-mediated cleavage of HA-SDE2$_{UBL}$ΔG77 from the chitin resin resulted in mass ions higher than expected. The additional masses increased by 140, which corresponds to the mass of one (140 Da) or multiple MESNA molecules. Addition of DTT lead to the disappearance of these mass ions, suggesting that they are due to the formation of disulfide bonds between Cys residues and MESNA which are reduced in the presence of DTT. HPLC purification and protein refolding then intact LCMS analysis gave a *m/z* signal corresponding to the oxidised HA-SDE2$_{UBL}$-MESNA molecular weight. Created in BioRender. Maniaci, C. (2026) https://BioRender.com/sel9wzz

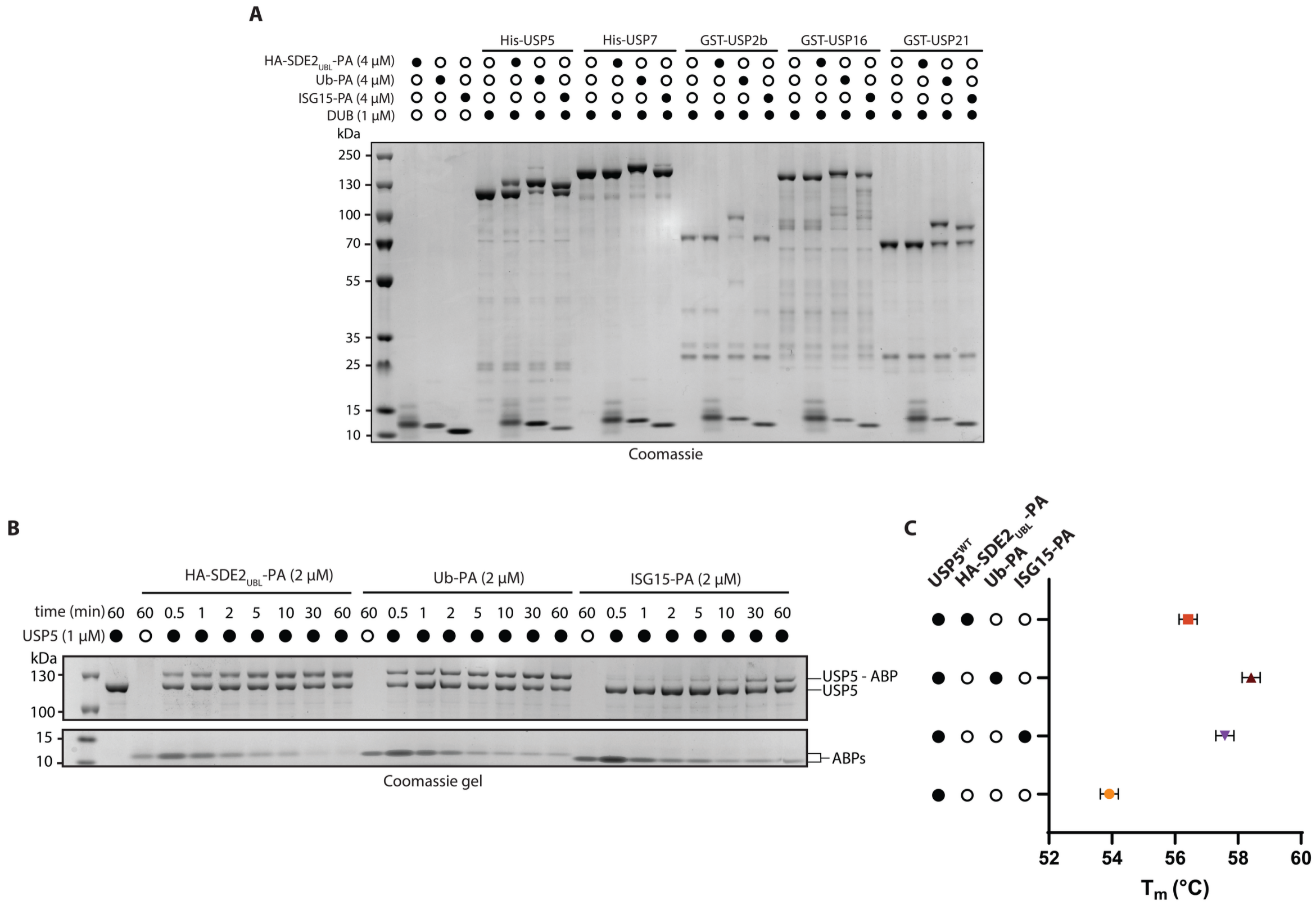

Supplementary Figure 6 | Comparison of SDE2$_{UBL}$, Ub and ISG15derived ABPs. (A) The reactivities of HA-SDE2$_{UBL}$-PA, Ub-PA or ISG15-PA were compared across a panel of DUBs. Each DUB (1 µM) was incubated separately with each ABP (2 µM each) for 1 h at 37 °C. A band shift was observed for ABP-reactive DUBs by SDS-PAGE and Coomassie staining. **(B)** Time course assay to assess the kinetic of ABP reactivity. USP5^{WT} (1 µM) was incubated separately with each ABP for 1 h at 37 °C with a sample taken at the indicated time points. **(C)** Thermal shift analysis of USP5-ABP complexes. USP5^{WT} (1 µM) was incubated with each ABP for 1 h at 37 °C before thermal cycling from 10-95 °C in the presence of SYPRO orange was performed. Calculated melting temperatures (T_m) indicate the stability of the USP5-ABP covalent complex. Source data are provided as a Source Data file.

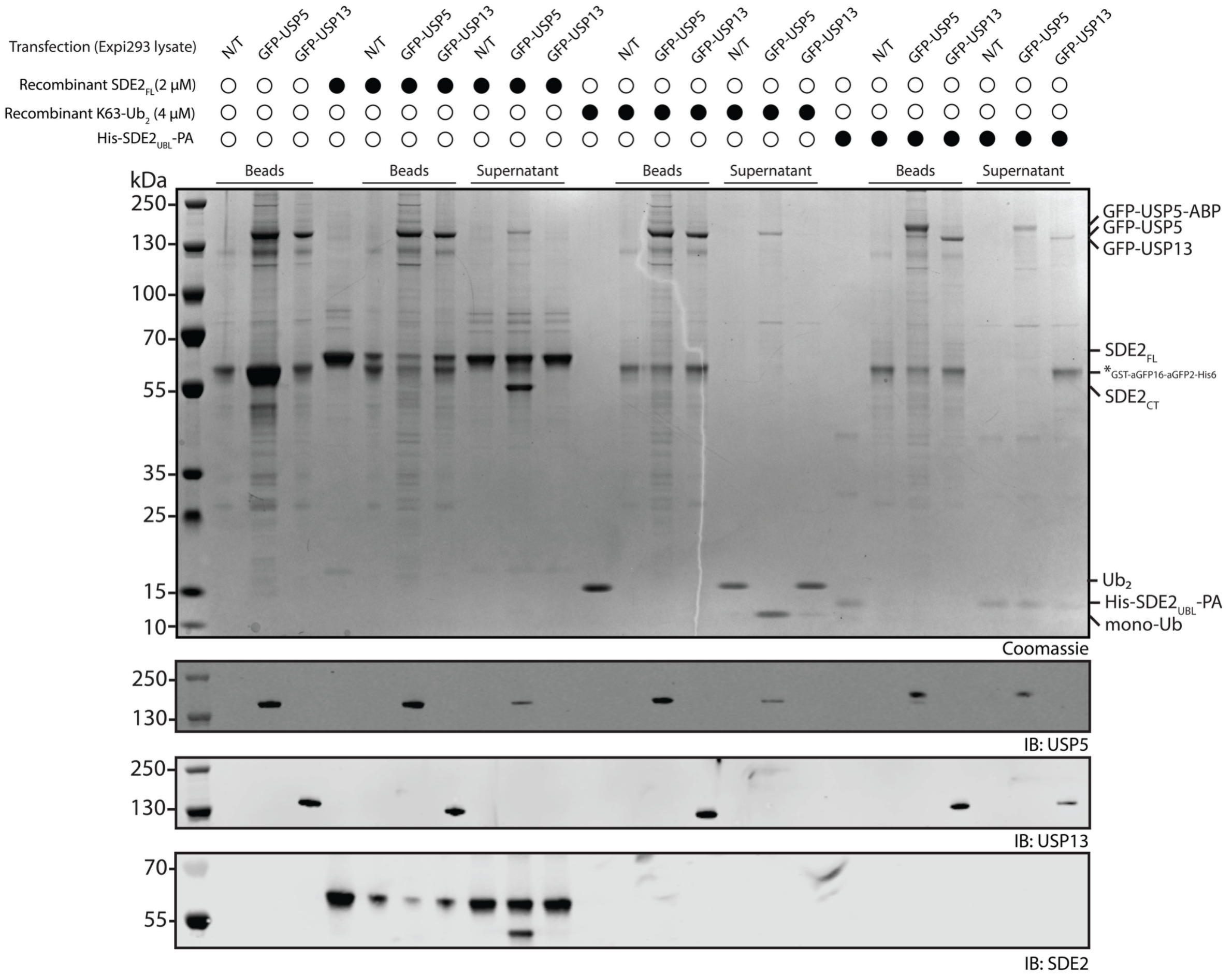

Supplementary Figure 7 | Extended on-bead assessment of GFP-USP5 and GFP-USP13 activity. α-GFP beads conjugated to Expi293 recombinantly expressed GFP-USP5 or GFP-USP13 were incubated with recombinant $SDE2_{FL}$ (2 µM), K63-Ub_2 (4 µM) or His-$SDE2_{UBL}$-PA (5 µM) for 1 h at 37 °C. The beads and reaction supernatant were analysed separately via SDS-PAGE followed by staining with Coomassie blue or immunoblotting.

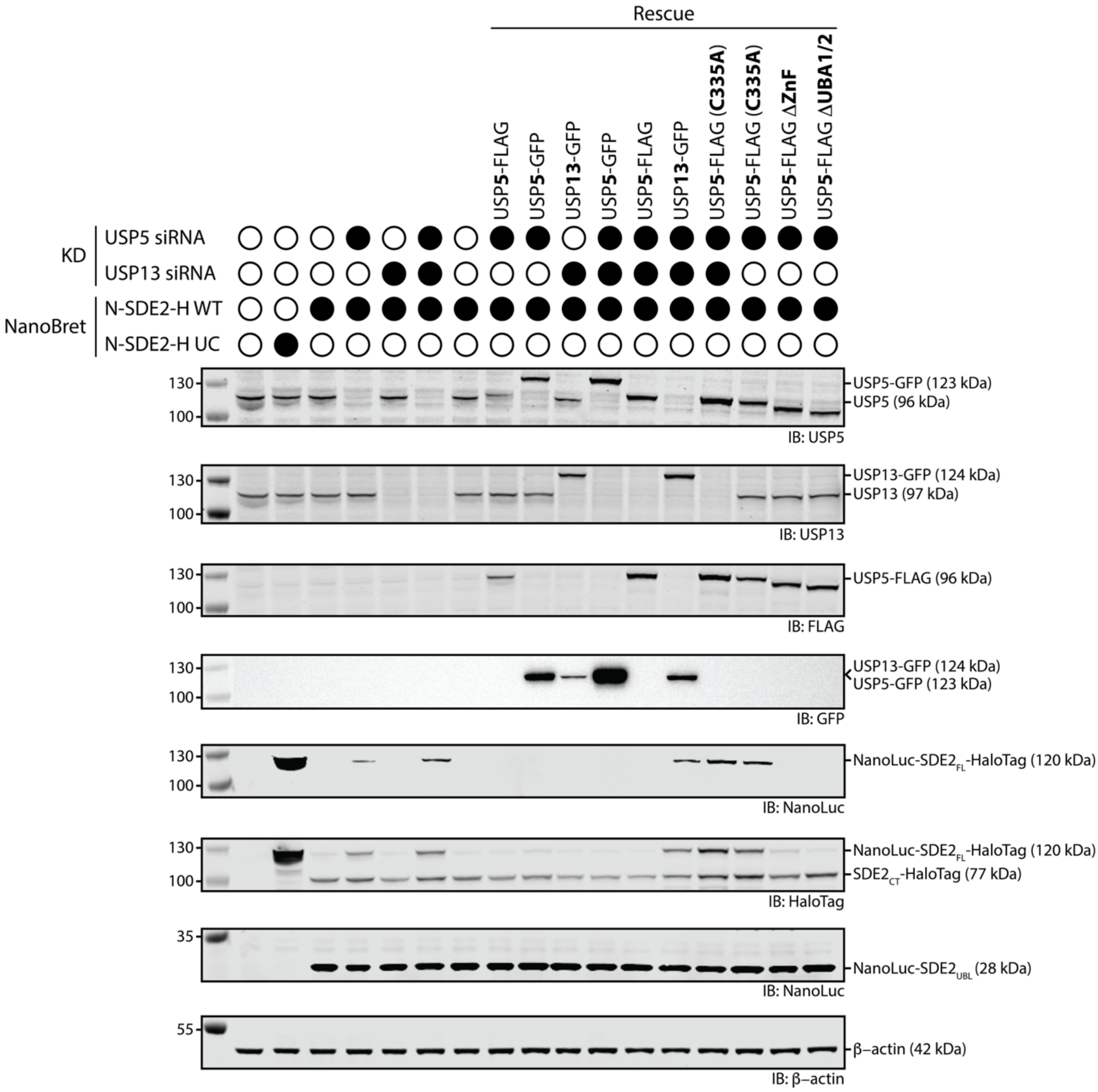

Supplementary Figure 8 | Western blot analysis of HEK293 cells used for NanoBRET®. Cells overexpressing NanoLuc-SDE2$_{FL}$-HaloTag (N-SDE2-H^{WT}) or G76A G77A uncleavable mutant (N-SDE2-H^{UN}) were treated with USP5 or USP13 specific siRNA, singularly or in combination. Control siRNA was used to assess potential non-specific effects.

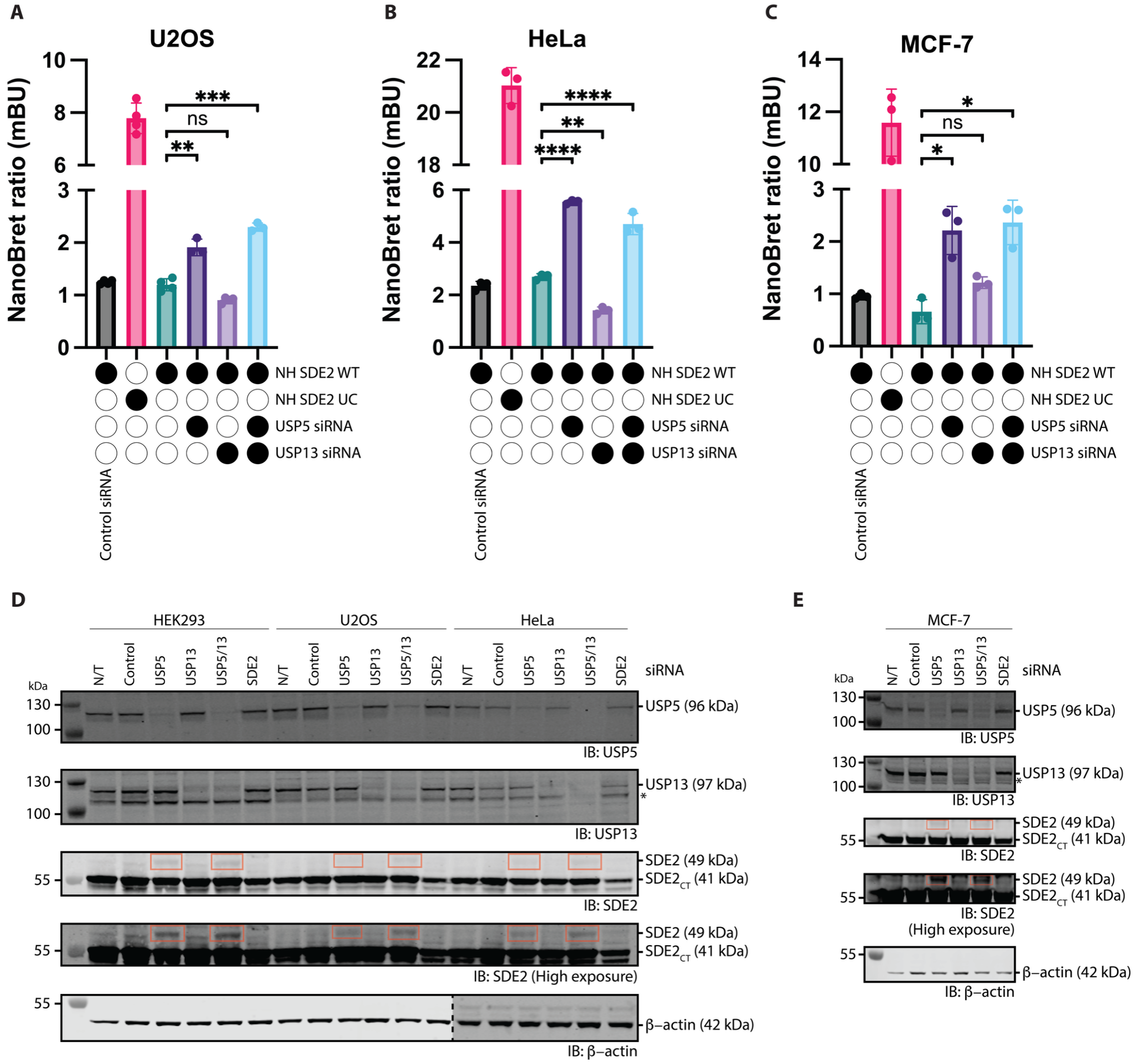

Supplementary Figure 9 | USP5 is solely responsible for $SDE2_{FL}$ cleavage across multiple cell lines. Calculated NanoBRET® ratios in **(A)** U2OS, **(B)** HeLa and **(C)** MCF-7 cells treated with USP5 siRNA, USP13 siRNA, combination of both siRNA, or control siRNA. NT = not treated; WT = wild type; UC = uncleavable; USP5/13 = treated with both USP5 and USP13 siRNA; Graph depicts the mean calculated NanoBRET® ratios (dots) + SEM of n=3 independent biological replicates (bar). One way ANOVA was performed to calculate P values, and levels of significance are denoted as follows: ** P<0.01; *** P<0.001; ****P<0.0001, ns=not significant P>0.05. Western blot analysis of **(D)** HEK293, U2OS, HeLa and **(E)** MCF-7 cells treated for 72 h with siRNA targeting USP5, USP13, SDE2 or control siRNA. * = nonspecific band. Source data are provided as a Source Data file.

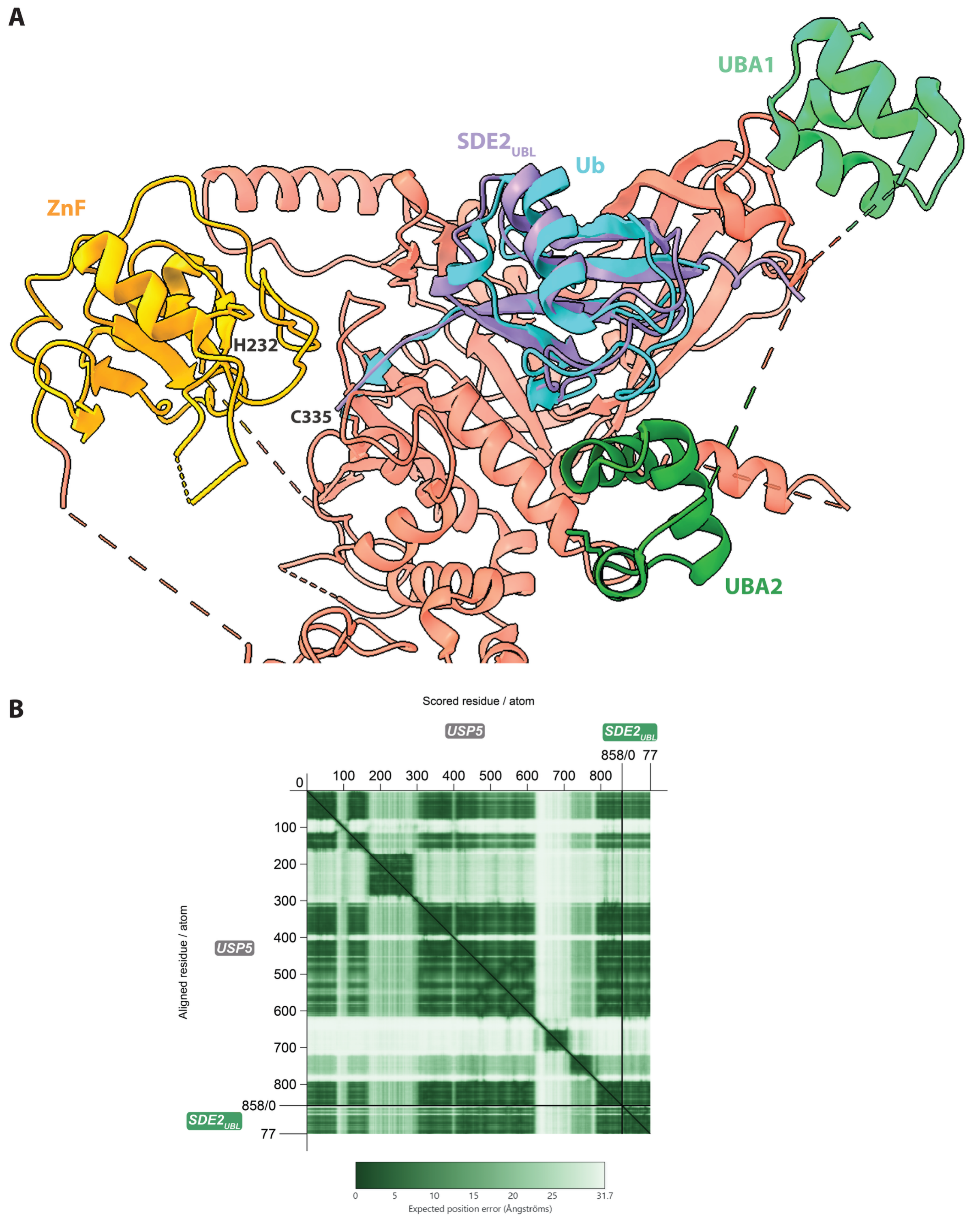

Supplementary Figure 10 | SDE2$_{UBL}$ was modelled in a USP5 bound state using AlphaFold 3.[3] **(A)** SDE2$_{UBL}$ structure was extracted and superimposed onto Ub (RMSD = 2.029) in a solved USP5-Ub crystal structure (PDB: 3IHP). Legend: Ub – cyan blue, SDE2$_{UBL}$ - purple, USP5 – light tomato red, ZnF – orange, UBA1 – light green, UBA2 – dark green. Mutations were introduced in each of the key Ub binding sites and are indicated. **(B)** Predicted alignment error plot corresponding to the displayed AlphaFold 3 model.

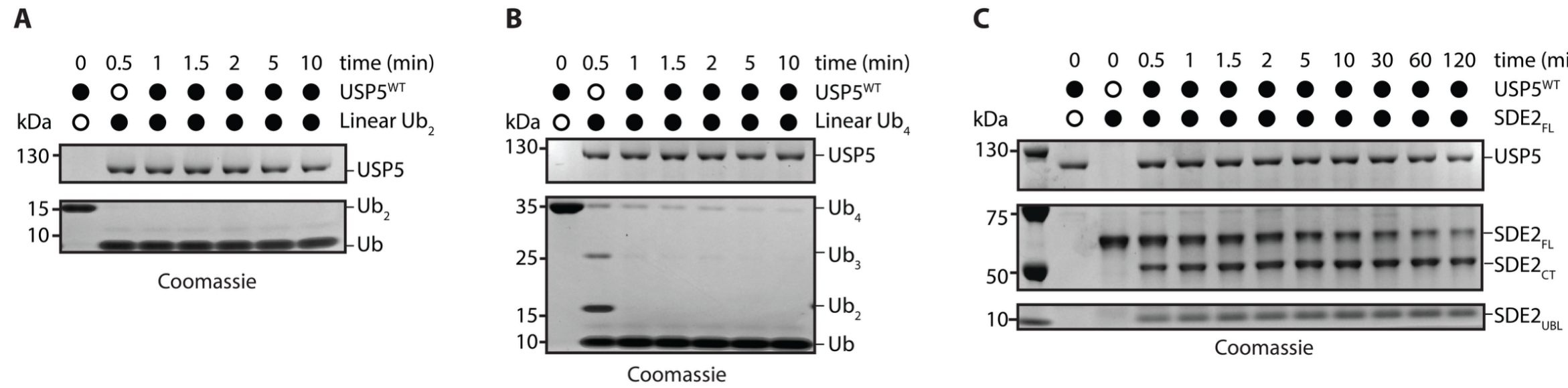

Supplementary Figure 11 | Comparison of USP5-mediated cleavage of SDE2$_{FL}$ and Ub substrates. USP5^{WT} (1 µM) was incubated at 37 °C with **(A)** linear diubiquitin (Ub$_2$, 3 µM), **(B)** linear tetraubiquitin (Ub$_4$, 3 µM), or **(C)** SDE2$_{FL}$ (F, 3 µM). Samples were taken at the indicated time points and cleavage assessed by SDS-PAGE and Coomassie staining.

Supplementary Table 1: *ITC result from the titration of SDE2 constructs into USP5 variants. Reported values are derived from two replicates of each condition. Key: B.L.Q= below limit of quantification. Some evidence of weak binding; N/D= not determined due to weak binding; No binding= no evidence of binding.*

USP5 construct	SDE2 construct (residues)	K_d (µM)	N	ΔH
WT	FL (1-451)		B.L.Q	
	CT (78-451)		No binding	
	UBL (1-77)	26.6 ± 5.0	N/D	N/D
	UBL (1-76)		B.L.Q	
C335A	UBL (1-77)	1.67 ± 0.62	2.01 ± 0.04	-7.46 ± 0.34
	UBL (1-76)	34.3 ± 14.6	N/D	N/D
C335R	UBL (1-77)	2.15 ± 0.55	0.988 ± 0.083	-17.6 ± 1.7
	UBL (1-76)	34.4 ± 14.6	N/D	-13.4 ± 1.3
H232A	UBL (1-77)		No binding	
ΔZnF (Δ197-269)	UBL (1-77)		No binding	

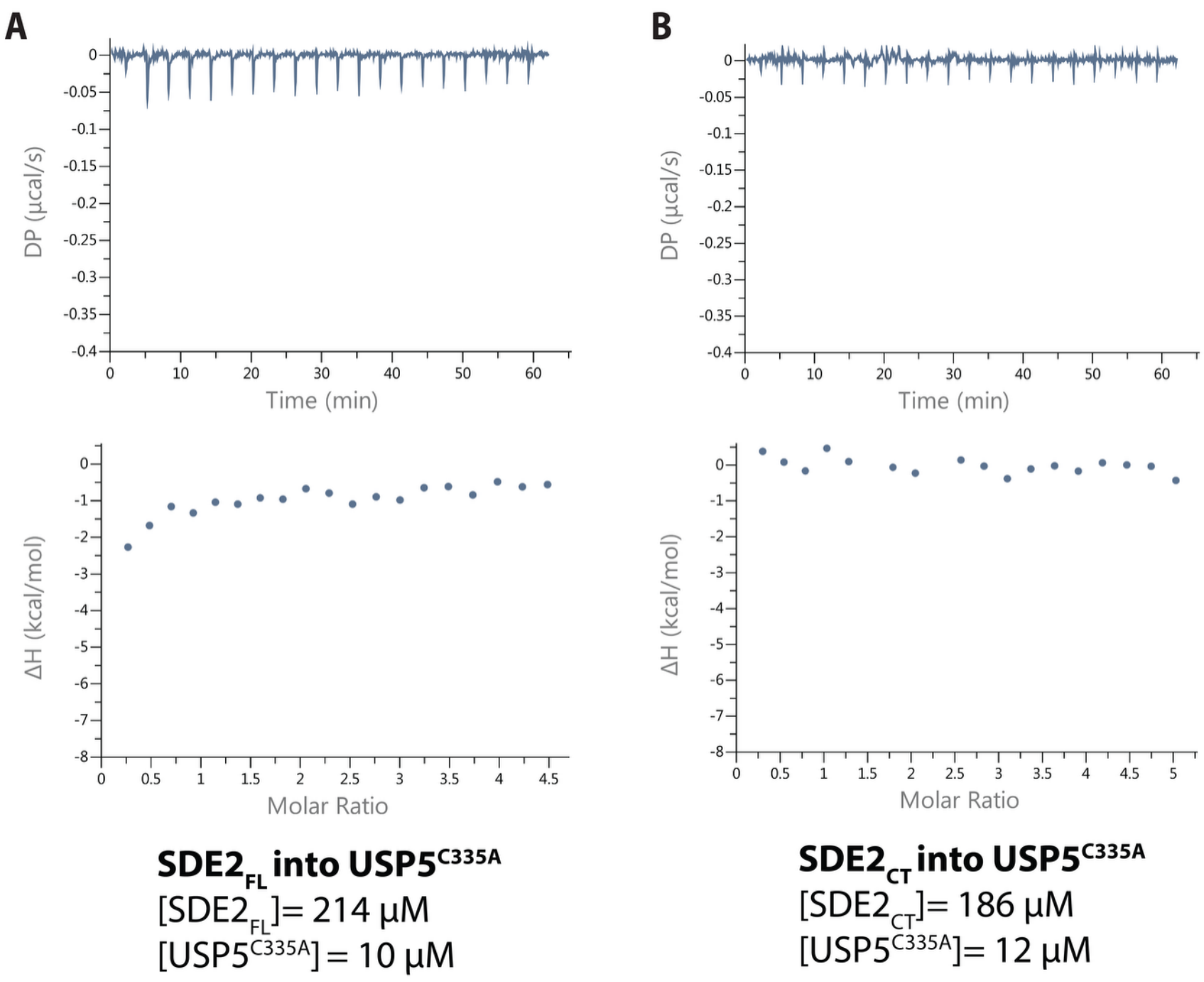

Supplementary Figure 12 | Assessment of the binding interaction between SDE2$_{FL}$ and SDE2$_{CT}$ and USP5^{C335A} by ITC. SDE2$_{FL}$ (A, 206 µM) or SDE2$_{CT}$ (B, 186 µM) were titrated into USP5^{C335A} (10-12 µM). Isotherm curves are representative of n=2 independent experiments. The K_d and N are presented as the mean + SEM of the two experiments.

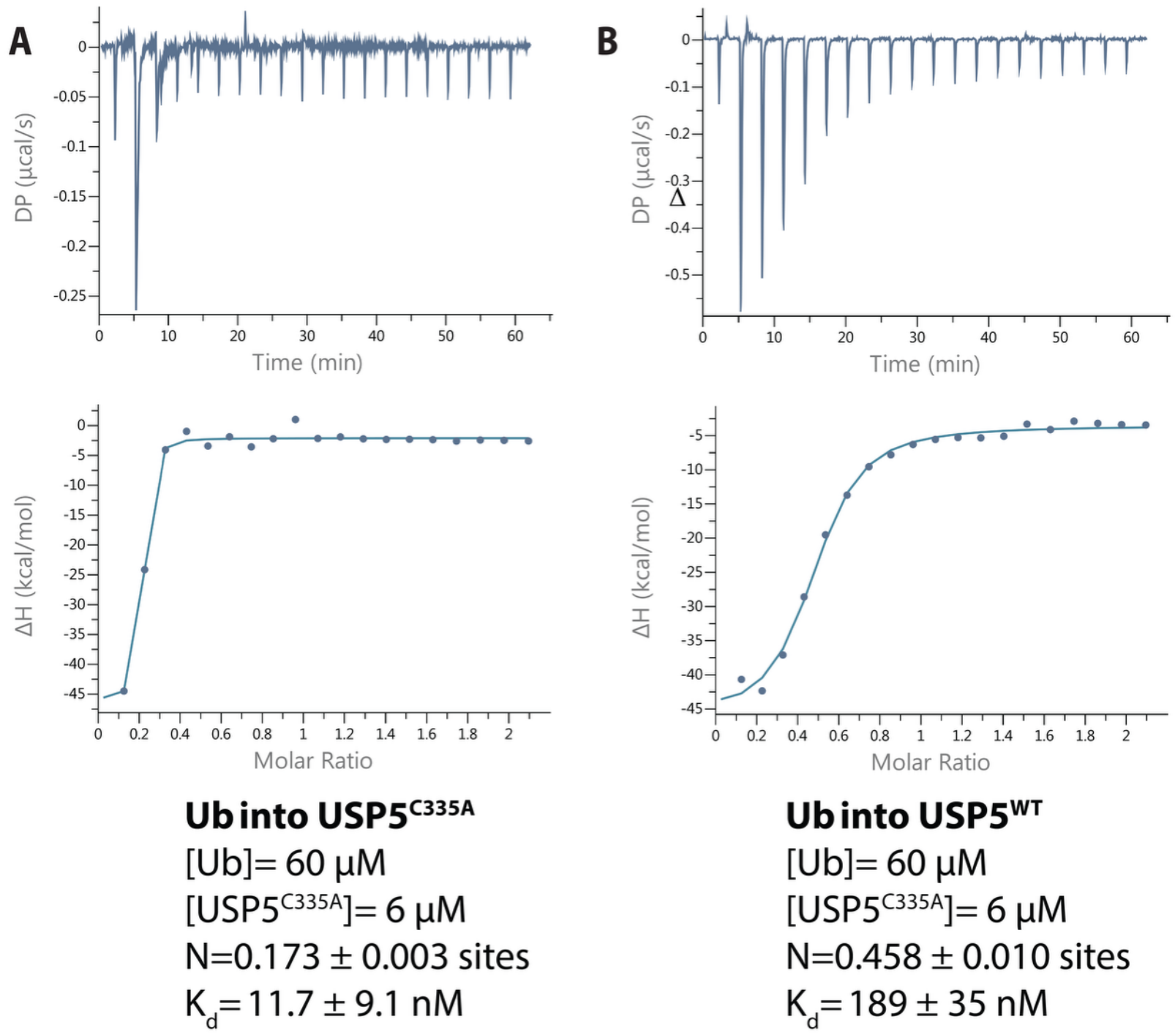

Supplementary Figure 13 | Assessment of the binding interaction between monoubiquitin (Ub) and USP5^{WT} and C335A by ITC and TR-FRET. (A-B) Using ITC, Ub (60 µM) was titrated into USP5^{WT} (60 µM, A) or USP5^{C335A} (60 µM, B). Isotherm curves are representative of n=2 independent experiments. The K_d and N are presented as the mean + SEM of the two experiments.

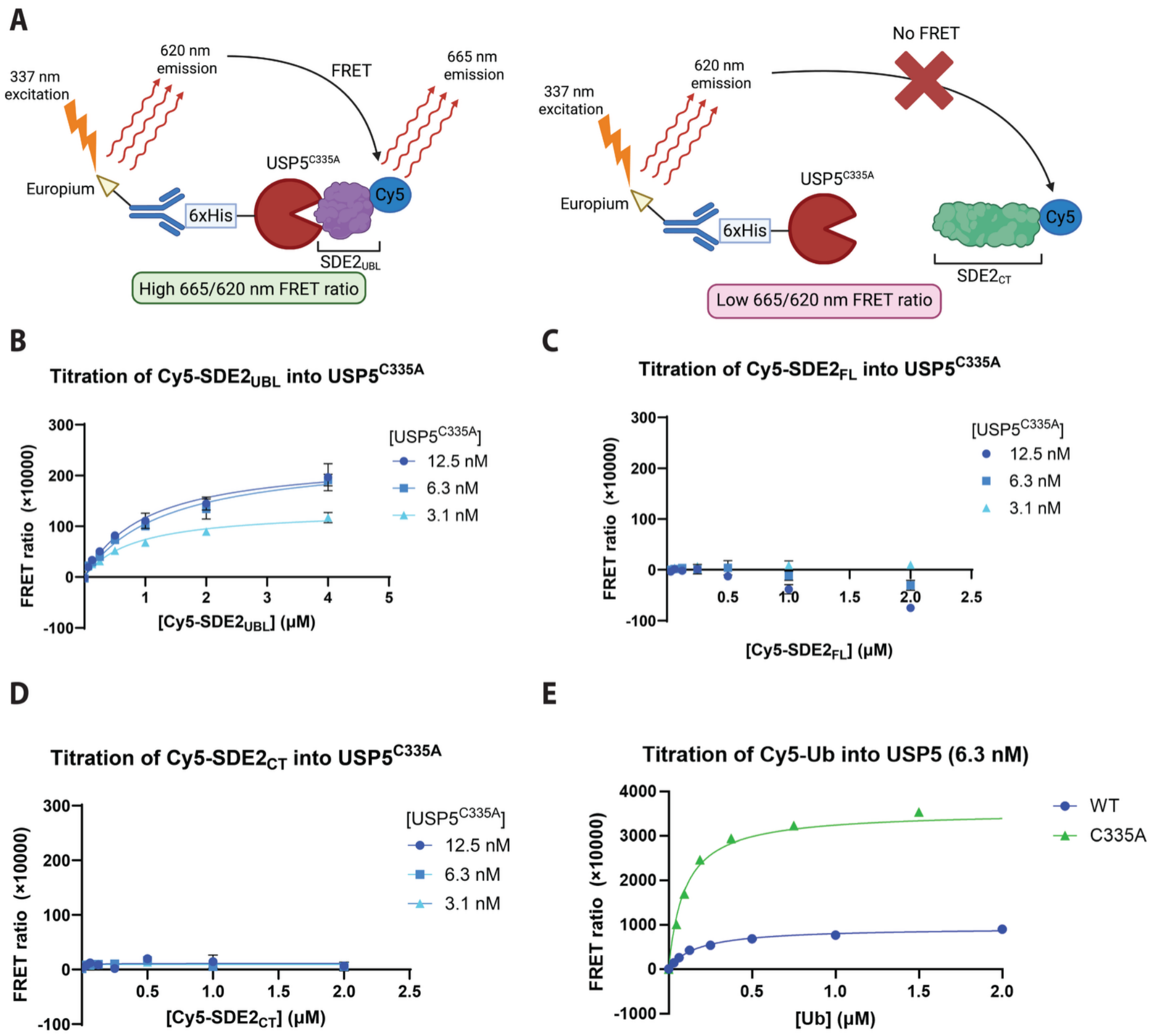

Supplementary Figure 14 | TR-FRET assessment of SDE2$_{FL}$ (A) A TR-FRET assay was established to monitor binding between USP5 and SDE2 domains. His$_6$-tagged USP5 is preincubated with an Eu-tagged His$_6$- antibody (FRET donor), followed by the addition of a Cy5-labelled SDE2 construct (FRET acceptor). The ratio of light emitted at 665 nm to 620 nm is calculated to quantify binding affinity. Cy5-SDE2$_{UBL}$. Created in BioRender. Maniaci, C. (2026) https://BioRender.com/c1w8j56 (**B**), Cy5-SDE2$_{FL}$ (**C**) or Cy5-SDE2$_{CT}$ (**D**) were titrated into USP5^{C335A} (at 3.1, 6.3 or 12.5 nM) and the FRET ratio was measured. An increasing FRET ratio indicative of binding was observed upon addition of Cy5-SDE2$_{UBL}$, but not Cy5-SDE2$_{FL}$ or Cy5-SDE2$_{CT}$. (**E**) Cy5-Ub was titrated into USP5^{WT} or USP5^{C335A} (at 6.3 nM). TR-FRET data is derived from n=3 technical triplicates and points are presented as the mean ± SEM. Curves fitted using a one-site specific curve to calculate a K_d. Source data are provided as a Source Data file.

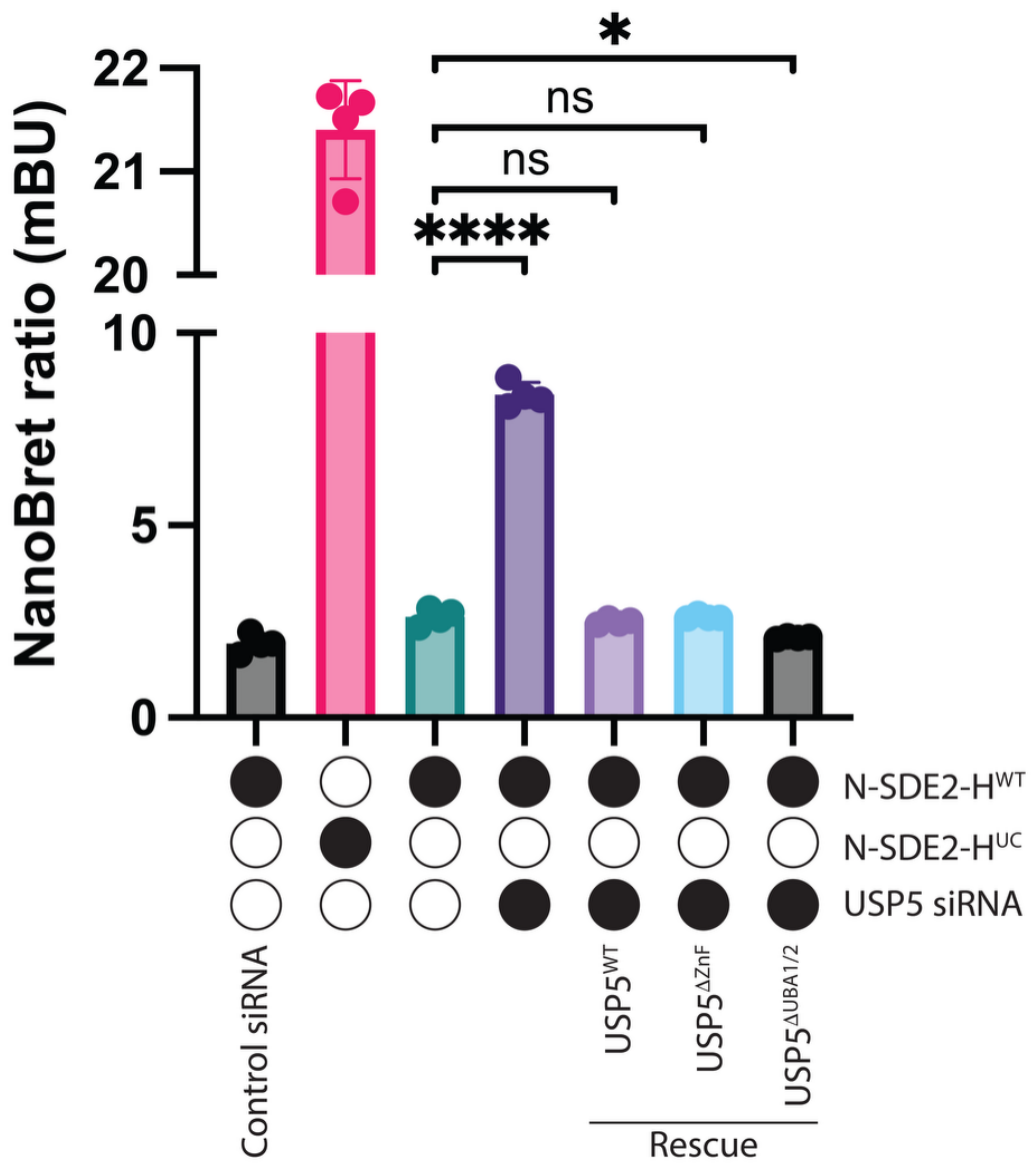

Supplementary Figure 15 | Truncated USP5 constructs cleave $SDE2_{FL}$ in overexpression system. NanoBRET® assay was conducted in HEK293 cells after USP5 knockdown with siRNA. Rescues were performed by exogenously expressing truncated USP5 constructs lacking either the ZnF-UBP domain (ΔZnF), or both UBA domains (ΔUBA1/2). WT= wild-type, UN= uncleavable. Graph depicts the mean calculated NanoBRET® ratios (dots) + SEM of n=3 independent biological replicates (bar). One way ANOVA was performed to calculate P values, and levels of significance are denoted as follows: * P<0.05; **** P<0.0001, ns=not significant P>0.05.

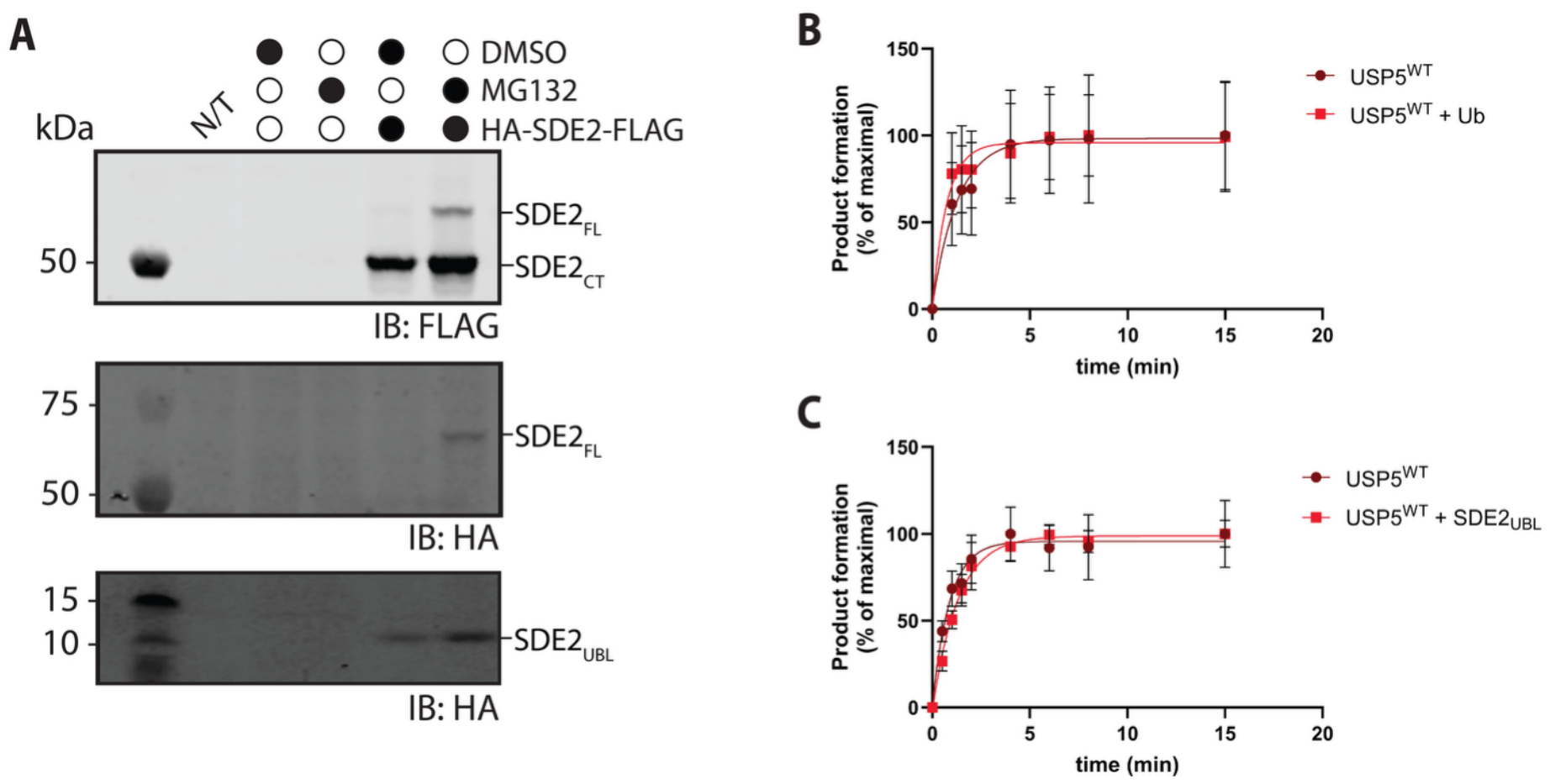

Supplementary Figure 16 | Investigation into the effect of excess $SDE2_{UBL}$ on the processing of $SDE2_{FL}$ (A) HEK239 cells were transfected with a plasmid encoding HA-$SDE2_{FL}$-FLAG in the presence or absence of proteasome inhibitor MG132. Accumulation of $SDE2_{UBL}$ corresponded with impaired $SDE2_{FL}$ processing. **(B-C)** Normalised accumulation of $SDE2_{CT}$ over a 0-15 min reaction of $SDE2_{FL}$ (3 µM) and USP5 (100 nM) at 25 °C, in the absence or presence of Ub (B, 30 µM) or $SDE2_{UBL}$ (C, 30 µM). Samples were collected at indicated time points, assessed by SDS-PAGE and immunoblotting for quantification. Data is representative of triplicate experiments presented as mean ± SD. One-phase non-linear association and an extra sum-of-squares F-test were performed which found no significant difference in rate constants (K) between untreated and either Ub (F = 1.152, P = 0.2893) or $SDE2_{UBL}$ (F = 4.258, P = 0.0445) treatment. Source data are provided as a Source Data file.

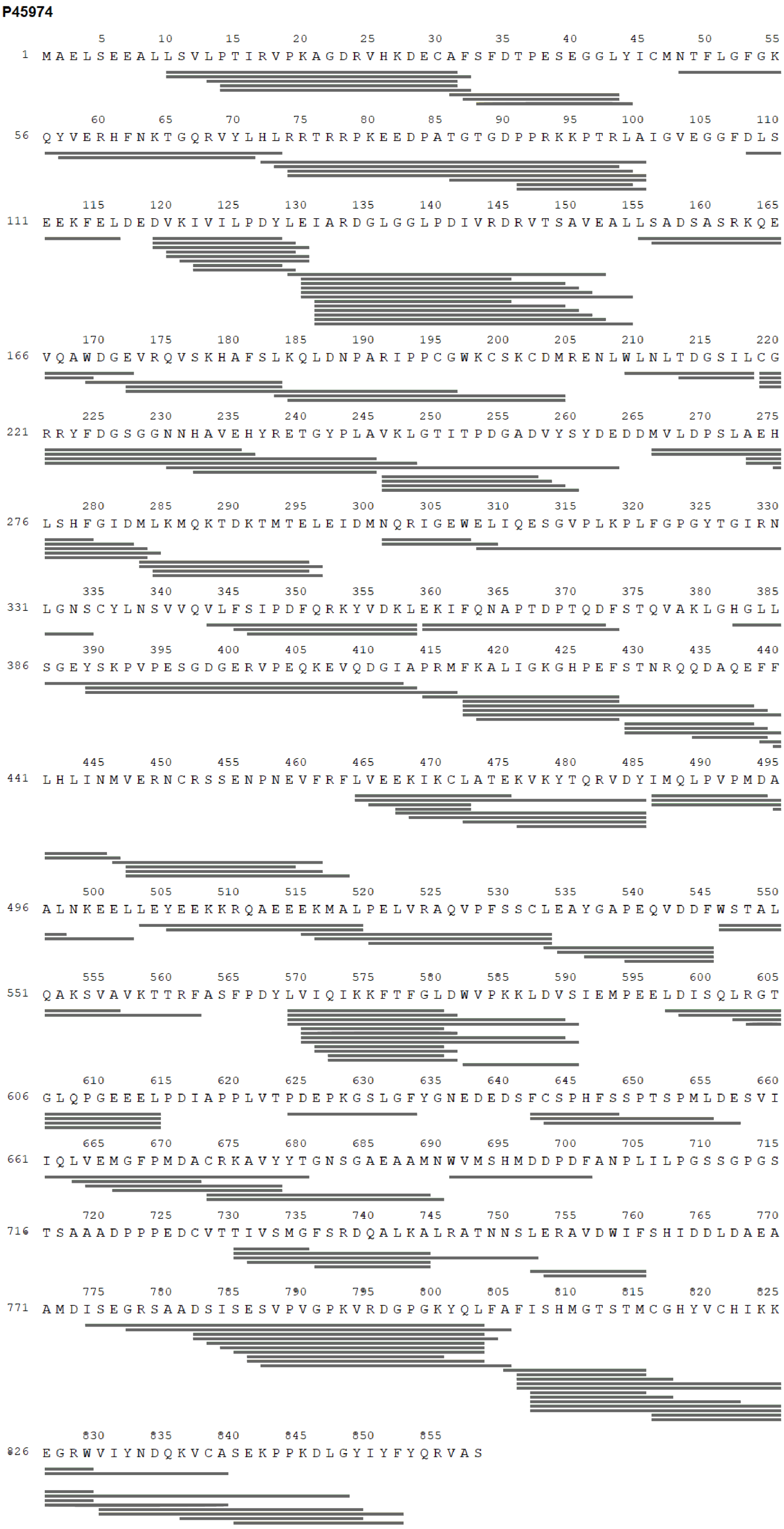

Supplementary Figure 17 | HDX-MS coverage map for USP5 (UniProt: P45974). Grey bars represent identified peptides

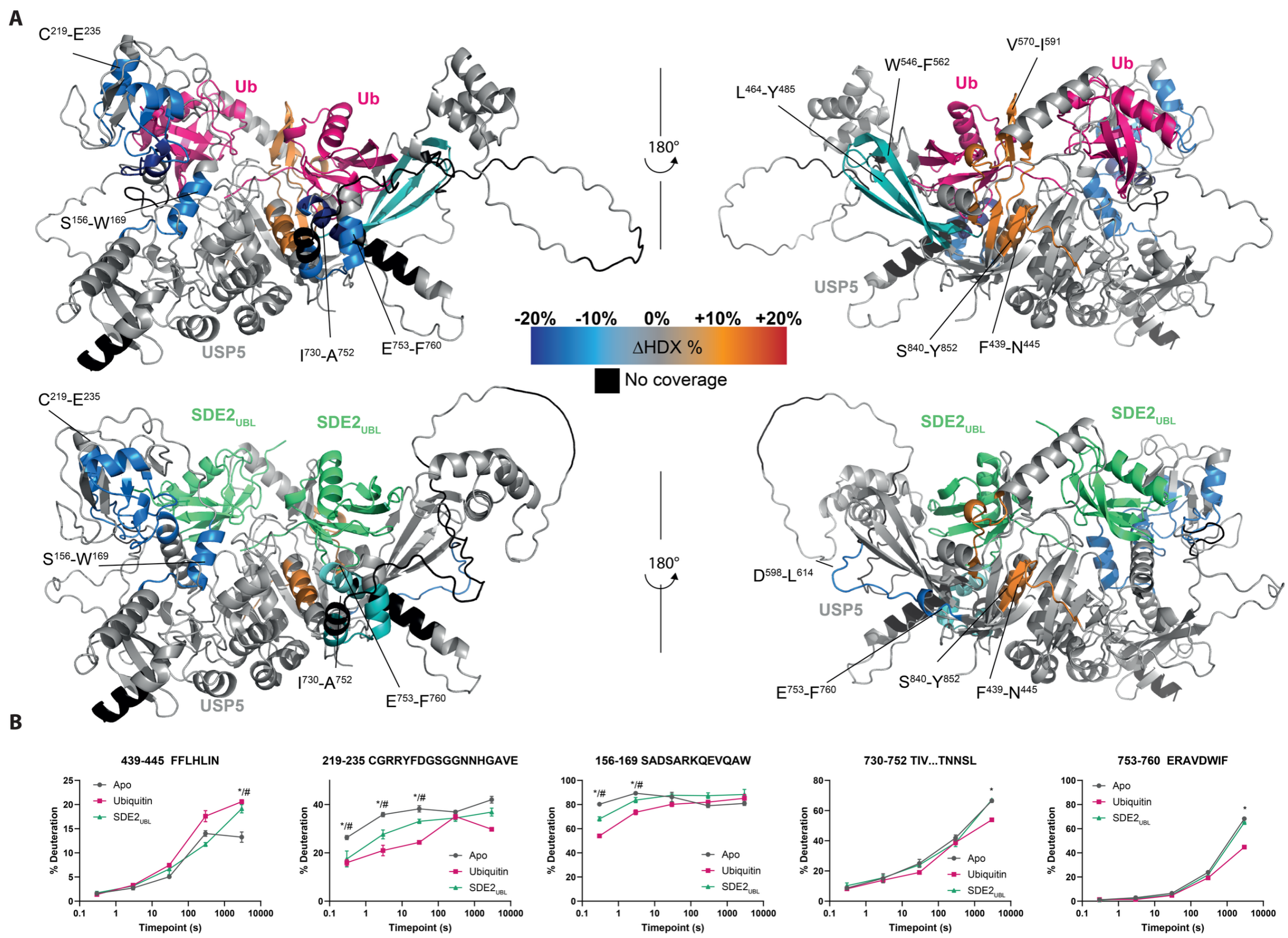

Supplementary Figure 18 | Comparison of binding models of Ub and SDE2UBL with USP5WT informed by HDX-MS. (A) AlphaFold 3[3] was used to predict the binding of $USP5^{WT}$ with two molecules of Ub or $SDE2_{UBL}$. Hydrogen-deuterium exchange (HDX) experiments were performed with recombinant $USP5^{WT}$ (6 µM) and Ub or $SDE2_{UBL}$ (200 µM) and HDX perturbation (ΔHDX) was mapped onto the respective models. **(B)** Deuteration profiles of key USP5 peptides across the three treatment groups. * indicates p≤0.05 between Apo and Ub, # indicates p≤0.05 between Apo and $SDE2_{UBL}$.

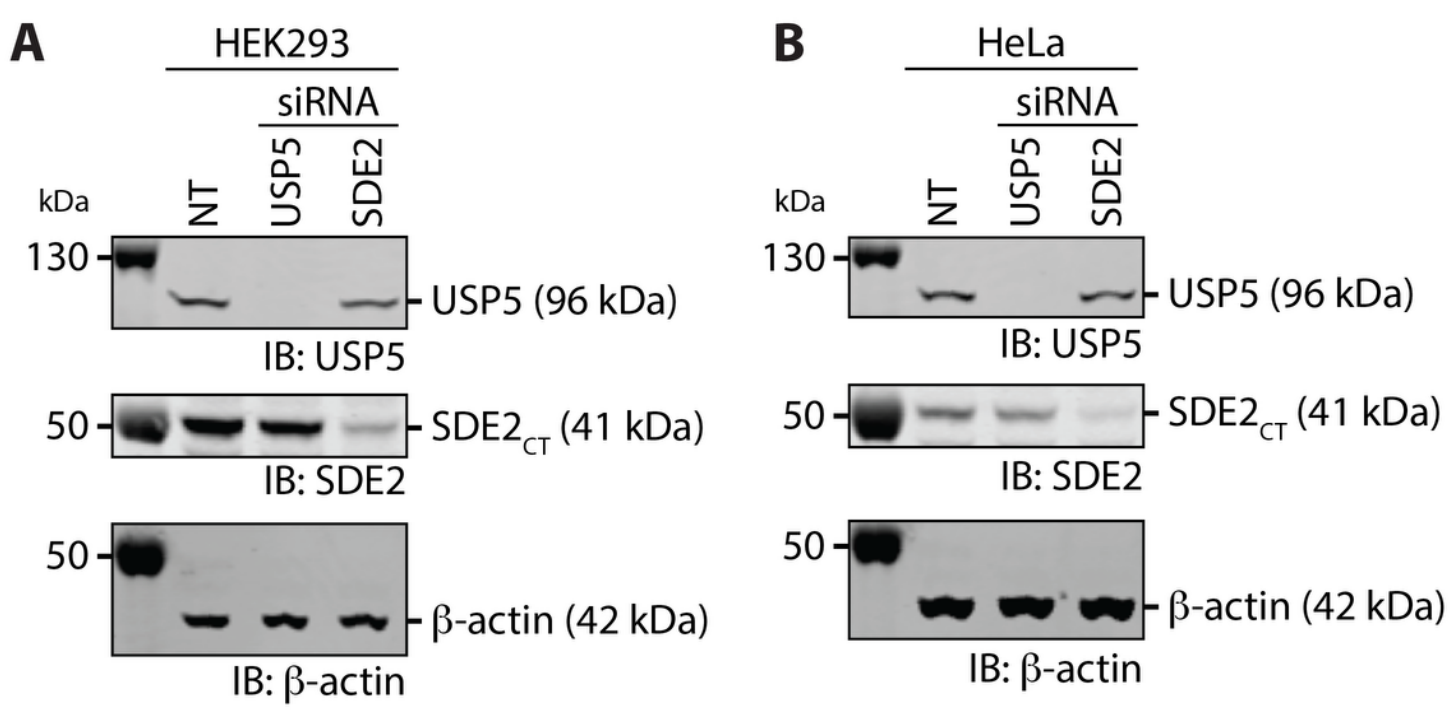

Supplementary Figure 19 | Confirmation of USP5 and SDE2 KD in intron retention PCR experiment. USP5 and SDE2 were knocked down (KD) using siRNA in (A) HEK293 and (B) HeLa cells. KD was confirmed via immunoblotting against USP5 and SDE2; β-actin was used as a control. NT – non-treated.

Supplementary Table 2: *HDX-MS Table*

	Apo USP5	USP5 + Ub	USP5 + SDE2$_{UBL}$
**# Of peptides**	177	179	179
**Coverage (%)**	86.7	86.5	85.9
**Gaps in coverage**	1-10, 205-210, 335-345, 564-571, 590-598, 635-642, 703-730, 760-775, 854-858		
**Av. Length**	15.8		
**Redundancy**	3.5		
**Time points**	0.3s[1] / 3s/ 30s[2]/ 300s[2]/ 3000s[2]		
**Replicates**	3 ND, 3D		
**Replicability[3]**	1.83%	1.75%	1.79%
**Significance threshold[4]**	>7.5%/1.0 Da/ passes unpaired t-test with $P < 0.05$		

[1]0.3 s timepoint created by pipetting 3 s by hand with ice-cold solutions.
[2]Conducted by liquid handling robot.
[3]Mean standard deviation between replicates
[4]A peptide must meet all these criteria to have a significant change in solvent exchange.

Reagents

Supplementary Table 3*: Plasmids used in this study, registered in the Reagents & Services database, University of Dundee (DU number)*

NAME	PROTEIN	MUTATIONS	WT UNIPROT CODE	TAG	BACKBONE AND VECTOR TYPE	DU NUMBER
**6×His-SUMO-SDE2**	SDE2	none	Q6IQ49	N-term 6xHis SUMO	pET15b (bacterial)	DU75576
**HA-SDE2$_{UBL}$ (1-76)-intein-CBD**	SDE2	Truncation (aa 1-76)	Q6IQ49	N-term HA	pTXB1 (mammalian)	DU78718
**Halo-SDE2-Nano**	SDE2	none	Q6IQ49	N-term NanoLuc, C-term HaloTag	pF Nanoluc SDE2 Halo (mammalian)	DU75755
**USP5^{WT}**	USP5	none	P45974	N-term 6×His	pET15b (bacterial)	DU15641
**USP5^{C335A}**	USP5	C335A	P45974	N-term 6×His	pET15b (bacterial)	DU75867
**USP5^{H232A}**	USP5	H232A	P45974	N-term 6×His	pET15b (bacterial)	DU78711

**USP5^{C335R}**	USP5	C335R	P45974	N-term 6×His	pET15b (bacterial)	DU78704
**USP5^{M666E}**	USP5	M666E	P45974	N-term 6×His	pET15b (bacterial)	DU78705
**USP5^{M734E}**	USP5	M734	P45974	N-term 6×His	pET15b (bacterial)	DU78706
**USP5$^{H232A\ C335R}$**	USP5	H232A C335R	P45974	N-term 6×His	pET15b (bacterial)	DU78707
**USP5$^{H232A\ M666E}$**	USP5	H232A M666E	P45974	N-term 6×His	pET15b (bacterial)	DU78708
**USP5$^{H232A\ M743E}$**	USP5	H232A M734E	P45974	N-term 6×His	pET15b (bacterial)	DU78709
**USP5$^{C3335R\ M666E}$**	USP5	C335R M666E	P45974	N-term 6×His	pET15b (bacterial)	DU78710
**USP5$^{C335R\ M743E}$**	USP5	C335R M734E	P45974	N-term 6×His	pET15b (bacterial)	DU78720
**USP5$^{\Delta ZnF}$**	USP5	Deletion of ZnF-UBP (residues 197-269)	P45974	N-term 6×His	pET15b (bacterial)	DU83405
**FLAG-USP5^{WT}**	USP5	none	P45974	N-term FLAG	pCMV5D (mammalian)	DU13505
**FLAG-USP5^{C335A}**	USP5	C335A	P45974	N-term FLAG	pCMV5D (mammalian)	DU78075
**FLAG- USP5 $^{\Delta ZnF}$**	USP5	Deletion of ZnF-UBP (residues 197-269)	P45974	N-term FLAG	pCMV5D (mammalian)	DU83406
**FLAG- USP5 $^{\Delta UBA1/2}$**	USP5	Deletion of UBA1 and UBA 2 domains (residues 654-762)	P45974	N-term FLAG	pCMV5D (mammalian)	DU83407
**GFP-USP5**	USP5	none	P45974	N-term GFP	pcDNA5D FRT/TO GFP (mammalian)	DU25397
**GFP-USP13**	USP13	none	Q92995	N-term GFP	pcDNA5D FRT/TO GFP (mammalian)	DU24495

Supplementary Table 4: Recombinant proteins obtained from Reagents and Services, University of Dundee

DU number	Construct	MW (Da)
**DU13025**	GST-USP2(2-353)	72,686
**DU15644**	His_6-USP7(1-1102)	130,633
**DU10952**	GST-USP13(318-863)	88,475
**DU14352**	His_6-USP14(1-494)	58,476
**DU46239**	GST-USP16(1-823)	120,318
**DU22385**	GST-USP21(196-565)	67,325
**DU20027**	Monoubiquitin	8,559
**DU20729**	Ubiquitin linear dimer	17,640
**DU20027-K11**	Ubiquitin dimer (K11)	17,640
**DU20027-K63**	Ubiquitin dimer (K63)	17,640
**DU20766**	Ubiquitin tetramer (linear)	34,745

Supplementary Table 5: DNA oligos used for intron retention PCR.

Gene	Type	Sequence (5' to 3')
**CUL7**	Forward	TGAGCGAGGCTCACAGA
	Reverse	TTTGCTCGTGAAGGTCCAG
**KIFC2**	Forward	TCTTCAGAGAGCTGGAACCT
	Reverse	CTCCACCATGCTGAGTGTC
**SPATA20**	Forward	TTGATGGTGTCAGGCTAT
	Reverse	AAGAGCTTGTCCTGTGTGTC
**FBF1**	Forward	AGCCTGCACGAGTTGTC
	Reverse	CCGGCTCTGCTCATTCA
**RFNG**	Forward	TTTGCCACGTGGATGATGA
	Reverse	CCATGGGCTCATCTTGAGG

Immunoprecipitation and mass spectrometry

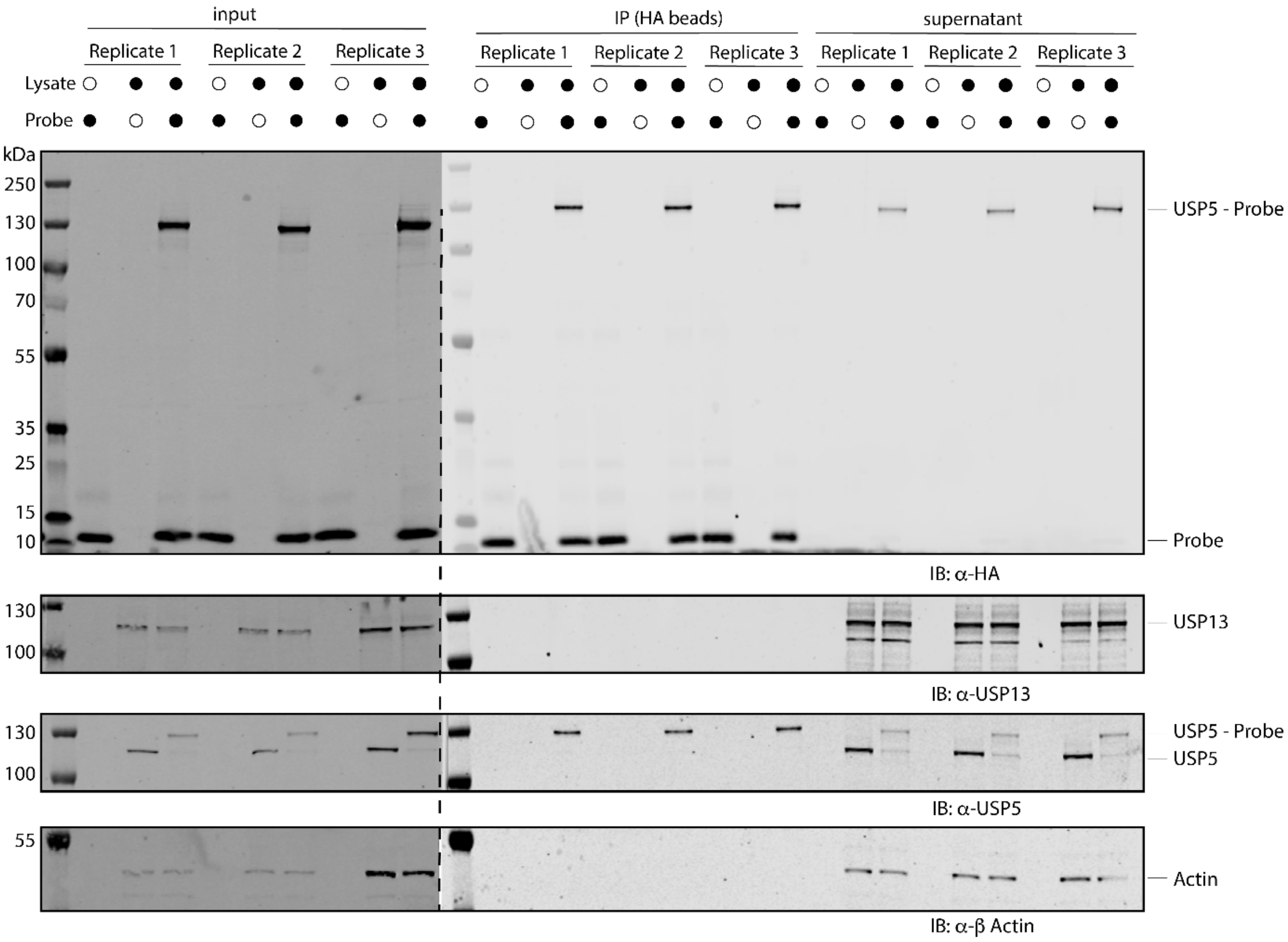

Supplementary Figure 20 | Immunoblot analysis of immunoprecipitation samples prepared for activity-based probe profiling using HA-SDE2$_{UBL}$-PA. In triplicate, HEK293T cell lysate was incubated with HA-SDE2$_{UBL}$-PA (1 μM) for 45 min at 37 °C and immunoprecipitated with anti-HA beads. Unbound sample (supernatant) was removed and bound protein eluted (IP) by boiling.

Characterisation of ABPs

HA-SDE2$_{UBL}$ΔG77-PA (HA-SDE2$_{UBL}$-PA)

Sequence:
MYPYDVPDYAGGGGSAEAAALVWIRGPGFGCKAVRCASGRCTVRDFIHRHCQDQNVPVENF FVKCNGALINTSDTVQHGAVYSLEPRLCG-*propargylamine*

Calculated mass (Expasy- ProtParam): 9730

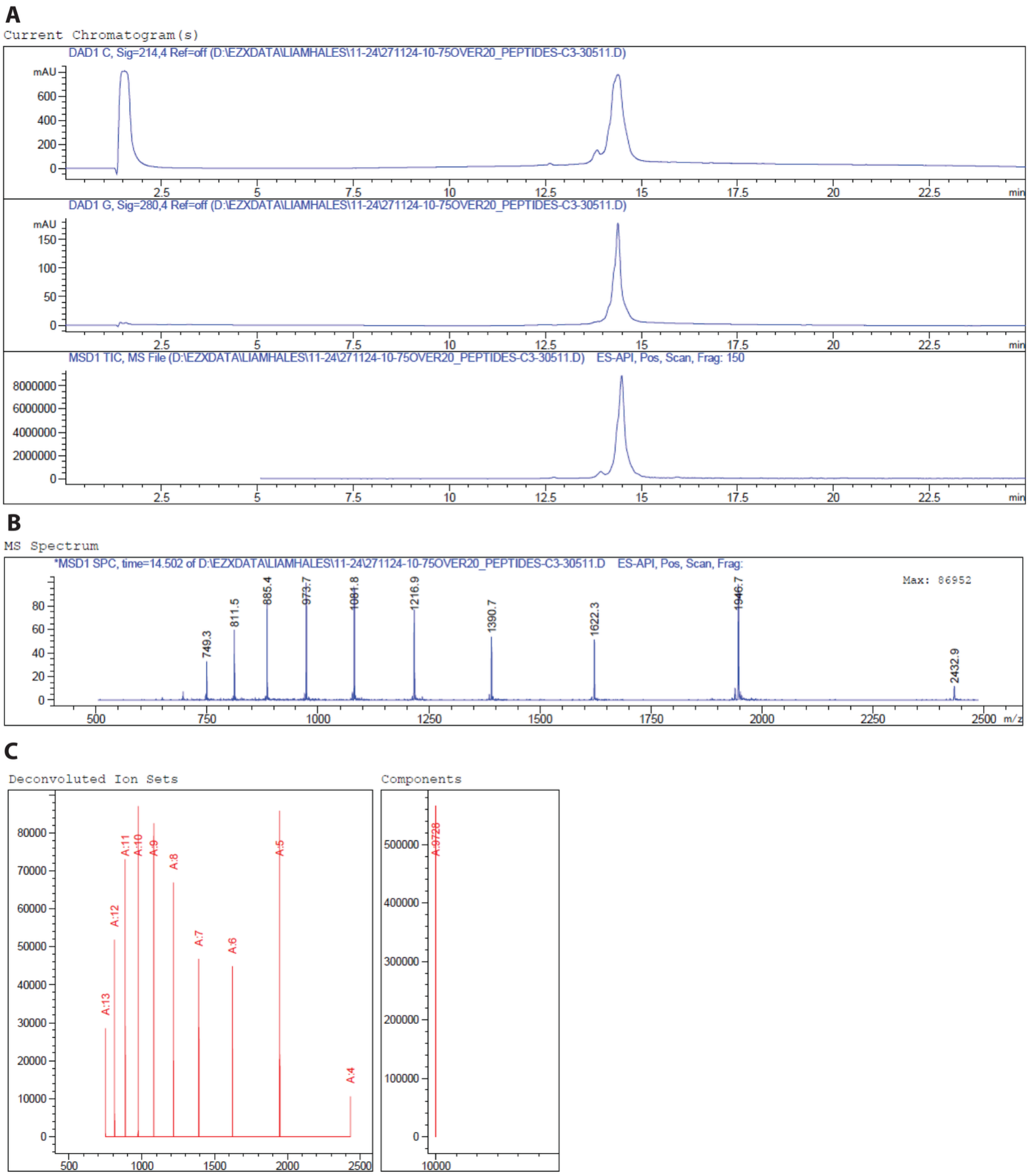

Supplementary Figure 21 | LCMS analysis of HA-SDE2$_{UBL}$ΔG77-PA. (A) Chromatogram (214 nm/280 nM) and total ion count (TIC) trace. **(B)** ES-API mass spectrum (positive scan). **(C)** Deconvoluted mass spectrum identifying the parent ion mass.

ISG15$_{CTD}$ΔG157-PA (ISG15-PA)

Sequence:
MDEPLSILVRNNKGRSSTYEVRLTQTVAHLKQQVSGLEGVQDDLFWLTFEGKPLEDQLPLGE
YGLKPLSTVFMNLRLRG-*propargylamine*

Calculated mass (Expasy- ProtParam): 9012

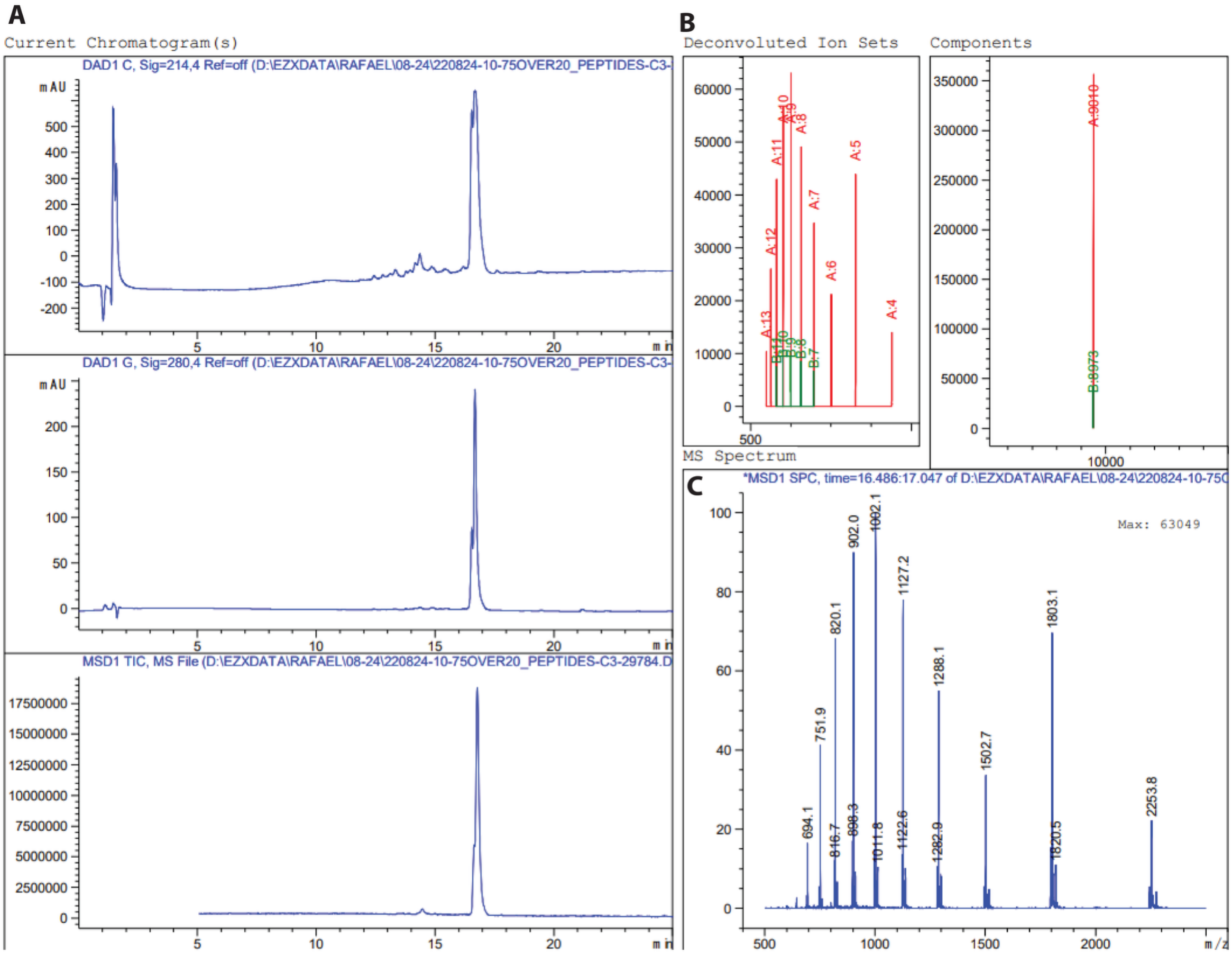

Supplementary Figure 22 | LCMS analysis of ISG15$_{CTD}$ΔG157-PA. (A) Chromatogram (214 nm/280 nM) and total ion count (TIC) trace. **(B)** Deconvoluted mass spectrum identifying the parent ion mass. **(C)** ES-API mass spectrum (positive scan).

His-UbΔG76-PA (Ub-PA)

Sequence:
MHHHHHHHHLEVLFQGPMQIFVKTLTGKTITLEVEPSDTIENVKAKIQDKEGIPPDQQRLIFAG
KQLEDGRTLSDYNIQKESTLHLVLRLRG-*propargylamine*

Calculated mass (Expasy- ProtParam): 10,657

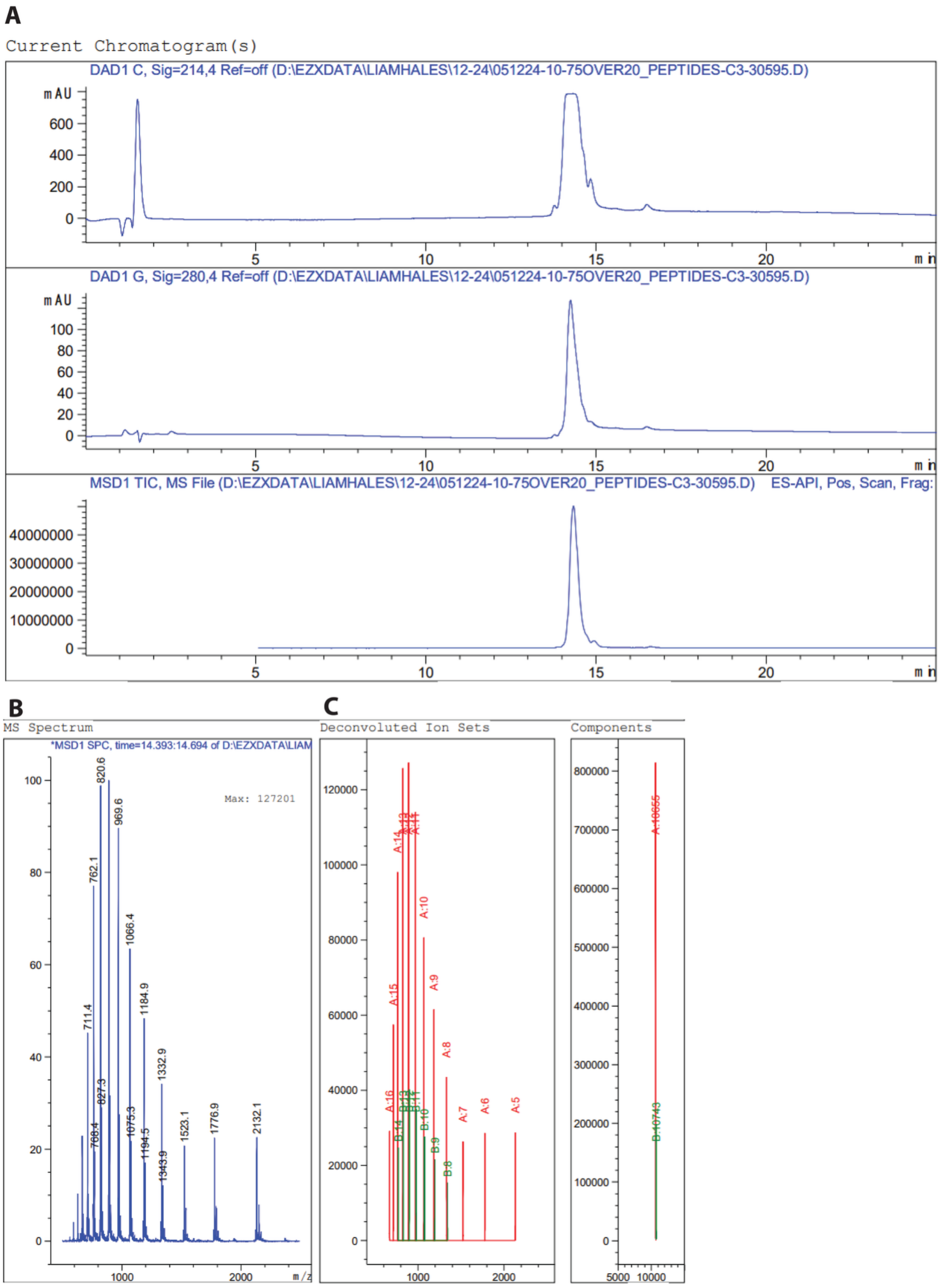

Supplementary Figure 23| LCMS analysis of His-UbΔG76-PA. (A) Chromatogram (214 nm/280 nM) and total ion count (TIC) trace. **(B)** ES-API mass spectrum (positive scan). **(C)** Deconvoluted mass spectrum identifying the parent ion mass.

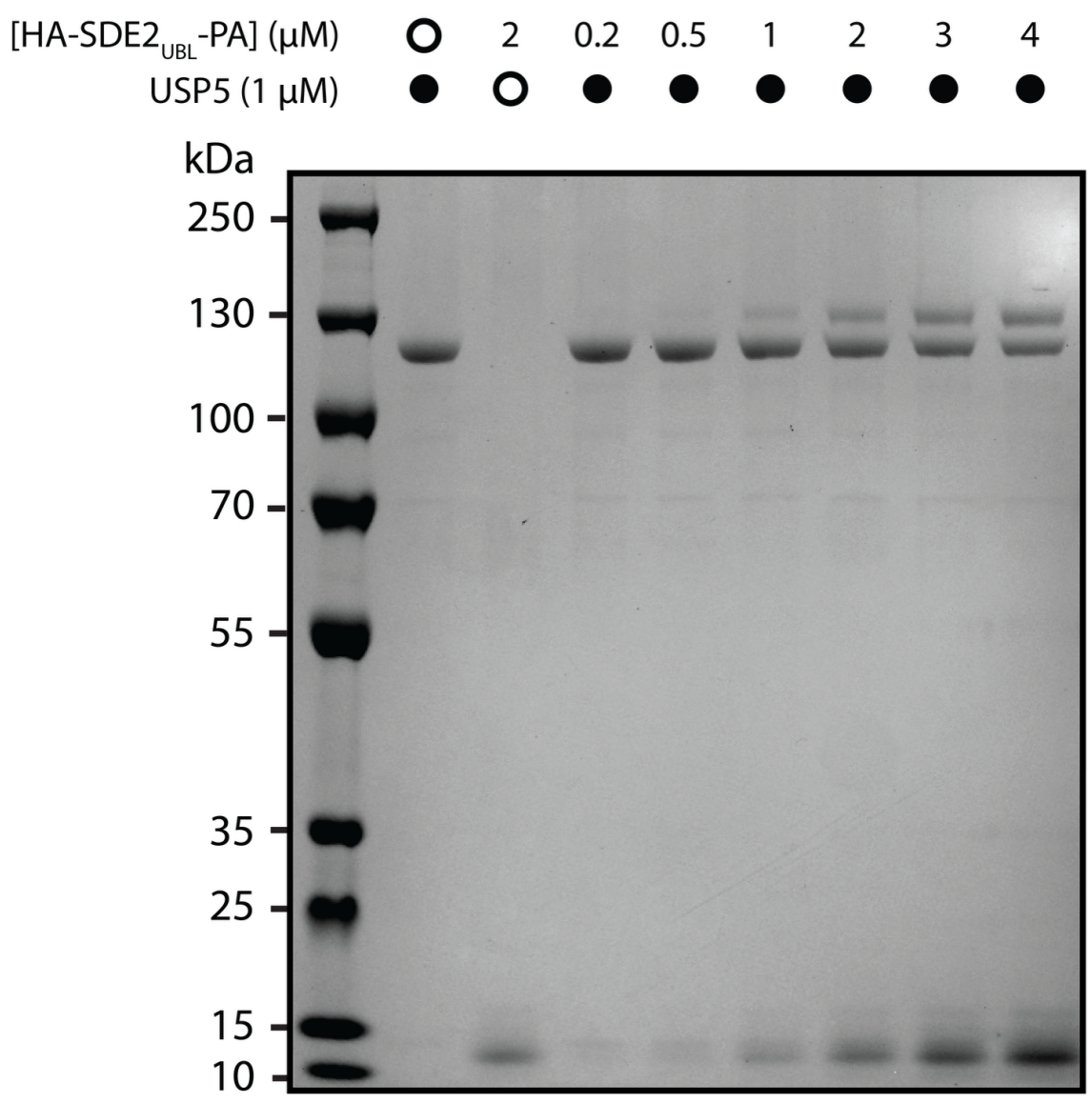

Supplementary Figure 24 | Representative SDE2$_{UBL}$-PA concentration dependant labelling of USP5. USP5 (1 µM) was incubated with HA-SDE2$_{UBL}$-PA at the indicated concentrations for 1 h at 37 °C and analysed by SDS-PAGE. Note: labelling efficiency varies between preparations, thus the appropriate working concentration for each preparation is assessed individually.

Thermal shift assay

Supplementary Table 6: *Melting temperatures obtained from three technical replicates*

	**USP5**			**USP5 + HA-SDE2$_{UBL}$-PA**			**USP5 + His-Ub-PA**			**USP5 + ISG15-PA**		
**Melting temperature (°C)**	53.75	53.75	54.25	56.25	56.75	56.25	58.25	58.75	58.25	57.25	57.75	57.25

Circular Dichroism

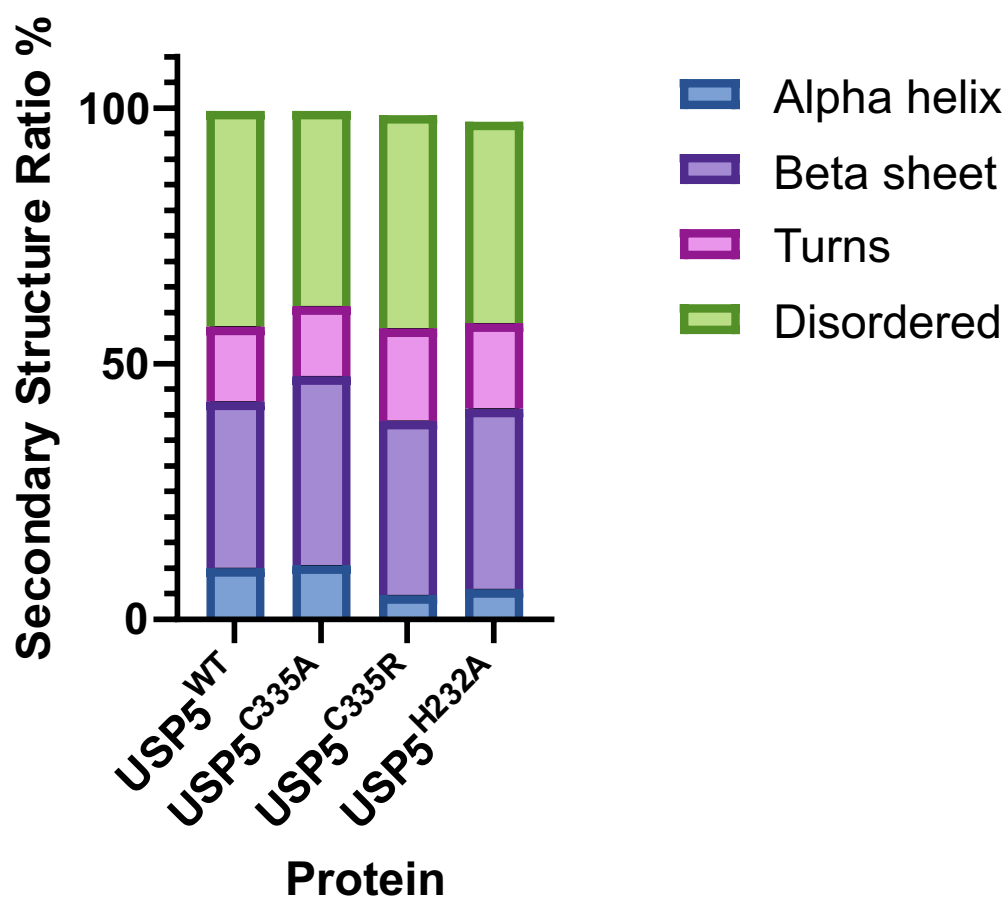

Supplementary Figure 25 | Estimation of secondary structure content for USP5 variants. The absorption profile (190-350 nm) of USP5 variants (4-11 µM) were used to estimate the percentage of secondary structural elements. No significant differences were observed indicating the structural integrity of the mutants.

ITC

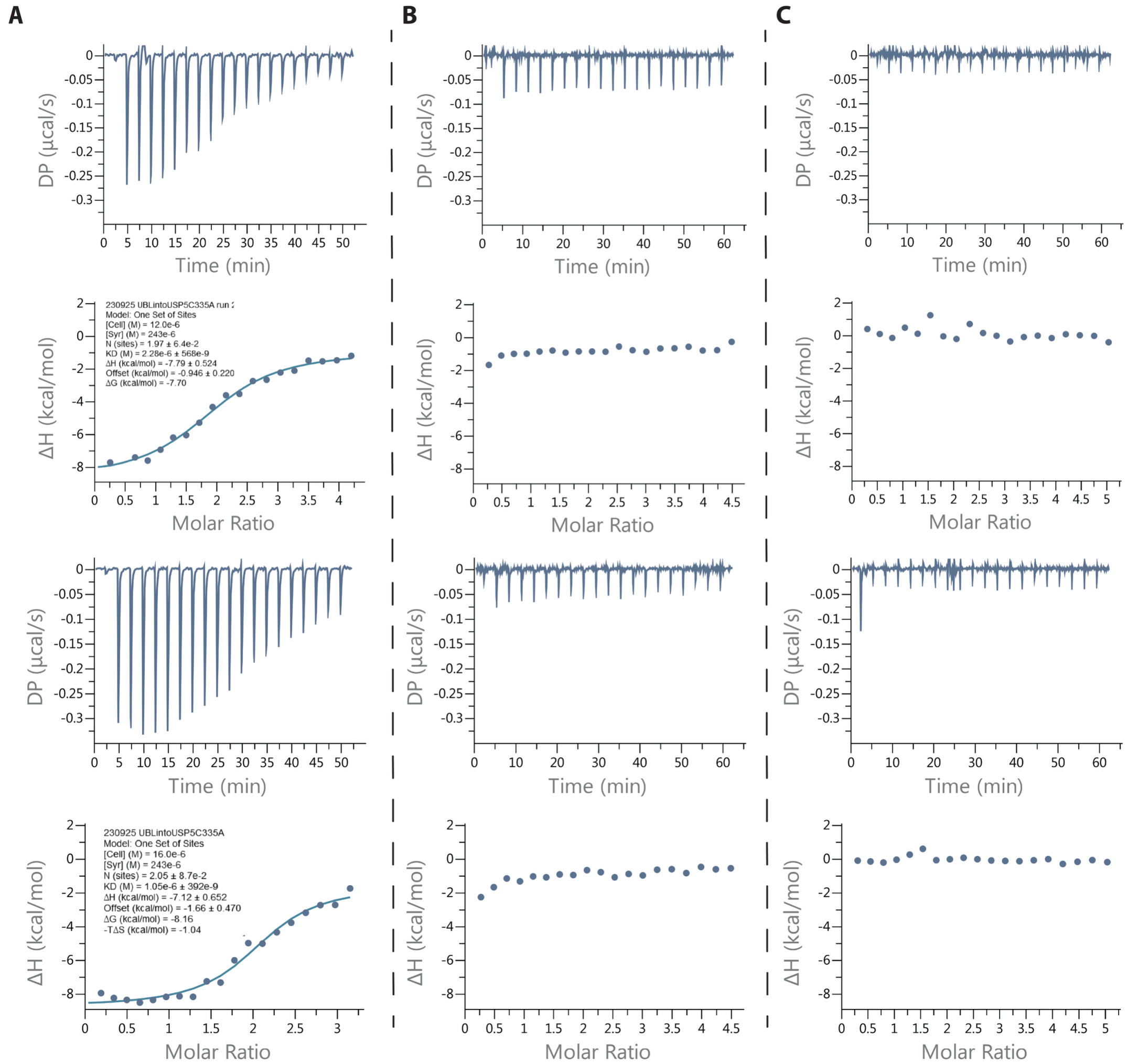

Supplementary Figure 26 | Full ITC data from the titration of SDE2 constructs into USP5^{C335A}
(A) SDE2$_{UBL}$ (243 µM) was titrated into USP5^{C335A} (12-16 µM). **(B)** SDE2$_{FL}$ (214 µM) was titrated into USP5^{C335A} (10 µM). **C.** SDE2$_{CT}$ (186 µM) was titrated into USP5^{C335A} (12 µM).

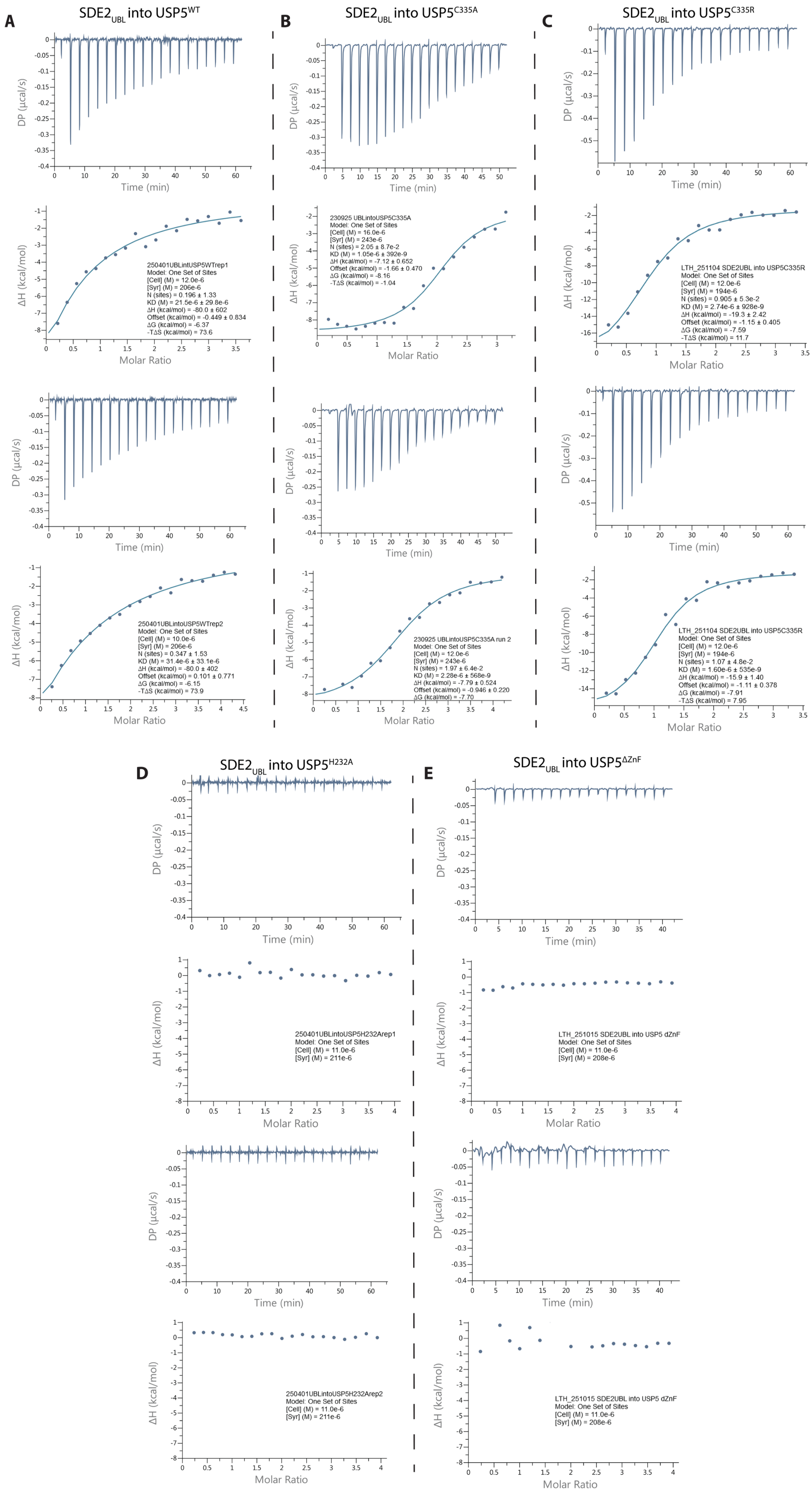

A
SDE2UBL into USP5WT
DP (µcal/s)
Time (min)
ΔH (kcal/mol)
Molar Ratio
250401UBLintUSP5WTrep1
Model: One Set of Sites
[Cell] (M) = 12.0e-6
[Syr] (M) = 206e-6
N (sites) = 0.196 ± 1.33
KD (M) = 21.5e-6 ± 29.8e-6
ΔH (kcal/mol) = -80.0 ± 602
Offset (kcal/mol) = -0.449 ± 0.834
ΔG (kcal/mol) = -6.37
-TΔS (kcal/mol) = 73.6
250401UBLintoUSP5WTrep2
Model: One Set of Sites
[Cell] (M) = 10.0e-6
[Syr] (M) = 206e-6
N (sites) = 0.347 ± 1.53
KD (M) = 31.4e-6 ± 33.1e-6
ΔH (kcal/mol) = -80.0 ± 402
Offset (kcal/mol) = 0.101 ± 0.771
ΔG (kcal/mol) = -6.15
-TΔS (kcal/mol) = 73.9
B
SDE2UBL into USP5C335A
230925 UBLintoUSP5C335A
Model: One Set of Sites
[Cell] (M) = 16.0e-6
[Syr] (M) = 243e-6
N (sites) = 2.05 ± 8.7e-2
KD (M) = 1.05e-6 ± 392e-9
ΔH (kcal/mol) = -7.12 ± 0.652
Offset (kcal/mol) = -1.66 ± 0.470
ΔG (kcal/mol) = -8.16
-TΔS (kcal/mol) = -1.04
230925 UBLintoUSP5C335A run 2
Model: One Set of Sites
[Cell] (M) = 12.0e-6
[Syr] (M) = 243e-6
N (sites) = 1.97 ± 6.4e-2
KD (M) = 2.28e-6 ± 568e-9
ΔH (kcal/mol) = -7.79 ± 0.524
Offset (kcal/mol) = -0.946 ± 0.220
ΔG (kcal/mol) = -7.70
C
SDE2UBL into USP5C335R
LTH_251104 SDE2UBL into USP5C335R
Model: One Set of Sites
[Cell] (M) = 12.0e-6
[Syr] (M) = 194e-6
N (sites) = 0.905 ± 5.3e-2
KD (M) = 2.74e-6 ± 928e-9
ΔH (kcal/mol) = -19.3 ± 2.42
Offset (kcal/mol) = -1.15 ± 0.405
ΔG (kcal/mol) = -7.59
-TΔS (kcal/mol) = 11.7
LTH_251104 SDE2UBL into USP5C335R
Model: One Set of Sites
[Cell] (M) = 12.0e-6
[Syr] (M) = 194e-6
N (sites) = 1.07 ± 4.8e-2
KD (M) = 1.60e-6 ± 535e-9
ΔH (kcal/mol) = -15.9 ± 1.40
Offset (kcal/mol) = -1.11 ± 0.378
ΔG (kcal/mol) = -7.91
-TΔS (kcal/mol) = 7.95
D
SDE2UBL into USP5H232A
250401UBLintoUSP5H232Arep1
Model: One Set of Sites
[Cell] (M) = 11.0e-6
[Syr] (M) = 211e-6
250401UBLintoUSP5H232Arep2
Model: One Set of Sites
[Cell] (M) = 11.0e-6
[Syr] (M) = 211e-6
E
SDE2UBL into USP5ΔZnF
LTH_251015 SDE2UBL into USP5 dZnF
Model: One Set of Sites
[Cell] (M) = 11.0e-6
[Syr] (M) = 208e-6
LTH_251015 SDE2UBL into USP5 dZnF
Model: One Set of Sites
[Cell] (M) = 11.0e-6
[Syr] (M) = 208e-6

Supplementary Figure 27 | Full ITC data from the titration of SDE2$_{UBL}$ into USP5 variants. SDE2$_{UBL}$ was titrated into USP5^{WT} (**A**), USP5^{C335A} (**B**), USP5^{C335R} (**C**), USP5^{H232A} (**D**) or USP5$^{\Delta ZnF}$ (**E**). Data was fit using a one-site model and the calculated values are presented.

SDE2 cleavage time course

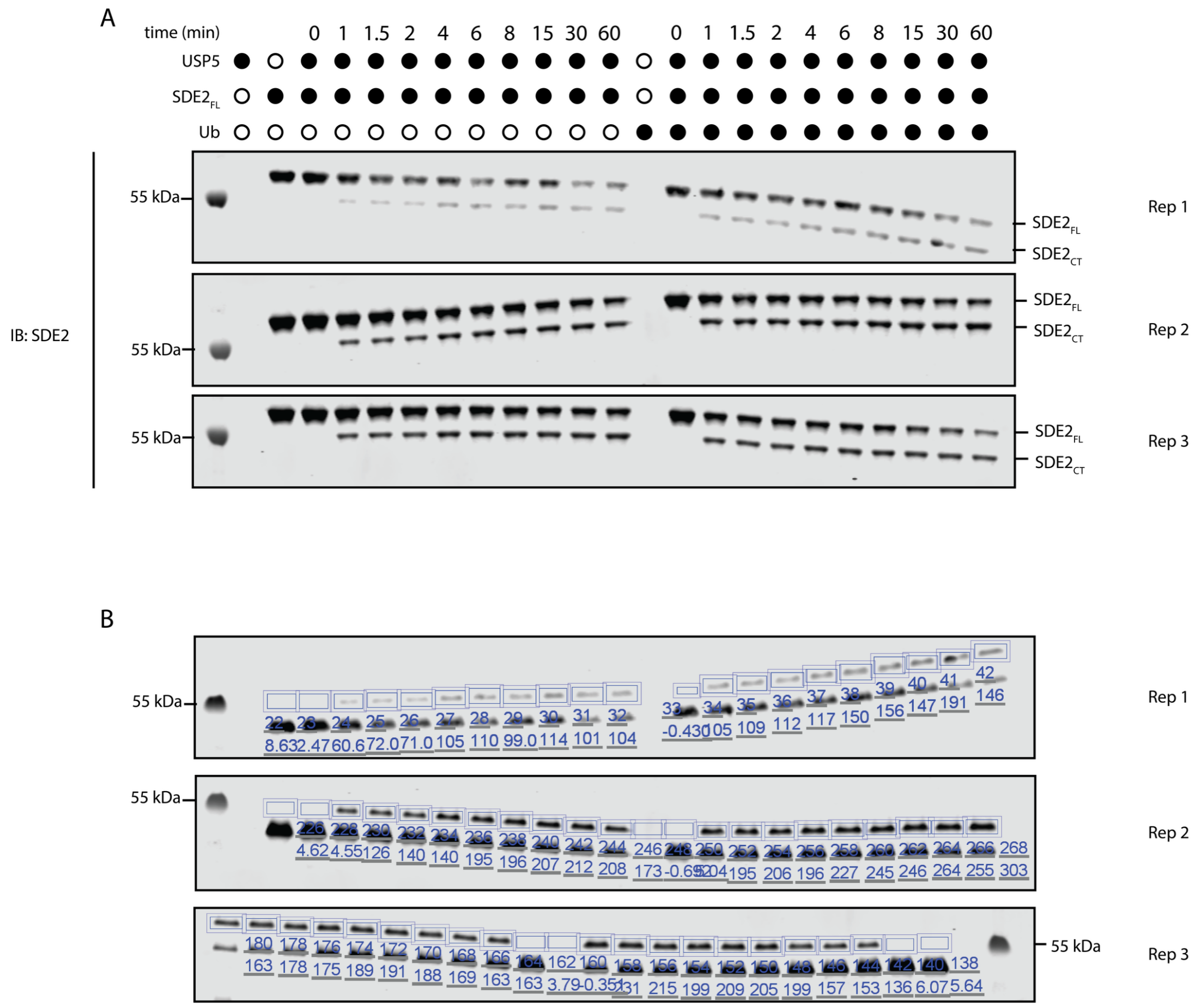

Supplementary Figure 28 | Immunoblots used to produce Supplementary Figure 16B showing cleavage of SDE2$_{FL}$ by USP5 in the absence or presence of Ub. (A) SDE2$_{FL}$ (3 µM) and USP5 (100 nM) were incubated at 25 °C, in the absence or presence of Ub (30 µM) Samples were collected at indicated time points, assessed by SDS-PAGE and immunoblotted. for quantification using ImageStudio. Reactions were performed in triplicate. **(B)** The bands corresponding to SDE2$_{CT}$ were quantified at each time-point using ImageStudio.

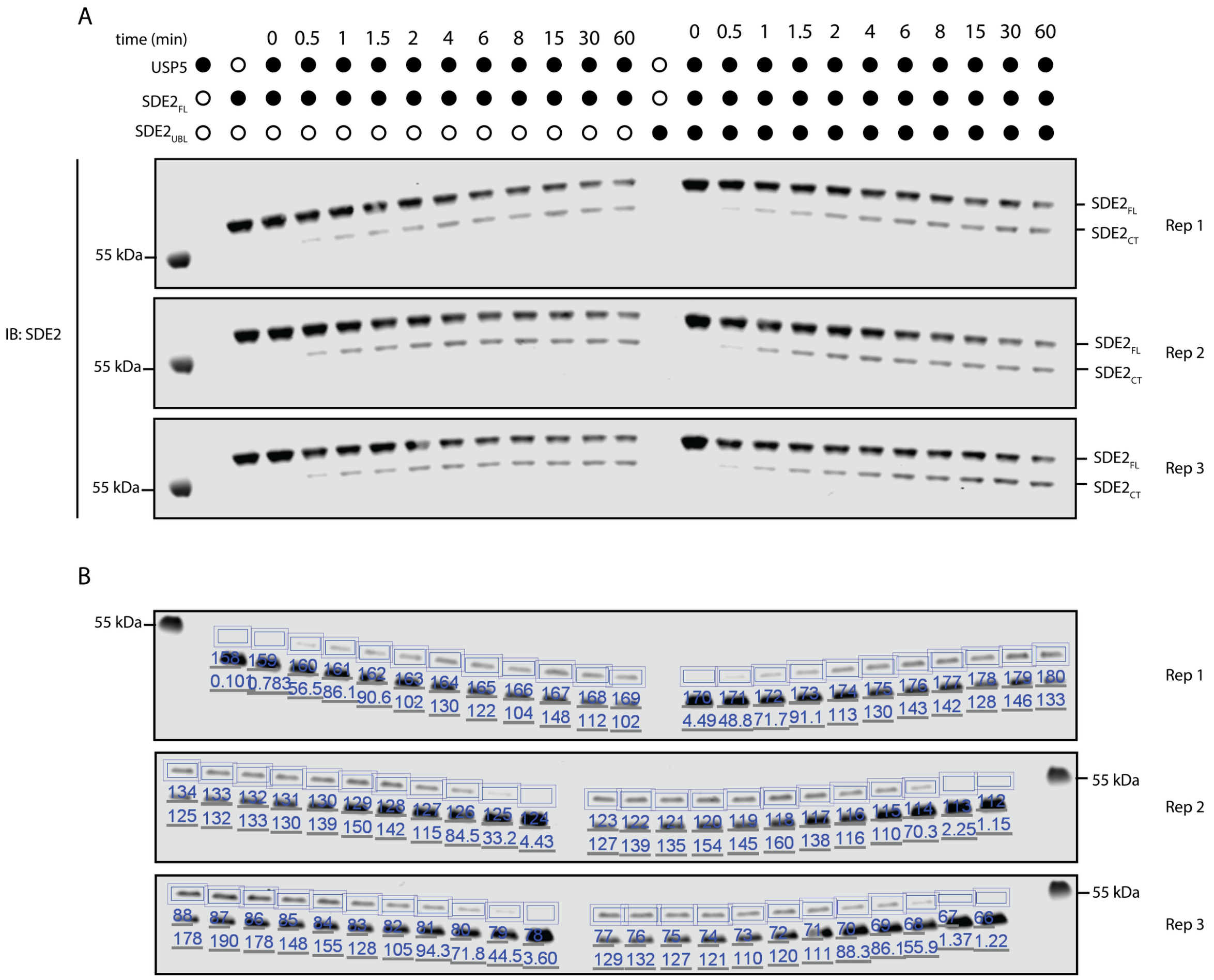

Supplementary Figure 29 | Immunoblots used to produce Supplementary Figure 16C showing cleavage of SDE2$_{FL}$ by USP5 in the absence or presence of SDE2$_{UBL}$. (A) SDE2$_{FL}$ (3 µM) and USP5 (100 nM) were incubated at 25 °C, in the absence or presence of SDE2$_{UBL}$ (30 µM) Samples were collected at indicated time points, assessed by SDS-PAGE and immunoblotted. for quantification using ImageStudio. Reactions were performed in triplicate. **(B)** The bands corresponding to SDE2$_{CT}$ were quantified at each time-point using ImageStudio.

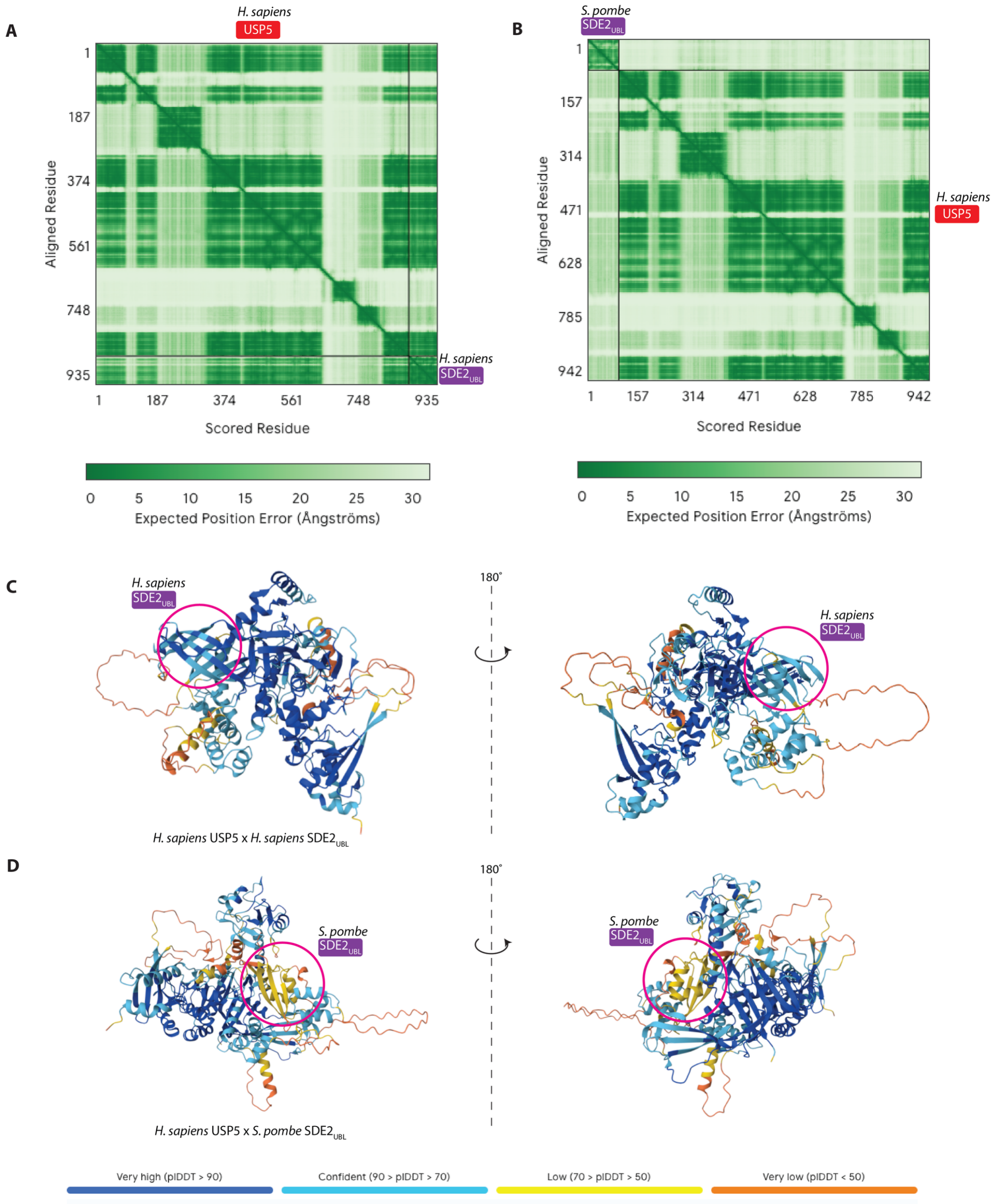

Supplementary Figure 30 | SDE2$_{UBL}$ AF3 models in USP5. Predicted alignment error plots for **(A)** *Homo sapiens* and **(B)** *Schizosaccharomyces pombe* SDE2$_{UBL}$ bound in human USP5. pLDDT score overlaid from two viewing angles for **(C)** *H. sapiens* and for **(D)** *S. pombe*.

P45974 10-31: LSVLPTIRVPKAGDRVHKDECA (#1)

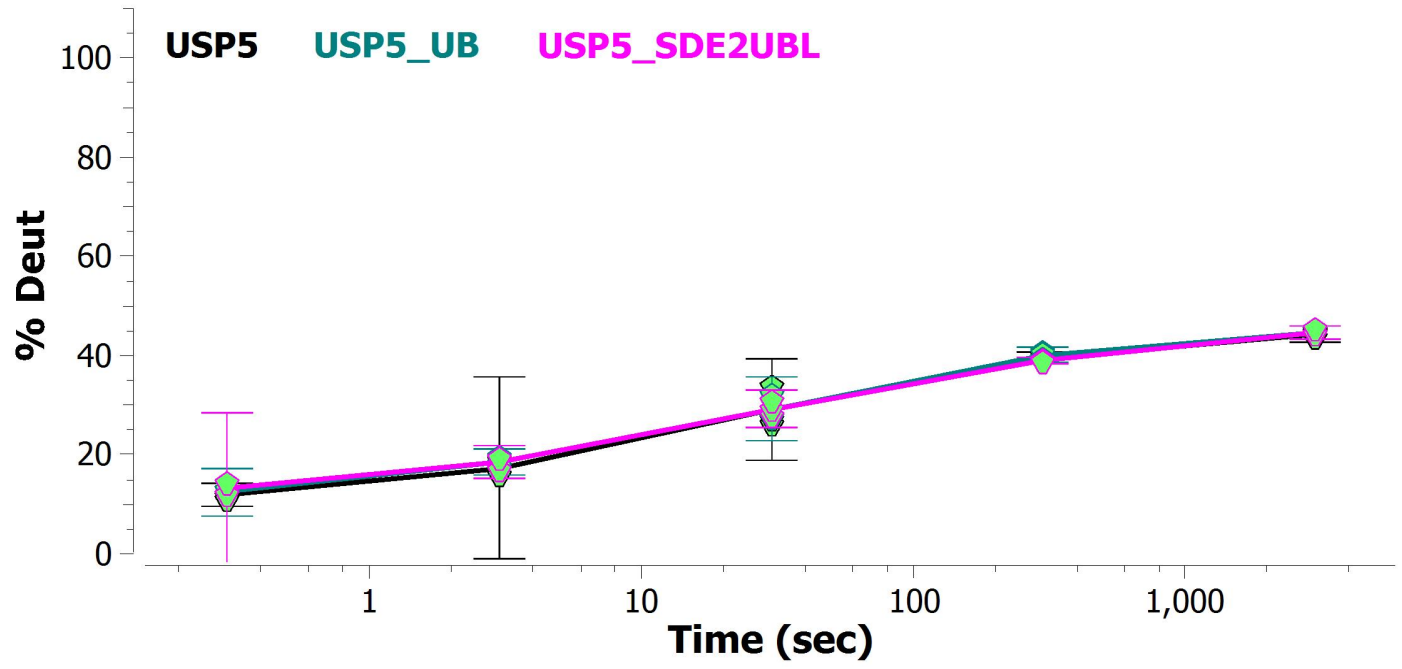

P45974 10-32: LSVLPTIRVPKAGDRVHKDECAF (#2)

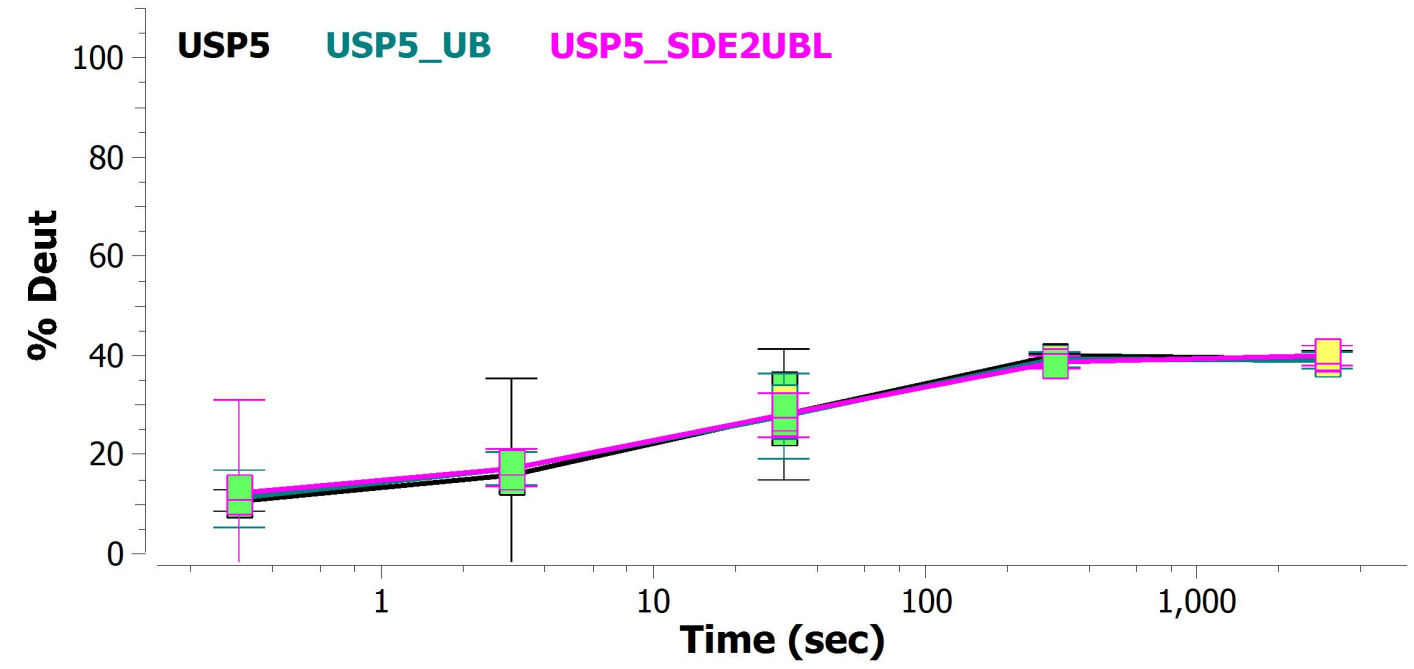

P45974 13-31: LPTIRVPKAGDRVHKDECA (#3)

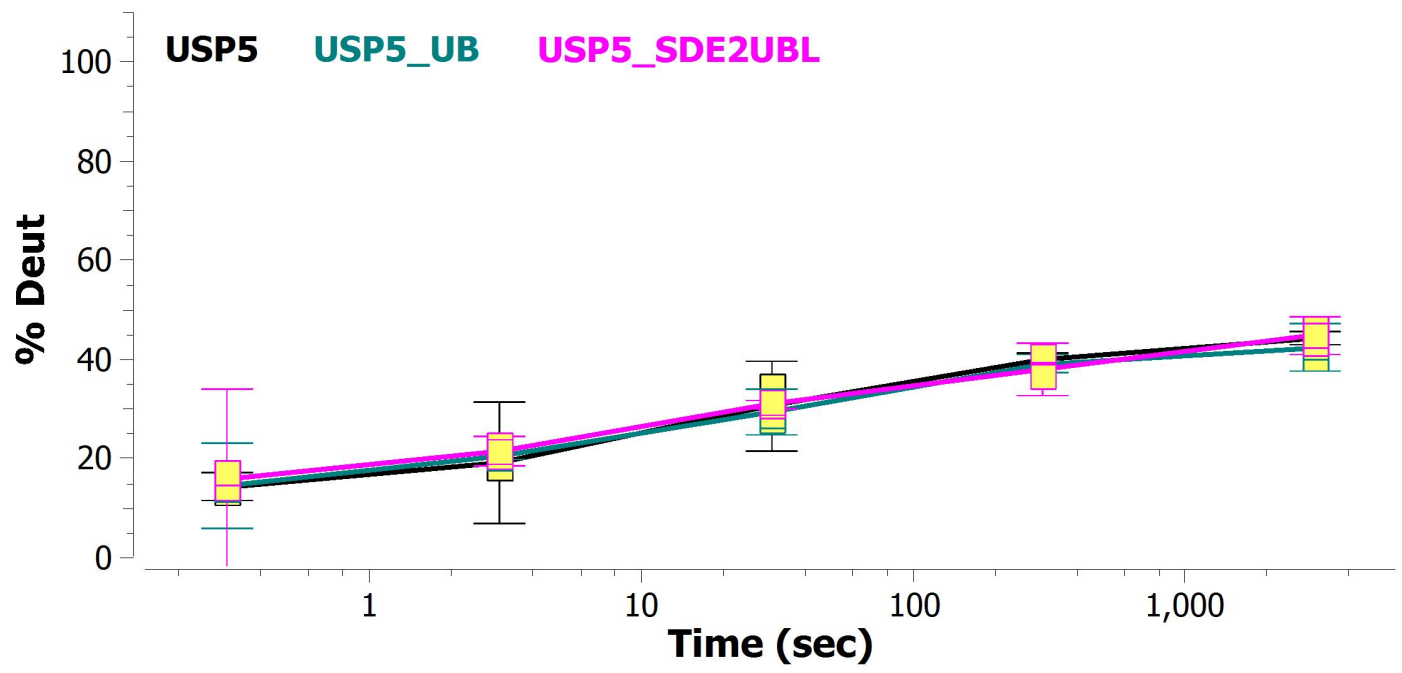

P45974 14-31: PTIRVPKAGDRVHKDECA (#4)

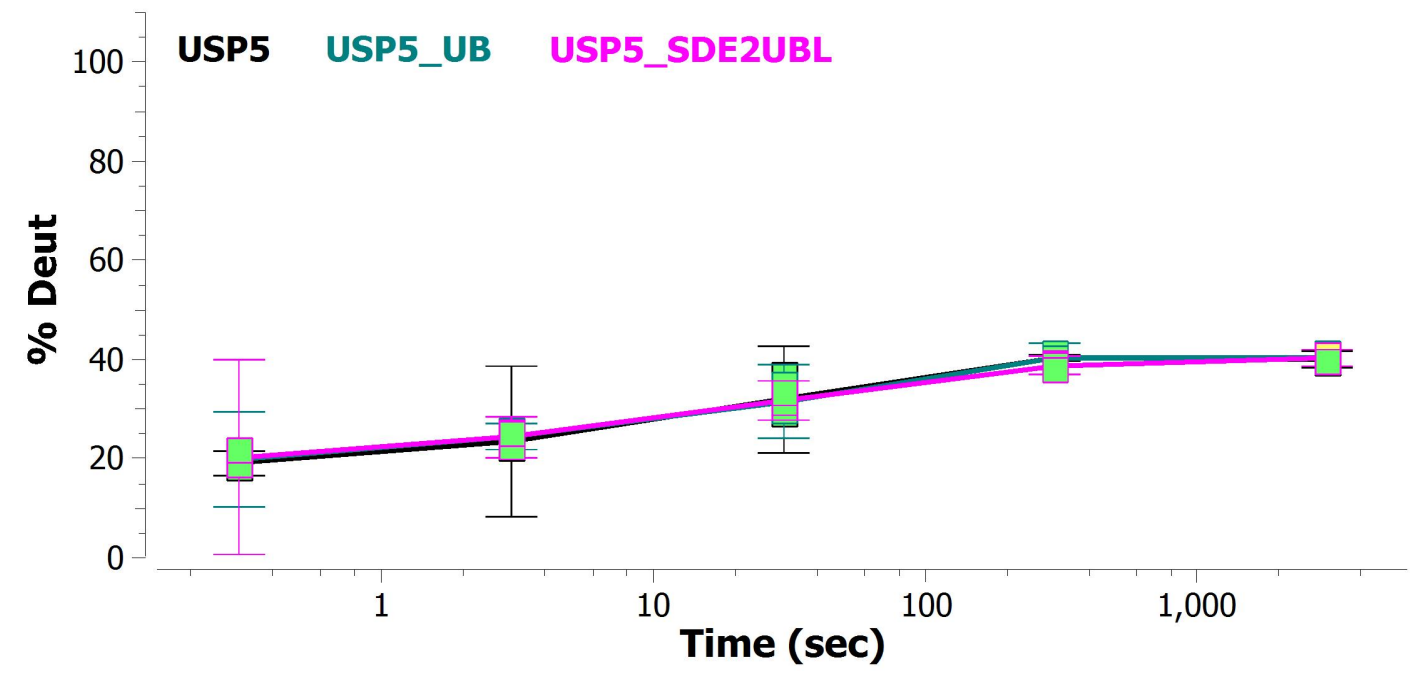

P45974 14-32: PTIRVPKAGDRVHKDECAF (#5)

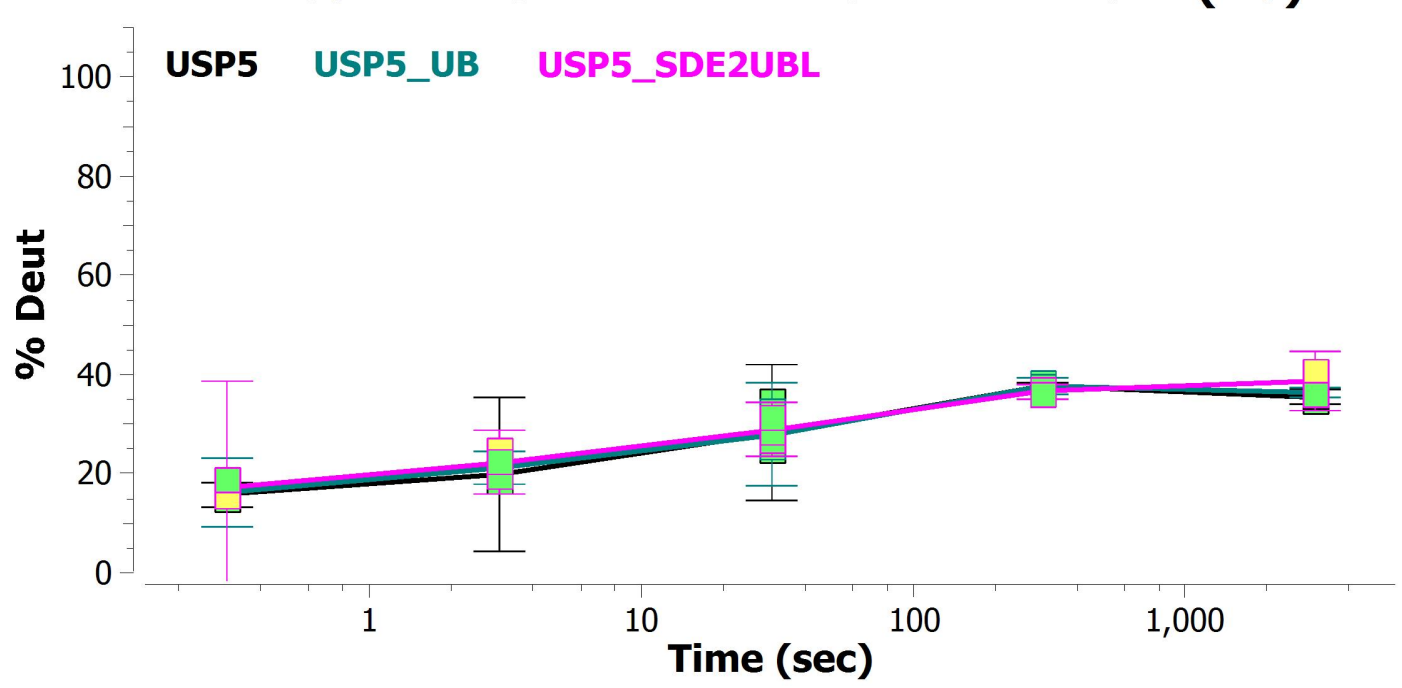

P45974 31-43: AFSFDTPESEGGL (#6)

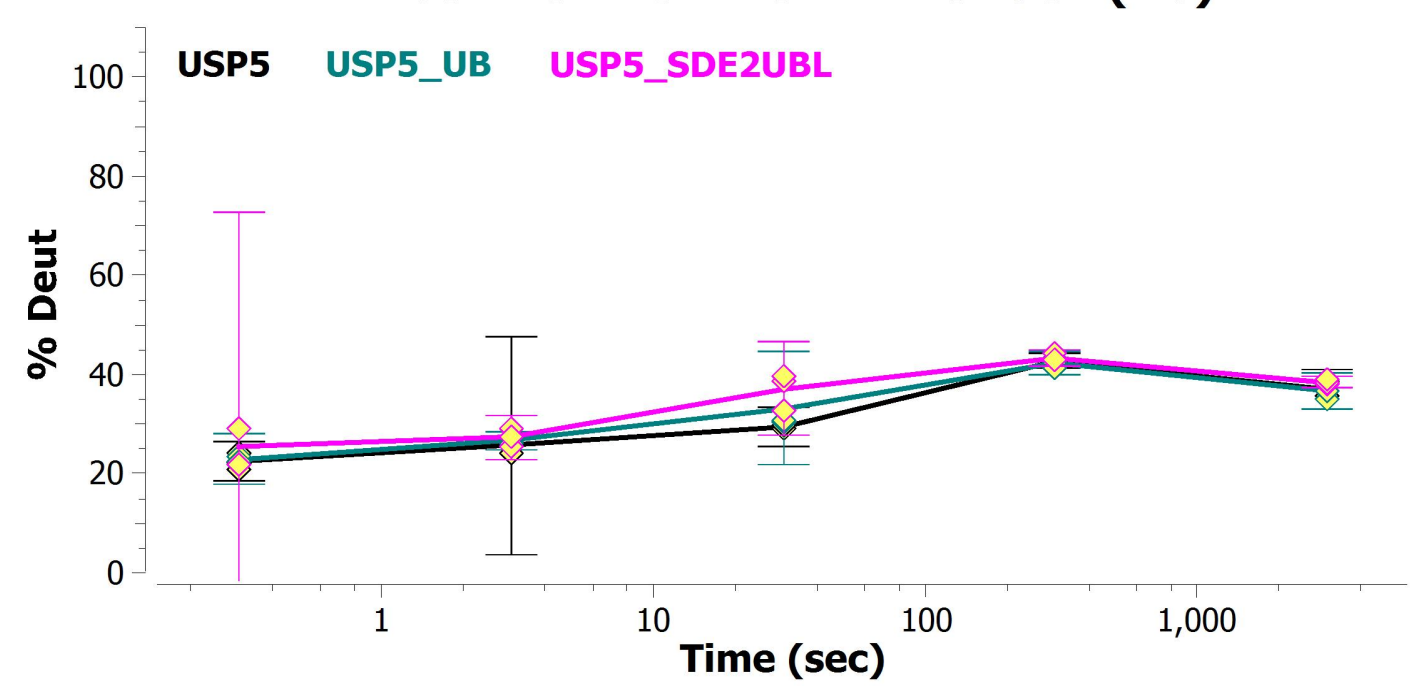

P45974 32-43: FSFDTPESEGGL (#7)

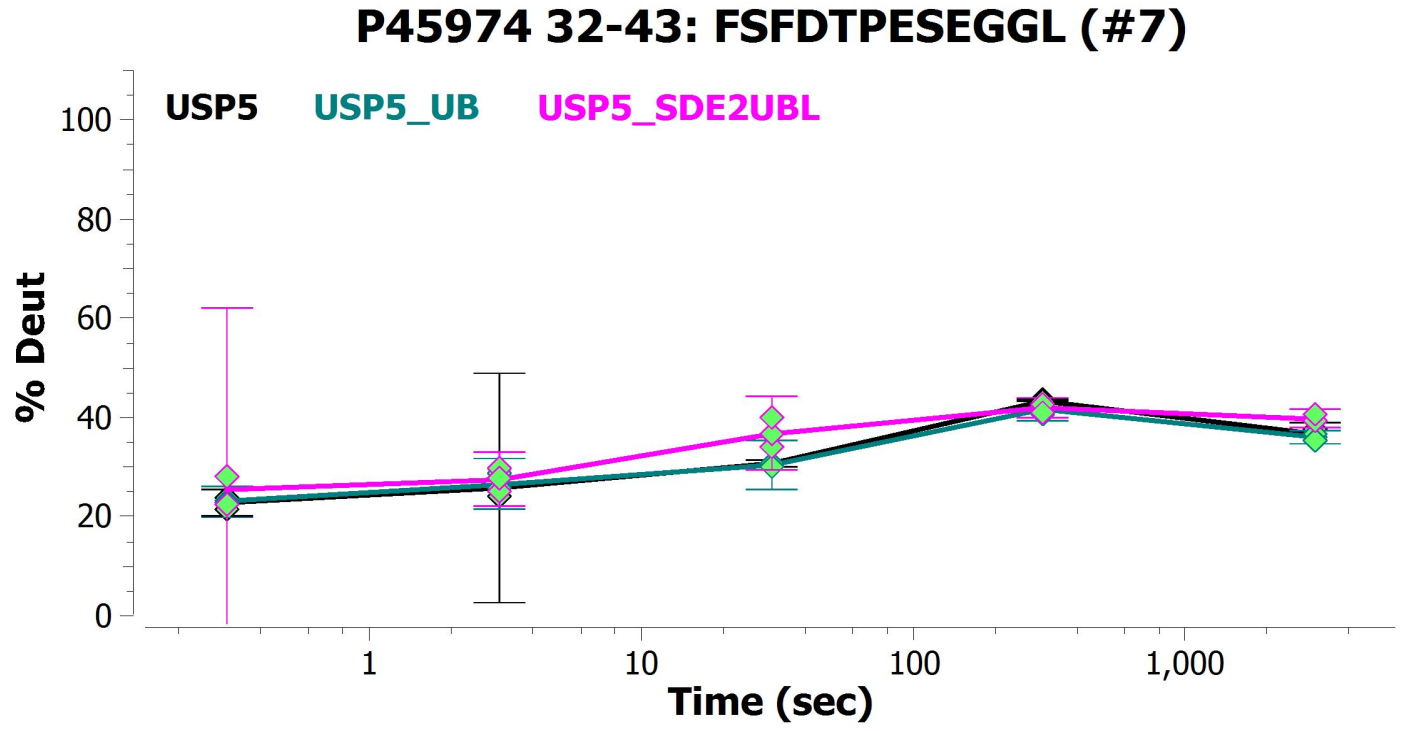

P45974 33-44: SFDTPESEGGLY (#8)

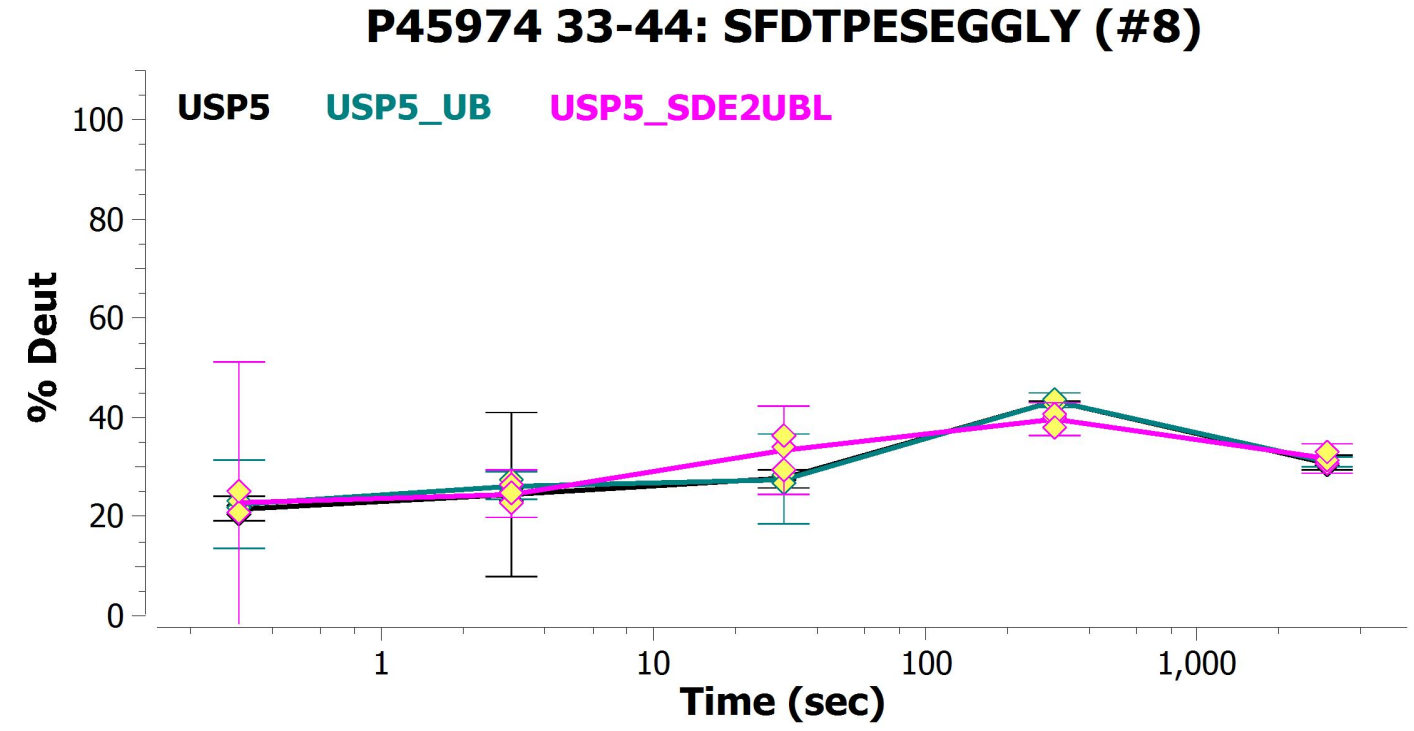

P45974 48-71: NTFLGFGKQYVERHFNKTGQRVYL (#9)

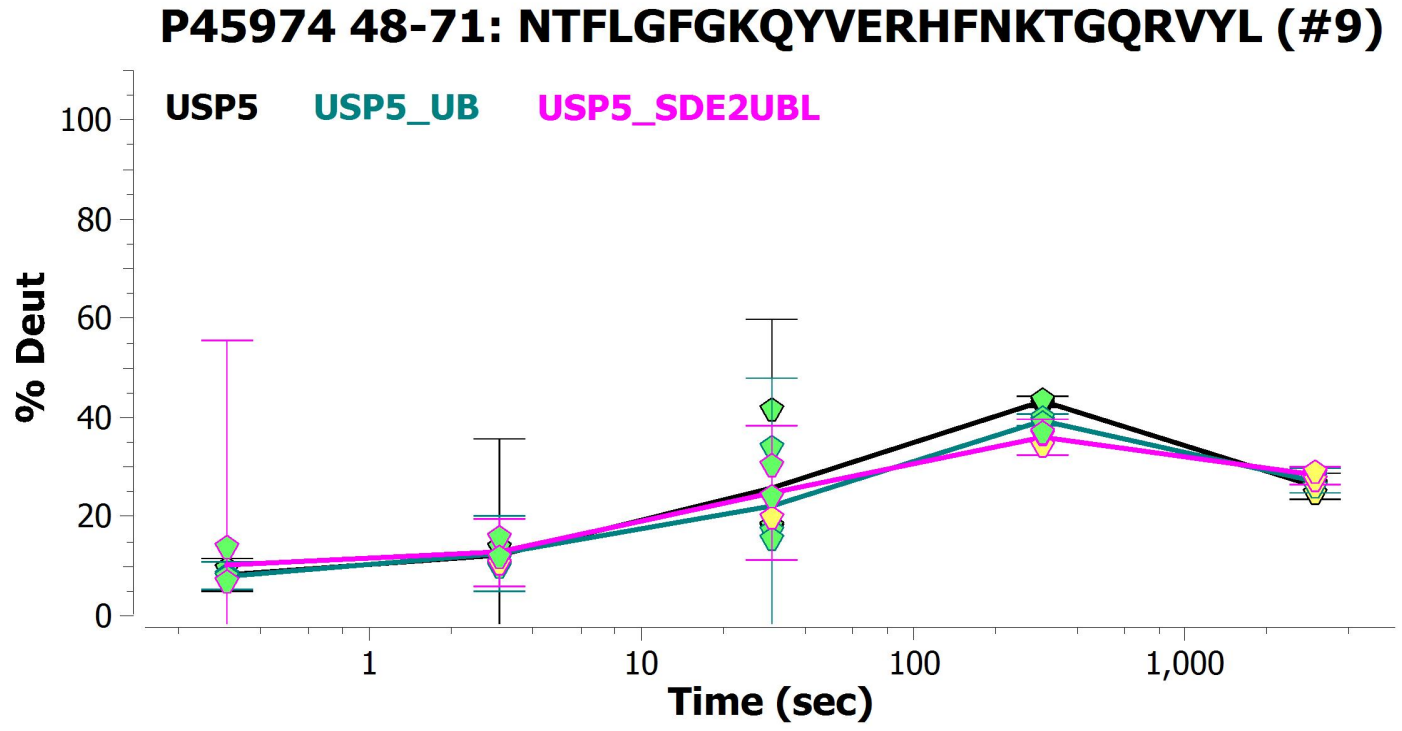

P45974 48-73: NTFLGFGKQYVERHFNKTGQRVYLHL (#10)

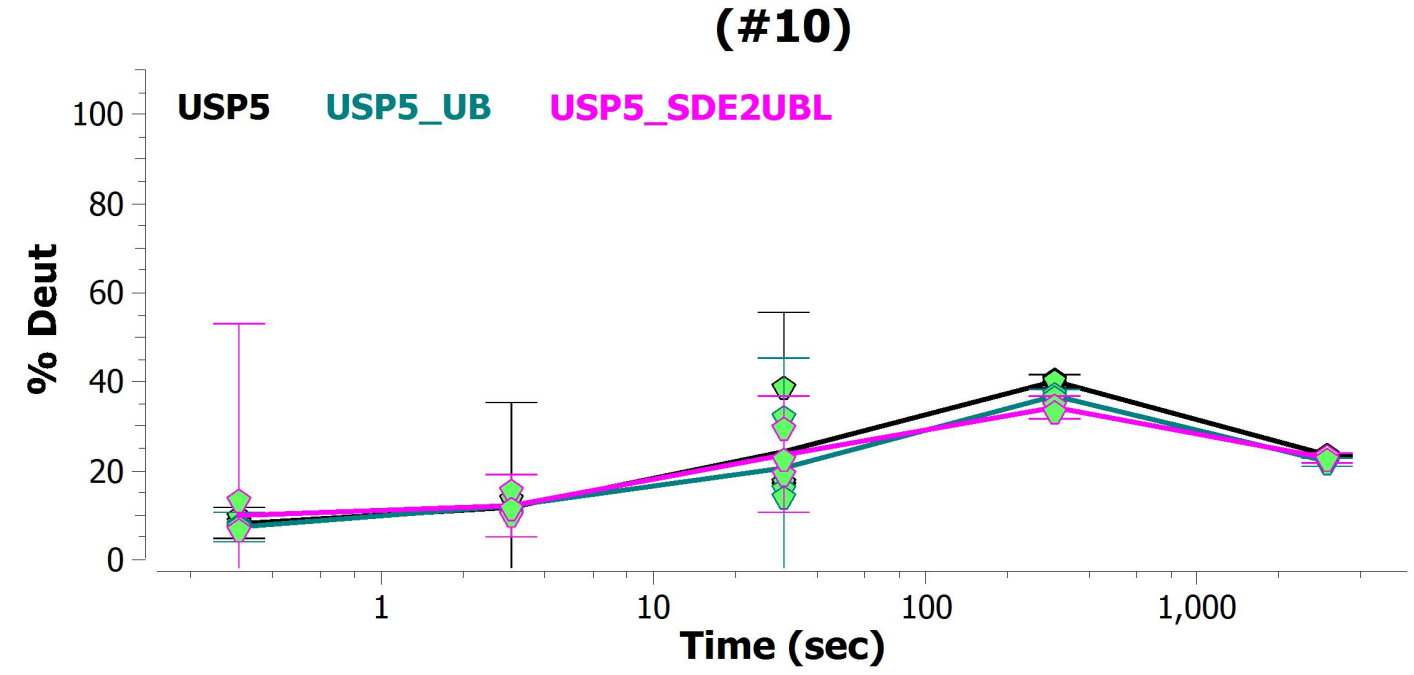

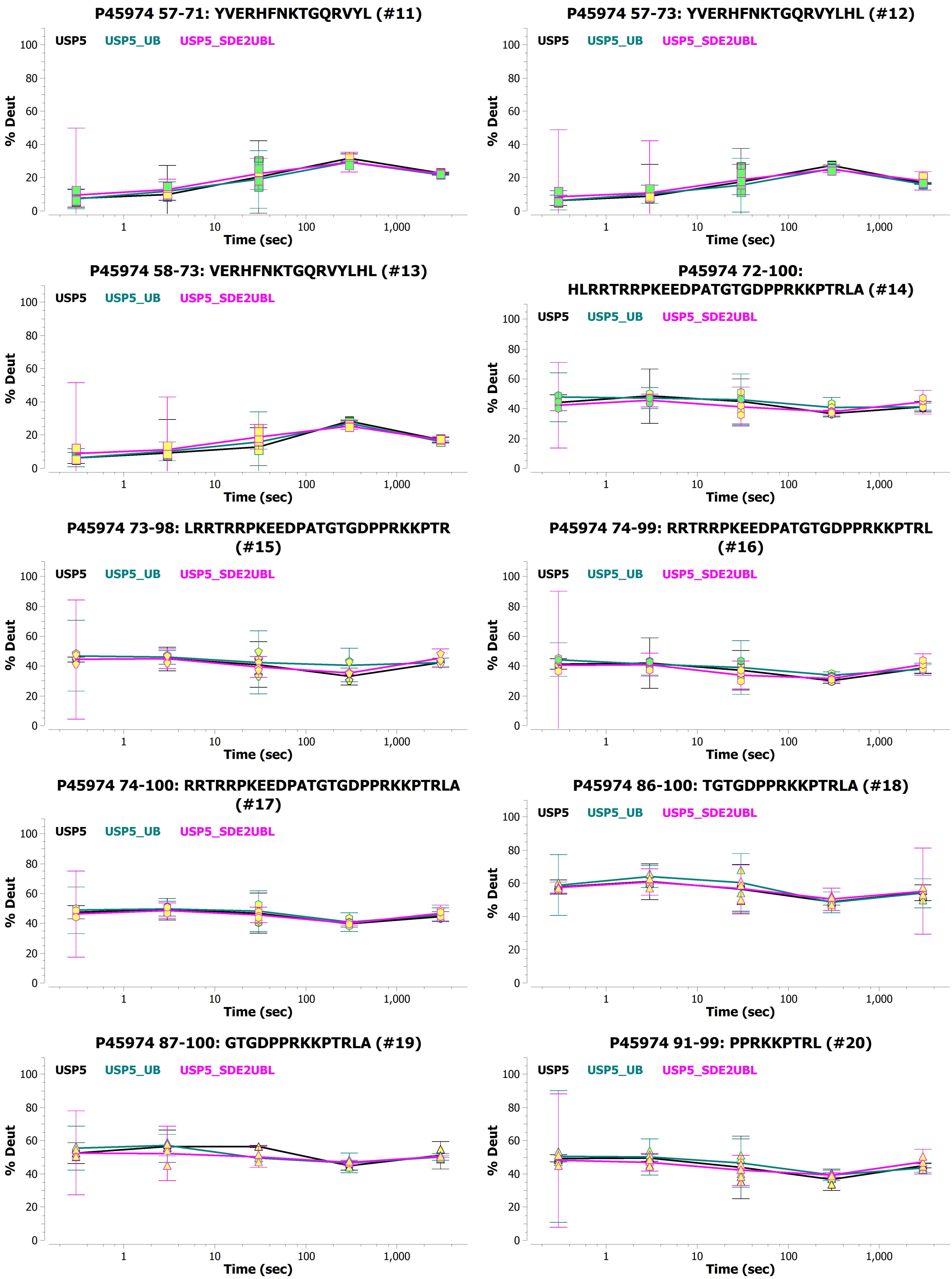
P45974 57-71: YVERHFNKTGQRVYL (#11)
USP5 USP5_UB USP5_SDE2UBL
% Deut
Time (sec)
P45974 57-73: YVERHFNKTGQRVYLHL (#12)
USP5 USP5_UB USP5_SDE2UBL
% Deut
Time (sec)
P45974 58-73: VERHFNKTGQRVYLHL (#13)
USP5 USP5_UB USP5_SDE2UBL
% Deut
Time (sec)
P45974 72-100: HLRRTRRPKEEDPATGTGDPPRKKPTRLA (#14)
USP5 USP5_UB USP5_SDE2UBL
% Deut
Time (sec)
P45974 73-98: LRRTRRPKEEDPATGTGDPPRKKPTR (#15)
USP5 USP5_UB USP5_SDE2UBL
% Deut
Time (sec)
P45974 74-99: RRTRRPKEEDPATGTGDPPRKKPTRL (#16)
USP5 USP5_UB USP5_SDE2UBL
% Deut
Time (sec)
P45974 74-100: RRTRRPKEEDPATGTGDPPRKKPTRLA (#17)
USP5 USP5_UB USP5_SDE2UBL
% Deut
Time (sec)
P45974 86-100: TGTGDPPRKKPTRLA (#18)
USP5 USP5_UB USP5_SDE2UBL
% Deut
Time (sec)
P45974 87-100: GTGDPPRKKPTRLA (#19)
USP5 USP5_UB USP5_SDE2UBL
% Deut
Time (sec)
P45974 91-99: PPRKKPTRL (#20)
USP5 USP5_UB USP5_SDE2UBL
% Deut
Time (sec)

P45974 91-100: PPRKKPTRLA (#21)

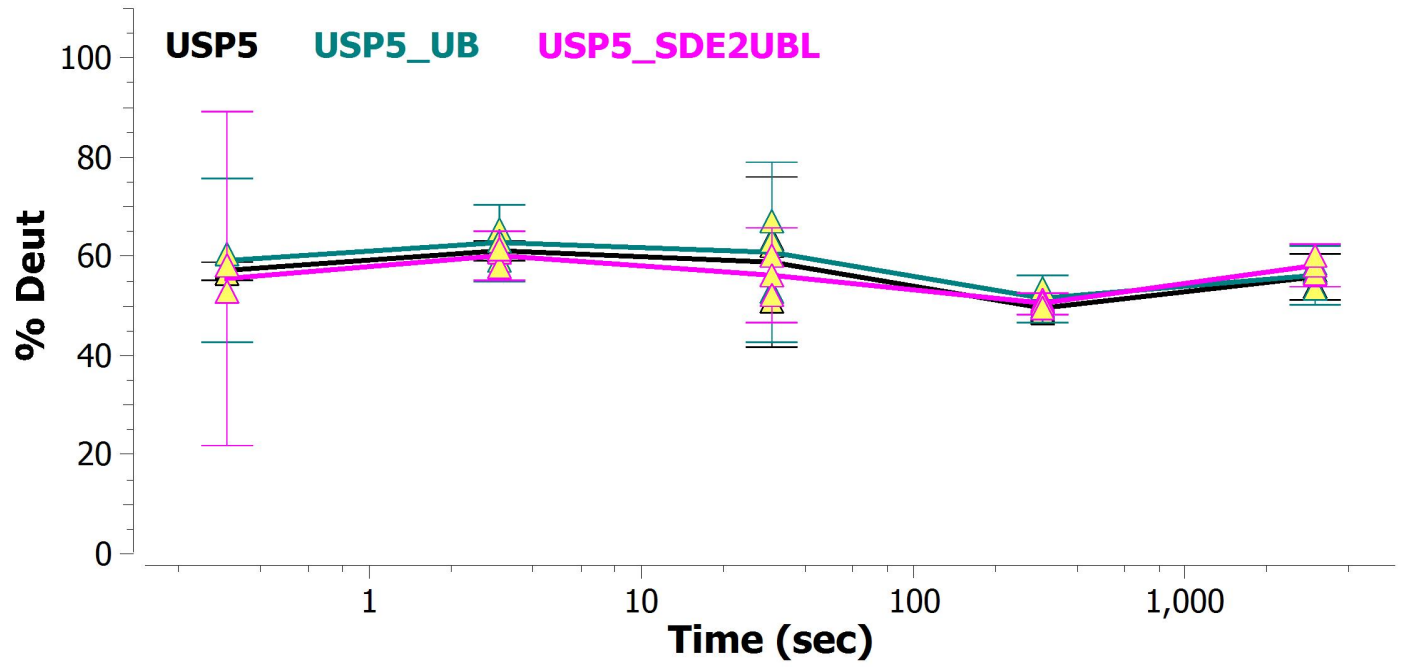

P45974 108-116: DLSEEKFEL (#22)

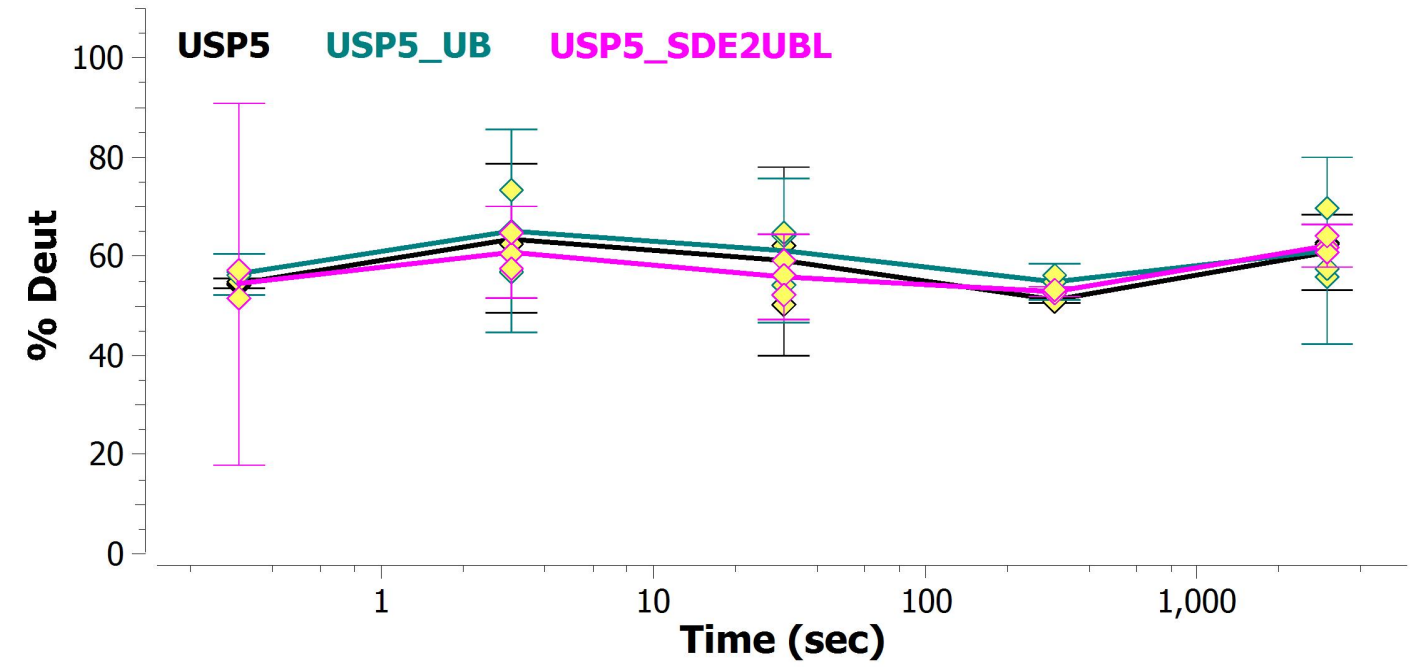

P45974 119-128: DVKIVILPDY (#23)

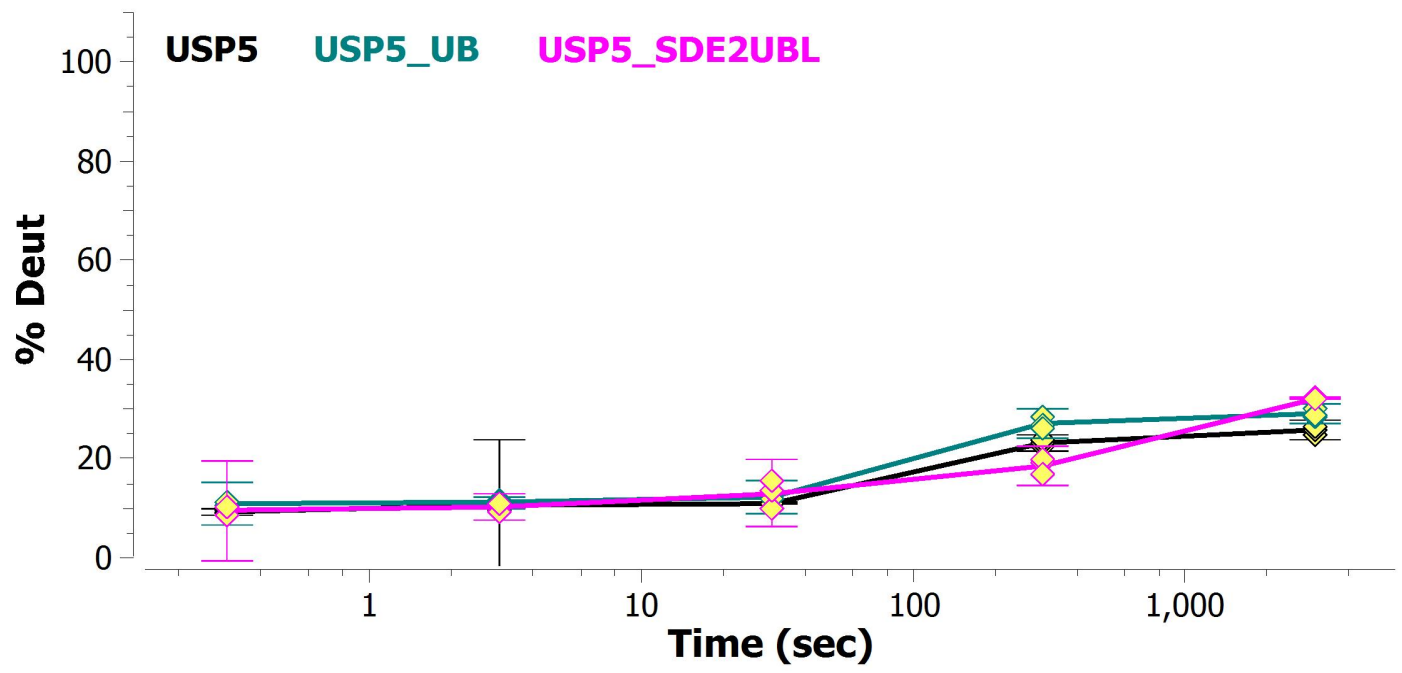

P45974 119-129: DVKIVILPDYL (#24)

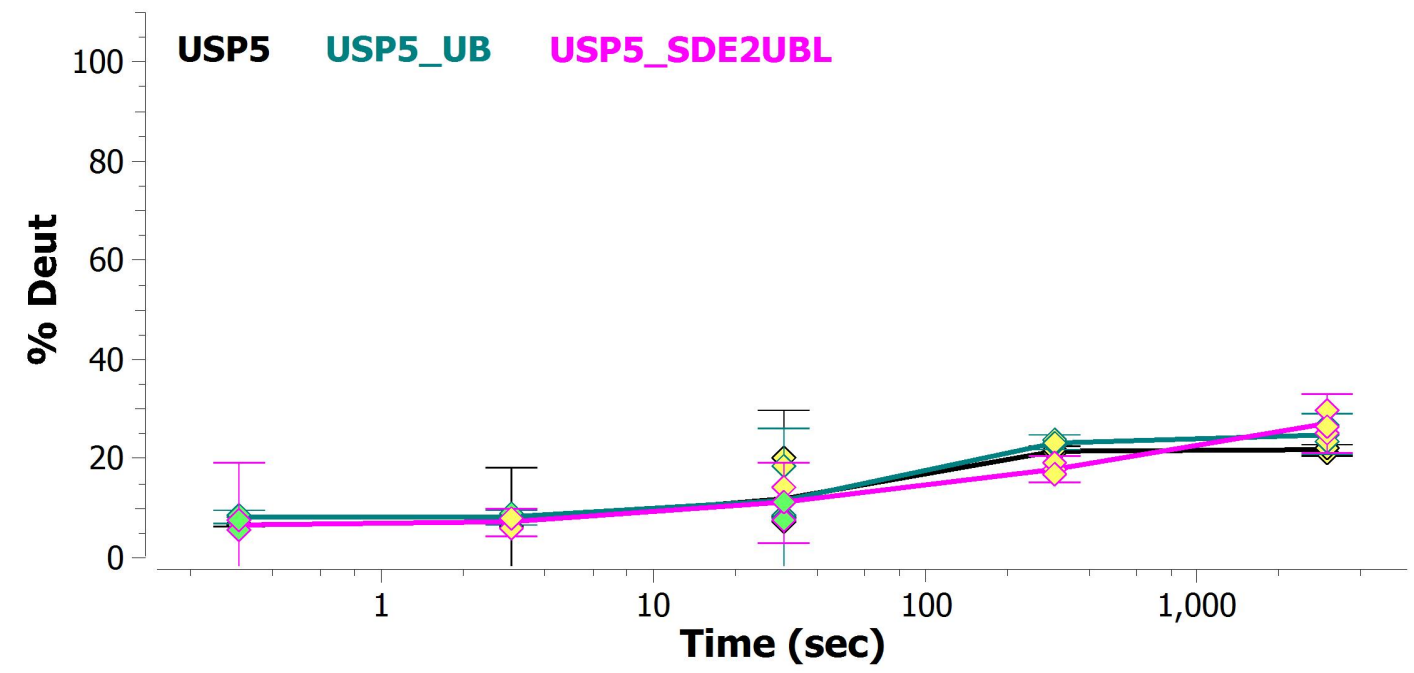

P45974 119-130: DVKIVILPDYLE (#25)

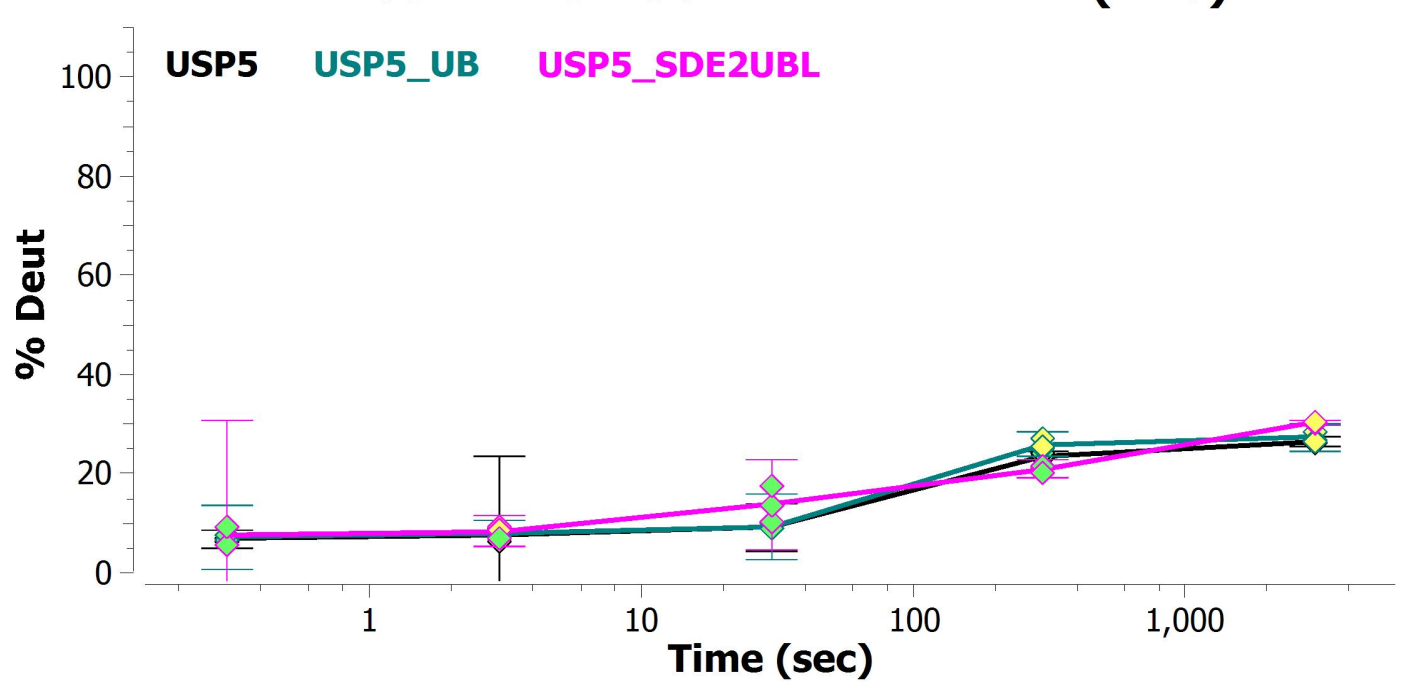

P45974 120-129: VKIVILPDYL (#26)

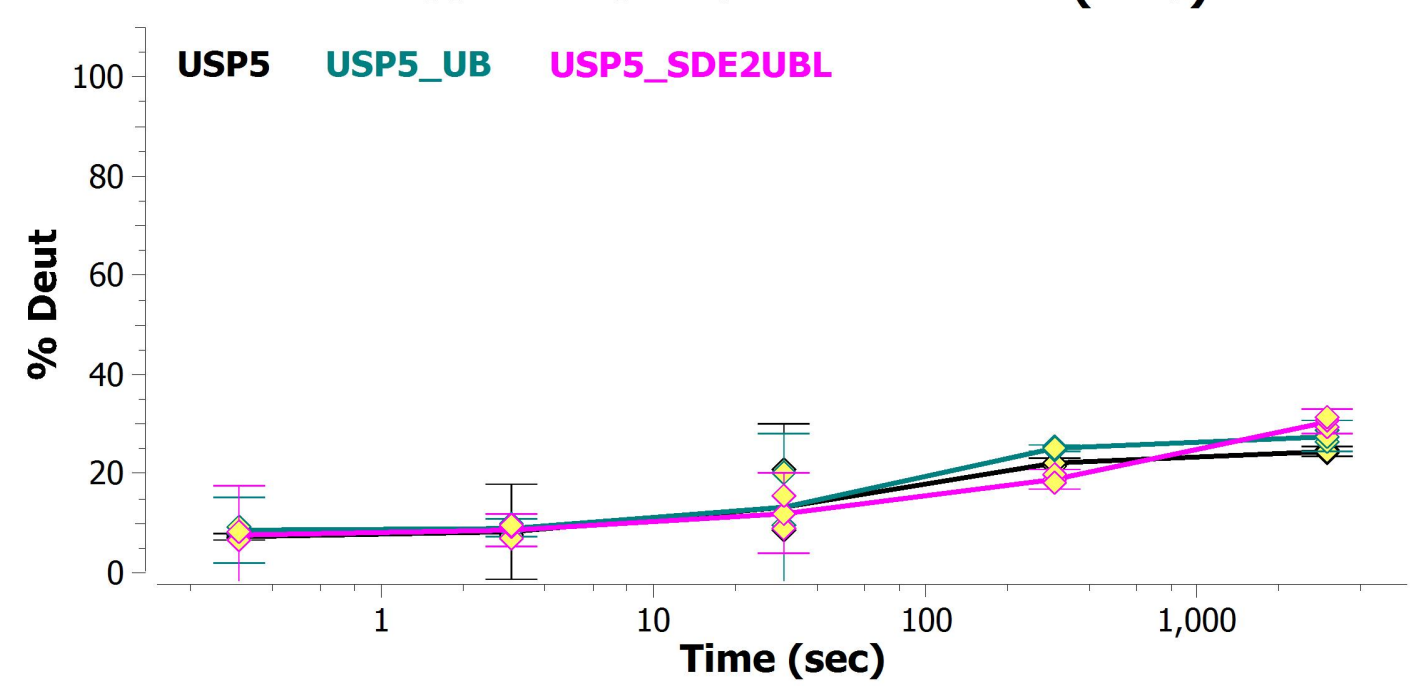

P45974 120-130: VKIVILPDYLE (#27)

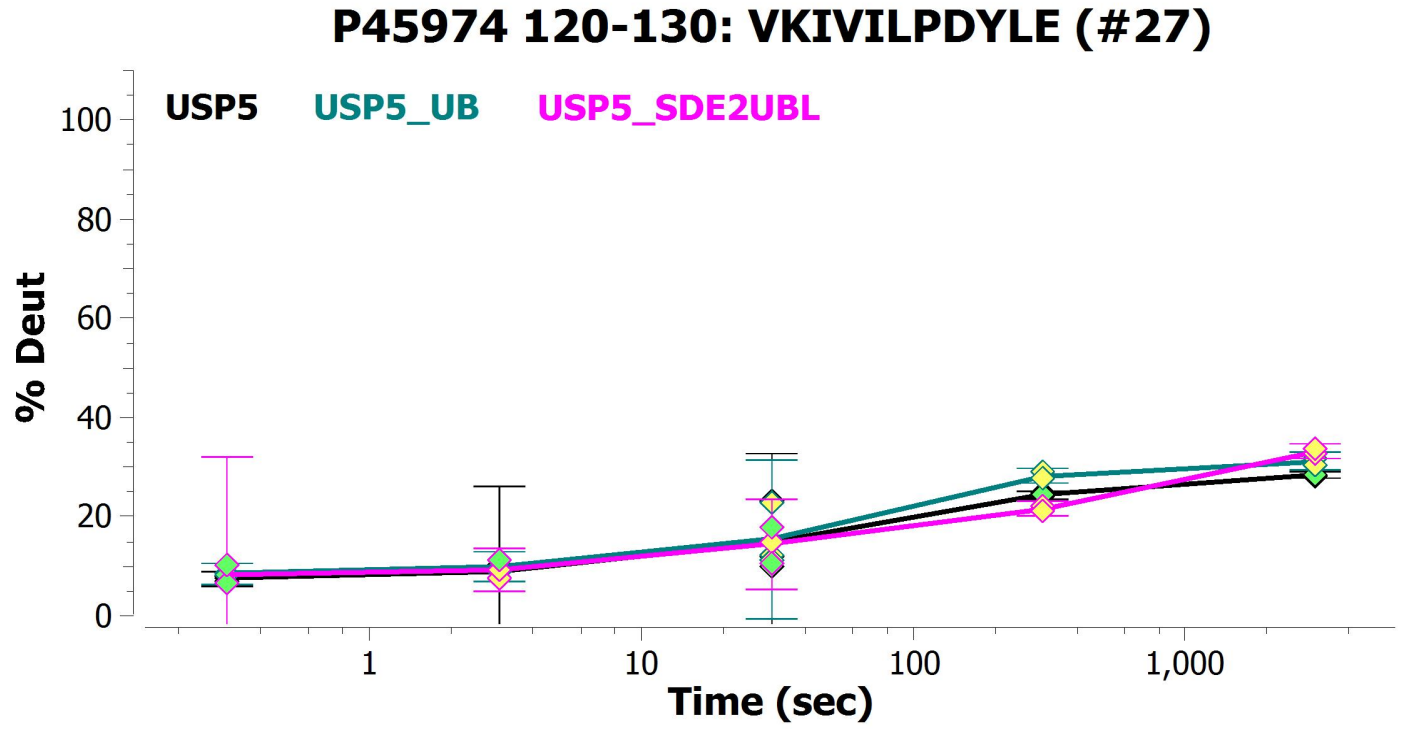

P45974 121-130: KIVILPDYLE (#28)

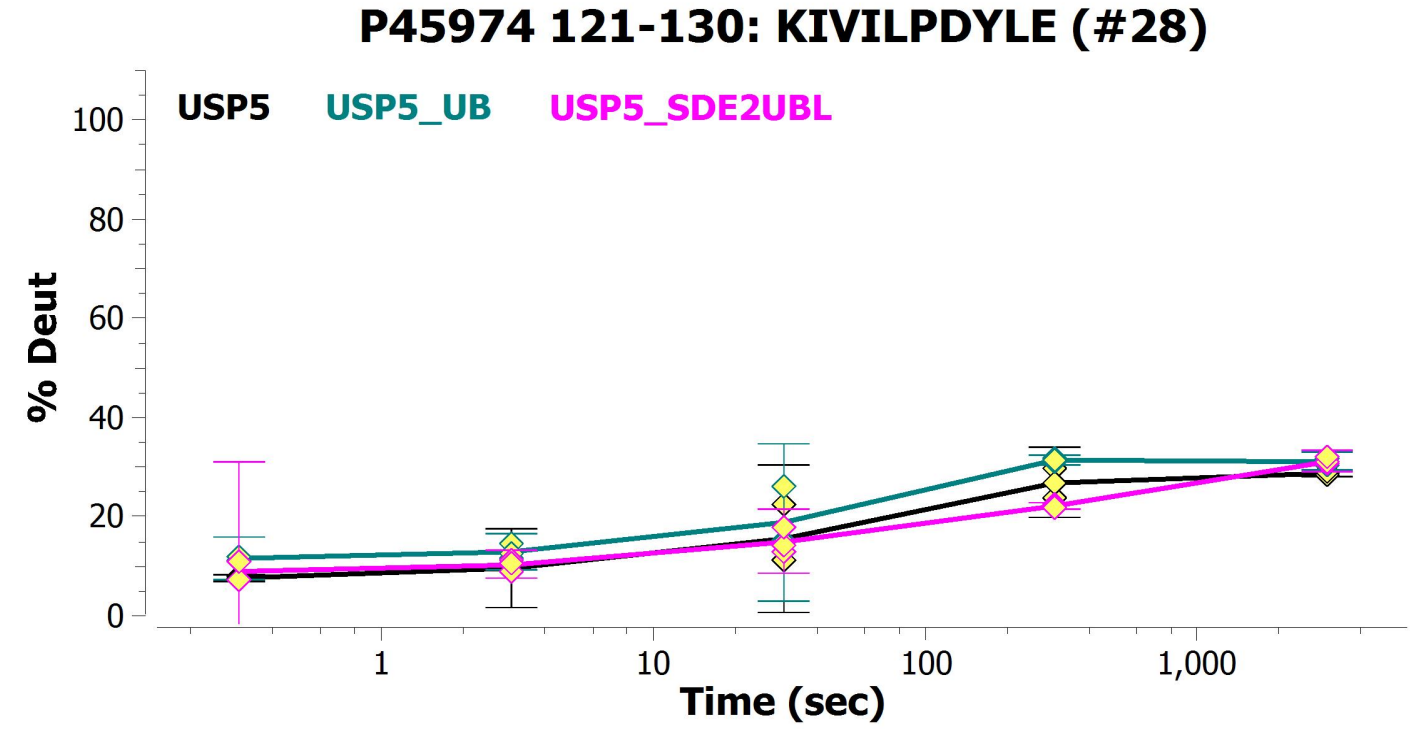

P45974 122-128: IVILPDY (#29)

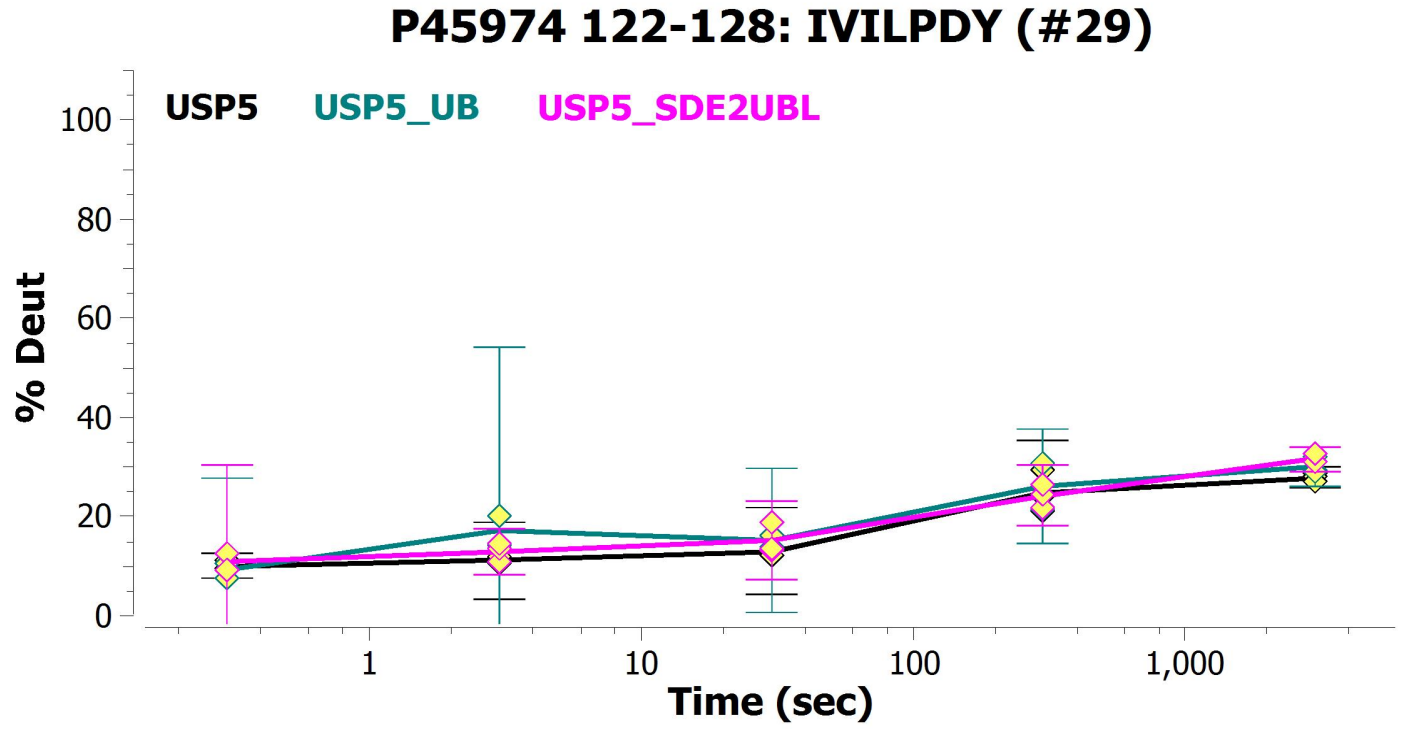

P45974 122-129: IVILPDYL (#30)

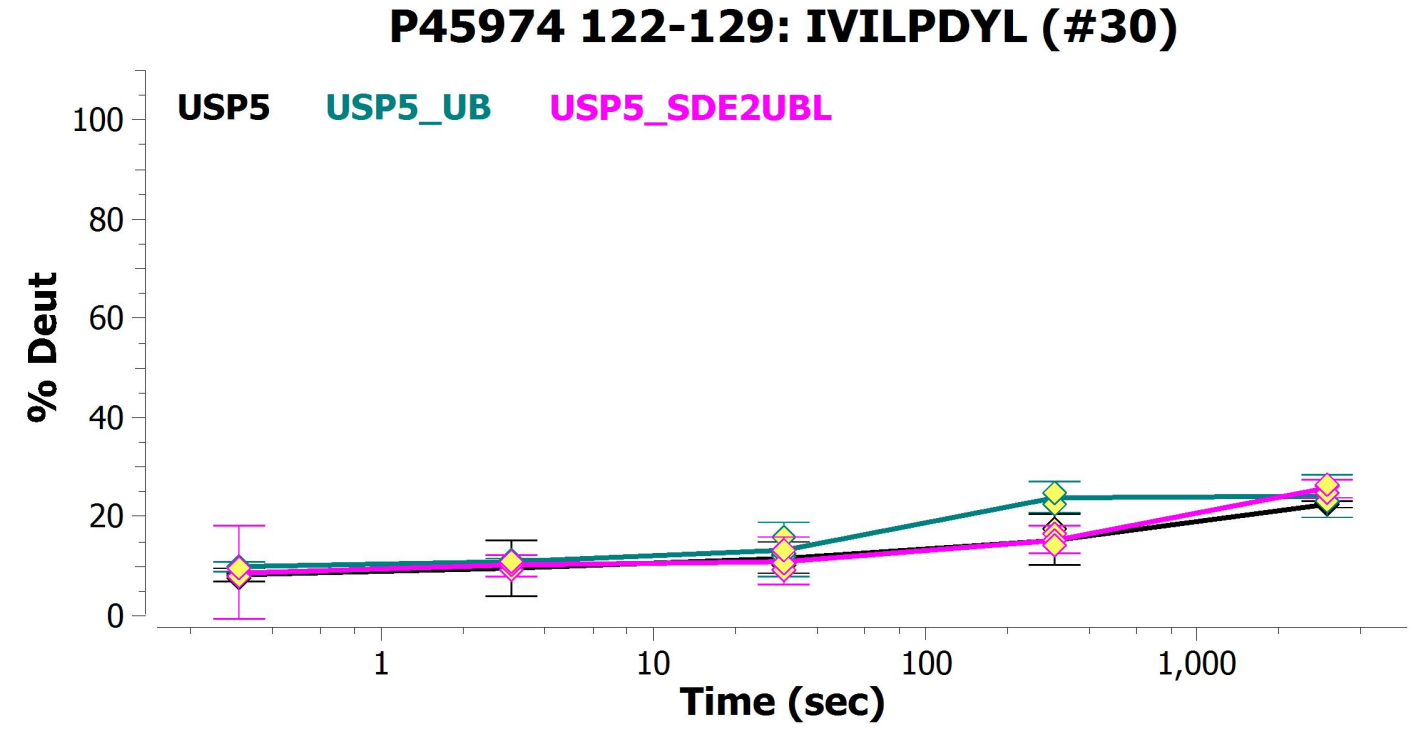

P45974 129-152: LEIARDGLGGLPDIVRDRVTSAVE (#31)

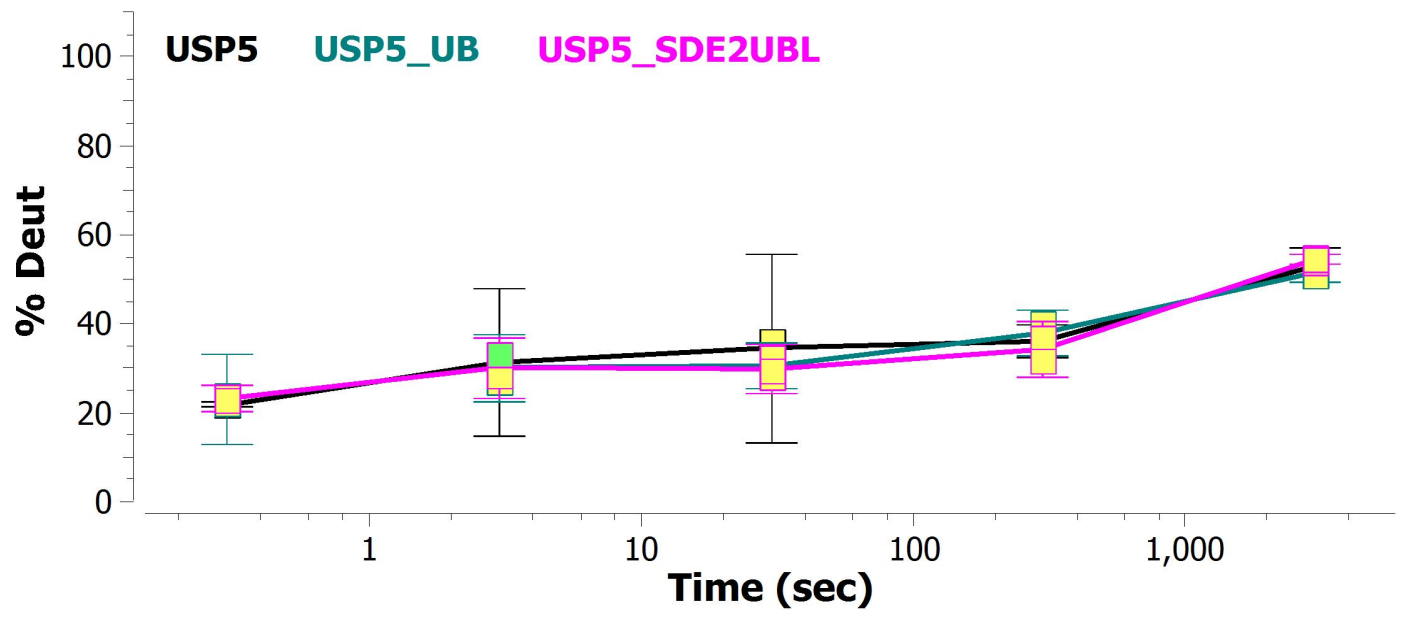

P45974 130-145: EIARDGLGGLPDIVRD (#32)

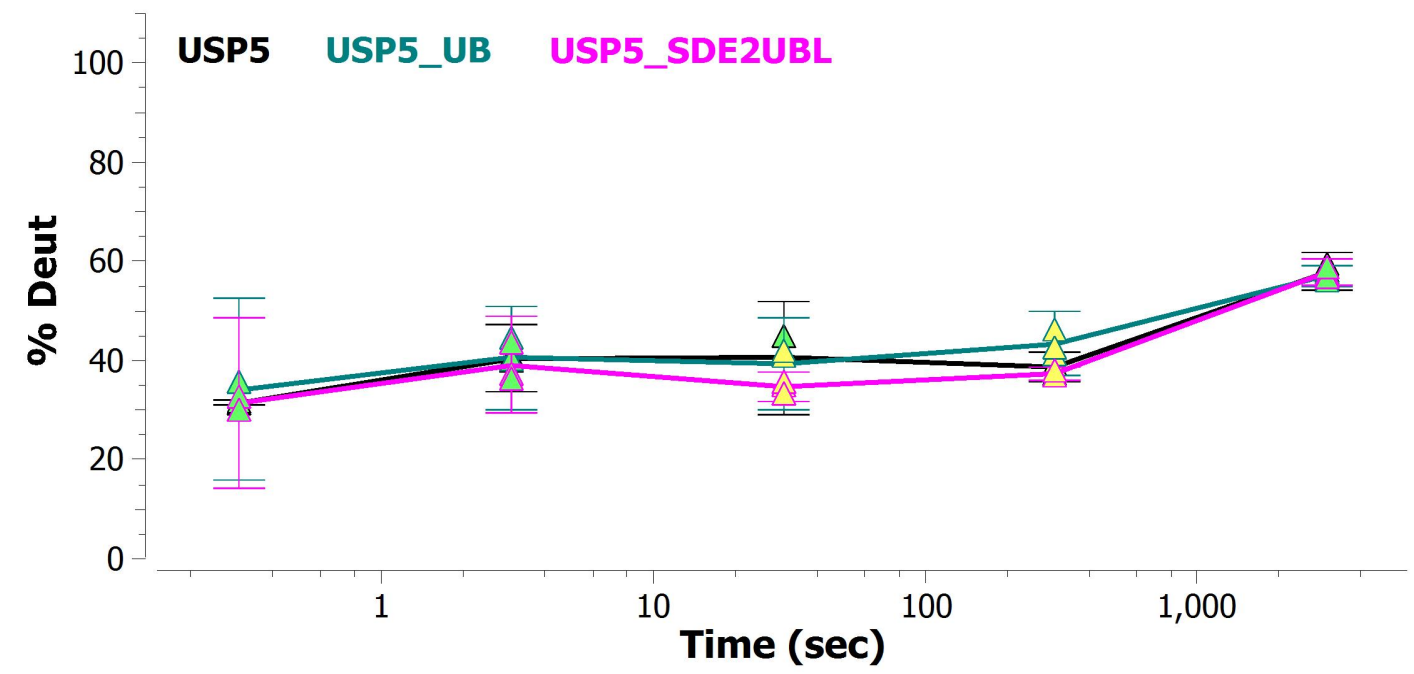

P45974 130-149: EIARDGLGGLPDIVRDRVTS (#33)

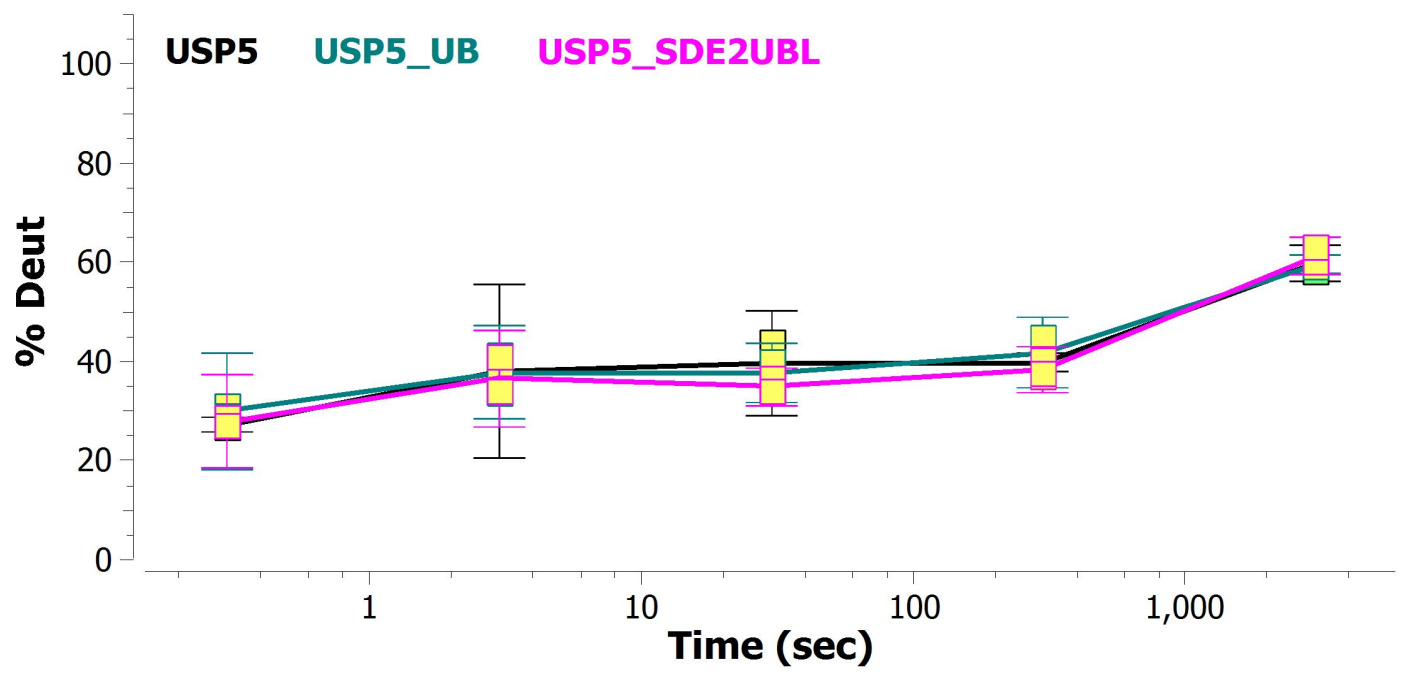

P45974 130-150: EIARDGLGGLPDIVRDRVTSA (#34)

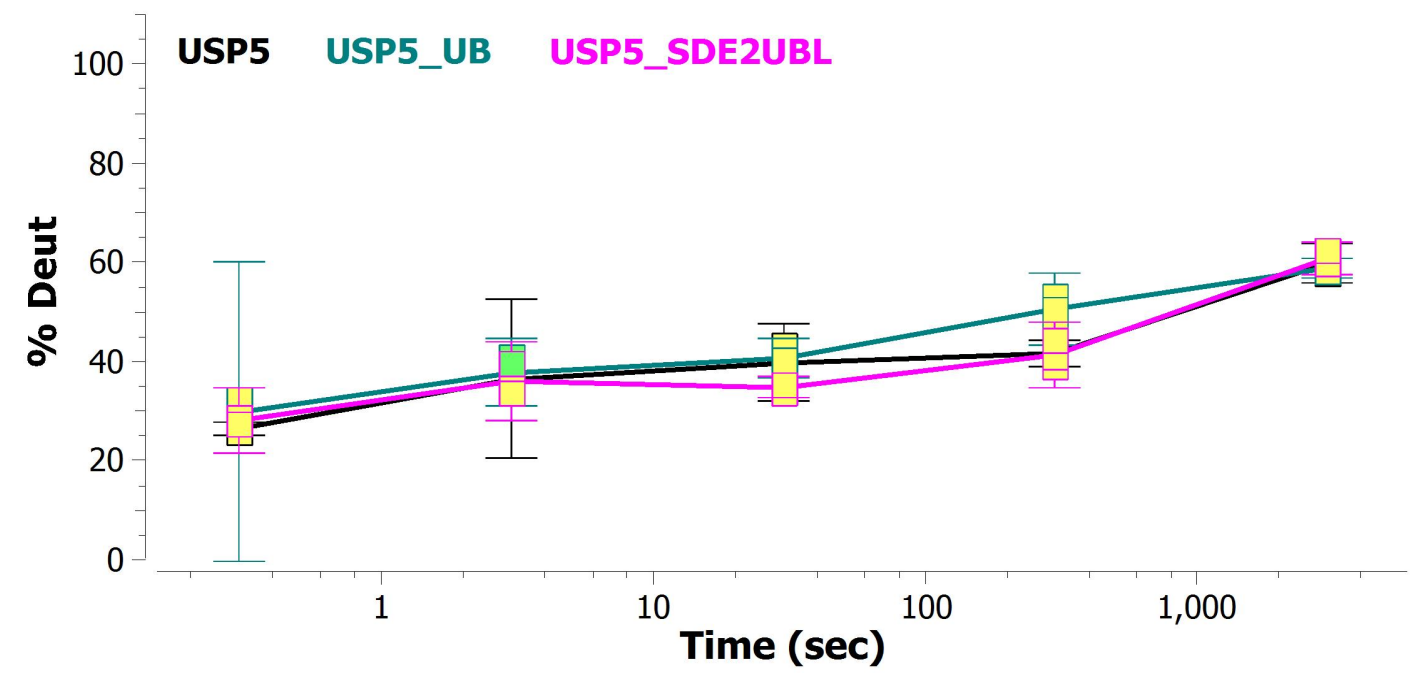

P45974 130-151: EIARDGLGGLPDIVRDRVTSAV (#35)

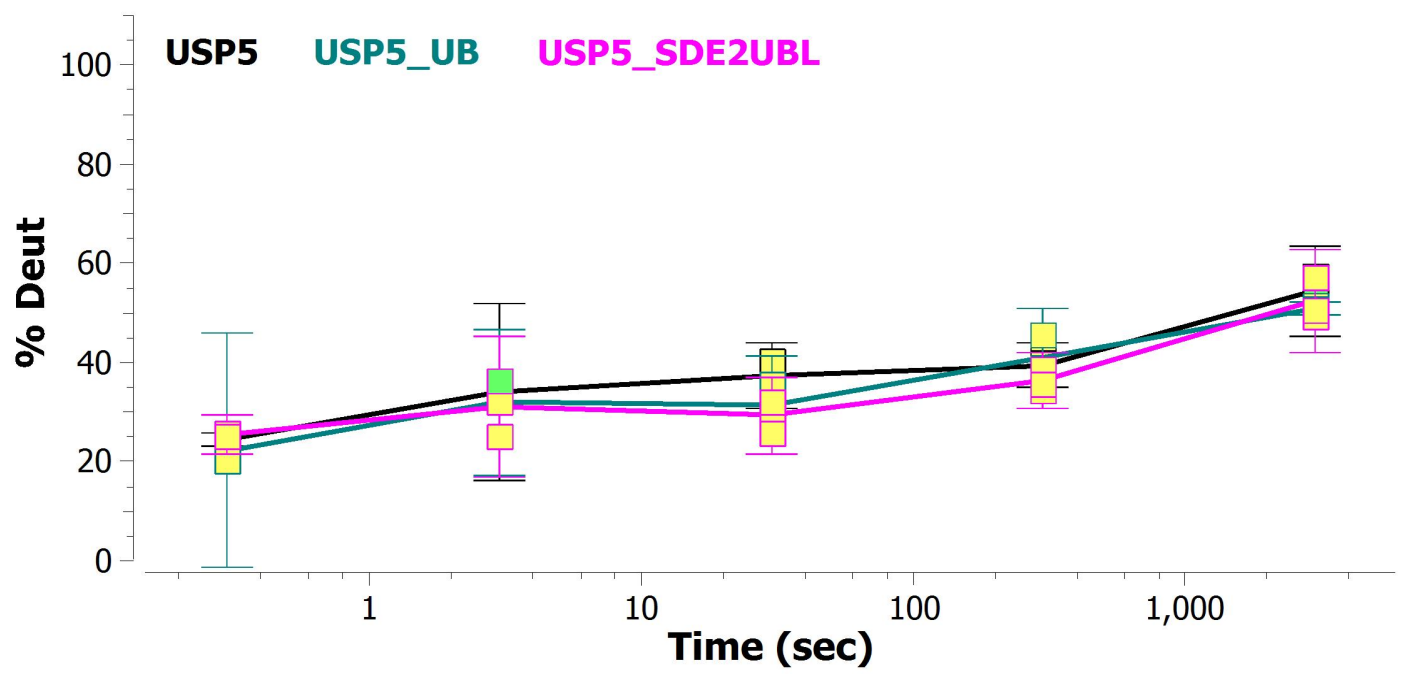

P45974 130-154: EIARDGLGGLPDIVRDRVTSAVEAL (#36)

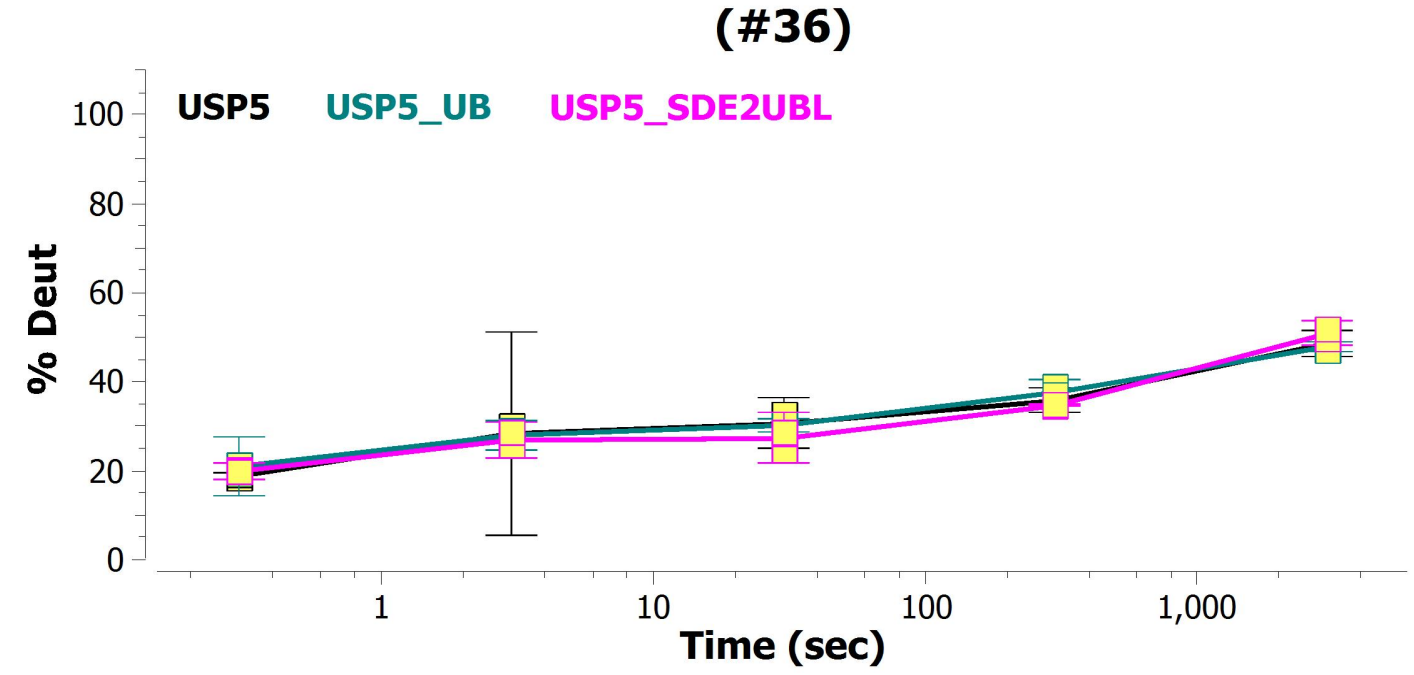

P45974 131-145: IARDGLGGLPDIVRD (#37)

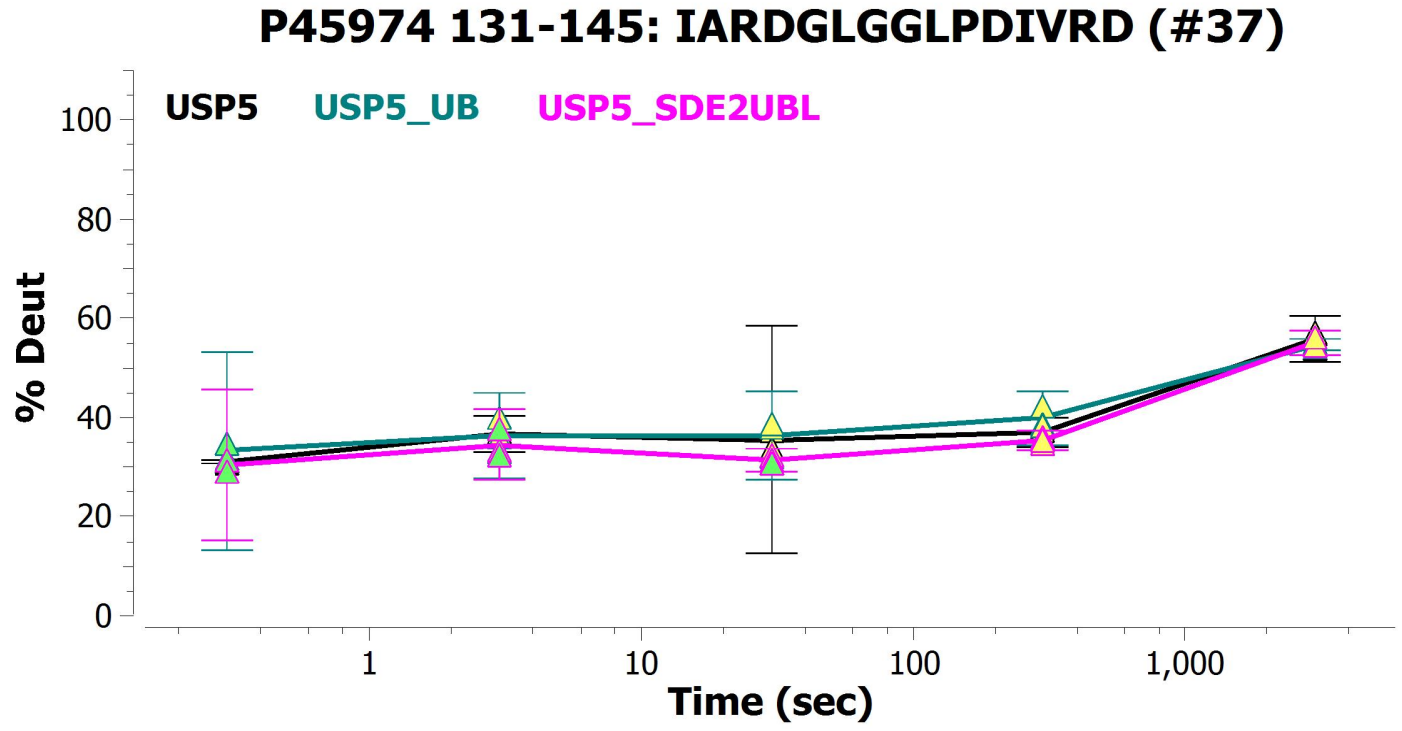

P45974 131-149: IARDGLGGLPDIVRDRVTS (#38)

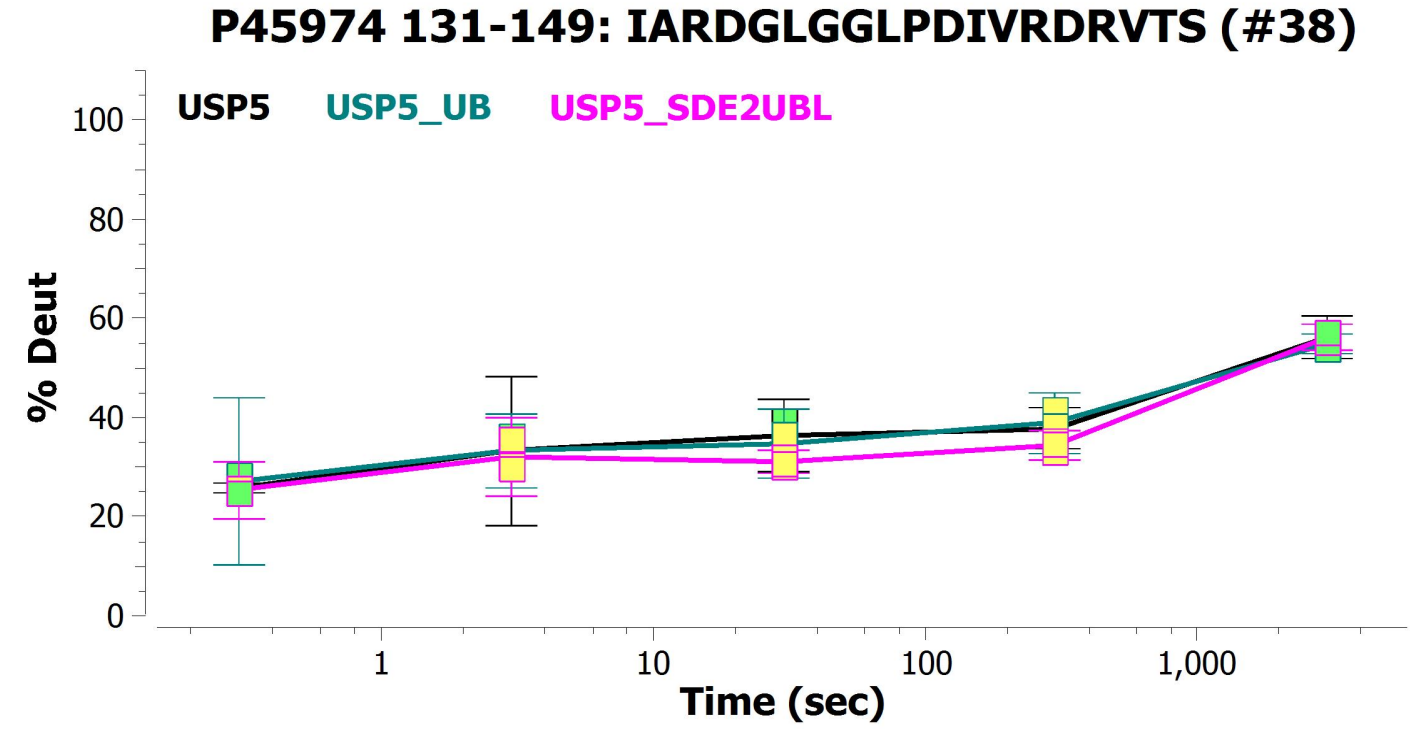

P45974 131-150: IARDGLGGLPDIVRDRVTSA (#39)

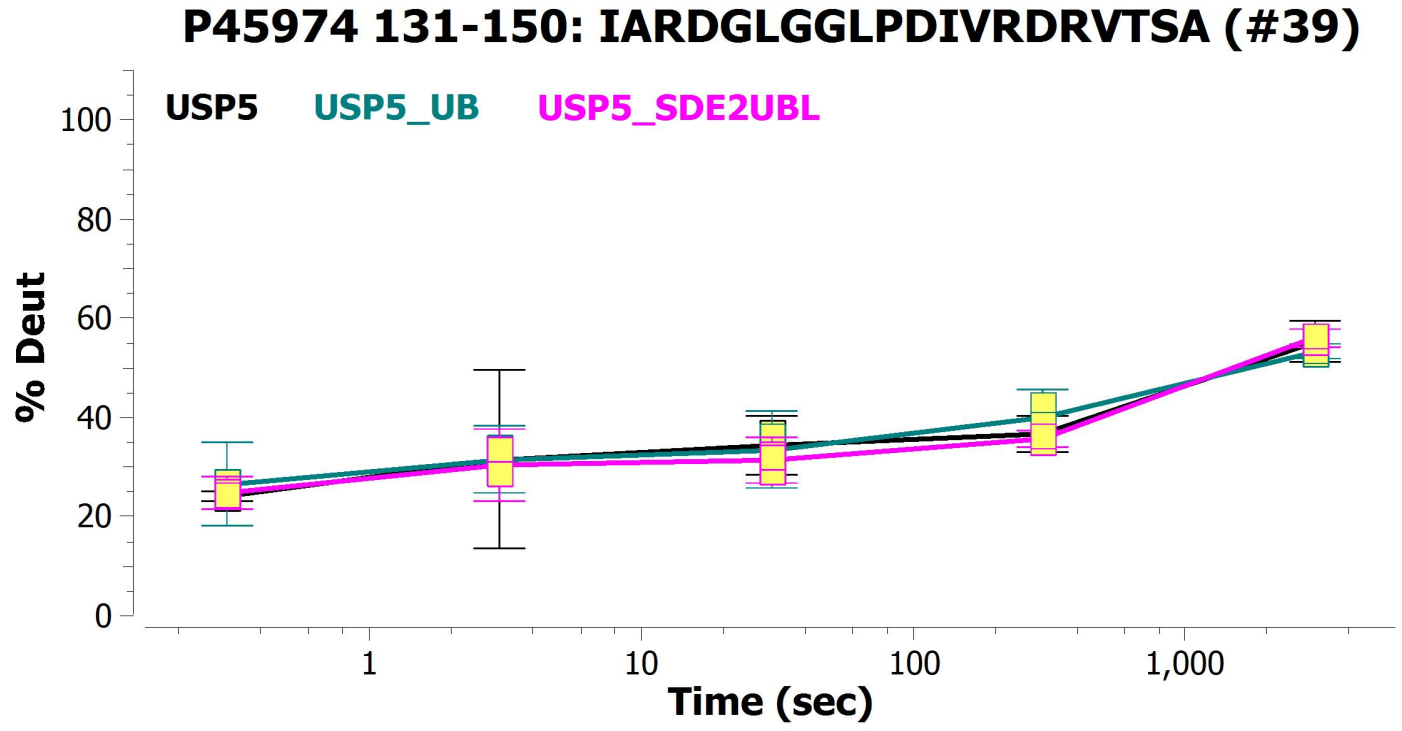

P45974 131-151: IARDGLGGLPDIVRDRVTSAV (#40)

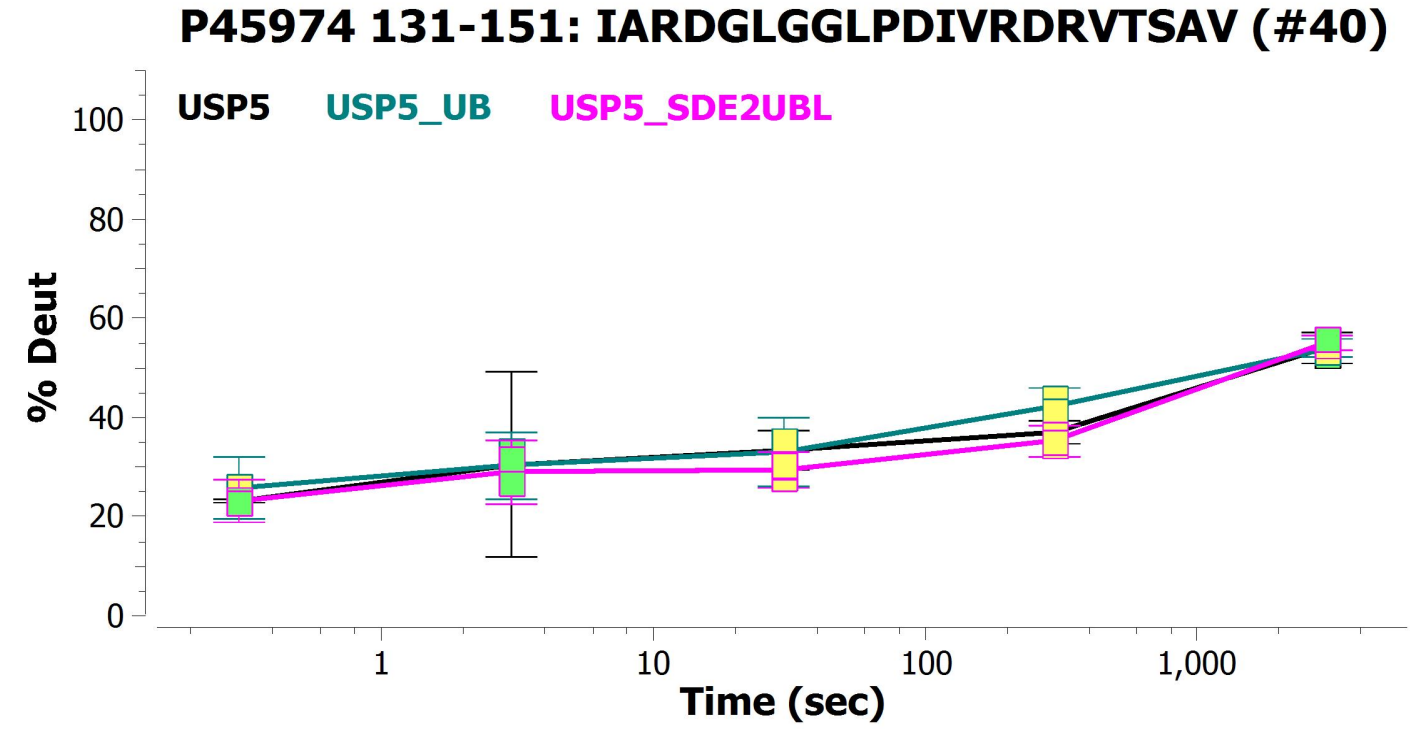

P45974 131-152: IARDGLGGLPDIVRDRVTSAVE (#41)

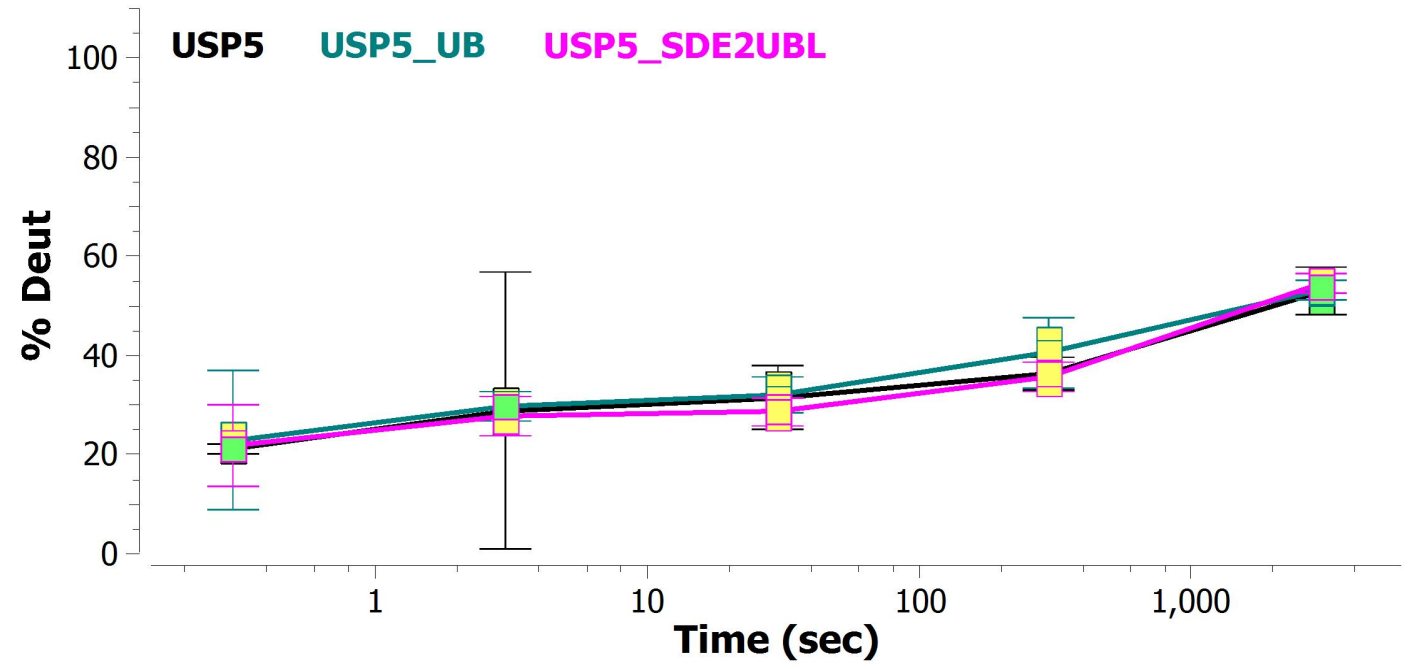

P45974 131-154: IARDGLGGLPDIVRDRVTSAVEAL (#42)

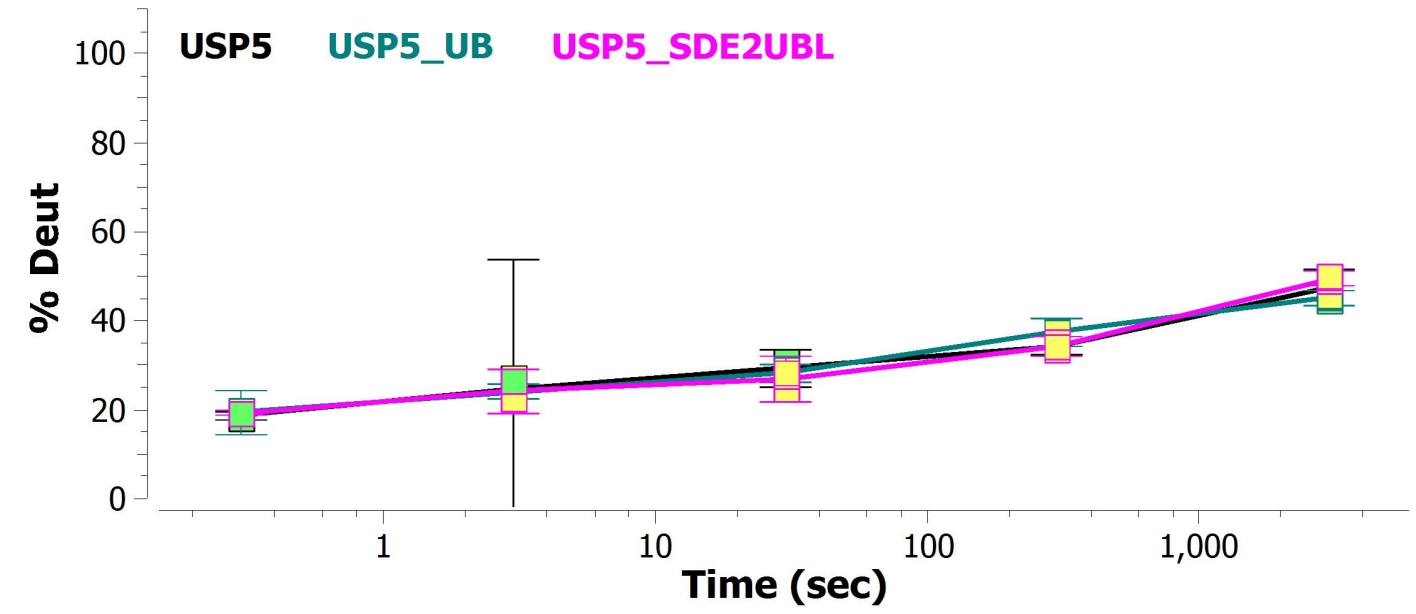

P45974 155-172: LSADSASRKQEVQAWDGE (#43)

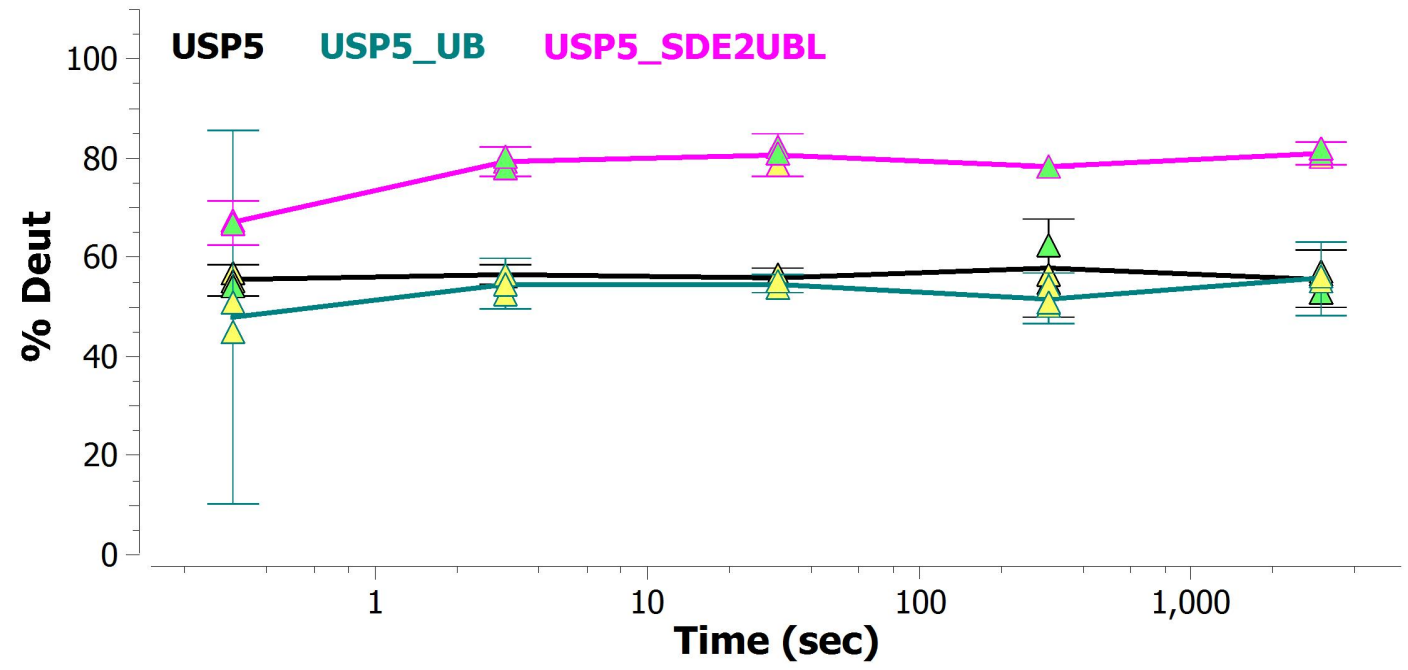

P45974 156-169: SADSASRKQEVQAW (#44)

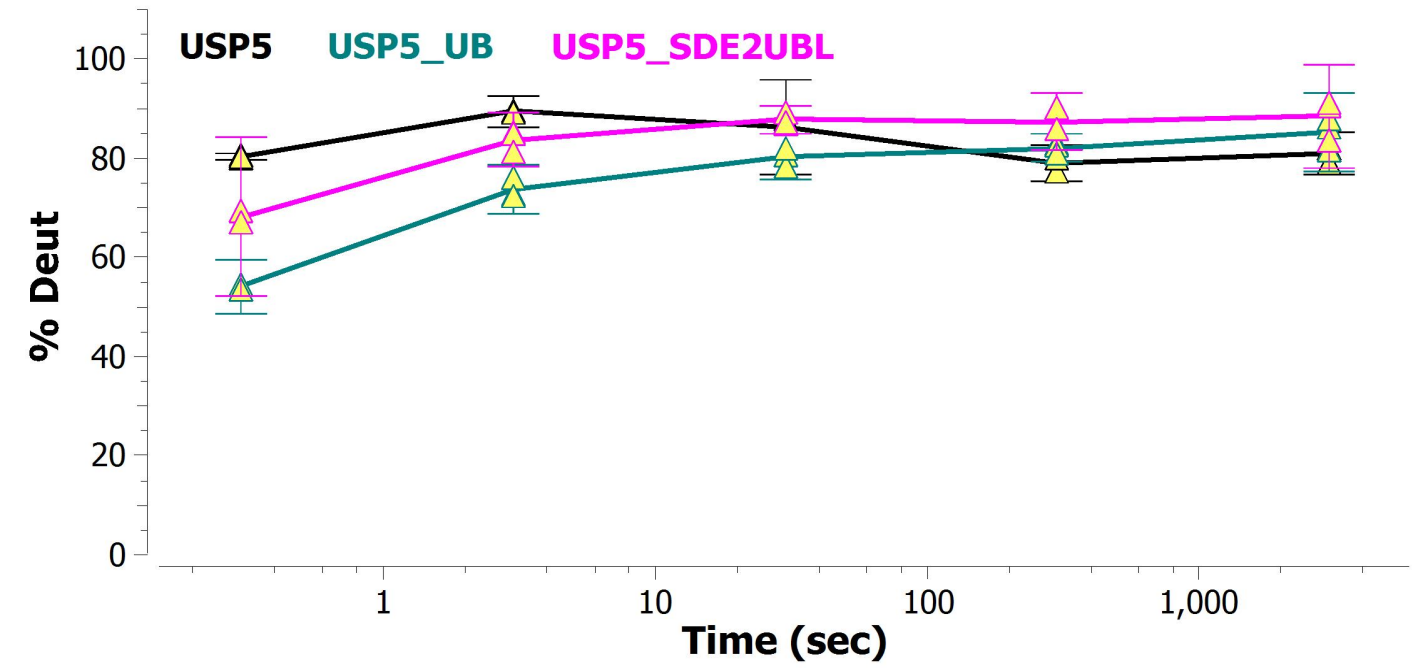

P45974 169-183: WDGEVRQVSKHAFSL (#45)

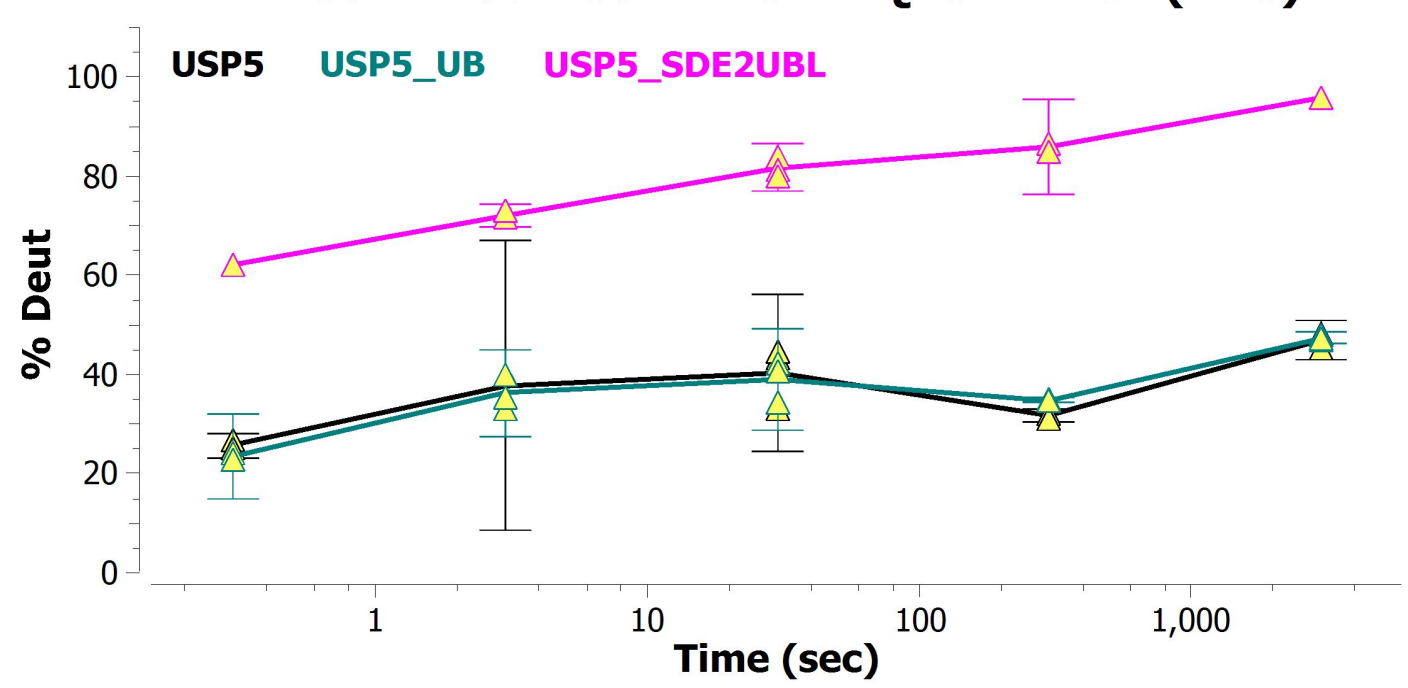

P45974 172-183: EVRQVSKHAFSL (#46)

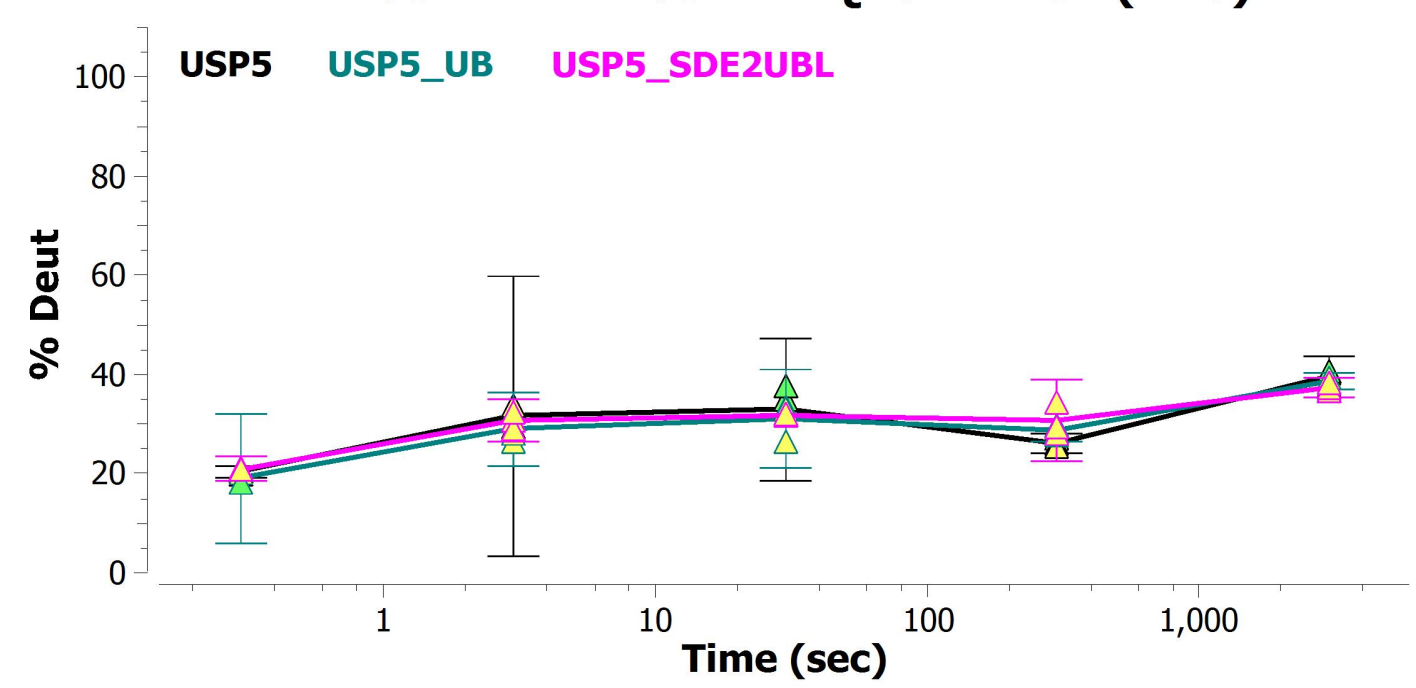

P45974 172-196: EVRQVSKHAFSLKQLDNPARIPPCG (#47)

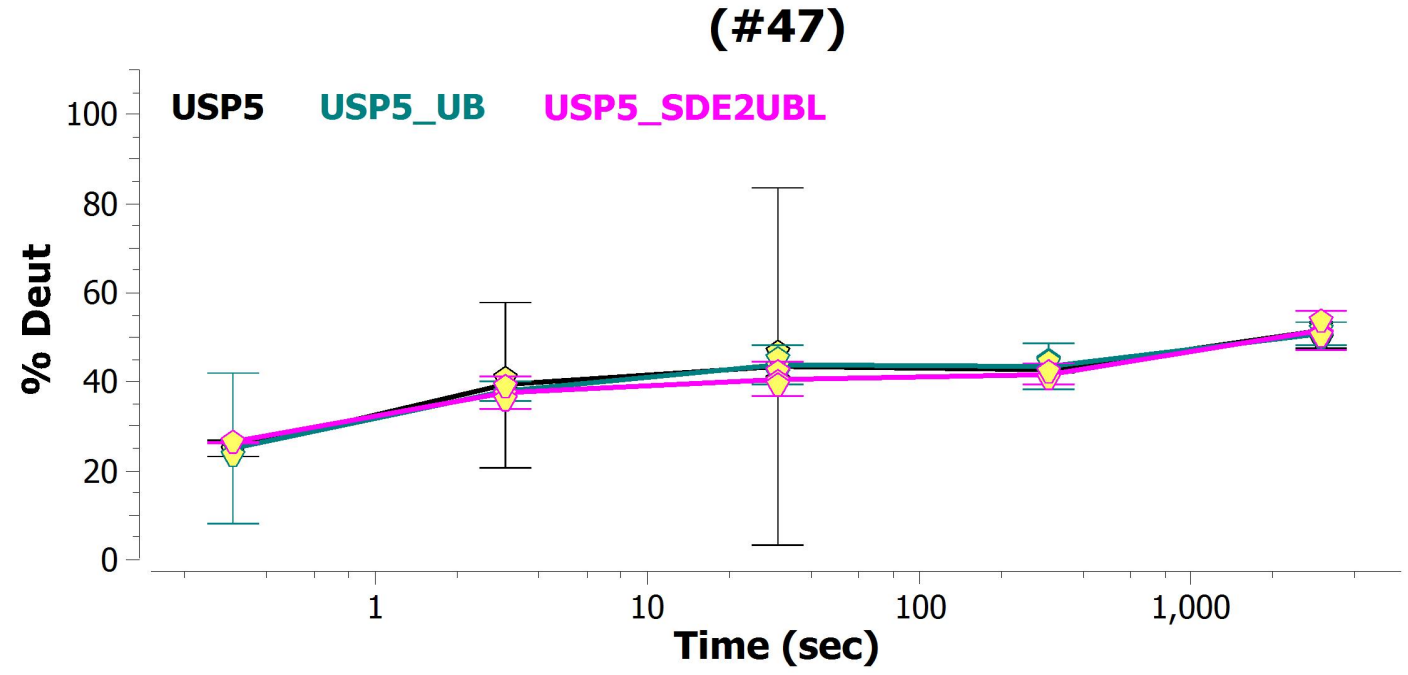

P45974 183-204: LKQLDNPARIPPCGWKCSKCDM (#48)

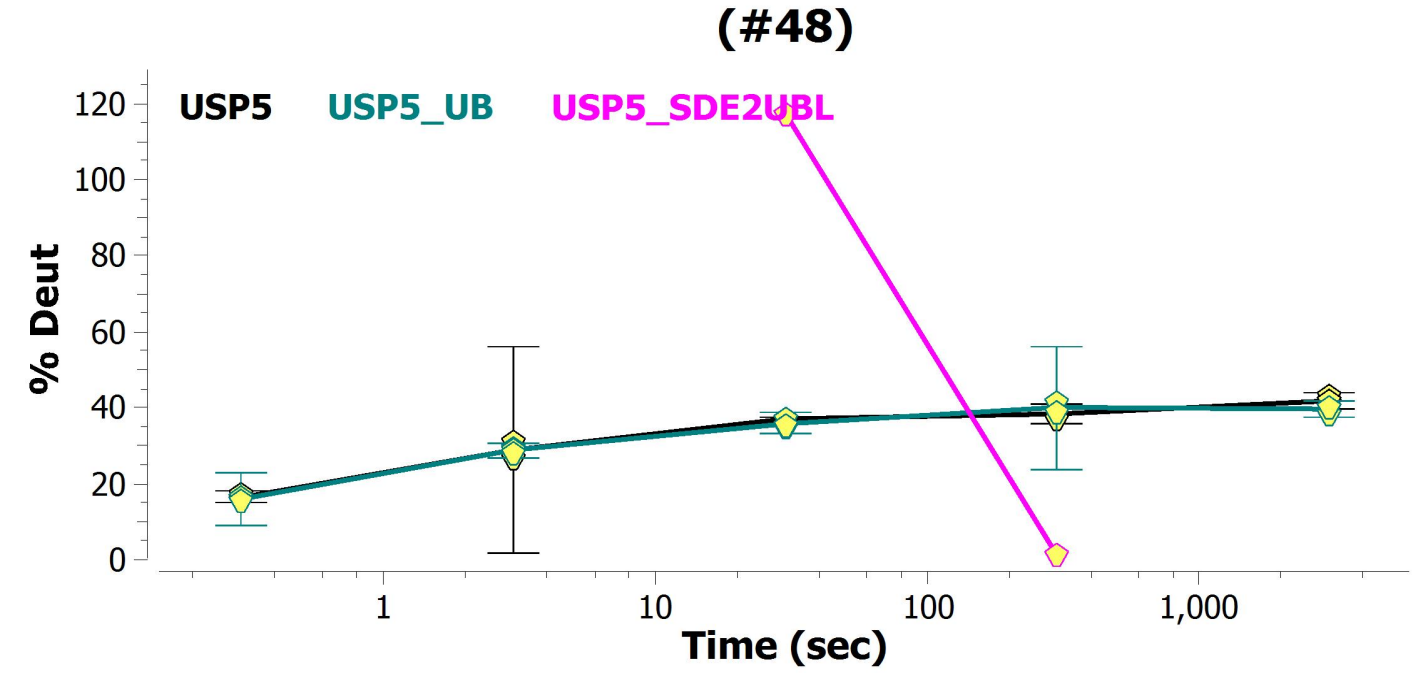

P45974 184-204: KQLDNPARIPPCGWKCSKCDM (#49)

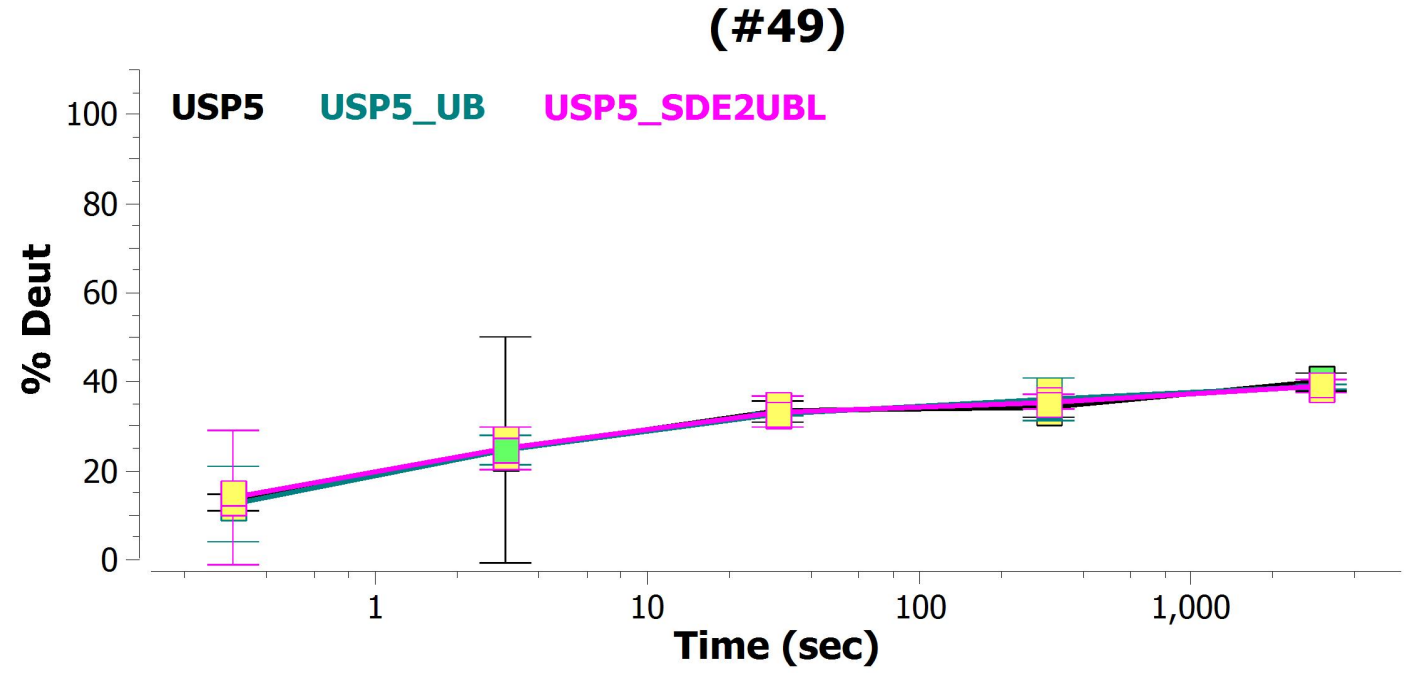

P45974 209-218: WLNLTDGSIL (#50)

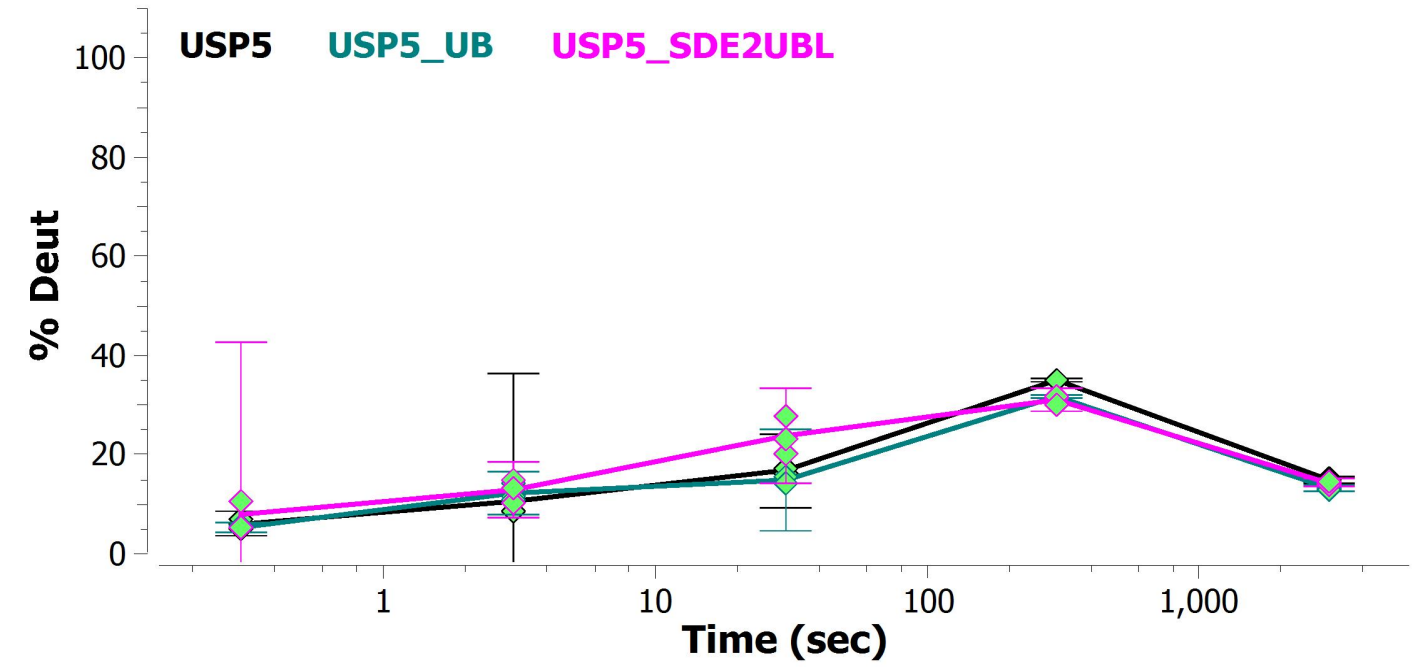

P45974 213-218: TDGSIL (#51)

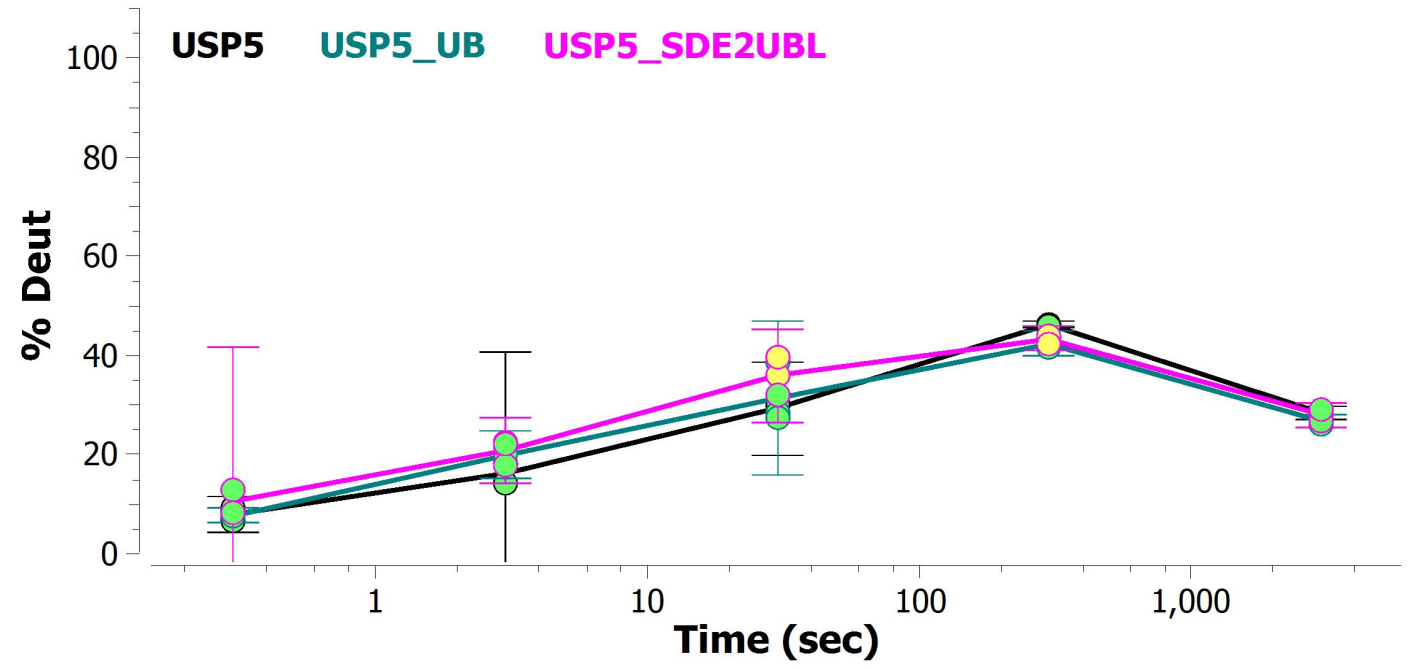

P45974 219-235: CGRRYFDGSGGNNHAVE (#52)

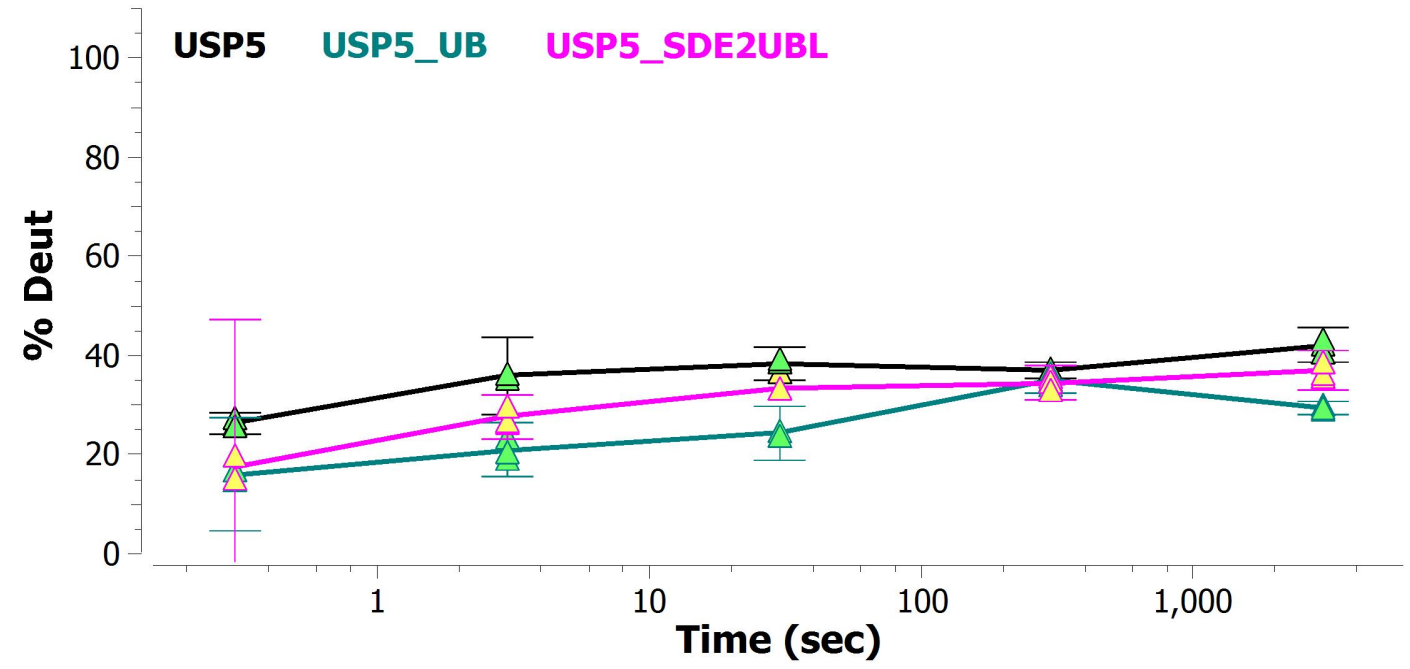

P45974 219-236: CGRRYFDGSGGNNHAVEH (#53)

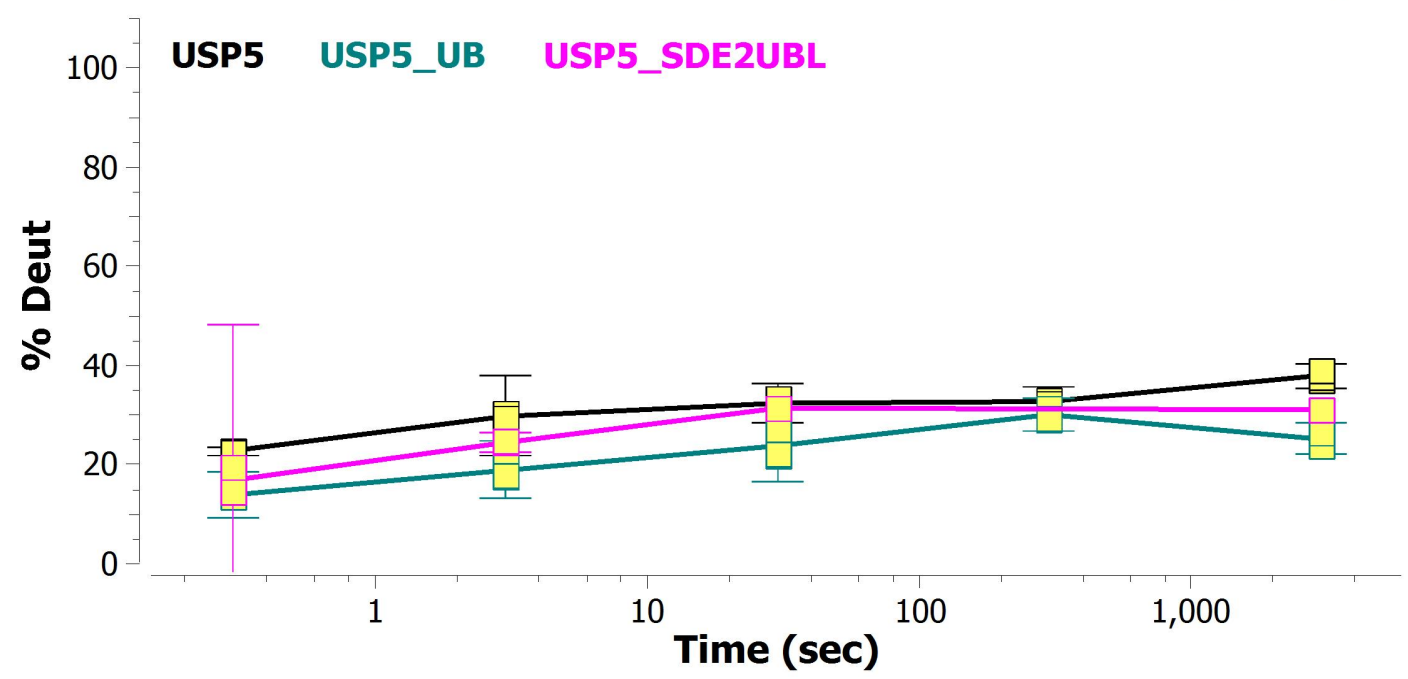

P45974 219-245: CGRRYFDGSGGNNHAVEHYRETGYPLA (#54)

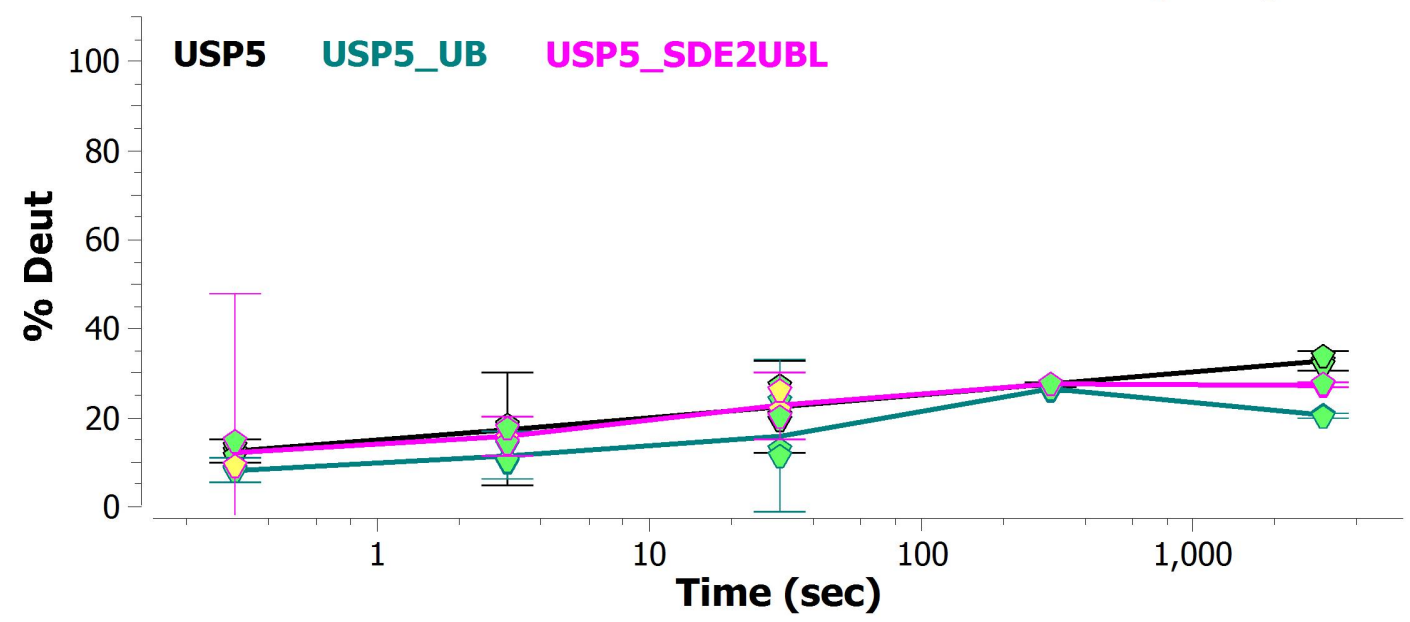

P45974 219-248: CGRRYFDGSGGNNHAVEHYRETGYPLAVKL (#55)

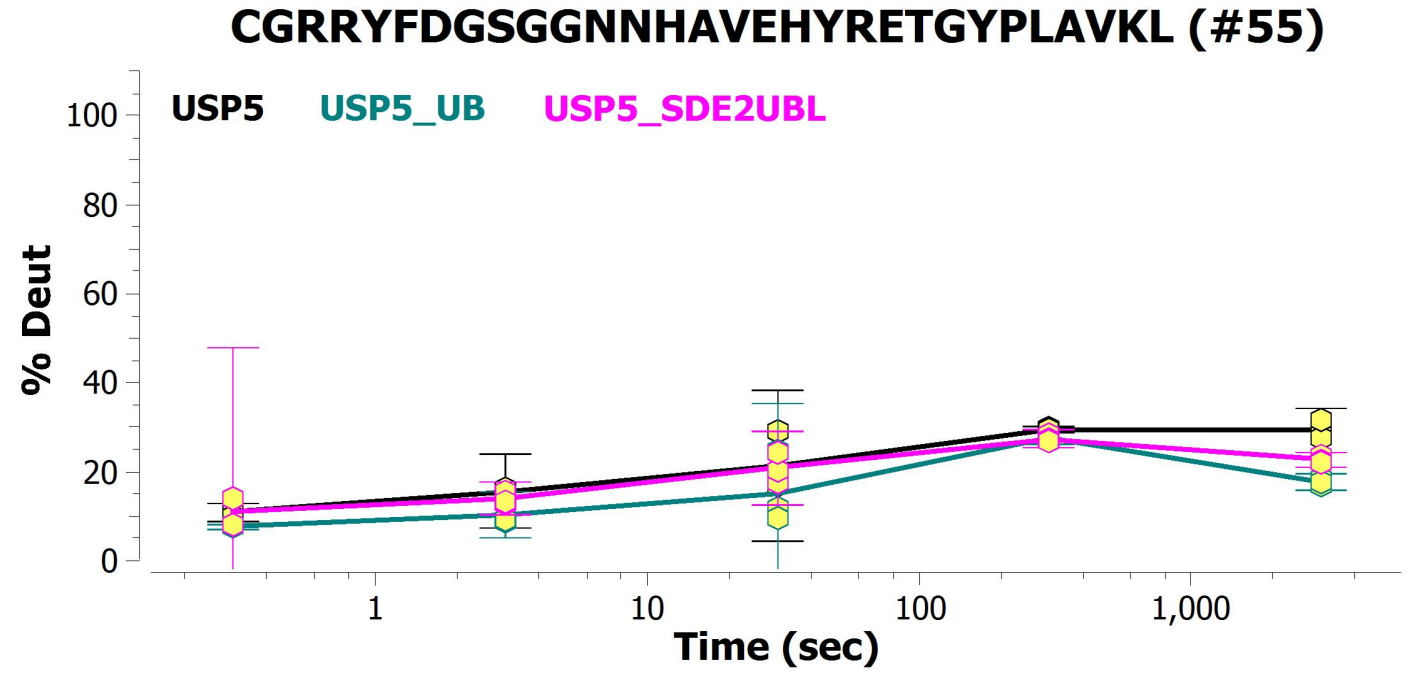

P45974 230-263: NNHAVEHYRETGYPLAVKLGTITPDGADVYSYDE (#56)

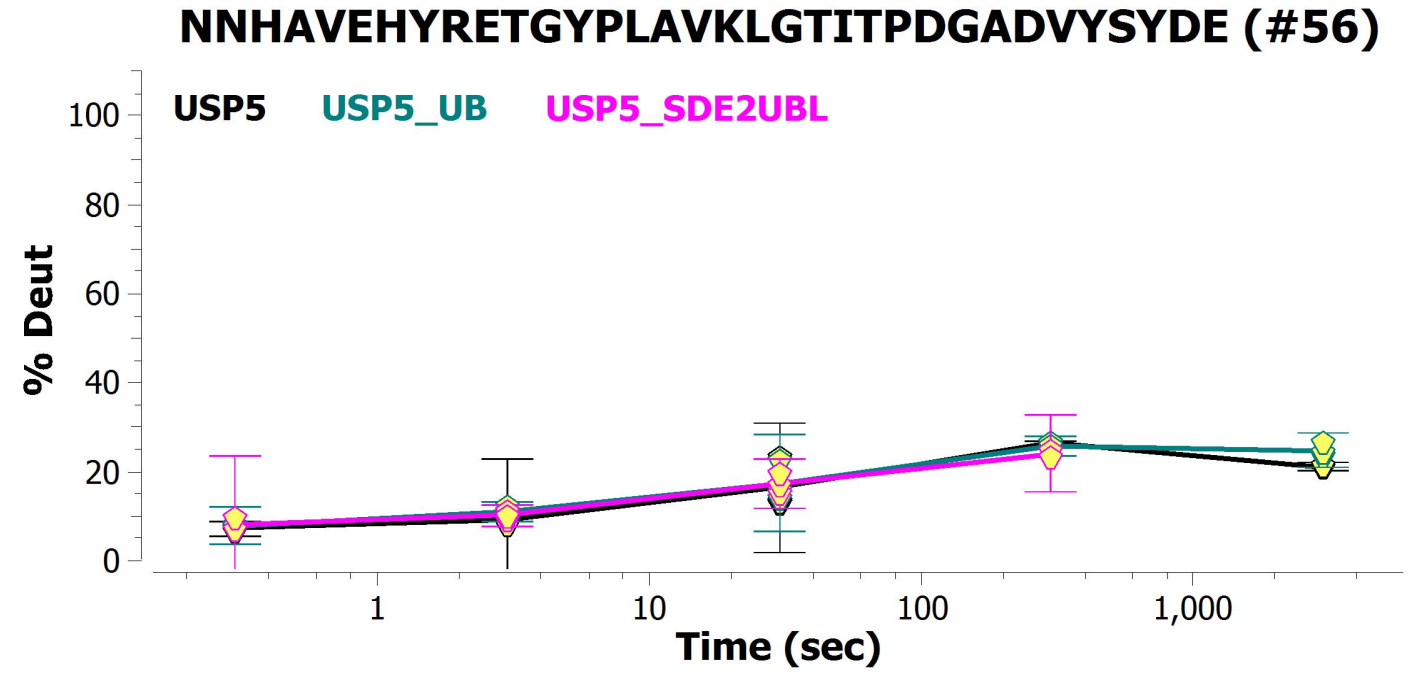

P45974 232-245: HAVEHYRETGYPLA (#57)

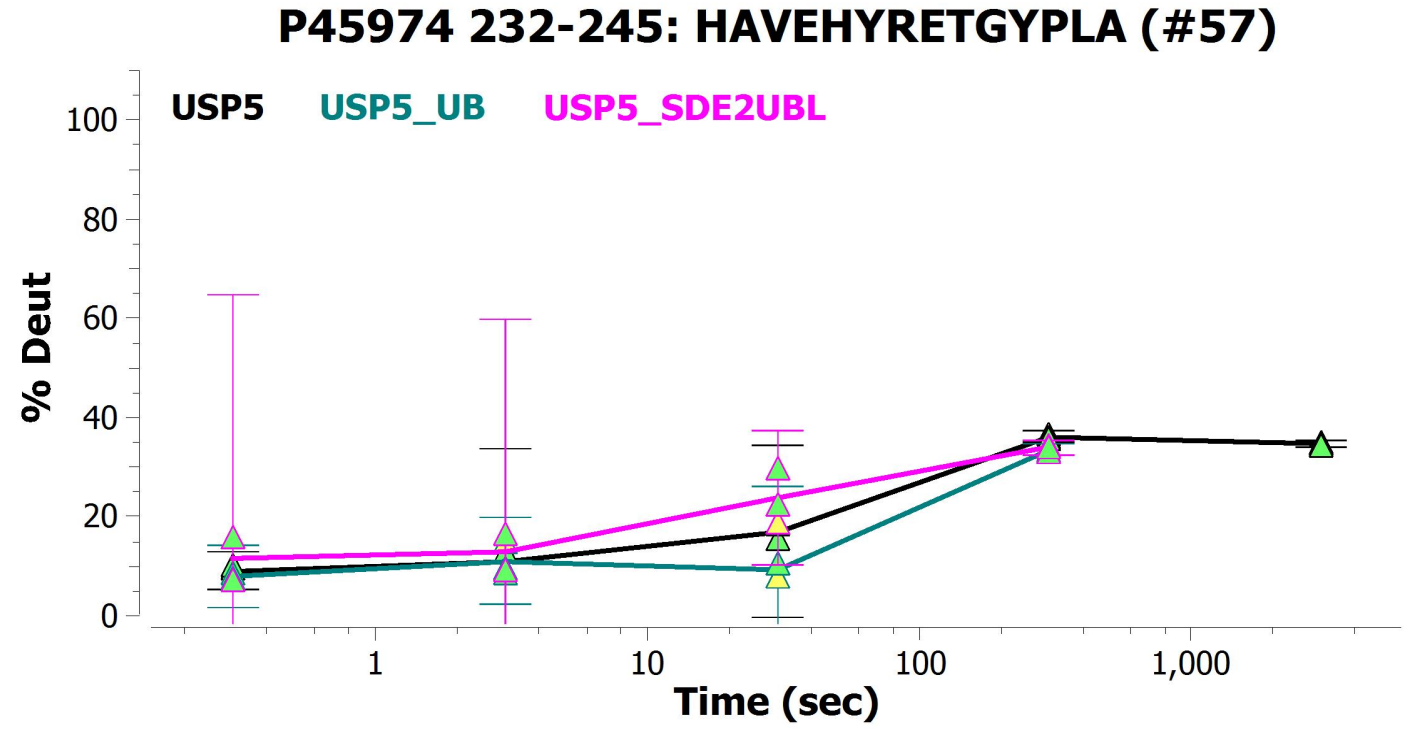

P45974 246-257: VKLGTITPDGAD (#58)

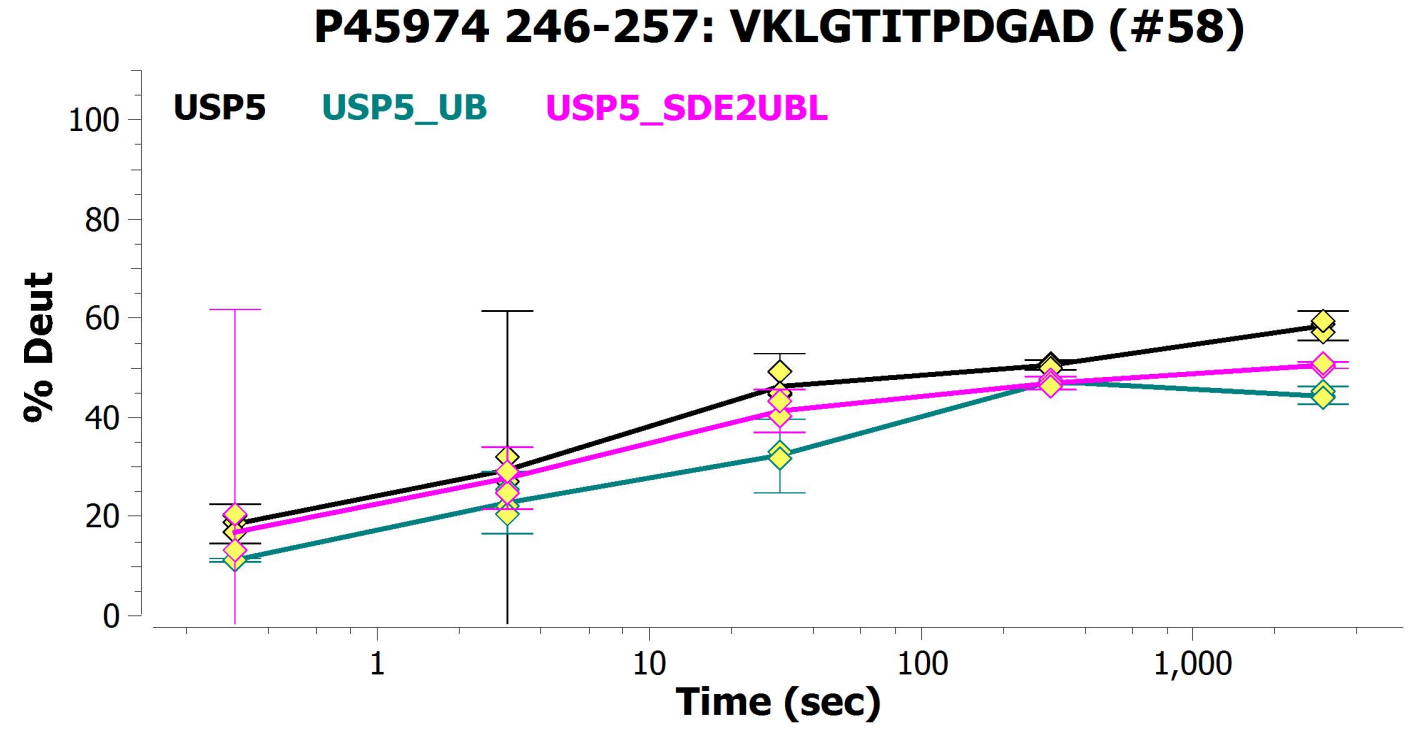

P45974 246-258: VKLGTITPDGADV (#59)

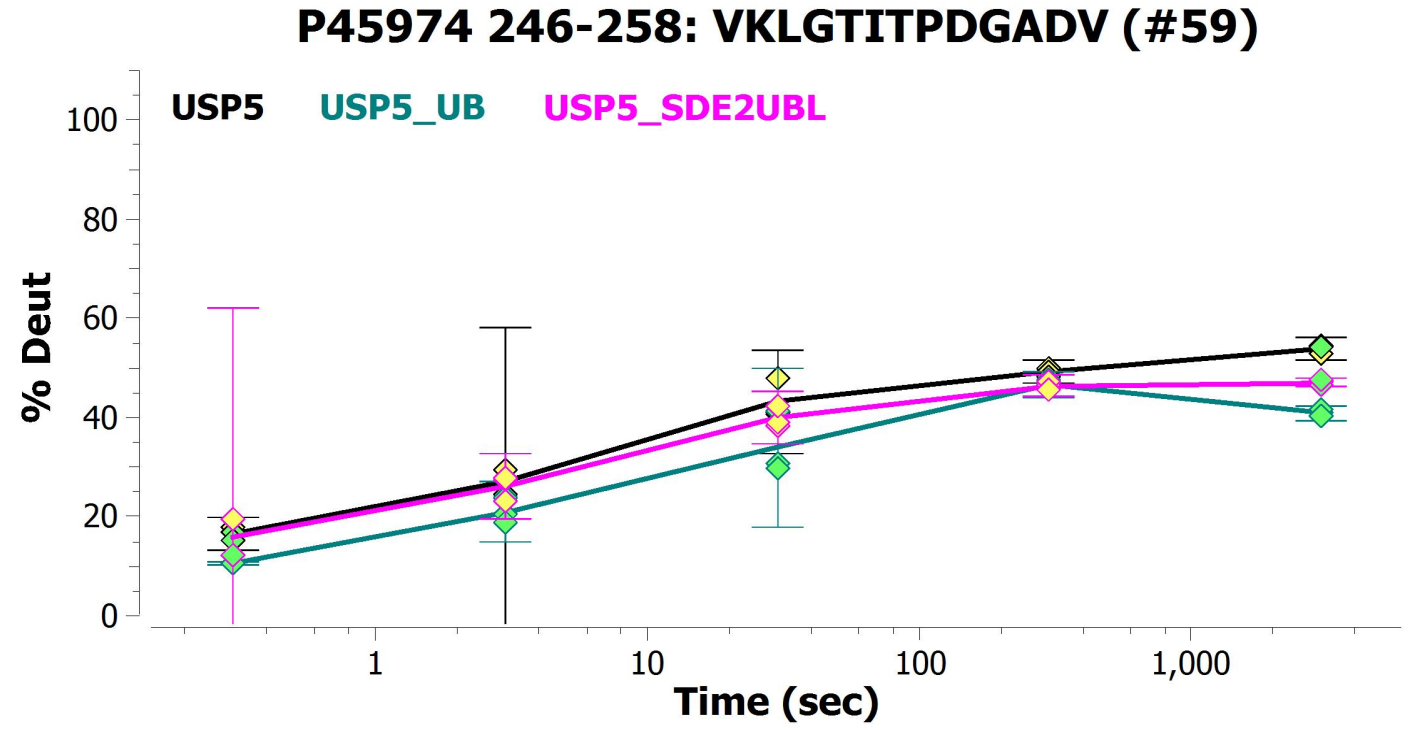

P45974 246-259: VKLGTITPDGADVY (#60)

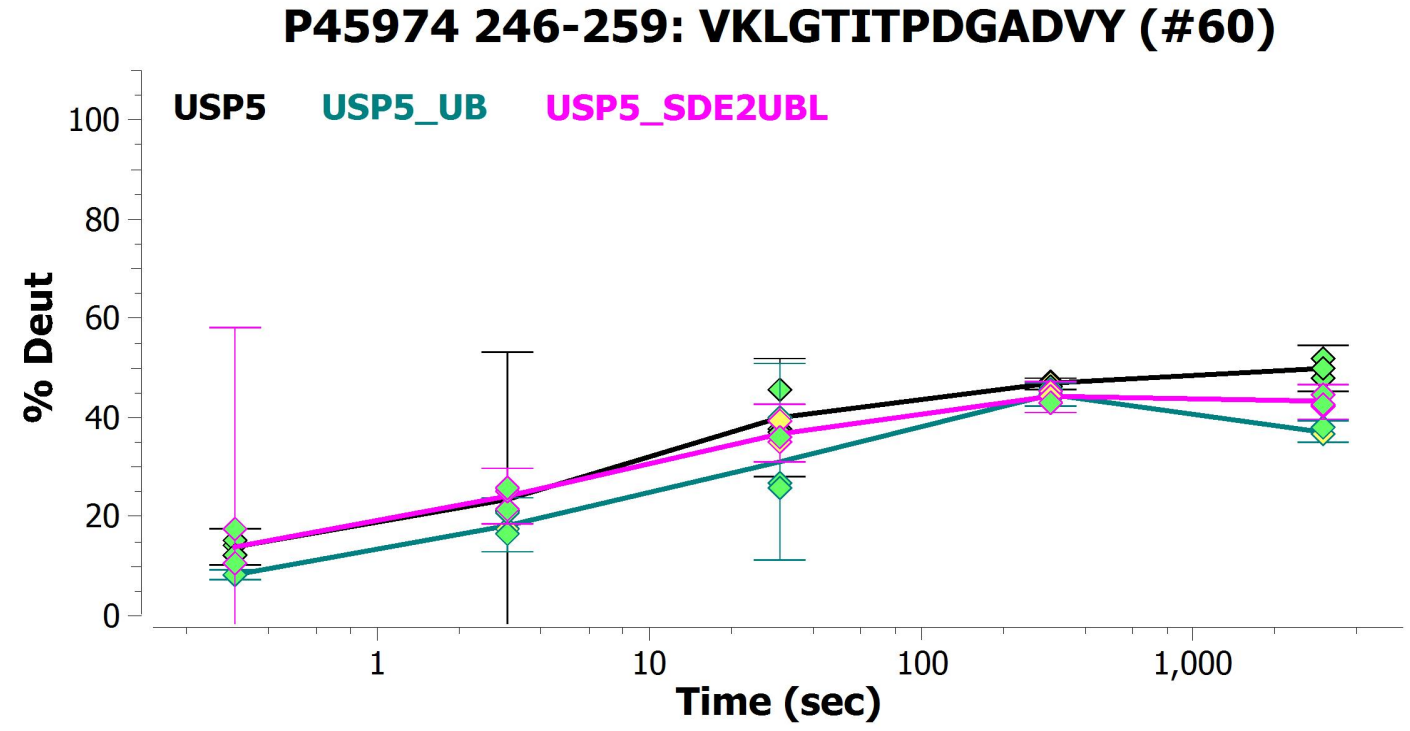

P45974 246-260: VKLGTITPDGADVYS (#61)

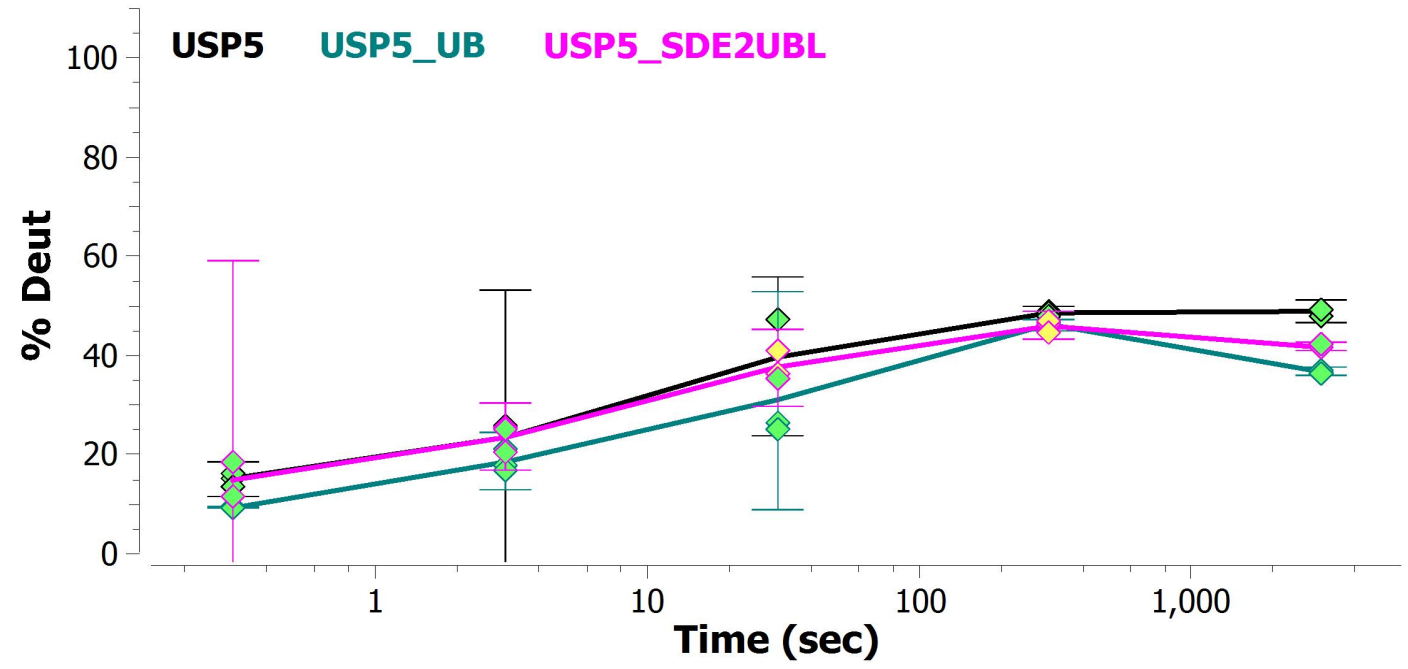

P45974 266-279: MVLDPSLAEHLSHF (#62)

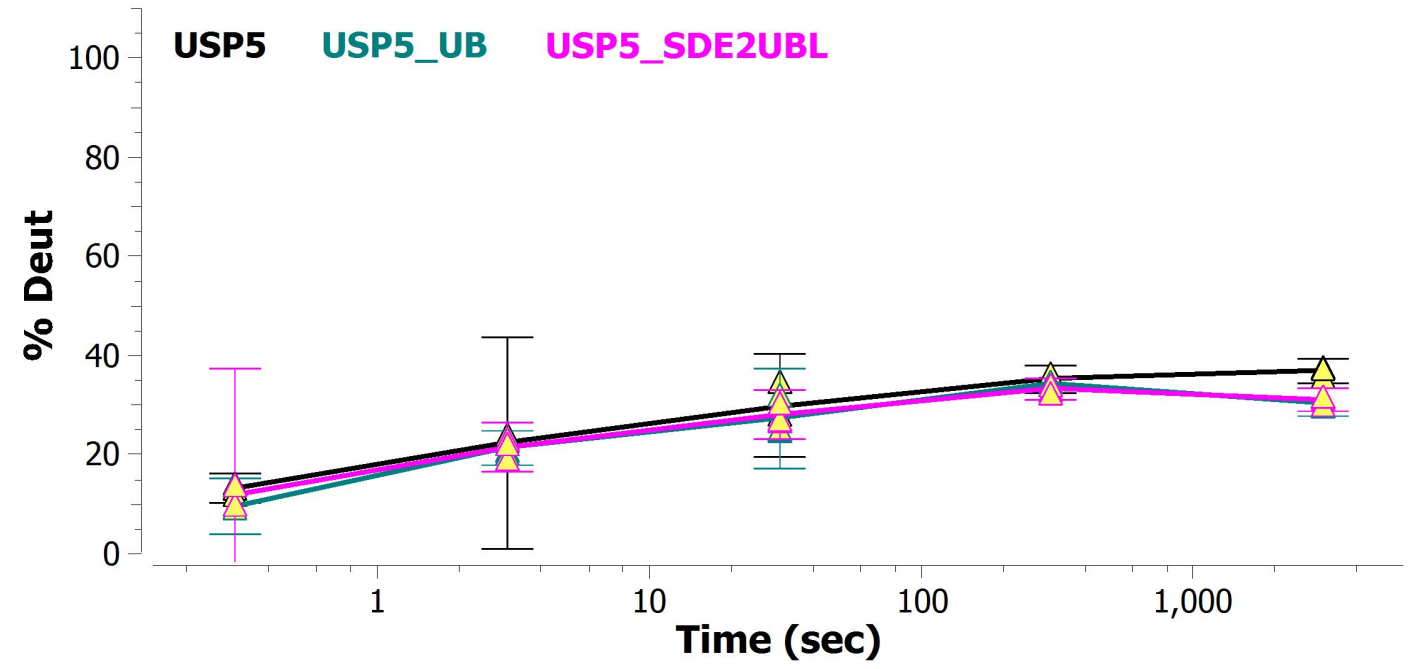

P45974 266-282: MVLDPSLAEHLSHFGID (#63)

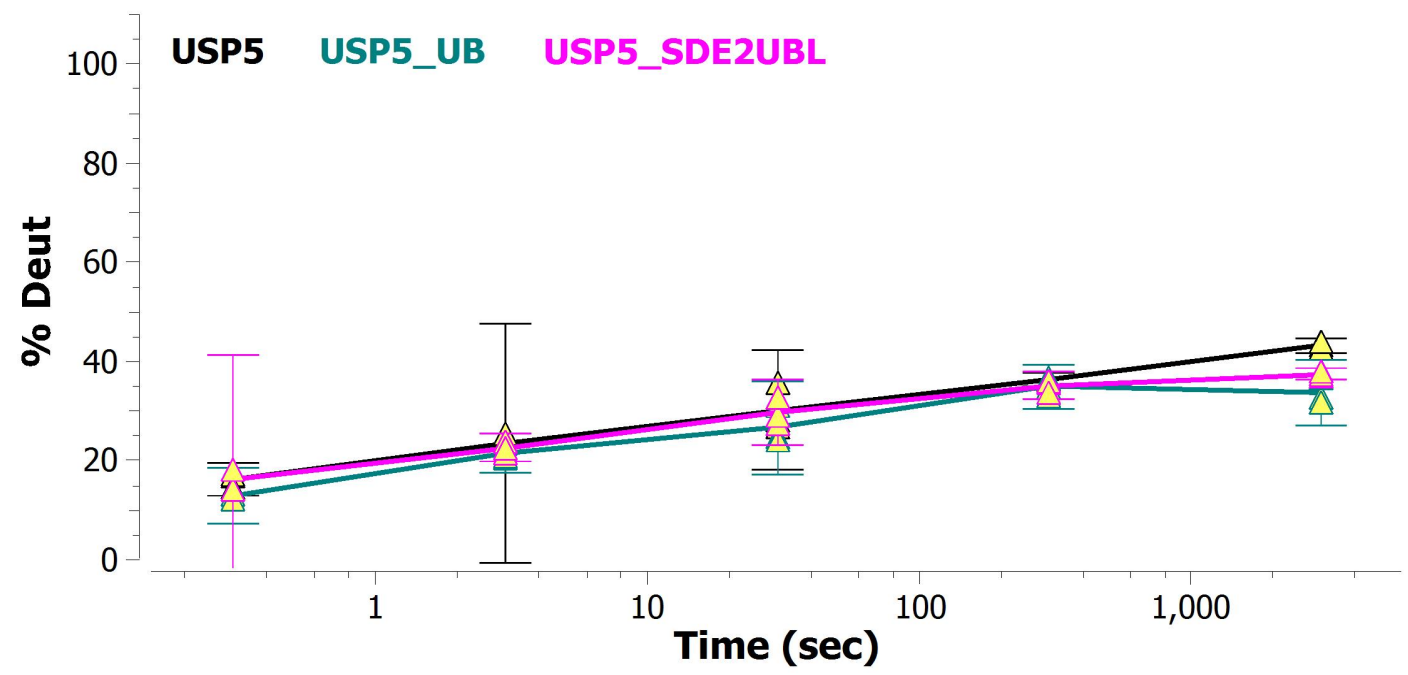

P45974 273-283: AEHLSHFGIDM (#64)

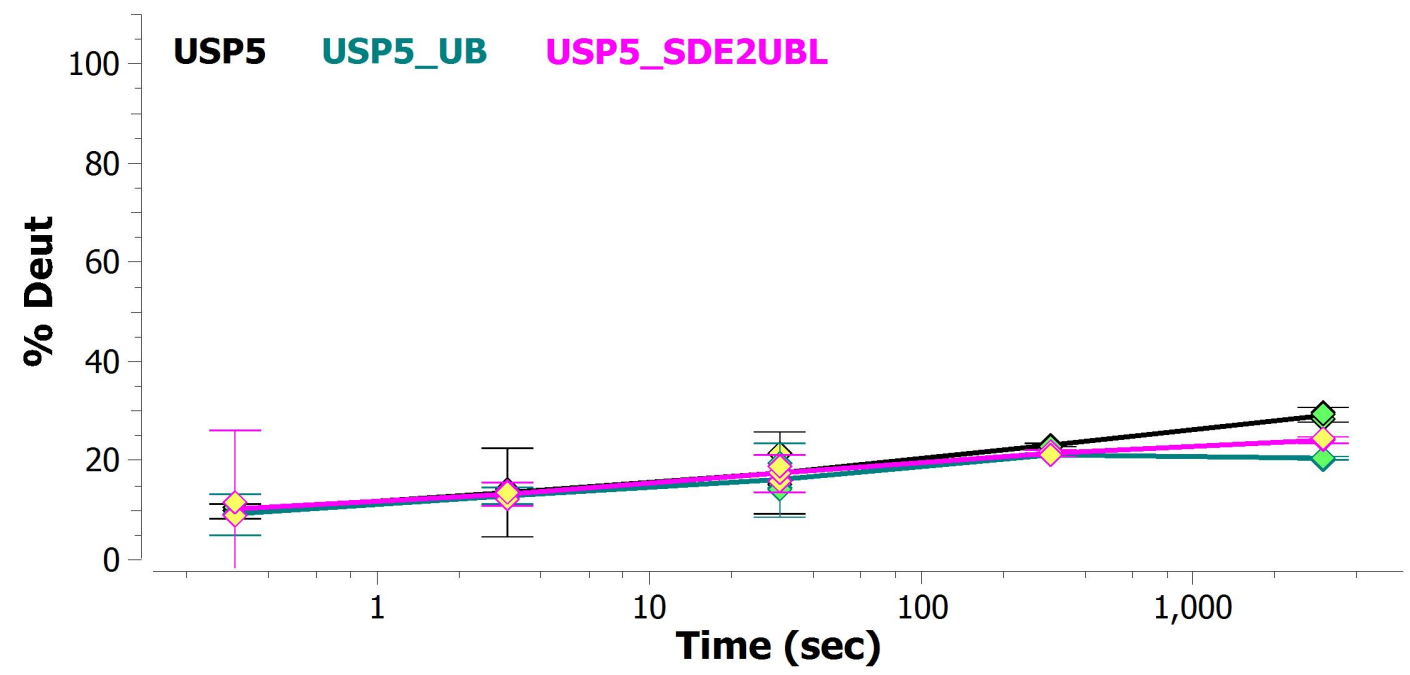

P45974 273-284: AEHLSHFGIDML (#65)

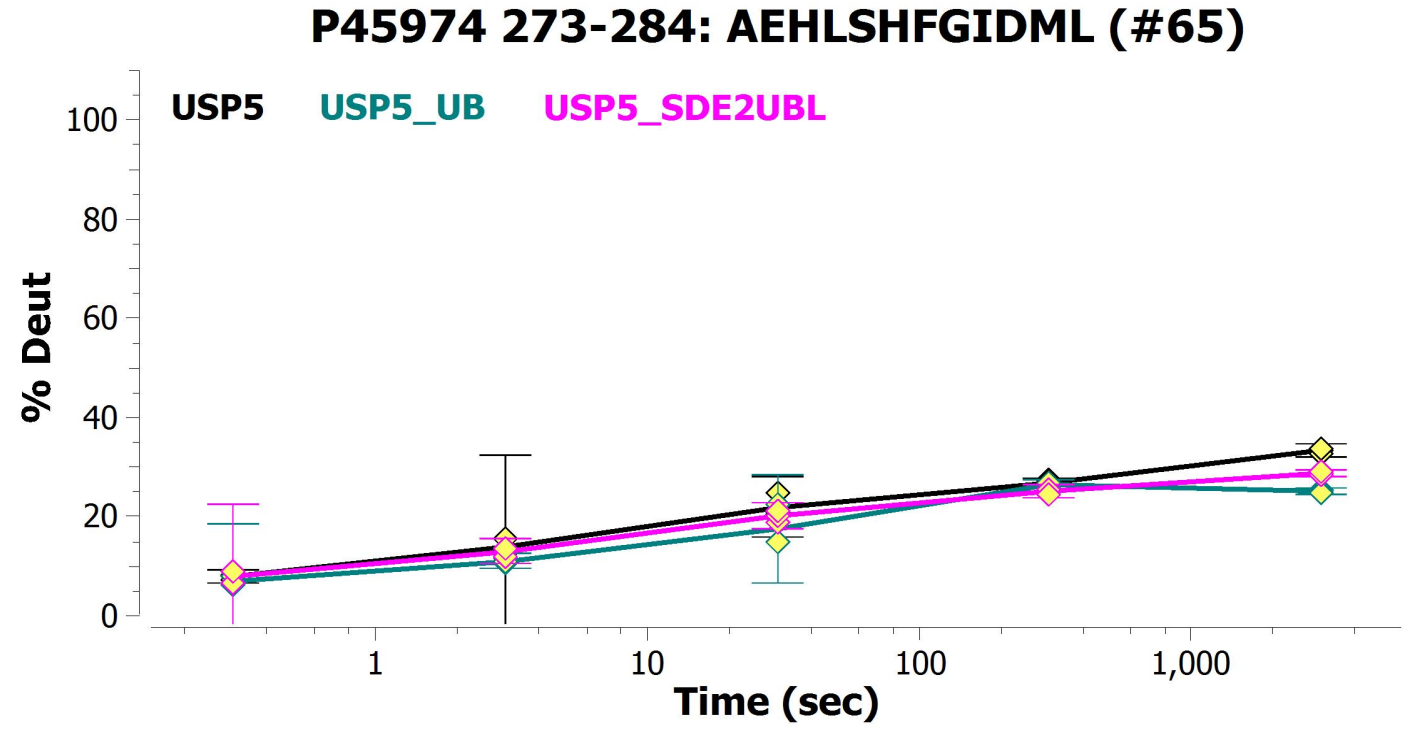

P45974 275-283: HLSHFGIDM (#66)

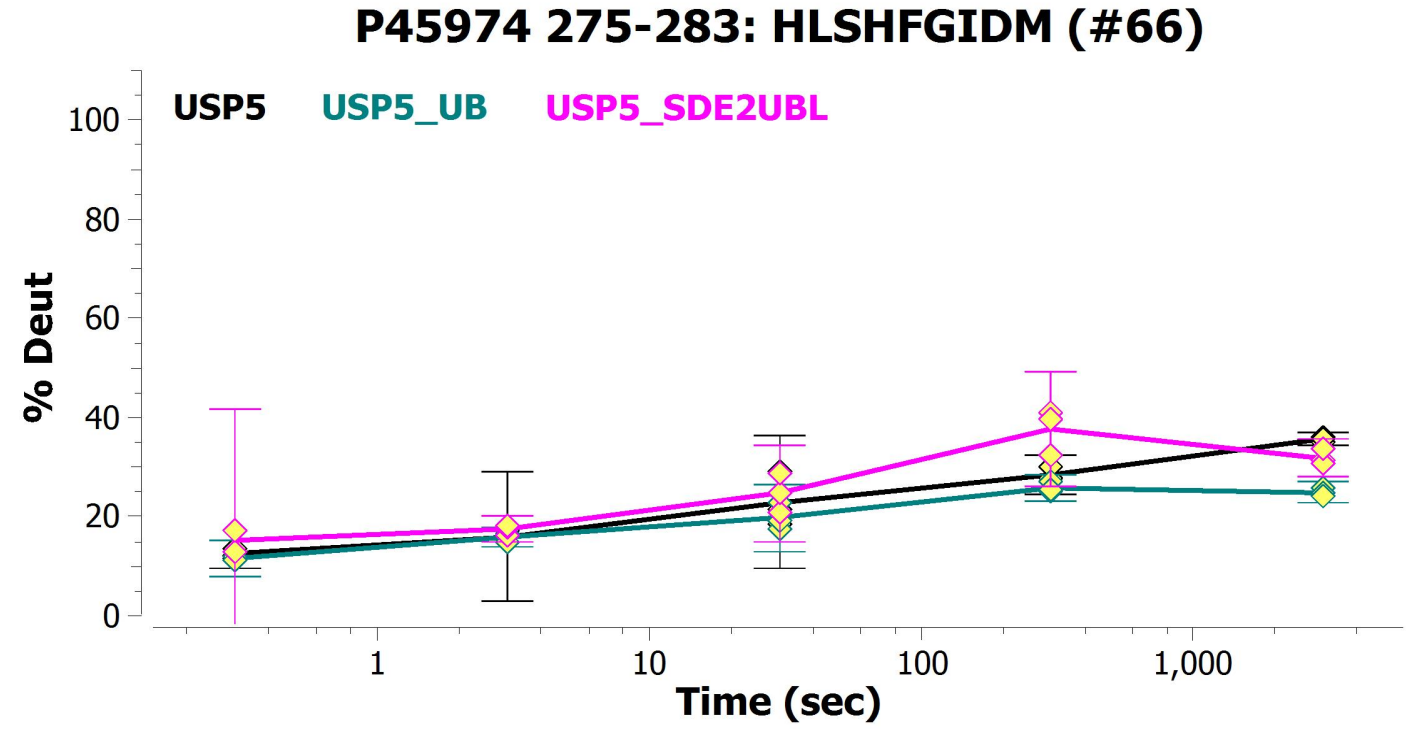

P45974 283-295: MLKMQKTDKTMTE (#67)

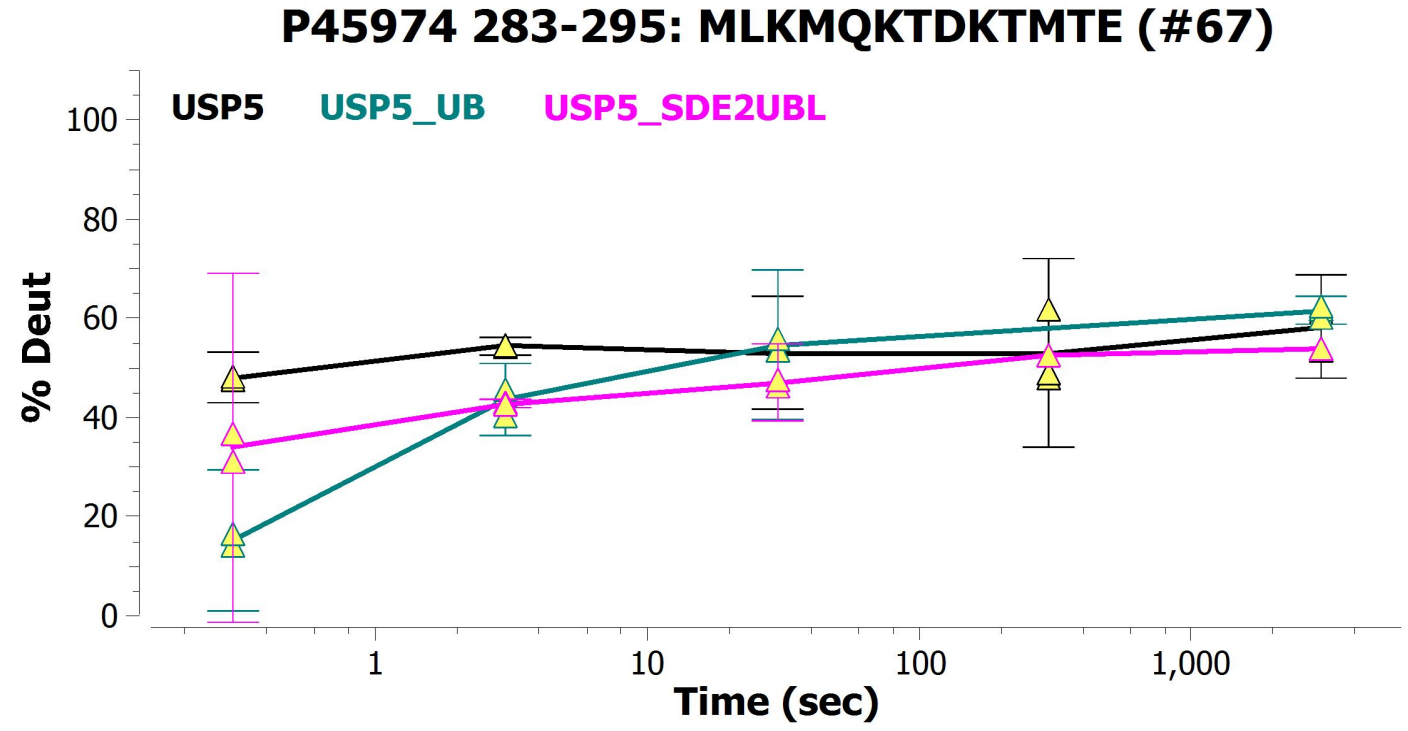

P45974 283-296: MLKMQKTDKTMTEL (#68)

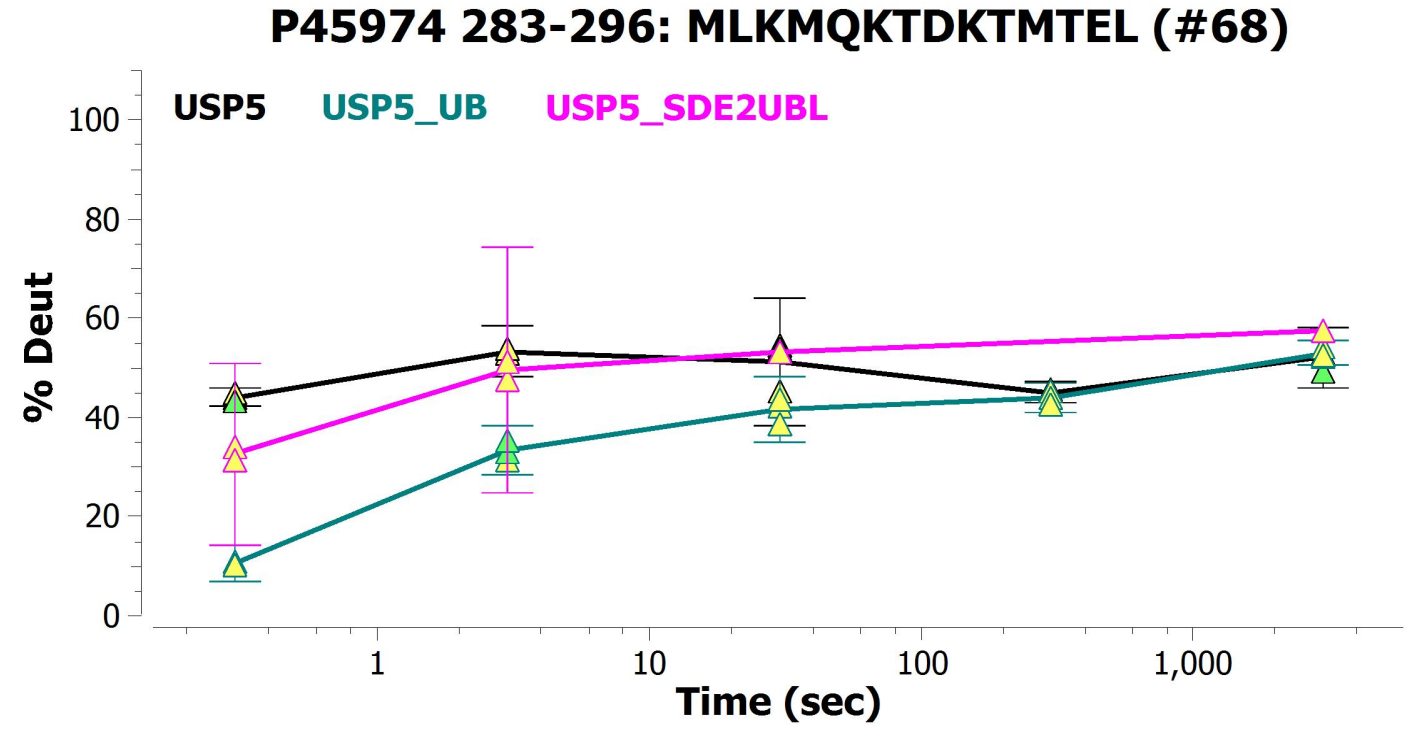

P45974 284-295: LKMQKTDKTMTE (#69)

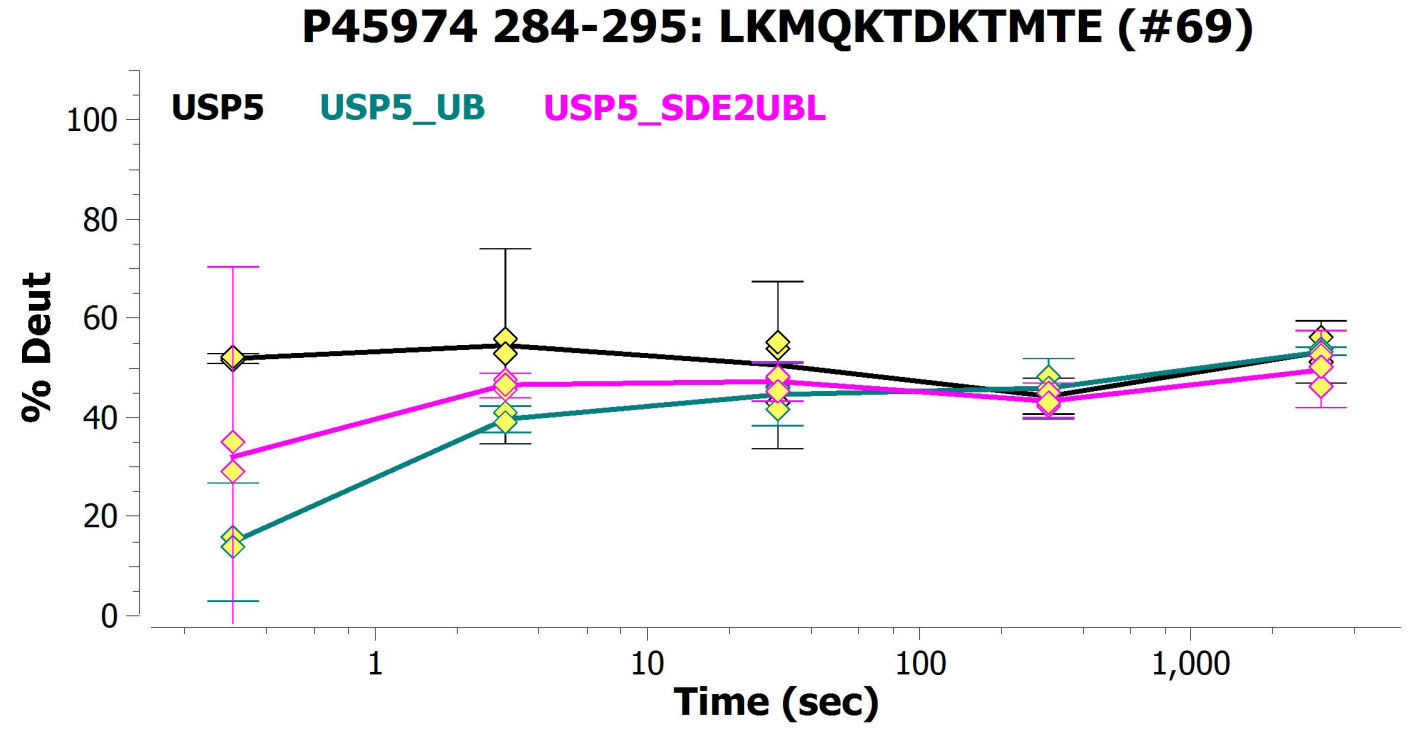

P45974 284-296: LKMQKTDKTMTEL (#70)

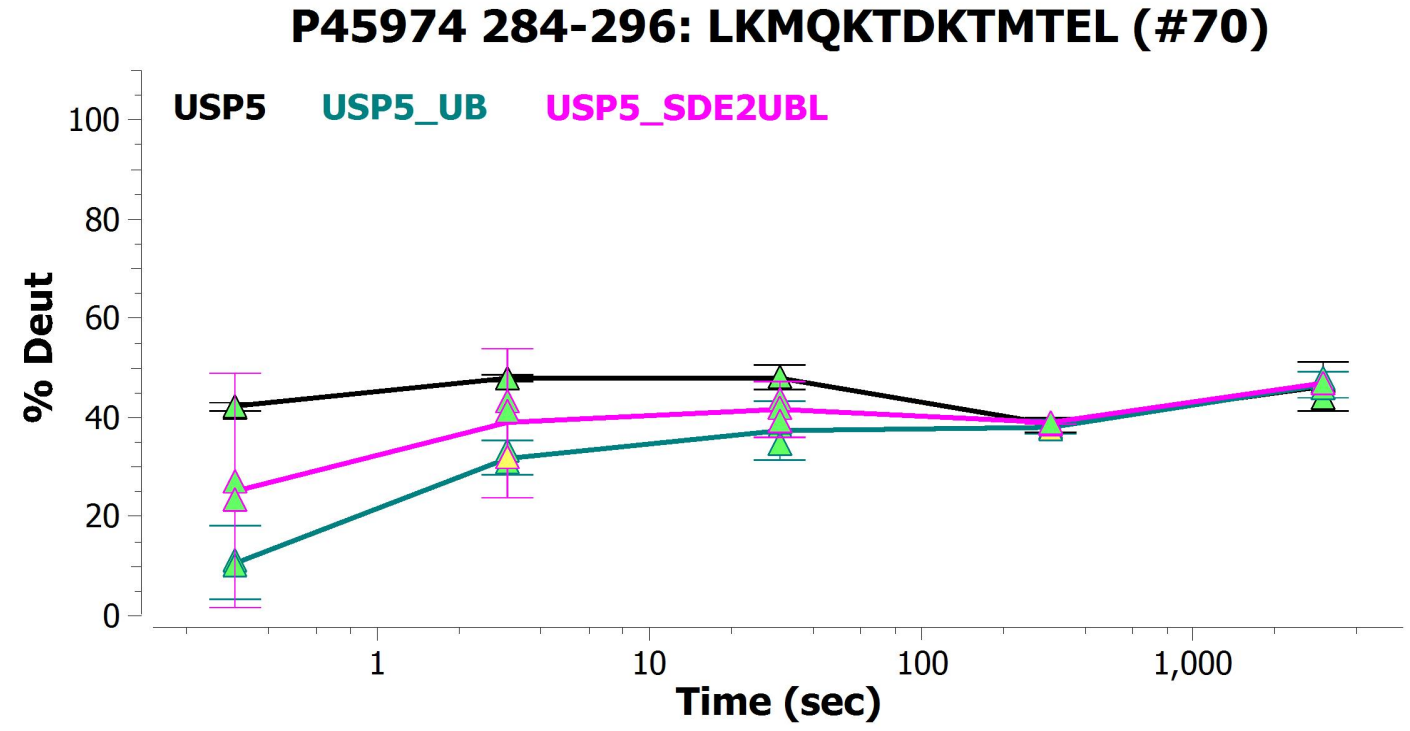

P45974 297-306: EIDMNQRIGE (#71)

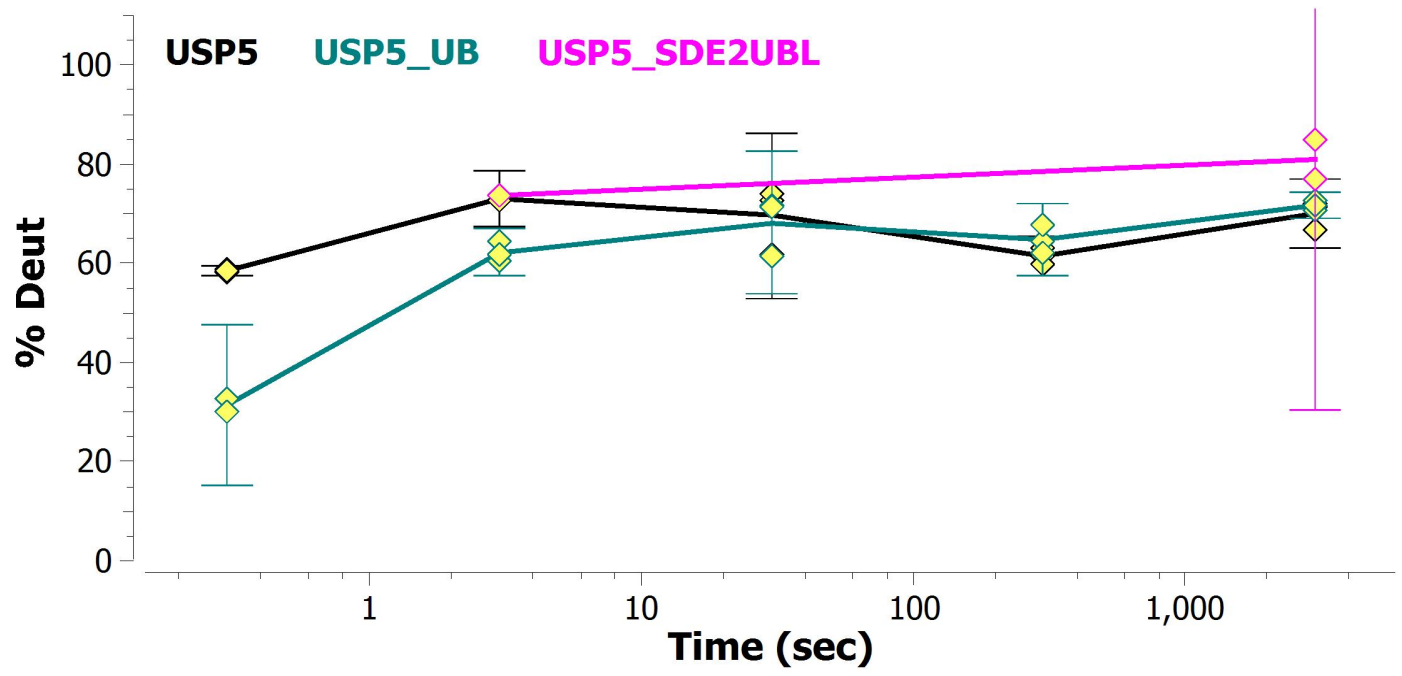

P45974 301-307: NQRIGEW (#72)

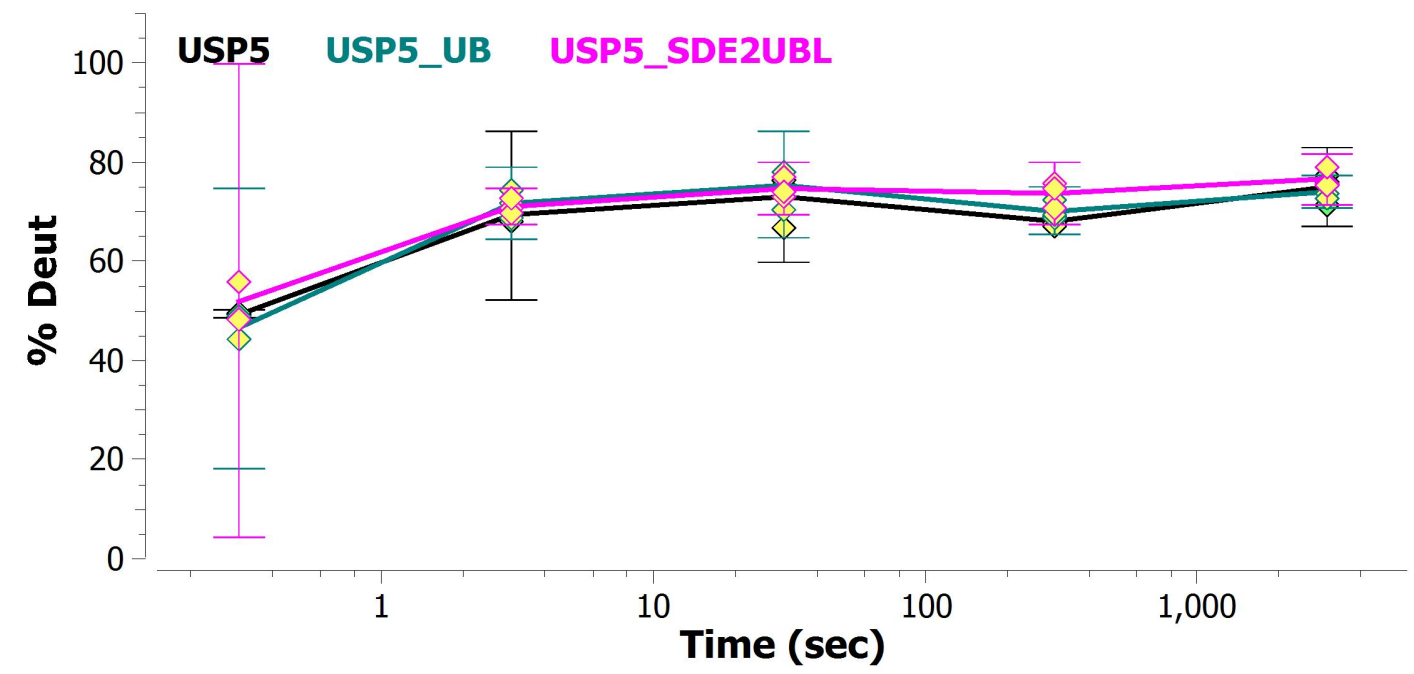

P45974 301-309: NQRIGEWEL (#73)

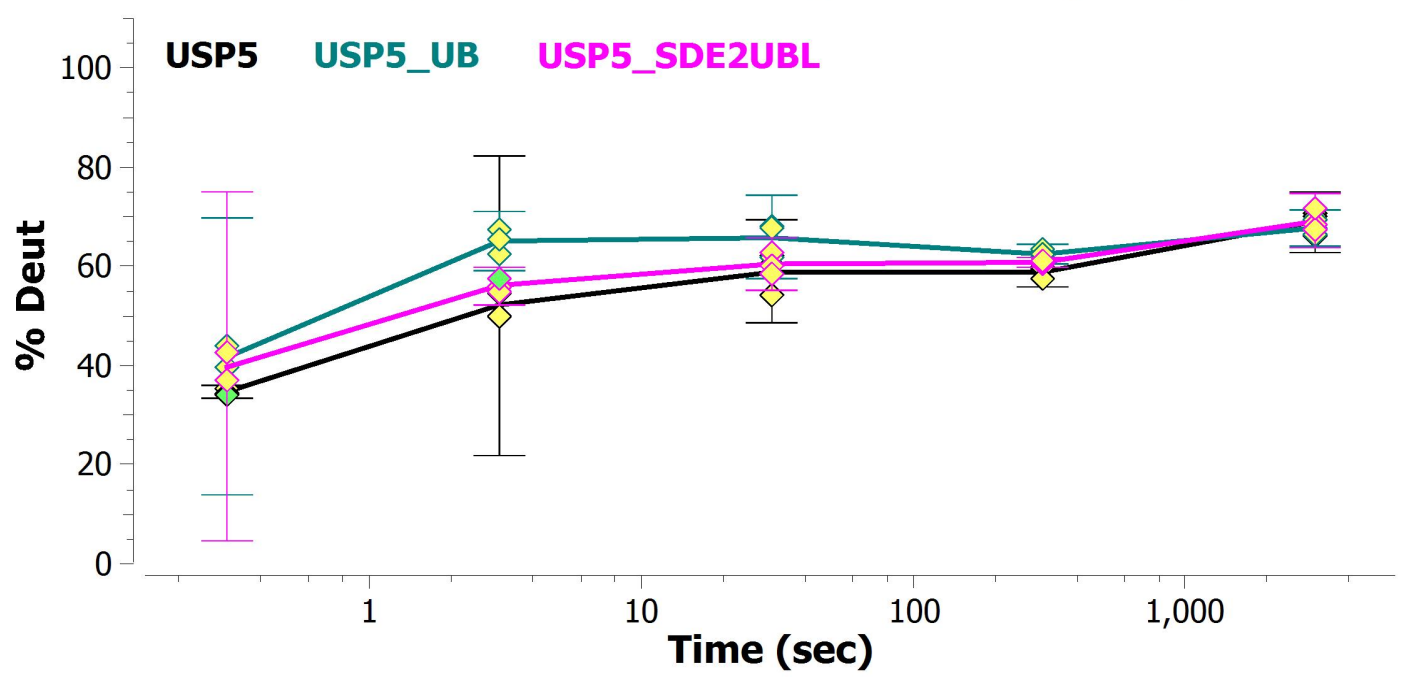

P45974 308-334: ELIQESGVPLKPLFGPGYTGIRNLGNS (#74)

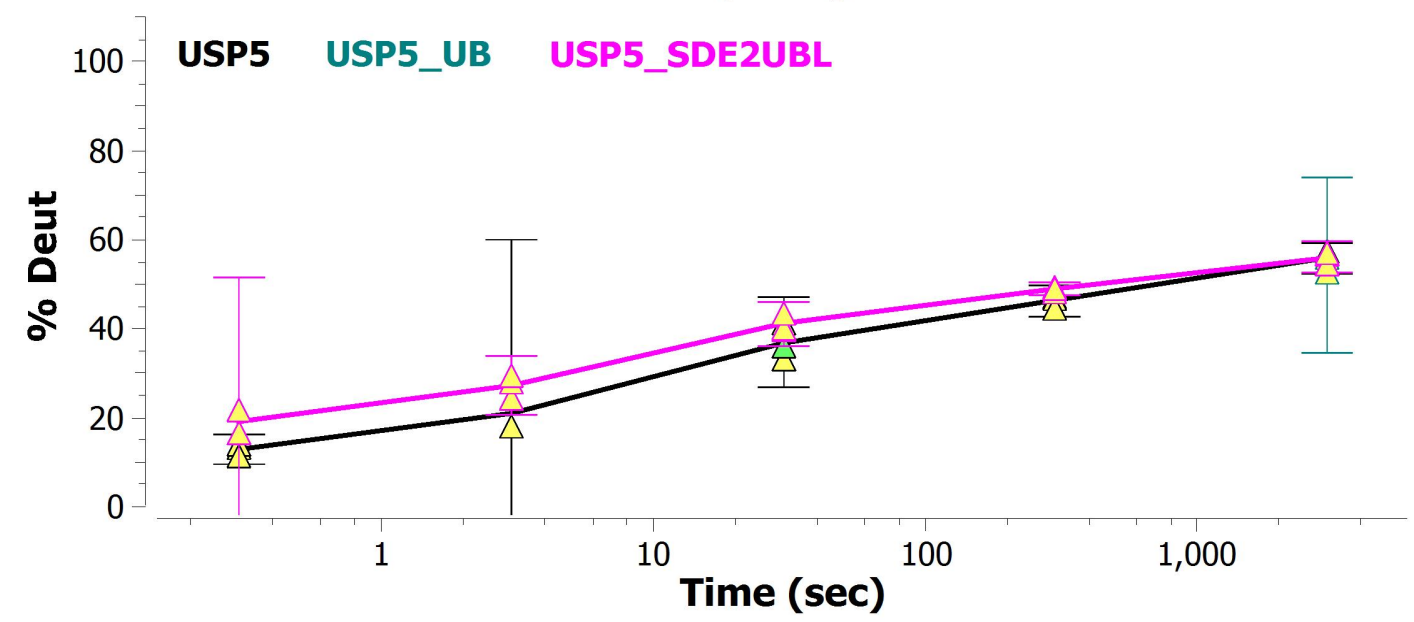

P45974 343-358: VLFSIPDFQRKYVDKL (#75)

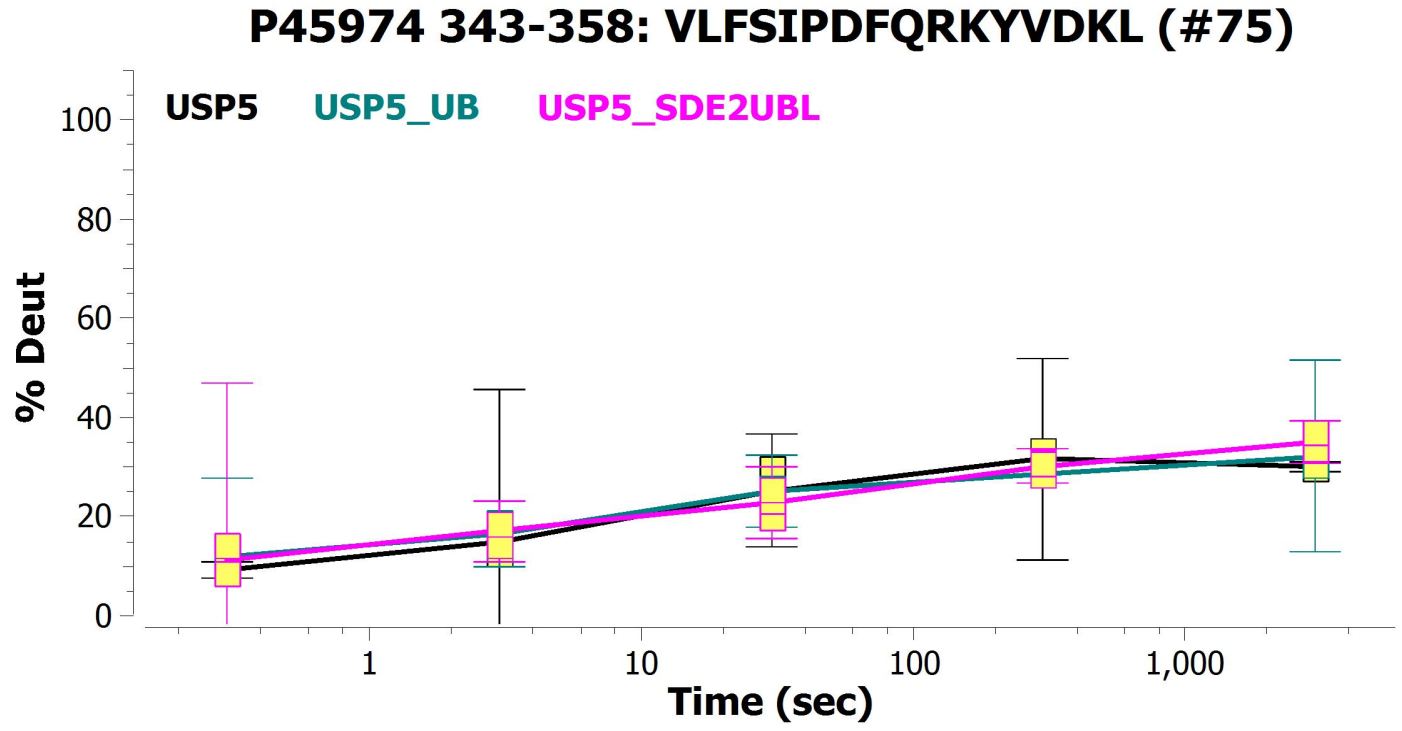

P45974 345-358: FSIPDFQRKYVDKL (#76)

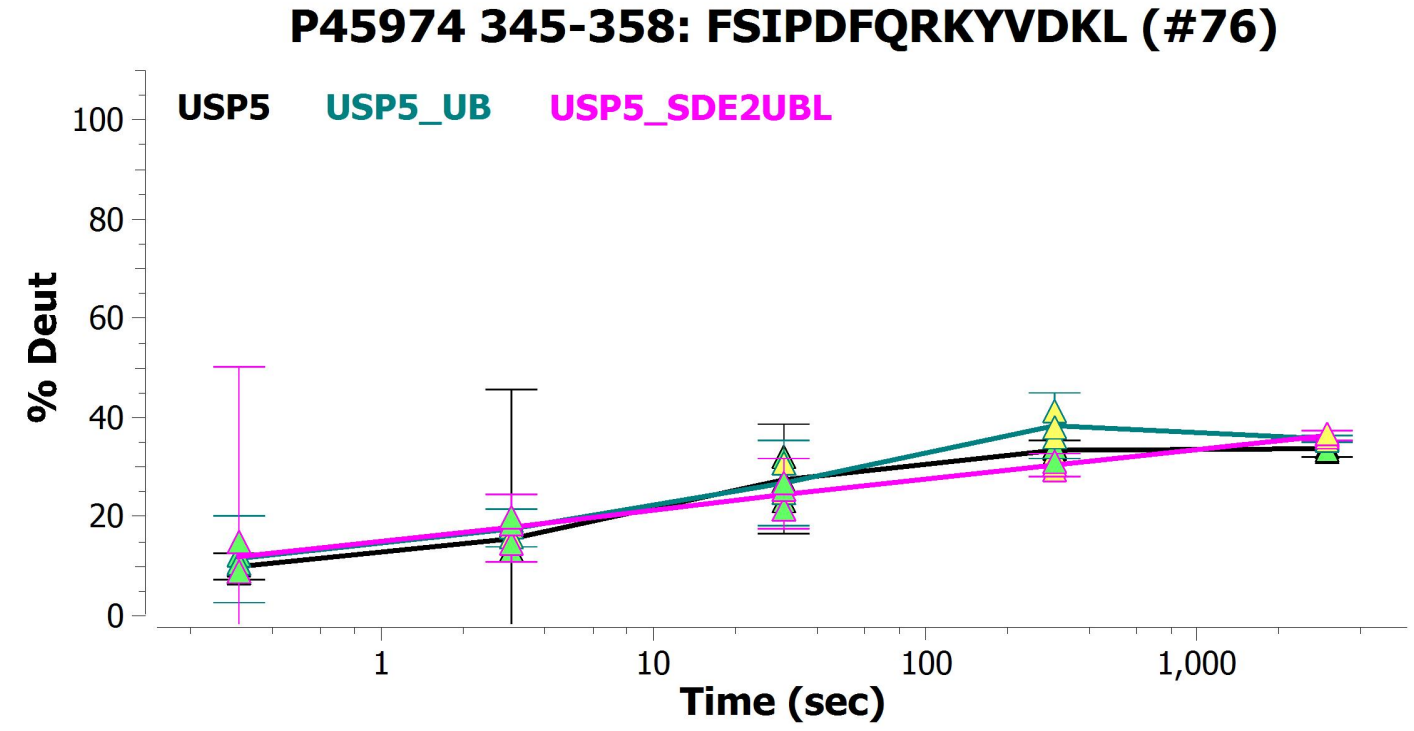

P45974 346-358: SIPDFQRKYVDKL (#77)

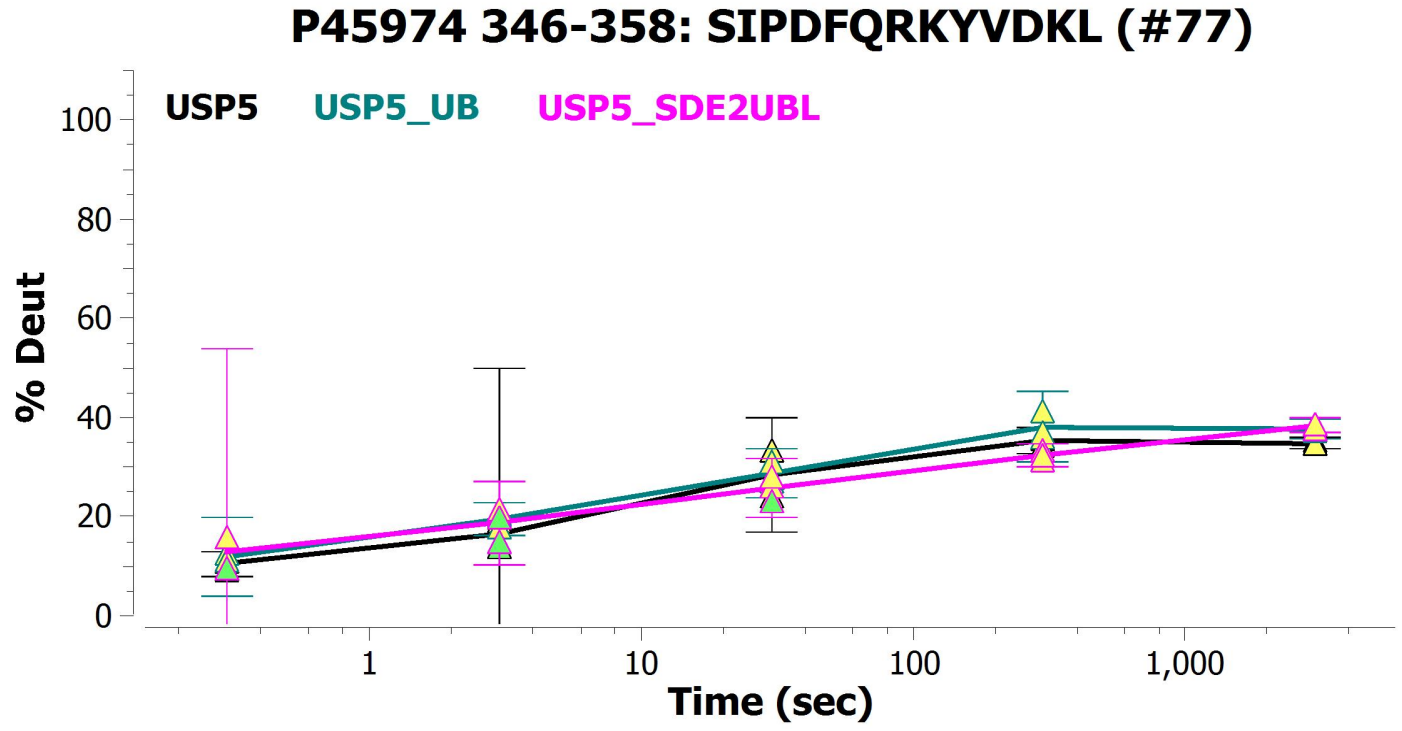

P45974 349-362: DFQRKYVDKLEKIF (#78)

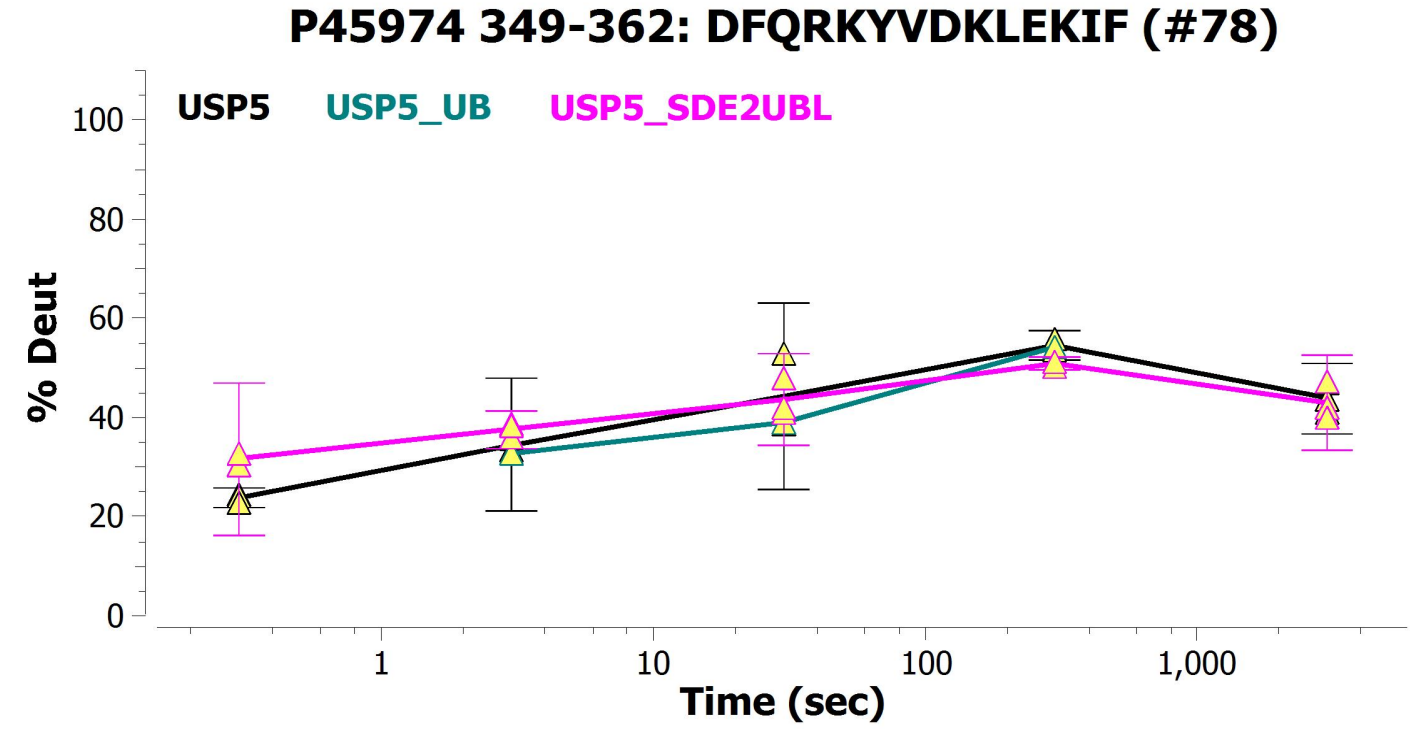

P45974 359-372: EKIFQNAPTDPTQD (#79)

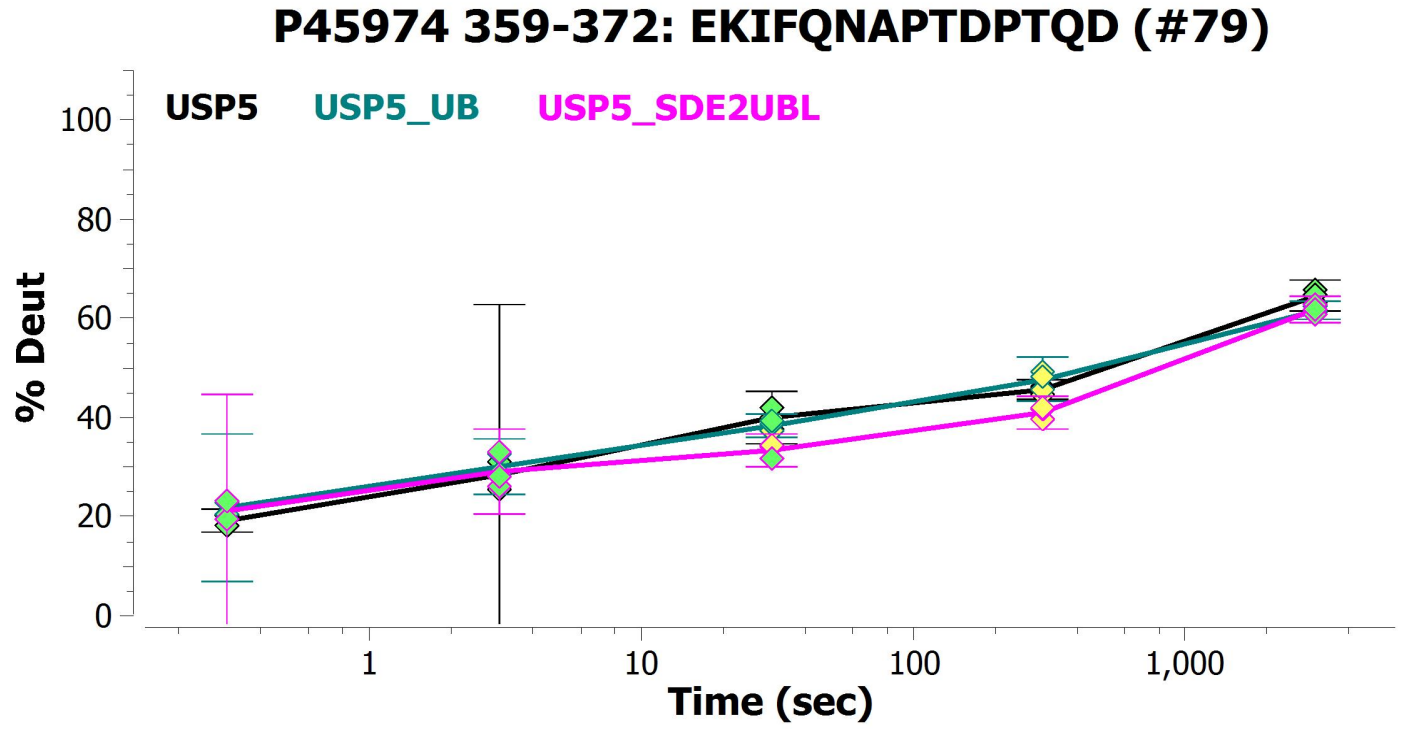

P45974 359-373: EKIFQNAPTDPTQDF (#80)

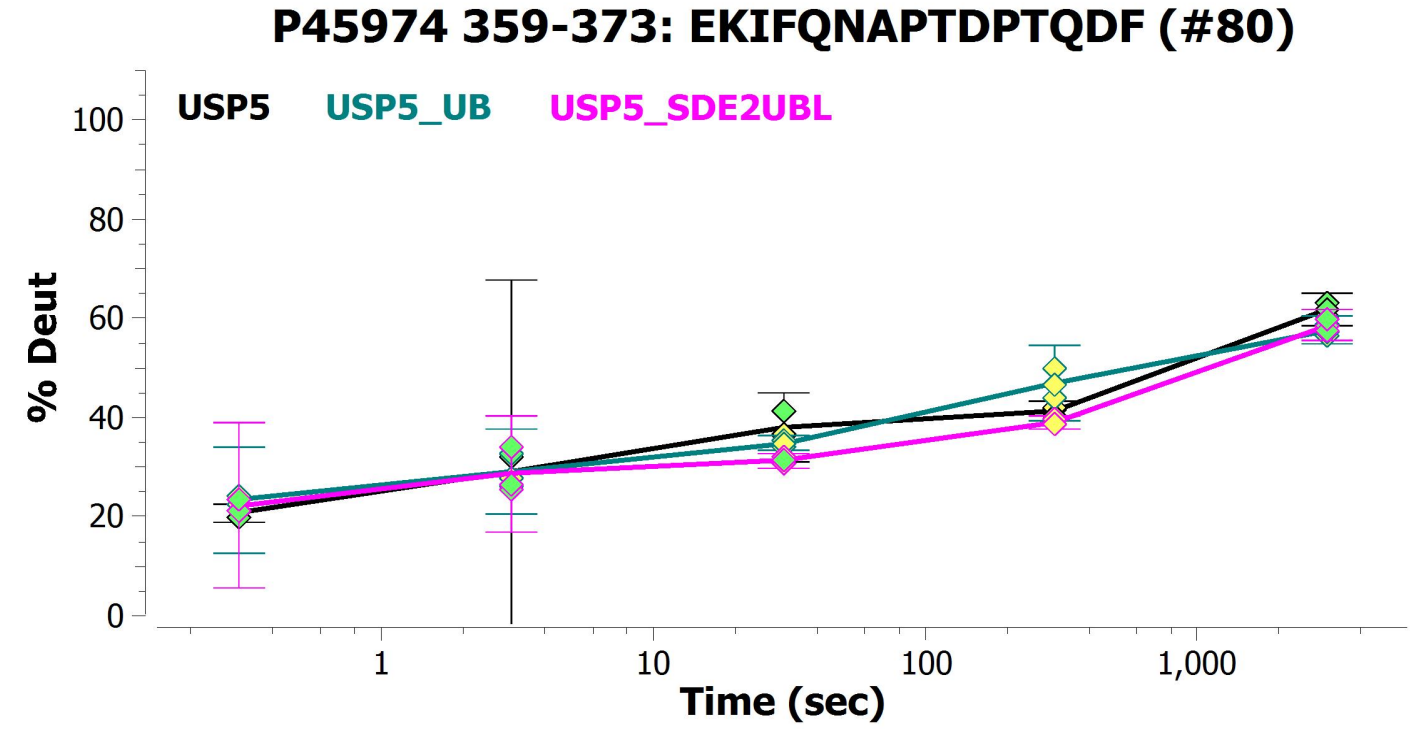

P45974 373-388: FSTQVAKLGHGLLSGE (#81)

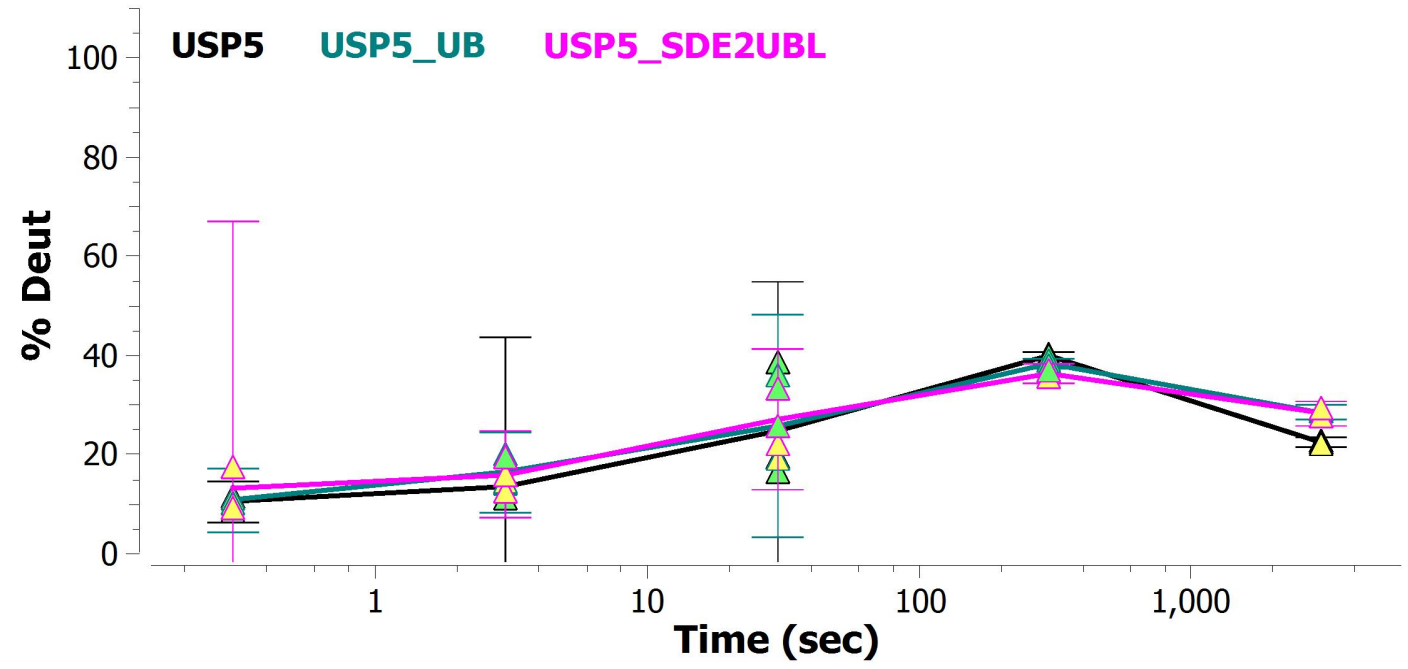

P45974 374-388: STQVAKLGHGLLSGE (#82)

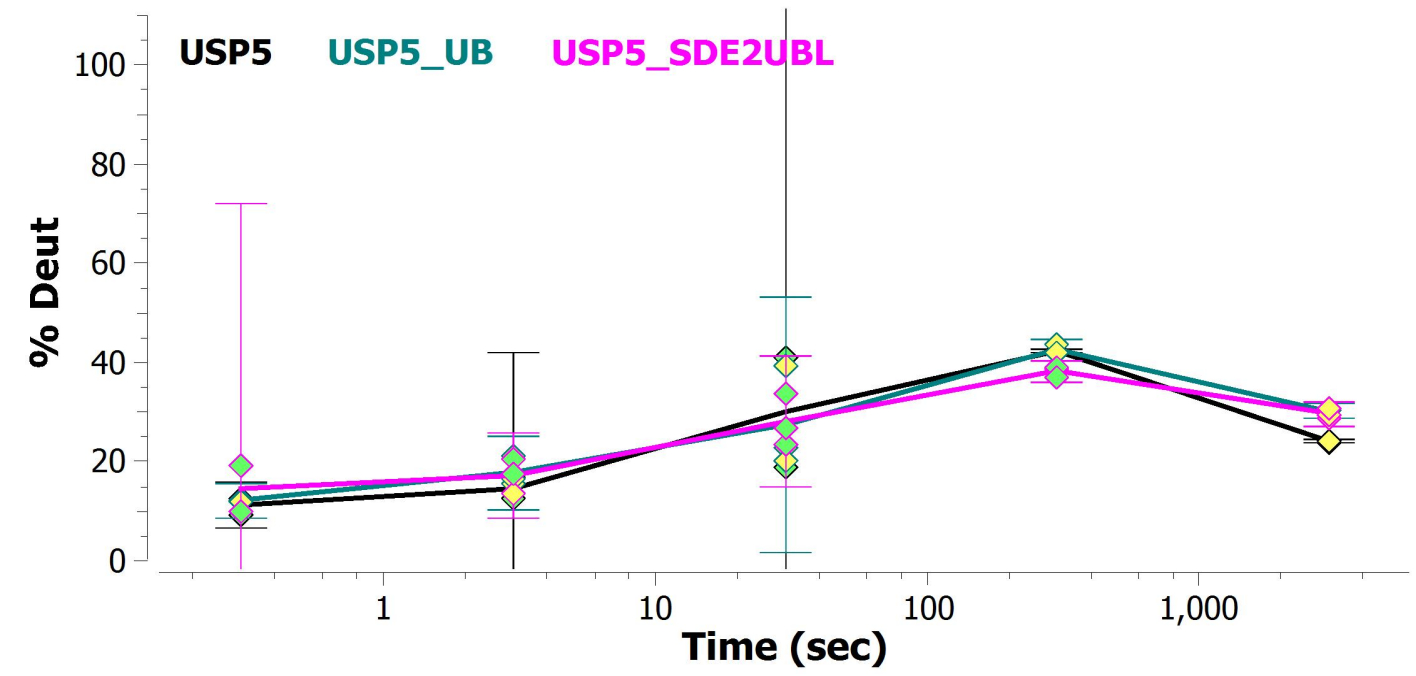

P45974 378-388: AKLGHGLLSGE (#83)

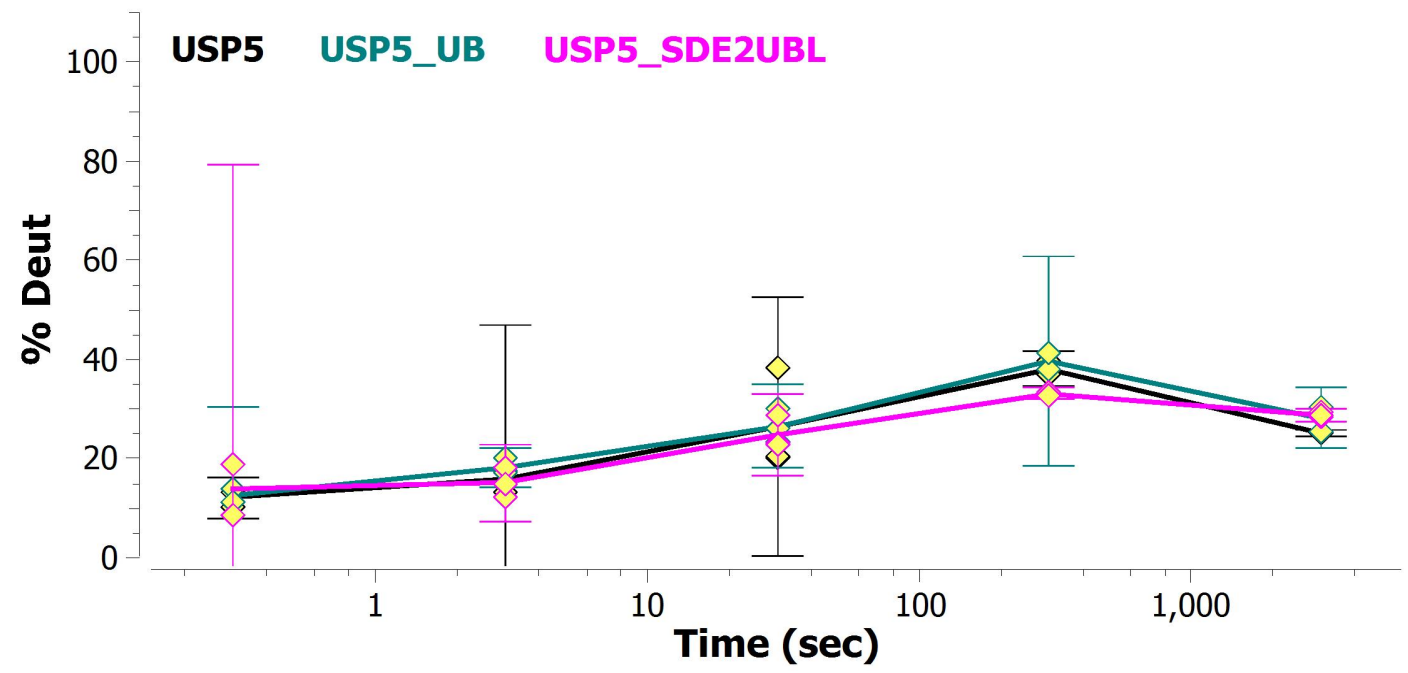

P45974 382-412: HGLLSGEYSKPVPESGDGERVPEQKEVQDGI (#84)

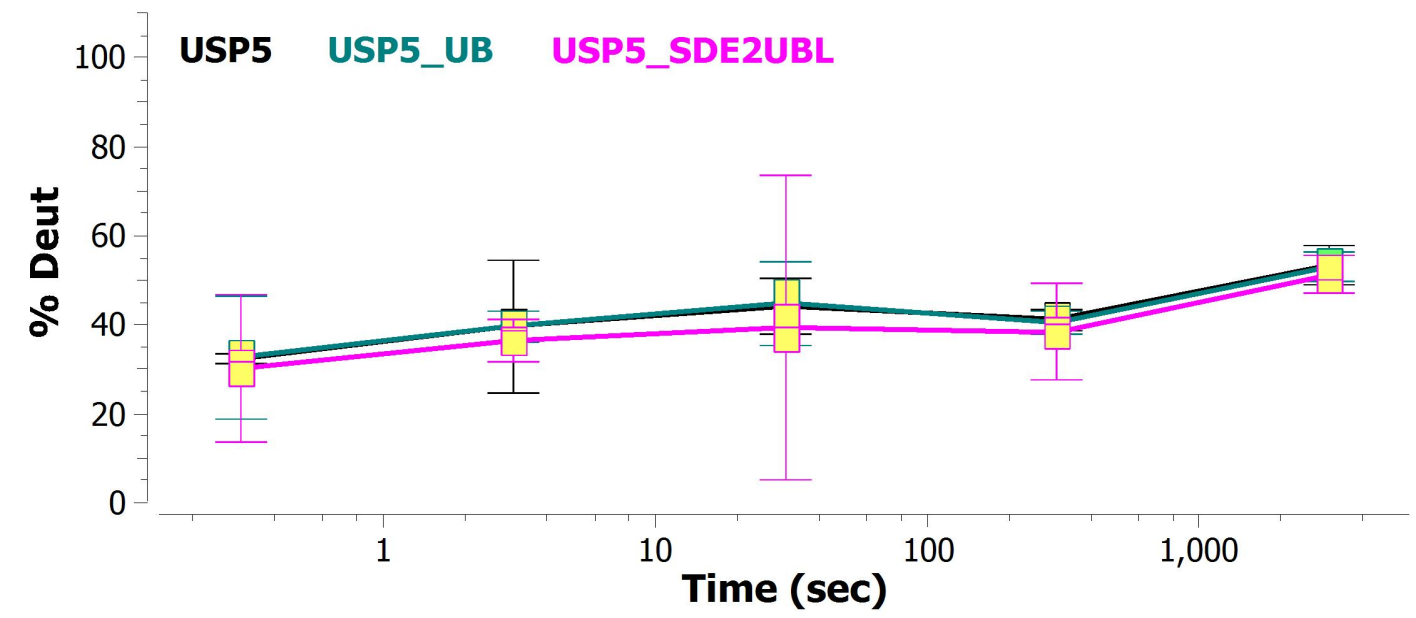

P45974 389-413: YSKPVPESGDGERVPEQKEVQDGIA (#85)

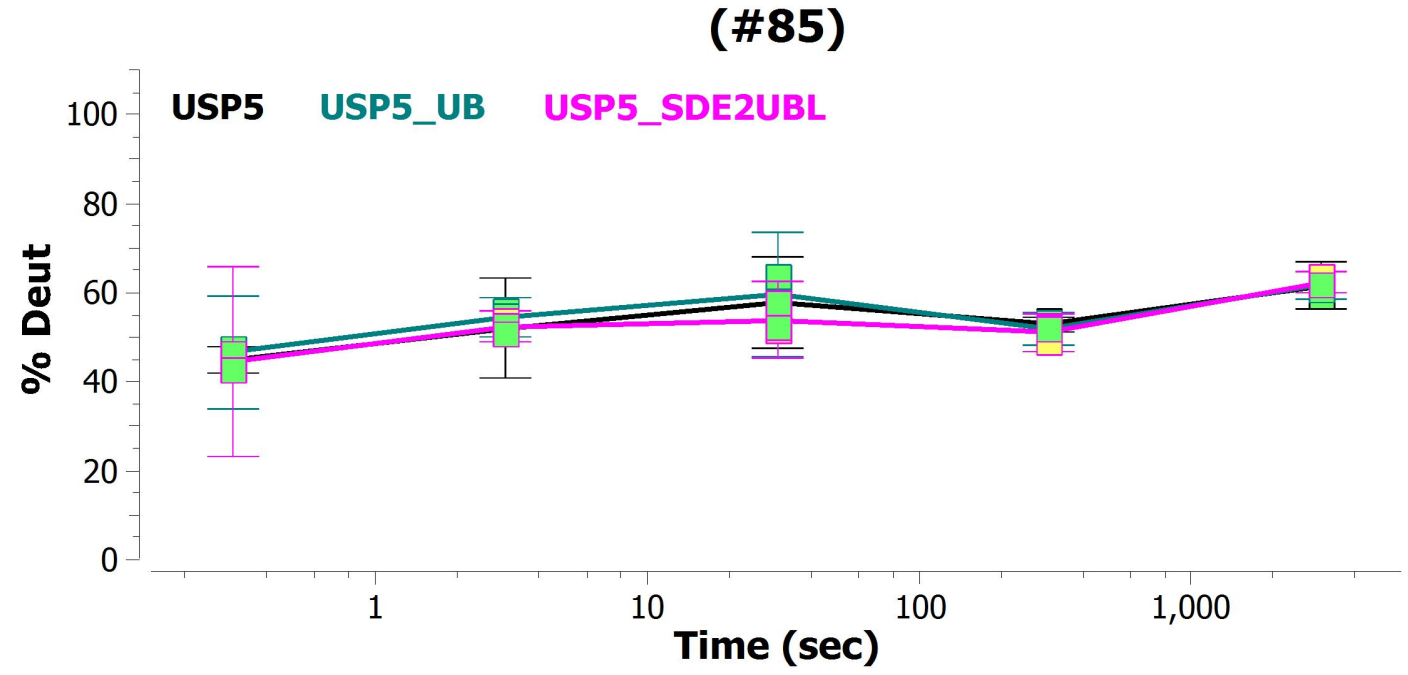

P45974 389-416: YSKPVPESGDGERVPEQKEVQDGIAPRM (#86)

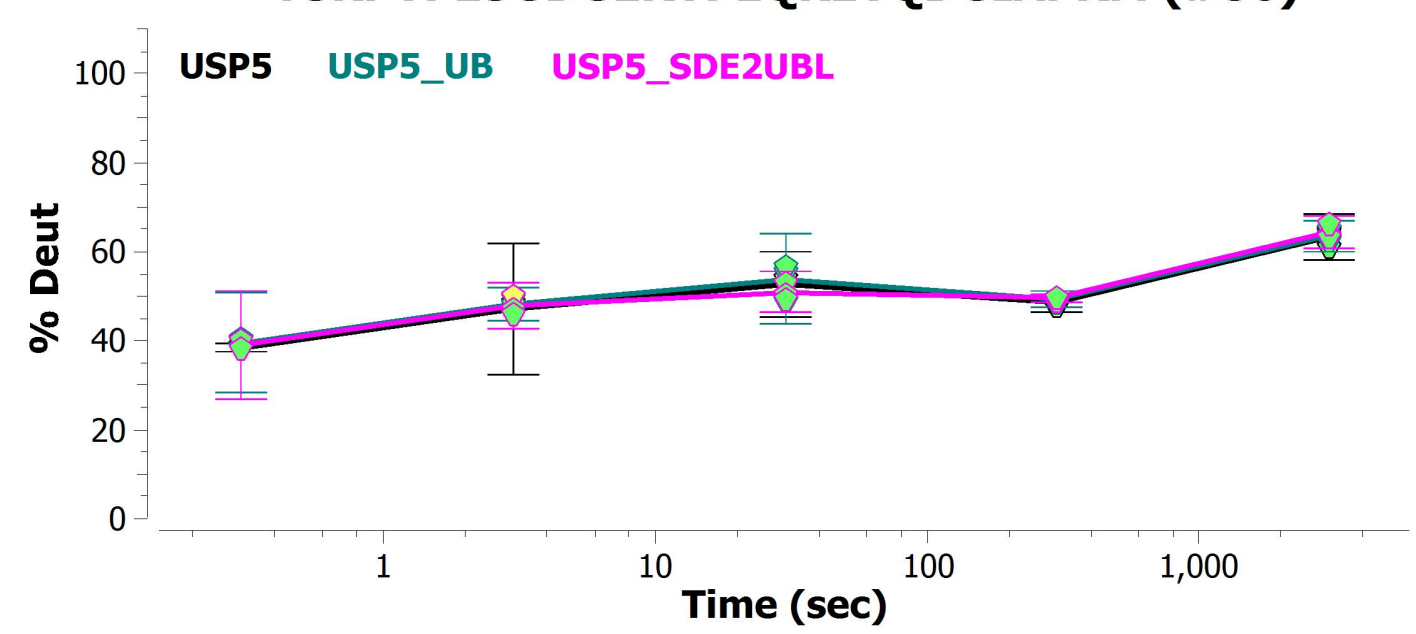

P45974 414-428: PRMFKALIGKGHPEF (#87)

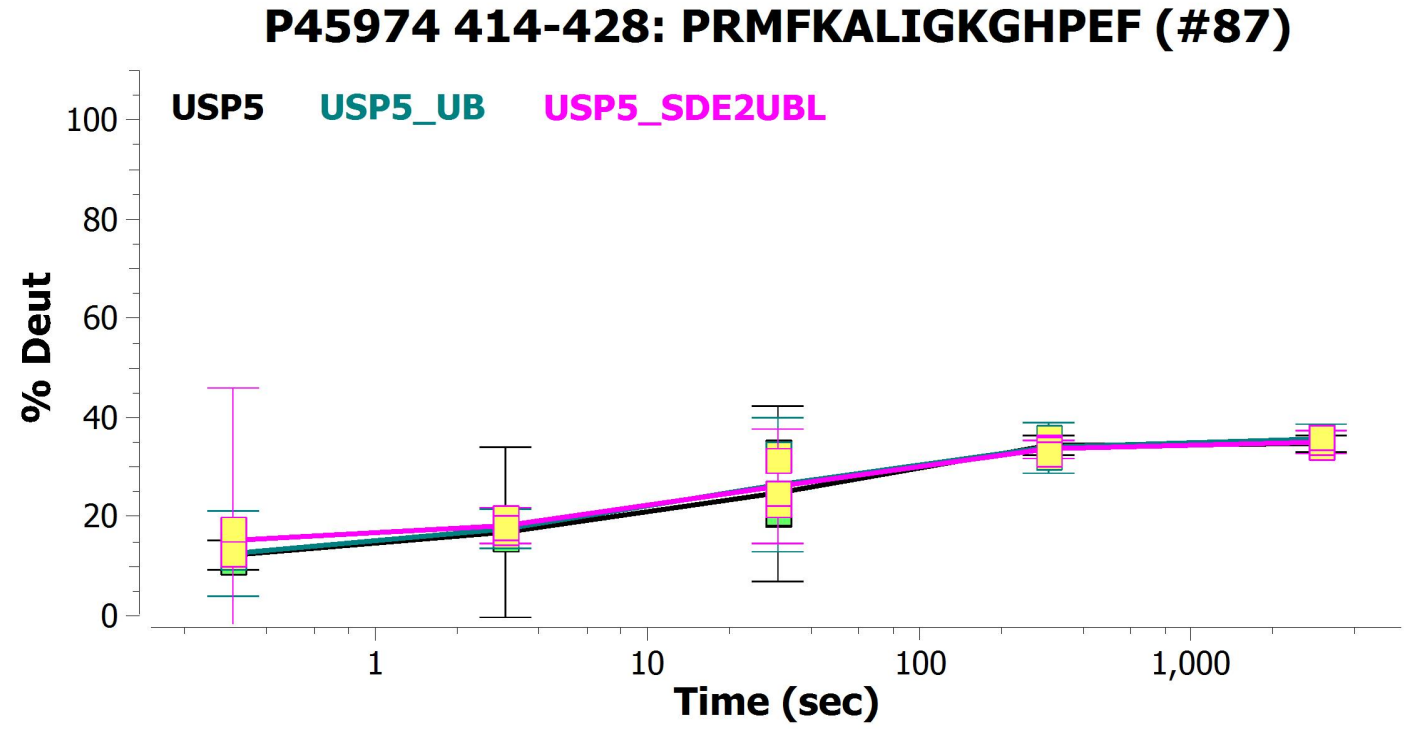

P45974 417-428: FKALIGKGHPEF (#88)

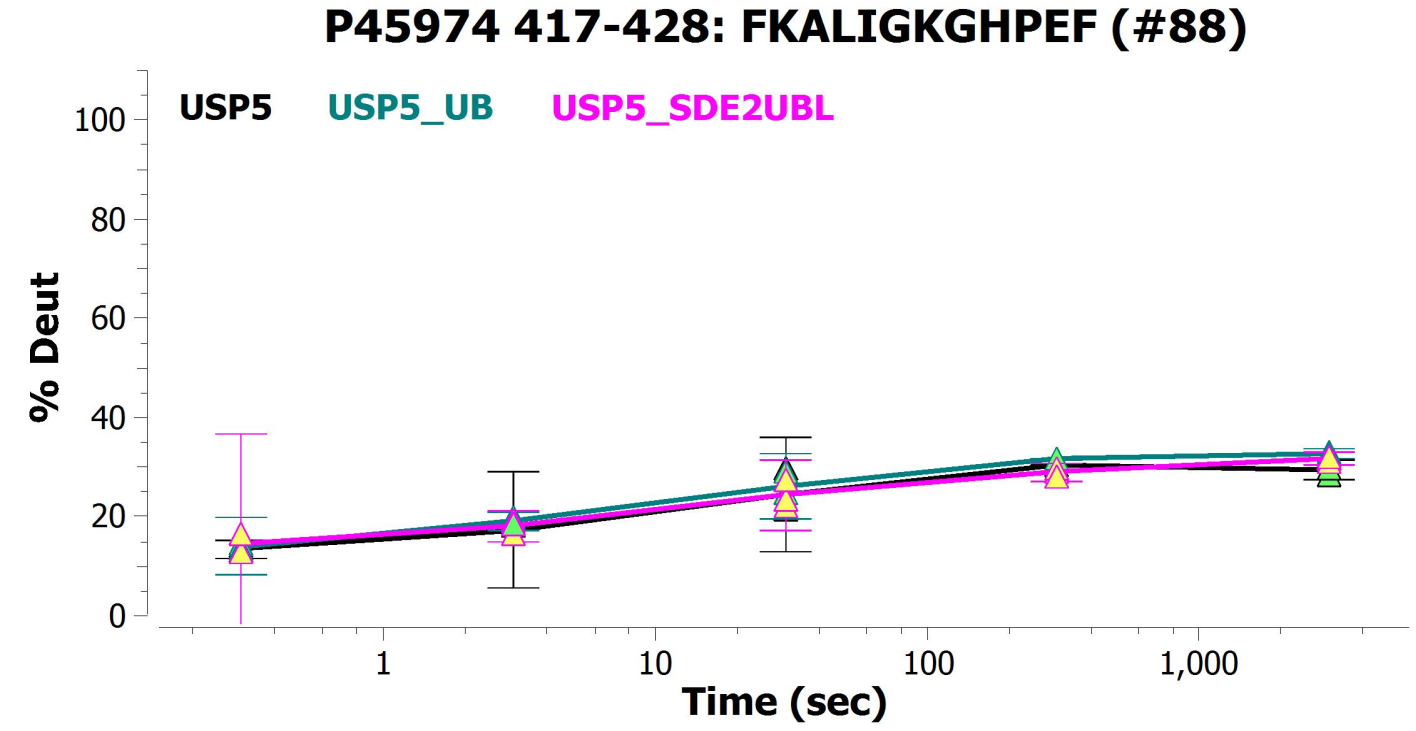

P45974 417-438: FKALIGKGHPEFSTNRQQDAQE (#89)

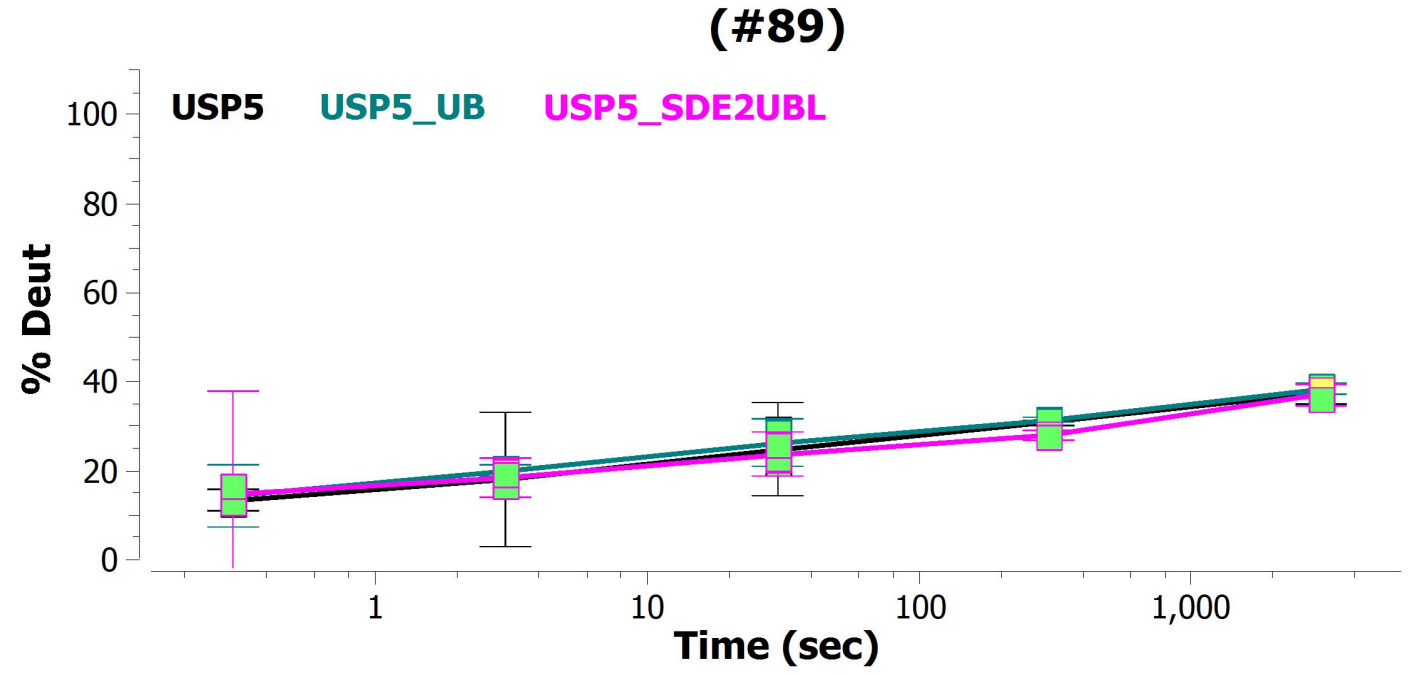

P45974 417-439: FKALIGKGHPEFSTNRQQDAQEF (#90)

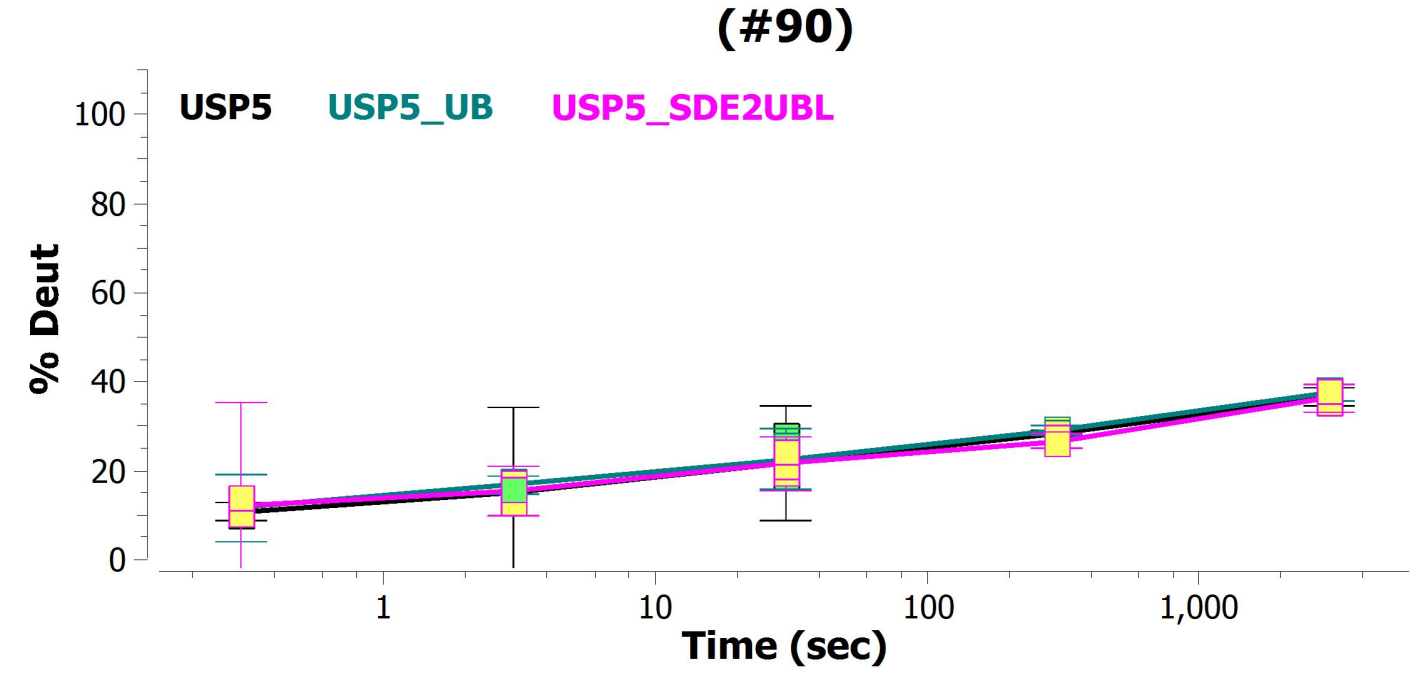

P45974 417-440: FKALIGKGHPEFSTNRQQDAQEFF (#91)

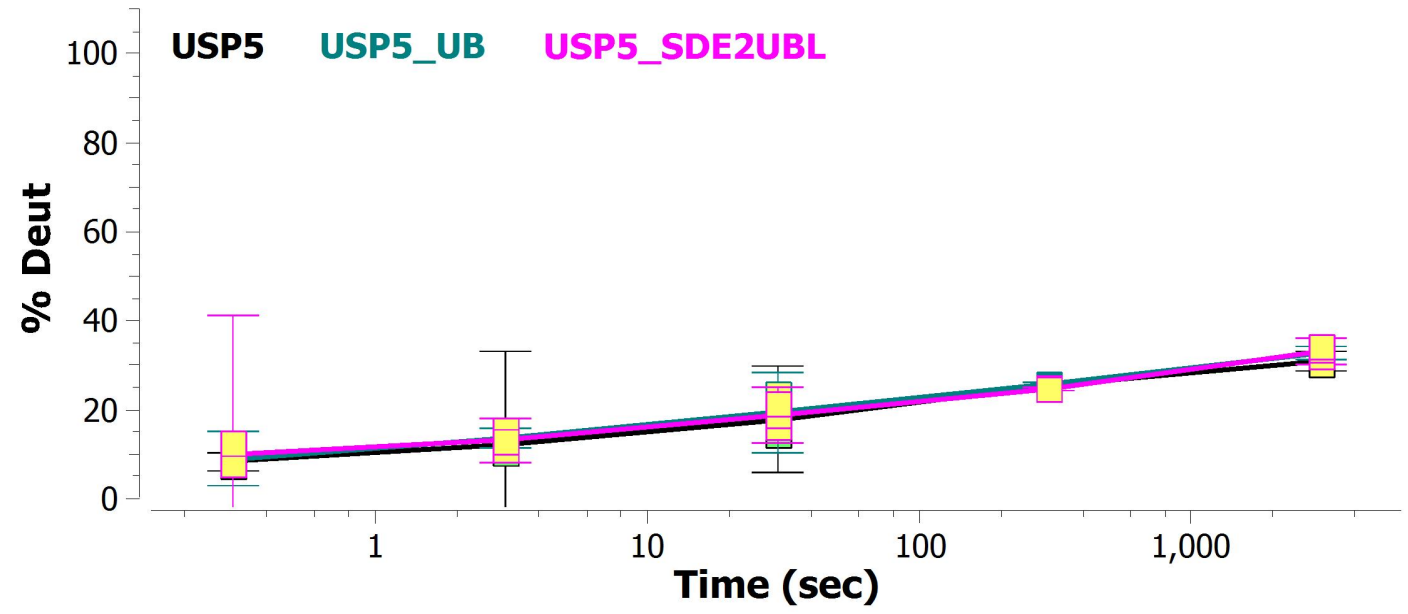

P45974 418-428: KALIGKGHPEF (#92)

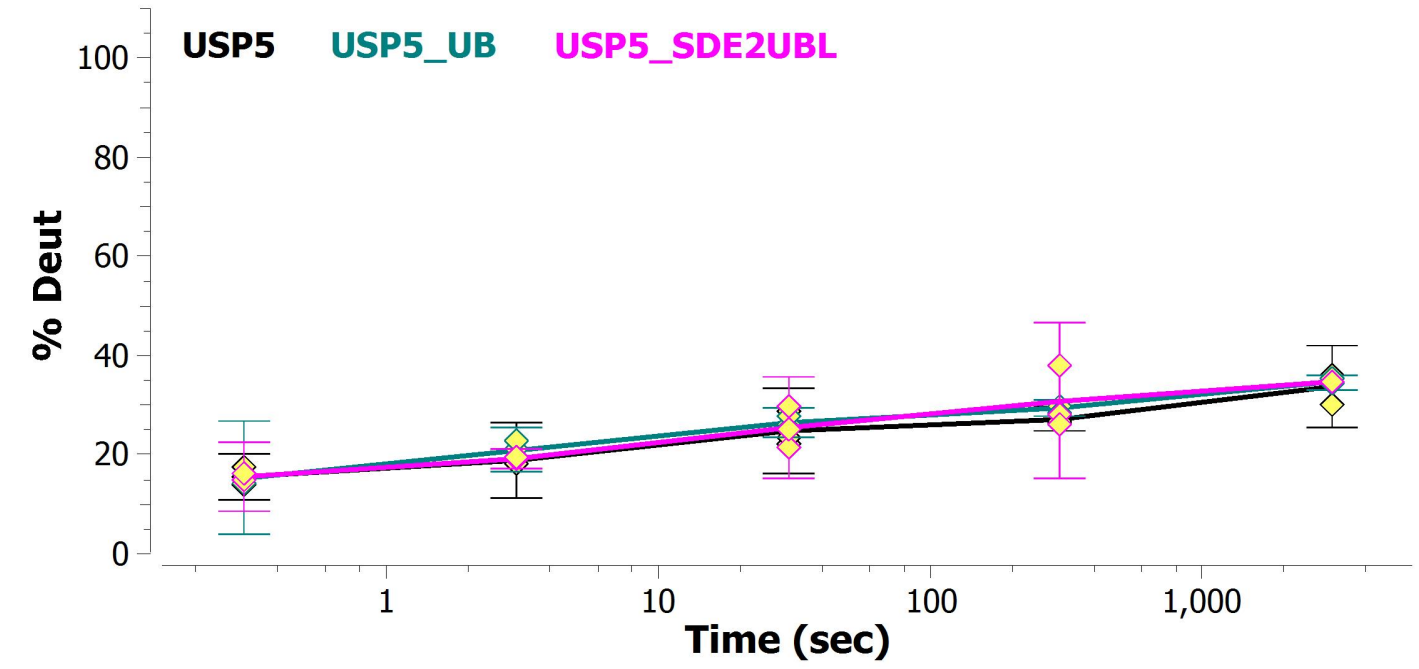

P45974 429-438: STNRQQDAQE (#93)

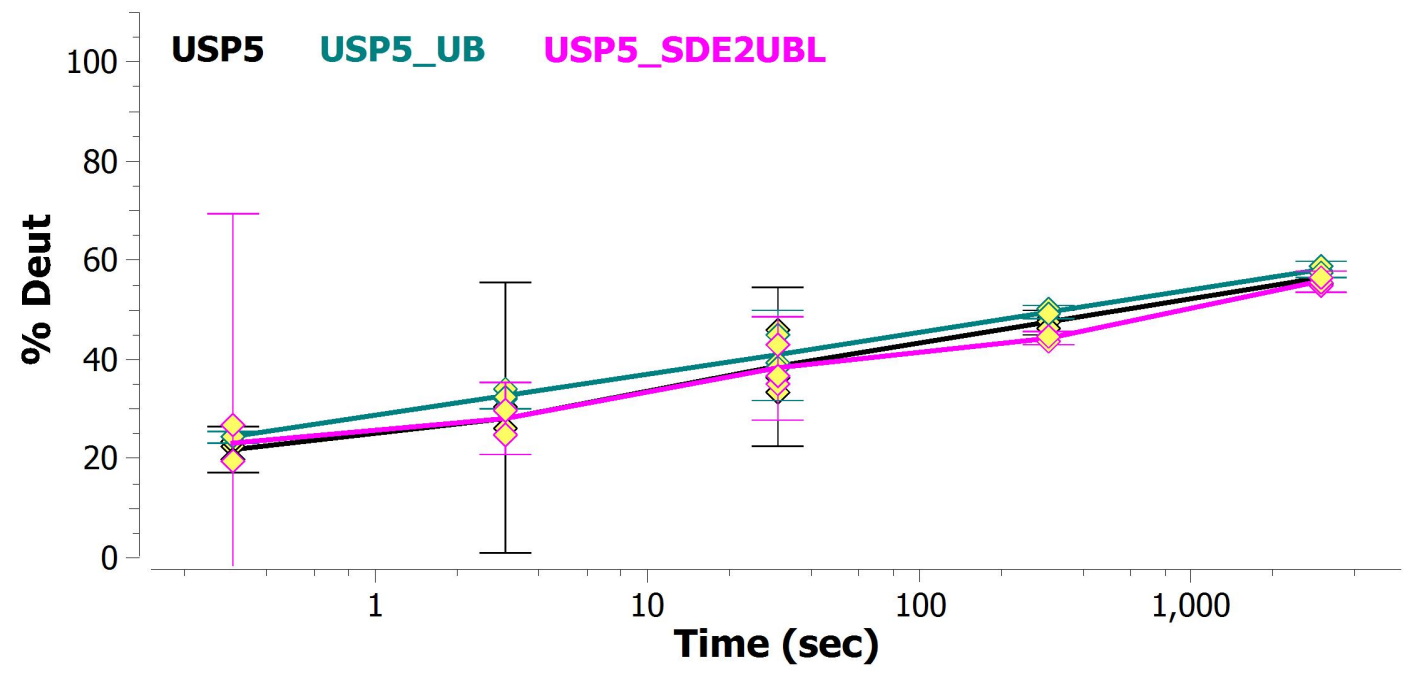

P45974 429-439: STNRQQDAQEF (#94)

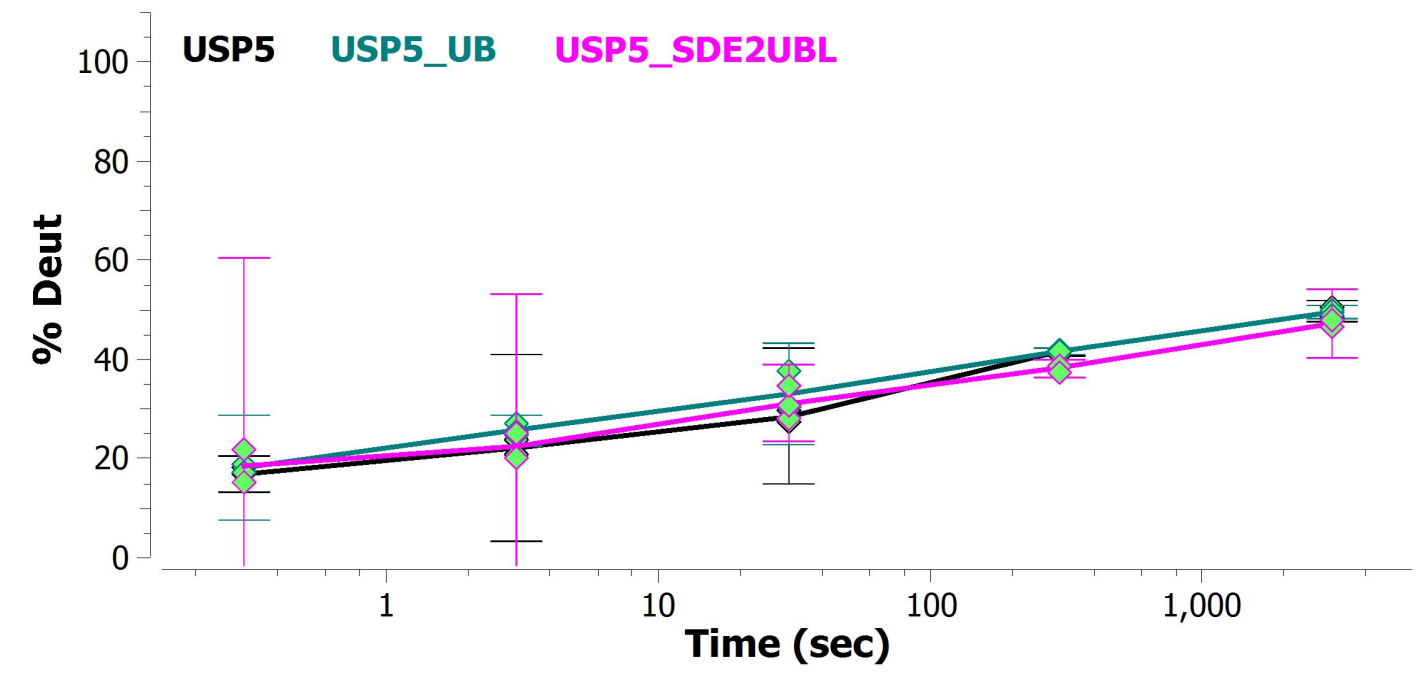

P45974 429-440: STNRQQDAQEFF (#95)

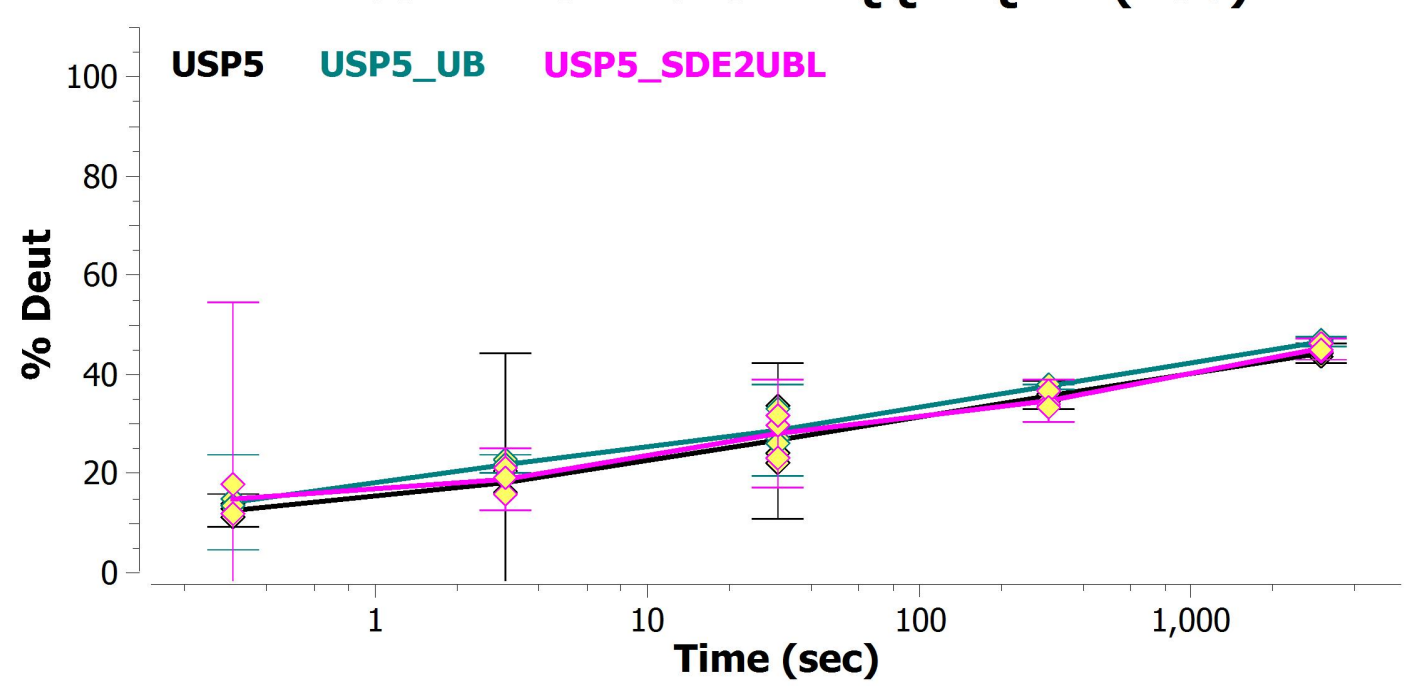

P45974 434-439: QDAQEF (#96)

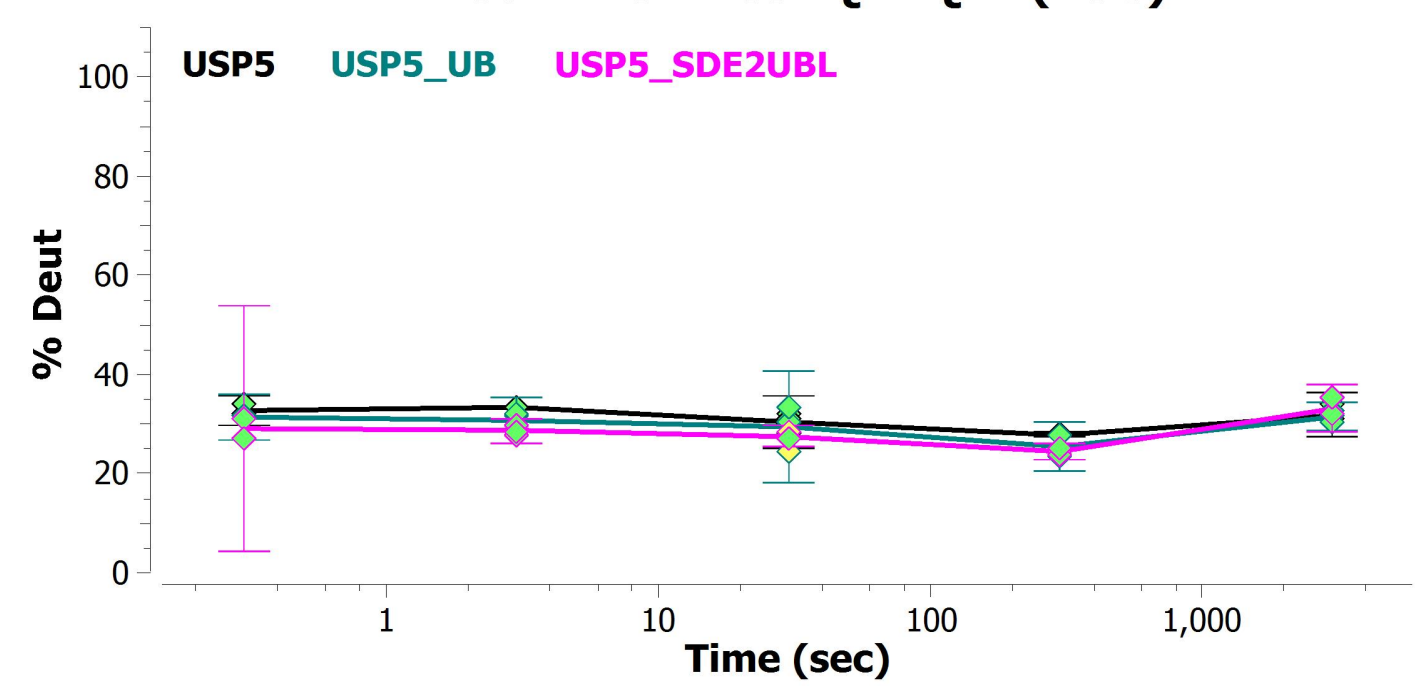

P45974 439-445: FFLHLIN (#97)

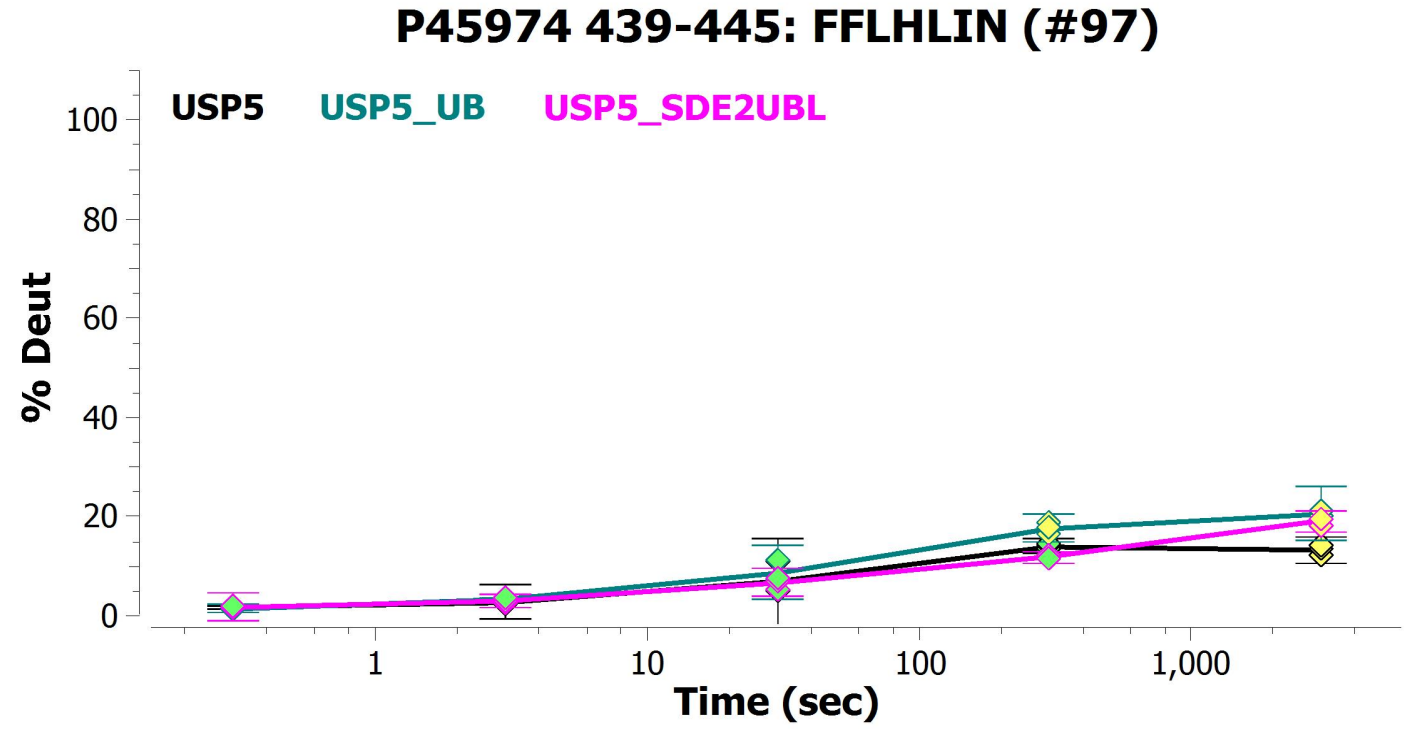

P45974 440-446: FLHLINM (#98)

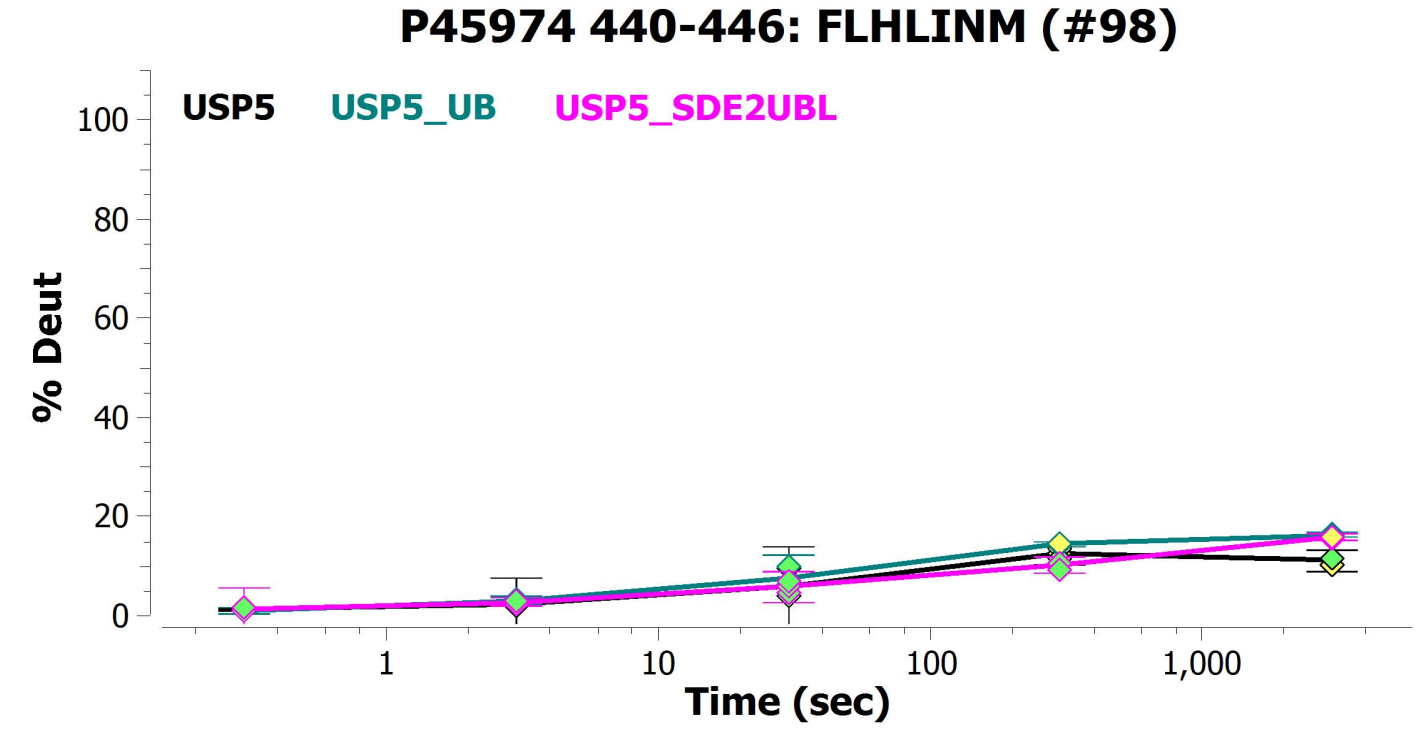

P45974 446-461: MVERNCRSSENPNEVF (#99)

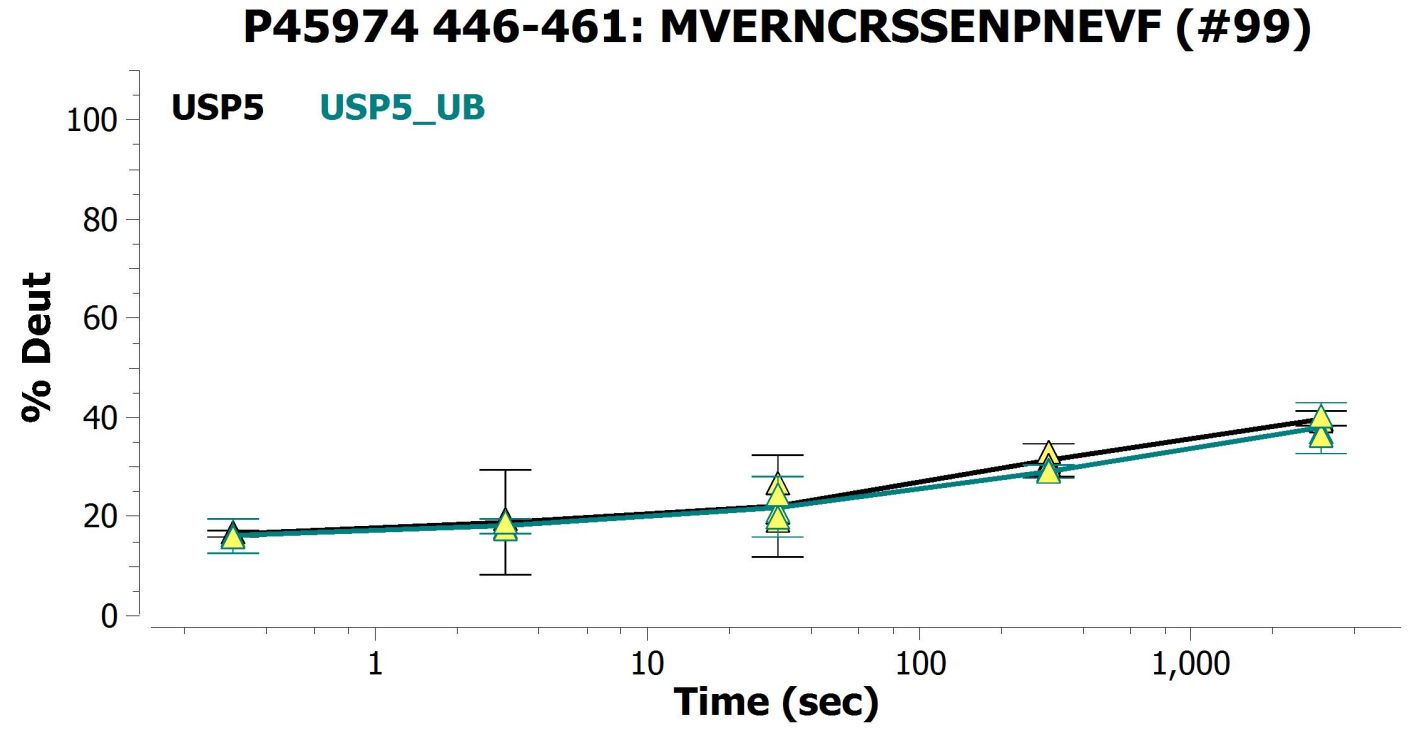

P45974 447-459: VERNCRSSENPNE (#100)

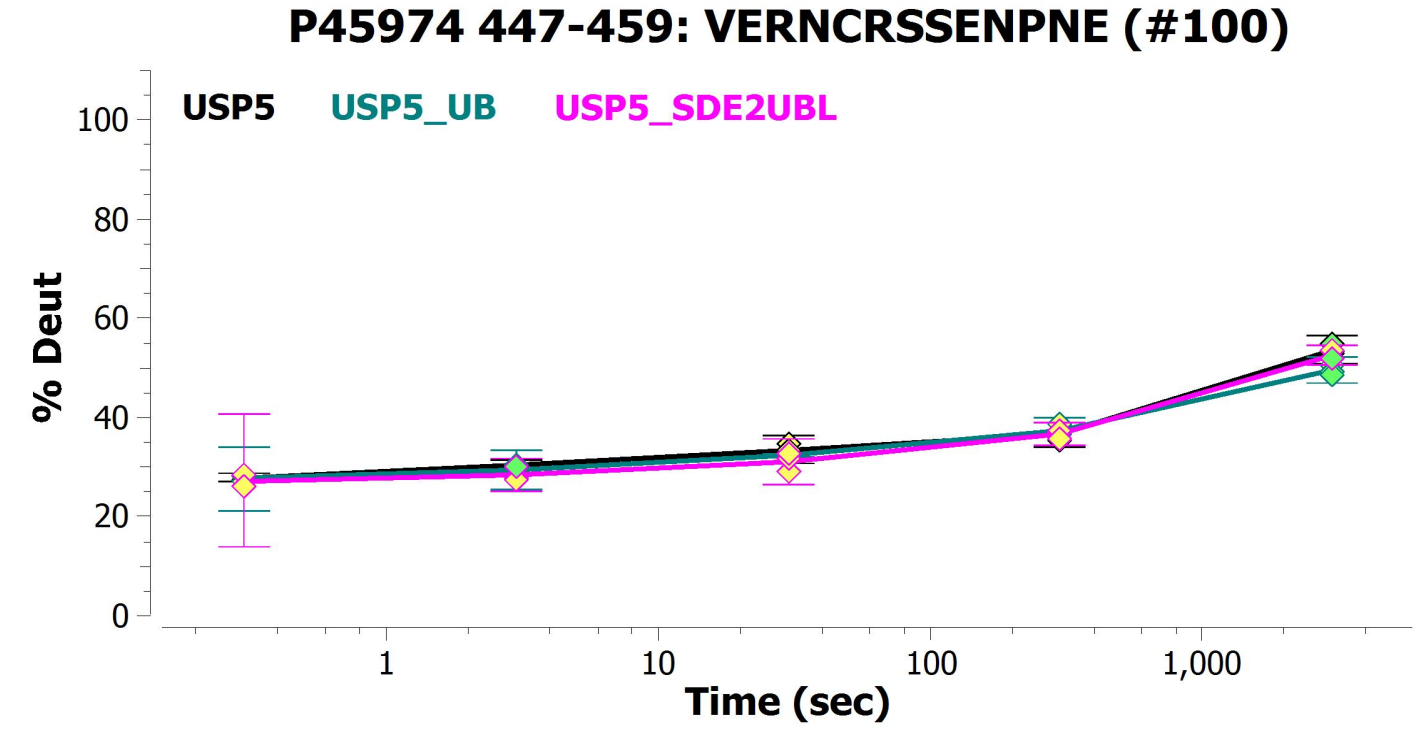

P45974 447-461: VERNCRSSENPNEVF (#101)

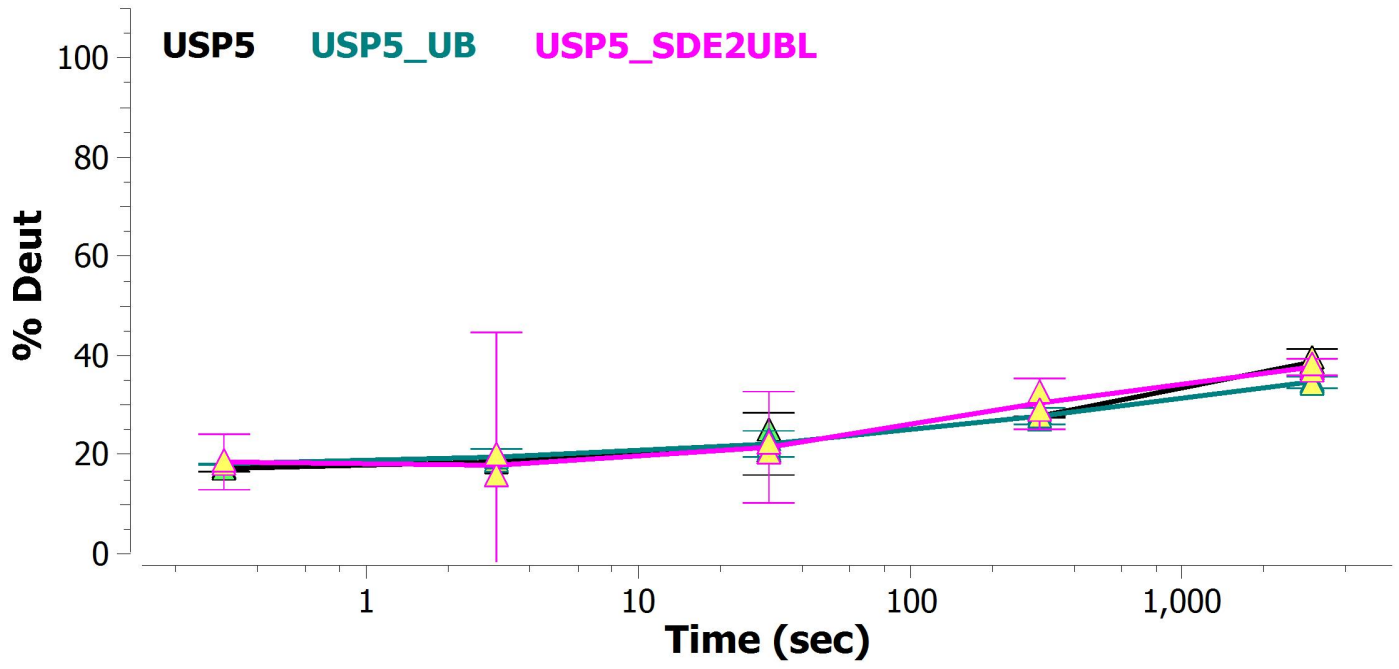

P45974 447-463: VERNCRSSENPNEVFRF (#102)

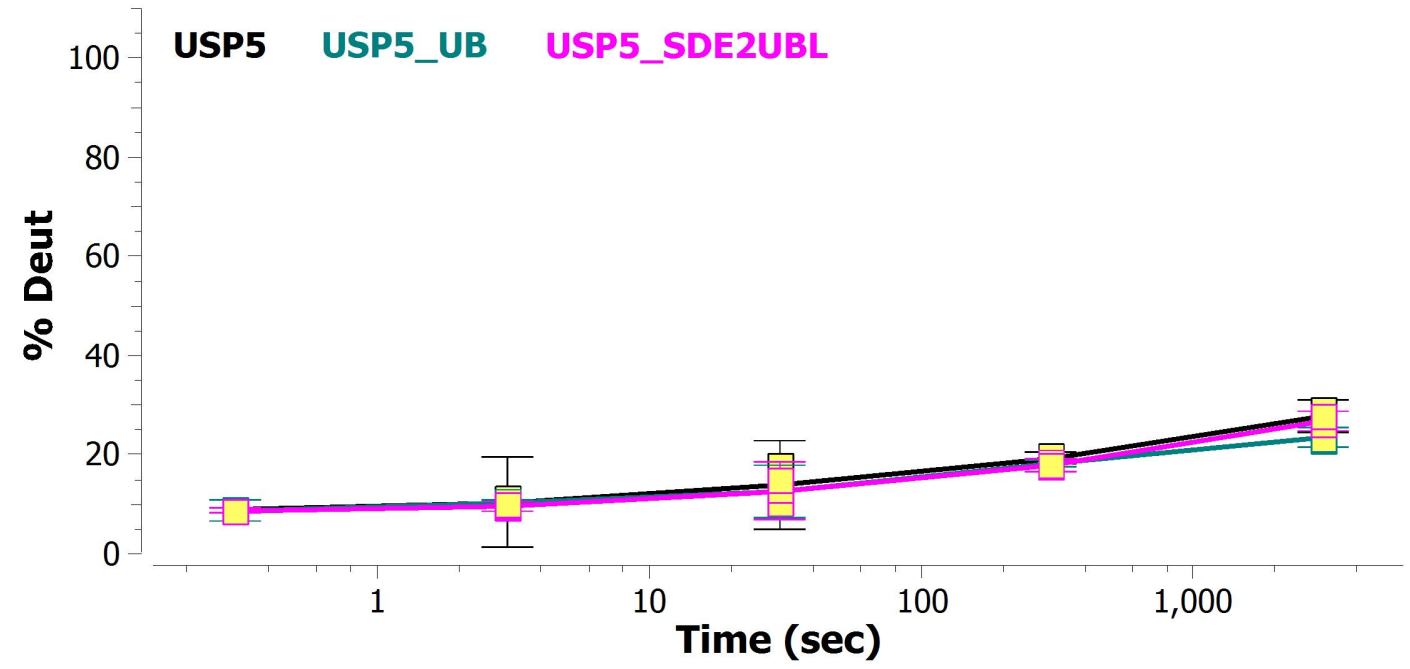

P45974 464-475: LVEEKIKCLATE (#103)

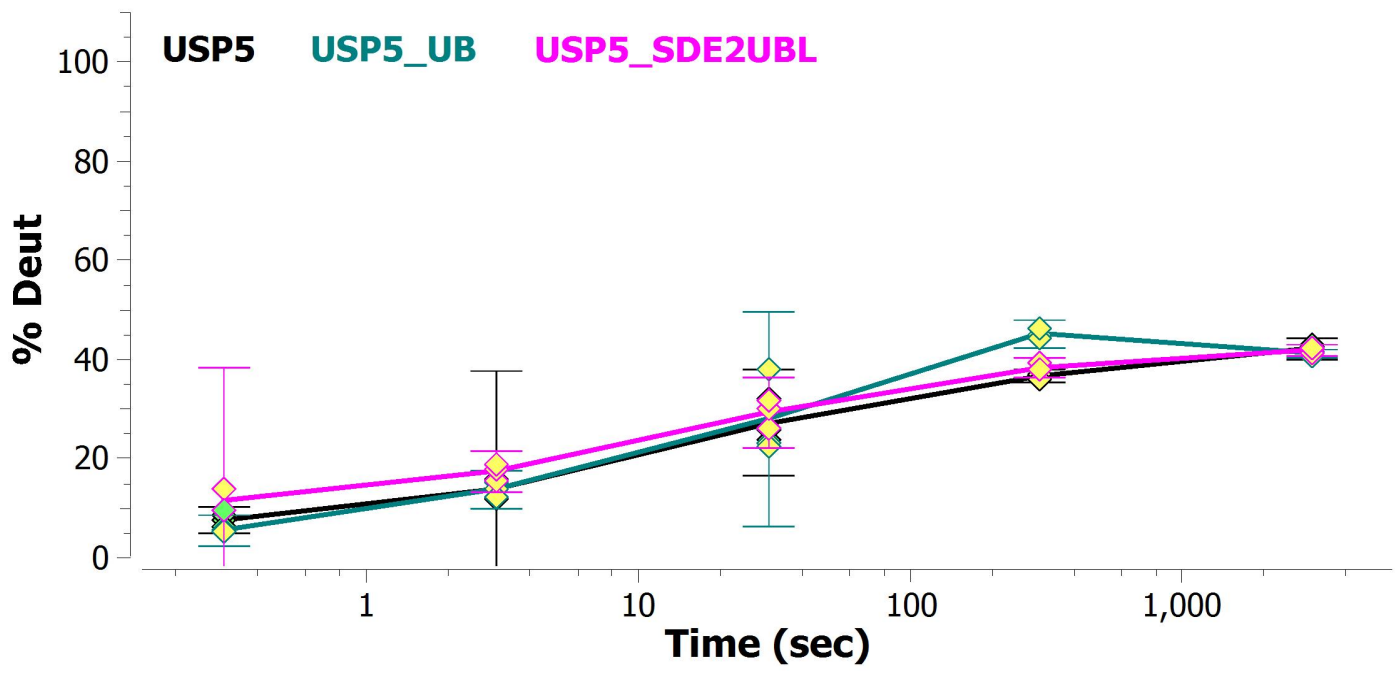

P45974 464-485: LVEEKIKCLATEKVKYTQRVDY (#104)

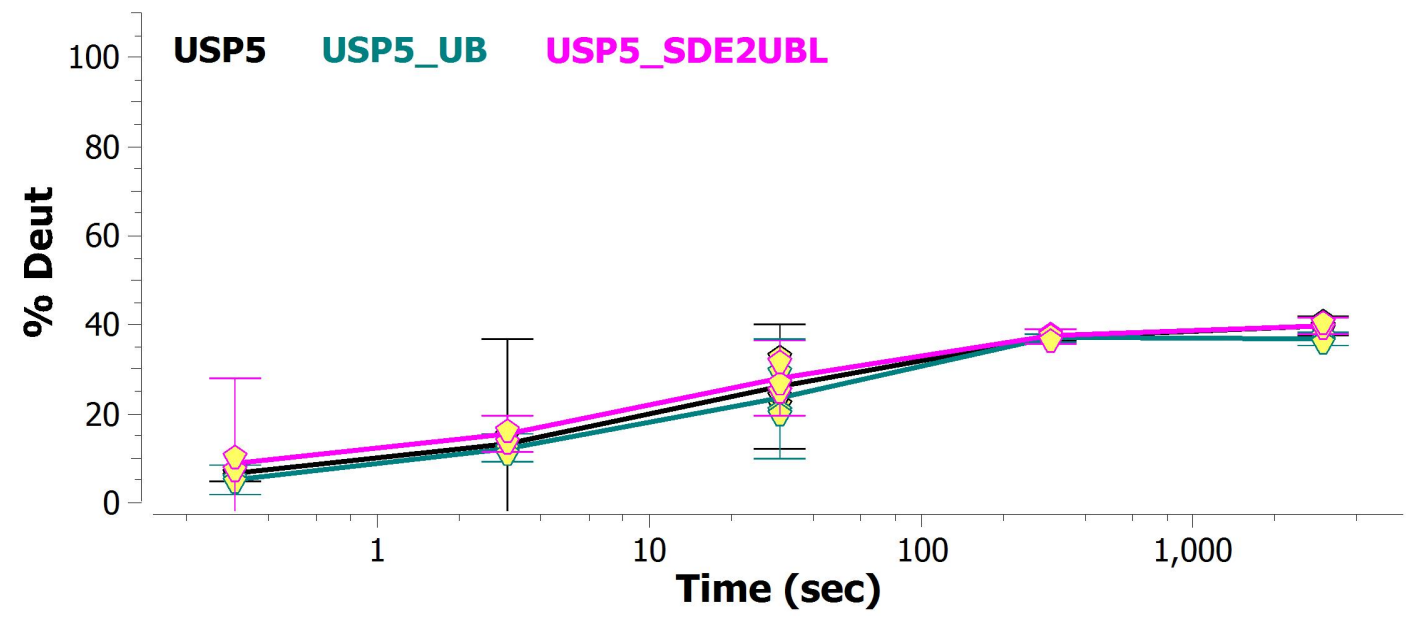

P45974 465-472: VEEKIKCL (#105)

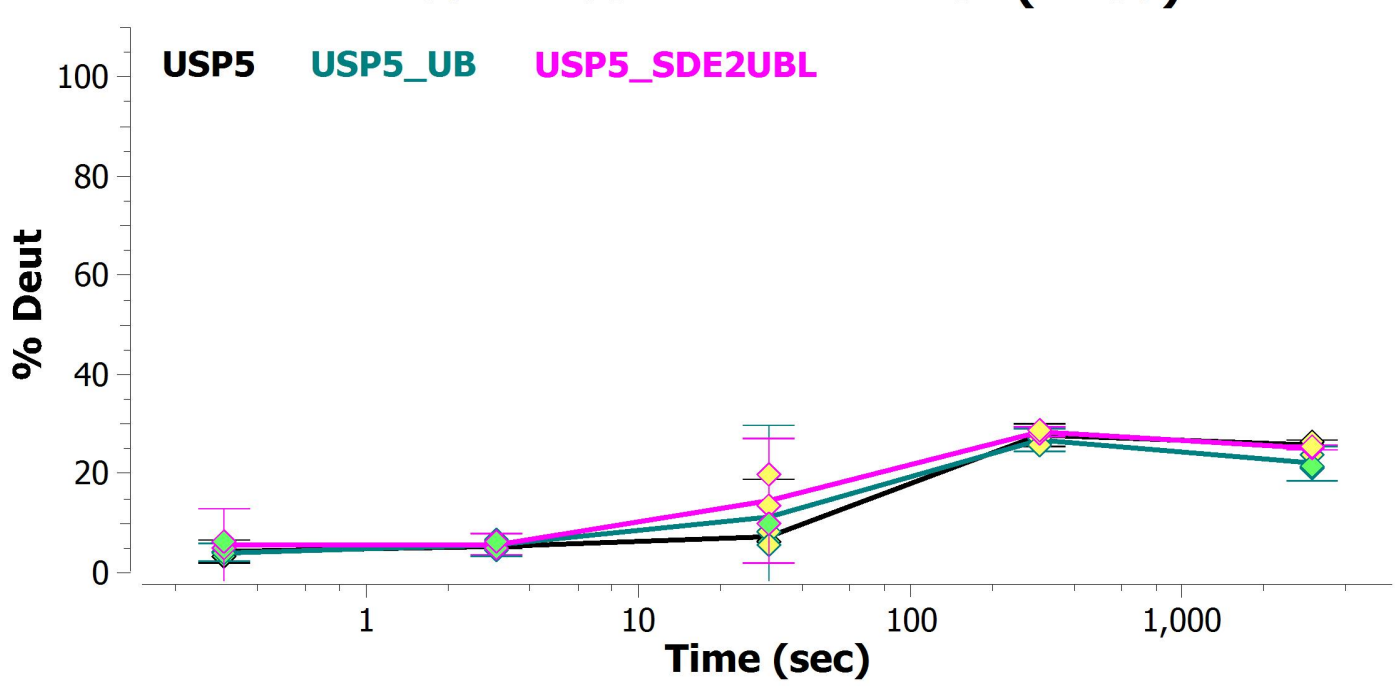

P45974 467-472: EKIKCL (#106)

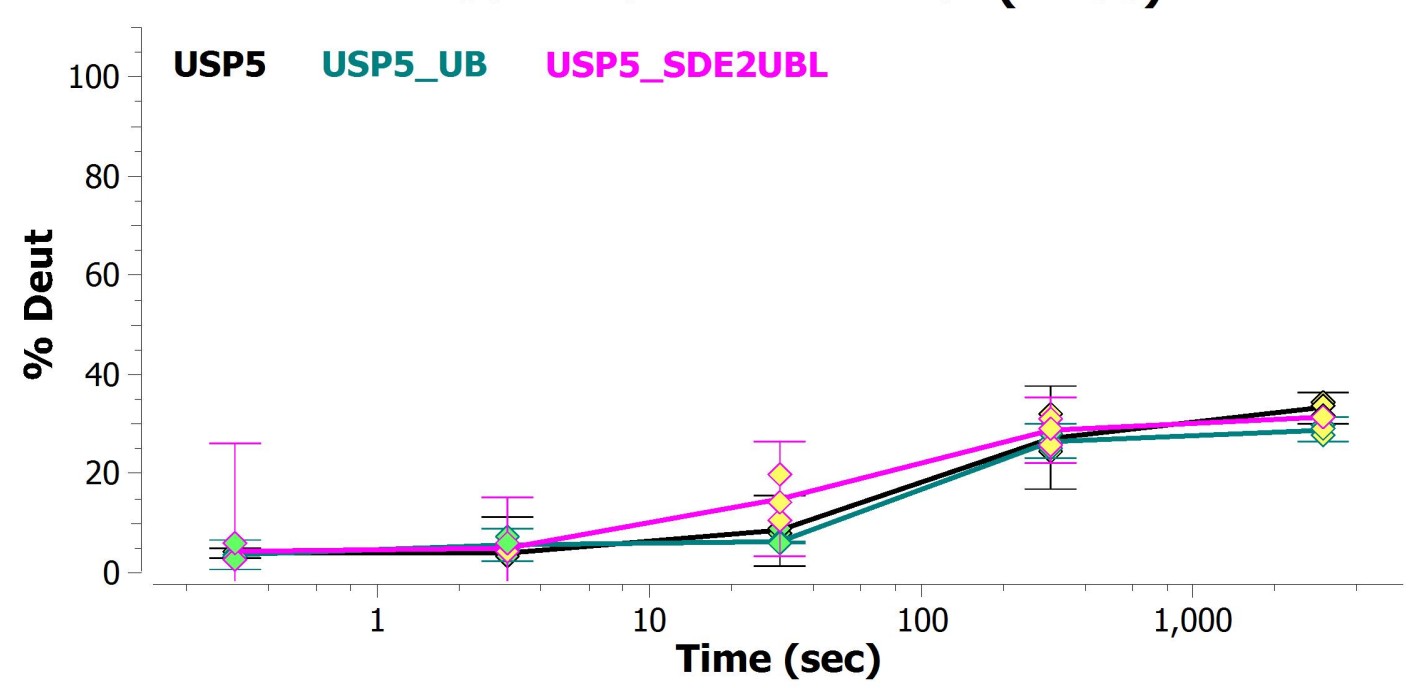

P45974 467-485: EKIKCLATEKVKYTQRVDY (#107)

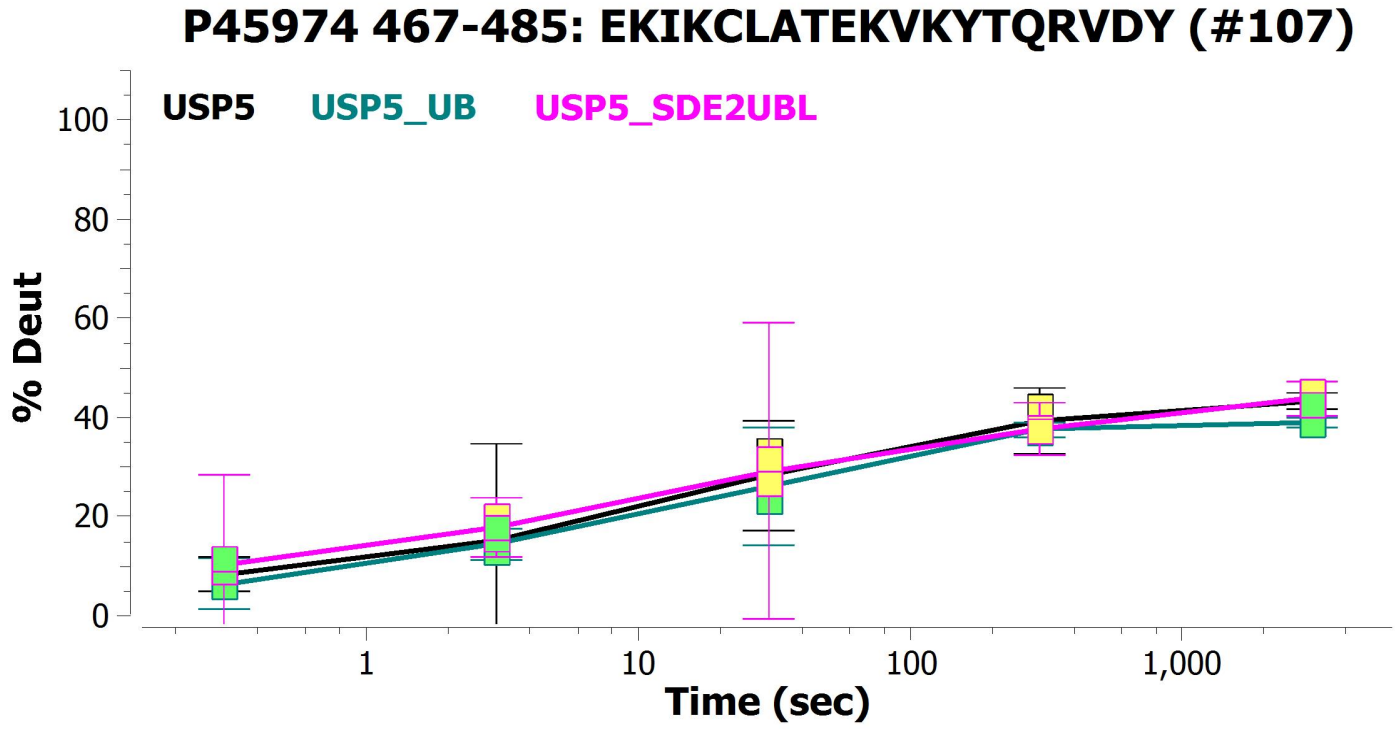

P45974 468-485: KIKCLATEKVKYTQRVDY (#108)

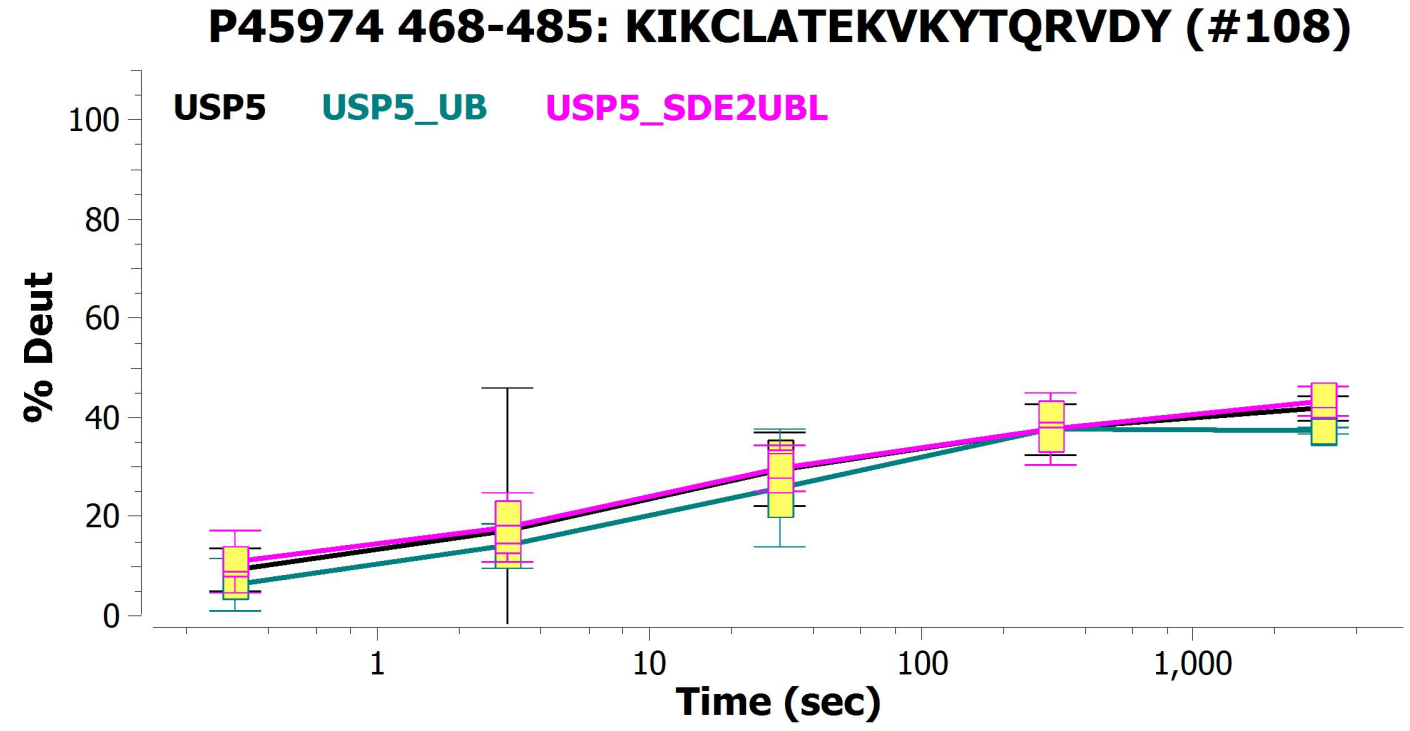

P45974 472-485: LATEKVKYTQRVDY (#109)

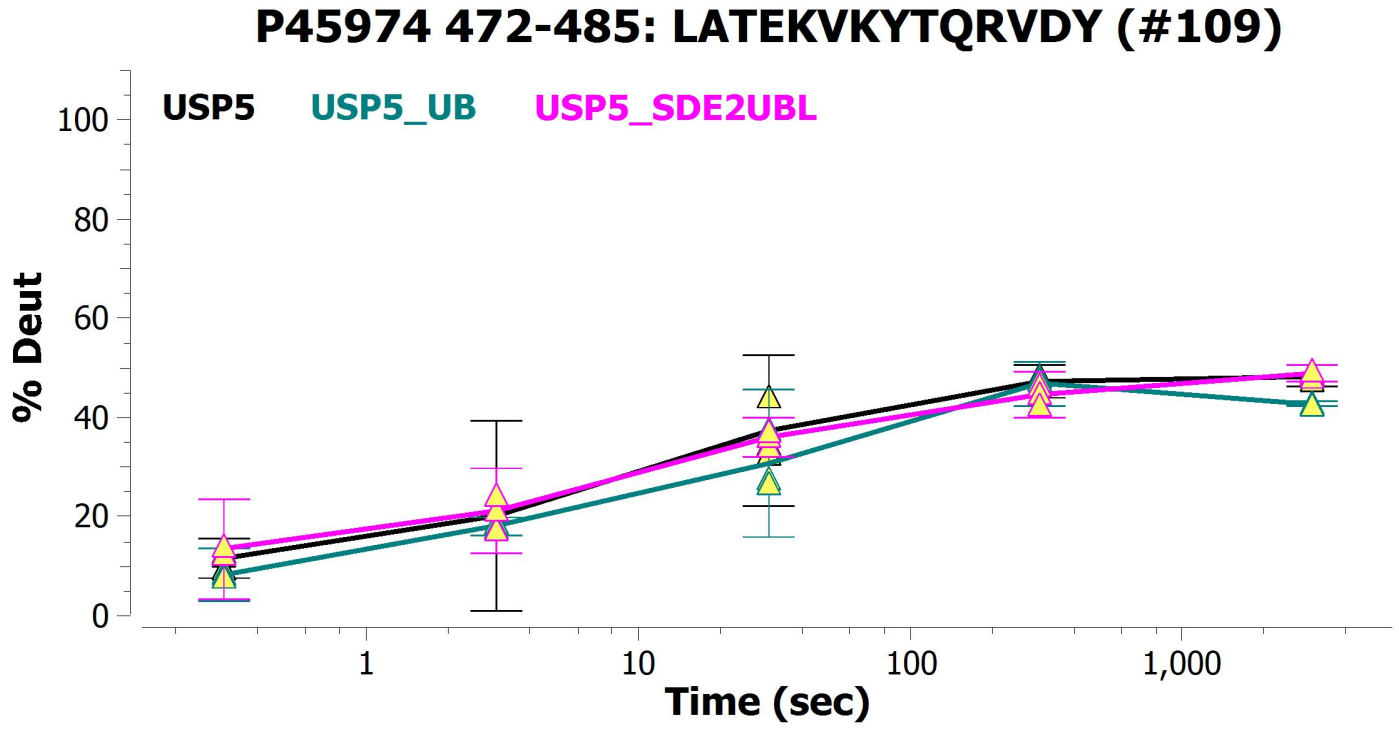

P45974 476-485: KVKYTQRVDY (#110)

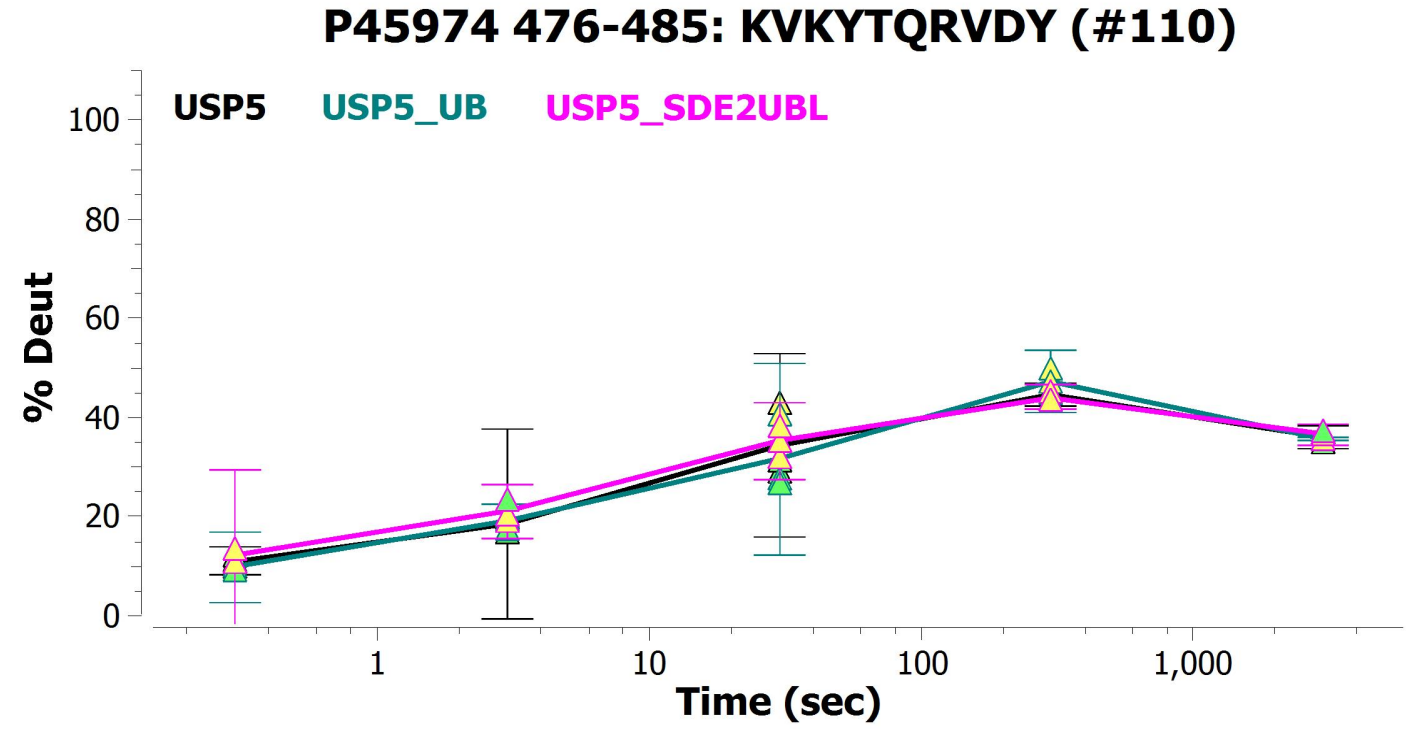

P45974 486-494: IMQLPVPMD (#111)

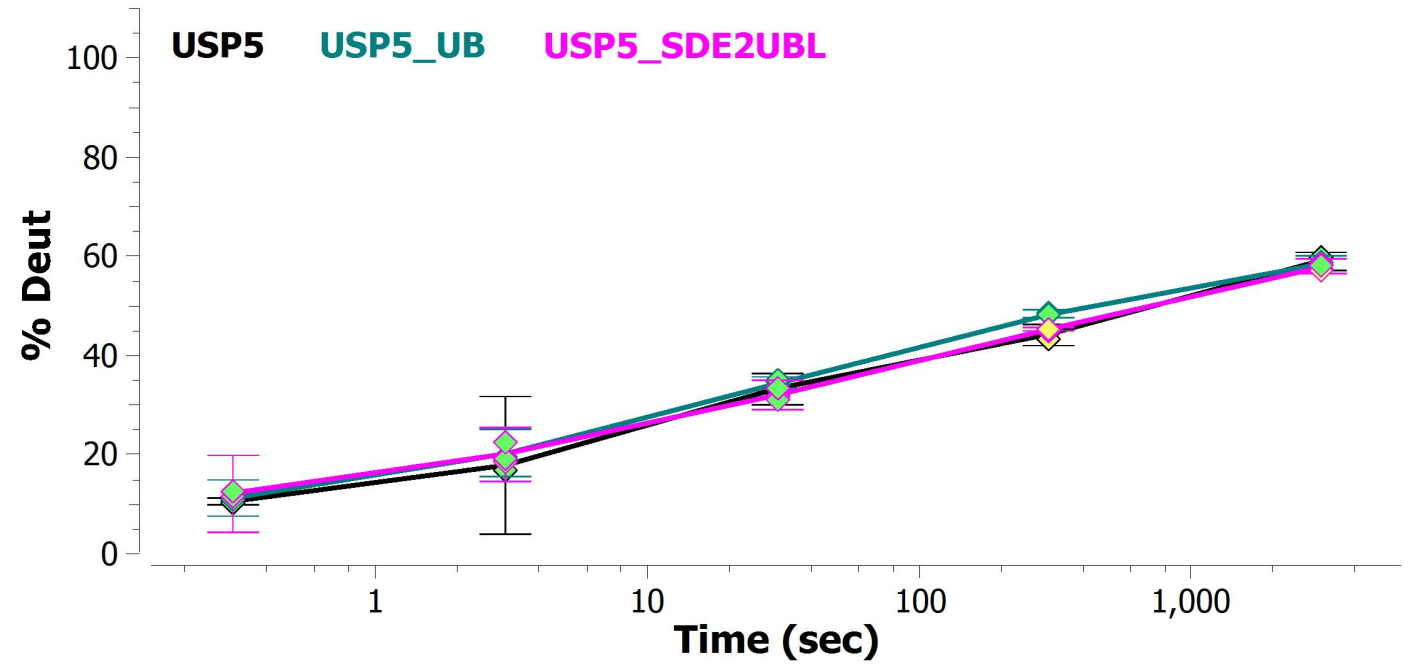

P45974 486-495: IMQLPVPMDA (#112)

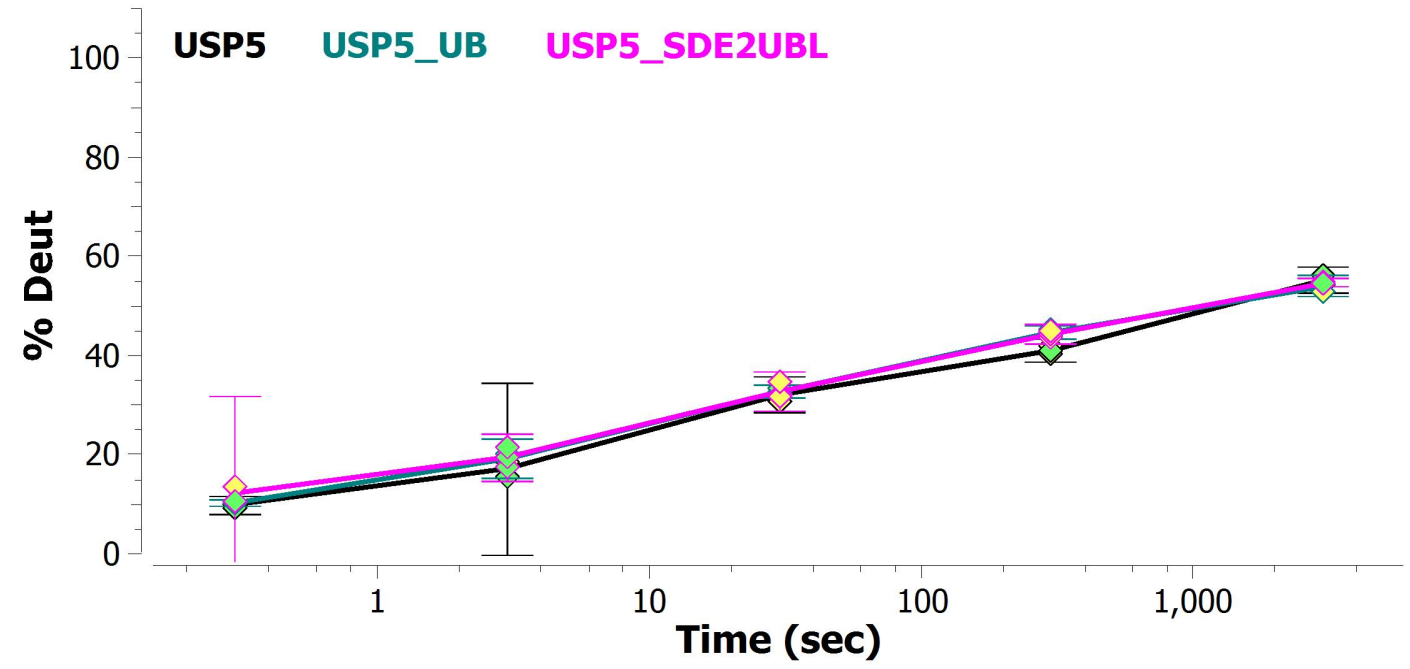

P45974 486-497: IMQLPVPMDAAL (#113)

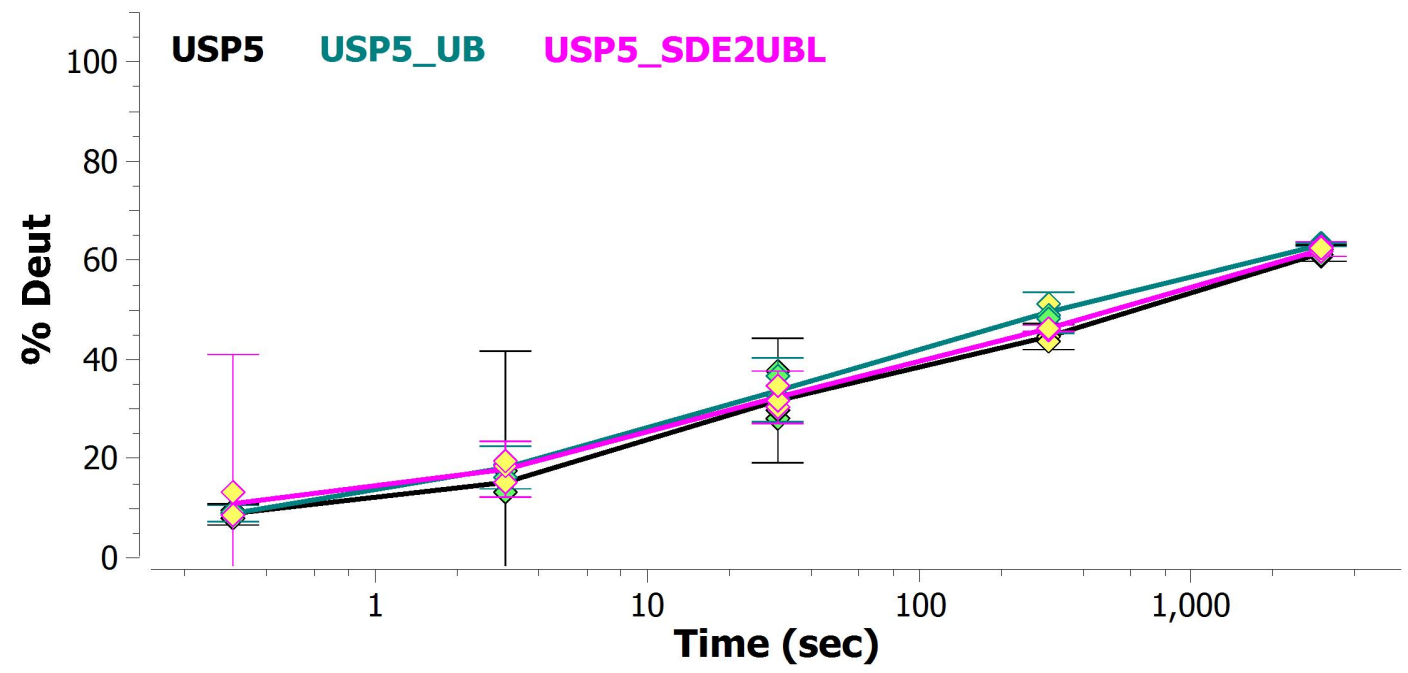

P45974 495-502: AALNKEEL (#114)

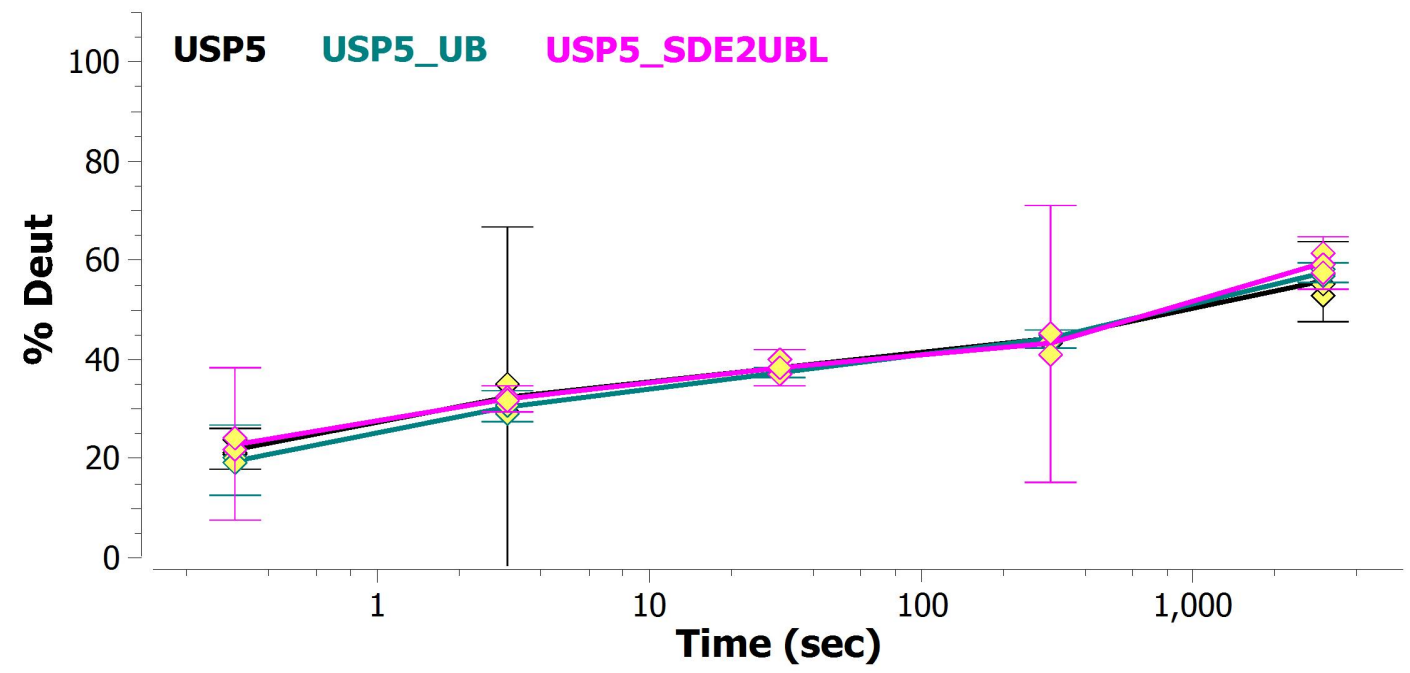

P45974 503-519: LEYEEKKRQAEEEKMAL (#115)

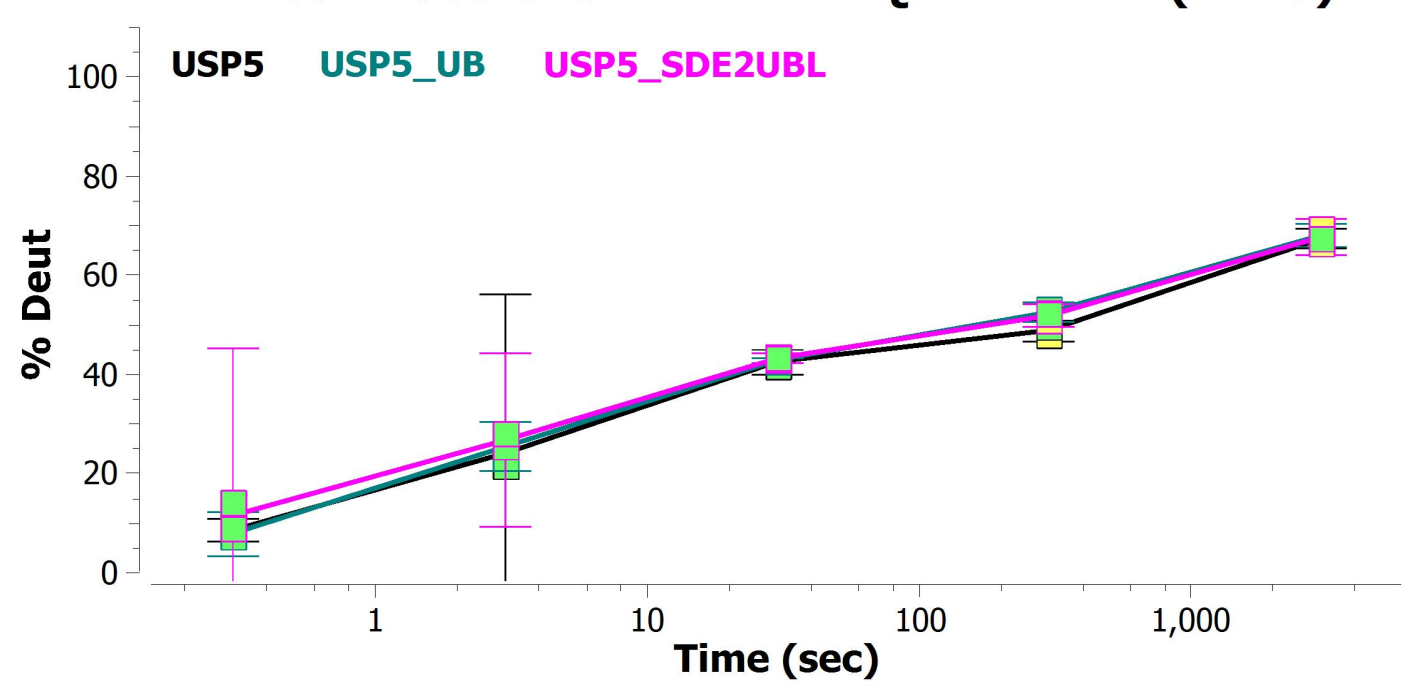

P45974 505-519: YEEKKRQAEEEKMAL (#116)

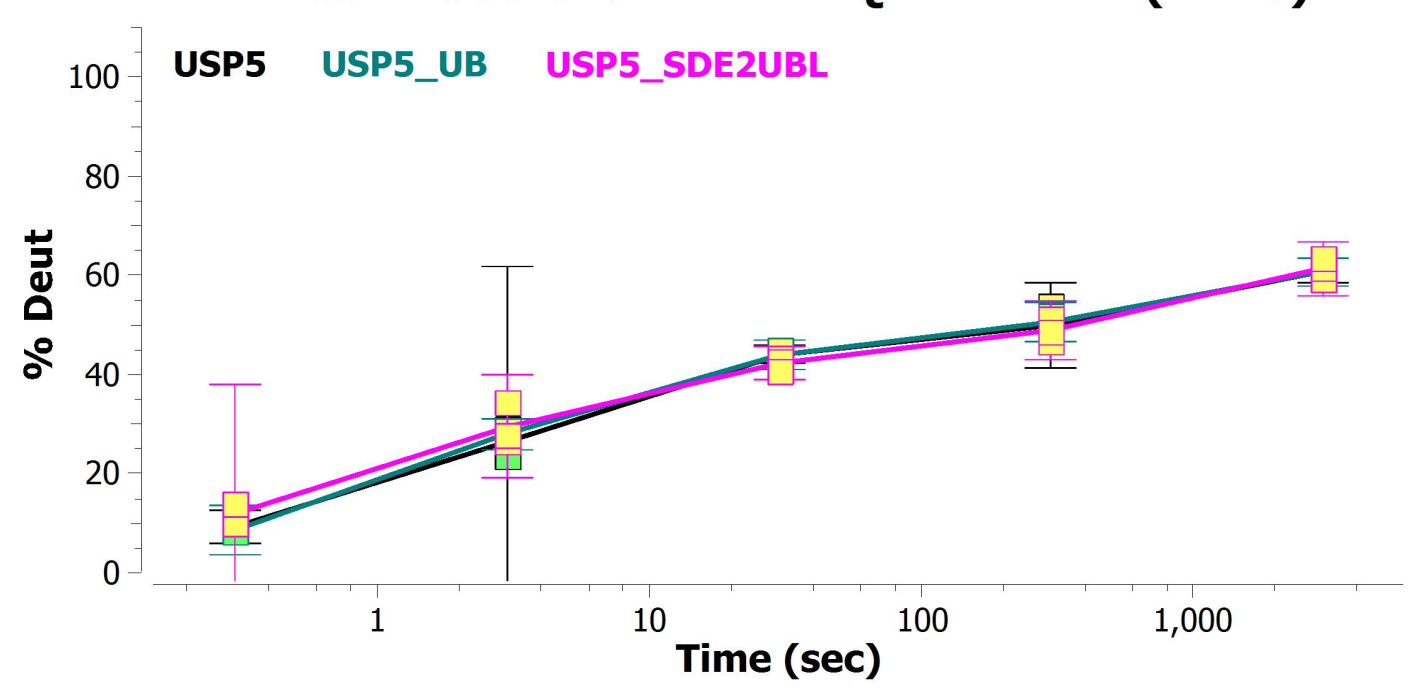

P45974 515-533: EKMALPELVRAQVPFSSCL (#117)

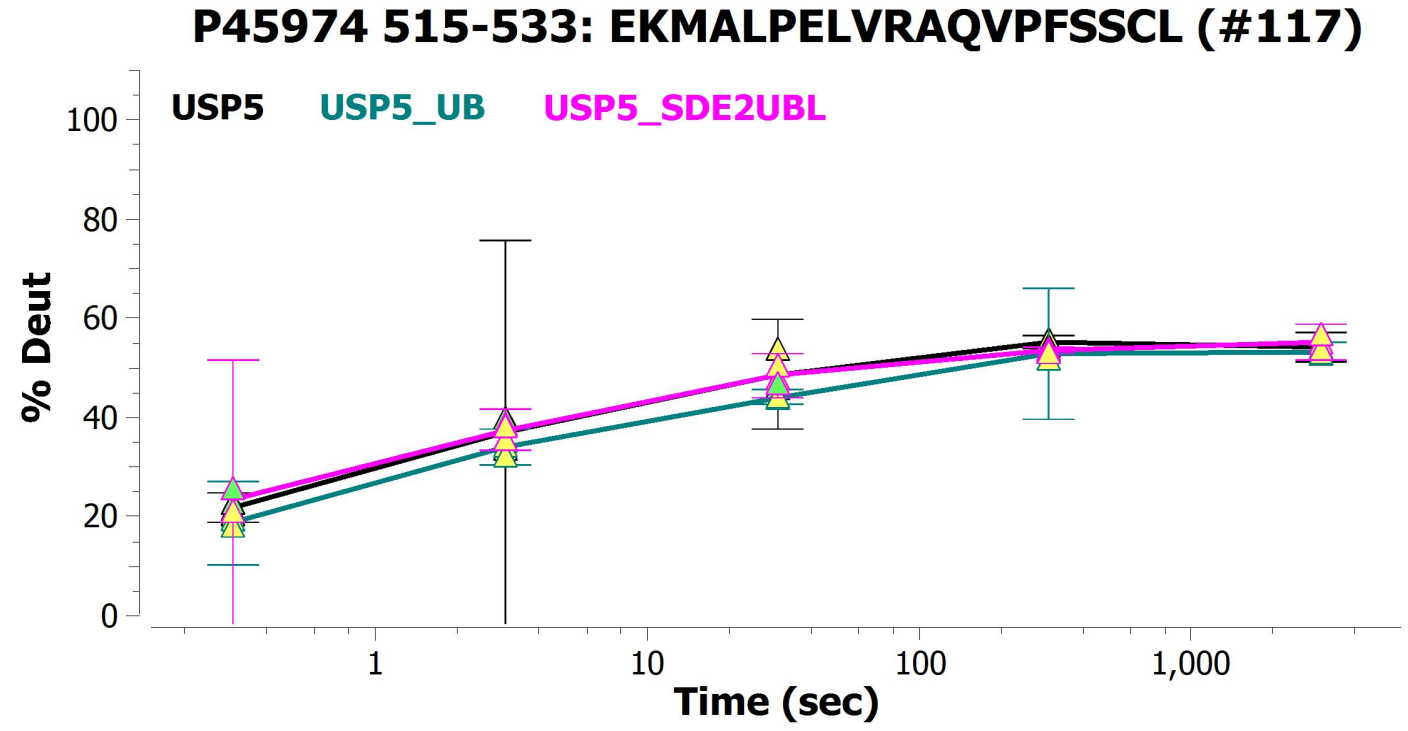

P45974 516-533: KMALPELVRAQVPFSSCL (#118)

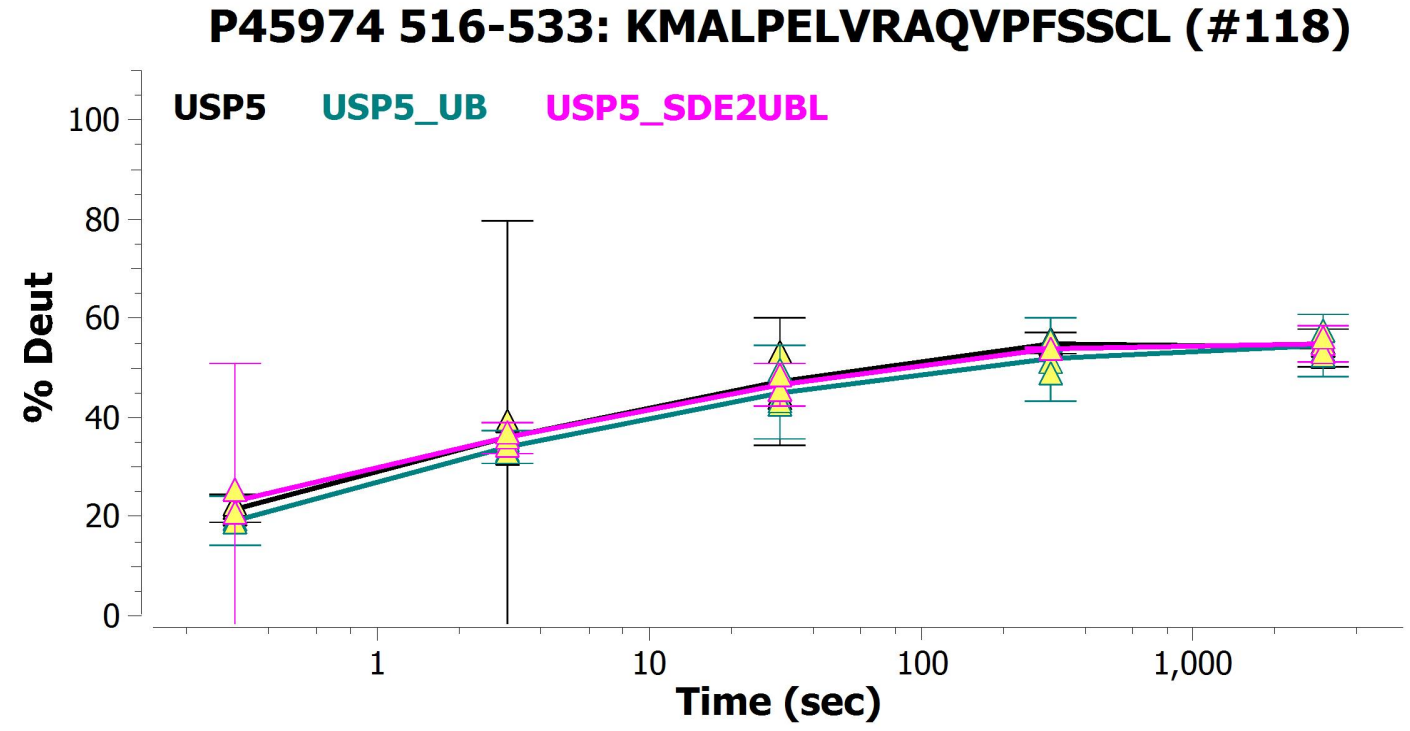

P45974 520-533: PELVRAQVPFSSCL (#119)

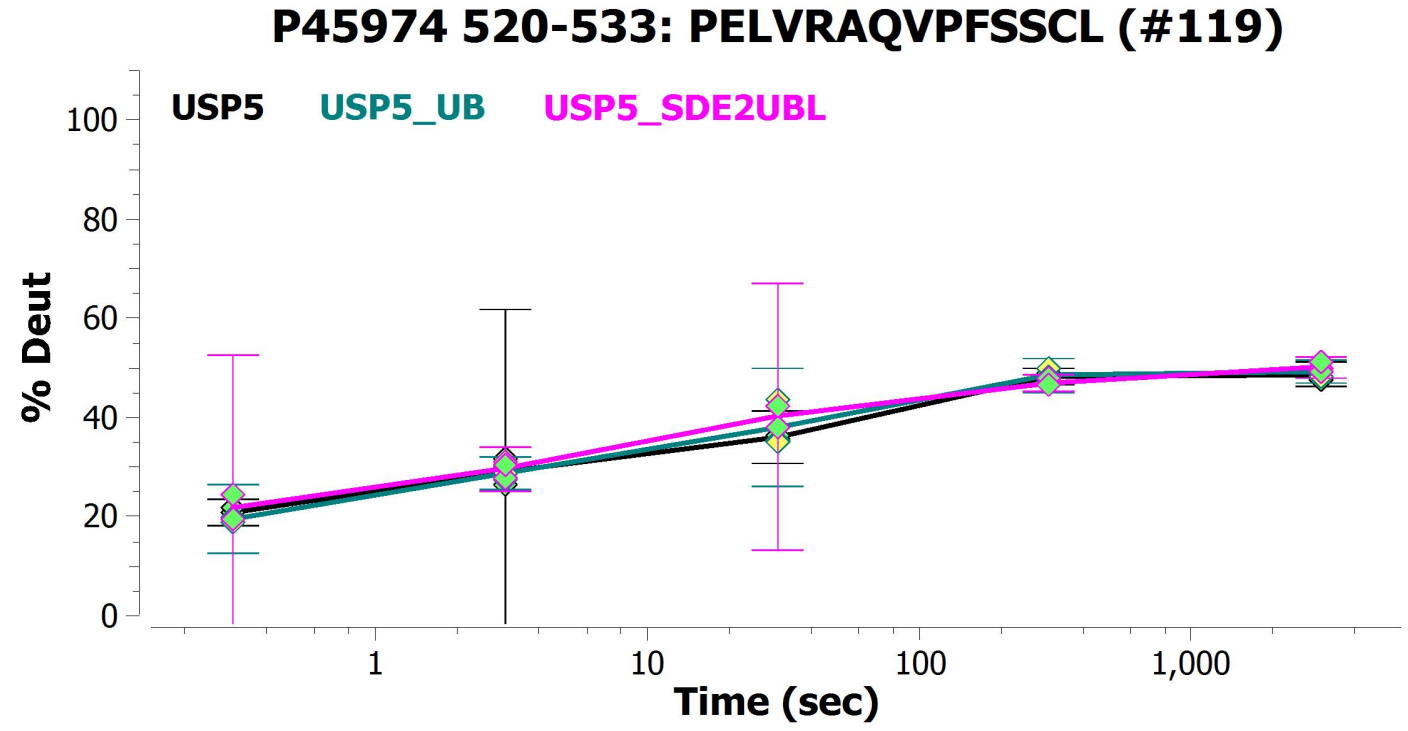

P45974 523-533: VRAQVPFSSCL (#120)

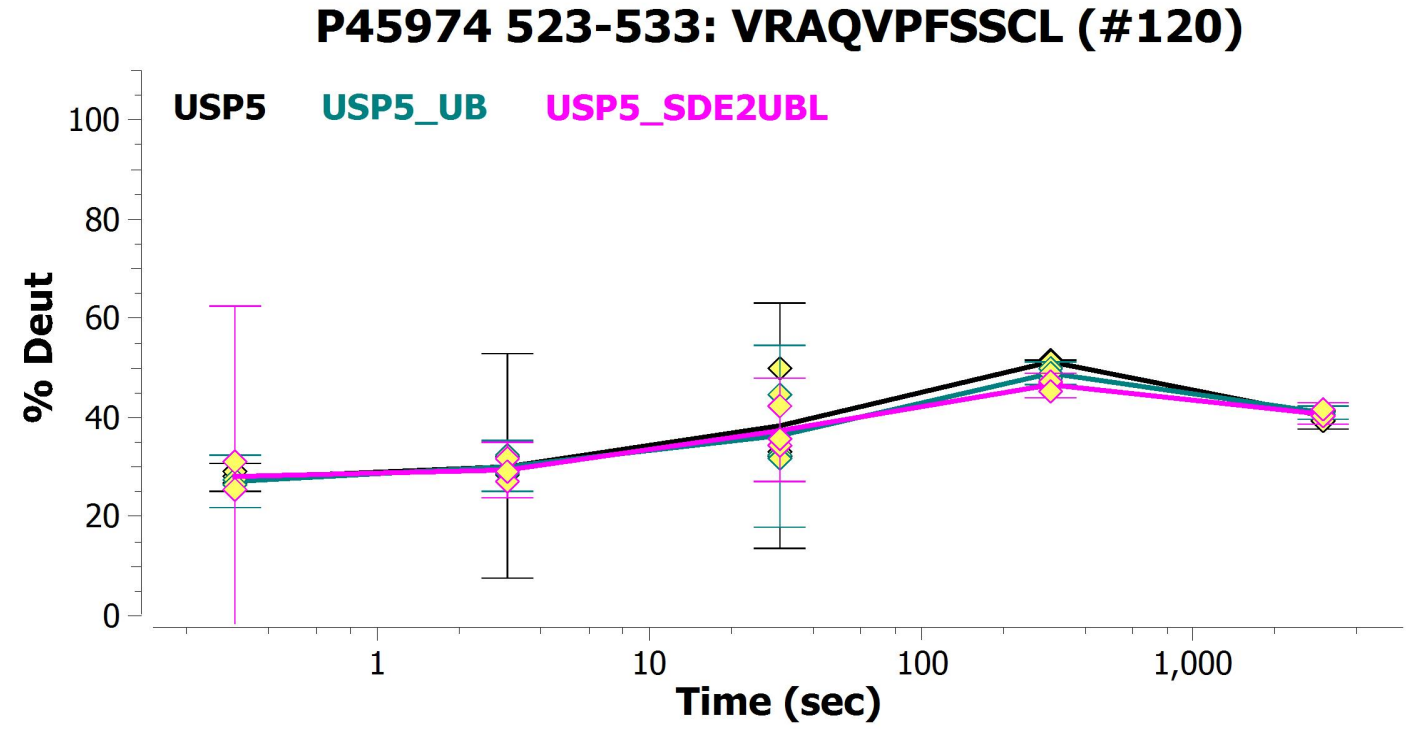

P45974 533-545: LEAYGAPEQVDDF (#121)

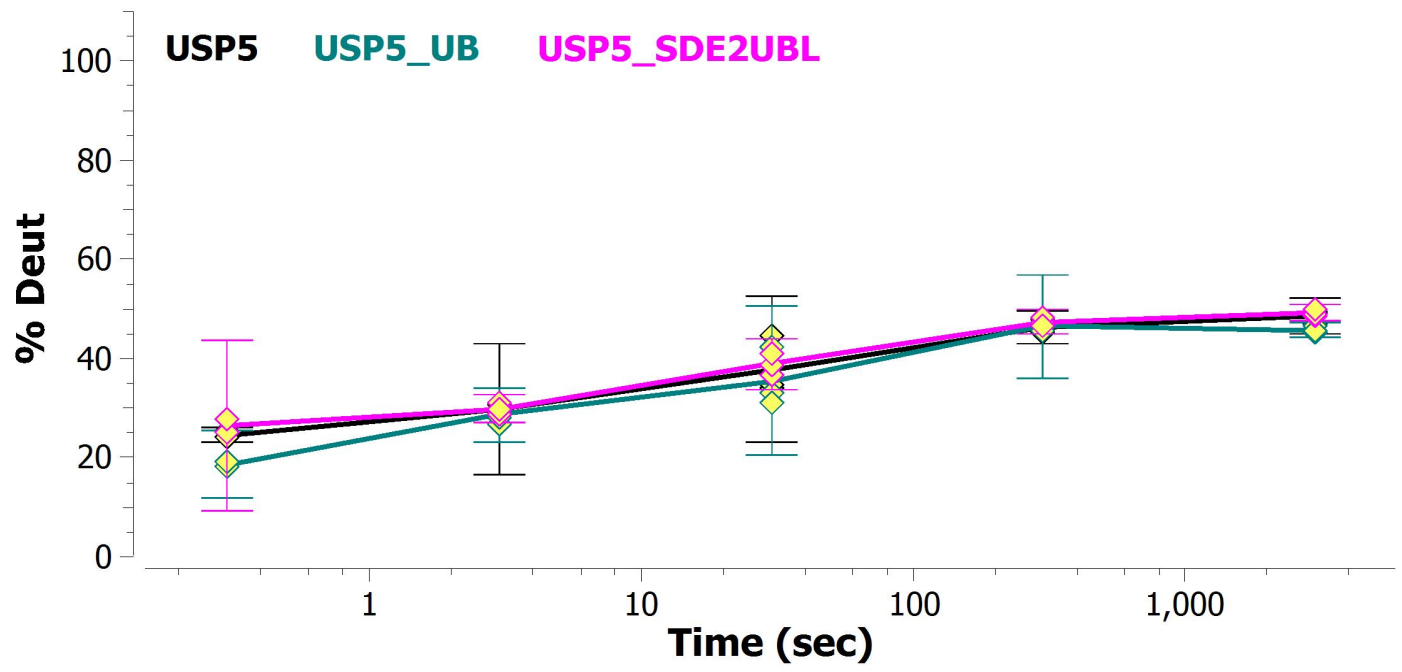

P45974 534-545: EAYGAPEQVDDF (#122)

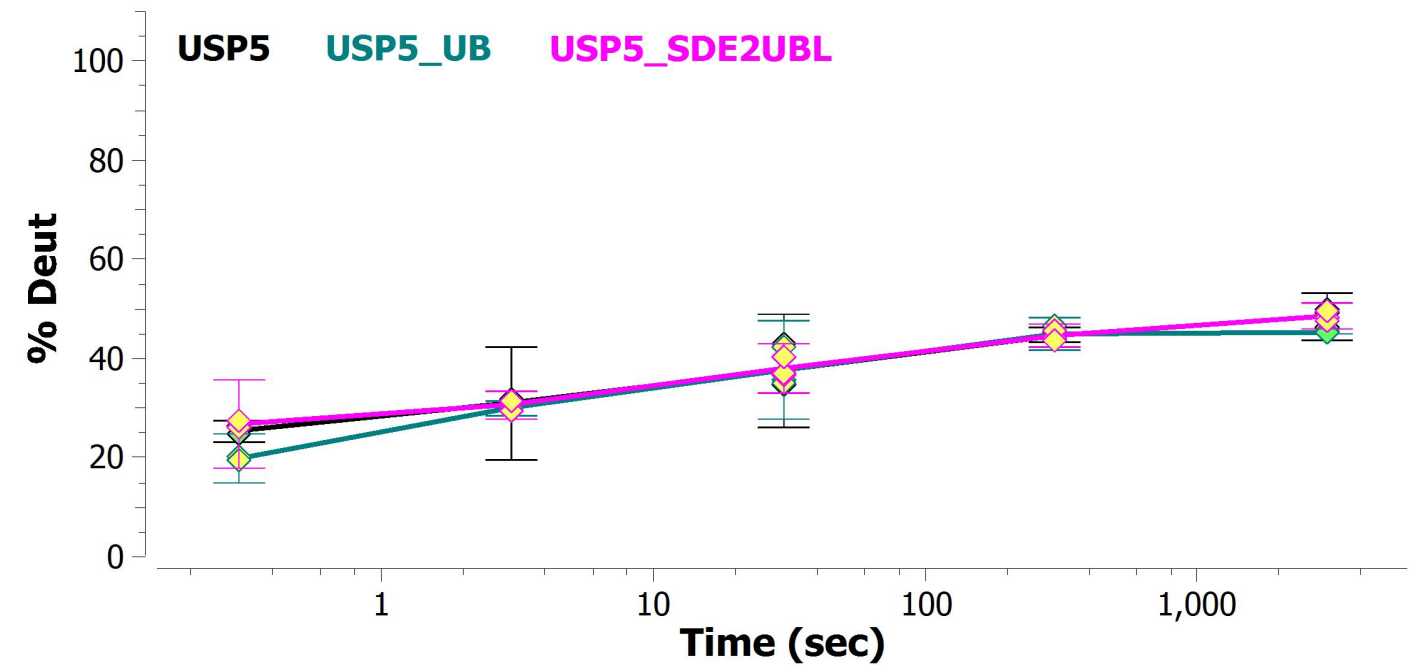

P45974 536-545: YGAPEQVDDF (#123)

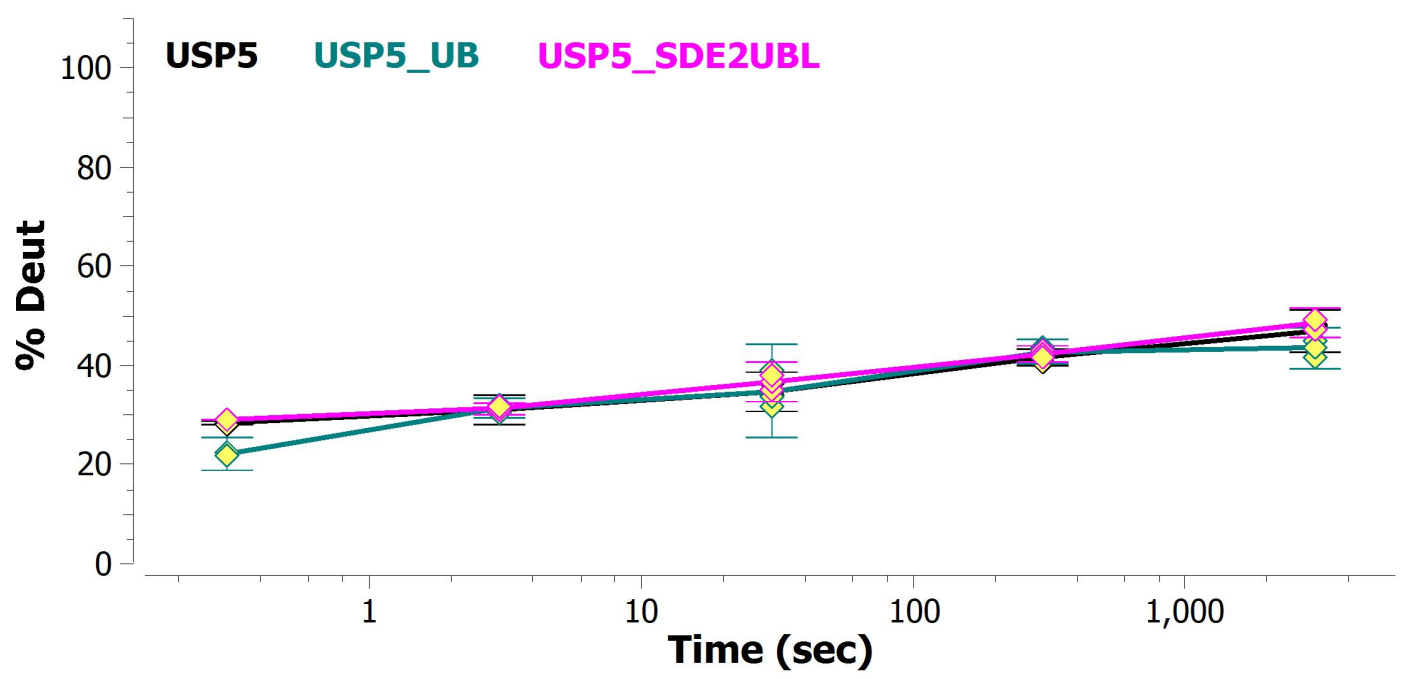

P45974 539-545: PEQVDDF (#124)

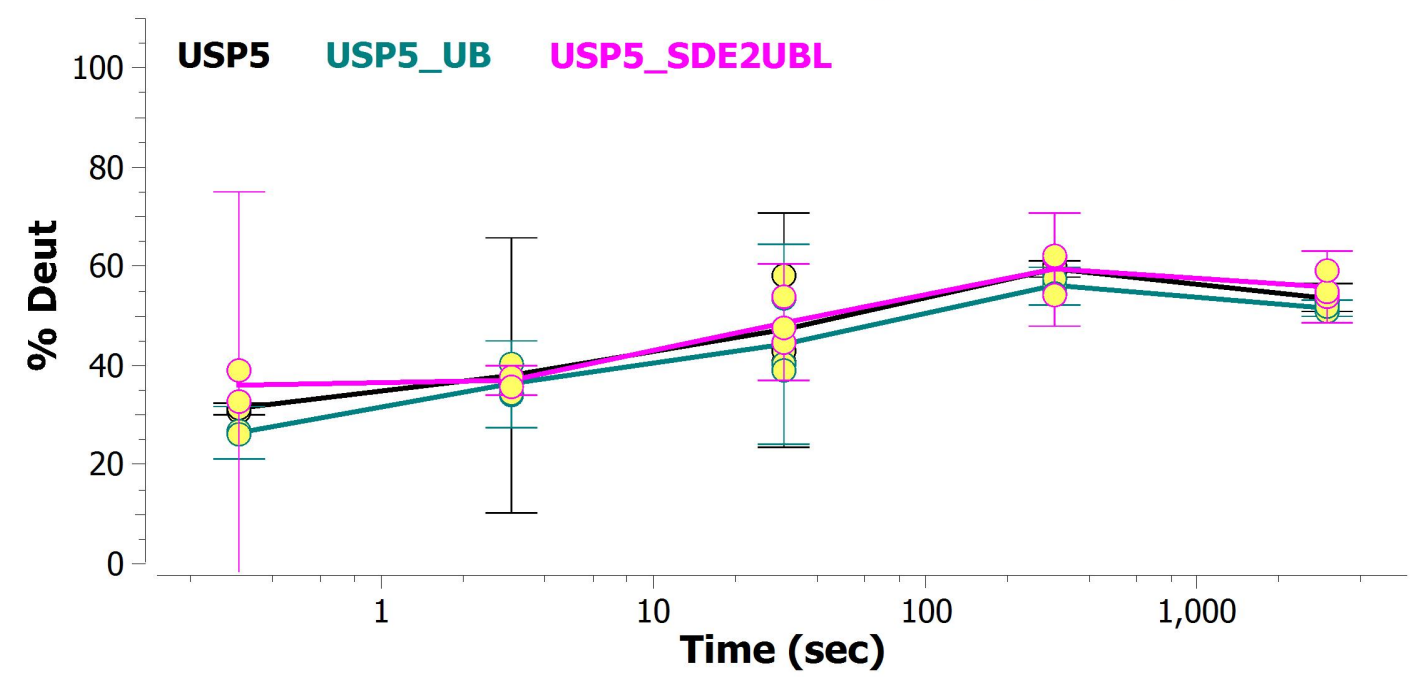

P45974 546-556: WSTALQAKSVA (#125)

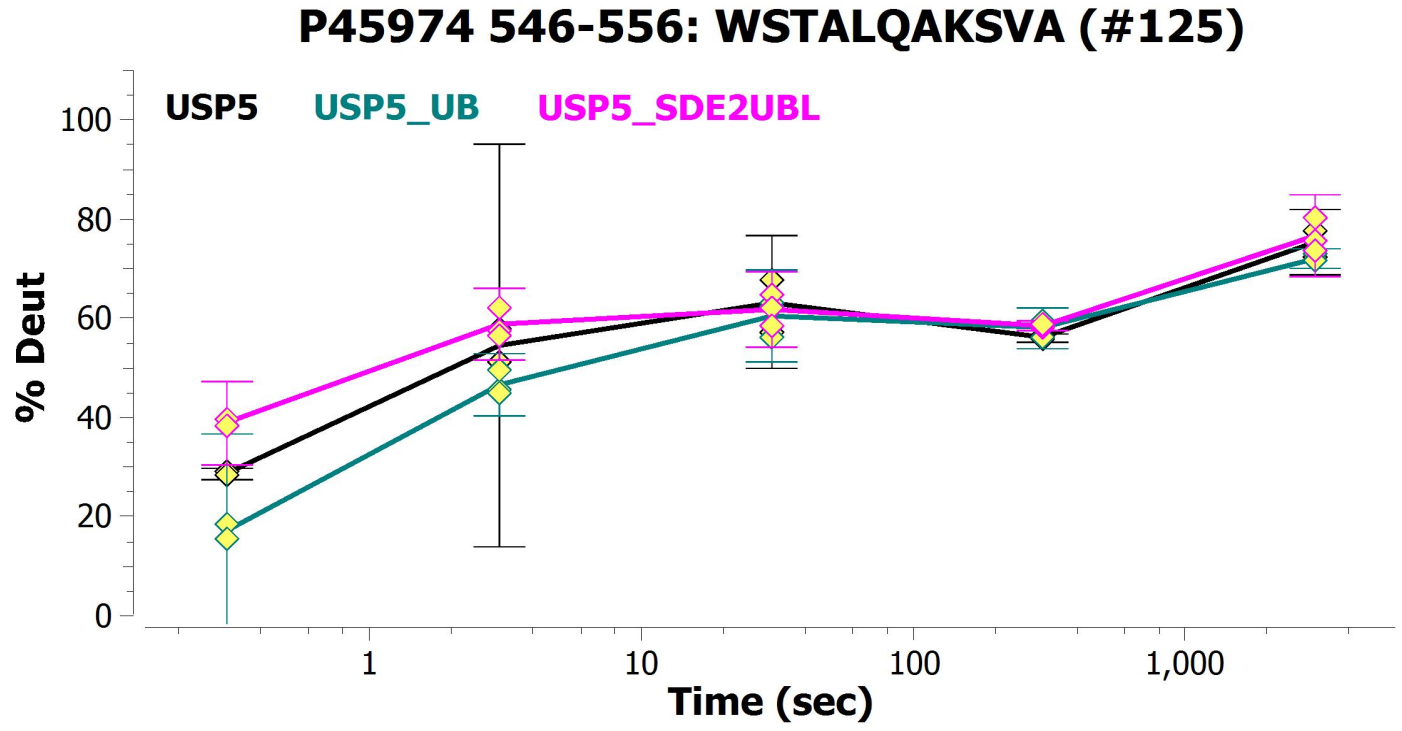

P45974 546-562: WSTALQAKSVAVKTTRF (#126)

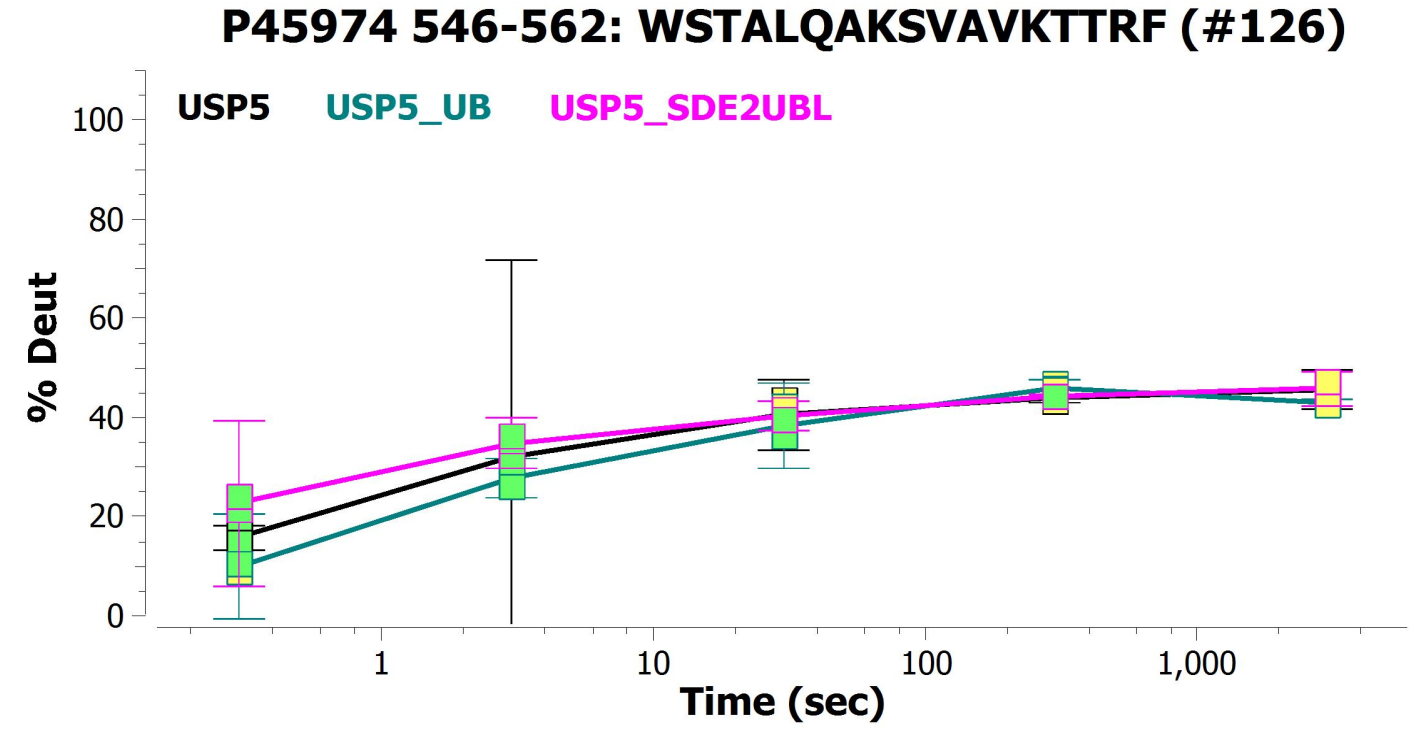

P45974 569-580: LVIQIKKFTFGL (#127)

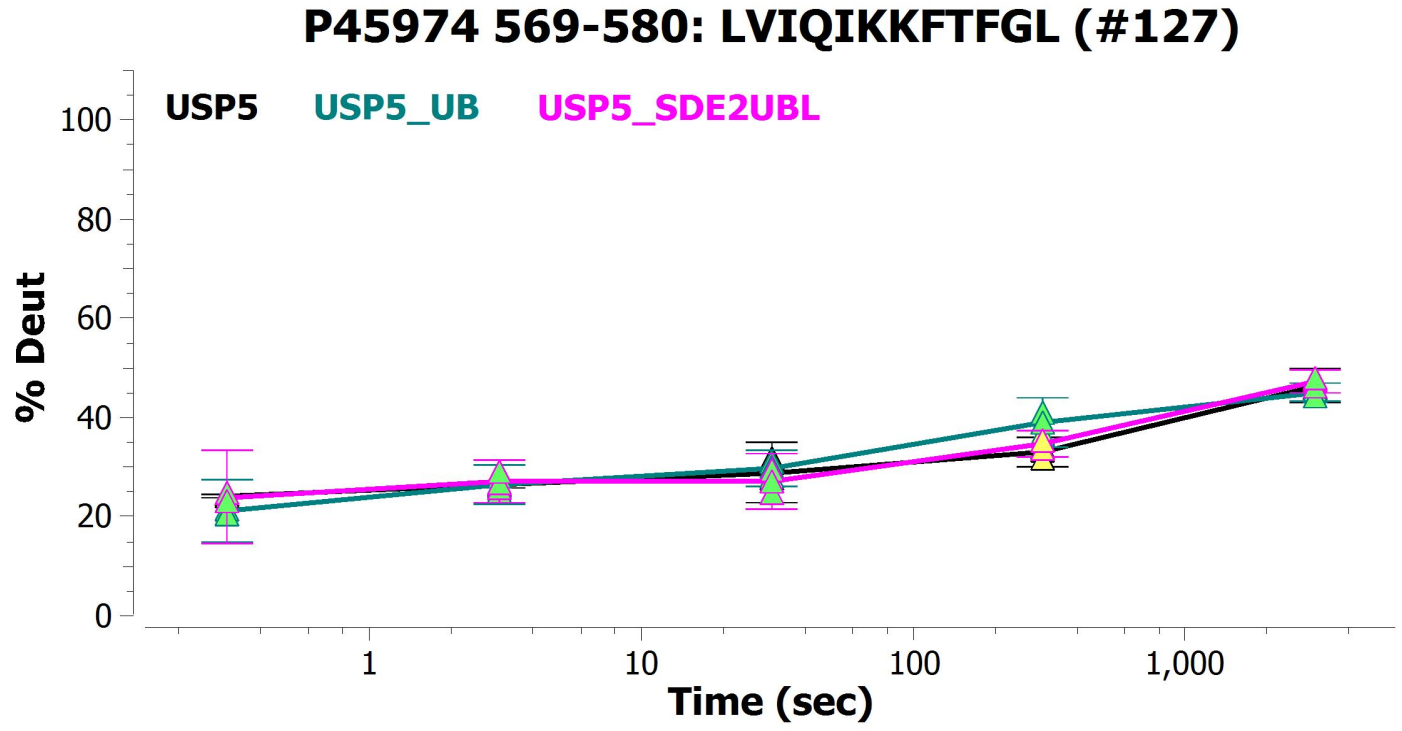

P45974 569-581: LVIQIKKFTFGLD (#128)

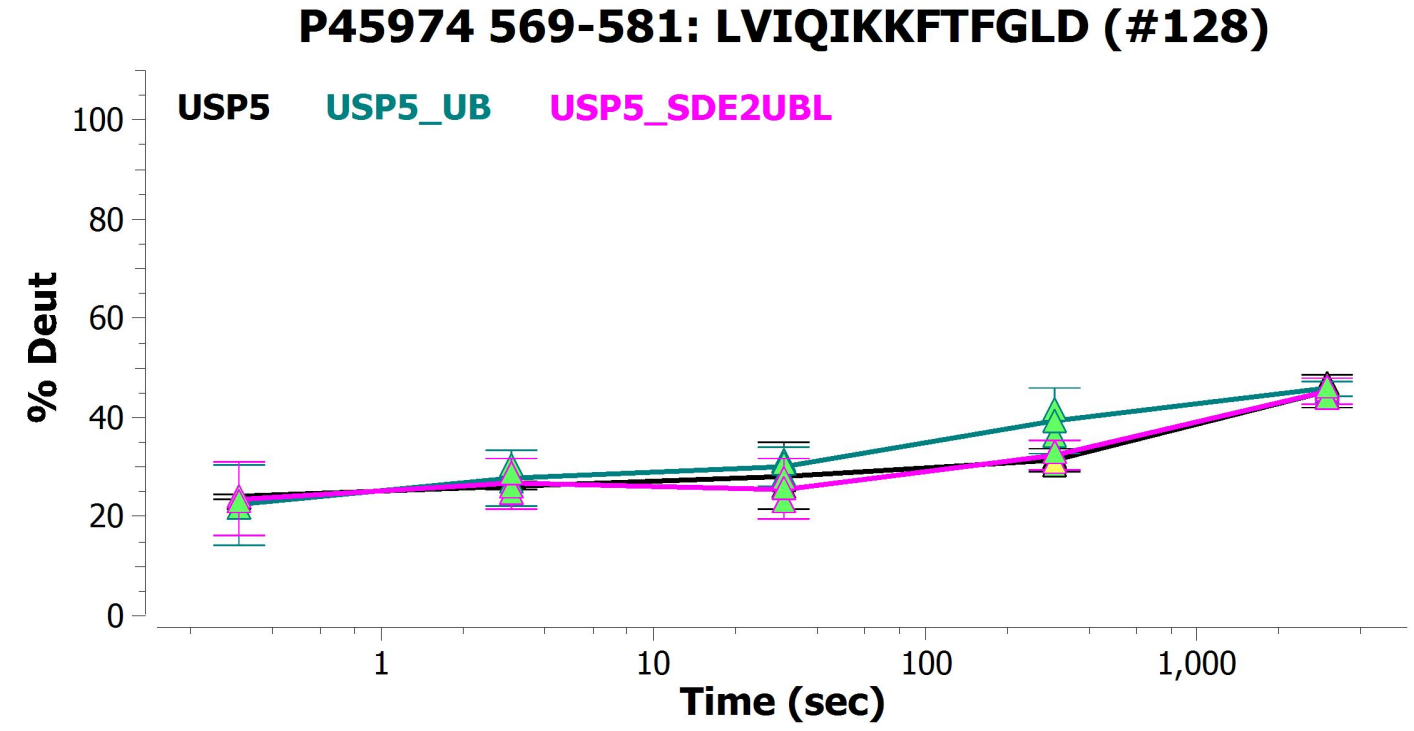

P45974 569-589: LVIQIKKFTFGLDWVPKKLDV (#129)

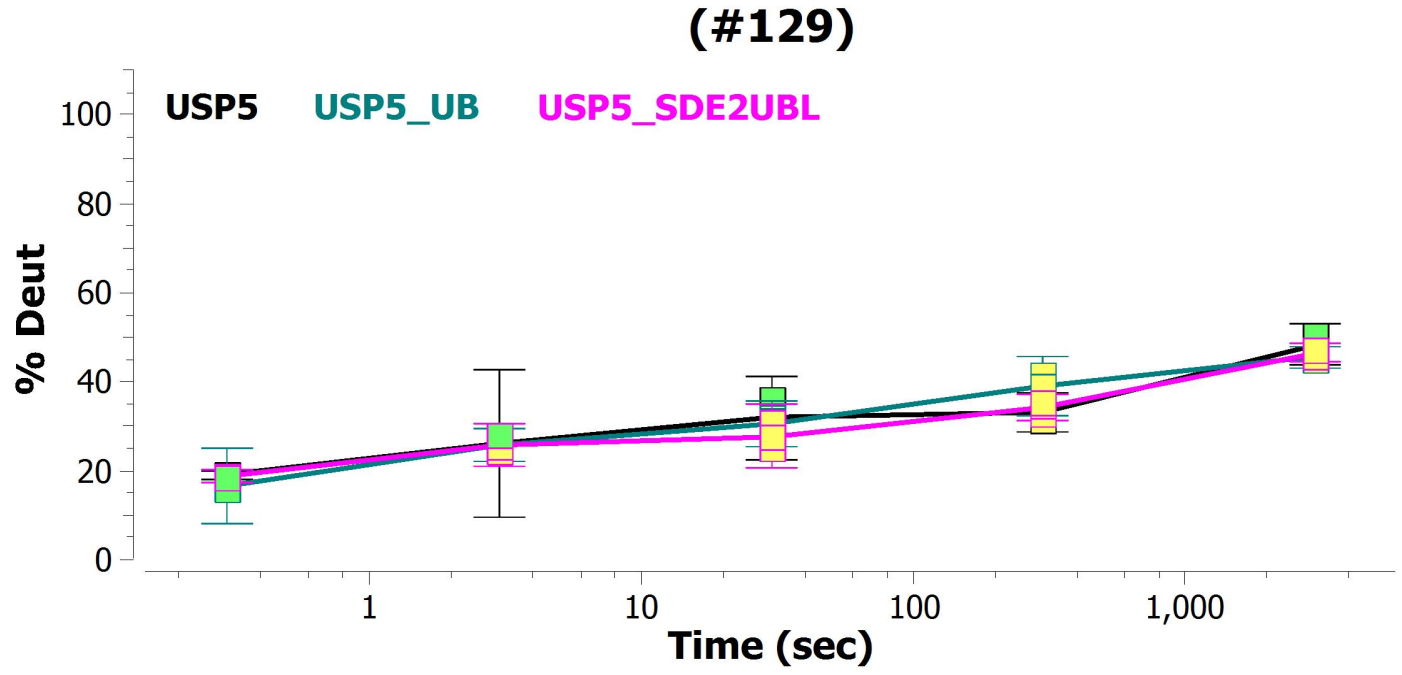

P45974 569-590: LVIQIKKFTFGLDWVPKKLDVS (#130)

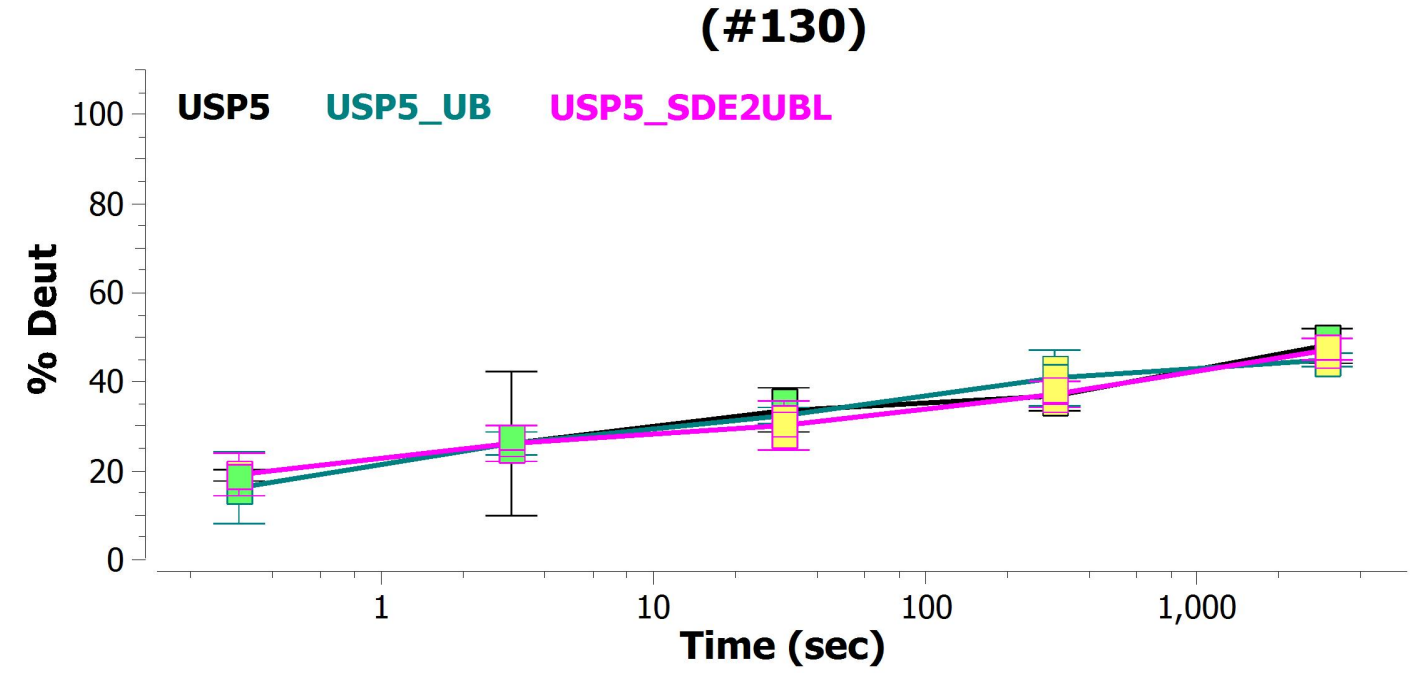

P45974 570-580: VIQIKKFTFGL (#131)

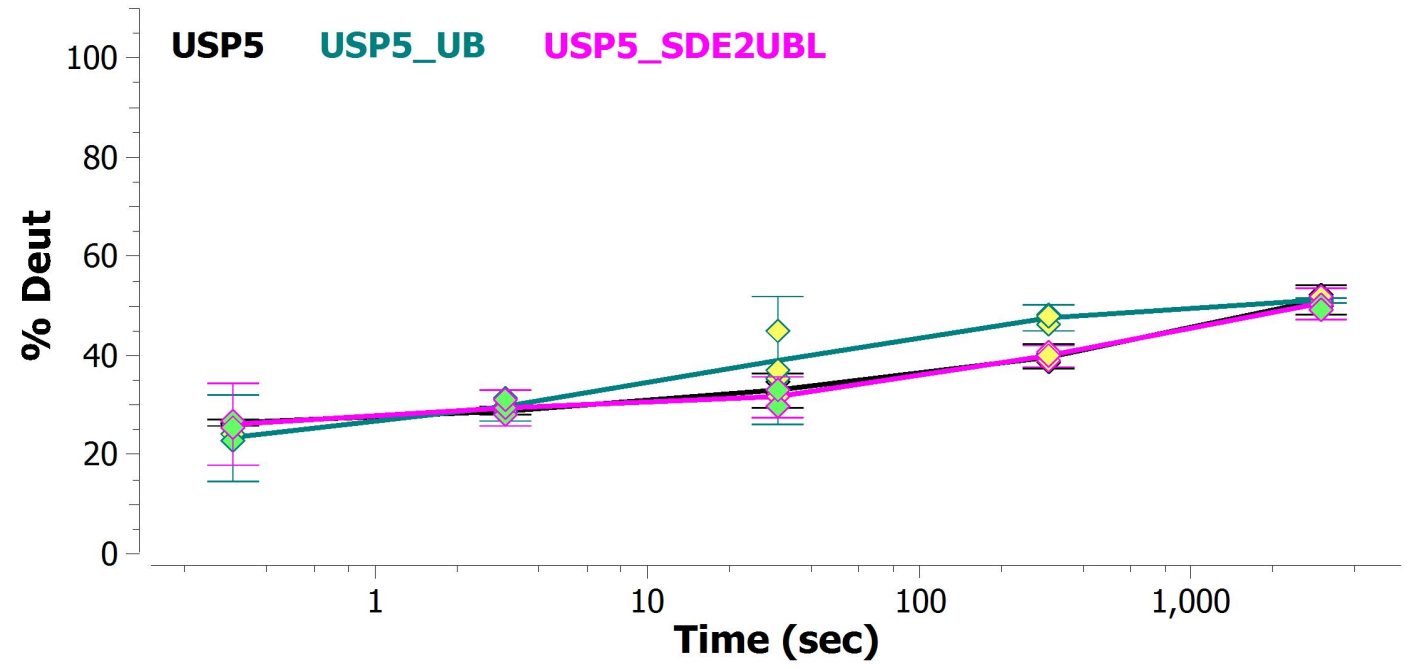

P45974 570-581: VIQIKKFTFGLD (#132)

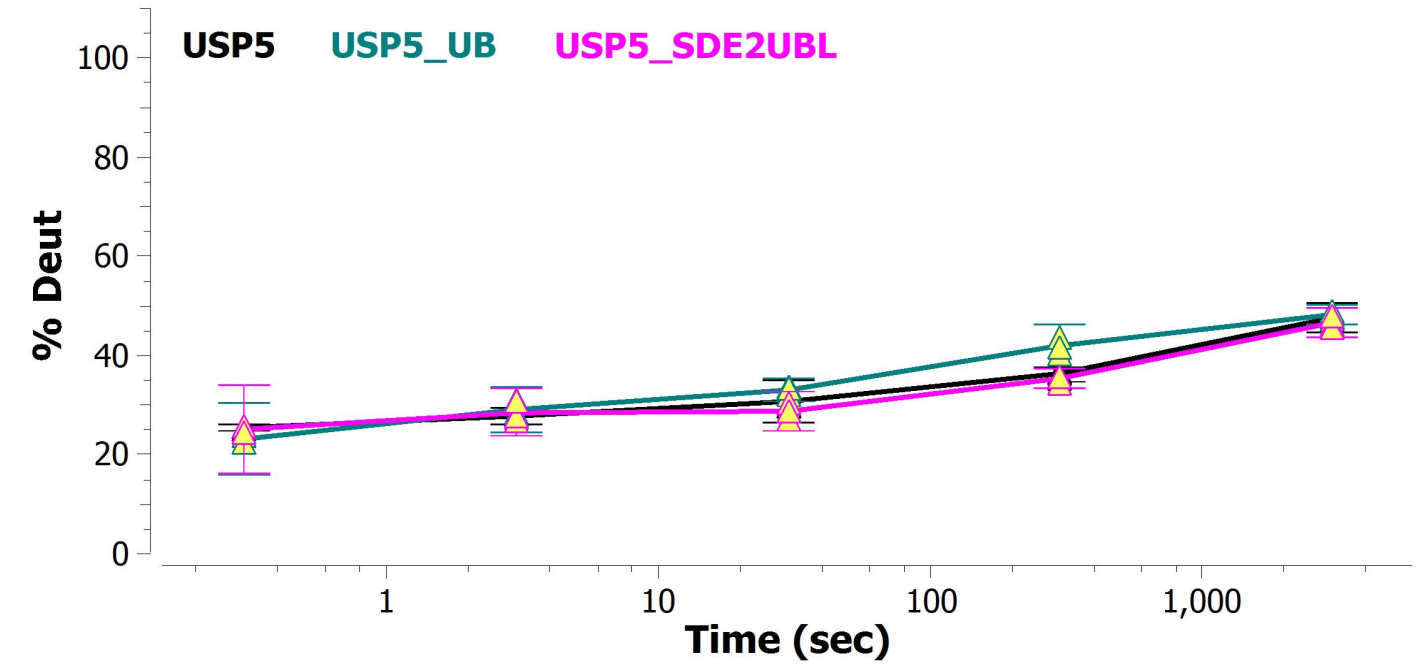

P45974 570-589: VIQIKKFTFGLDWVPKKLDV (#133)

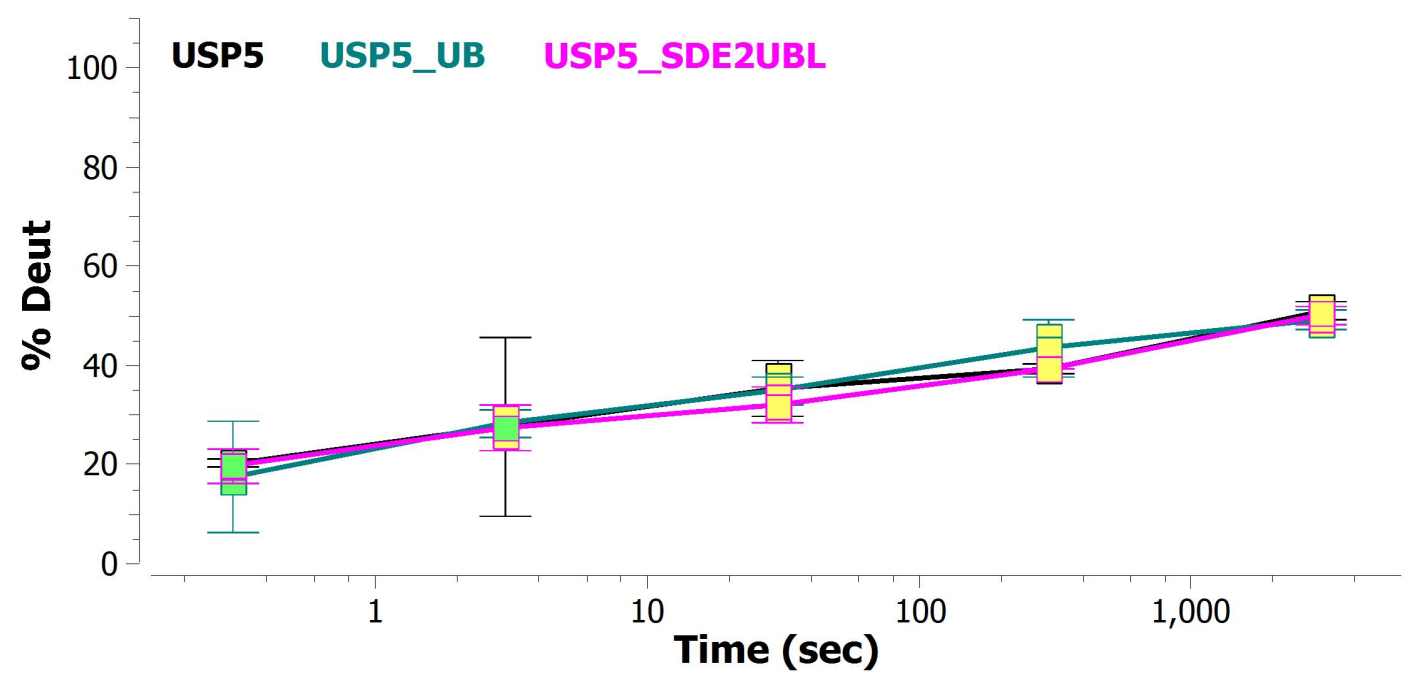

P45974 570-590: VIQIKKFTFGLDWVPKKLDVS (#134)

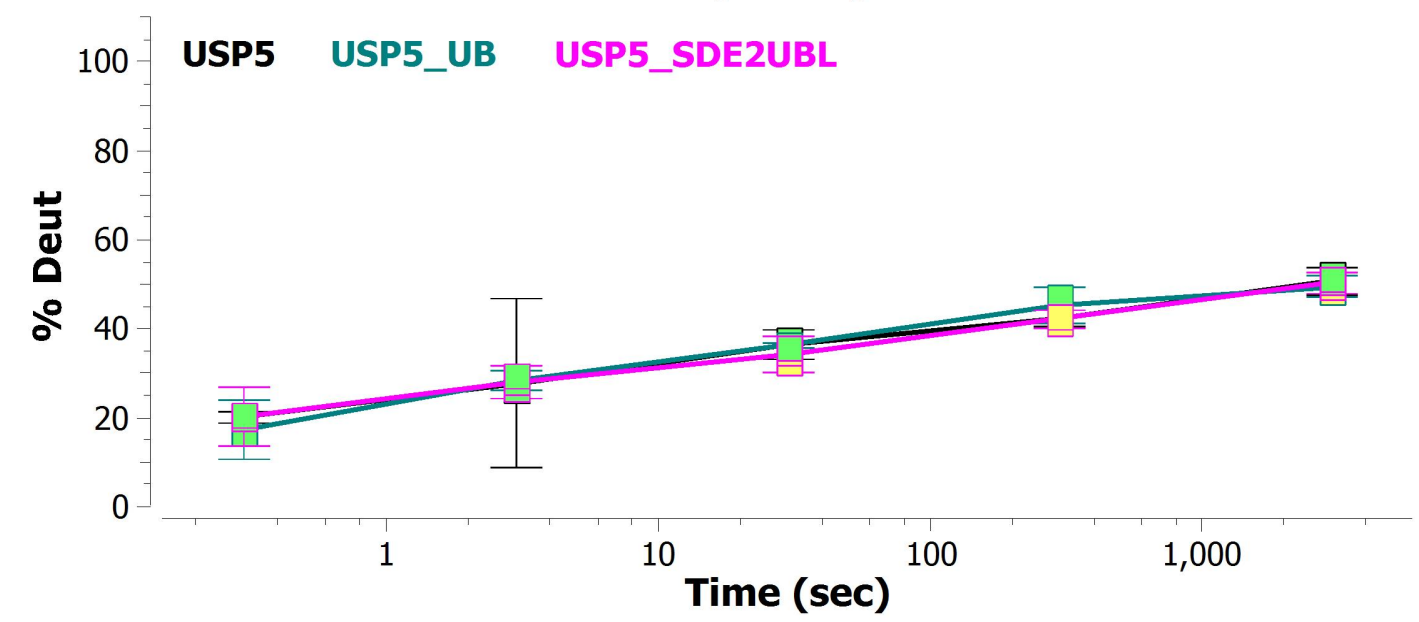

P45974 571-580: IQIKKFTFGL (#135)

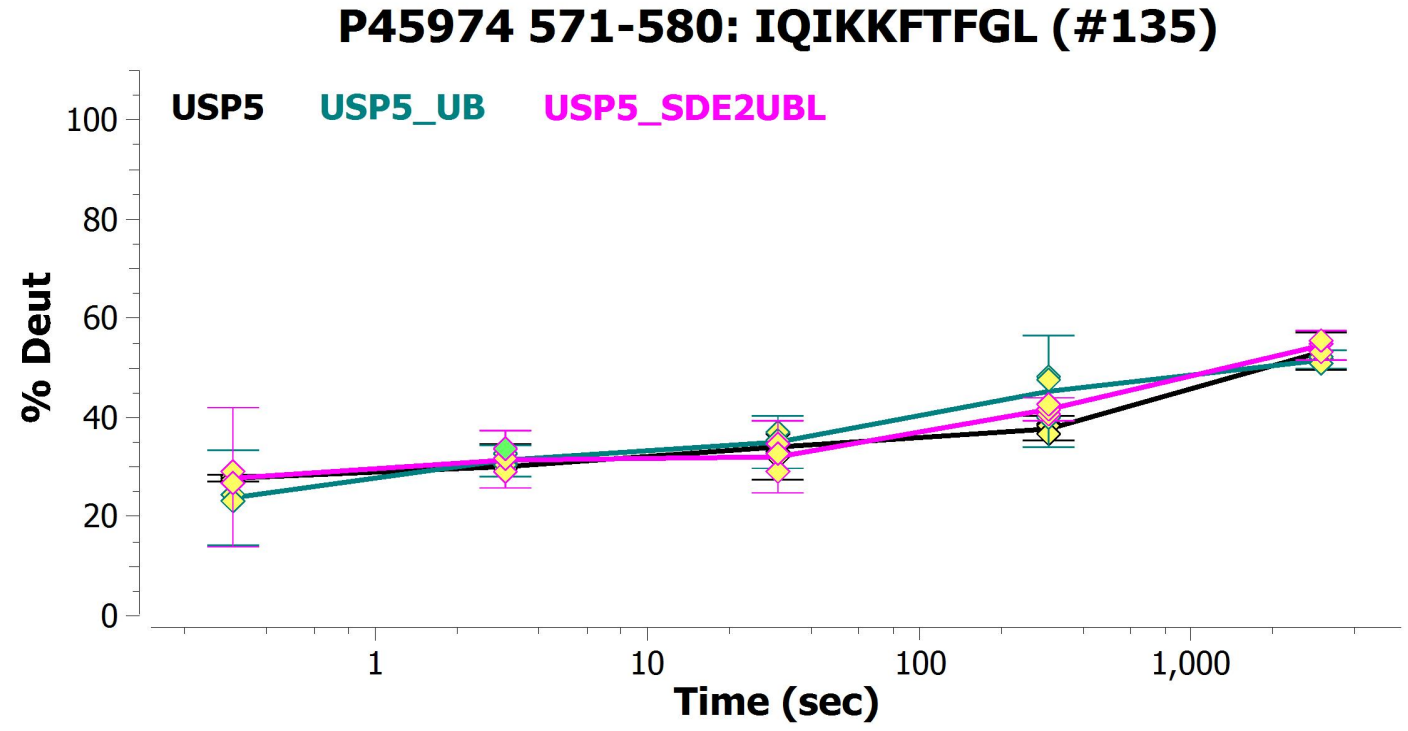

P45974 571-581: IQIKKFTFGLD (#136)

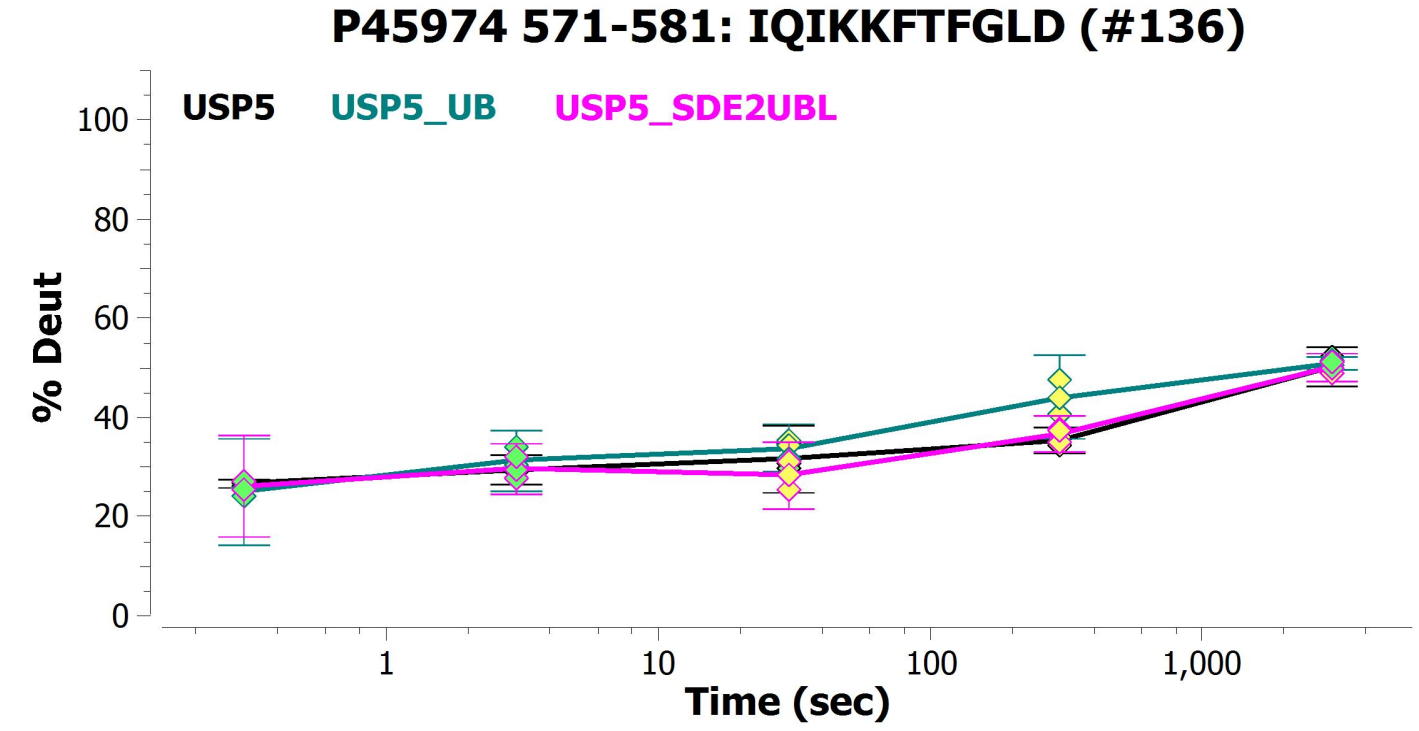

P45974 572-580: QIKKFTFGL (#137)

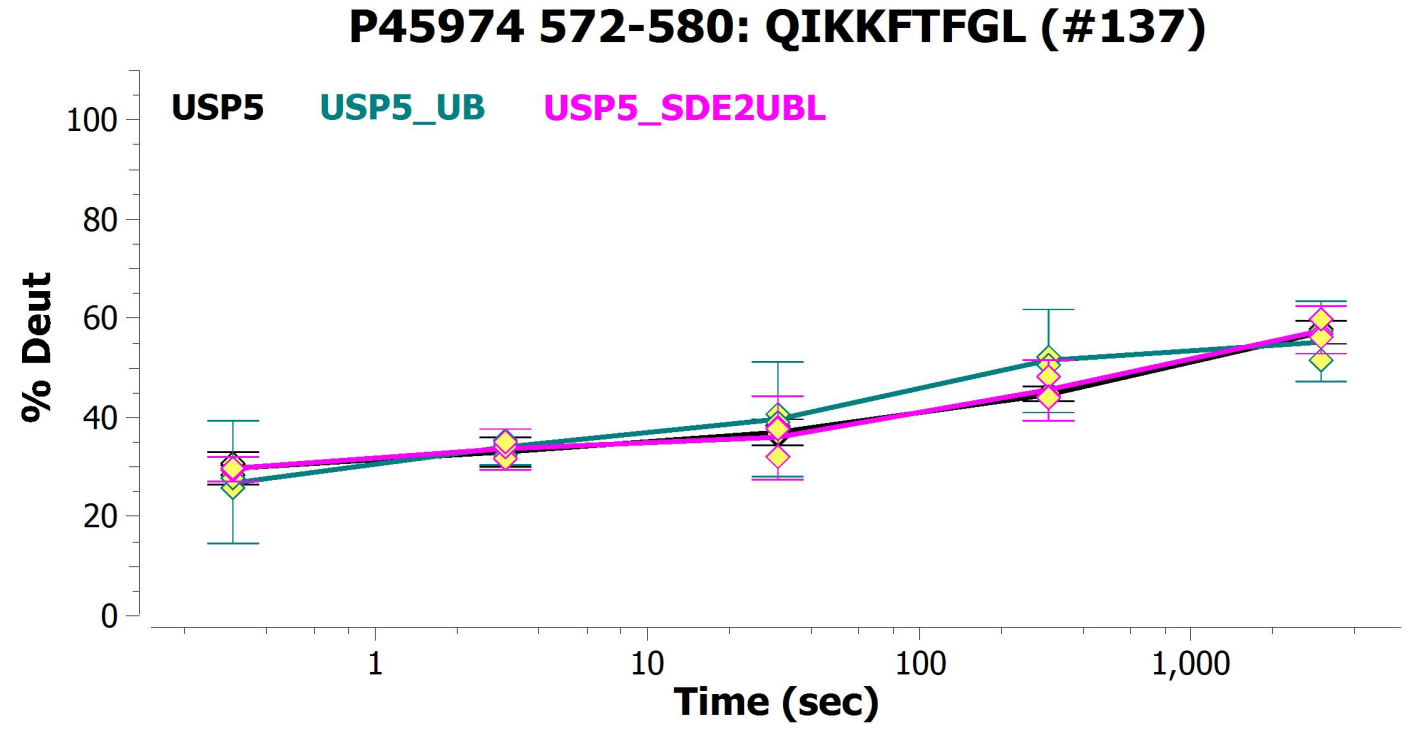

P45974 572-581: QIKKFTFGLD (#138)

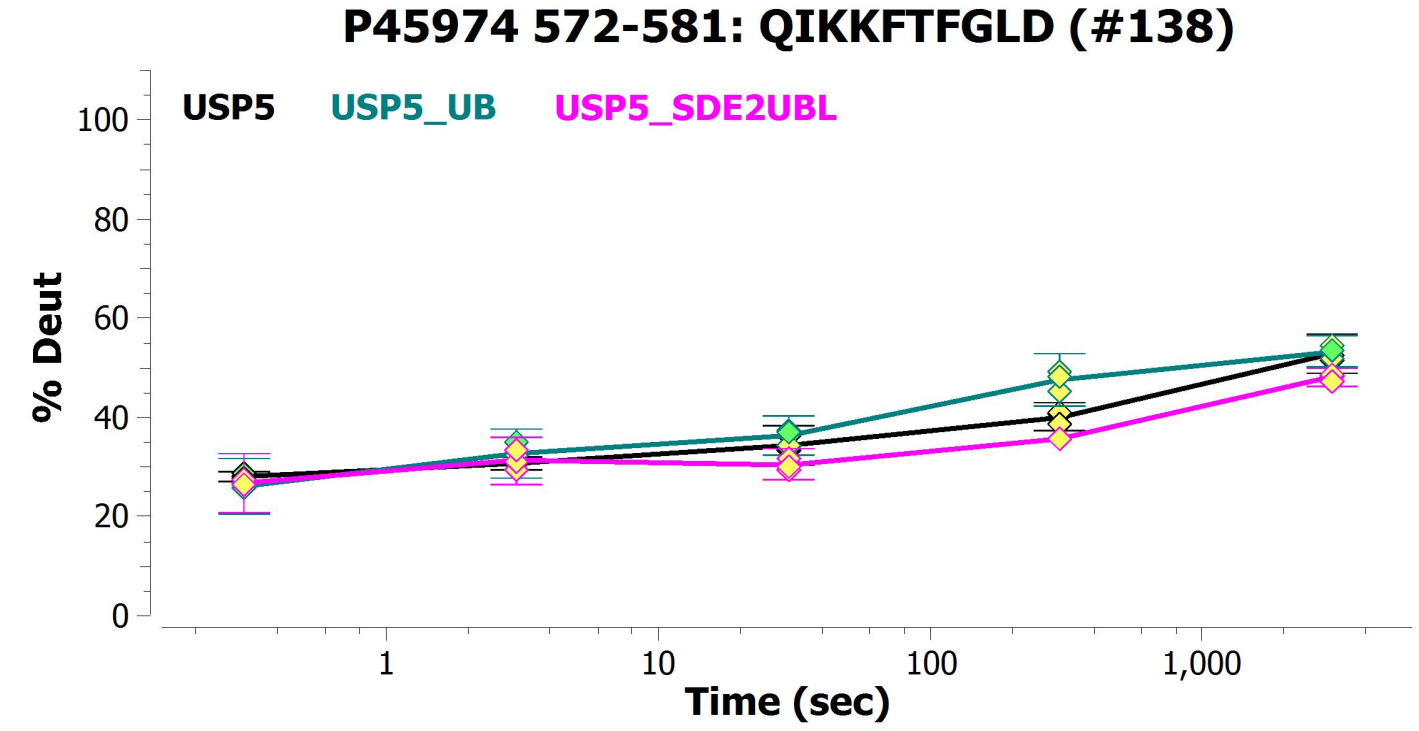

P45974 582-590: WVPKKLDVS (#139)

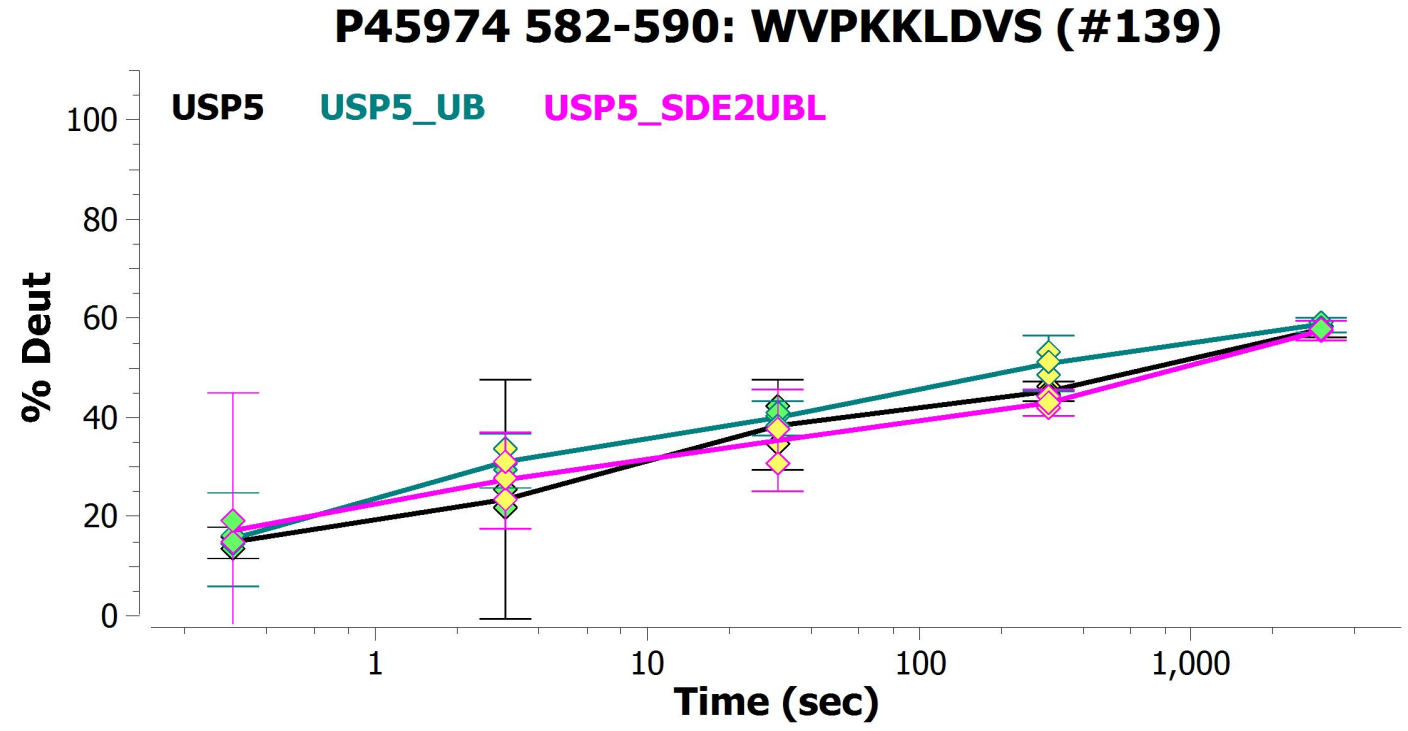

P45974 597-614: LDISQLRGTGLQPGEEEL (#140)

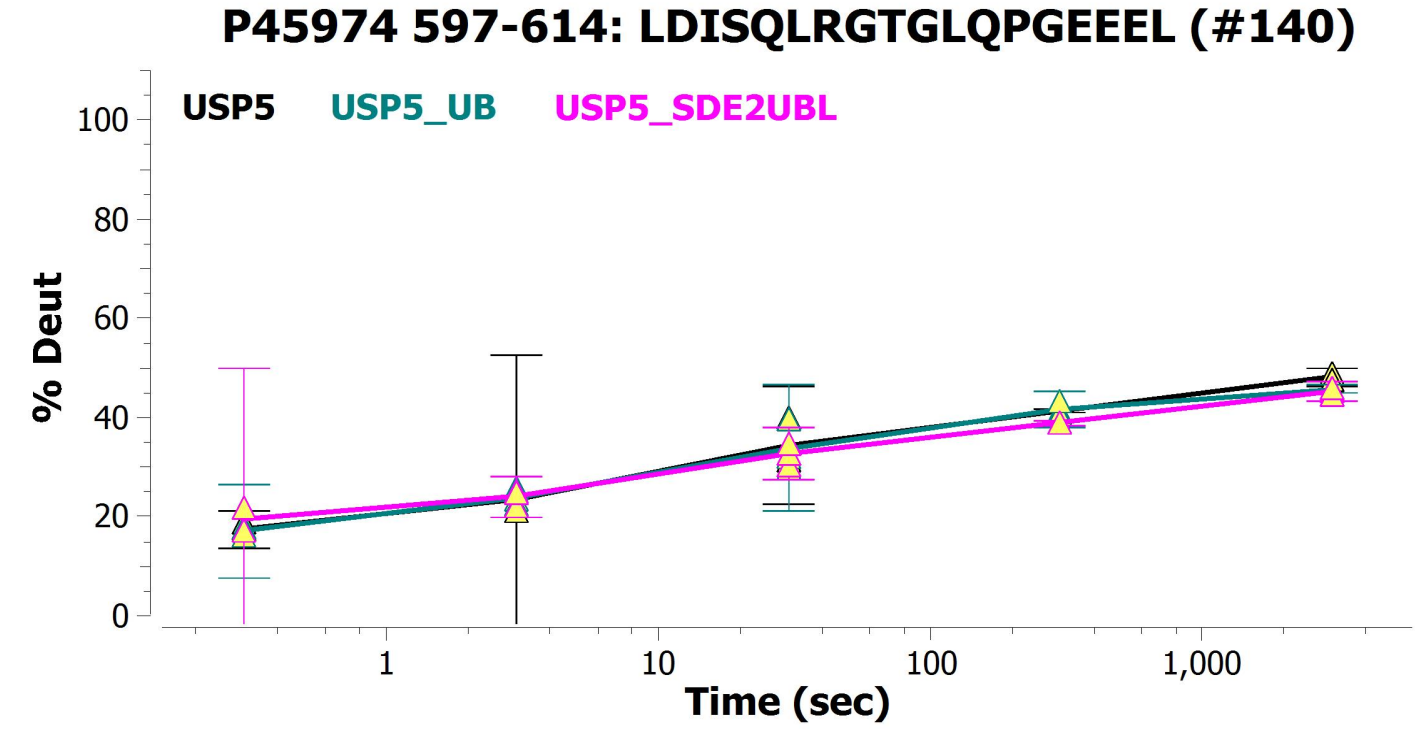

P45974 598-614: DISQLRGTGLQPGEEEL (#141)

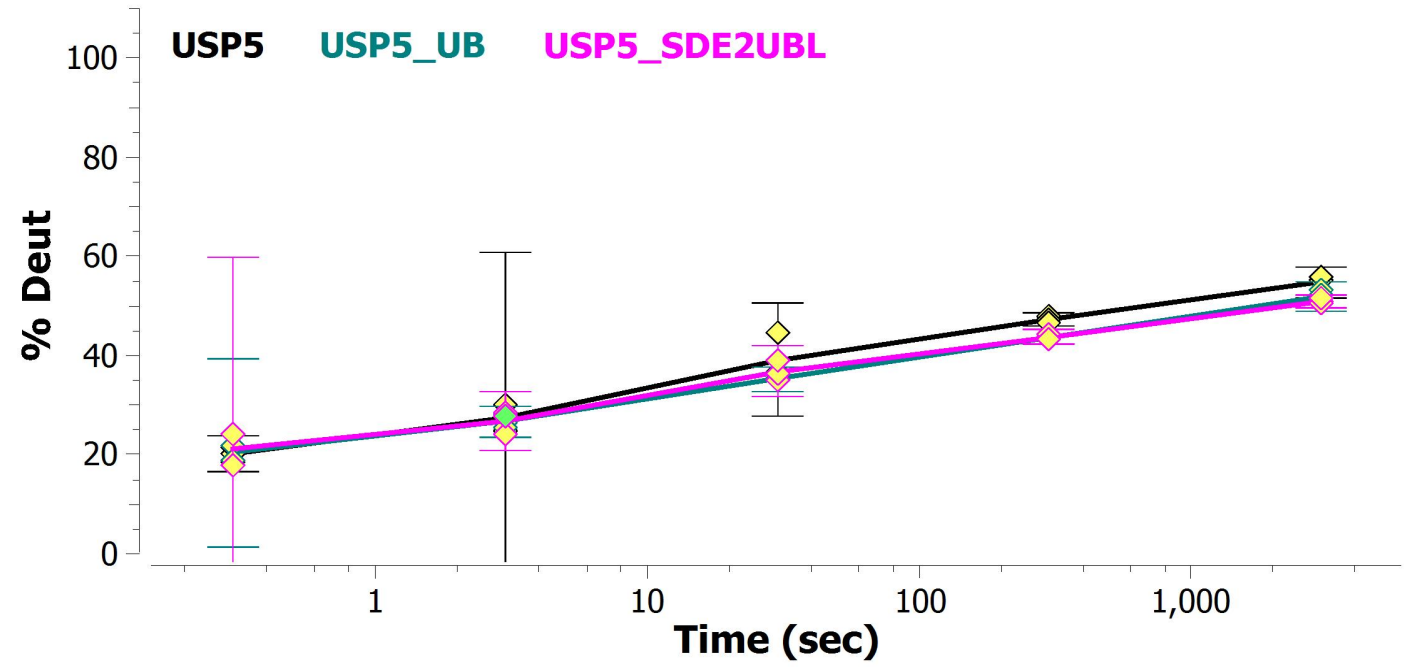

P45974 602-614: LRGTGLQPGEEEL (#142)

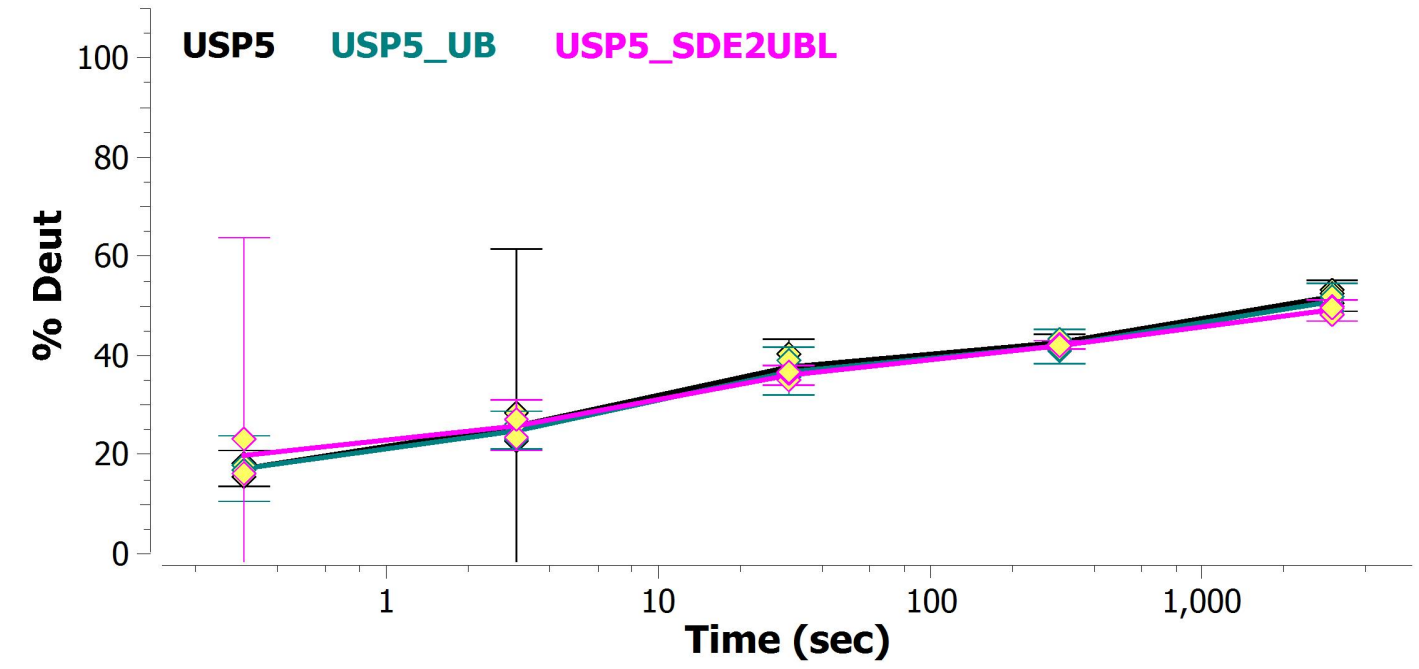

P45974 603-614: RGTGLQPGEEEL (#143)

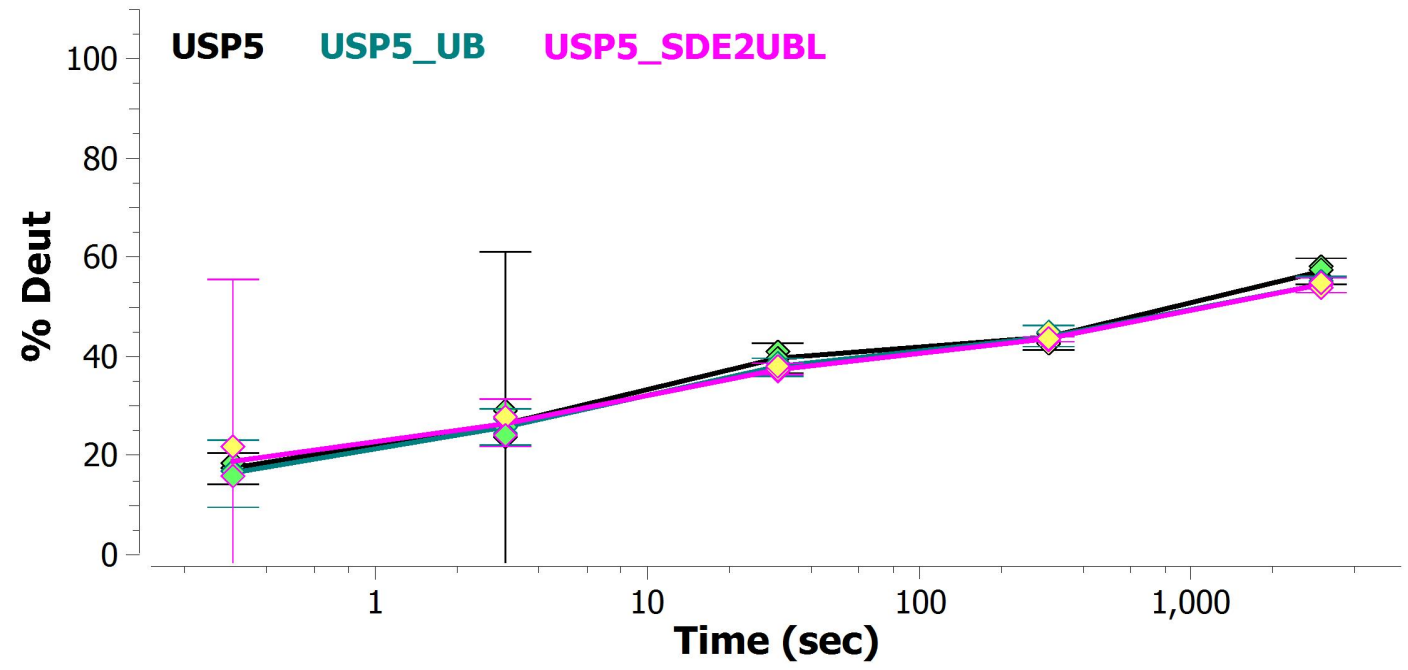

P45974 624-633: PDEPKGSLGF (#144)

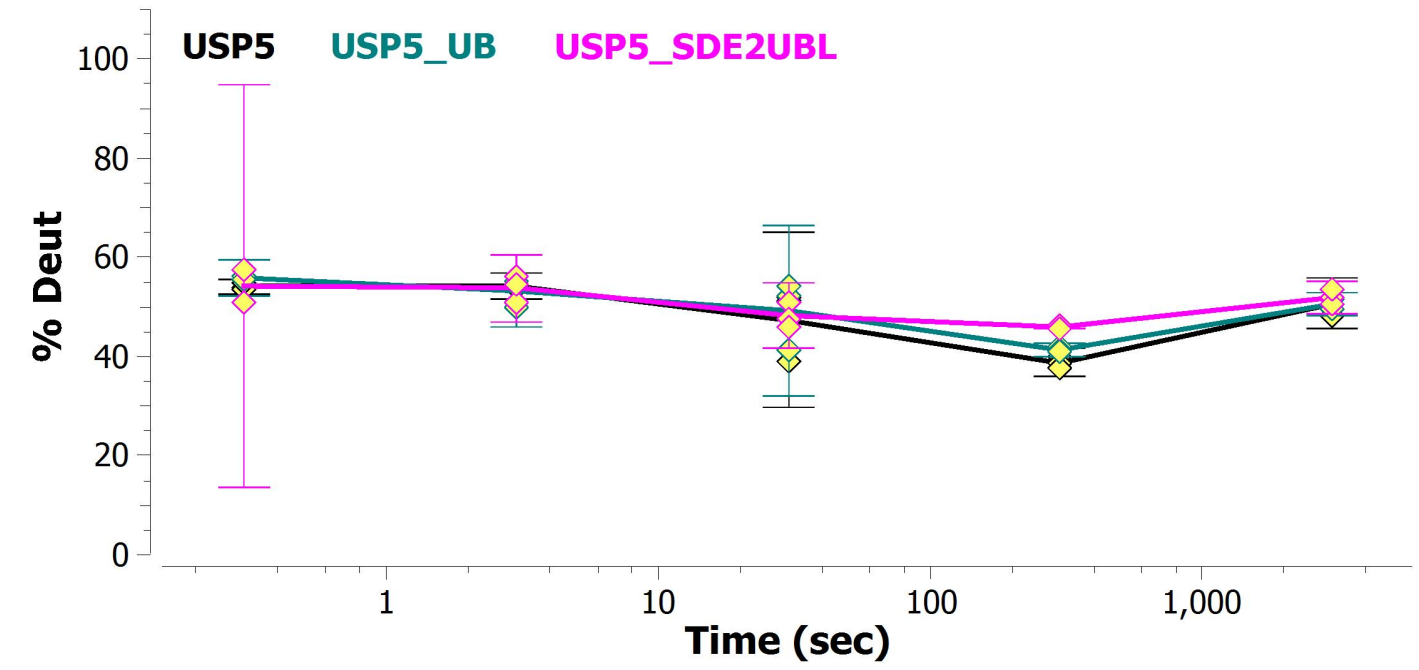

P45974 642-648: FCSPHFS (#145)

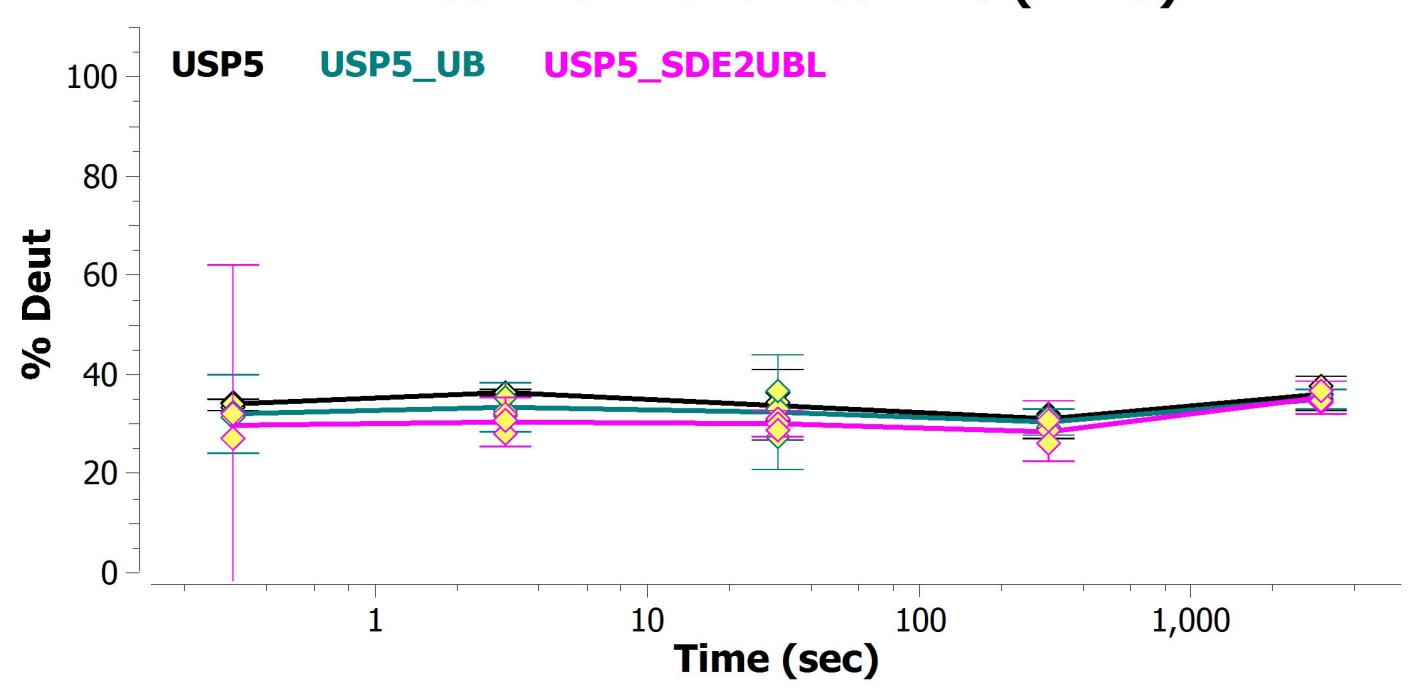

P45974 642-655: FCSPHFSSPTSPML (#146)

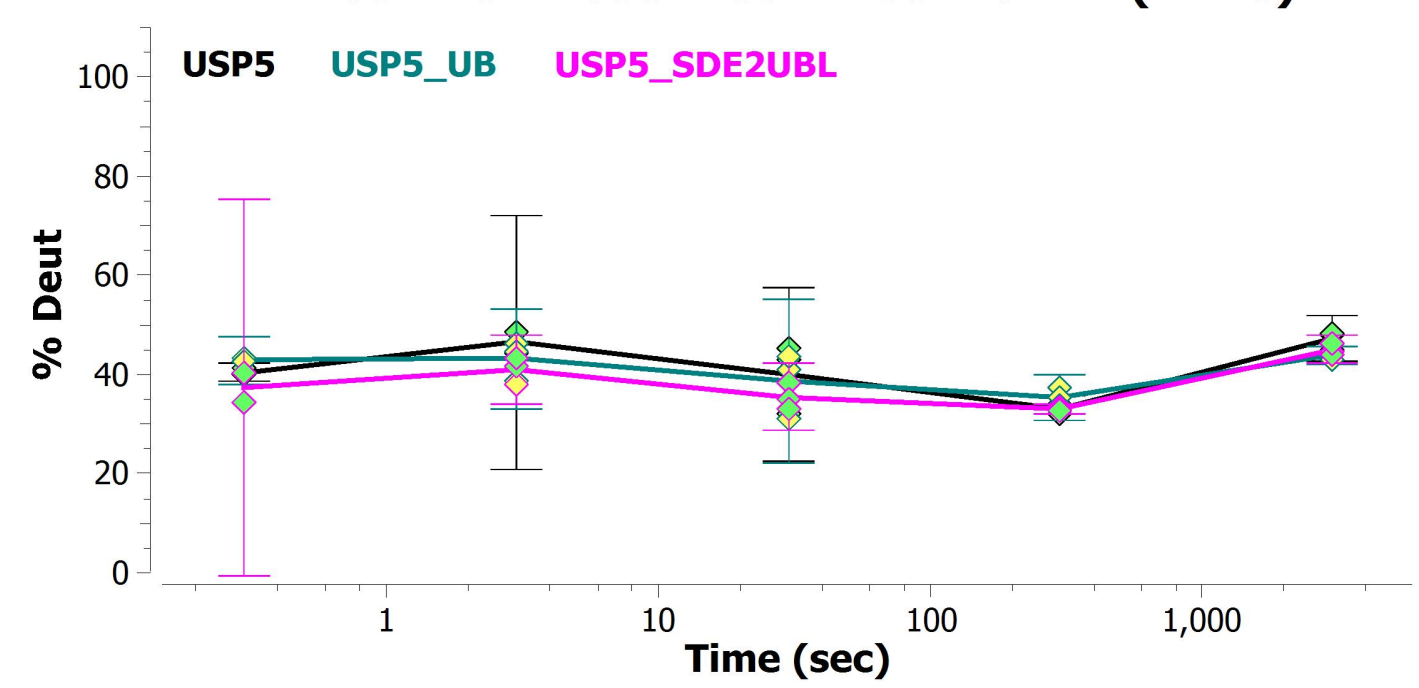

P45974 643-655: CSPHFSSPTSPML (#147)

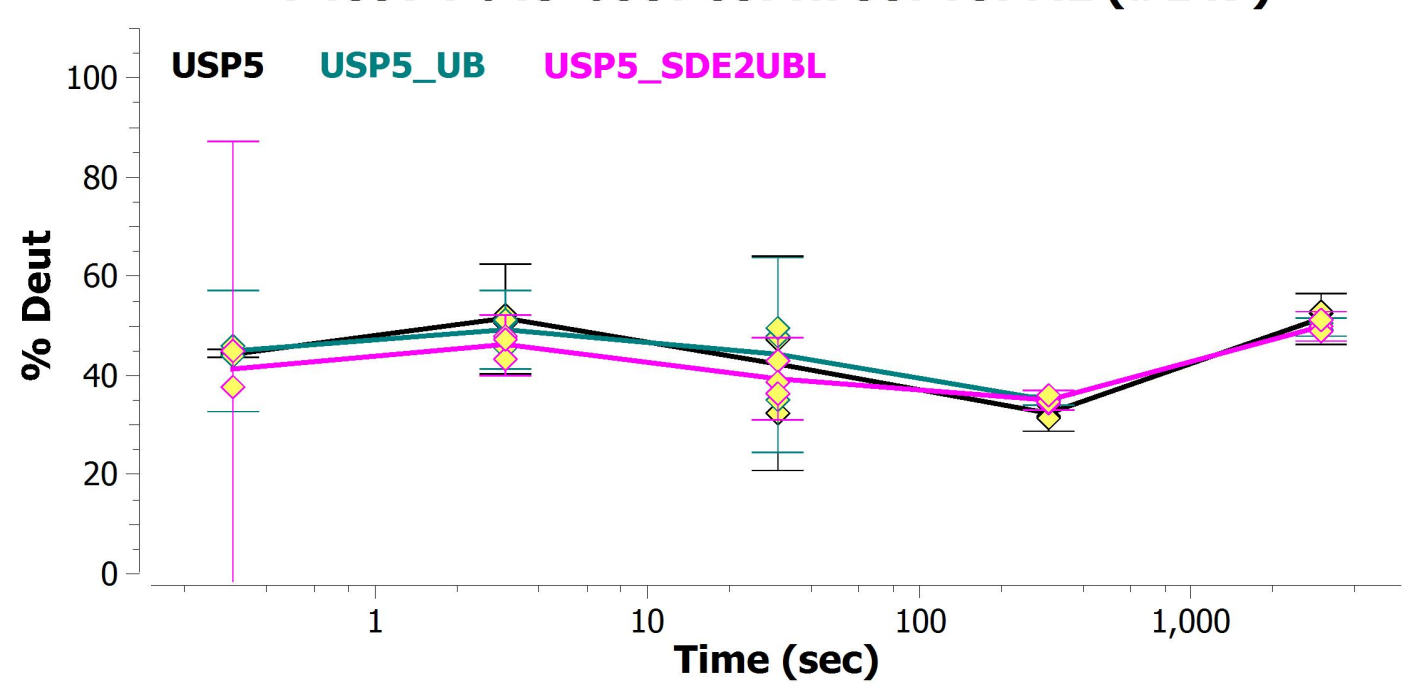

P45974 643-657: CSPHFSSPTSPMLDE (#148)

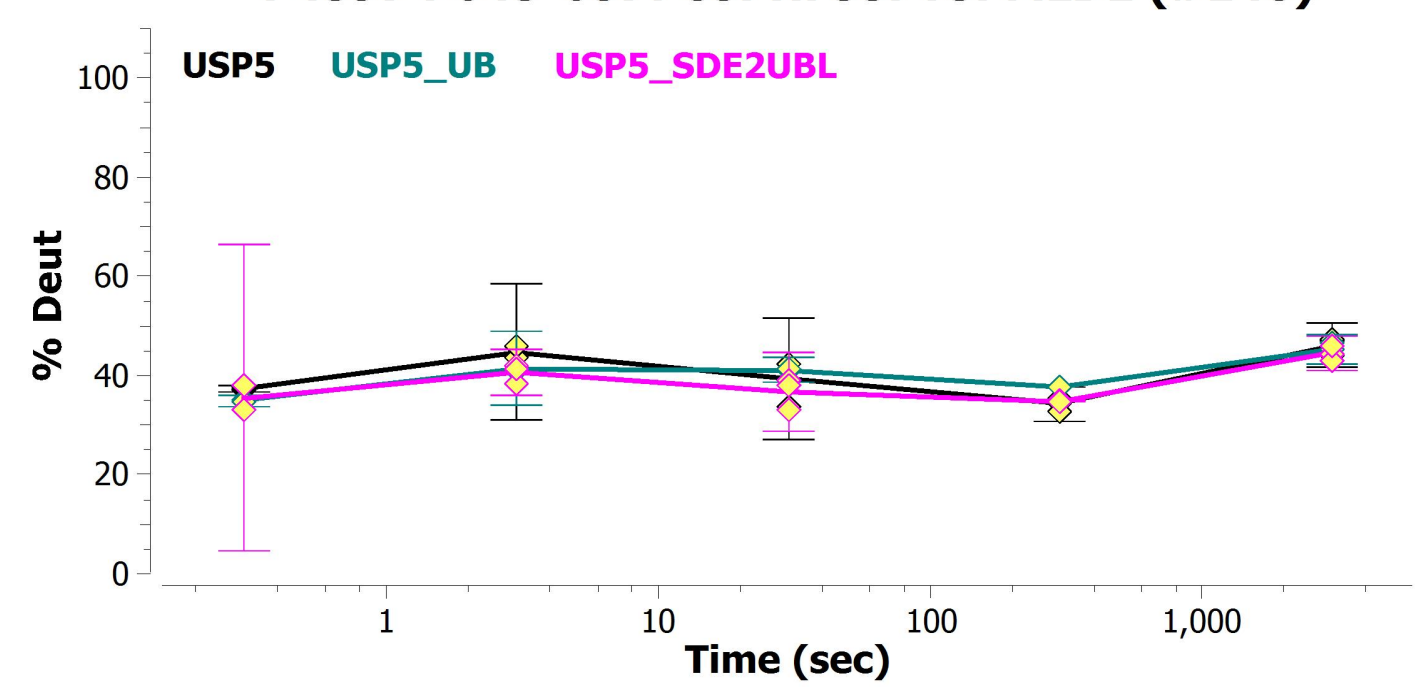

P45974 661-680: IQLVEMGFPMDACRKAVYYT (#149)

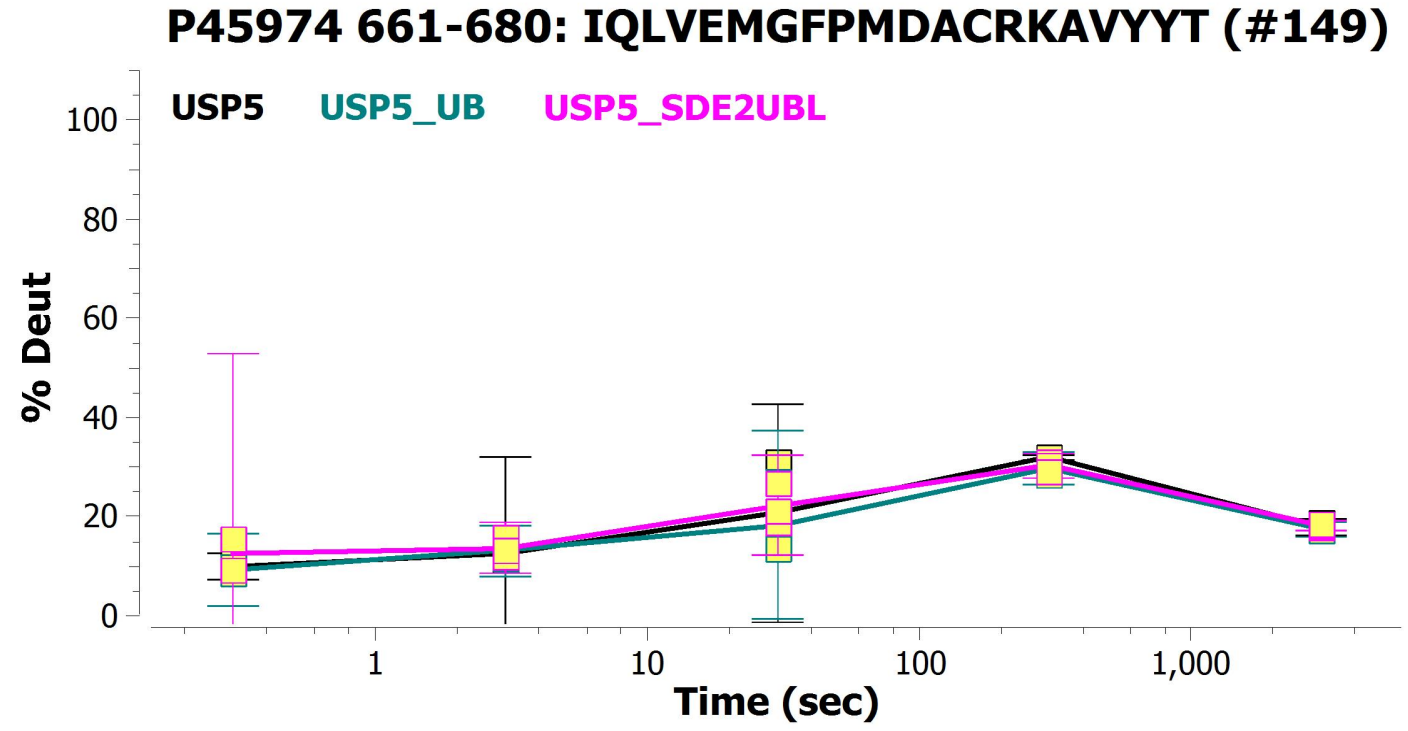

P45974 663-672: LVEMGFPMDA (#150)

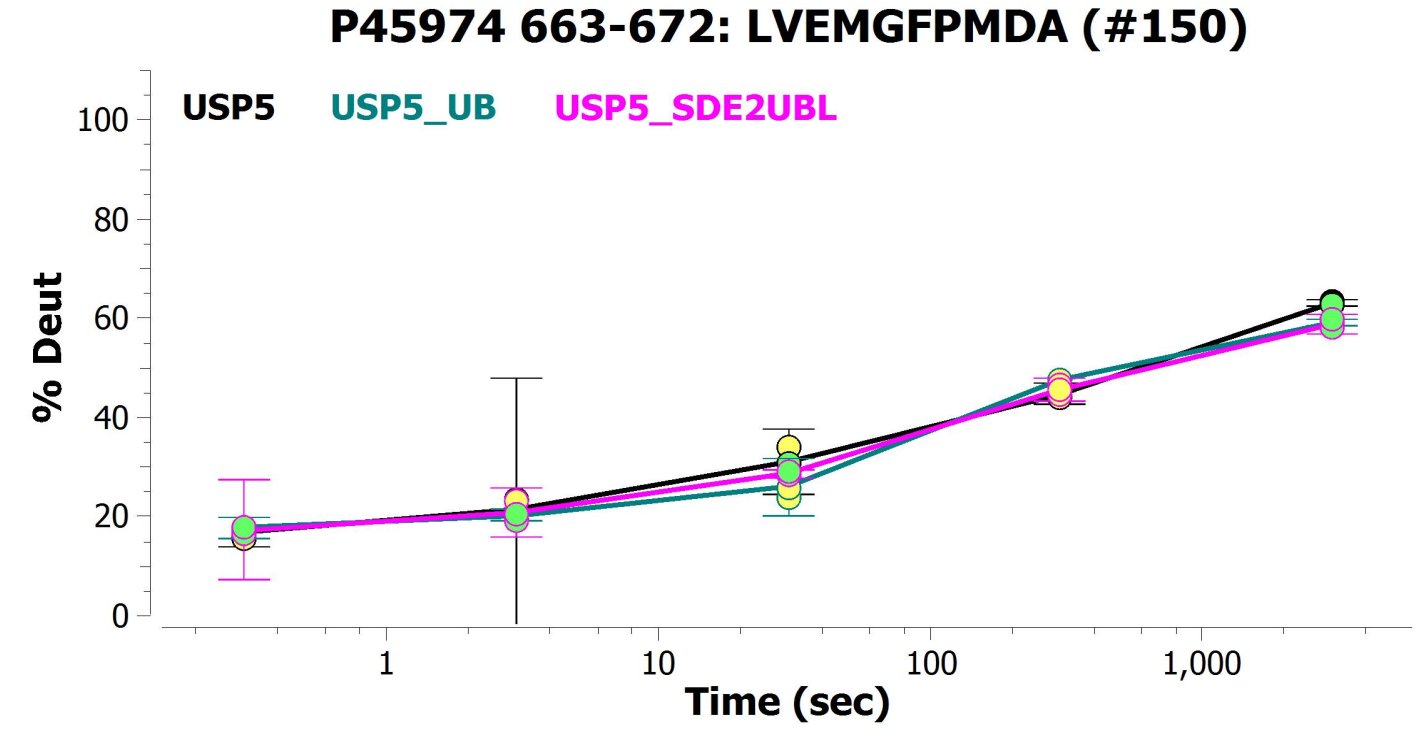

P45974 664-678: VEMGFPMDACRKAVY (#151)

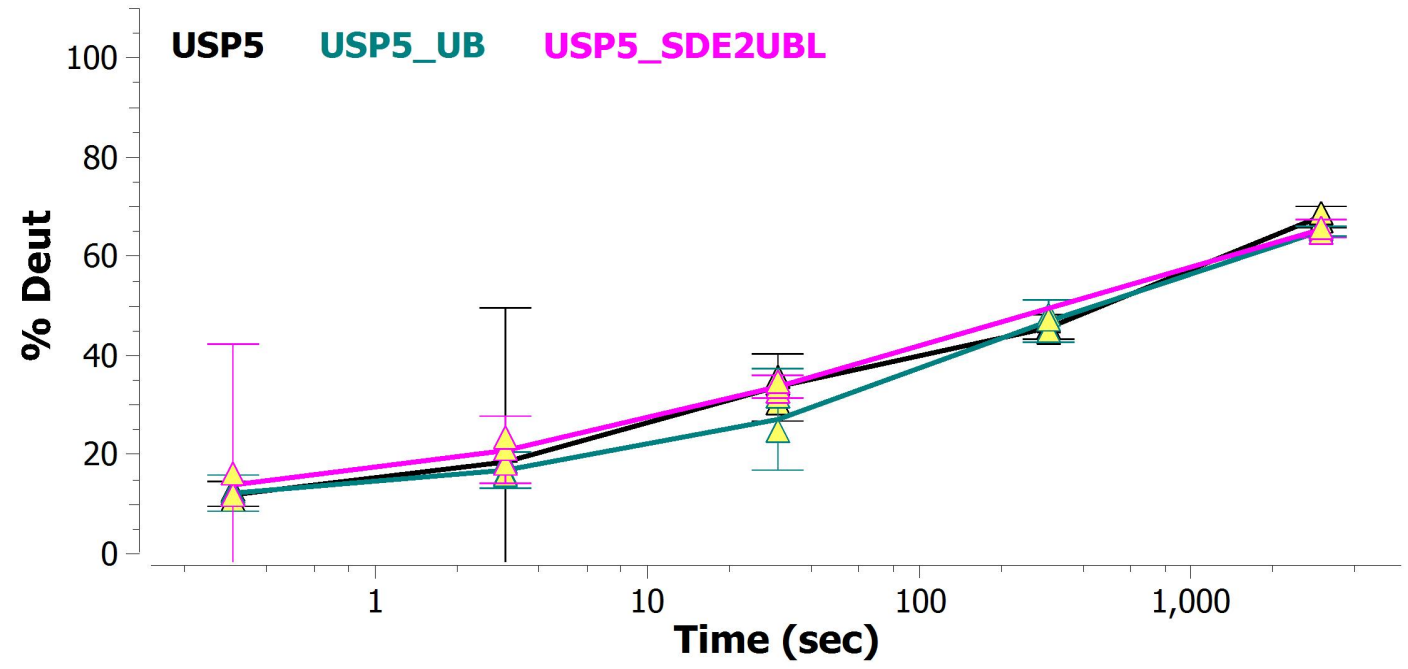

P45974 666-678: MGFPMDACRKAVY (#152)

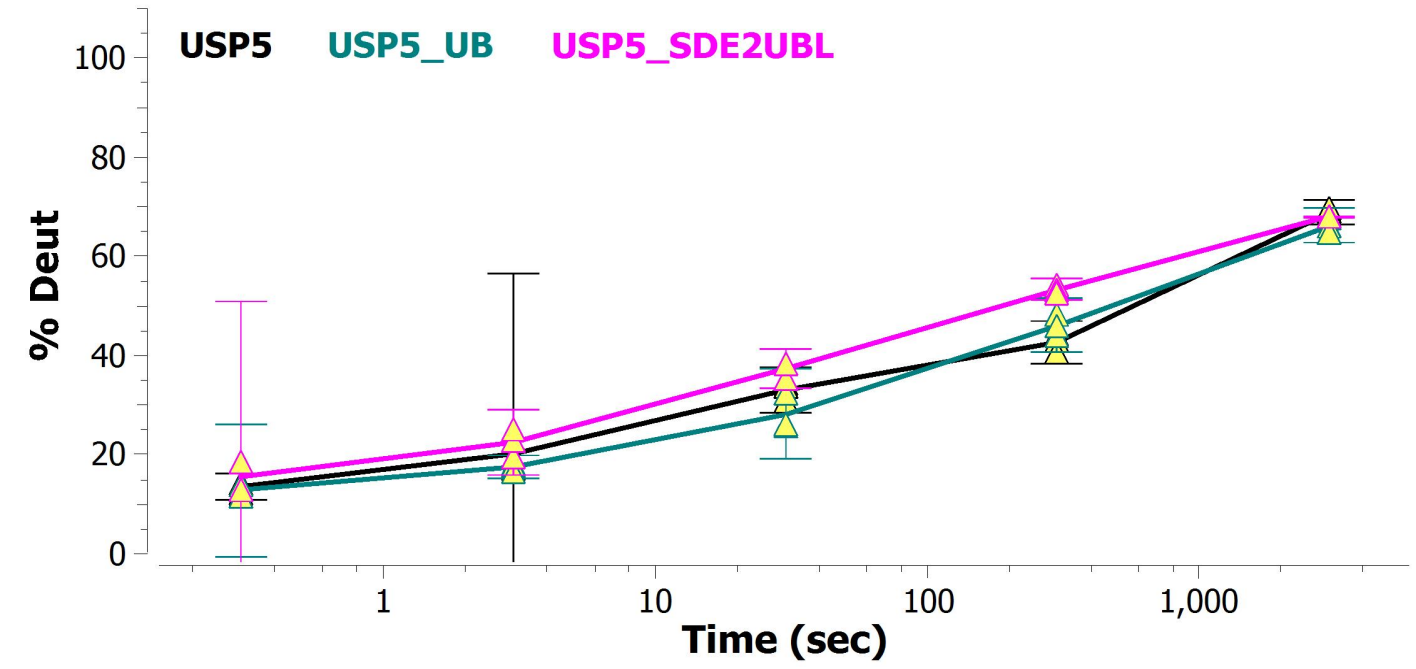

P45974 673-689: CRKAVYYTGNSGAEAAM (#153)

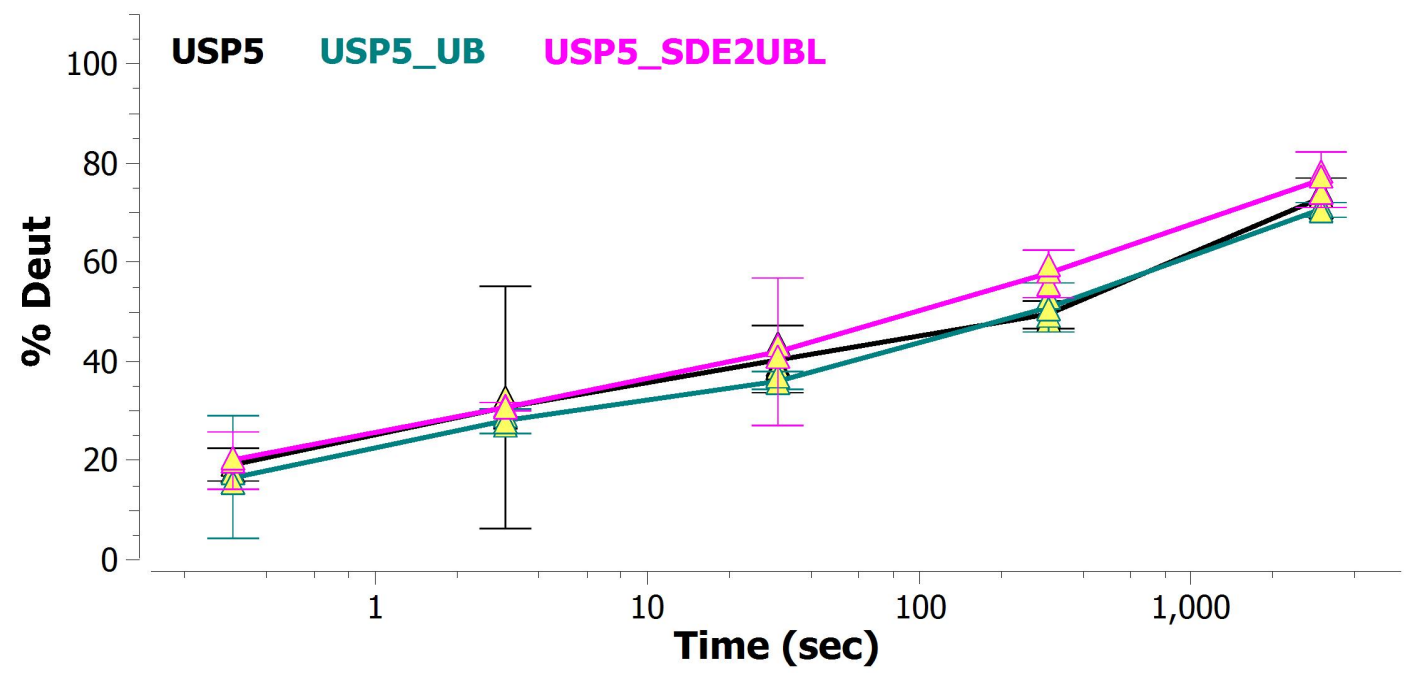

P45974 673-690: CRKAVYYTGNSGAEAAMN (#154)

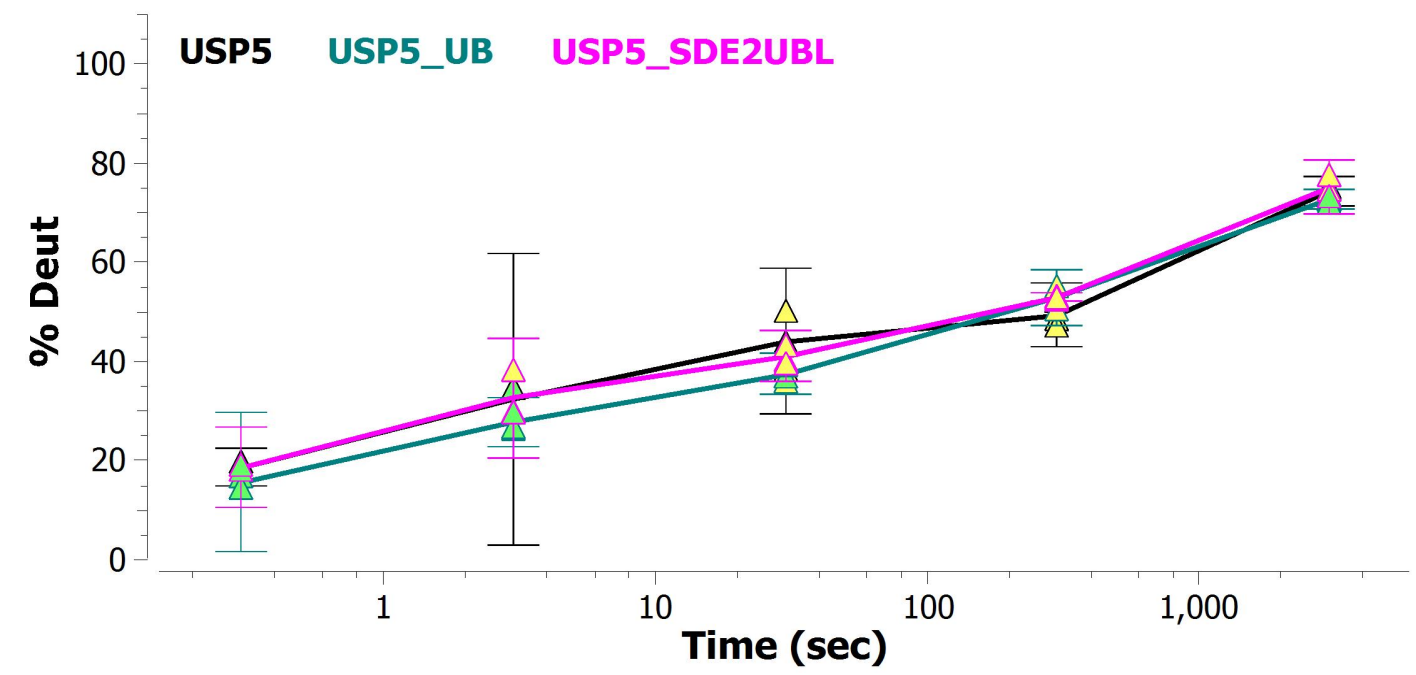

P45974 691-701: WVMSHMDDPDF (#155)

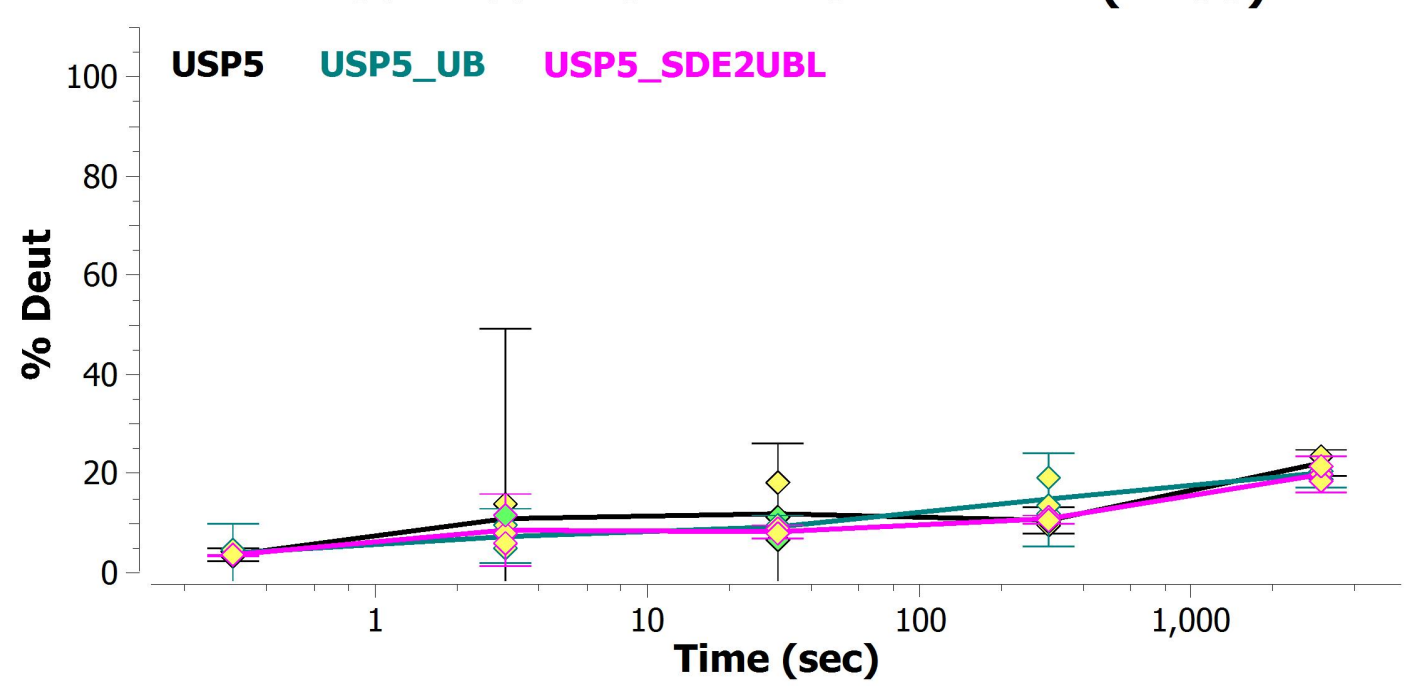

P45974 730-735: TIVSMG (#156)

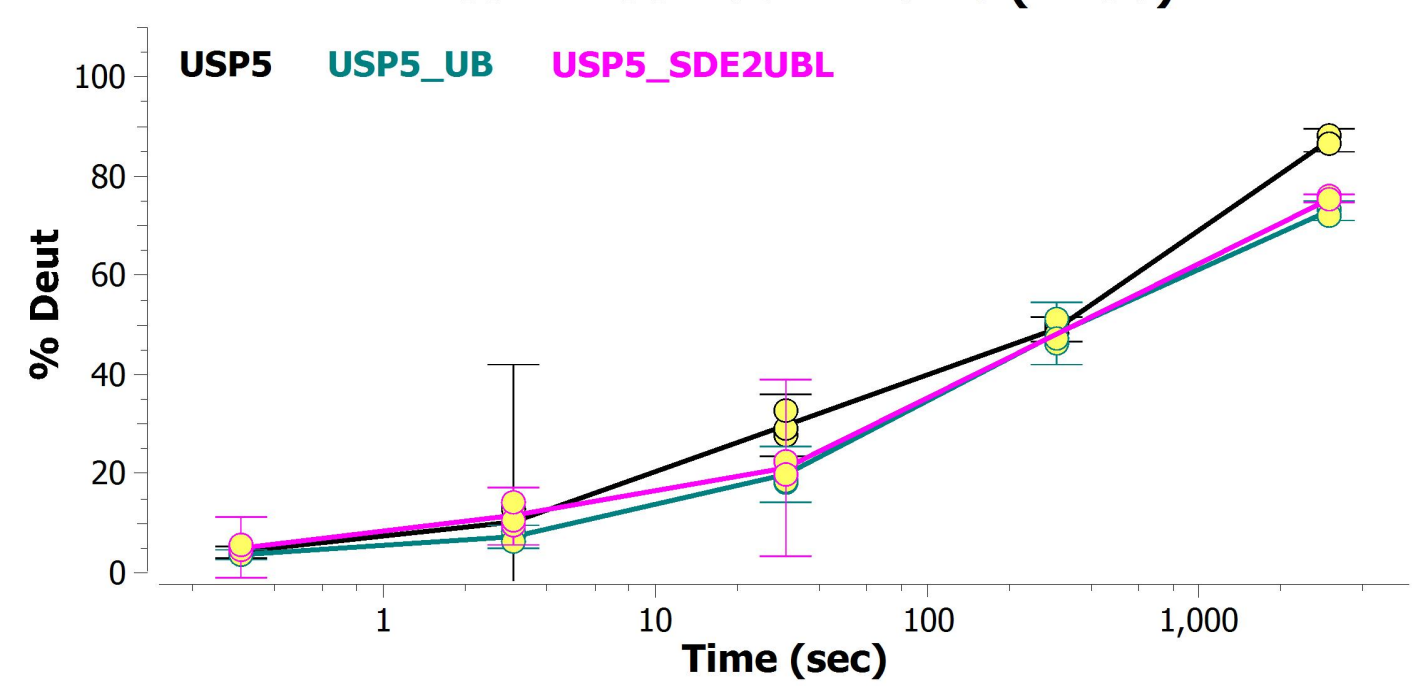

P45974 730-744: TIVSMGFSRDQALKA (#157)

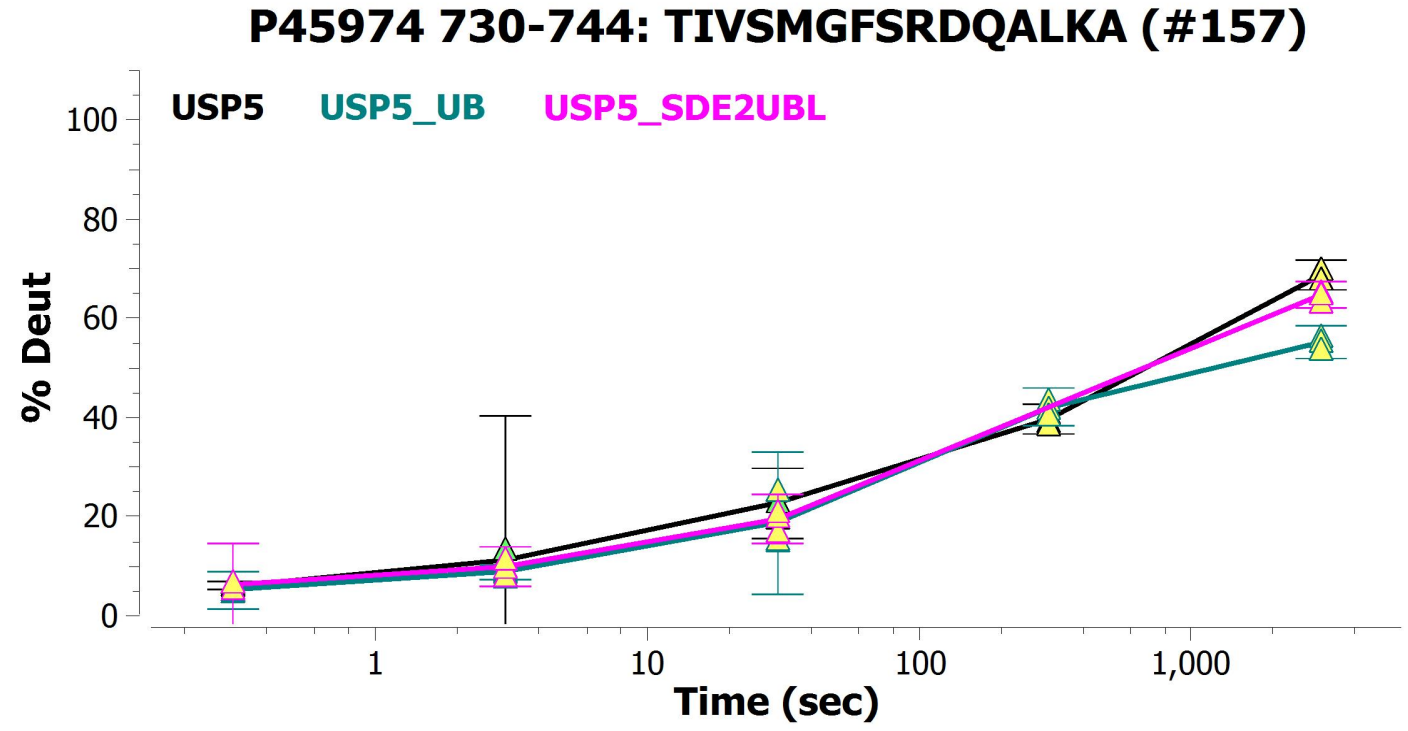

P45974 730-752: TIVSMGFSRDQALKALRATNNSL (#158)

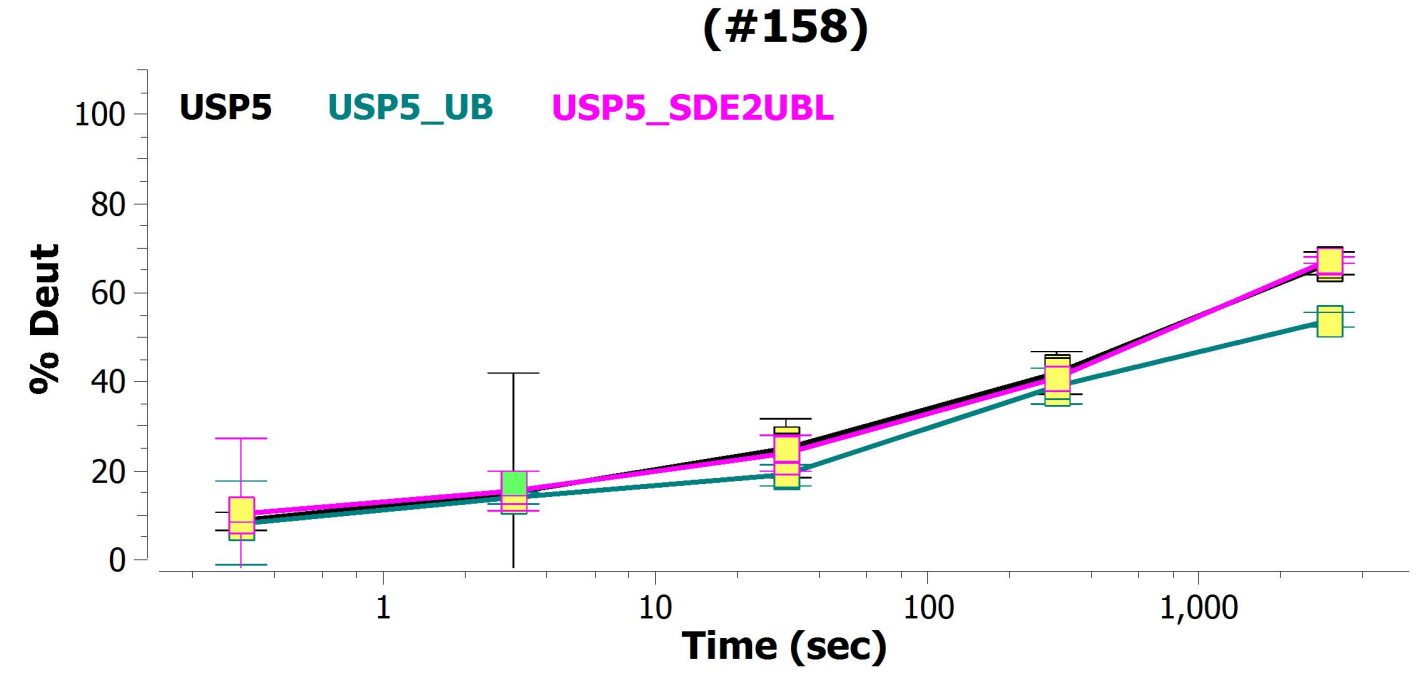

P45974 731-744: IVSMGFSRDQALKA (#159)

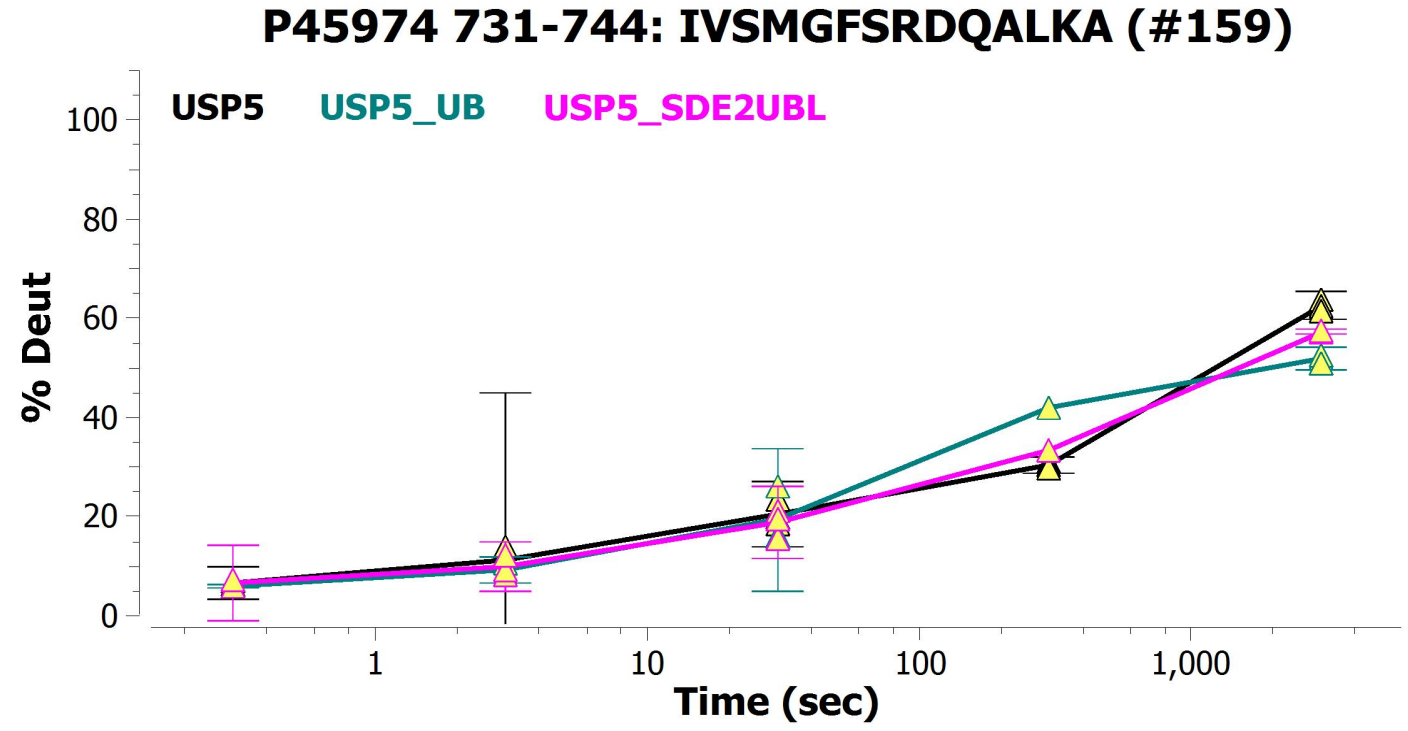

P45974 736-744: FSRDQALKA (#160)

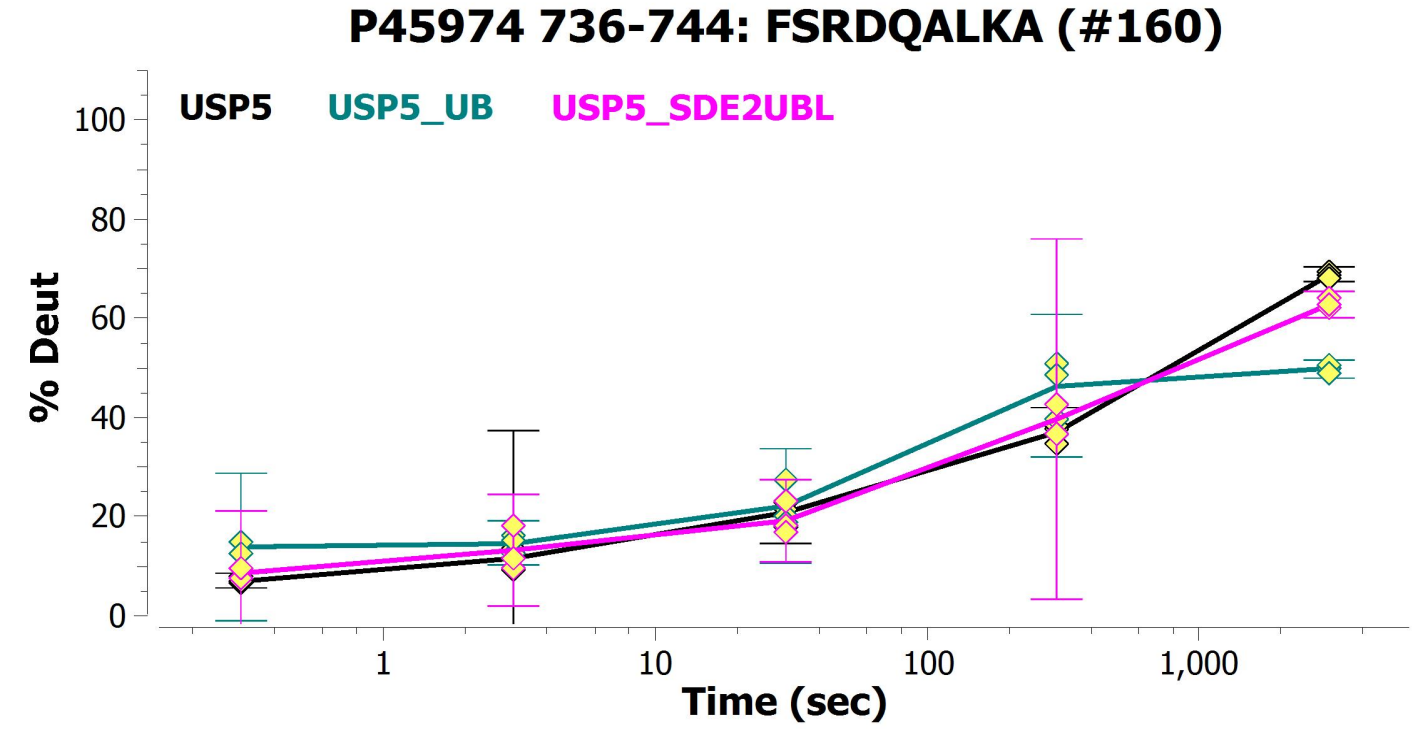

P45974 752-760: LERAVDWIF (#161)

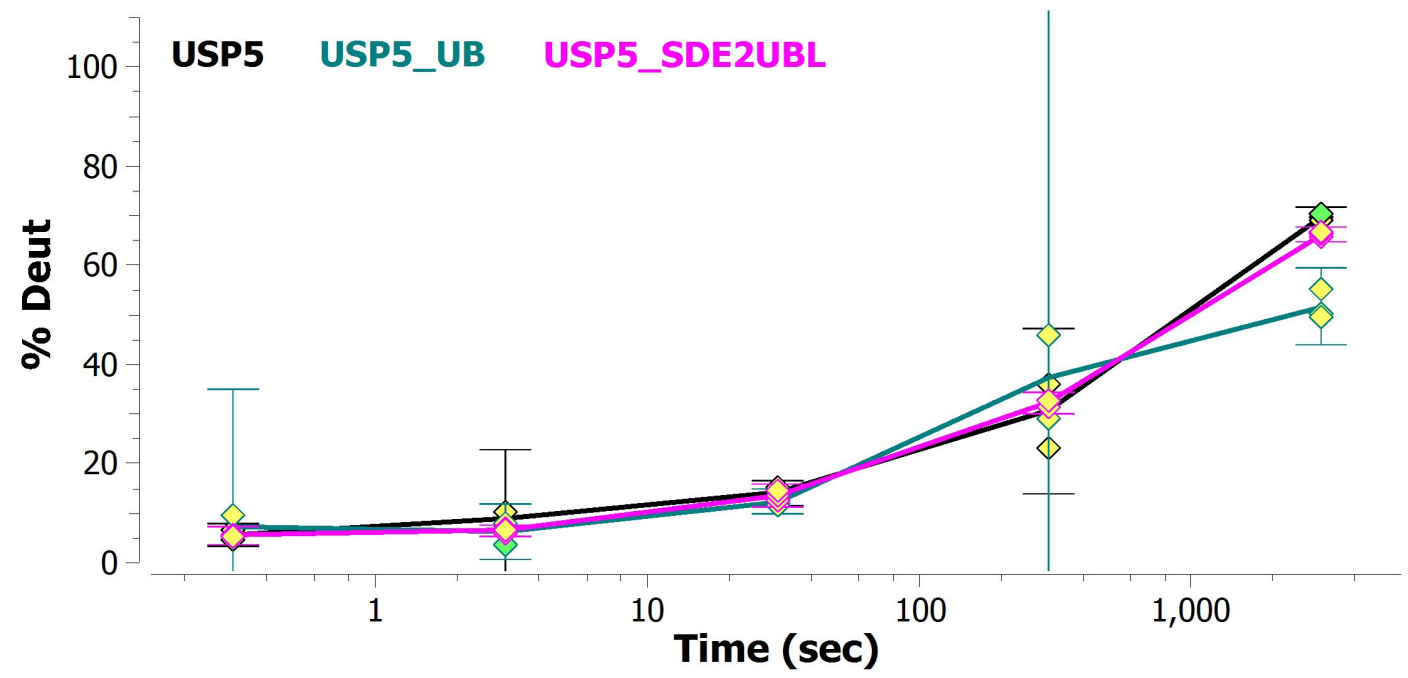

P45974 753-760: ERAVDWIF (#162)

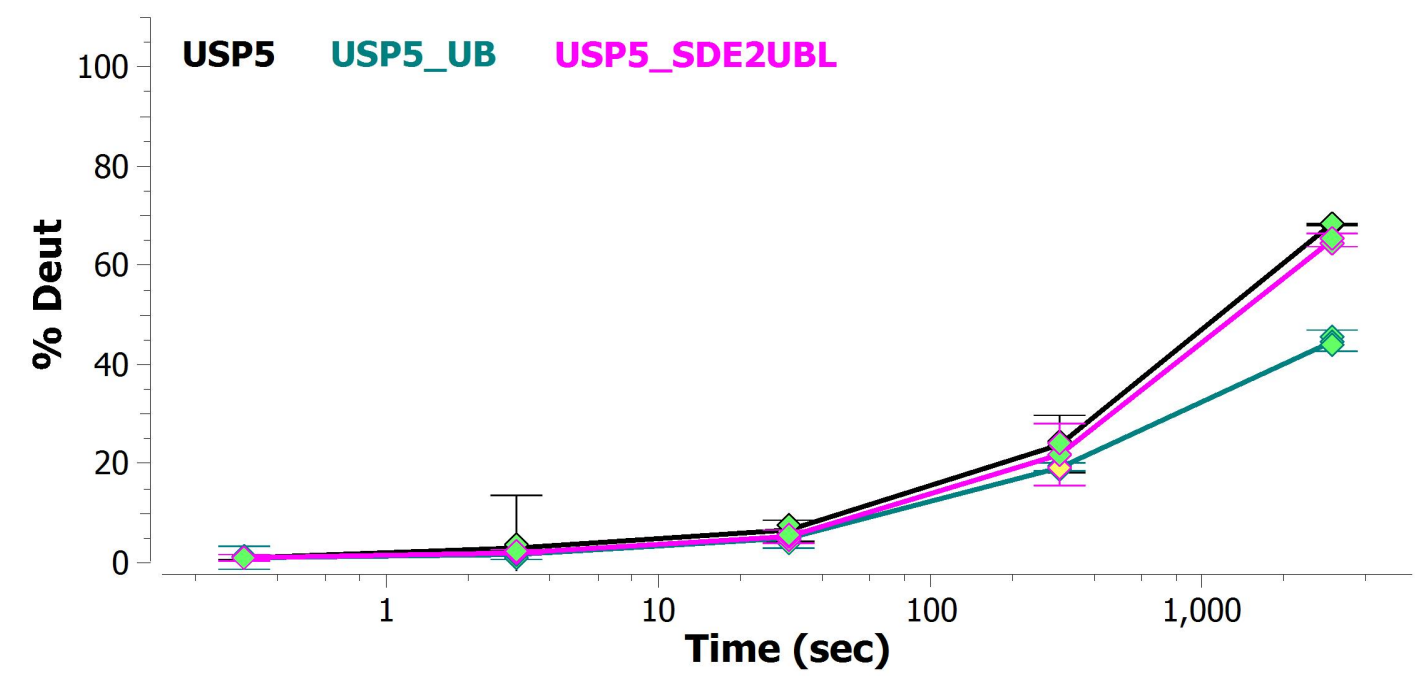

P45974 774-803: ISEGRSAADSISESVPVGPKVRDGPGKYQL (#163)

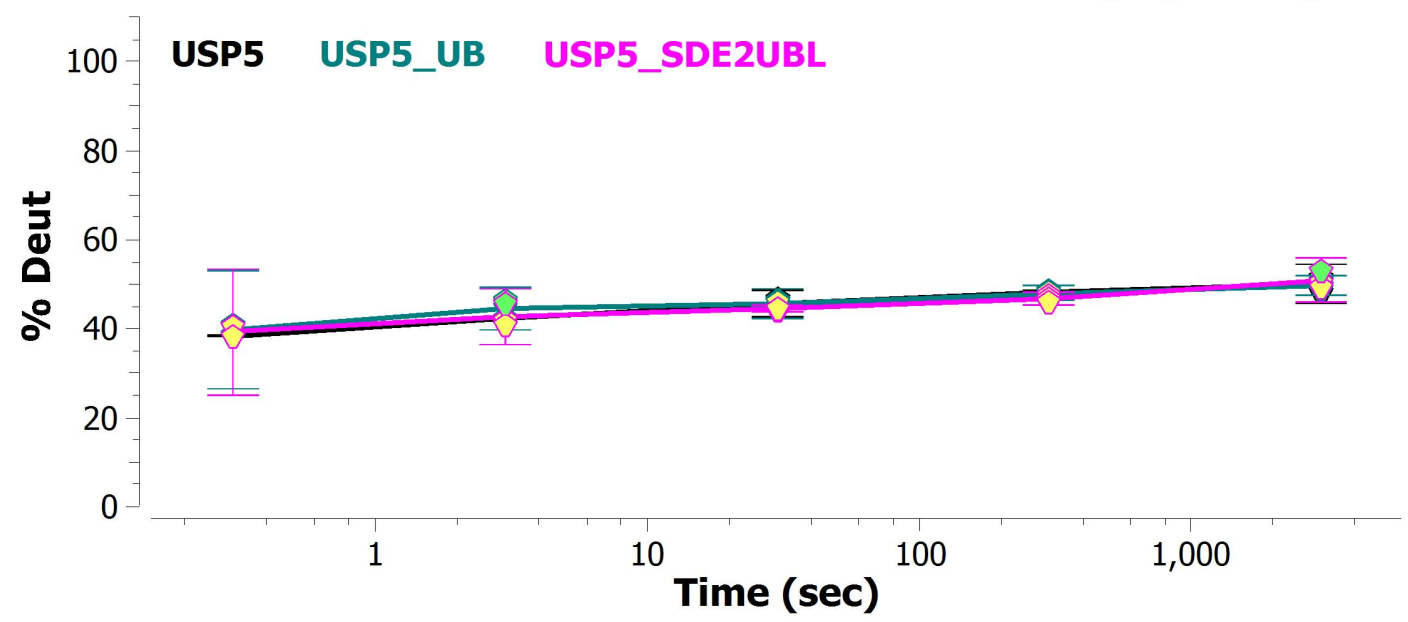

P45974 777-805: GRSAADSISESVPVGPKVRDGPGKYQLFA (#164)

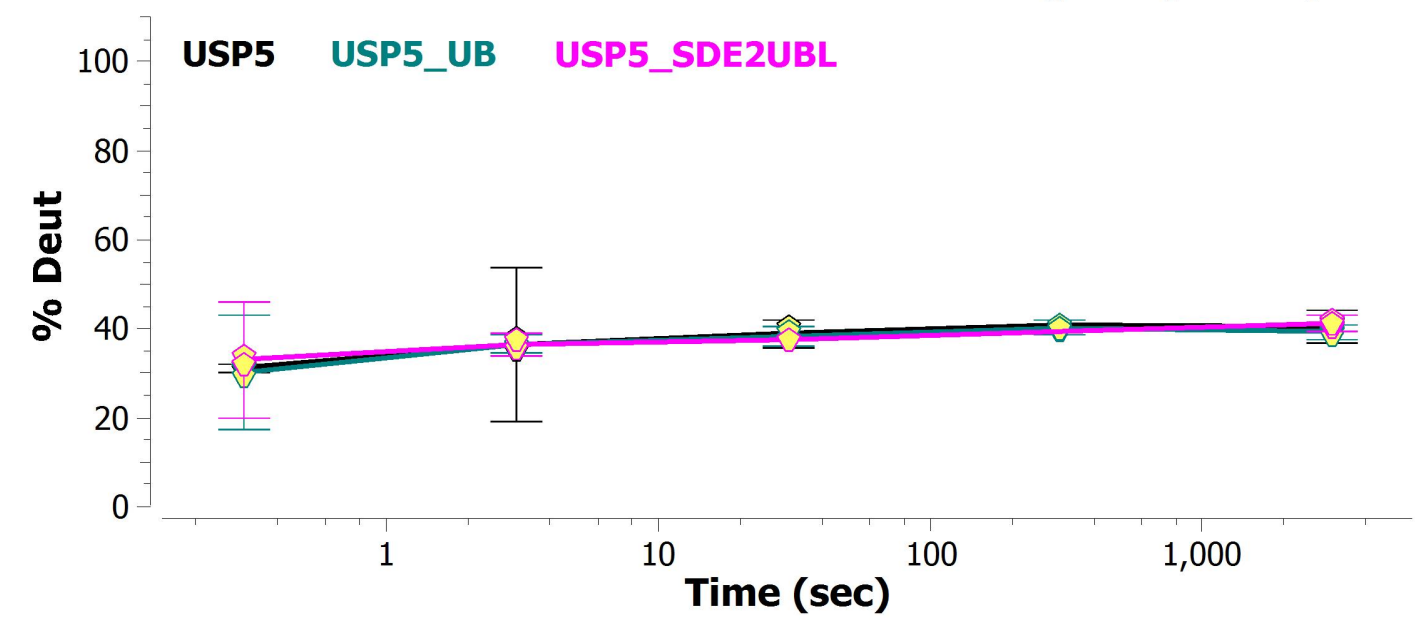

P45974 782-803: DSISESVPVGPKVRDGPGKYQL (#165)

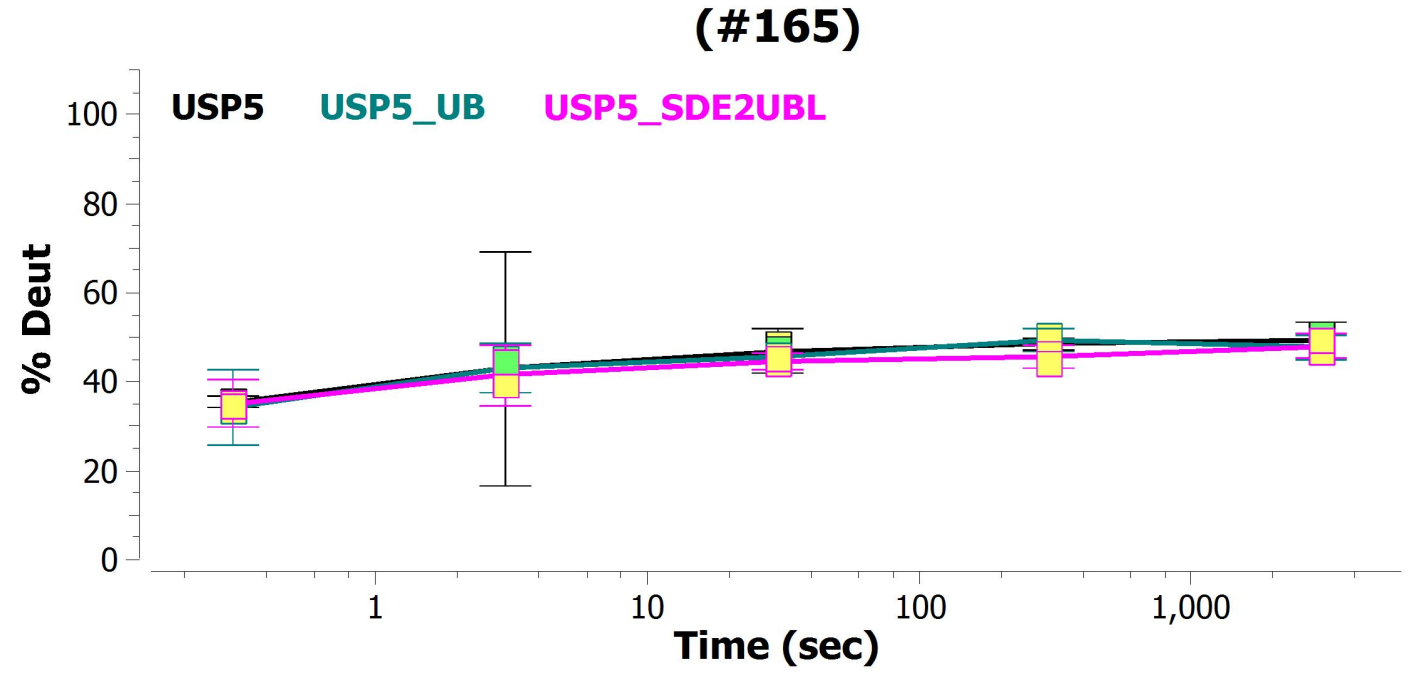

P45974 782-804: DSISESVPVGPKVRDGPGKYQLF (#166)

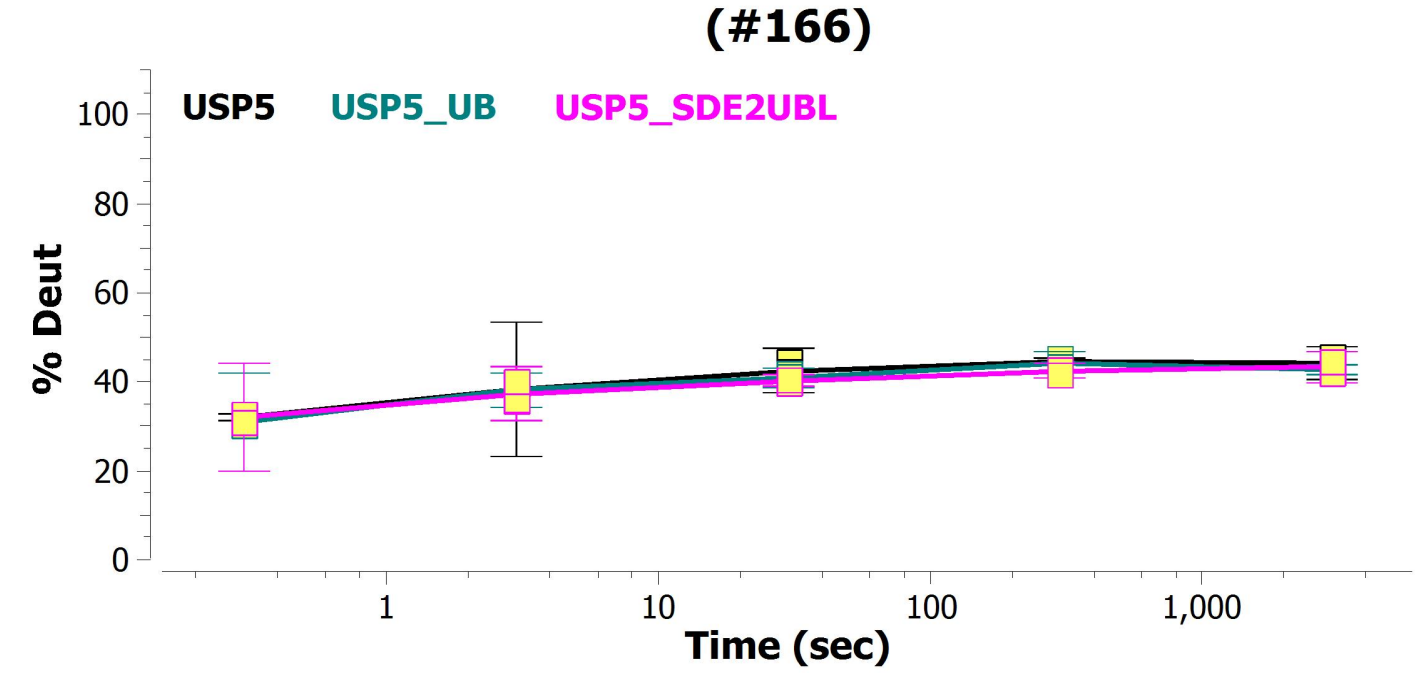

P45974 783-803: SISESVPVGPKVRDGPGKYQL (#167)

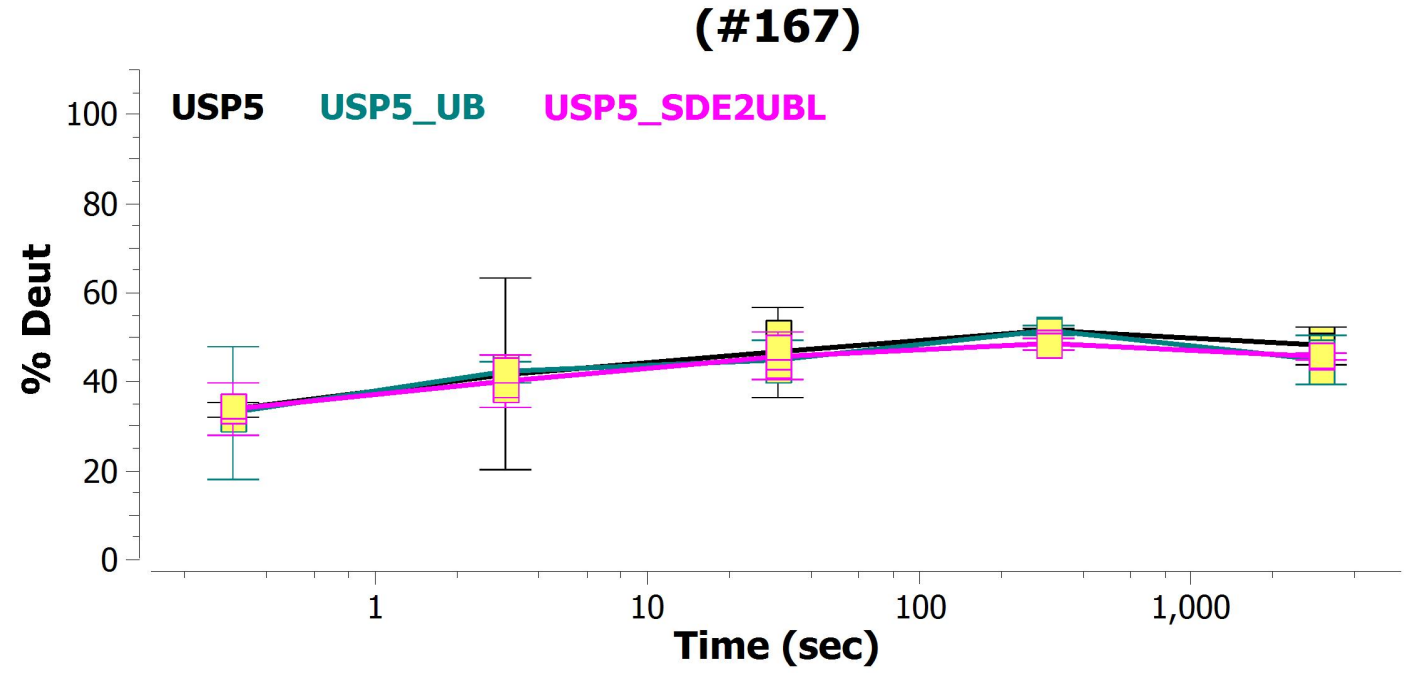

P45974 784-803: ISESVPVGPKVRDGPGKYQL (#168)

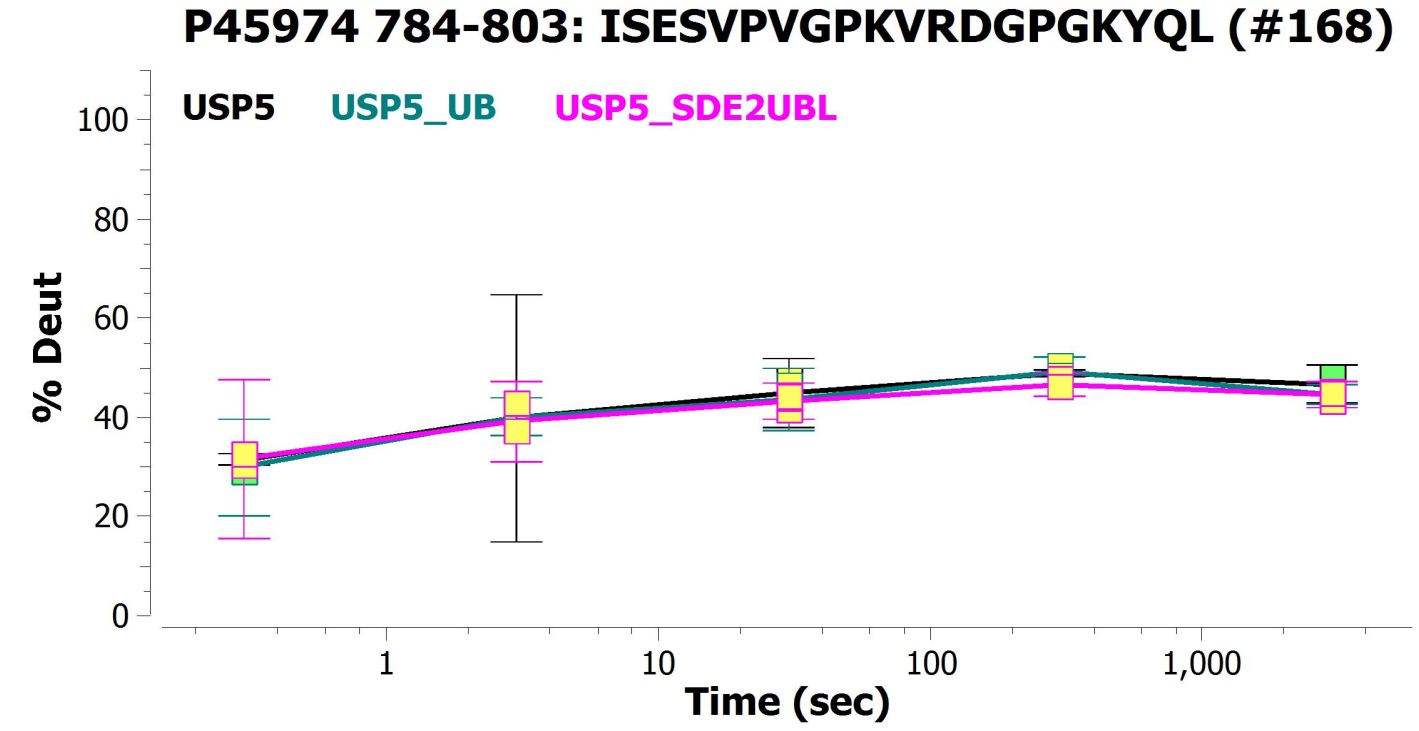

P45974 785-803: SESVPVGPKVRDGPGKYQL (#169)

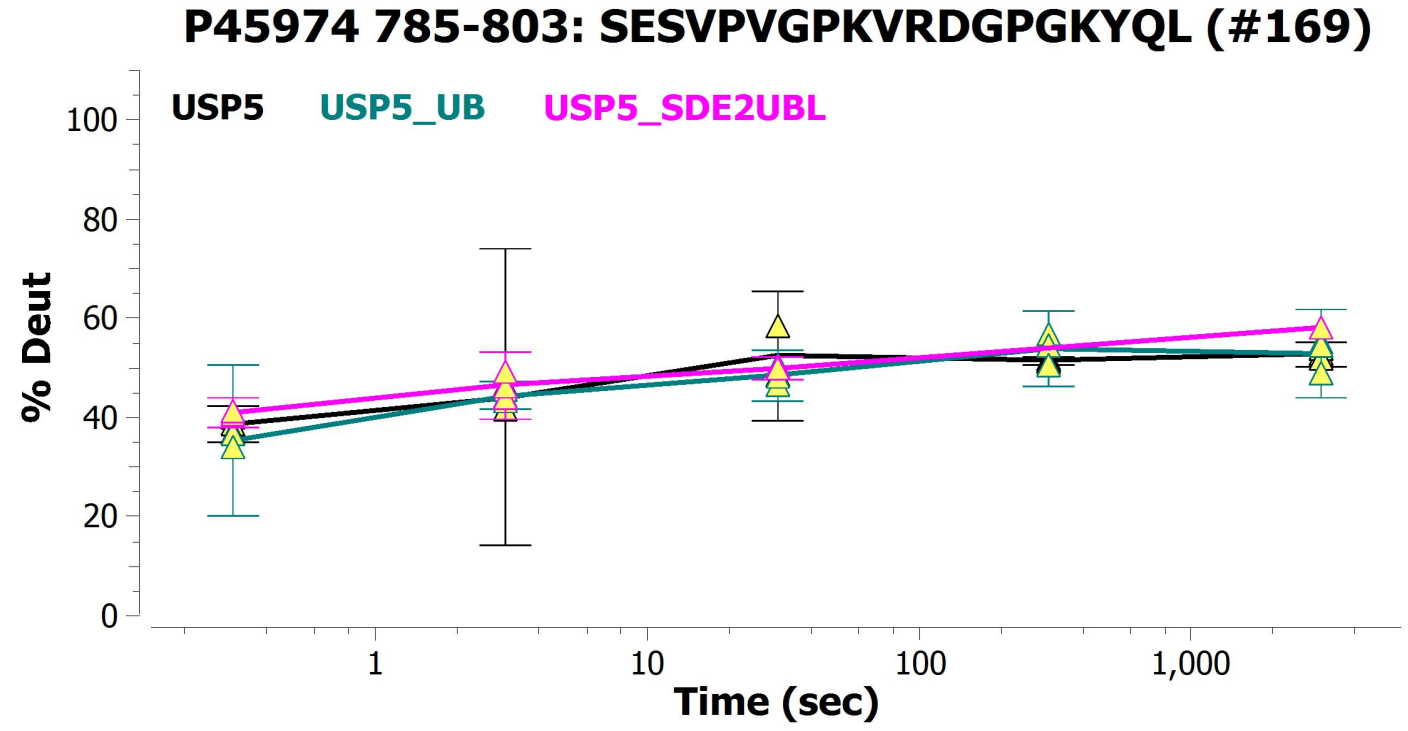

P45974 786-800: ESVPVGPKVRDGPGK (#170)

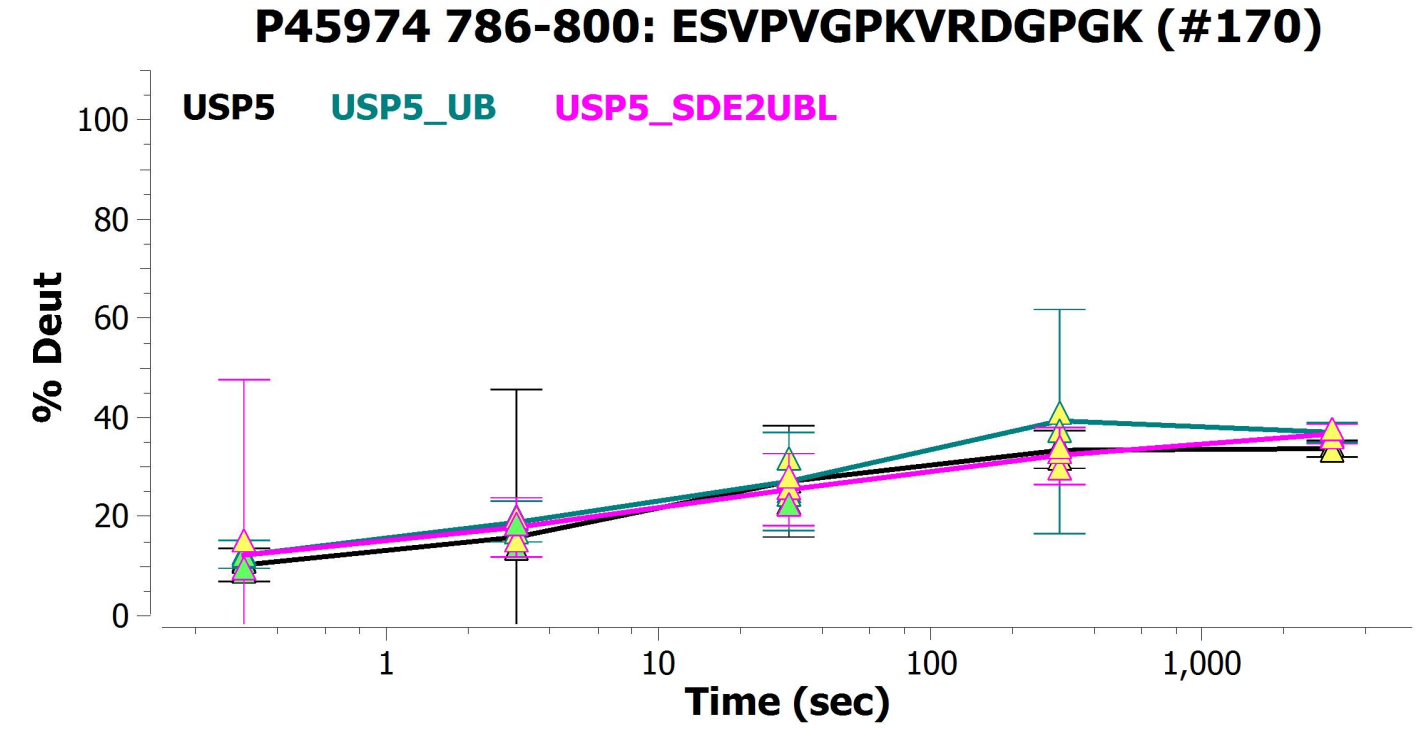

P45974 786-803: ESVPVGPKVRDGPGKYQL (#171)

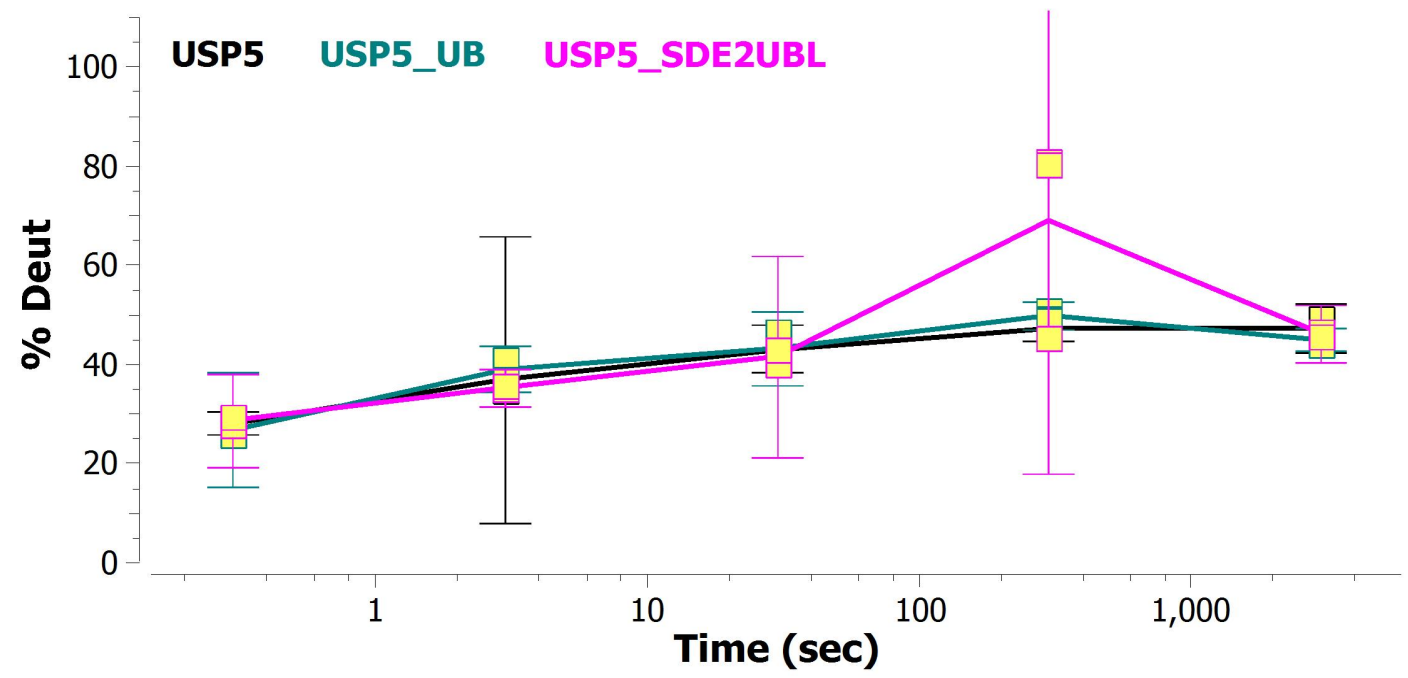

P45974 787-805: SVPVGPKVRDGPGKYQLFA (#172)

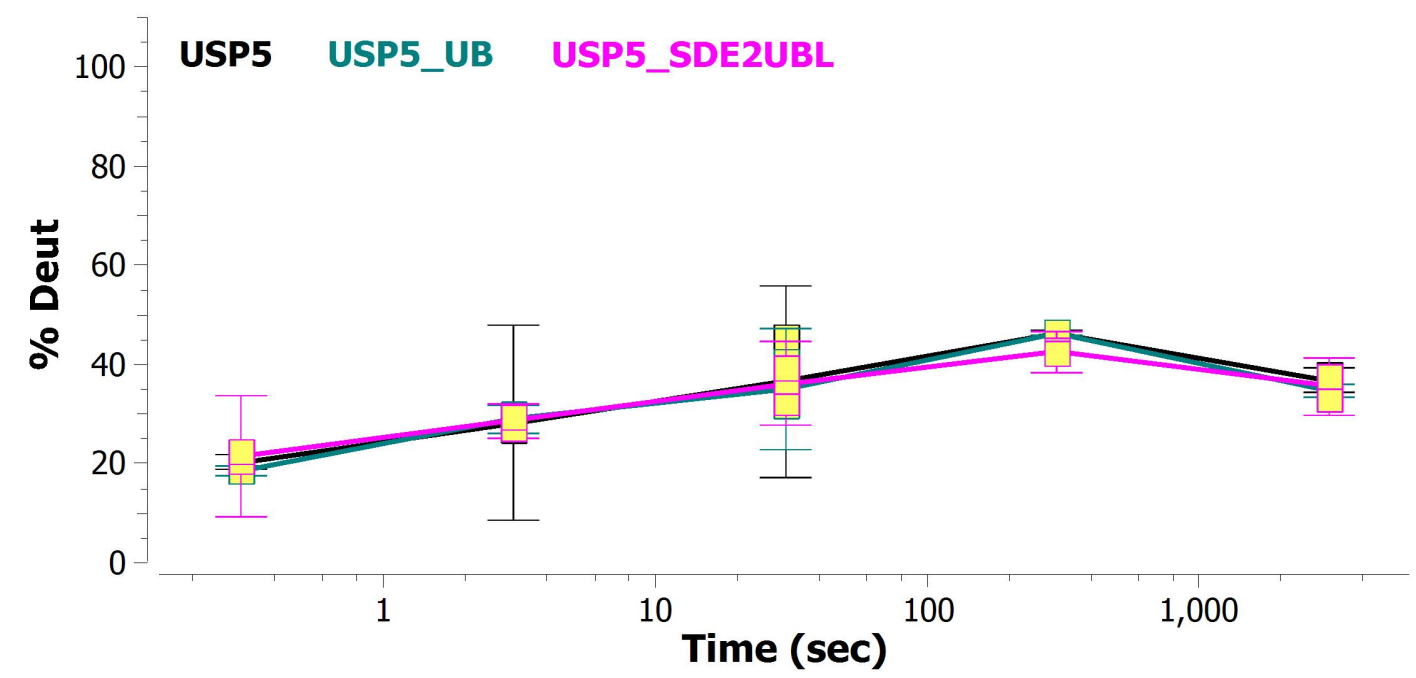

P45974 805-815: AFISHMGTSTM (#173)

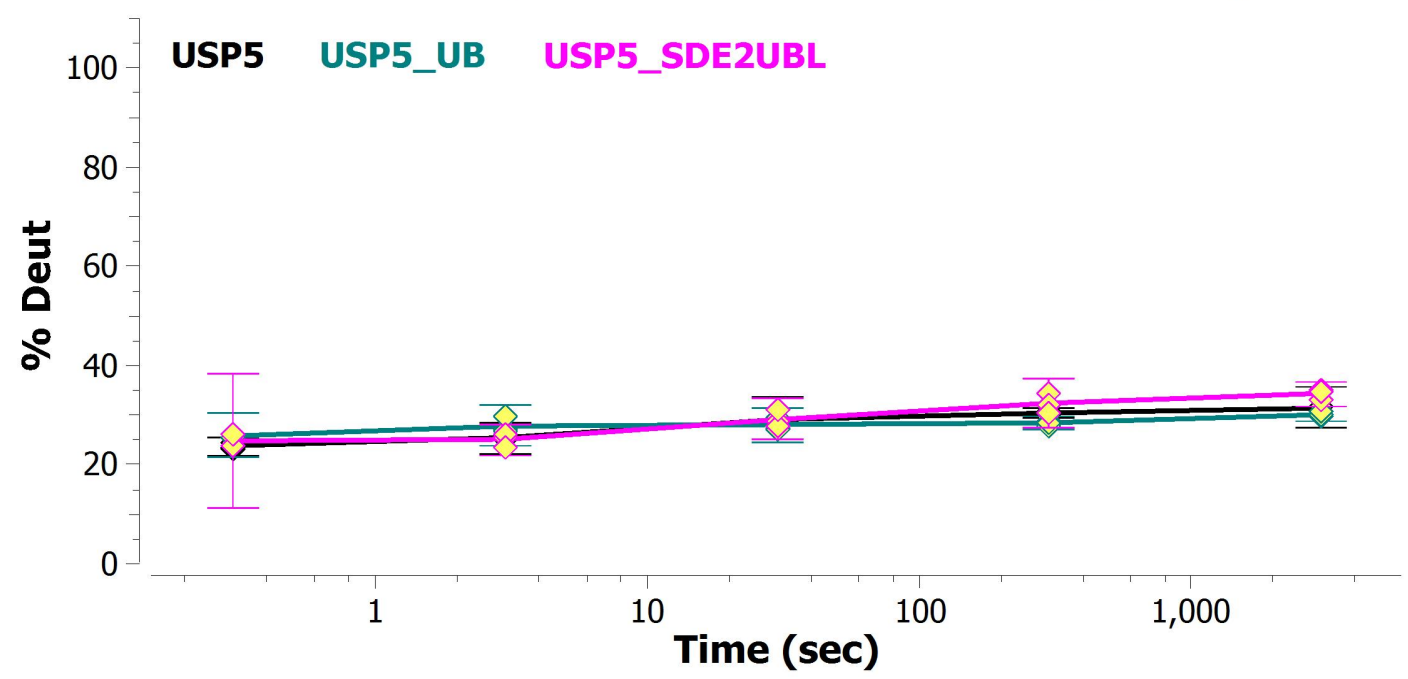

P45974 806-815: FISHMGTSTM (#174)

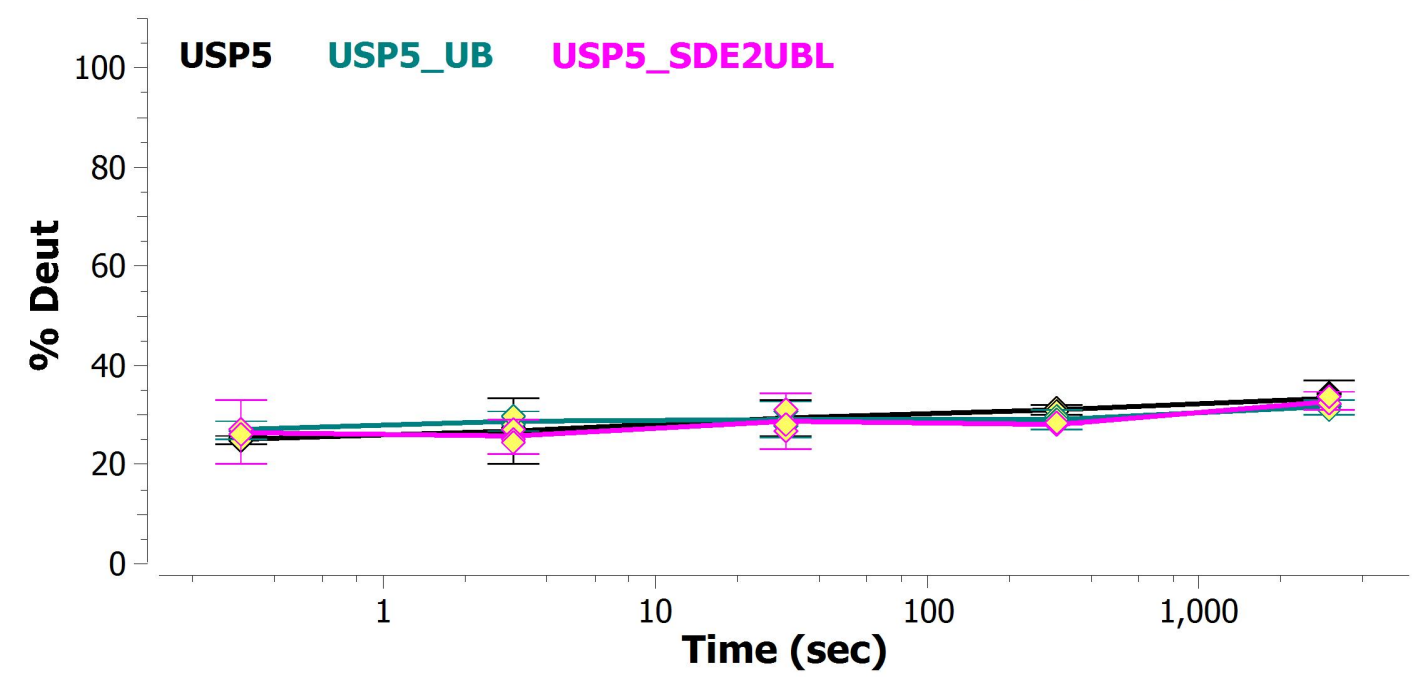

P45974 806-817: FISHMGTSTMCG (#175)

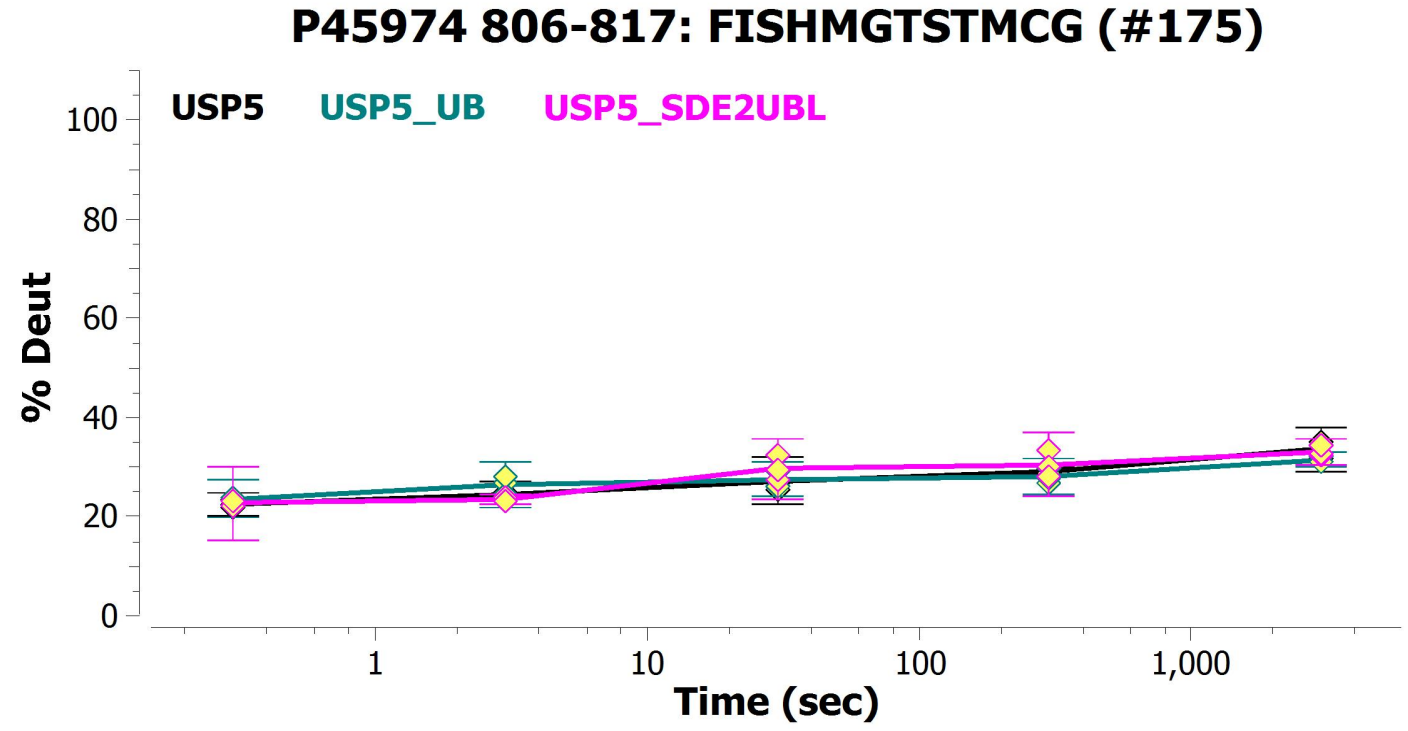

P45974 806-829: FISHMGTSTMCGHYVCHIKKEGRW (#176)

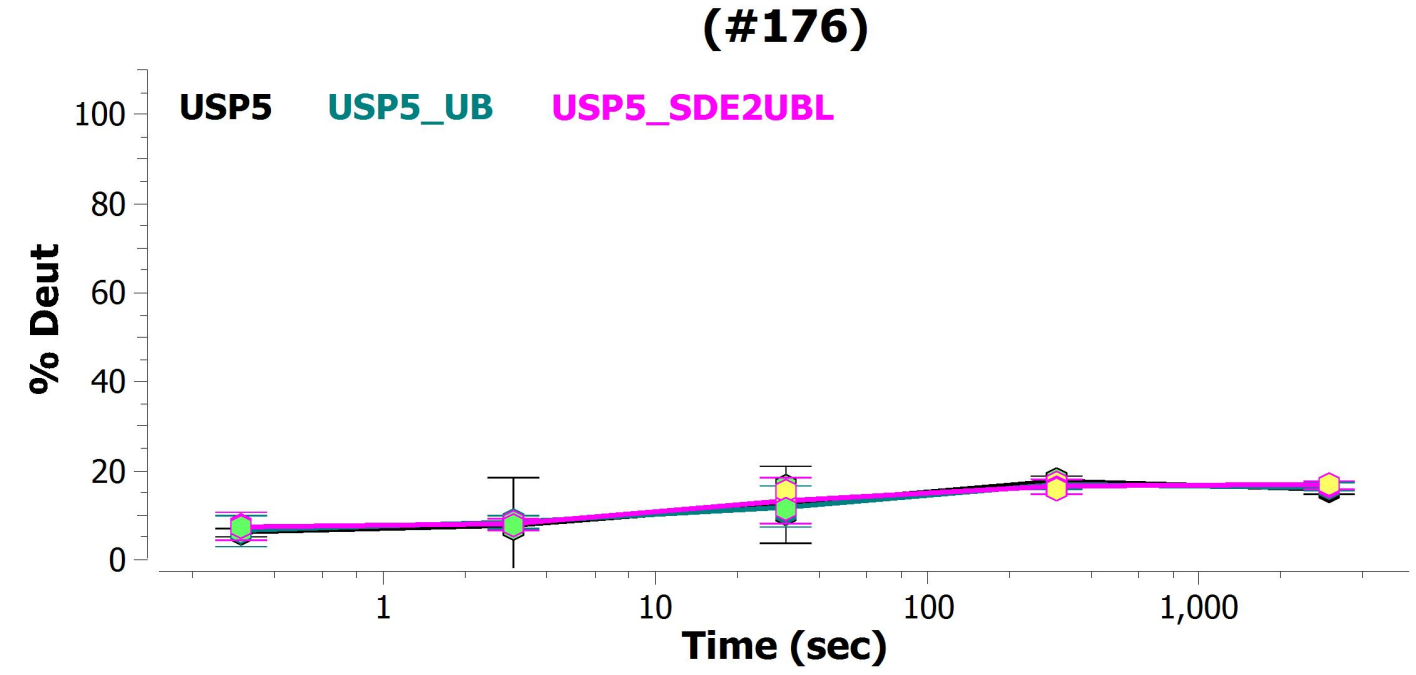

P45974 806-839: FISHMGTSTMCGHYVCHIKKEGRWVIYNDQKVCA (#177)

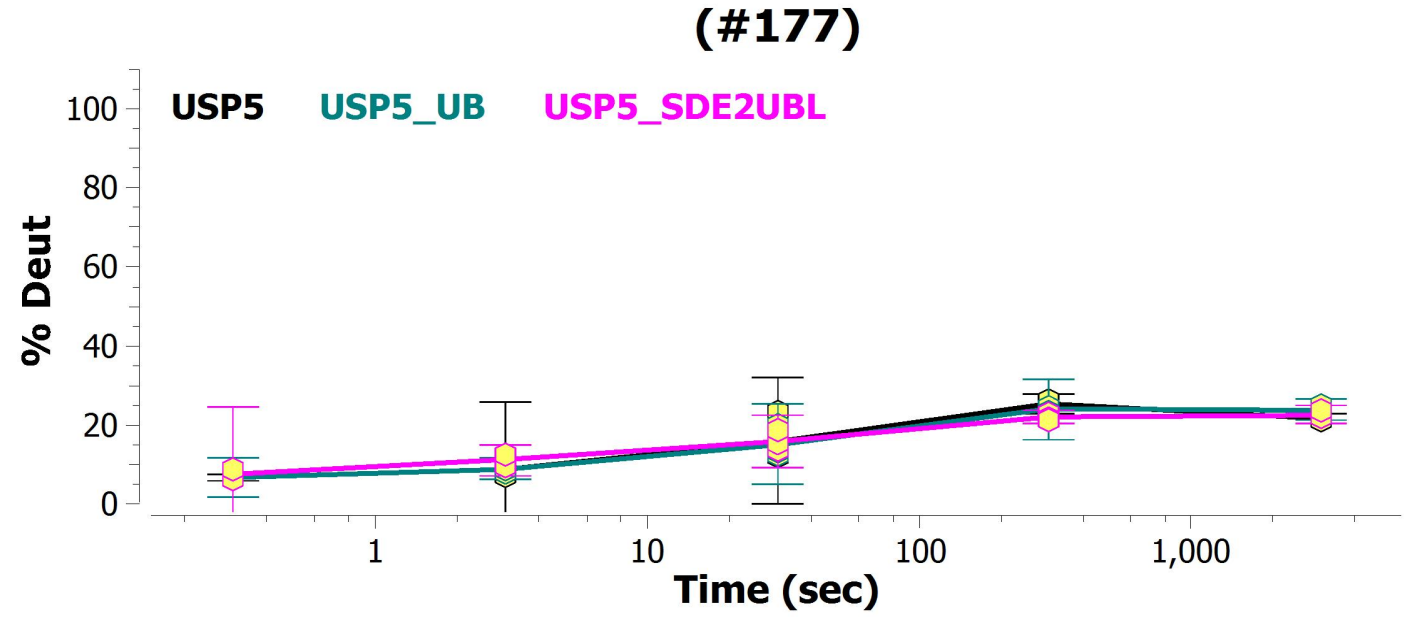

P45974 807-815: ISHMGTSTM (#178)

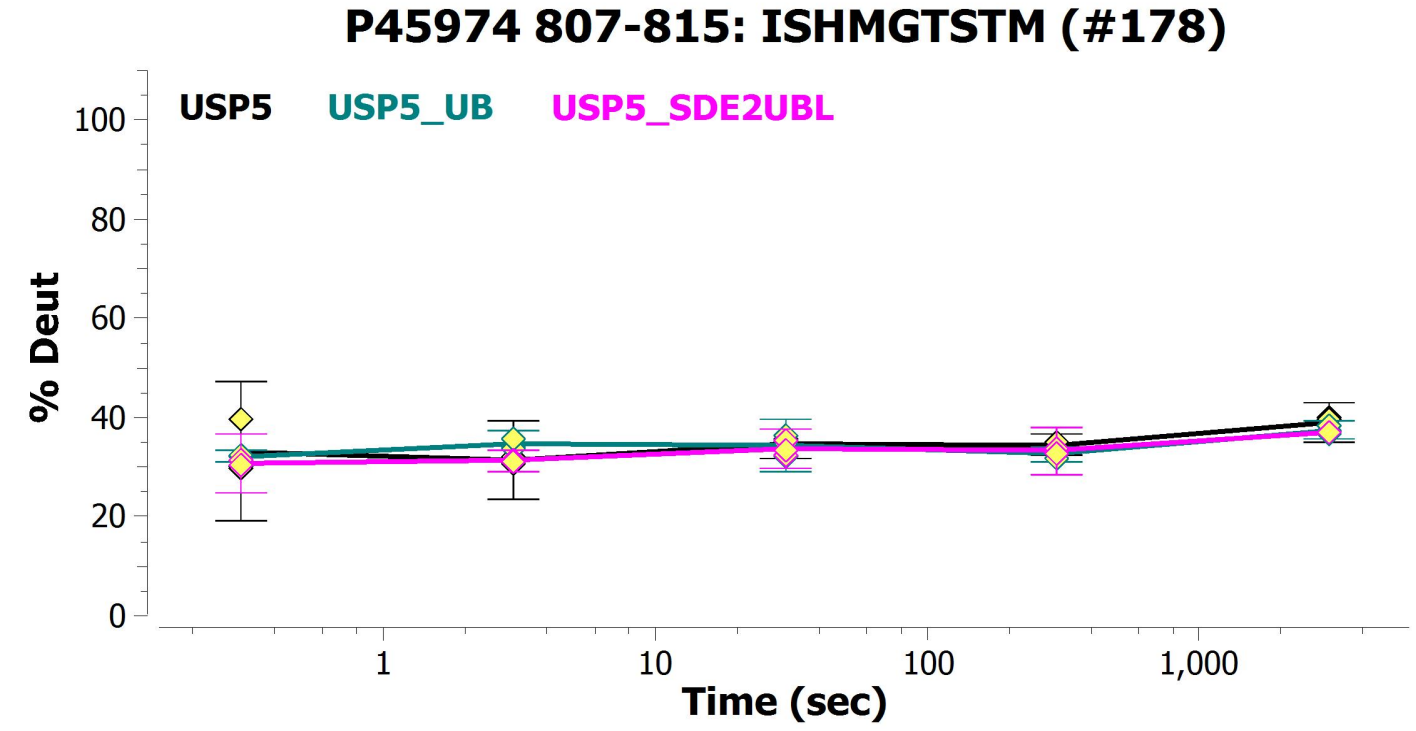

P45974 807-817: ISHMGTSTMCG (#179)

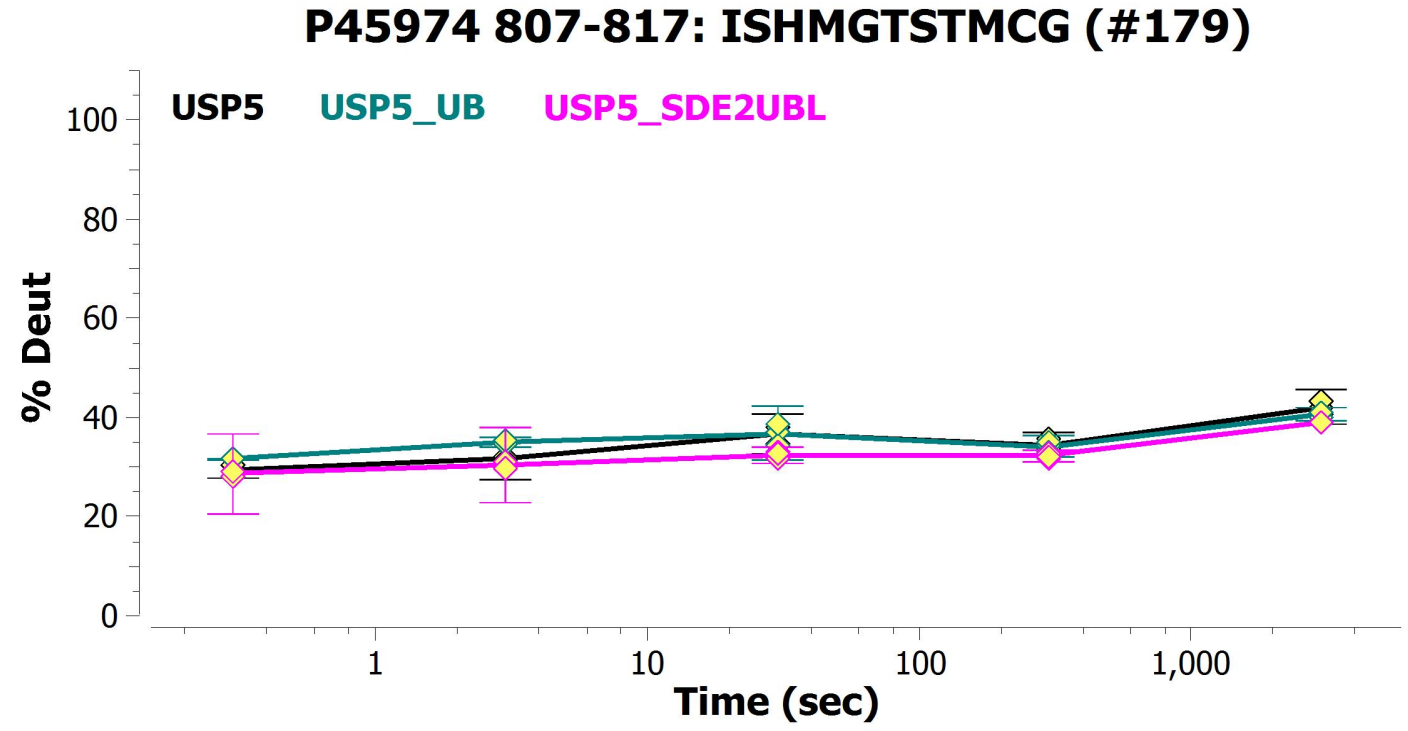

P45974 807-822: ISHMGTSTMCGHYVCH (#180)

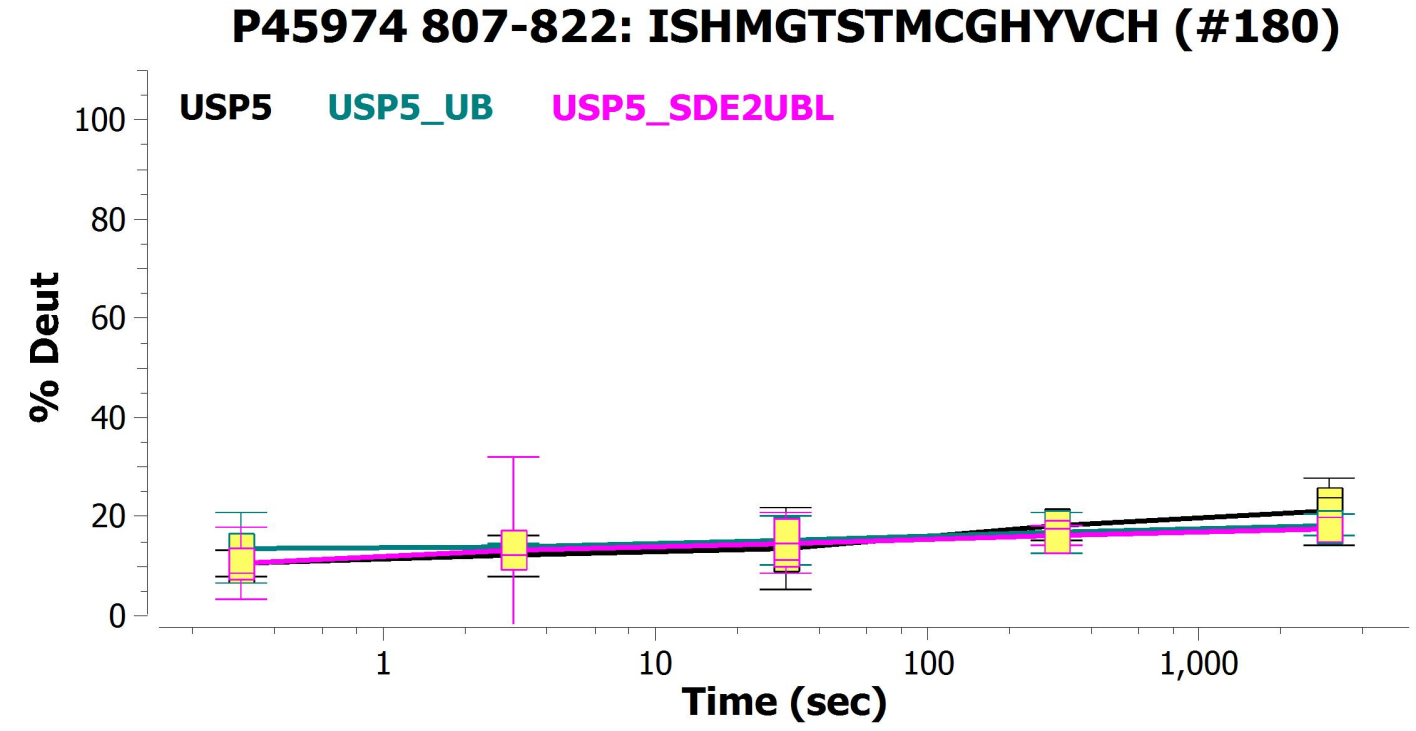

P45974 807-829: ISHMGTSTMCGHYVCHIKKEGRW (#181)

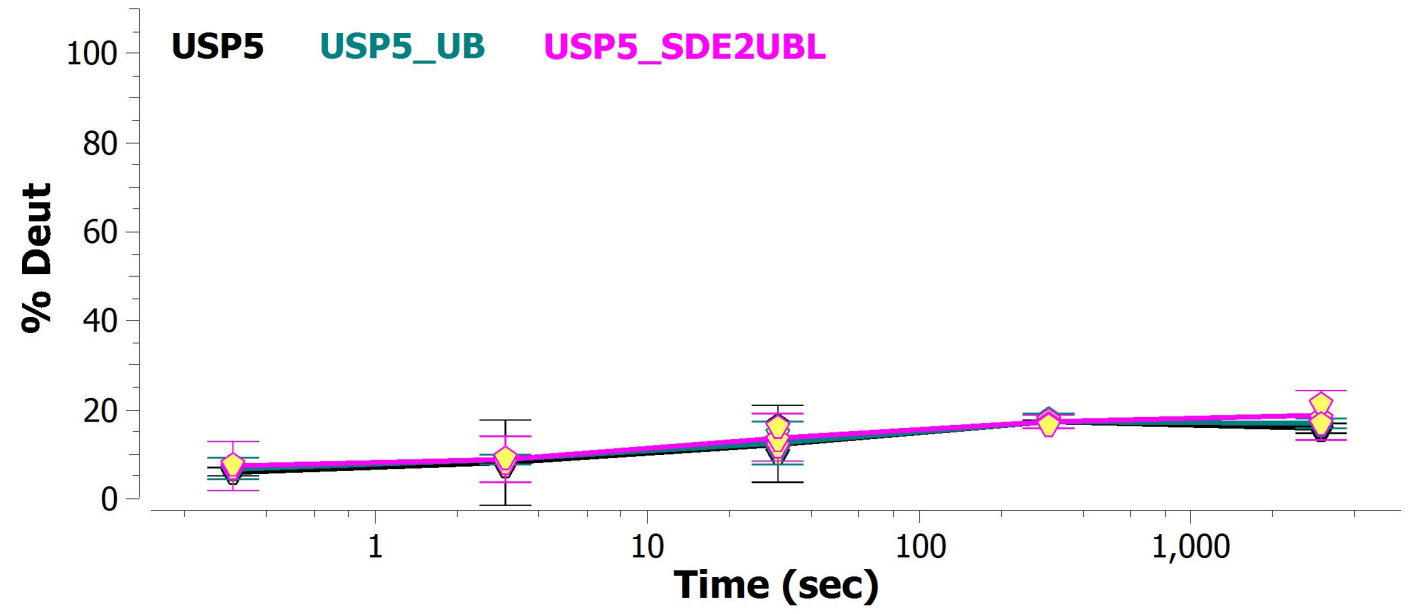

P45974 807-848: SHMGTSTMCGHYVCHIKKEGRWVIYNDQKVCASEKPPKDL (#182)

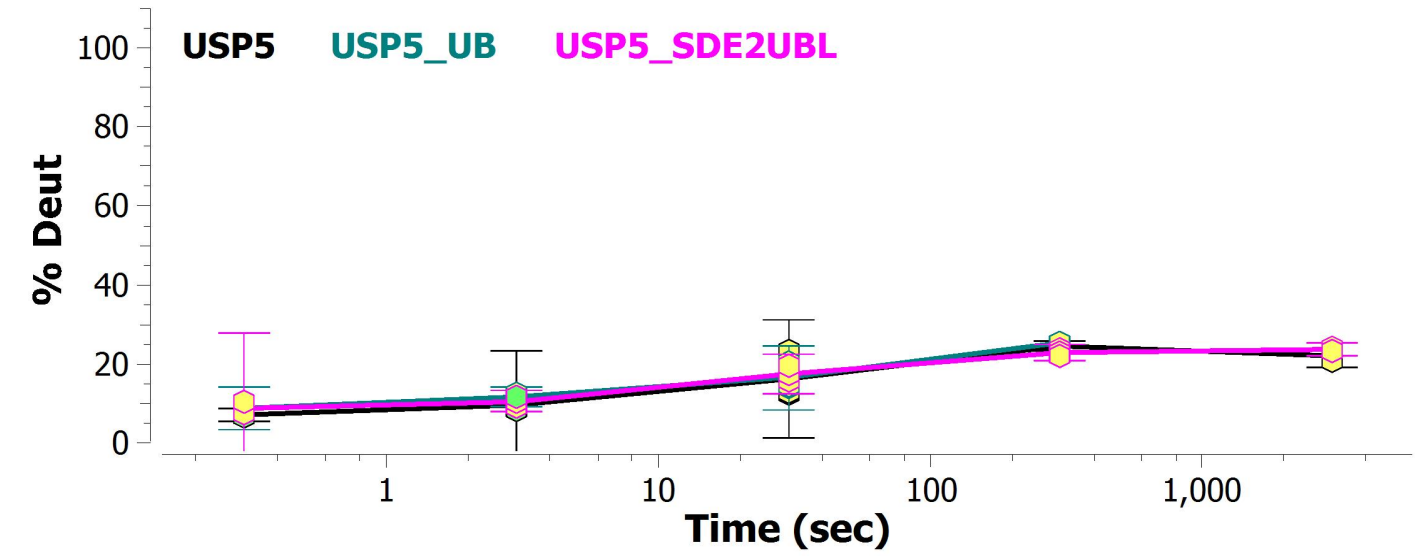

P45974 816-829: CGHYVCHIKKEGRW (#183)

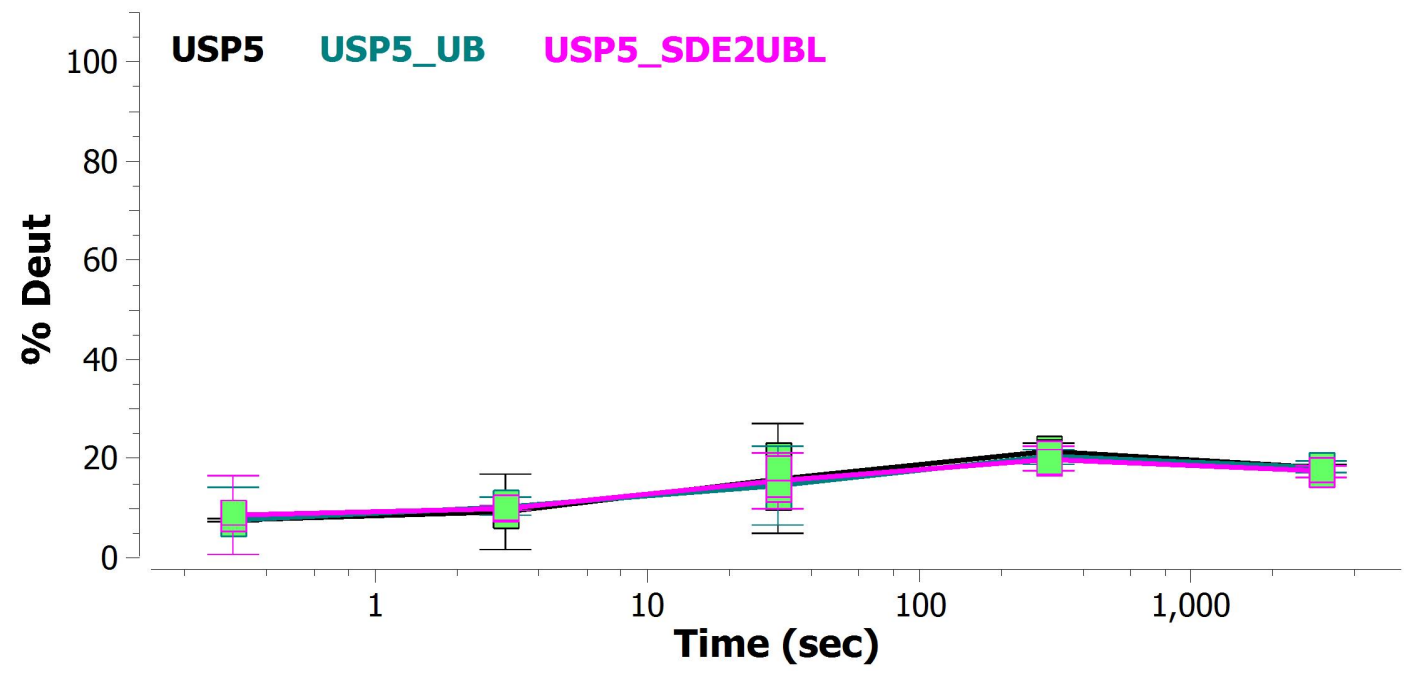

P45974 816-839: CGHYVCHIKKEGRWVIYNDQKVCA (#184)

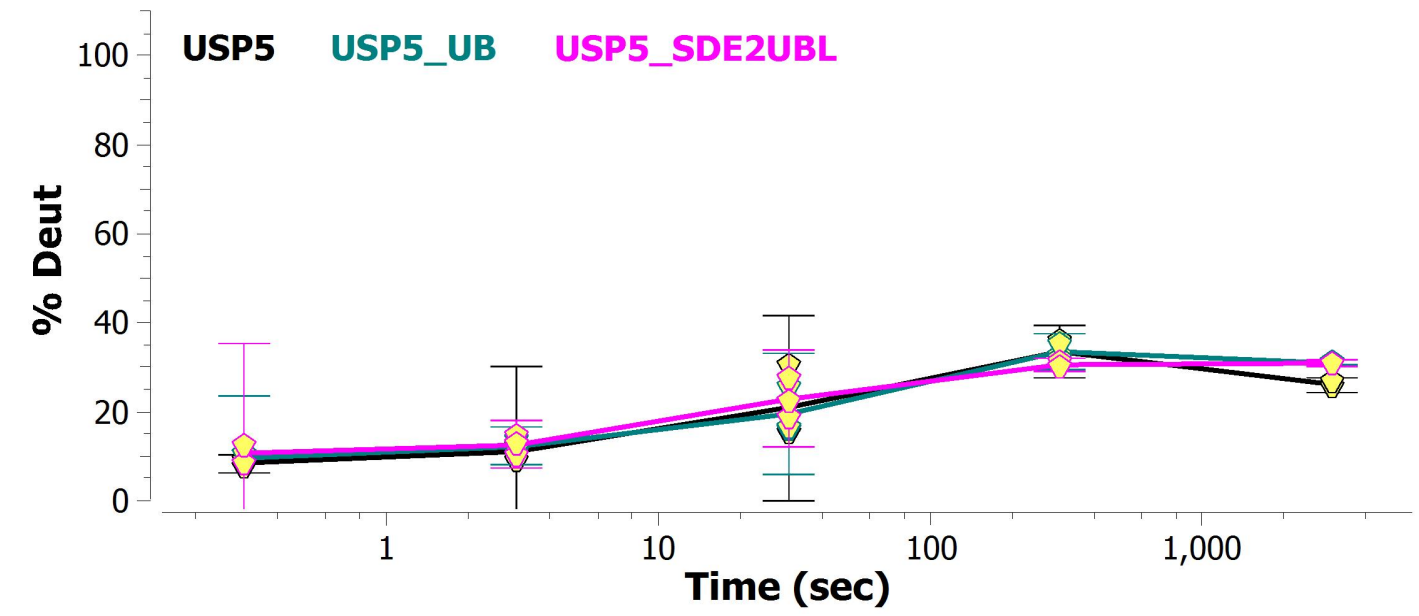

P45974 818-829: HYVCHIKKEGRW (#185)

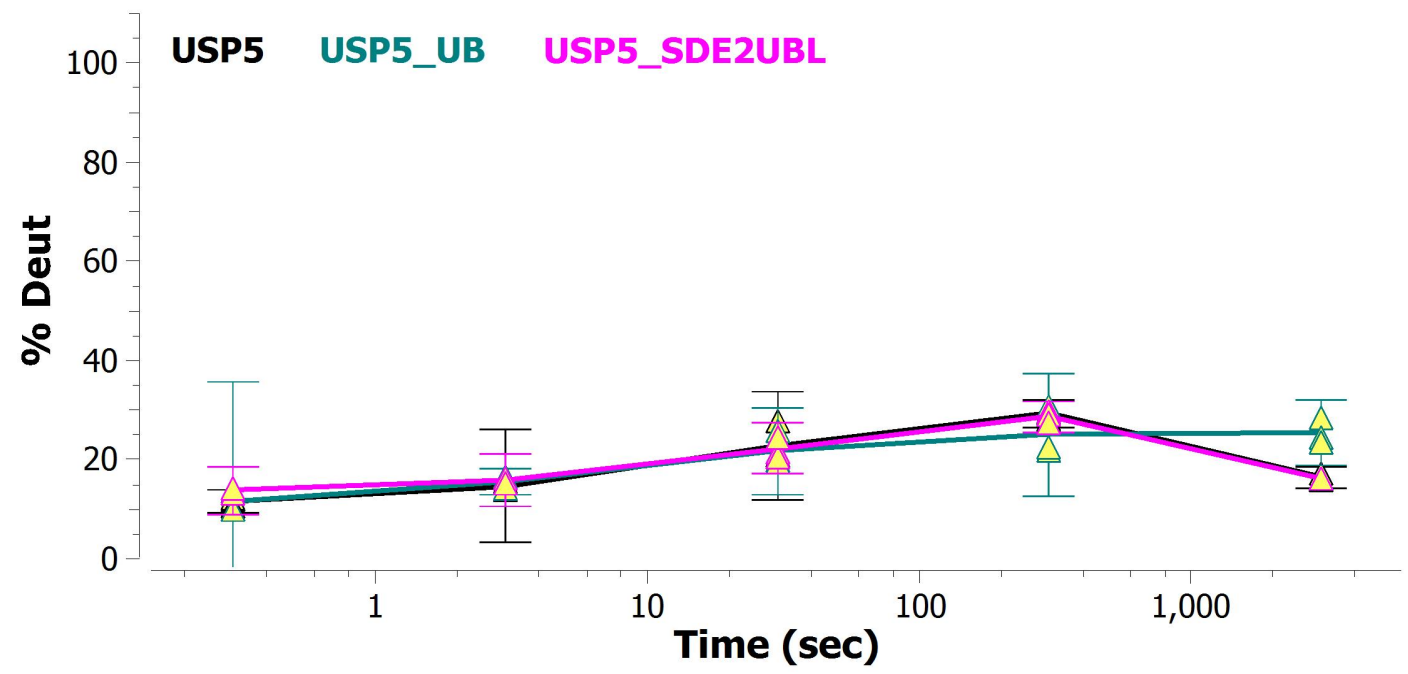

P45974 824-831: KKEGRWVI (#186)

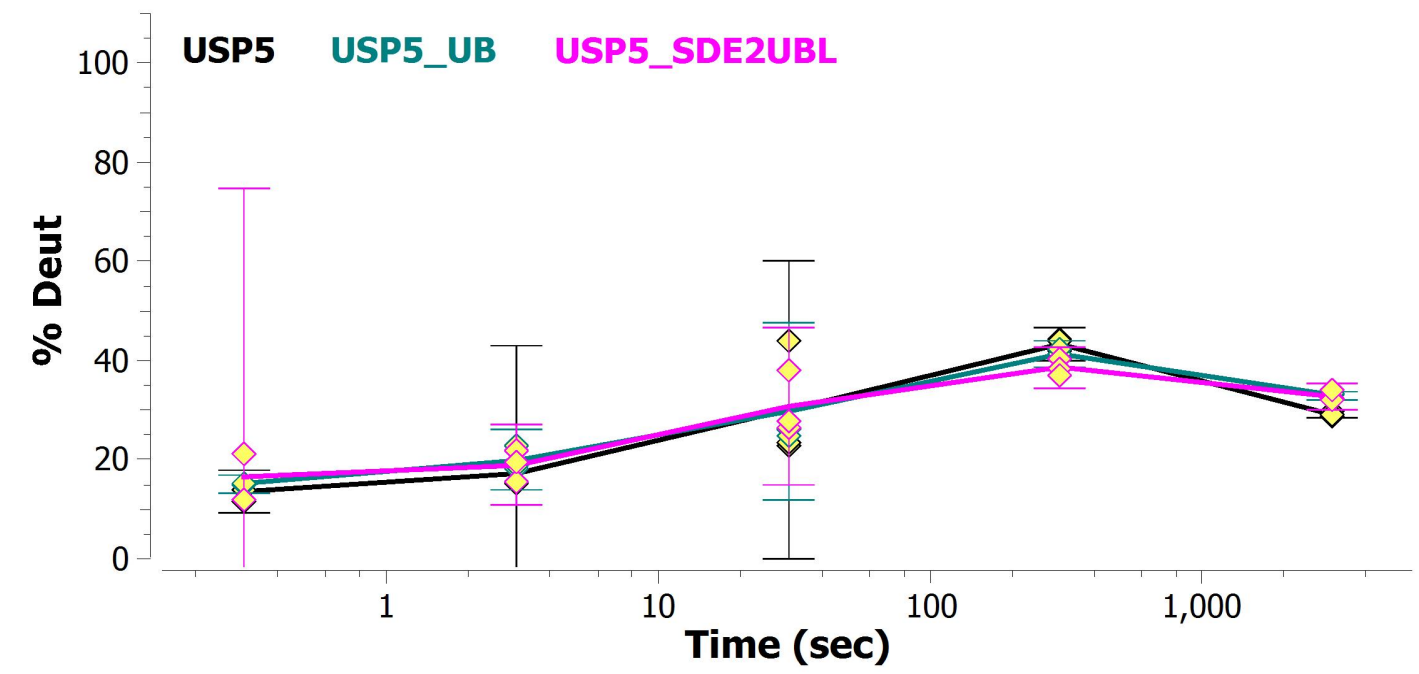

P45974 830-849: VIYNDQKVCASEKPPKDLGY (#187)

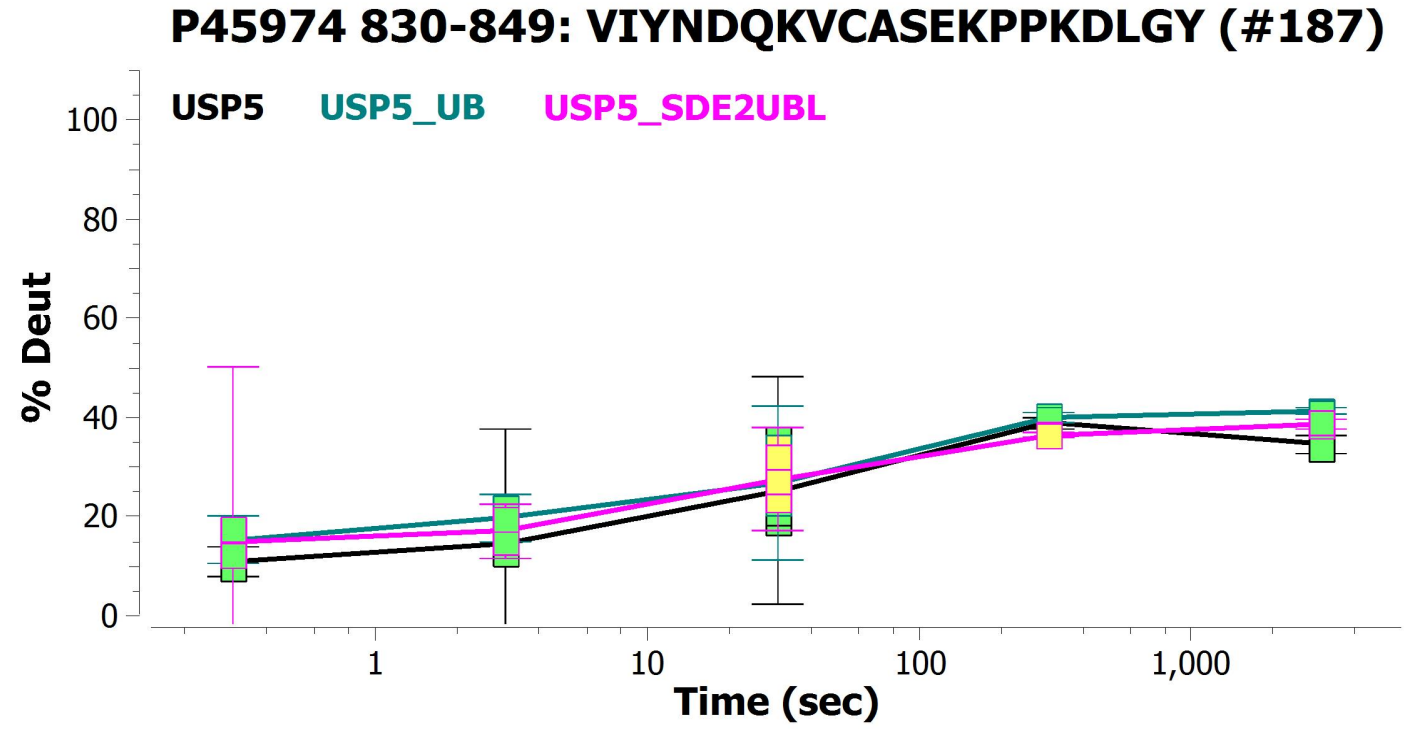

P45974 830-852: VIYNDQKVCASEKPPKDLGYIYF (#188)

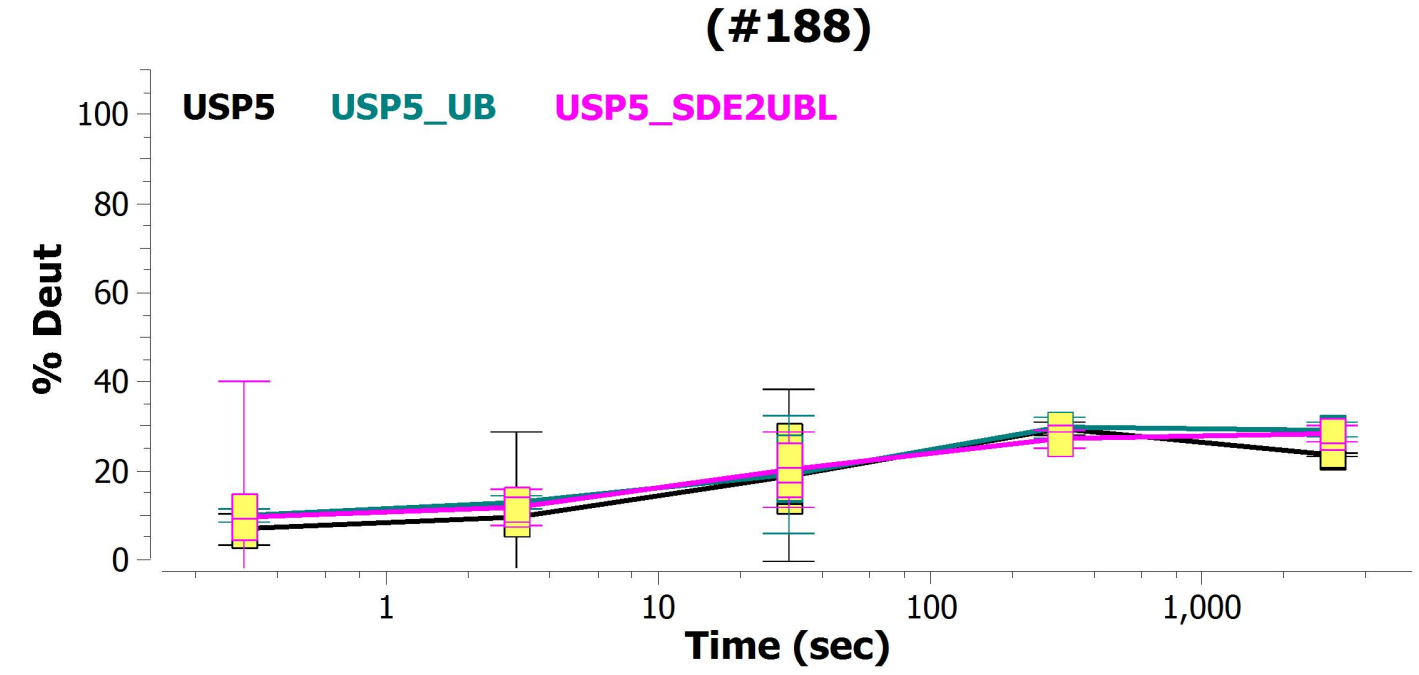

P45974 836-849: KVCASEKPPKDLGY (#189)

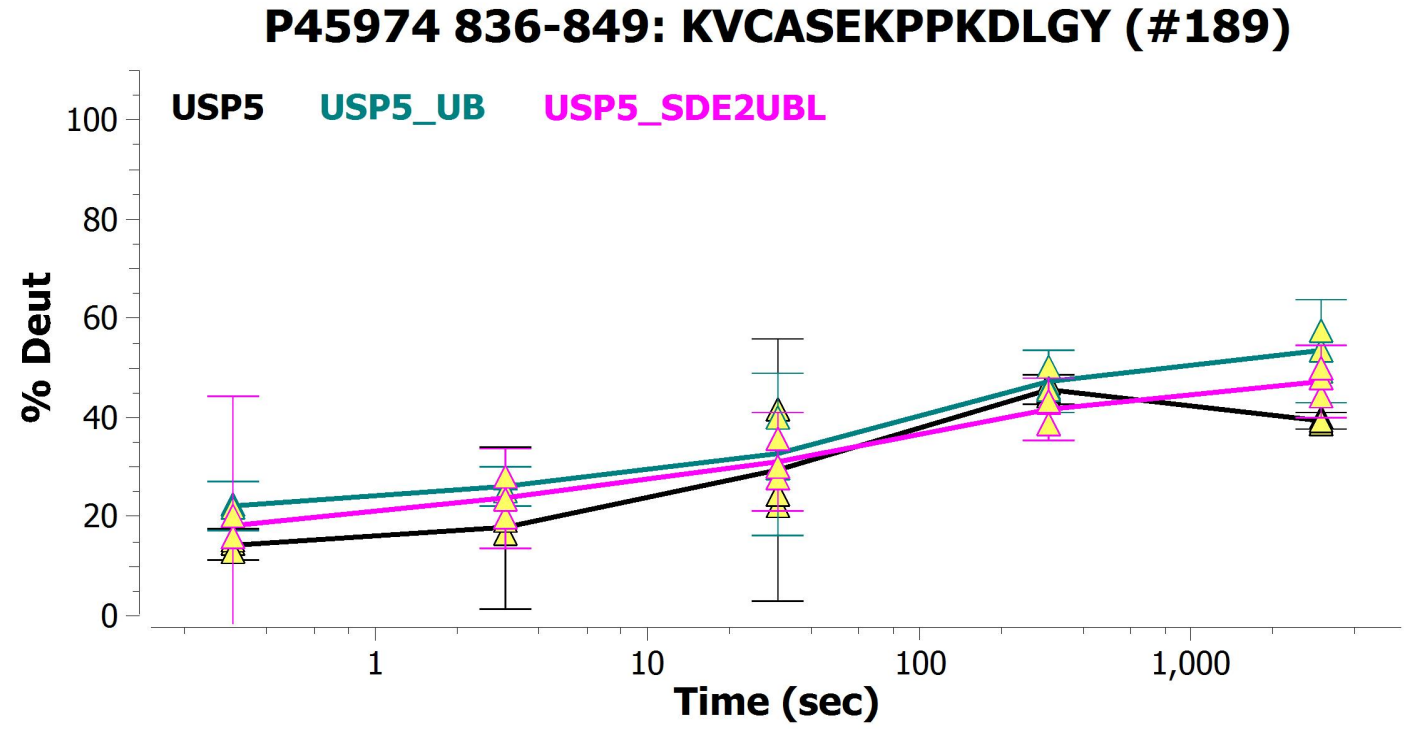

P45974 840-852: SEKPPKDLGYIYF (#190)

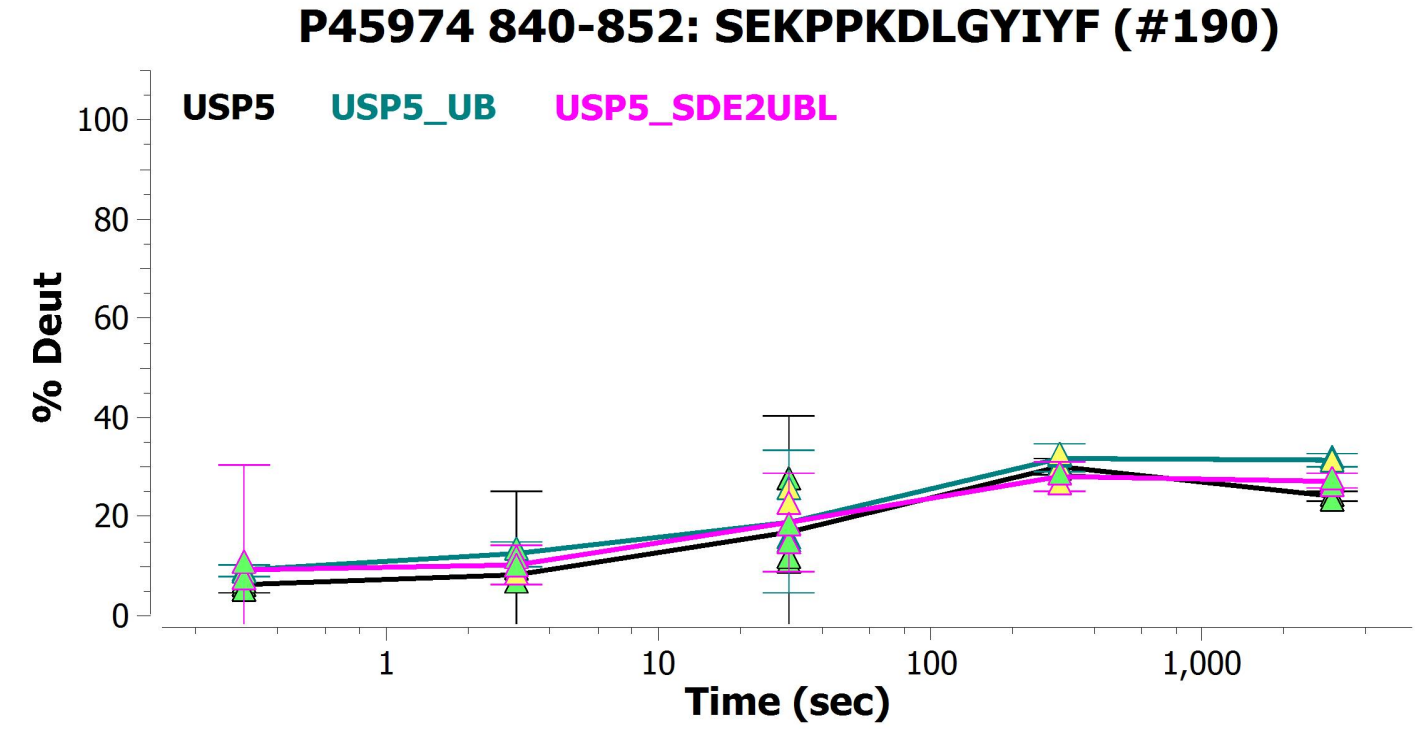

Supplementary Figure 31 | HDX-MS uptake plot tables. Tables reporting peptide-level deuterium incorporation. These data were used to generate the uptake plots shown in the Supplementary Figure 17 and 18.

References

1 Sievers, F. *et al.* Fast, scalable generation of high-quality protein multiple sequence alignments using Clustal Omega. *Mol. Syst. Biol.* **7**, 539 (2011). https://doi.org/https://doi.org/10.1038/msb.2011.75

2 Waterhouse, A. M., Procter, J. B., Martin, D. M. A., Clamp, M. & Barton, G. J. Jalview Version 2—a multiple sequence alignment editor and analysis workbench. *Bioinformatics* **25**, 1189-1191 (2009). https://doi.org/10.1093/bioinformatics/btp033

3 Abramson, J. *et al.* Accurate structure prediction of biomolecular interactions with AlphaFold 3. *Nature* **630**, 493-500 (2024). https://doi.org/10.1038/s41586-024-07487-w
