# Supplementary material for: USP5 acts as a proteolytic switch to control SDE2 function via UBL-directed cleavage
Source: Nat Commun. 2026 Aug 14;17:9775. doi: 10.1038/s41467-026-76751-6 (PMC13572447; doi:10.1038/s41467-026-76751-6)
Supplement: Supplementary file 3 [file 41467_2026_76751_MOESM3_ESM.pdf]

nature portfolio

Peer Review File

USP5 acts as a proteolytic switch to control SDE2 function via UBL-directed cleavage

Corresponding Author: Dr Chiara Maniaci

This file contains all reviewer reports in order by version, followed by all author rebuttals in order by version.

Version 0:

Reviewer comments:

Reviewer #1

(Remarks to the Author)
Title: A Proteolytic Switch: USP5 controls SDE2 function via UBL-directed cleavage

Summary:
This manuscript describes the identification of USP5 as the protease responsible for cleaving the N-terminal ubiquitin-like domain (UBL) of SDE2, a protein involved in genome regulation, DNA repair, and RNA processing. The authors use a combination of biochemical fractionation, proteomics, activity-based probe design, mutagenesis, and binding assays to characterise this previously unreported processing event.

The study offers a technically thorough exploration of the USP5–SDE2 relationship. However, while the biochemical evidence is robust, the manuscript would benefit from additional clarification of methodology, reduction of redundancy across experiments, and—critically—greater biological and structural validation to support the physiological relevance and mechanistic specificity of the findings.

I believe the study has merit, but in its current form, it falls short of fully justifying its conceptual impact. The manuscript would be strengthened considerably by addressing the points below.

Major Comments:
1. Functional and Phenotypic Validation is Lacking

While the authors show that USP5 cleaves SDE2 at the UBL domain, the biological consequence of this cleavage remains undefined. Given that SDE2 is implicated in splicing, DNA repair, and replication stress, it would be valuable to explore:

- Whether USP5 knockdown/inhibition with small molecules phenocopies cleavage-deficient SDE2.
- Whether cleavage impacts known SDE2 functions or associated signalling pathways.
- Whether this event is dynamically regulated during stress conditions.

The inclusion of functional/phenotypic assays would substantially improve the manuscript's impact and biological relevance.

2. Use of a Single Cell Line Limits Generalisability

The majority of experiments are conducted in HEK293 or HEK293T cells. U2OS cells are used only in Figure 4C, with no rationale provided. Given the potential for cell-type-specific regulation of DUBs, it would be important to:

- Justify the use of HEK293T as the primary model.
- Acknowledge the limitation of relying on one cell line.
- Ideally, confirm cleavage of SDE2 by USP5 in a number of unrelated cell lines.

Including broader cellular validation would strengthen the argument that USP5 is the main effector of SDE2 processing.

3. Experimental Redundancy and Data Consolidation

The authors employ a wide array of orthogonal assays (SDS-PAGE, MALDI-TOF, ABP labelling, TR-FRET, and ITC) to demonstrate USP5 cleavage of SDE2. While the methodological rigour is appreciated, the repetition of similar results in multiple formats creates unnecessary redundancy.

The authors might consider condensing overlapping experiments, potentially moving some data to supplementary figures, to focus the main narrative.

4. Structural Insight is Underdeveloped

The central mechanistic claim is that SDE2-UBL mimics ubiquitin to engage USP5. However, no structural models or experimental structures are presented. AlphaFold predictions are referenced but are insufficient to demonstrate binding specificity.

Suggestions for improvement include:

- Docking models comparing USP5 bound to SDE2-UBL (WT and with mutations as in Fig. 3A), full-length SDE2, and ubiquitin.
- Consideration of experimental approaches such as x-ray crystallography, HDX-MS, cryo-EM or cross-linking mass spectrometry.

These additions would clarify the molecular basis for substrate selectivity.

Specific Comments and Clarifications:

- Line 39: The phrase “proteomic profiling and mass spectrometry” appears redundant—consider streamlining.
- Lines 40 and 115: The use of “in cell” and “in cellulo” is inconsistent. Standardise terminology throughout.
- Figure 2D: Only 24 proteins were identified across 9 fractions, which seems unexpectedly low. Can you clarify? How many total fractions were collected, and what were the criteria for inclusion?
- Figure 2G: USP13 (aa 318–363) shows cleavage activity, yet this is not recapitulated in subsequent experiments. The authors should reconcile this discrepancy.
- Line 232: Activity-based protein profiling (ABPP) measures reactivity, not interaction per se. The wording should be revised accordingly.
- Figure 3A: SDE2-UBL is not shown in the gel—why? Is it degraded? SDE2 is meant to be degraded upon cleavage. Should we see this effect in this figure? Please clarify. Additionally, combining this data with structural modelling of SDE2 WT, mutants, and ubiquitin with USP5 would strengthen the analysis.
- Line 497 vs. 506: The text appears to both affirm and deny USP5’s ability to cleave ubquitin from modified proteins. Please clarify.
- ISG15-PA in Figure 5B: Since this probe is based on a truncated C-terminal domain, the reactivity with USP5 may be underestimated. This caveat should be acknowledged.
- ABP labelling: Probe titration experiments would be valuable to define the optimal ABP concentration for specific labelling.
- C335A mutant: This mutant increases binding to Ubiquitin affinity in DUBs and does not, in isolation, demonstrate C335-dependence. Complementary mutants such as C335R or C335S should be considered.
- Figure 4C: U2OS cells are used only here, but not described in the Methods. Please include their source and culture conditions.

Conclusion:
This study addresses a relevant and timely question in post-translational regulation, and the biochemical characterisation of USP5 as a protease for SDE2 is executed with rigour. However, in its current form, the manuscript does not yet provide sufficient mechanistic insight or biological validation to support the claims of novelty and significance.

Reviewer #2

(Remarks to the Author)
Hales et al. present an intriguing manuscript exploring how the spliceosome factor and DNA damage response regulator, Silencing Defective 2 (SDE2), undergoes post-translational cleavage by the deubiquitinating enzyme USP5. SDE2 is conserved from yeast to humans, and while the enzymes responsible for this cleavage in yeast (Ubp5, Ubp15) are known, those in humans remain unidentified. By employing target-driven methods with recombinant proteases and unbiased screening, the authors discover that the deubiquitinating enzyme USP5 (not an orthologue of Ubp5/15) cleaves SDE2 by recognising its N-terminal UBL domain.

Overall, the paper is well written and the experiments are conducted to high standards, complemented by biochemical tests that support both in vitro and in cellulo findings. Occasionally, it feels somewhat exhaustive, and it's unclear why some data- such as alanine scanning mutagenesis- are included beyond the fact that the experiments were performed. My main feedback for the authors focuses on clarifying the mechanisms by which USP5 recognises SDE2.

Introduction: Provide additional details explaining why SDE2 must be processed for incorporation into spliceosomes. For example, is steric occlusion from the N-terminal UBL preventing its incorporation? A general description of why SDE2

requires proteolytic processing to become functional would help better set the scene for identifying enzymes that fulfil this role.

The assessment of Ubp5/15 orthologues was thorough, and it was nice to see the conventional biochemical method of cellular fractionation used to identify human proteases involved in SDE2 cleavage. However, the narrative became somewhat disrupted when the authors introduced a focused MALDI-TOF screen. They should clarify the rationale for conducting two screens, especially since the key outcome was already obtained. Additionally, more details are needed about the MALDI-TOF screen, such as why some control substrates do not react with the tested DUBs. Specifically, it should be explained how the authors conclude that USP22, USP26, and others are functional if no activity is observed with the control substrate.

Furthermore, the authors observe activity from USP13 towards SDE2 in the MALDI-TOF screen, with this activity being more pronounced towards SDE2 than the control substrate. How do the authors reconcile this activity when the subsequent set of experiments shows no activity from USP13?

The reason for concentrating on residues from Ser69-Leu88 of SDE2 for USP5 cleavage was unclear, both from Figure 1 c,d and the main text, and should be clarified.

The authors created a HA-SDE2-UBL-PA probe and tested its reactivity with USP5 and USP13. USP5 reacted, but USP13 did not, even from HEK293 extracts. However, this does not rule out USP13's ability to cleave SDE2, since it recognises full-length SDE2 but may not bind strongly enough to the UBL domain. Notably, recombinant USP13 does not react with the SDE2-UBL probe, likely because a shorter USP13 fragment was used (see size differences in Figure 3d), missing the N-terminal ZnF domain essential for USP5 activity. Therefore, the authors should repeat this assay using full-length USP13. The in cellulo depletion using siRNAs against USP5 or USP13 shown in Figure 4c appears to show a stronger dependence on USP13 for cleavage of SDE2 than USP5. Though any comparable cleavage of endogenous SDE2 is difficult to see, why do the authors use two Western blots for full-length and cleaved SDE2? Why does there appear to be no effect by USP5? Is this experiment performed under steady-state conditions?

The authors used ITC to measure the interaction between USP5 and SDE2. Why does SDE2 UBL bind USP5 with 1µM affinity, but full-length SDE2 cannot? The authors show that the USP5 ZnF drives the interaction between SDE2 UBL and USP5, but there is no contribution of the ZnF in cleaving full-length SDE2. Is it possible that the ZnF only binds to processed SDE2, similar to how it recognises the C-terminus of ubiquitin? The authors could test this by showing whether SDE2 UBL requires the C-terminal Glygly for binding to USP5. Additionally, the authors should examine whether USP5 ZnF binds with a similar affinity to SDE2 UBL compared to full-length USP5.

The authors should also consider that the binding of SDE2 UBL to USP5 ZnF might be causing product inhibition, which could explain why only 50% of SDE2 is processed in assays with recombinantly purified proteins, but not in cell extract experiments. Have they thought about conducting competition assays—such as titrating SDE2 UBL while observing the cleavage of full-length SDE2? Additionally, what role does ubiquitin play in SDE2 cleavage? Does it influence the kinetics of the process?

Reviewer #3

(Remarks to the Author)
The authors in this paper investigate the DUB enzyme responsible for the cleavage of human SDE2FL into SDE2CT and SDE2UBL. Through a combination of biochemical and cellular assays using distinct readouts, the authors were able to identify USP5 as the protease responsible for the proteolytic event. The experiments broadly support the claims made by the authors and have great value in the field; however, some clarifications are required, experiments should be repeated, and additional experiments can be incorporated to recommend the manuscript for publication in this journal.

Major comments:
• The band for HA-SDE2UBL-PA samples in figure 5A is notoriously faint, except for the sample where USP5 was used. It almost suggests that more SDE2-based probe was used. Can you comment on this?
• It is mentioned that KD of USP5 “consistently impaired SDE2 processing, as indicated by the accumulation of SDE2FL”. While this is greatly noted in the NanoBRET system (figure 4C), it is hard to appreciate for the endogenous protein (figure 4D). While there is a noticeable difference between USP13 siRNA and USP5 siRNA samples, it is negligible when comparing USP5 samples to NT or control siRNA. Can you repeat the experiment and incorporate a sample where USP5 and USP13 are overexpressed? Overexpression of the DUBs could serve as a positive control to demonstrate that enhanced cleavage mimics the SDE2FL siRNA control.
• An overview of the biological functions of SDE2 was described, but how USP5 perturbation changes its cellular functions is not explored. I believe the manuscript can greatly benefit from tying USP5’s activity to SDE2 function, which can be performed via siRNA as tested in some experiments, or assessing the potential of incorporating commercially available USP5 inhibitors. (https://pubs.acs.org/doi/10.1021/acs.jmedchem.1c00889)

Minor comments:
• It is mentioned that there’s the possibility that a cofactor is present in cell lysate or a PTM is at play and absent in biochemical environment/recombinant protein; it has been reported before that mammalian expression systems, in some instance,s can retain PTMs. Is this something that can be explored further? Is there a possibility that a signaling event on

SDE2 is triggering the binding/proteolytic event in cells?
Furthermore, in the pool of proteins identified from proteomics in figure 2D, is there any protein that has been reported to interact with USP5/can serve as a scaffolding protein or aid in the cleavage of SDE2 by USP5? Highlighting such candidates could help explain why cleavage is more efficient in lysates than in vitro.
• In addition to the previous comment in figure 5A, it seems that USP7 has ISG15 activity, as there's a faint band above USP7 in lane 4 of the USP7 samples; however, it's hard to tell since the band for USP7 is too broad and it masks this finding. Is this something previously observed for USP7? Can the experiment be repeated using different PAGE conditions to promote better separation of high molecular weight bands?
• Mislabeling:
o In line 264, figure 3C is referenced; however, it highlights the experiment in figure 3D? Can you confirm?
o Figures 6F,G are mislabeled as 6H,I in text and figure caption
• Figure 5C can be placed in the supplemental section. It feels secondary and not critical to the main argument of the figure.

Version 1:

Reviewer comments:

Reviewer #1

(Remarks to the Author)
The revised manuscript by Hales et al. presents a technically rigorous and comprehensive study identifying USP5 as the protease responsible for SDE2 cleavage. The biochemical and mechanistic aspects of the work are strong, and the use of multiple orthogonal approaches is a clear strength.

The authors have addressed several of the previous concerns, most notably by incorporating additional cellular data linking USP5 depletion to intron retention in SDE2-dependent transcripts. This represents a meaningful improvement over the original submission.

However, despite these revisions, I remain concerned that the manuscript still falls short of fully establishing the biological relevance and mechanistic robustness expected for publication in Nature Communications. In particular, key controls are missing in critical experiments, the functional validation remains limited in scope, and some of the structural interpretations appear overstated given the available data.

For these reasons, I believe that further revision is required.

Major comments

1. Missing controls in the intron retention experiments (Figure 6)
The intron retention analysis is now central to the manuscript's biological claims. However, the figure does not clearly show knockdown efficiency for USP5 and/or SDE2 within the same experimental context. Without these controls, it is difficult to directly attribute the observed phenotype to the intended perturbation. These controls are essential and should be included alongside the RT-PCR data.

2. Limited functional validation of biological relevance
While the addition of intron retention data is appreciated, the manuscript still relies largely on a single molecular readout (without accompanying controls) to support the biological significance of USP5-mediated SDE2 cleavage. Given the broader roles of SDE2 in splicing, DNA repair, and replication stress, this represents a relatively narrow validation of biological impact. At minimum, the authors should better contextualise this limitation; ideally, an additional proof-of-concept experiment demonstrating functional consequences at the cellular level (e.g. under stress conditions) would substantially strengthen the manuscript.

3. Clarity and interpretation of gel-based data
Figures presenting intron retention and related assays show bands without clear annotation of what is being measured (e.g. RT-PCR products vs immunoblotting). While this may be inferred by a specialist reader, clearer labelling within figure panels and legends is necessary to ensure readability and transparency.

4. Structural interpretation remains somewhat overstated
The inclusion of HDX-MS data is a valuable addition; however, the conclusions drawn from these experiments should be moderated. The coverage of USP5 is incomplete (85% is considered low for HDX-MS), including regions proximal to the catalytic site, which limits the strength of the mechanistic claims regarding binding mode and substrate mimicry. These limitations should be more explicitly acknowledged, and the interpretation tempered accordingly.

Reviewer #2

(Remarks to the Author)
The authors should be commended for their thorough effort in addressing all the reviewer comments and for addressing my

concerns. In particular, the inclusion of functional assays demonstrating splicing impairment in several candidate proteins further strengthened the paper and underscored the importance of USP5-mediated processing of SDE2.

I have one minor comment: for the AlphaFold 3 modelling, please include the corresponding PAE plots in Supplementary Figures 4a, b and in Supplementary Figure 10.

Reviewer #3

(Remarks to the Author)
The manuscript is well-written, and this revised version shows significant improvements in cohesion and scientific rigor. The follow-up experiments and the subsequent refinements to the data effectively support the authors' original claims.

This work is significant to the field, as it opens new avenues for studying DUBs in novel regulatory contexts. Furthermore, the methodology is sound and the procedures are described with sufficient detail to allow for reproducibility. Therefore, I recommend this revised manuscript for publication

Version 2:

Reviewer comments:

Reviewer #1

(Remarks to the Author)
I would like to thank the authors for their efforts in improving the manuscript and for addressing the reviewers’ comments to a great extent. I believe the revised version is substantially strengthened compared to the original submission.

I would therefore recommend the manuscript in its current form for publication.

Open Access This Peer Review File is licensed under a Creative Commons Attribution 4.0 International License, which permits use, sharing, adaptation, distribution and reproduction in any medium or format, as long as you give appropriate credit to the original author(s) and the source, provide a link to the Creative Commons license, and indicate if changes were made.
In cases where reviewers are anonymous, credit should be given to 'Anonymous Referee' and the source.
The images or other third party material in this Peer Review File are included in the article’s Creative Commons license, unless indicated otherwise in a credit line to the material. If material is not included in the article’s Creative Commons license and your intended use is not permitted by statutory regulation or exceeds the permitted use, you will need to obtain permission directly from the copyright holder.
To view a copy of this license, visit https://creativecommons.org/licenses/by/4.0/

A Proteolytic Switch: USP5 controls SDE2 function via UBL-directed cleavage

Reviewer's Comments – Response letter

We would like to thank all Reviewers for their time which was dedicated towards giving thoughtful comments on our work. All raised points are valid and highly relevant. As outlined in the following response, we believe that we have addressed them thoroughly with additional experiments, analyses and changes to the text. As a result, we are convinced that the quality of the index submission is substantially improved. We are hopeful that this revised version will now meet all expectations. For simplicity, all text changes/additions related to revisions are highlighted in the main manuscript file.

Reviewer #1 (Remarks to the Author)

Summary:

This manuscript describes the identification of USP5 as the protease responsible for cleaving the N-terminal ubiquitin-like domain (UBL) of SDE2, a protein involved in genome regulation, DNA repair, and RNA processing. The authors use a combination of biochemical fractionation, proteomics, activity-based probe design, mutagenesis, and binding assays to characterise this previously unreported processing event.

The study offers a technically thorough exploration of the USP5–SDE2 relationship. However, while the biochemical evidence is robust, the manuscript would benefit from additional clarification of methodology, reduction of redundancy across experiments, and—critically—greater biological and structural validation to support the physiological relevance and mechanistic specificity of the findings.

I believe the study has merit, but in its current form, it falls short of fully justifying its conceptual impact. The manuscript would be strengthened considerably by addressing the points below.

We thank the reviewer for the thoughtful and constructive evaluation of our manuscript and for recognising the technical rigour of the biochemical and mechanistic analyses. We appreciate the reviewer's suggestions to further strengthen the biological and structural relevance, and presentation of the study. In response, we have revised the manuscript substantially, including the addition of new cellular validation experiments, reduction of redundancy across experiments, and expansion of the structural interpretation of the USP5–SDE2 interaction. Our detailed responses to each comment are provided below.

Major Comments:

1. Functional and Phenotypic Validation is Lacking

While the authors show that USP5 cleaves SDE2 at the UBL domain, the biological consequence of this cleavage remains undefined. Given that SDE2 is implicated in splicing, DNA repair, and replication stress, it would be valuable to explore:

- Whether USP5 knockdown/inhibition with small molecules phenocopies cleavage-deficient SDE2.
- Whether cleavage impacts known SDE2 functions or associated signalling pathways.

- Whether this event is dynamically regulated during stress conditions.

The inclusion of functional/phenotypic assays would substantially improve the manuscript's impact and biological relevance.

We thank the reviewer for this important suggestion. In the revised manuscript we have expanded the cellular analyses to directly investigate the functional consequences of USP5-catalysed SDE2 cleavage on the known SDE2 biology, specifically in the context of its role in the spliceosome.
To this end, we analysed intron processing following USP5 or SDE2 depletion and observed comparable increases in intron retention in previously reported SDE2-dependent transcripts, demonstrating that USP5-mediated cleavage is required for proper SDE2 activity in spliceosomal function. This analysis was performed in HEK293 and HeLa cells, with comparable results. These data have now been incorporated into the Results section (Figure 6).
Together, these results provide functional evidence that USP5-mediated cleavage of SDE2 is required for proper SDE2 activity in RNA splicing pathways.
Regarding dynamic regulation under stress conditions, we agree that this represents an interesting direction for future work. While a detailed analysis of stress-dependent regulation is beyond the scope of the present study, we have added a discussion of this possibility in the revised Discussion.

2. Use of a Single Cell Line Limits Generalisability
The majority of experiments are conducted in HEK293 or HEK293T cells. U2OS cells are used only in Figure 4C, with no rationale provided. Given the potential for cell-type-specific regulation of DUBs, it would be important to:
- Justify the use of HEK293T as the primary model.
- Acknowledge the limitation of relying on one cell line.
- Ideally, confirm cleavage of SDE2 by USP5 in a number of unrelated cell lines.

Including broader cellular validation would strengthen the argument that USP5 is the main effector of SDE2 processing.

We thank the reviewer for raising this point. HEK293 cells were chosen as the primary model due to their high transfection efficiency and compatibility with NanoBRET and RNA interference approaches, which enabled robust perturbation and rescue experiments. We used HEK293 cells consistently across biological assays to ensure experimental comparability.
To address the reviewer's concern regarding generalisability, we have now extended our analysis to additional cell lines. Specifically, we confirmed that USP5 depletion impairs SDE2 processing in HeLa, U2OS and MCF-7. These models were selected based on their widespread use in studies of SDE2 (HeLa, U2OS), [1] DUB biology (MCF7),[2] and related pathways, allowing direct comparison with existing literature. Across all cell lines tested, we observed consistent results supporting USP5 as the primary protease responsible for $SDE2_{UBL}$ cleavage.
Furthermore, increased intron retention was also observed in HeLa cells following USP5 depletion, consistent with the phenotype observed in HEK293 cells (new Figure 6). HeLa cells were selected for this analysis for consistency as they have been previously used in studies employing similar splicing assays.[1]
These additional experiments support the conclusion that USP5-mediated SDE2 cleavage is not restricted to a single cell type.

3. Experimental Redundancy and Data Consolidation
The authors employ a wide array of orthogonal assays (SDS-PAGE, MALDI-TOF, ABP labelling, TR-FRET, and ITC) to demonstrate USP5 cleavage of SDE2. While the methodological rigour is appreciated, the repetition of similar results in multiple formats creates unnecessary redundancy. The authors might consider condensing overlapping experiments, potentially moving some data to supplementary figures, to focus the main narrative.

We thank the reviewer for this helpful suggestion. While the use of orthogonal assays was intended to provide strong validation of the biochemical findings, we agree that some results could be streamlined.
In the revised manuscript we have reduced redundancy and improved clarity by relocating several datasets to the Supplementary Information. These include portions of the MALDI-TOF DUB screening (previously Figure 2G, now in Supplementary Figure 3), and the intact LC-MS characterization of HA-$SDE2_{UBL}$-PA (previously Figure 3C, now in Supplementary Figure 5). In addition, Figure 5 and Figure 6 have been extensively redesigned: several datasets, including ABP -labelling evaluation, ITC and TR-FRET analysis have been moved to the Supplementary Information, and replaced with new data that strengthen the mechanistic and biological framework of the manuscript.
We have also consolidated overlapping datasets and simplified the presentation throughout to reduce repetition.
Together, we believe these revisions improve the clarity and focus of the manuscript while preserving the robustness of the conclusions.

4. Structural Insight is Underdeveloped
The central mechanistic claim is that SDE2-UBL mimics ubiquitin to engage USP5. However, no structural models or experimental structures are presented. AlphaFold predictions are referenced but are insufficient to demonstrate binding specificity.
Suggestions for improvement include:
- Docking models comparing USP5 bound to SDE2-UBL (WT and with mutations as in Fig. 3A), full-length SDE2, and ubiquitin.
- Consideration of experimental approaches such as x-ray crystallography, HDX-MS, cryo-EM or cross-linking mass spectrometry.

These additions would clarify the molecular basis for substrate selectivity.

We thank the reviewer for this helpful suggestion to further strengthen the structural interpretation of the USP5–SDE2 interaction. While obtaining a high-resolution structure of the USP5–$SDE2_{UBL}$ complex (e.g. by X-ray crystallography or cryo-EM) remains an important long-term objective, we have not yet been able to obtain such a structure.
To address this point within the scope of the current study, we have expanded our mechanistic analysis using complementary structural approaches. In particular, we have incorporated hydrogen–deuterium exchange mass spectrometry (HDX–MS) to interrogate the interaction between $SDE2_{UBL}$ and $USP5_{WT}$. Although this approach does not provide atomic-resolution detail, it enables mapping of interaction interfaces and conformational changes. Our HDX–MS data reveal significant perturbations in regions corresponding to both the ZnF-UBP domain and the

catalytic C-box, supporting a model in which $SDE2_{UBL}$ engages two distinct binding sites on USP5. Furthermore, comparison with HDX–MS of ubiquitin-bound USP5 demonstrates that the interaction pattern is highly conserved, consistent with a mechanism of substrate mimicry.

We have also expanded the discussion of the AlphaFold-based structural model, particularly in the context of the alanine scanning analysis (Figure 3A) and the contribution of the C-terminal tail residues of $SDE2_{UBL}$ to cleavage and the predicted interaction with USP5 (Supplementary Information, Figure 4). Mutations L70A and R73A completely prevented SDE2 cleavage, while mutations of amino acids E71 and L74 resulted in approximately 50% cleavage of SDE2, indicating that these amino acids play a supporting role in the cleavage process. When mapping these residues onto a AlphaFold 3 model of $SDE2_{UBL}$ -USP5, we noticed that while L70A, R73A and L74A correspond to positions that are also conserved in Ub and are known to contribute to recognition by DUB; E71 is not conserved in ubiquitin, suggesting that it may represent a UBL-specific determinant contributing to SDE2 recognition. More specifically, E71 seems to form a hydrogen bond interaction with Q488 of USP5.

All in all, using alanine scanning, ITC and TR-FRET, we have rigorously shown how the USP5-SDE2 interaction is driven by the UBL-domain. Complementary HDX MS study has revealed a dual binding model, which has helped to develop a more sophisticated binding-regulation model of SDE2. Alanine scanning confirmed similarities with Ub on individual amino acid scale, while also hinting at potential residues (E71), that give SDE2 its unique specificity. Overall, our analysis supports that SDE2 cleavage relies on similar structural determinants of ubiquitin. With the addition of AlphaFold-based structural models and HDX–MS we are able to provide a strong structural framework for the mechanistic interpretation of the USP5–SDE2 interaction.

Specific Comments and Clarifications:

- Line 39: The phrase "proteomic profiling and mass spectrometry" appears redundant—consider streamlining.

Thank you, this has now been modified to "proteomic profiling"

- Lines 40 and 115: The use of "in cell" and "in cellulo" is inconsistent. Standardise terminology throughout.

Thanks for highlighting this, "in cellulo" has been replaced with "in cell"

- Figure 2D: Only 24 proteins were identified across 9 fractions, which seems unexpectedly low. Can you clarify? How many total fractions were collected, and what were the criteria for inclusion?

During SEC purification, the two Source Q fractionation, A12 and B1 were individually subject to separation by SEC and 24 fractions were collected each time. These fractions were screened for their ability to cleave recombinant $SDE2_{FL}$, revealing that activity was concentrated in fractions B2 through B4 (Figure 2C). To improve clarity, we have now included a detailed workflow of sample processing and data analysis in Supplementary Figure 2.

Active SEC fractions B2-B4, together with adjacent fractions (A11-B1 and B5-B7), were subjected to tryptic digestion followed by mass spectrometry analysis. Cleavage activity was quantified by measuring the intensity of the $SDE2_{CT}$ band and used as a proxy for enzymatic

activity (Supplementary Figure 2B). Overall, proteomic analysis identified 4339 protein groups across the nine analyzed SEC fractions (Supplementary Figure 2D).
To identify candidate enzymes associated with SDE2 cleavage, we focused on proteins enriched in the active fractions. For each protein, relative abundance was correlated with the measured cleavage activity, and Pearson Correlation Coefficients (PCC) and associated p-values (Pval) were calculated. Proteins with a PCC $\geq$0.9 and Pval $\leq$ to 0.05 were retained as candidates. Using this approach, we identified 53 candidate proteins in the Source Q A12 fraction and 172 in the B1 fraction (Supplementary Figure 2D). Of these, 24 proteins were common across all active fractions. Notably, only two of these—USP5 and USP13—were annotated with known or predicted protease activity.

We have revised the main text and Methods section to improve clarity, and the full data analysis pipeline is now available as part of the PRIDE dataset.

- Figure 2G: USP13 (aa 318–363) shows cleavage activity, yet this is not recapitulated in subsequent experiments. The authors should reconcile this discrepancy.

We thank the reviewer for highlighting this important point. Upon reanalysis of the MALDI–TOF dataset we identified an error in the reporting of the quantified values for USP13. We apologise for this oversight, which has now been corrected in the revised manuscript. In the updated analysis (Figure 2G), USP13 (aa 318–863) showed low but detectable activity towards SDE2 (<10%), suggesting that it may have the capacity to process SDE2 under these assay conditions. However, subsequent orthogonal assays, including recombinant cleavage assays using both USP13 expressed both mammalian cells and *E.coli*, activity-based probe labelling *in vitro* and in cell, and cellular depletion experiments, consistently failed to detect significant USP13-dependent processing of SDE2.
We note that the MALDI–TOF assay is a highly sensitive, endpoint-based assay that can detect low levels of proteolytic activity under defined *in vitro* conditions. However, it does not fully recapitulate physiological substrate recognition or catalytic efficiency. For this reason, we employed multiple orthogonal approaches to provide a more stringent assessment of enzyme specificity and activity in both biochemical and cellular contexts.
Importantly, in cellular assays, depletion of USP13 did not impair SDE2 processing, whereas USP5 depletion consistently resulted in accumulation of full-length SDE2 and loss of cleavage. Furthermore, full-length mammalian-derived USP13 did not cleave SDE2 *in vitro*, reinforcing the conclusion that USP13 does not play a major role in SDE2 processing under physiological conditions.
Together, these data suggest that the activity observed for USP13 in the MALDI–TOF assay likely reflects low-level or context-dependent activity, rather than a physiologically relevant role in SDE2 cleavage. This point has now been clarified in the revised manuscript.

- Line 232: Activity-based protein profiling (ABPP) measures reactivity, not interaction per se. The wording should be revised accordingly.

We thank the reviewer for this important point. We have revised the text to clarify that the development of the $SDE2_{UBL}$ activity-based probe is used as a strategy to interrogate functional engagement with catalytically active enzymes, rather than direct binding alone.

While non-specific reactivity is, in principle, possible, the propargylamine warhead used in this study is known to be relatively inert and typically requires precise positioning within the catalytic site of active cysteine proteases to form a covalent adduct.[3,4] This contrasts with more reactive electrophiles, which can display higher levels of non-specific labelling.

The text has been revised accordingly (page 12), and now reads:

"Given the importance of the UBL domain sequence for $SDE2_{FL}$ cleavage, we next sought to interrogate the $SDE2_{UBL}$-USP5/USP13 interaction through the development of an $SDE2_{UBL}$ activity-based probe (ABP) designed to selectively react with enzymes capable of recognizing $SDE2_{UBL}$."

- Figure 3A: SDE2-UBL is not shown in the gel—why? Is it degraded? SDE2 is meant to be degraded upon cleavage. Should we see this effect in this figure? Please clarify. Additionally, combining this data with structural modelling of SDE2 WT, mutants, and ubiquitin with USP5 would strengthen the analysis.

We thank the reviewer for raising this point. The library of SDE2 expression constructs used for the alanine scanning analysis includes a N-terminal HA tag and a C-terminal Flag tag, allowing independent detection of both cleavage products (HA-$SDE2_{UBL}$ and $SDE2_{CT}$-Flag). Due to its small size (~9 kDa), $SDE2_{UBL}$ fragment is often poorly resolved and not readily detectable by standard SDS–PAGE. For this reason, in the original version of this manuscript we monitored cleavage primarily via the appearance of the $SDE2_{CT}$ band. However, in the revised manuscript we have now included the corresponding HA immunoblot, which clearly demonstrates the presence of the released $SDE2_{UBL}$ fragment. We would also like to clarify that, although $SDE2_{CT}$ is subject to degradation via the N-end rule pathway in cells, this does not lead to its complete disappearance under steady-state conditions, as SDE2 is continuously synthesised. In a previous publication, we performed cycloheximide chase experiments and found that $SDE2_{CT}$ has an approximate half-life of ~1.5 hours.[5]

Regarding the reviewer's suggestion to integrate structural modelling, we have expanded the structural interpretation of these data in the revised manuscript. In particular, we now discuss the mutagenesis results in the context of predicted $SDE2_{UBL}$–USP5 interactions, alongside comparison with ubiquitin, to support a mechanism of substrate mimicry (Supplementary Figure 4 and Discussion), as explained in the comment above.

- Line 497 vs. 506: The text appears to both affirm and deny USP5's ability to cleave ubiquitin from modified proteins. Please clarify.

We thank the reviewer for highlighting this point. We agree that the original wording may have been unclear and could be interpreted as contradictory.

In the revised manuscript, we have clarified that although USP5 is catalytically capable of cleaving ubiquitin, it shows a strong preference for unanchored polyubiquitin chains, which constitute its primary physiological substrates. In contrast, there are relatively few reports describing USP5-mediated deubiquitination of ubiquitin conjugated to substrate proteins, with only one example confirmed in vitro.

Consistent with this, previous studies have shown that USP5 displays little or no activity towards isopeptidic linkages between ubiquitin and substrates proteins in vitro, e.g. monoubiquitinated substrates, further supporting its limited activity towards ubiquitinated proteins.

The text has been revised accordingly to more clearly distinguish between catalytic capability and substrate preference.

- ISG15-PA in Figure 5B: Since this probe is based on a truncated C-terminal domain, the reactivity with USP5 may be underestimated. This caveat should be acknowledged.

We thank the reviewer for this important point. We agree that the use of a truncated ISG15(CTD)-based probe may underestimate its reactivity towards USP5 compared to full-length ISG15.
We have now included a statement in the revised manuscript to acknowledge this limitation that reads as follow:
“This reduced reactivity may be explained by the truncated nature of ISG15-PA, which contains a single ubiquitin-like lobe compared to native ISG15 which instead contains two lobes.”
This clarification has been added to the relevant section of the Results.

- ABP labelling: Probe titration experiments would be valuable to define the optimal ABP concentration for specific labelling.

We thank the reviewer for this helpful suggestion. We have now included a representative probe titration experiment in the Supplementary Information (Supplementary Figure 19) to illustrate the optimisation of ABP concentration for specific labelling.
We note that we observed minor batch-to-batch variation in labelling efficiency. For this reason, probe titration is routinely performed for each batch of ABP to ensure optimal and specific labelling conditions.
This has now been clarified in the revised manuscript and methods section.

- C335A mutant: This mutant increases binding to Ubiquitin affinity in DUBs and does not, in isolation, demonstrate C335-dependence. Complementary mutants such as C335R or C335S should be considered.

We agree that the C335A mutant alone does not fully demonstrate C335-dependence, as substitution of the catalytic cysteine with alanine is known to increase ubiquitin binding affinity in several DUBs. To address this, we have now included additional experiments using a complementary mutant, $USP5^{C335R}$, which disrupts substrate binding at the catalytic site due to steric hindrance. Notably, while $USP5^{C335R}$ retained overall binding affinity towards $SDE2_{UBL}$, it altered the binding stoichiometry, consistent with disruption of the C-box interaction site. These results, together with the C335A data, support the conclusion that the catalytic C335 residue contributes to substrate engagement, while also highlighting the complexity of binding at this site. We have incorporated these additional data and expanded the discussion in the revised manuscript (Figure 5D and Discussion section).

- Figure 4C: U2OS cells are used only here, but not described in the Methods. Please include their source and culture conditions.

Details on U2OS cells source and culture have been added to the Method section.
We thank the reviewer again for this constructive suggestion, which has helped improve the clarity and depth of the manuscript.

Reviewer #2 (Remarks to the Author):

Hales et al. present an intriguing manuscript exploring how the spliceosome factor and DNA damage response regulator, Silencing Defective 2 (SDE2), undergoes post-translational cleavage by the deubiquitinating enzyme USP5. SDE2 is conserved from yeast to humans, and while the enzymes responsible for this cleavage in yeast (Ubp5, Ubp15) are known, those in humans remain unidentified. By employing target-driven methods with recombinant proteases and unbiased screening, the authors discover that the deubiquitinating enzyme USP5 (not an orthologue of Ubp5/15) cleaves SDE2 by recognising its N-terminal UBL domain.

Overall, the paper is well written and the experiments are conducted to high standards, complemented by biochemical tests that support both in vitro and in cellulo findings. Occasionally, it feels somewhat exhaustive, and it's unclear why some data- such as alanine scanning mutagenesis- are included beyond the fact that the experiments were performed. My main feedback for the authors focuses on clarifying the mechanisms by which USP5 recognises SDE2.

We thank Reviewer 2 for the thoughtful and constructive evaluation of our manuscript and for recognising the quality of the experimental work and biochemical analyses. We appreciate the reviewer's suggestions aimed at improving the mechanistic clarity and presentation of the study. In response, we have revised the manuscript to clarify the rationale for several experimental approaches, expanded both the mechanist and biological understanding behind this unprecedented cleavage event, and added additional discussion point addressing the mechanistic questions raised. Detailed responses to each comment are provided below.

- Introduction: Provide additional details explaining why SDE2 must be processed for incorporation into spliceosomes. For example, is steric occlusion from the N-terminal UBL preventing its incorporation? A general description of why SDE2 requires proteolytic processing to become functional would help better set the scene for identifying enzymes that fulfil this role.

We thank the reviewer for this helpful suggestion. We have expanded the Introduction to better explain the biological rationale for SDE2 processing in the context of its known functions.

Specifically, we now clarify that proteolytic cleavage of SDE2 is central to its function and underpins two key outcomes. First, removal of the N-terminal $SDE2_{UBL}$ domain alleviates steric occlusion, enabling the C-terminal fragment ($SDE2_{CT}$) to be incorporated into the spliceosome, where it promotes efficient excision of specific introns from a subset of pre-mRNAs. This activity is linked to spliceosomal components, including Cactin/Cay1, for which genetic and biochemical evidence supports a functional interplay with SDE2.

Second, the cleaved $SDE2_{CT}$—but not full-length SDE2—negatively regulates damage-induced monoubiquitination of PCNA, a modification required for translesion DNA synthesis. Timely proteasomal degradation of $SDE2_{CT}$ is therefore necessary to relieve this inhibitory activity, allowing proper S-phase progression and replication fork recovery following DNA damage. Failure to cleave or degrade SDE2 leads to its pathological accumulation, resulting in impaired PCNA ubiquitination, replication stress, and increased DNA damage.

Importantly, SDE2 homeostasis is itself governed by this cleavage event. Removal of the UBL domain exposes a destabilising N-terminal lysine (K78), triggering degradation of $SDE2_{CT}$ via the N-end rule pathway. Thus, SDE2 cleavage acts as a molecular switch that coordinates RNA processing and replication stress responses to maintain genome integrity.

These additions provide clearer context for the biological importance of identifying the protease responsible for SDE2 processing.

- The assessment of Ubp5/15 orthologues was thorough, and it was nice to see the conventional biochemical method of cellular fractionation used to identify human proteases involved in SDE2 cleavage. However, the narrative became somewhat disrupted when the authors introduced a focused MALDI-TOF screen. They should clarify the rationale for conducting two screens, especially since the key outcome was already obtained. Additionally, more details are needed about the MALDI-TOF screen, such as why some control substrates do not react with the tested DUBs. Specifically, it should be explained how the authors conclude that USP22, USP26, and others are functional if no activity is observed with the control substrate.

We thank the reviewer for this thoughtful comment. We agree that the rationale for employing both screening approaches required further clarification and have revised the manuscript accordingly.
The cell lysate fractionation strategy was designed as an unbiased approach to identify endogenous proteolytic activity associated with SDE2 cleavage. While this method enables discovery of candidate enzymes, it does not establish direct causality or substrate specificity. In contrast, the MALDI–TOF DUB assay was implemented as a targeted, orthogonal approach to directly assess the ability of recombinant enzymes to cleave SDE2 under controlled conditions.
We therefore view these approaches as complementary rather than redundant: the fractionation identifies candidate activities in a native cellular context, whereas the MALDI–TOF assay validates enzymatic activity and specificity. Together, they provide a more robust framework for identifying USP5 as the protease responsible for SDE2 cleavage. This rationale has now been clarified in the revised manuscript.
Regarding the apparent lack of activity for some DUBs in the MALDI–TOF assay, we note that several enzymes are known to require specific cofactors, post-translational modifications, or particular substrate architectures for activation, which may not be recapitulated under the assay conditions used. As such, lack of activity against the control substrate does not necessarily indicate that these enzymes are non-functional. This approach has precedent, as similar screening strategies have previously revealed context-dependent or non-canonical activities for DUBs, including JOSD1 and ATXN3.[6,7] Nevertheless, to improve clarity, we have expanded the description of the assay conditions and interpretation in the Methods and Results sections.
In addition, as suggested, we have moved data corresponding to DUBs with no detectable activity to the Supplementary Information to streamline the main narrative.

- Furthermore, the authors observe activity from USP13 towards SDE2 in the MALDI-TOF screen, with this activity being more pronounced towards SDE2 than the control substrate. How do the authors reconcile this activity when the subsequent set of experiments shows no activity from USP13?

We thank the reviewer for highlighting this important point. Upon reanalysis of the MALDI–TOF dataset, we identified an error in the reporting of the quantified values for USP13. We apologise for this oversight, which has now been corrected in the revised manuscript.
In the updated analysis (Figure 2G), USP13 (aa 318–863) displays low but detectable activity towards SDE2 (<10%), suggesting that it may have the capacity to process SDE2 under these assay

conditions. However, this activity was not supported by subsequent orthogonal assays. Recombinant cleavage assays using USP13 expressed in both mammalian cells and *E. coli*, activity-based probe labelling in vitro and in cells, and cellular depletion experiments all consistently failed to detect significant USP13-dependent processing of SDE2.

We note that the MALDI–TOF assay is a highly sensitive, endpoint-based method capable of detecting low levels of proteolytic activity under defined in vitro conditions, but it does not fully recapitulate physiological substrate recognition or catalytic efficiency. For this reason, we employed multiple orthogonal approaches to provide a more stringent assessment of enzyme specificity and activity in both biochemical and cellular contexts.

Interestingly, we observed that USP13 constructs corresponding to the minimal catalytic domain expressed in bacteria displayed higher activity in vitro. This suggests that endogenous USP13 may exist in an auto-inhibited state or require specific post-translational modifications or cofactors for activation. Indeed, USP13 has been reported to be extensively ubiquitylated and SUMOylated, which may regulate its activity. We therefore speculate that USP13 may only be capable of cleaving SDE2 under specific cellular conditions or signalling contexts not captured in our assays.

Importantly, in cellular assays, depletion of USP13 did not impair SDE2 processing, whereas USP5 depletion consistently resulted in accumulation of full-length SDE2 and loss of cleavage. Furthermore, full-length mammalian-derived USP13 did not cleave SDE2 in vitro, reinforcing the conclusion that USP13 does not play a significant role in SDE2 processing under the physiological conditions tested here.

Together, these data indicate that the activity observed for USP13 in the MALDI–TOF assay likely reflects low-level or context-dependent activity, rather than a physiologically relevant contribution to SDE2 cleavage. This point has now been clarified in the revised manuscript.

- The reason for concentrating on residues from Ser69-Leu88 of SDE2 for USP5 cleavage was unclear, both from Figure 1 c,d and the main text, and should be clarified.

We thank the reviewer for this important observation. The alanine scanning mutagenesis was designed to systematically define the sequence determinants required for SDE2 cleavage by USP5, particularly in light of the well-established role of the ubiquitin C-terminal tail in DUB recognition. In the revised manuscript, we now clarify that this experiment was specifically aimed at testing whether the $SDE2_{UBL}$ domain contains sequence elements analogous to ubiquitin that mediate USP5 recognition, particularly within the region surrounding the conserved diglycine motif.

Based on this rationale, we focused on residues spanning S69–K78, which encompass the previously reported cleavage site (GGK motif), as well as the adjacent region (G79–L88) to capture potential contributions from residues immediately downstream of the cleavage site.

Consistent with this hypothesis, mutation of the diglycine motif—either singly or in combination—significantly impaired SDE2 processing (Figure 3A). In addition, mutations at L70 and R73 completely abolished cleavage. Notably, these residues align with positions in ubiquitin that are known to contribute to DUB recognition, supporting a conserved mode of substrate engagement. In contrast, mutation of E71 and L74 resulted in only partial impairment (~50% cleavage), suggesting a more supportive role in the interaction.

To further interpret these findings, we performed AlphaFold3 modelling of $SDE2_{UBL}$ and ubiquitin in complex with USP5 (Supplementary Figure 4). These models suggest that L70 contributes to the hydrophobic core of $SDE2_{UBL}$, analogous to L69 in ubiquitin, and that its mutation likely disrupts structural integrity required for recognition. Similarly, R73 is predicted to form

electrostatic interactions with USP5 residues (E427 and E438), comparable to those mediated by R72 in ubiquitin, while L74 occupies a hydrophobic pocket equivalent to that engaged by L73 in ubiquitin.
A notable distinction is observed for E71^{SDE2}, which is predicted to form a hydrogen bond with Q488^{USP5}, an interaction not present in ubiquitin (where the equivalent residue is V70). This suggests that, while SDE2$_{UBL}$ mimics key features of ubiquitin, it also contains unique elements that contribute to its recognition.
Together, these mutagenesis and modelling data clarify the rationale for focusing on this region and demonstrate that SDE2 cleavage is governed by sequence and structural determinants within the UBL C-terminal tail that partially overlap with, but are not identical to, those of ubiquitin. This point has now been clarified in both the main text and figure legend.

- The authors created a HA-SDE2-UBL-PA probe and tested its reactivity with USP5 and USP13. USP5 reacted, but USP13 did not, even from HEK293 extracts. However, this does not rule out USP13's ability to cleave SDE2, since it recognises full-length SDE2 but may not bind strongly enough to the UBL domain. Notably, recombinant USP13 does not react with the SDE2-UBL probe, likely because a shorter USP13 fragment was used (see size differences in Figure 3d), missing the N-terminal ZnF domain essential for USP5 activity. Therefore, the authors should repeat this assay using full-length USP13.

We thank the reviewer for this important point regarding the use of a truncated USP13 construct. The truncated variant was initially used because full-length USP13 proved unstable when expressed in bacteria and displayed minimal enzymatic activity towards ubiquitin-based substrates under the used assay conditions.
To directly address the reviewer's concern, we have now repeated the experiment using full-length USP13 recombinantly expressed and purified from Expi293 mammalian cells, which more closely reflects the physiological context and preserves potential regulatory cofactors and post-translational modifications. Under these conditions, full-length USP13 did not exhibit detectable cleavage of recombinant SDE2$_{FL}$ or react with HA-SDE2$_{UBL}$-PA probe (Figure 3F and Supplementary Figure 7). In contrast, USP5 purified using the same approach retained robust activity against both SDE2$_{FL}$ and HA-SDE2$_{UBL}$-PA, confirming that the assay conditions are compatible with SDE2 processing.
We agree that lack of reactivity with the SDE2$_{UBL}$-based probe does not formally exclude the possibility that USP13 recognises full-length SDE2 through additional domains. However, even when using full-length USP13 in both biochemical and cellular assays, we did not observe evidence of SDE2 processing.
Together, these data indicate that the absence of USP13 activity is not due to the use of a truncated construct or probe limitations but rather reflects a lack of significant catalytic activity towards SDE2 under the conditions tested. This has now been clarified in the revised manuscript.

-The in cellulo depletion using siRNAs against USP5 or USP13 shown in Figure 4c appears to show a stronger dependence on USP13 for cleavage of SDE2 than USP5. Though any comparable cleavage of endogenous SDE2 is difficult to see, why do the authors use two Western blots for full-length and cleaved SDE2? Why does there appear to be no effect by USP5? Is this experiment performed under steady-state conditions?

We thank the reviewer for raising this important point and agree that the presentation of the siRNA experiment in the original version lacked clarity. To address this, we have repeated the experiment and included both knockdown and rescue conditions, with the updated data now presented in Figure 4E. Under these conditions, depletion of USP5 consistently impaired SDE2 processing, as evidenced by accumulation of full-length SDE2. Reintroduction of wild-type USP5 restored cleavage and reduced $SDE2_{FL}$ levels, whereas the catalytic mutant $USP5^{C335A}$ failed to rescue this phenotype. We notice a slight increase in stabilization of uncleaved $SDE2_{FL}$ when rescued with $USP5^{C335A}$ and we speculate this might be due to a sequestering effect caused by $USP5^{C335A}$ being the predominant species and the C→A mutation increasing affinity for the substrates. In contrast, USP13 knockdown did not affect SDE2 cleavage, and no change was observed upon reintroduction of USP13. Cells subjected to combined USP5/USP13 knockdown also displayed accumulation of $SDE2_{FL}$; however, this phenotype was rescued only by reintroduction of $USP5^{WT}$ and not by $USP5^{C335A}$ or $USP13^{WT}$. For comparison, we also performed the same experiment in U2OS, yielding consistent results (Supplementary Figure 9). A similar phenotype was observed in HeLa and MCF-7 cells and in the NanoBRET assay (Figure 4 B-D and Supplementary Figure 9). Together, these data demonstrate that USP5, but not USP13, is required for SDE2 processing across multiple cellular contexts.
We would also like to clarify that these experiments were performed under steady-state conditions. Regarding the Western blot presentation, the bands corresponding to $SDE2_{FL}$ and $SDE2_{CT}$ were derived from the same blot but shown at different exposure levels to allow accurate visualisation of both species. This has now been clarified in the figure.

-The authors used ITC to measure the interaction between USP5 and SDE2. Why does SDE2 UBL bind USP5 with 1μM affinity, but full-length SDE2 cannot? The authors show that the USP5 ZnF drives the interaction between SDE2 UBL and USP5, but there is no contribution of the ZnF in cleaving full-length SDE2. Is it possible that the ZnF only binds to processed SDE2, similar to how it recognises the C-terminus of ubiquitin? The authors could test this by showing whether SDE2 UBL requires the C-terminal Glygly for binding to USP5. Additionally, the authors should examine whether USP5 ZnF binds with a similar affinity to SDE2 UBL compared to full-length USP5.

We thank the reviewer for these insightful comments and suggestions. Our current understanding of the SDE2-USP5 binding interaction is as follows. $SDE2_{FL}$ is recognized by USP5 with relatively low affinity, which is below the detection limit of ITC under experimentally accessible concentrations. Despite this, USP5 efficiently processes $SDE2_{FL}$, indicating that stable, high-affinity binding is not required for catalysis and is likely driven by transient interactions at the catalytic site.
In contrast, once cleavage has occurred, the released $SDE2_{UBL}$ contains an unanchored C-terminal diglycine (GG) motif, enabling high-affinity binding to the ZnF-UBP domain of USP5. This is consistent with the canonical role of the ZnF-UBP domain in recognising free ubiquitin C-termini. Supporting this model, truncation of the terminal glycine (G77) significantly reduces binding of $SDE2_{UBL}$ to both $USP5^{WT}$ and $USP5^{C335A}$, demonstrating that the GG motif is required for tight interaction.
These observations support a model in which the ZnF-UBP domain primarily engages the cleaved product rather than the substrate. This can be rationalised by a product-sequestration mechanism, whereby the ZnF-UBP domain facilitates removal of the $SDE2_{UBL}$ product from the active site and

promotes its transfer to the ZnF-UBP binding pocket, thereby allowing subsequent $SDE2_{FL}$ molecules to access the catalytic site. A similar mechanism has been proposed for ubiquitin in the context of USP26, another ZnF-containing DUB.[8]
We therefore investigated whether ZnF-UBP–mediated binding of $SDE2_{UBL}$ could contribute to product inhibition. To test this, we performed $SDE2_{FL}$ cleavage assays in vitro in the presence of excess ubiquitin or $SDE2_{UBL}$. Comparison of initial reaction rates and maximal reaction extent revealed no significant differences between conditions (Supplementary Figure 16 B-C), indicating that neither product inhibition nor catalytic activation accounts for the reduced processing observed in the recombinant system.
In contrast, our cellular data support a potential product inhibition mechanism: we observed that accumulation of $SDE2_{UBL}$ following proteasome inhibition correlates with reduced $SDE2_{FL}$ processing (Supporting Figure 16 A) supporting a model in which product accumulation can negatively impact cleavage in cells. Although this effect could not be fully recapitulated in vitro, these findings are consistent with a model in which product binding to the ZnF-UBP domain contributes to regulation of USP5 activity in a cellular context.

-The authors should also consider that the binding of SDE2 UBL to USP5 ZnF might be causing product inhibition, which could explain why only 50% of SDE2 is processed in assays with recombinantly purified proteins, but not in cell extract experiments. Have they thought about conducting competition assays—such as titrating SDE2 UBL while observing the cleavage of full-length SDE2? Additionally, what role does ubiquitin play in SDE2 cleavage? Does it influence the kinetics of the process?

We thank the reviewer for this insightful suggestion. To investigate the possibility that binding of $SDE2_{UBL}$ to the ZnF-UBP domain may lead to product inhibition, we performed competition experiments *in vitro* by titrating increasing amounts of $SDE2_{UBL}$ while monitoring cleavage of $SDE2_{FL}$. Under these conditions, we did not observe a significant effect on either the rate or extent of $SDE2_{FL}$ processing (Supplementary Figure 15 B-C), indicating that product inhibition does not occur in the recombinant system.
We also assessed the potential role of ubiquitin in modulating SDE2 cleavage. Addition of excess ubiquitin did not significantly alter $SDE2_{FL}$ processing kinetics, suggesting that ubiquitin does not compete with or enhance SDE2 cleavage under these conditions.
In contrast, our cellular data support a context-dependent product inhibition mechanism. In mammalian cells, $SDE2_{FL}$ processing proceeds efficiently, likely because the cleaved $SDE2_{UBL}$ product is rapidly cleared via the proteasome. Consistent with this, treatment with the proteasome inhibitor MG132 leads to accumulation of $SDE2_{UBL}$ and a concomitant reduction in $SDE2_{FL}$ processing, supporting a model in which product accumulation can negatively impact cleavage in cells.
Together, these results suggest that while product inhibition is not evident in a recombinant system, it may contribute to the regulation of USP5 activity in a cellular context where product turnover is dynamically controlled. This has now been clarified in the revised manuscript.

We thank the reviewer for this constructive suggestion, which has significantly improved the clarity and depth of the manuscript.

Reviewer #3 (Remarks to the Author)

The authors in this paper investigate the DUB enzyme responsible for the cleavage of human SDE2FL into SDE2CT and SDE2UBL. Through a combination of biochemical and cellular assays using distinct readouts, the authors were able to identify USP5 as the protease responsible for the proteolytic event. The experiments broadly support the claims made by the authors and have great value in the field; however, some clarifications are required, experiments should be repeated, and additional experiments can be incorporated to recommend the manuscript for publication in this journal.

Major comments:

• The band for HA-SDE2UBL-PA samples in figure 5A is notoriously faint, except for the sample where USP5 was used. It almost suggests that more SDE2-based probe was used. Can you comment on this?

We thank the reviewer for pointing out this issue. Upon re-examination, we identified an inconsistency in the presentation of the probe labelling experiment.

We have therefore repeated the experiment using equal concentrations of HA–SDE2$_{UBL}$–PA across all DUB conditions and replaced the corresponding gel image in the revised manuscript (Supplementary Figure 6A). The updated data confirm the specificity of HA–SDE2$_{UBL}$–PA probe labelling, with USP5 showing robust reactivity, while other DUBs display no labelling by HA–SDE2$_{UBL}$–PA under identical conditions.

Importantly, these corrections do not affect the overall conclusions of the experiment but improve the accuracy and clarity of the data presentation.

• It is mentioned that KD of USP5 "consistently impaired SDE2 processing, as indicated by the accumulation of SDE2FL". While this is greatly noted in the NanoBRET system (figure 4C), it is hard to appreciate for the endogenous protein (figure 4D). While there is a noticeable difference between USP13 siRNA and USP5 siRNA samples, it is negligible when comparing USP5 samples to NT or control siRNA. Can you repeat the experiment and incorporate a sample where USP5 and USP13 are overexpressed? Overexpression of the DUBs could serve as a positive control to demonstrate that enhanced cleavage mimics the SDE2FL siRNA control.

We thank the reviewer for this helpful comment. We agree that, in the original version, the effect of USP5 knockdown on endogenous SDE2 processing was less clearly visualised than in the NanoBRET assay.

To address this, we have repeated the experiment and included both knockdown and rescue conditions, together with optimised detection of endogenous SDE2. The updated data (Figure 4E) show that depletion of USP5 leads to a clear accumulation of SDE2$_{FL}$, while reintroduction of USP5$_{WT}$ restores cleavage. In contrast, USP13 depletion or re-expression does not significantly affect SDE2 processing, consistent with our overall conclusions.

We have also incorporated rescue conditions in the NanoBRET assay, which yielded consistent results. Furthermore, these experiments were performed across multiple cell lines, all of which showed similar patterns, supporting a general mechanism.

• An overview of the biological functions of SDE2 was described, but how USP5 perturbation changes its cellular functions is not explored. I believe the manuscript can greatly benefit from

tying USP5's activity to SDE2 function, which can be performed via siRNA as tested in some experiments, or assessing the potential of incorporating commercially available USP5 inhibitors. (https://pubs.acs.org/doi/10.1021/acs.jmedchem.1c00889)

We thank the reviewer for this important suggestion. In the revised manuscript, we have expanded our cellular analyses to directly assess the functional consequences of USP5-mediated SDE2 cleavage, focusing on its established role in pre-mRNA splicing.

Specifically, we analysed intron processing following depletion of either USP5 or SDE2 and observed comparable increases in intron retention across previously reported SDE2-dependent transcripts.[1] These findings demonstrate that USP5-mediated cleavage is required for SDE2 to exert its function in spliceosomal activity.

This is consistent with the established role of SDE2 processing, whereby removal of the sterically occluding $SDE2_{UBL}$ domain permits the liberated $SDE2_{CT}$ to be incorporated into the spliceosome, promoting efficient excision of specific introns from a subset of pre-mRNAs. This function has been linked to spliceosomal association with the Cactin/Cay1 complex, with prior genetic and structural studies supporting this model.

This analysis has been performed in both HEK293 and HeLa cells, yielding consistent results, and is now included in the Results section (Figure 6).

Together, these data provide functional evidence linking USP5 activity to SDE2-dependent RNA processing pathways.

Regarding the use of USP5 inhibitors, we agree that this represents an interesting complementary approach. However, given the current limitations in specificity and cellular activity of available USP5 inhibitors, we focused on genetic perturbation strategies (siRNA and rescue), which provide a more controlled and mechanistically interpretable framework.

Minor comments:

• It is mentioned that there's the possibility that a cofactor is present in cell lysate or a PTM is at play and absent in biochemical environment/recombinant protein; it has been reported before that mammalian expression systems, in some instances, can retain PTMs. Is this something that can be explored further? Is there a possibility that a signaling event on SDE2 is triggering the binding/proteolytic event in cells?

Furthermore, in the pool of proteins identified from proteomics in figure 2D, is there any protein that has been reported to interact with USP5/can serve as a scaffolding protein or aid in the cleavage of SDE2 by USP5? Highlighting such candidates could help explain why cleavage is more efficient in lysates than in vitro.

We thank the reviewer for these insightful suggestions. We agree that the increased efficiency of SDE2 cleavage observed in cell lysates compared to recombinant systems likely reflects additional layers of regulation, including the presence of cofactors or post-translational modifications (PTMs).

While a detailed investigation of these possibilities is beyond the scope of the present study, we have considered this aspect in our analysis. In particular, the enhanced activity observed with mammalian-expressed USP5 compared to bacterially expressed protein suggests that PTMs or associated factors may contribute to optimal catalytic function. Likewise, it is plausible that SDE2 itself may undergo modifications or adopt conformations in cells that favour recognition and cleavage by USP5.

In addition, we observed accumulation of full-length SDE2 ($SDE2_{FL}$) upon proteasome inhibition with MG132. This suggests a potential product inhibition mechanism in which continuous cleavage and subsequent degradation of the $SDE2_{UBL}$ domain maintain the reaction in a constitutively active state. In contrast, *in vitro* systems lacking product turnover may reach a steady state, limiting overall processing efficiency. These points have now been incorporated into the revised Discussion.
Regarding the re-analysis of the proteomic dataset (Figure 2D), we did not identify any known cofactors or interacting proteins previously reported to regulate USP5 or SDE2. However, as this dataset was not generated using an enrichment-based approach (e.g. immunoprecipitation), it is unlikely to capture transient or low-abundance cofactors. As such, while these candidates are intriguing, further targeted studies will be required to establish any functional contribution.
Finally, we agree that signalling-dependent regulation of SDE2 cleavage represents an interesting possibility. Although we have not directly tested this, it is conceivable that cellular cues—such as replication stress or DNA damage signalling, where SDE2 is known to function—may modulate this process. We have now included this perspective in the revised Discussion.

• In addition to the previous comment in figure 5A, it seems that USP7 has ISG15 activity, as there's a faint band above USP7 in lane 4 of the USP7 samples; however, it's hard to tell since the band for USP7 is too broad and it masks this finding. Is this something previously observed for USP7? Can the experiment be repeated using different PAGE conditions to promote better separation of high molecular weight bands?

We thank the reviewer for this interesting observation.
To address this point, we repeated the experiment under modified conditions, including increased ABP concentrations. We consistently observed a high-molecular-weight band in the USP7 samples following ISG15-PA treatment. However, we also detected a similar higher-molecular-weight species upon Ub-PA treatment for both USP7 and USP5. These findings suggest that the observed band is more likely due to non-specific or off-target covalent labelling rather than bona fide ISG15-directed activity of USP7.
While this observation may be of broader interest, a detailed mechanistic investigation would require further optimisation of separation conditions and orthogonal validation assays. As this lies beyond the scope of the present study, we have not pursued this further but have now clarified this point in the revised manuscript.

• Mislabeling:
o In line 264, figure 3C is referenced; however, it highlights the experiment in figure 3D? Can you confirm?

We thank the reviewer for highlighting this inconsistency. In the original manuscript, the figure reference was incorrect and should have referred to Figure 3D rather than Figure 3C.
In the revised manuscript, Figure 3 has been updated and reorganised, and the correct panel is now labelled as Figure 3C. The text has been corrected accordingly to ensure consistency.

o Figures 6F,G are mislabeled as 6H,I in text and figure caption

Thank you, this has now been corrected

• Figure 5C can be placed in the supplemental section. It feels secondary and not critical to the main argument of the figure.

We agree with the reviewer's suggestion. The original Figure 5C has now been moved to the Supplementary Information.
In the revised manuscript, Figure 5 has been updated and reorganised to incorporate additional data supporting the structural and mechanistic aspects of $SDE2_{UBL}$ binding to USP5, thereby strengthening the main narrative.

References:

1 Floro, J. *et al.* SDE2 is an essential gene required for ribosome biogenesis and the regulation of alternative splicing. *Nucleic Acids Res.* **49**, 9424-9443 (2021). https://doi.org/10.1093/nar/gkab647
2 Pinto-Fernández, A. *et al.* Comprehensive Landscape of Active Deubiquitinating Enzymes Profiled by Advanced Chemoproteomics. *Front. Chem.* **7** (2019). https://doi.org/10.3389/fchem.2019.00592
3 Ekkebus, R. *et al.* On Terminal Alkynes That Can React with Active-Site Cysteine Nucleophiles in Proteases. *J. Am. Chem. Soc.* **135**, 2867-2870 (2013). https://doi.org/10.1021/ja309802n
4 Arkona, C. & Rademann, J. Propargyl Amides as Irreversible Inhibitors of Cysteine Proteases—A Lesson on the Biological Reactivity of Alkynes. *Angew. Chem. Int. Ed.* **52**, 8210-8212 (2013). https://doi.org/https://doi.org/10.1002/anie.201303544
5 Szulc, N. A. *et al.* DEGRONOPEDIA: a web server for proteome-wide inspection of degrons. *Nucleic Acids Res.* **52**, W221-W232 (2024). https://doi.org/10.1093/nar/gkae238
6 De Cesare, V. *et al.* Deubiquitinating enzyme amino acid profiling reveals a class of ubiquitin esterases. *Proc. Natl. Acad. Sci. USA* **118**, e2006947118 (2021). https://doi.org/10.1073/pnas.2006947118
7 Ritorto, M. S. *et al.* Screening of DUB activity and specificity by MALDI-TOF mass spectrometry. *Nat. Commun.* **5**, 4763 (2014). https://doi.org/10.1038/ncomms5763
8 Alexandrovics, J. A. *et al.* ZnF-UBP domains regulate deubiquitinase activity by relieving ubiquitin product inhibition. *bioRxiv*, 2025.2009.2028.679104 (2025). https://doi.org/10.1101/2025.09.28.679104

A Proteolytic Switch: USP5 controls SDE2 function via UBL-directed cleavage

Second round of Reviewer's Comments – Response letter

We would like to thank all reviewers for the time and effort dedicated to evaluating our revised manuscript. We are pleased to see that the additional experiments, analyses, and clarifications introduced during revision were positively received and helped to further strengthen the study. We are grateful for the thoughtful and constructive feedback provided throughout the review process, which has significantly improved the quality and clarity of the manuscript.

As a result, we hope that this revised version now addresses all outstanding concerns and will be suitable for publication. For clarity, all text changes and additions introduced during revision are highlighted in the revised manuscript.

Reviewer #1 (Remarks to the Author):

The revised manuscript by Hales et al. presents a technically rigorous and comprehensive study identifying USP5 as the protease responsible for SDE2 cleavage. The biochemical and mechanistic aspects of the work are strong, and the use of multiple orthogonal approaches is a clear strength.

The authors have addressed several of the previous concerns, most notably by incorporating additional cellular data linking USP5 depletion to intron retention in SDE2-dependent transcripts. This represents a meaningful improvement over the original submission.

However, despite these revisions, I remain concerned that the manuscript still falls short of fully establishing the biological relevance and mechanistic robustness expected for publication in Nature Communications. In particular, key controls are missing in critical experiments, the functional validation remains limited in scope, and some of the structural interpretations appear overstated given the available data.

For these reasons, I believe that further revision is required.

We thank Reviewer #1 for their careful evaluation of our revised manuscript and for recognising the technical rigor, comprehensive nature, and methodological strengths of the study. We particularly appreciate their acknowledgment that the additional experiments linking USP5 depletion to intron retention in SDE2-dependent transcripts meaningfully strengthened the work and improved the biological context of our findings.
We also appreciate the reviewer's positive assessment of the orthogonal approaches employed throughout the study and their recognition of the potential broader implications of this work for understanding DUBs in novel regulatory contexts.

At the same time, we acknowledge the reviewer's remaining concerns regarding the depth of functional validation and the current limitations of the structural interpretation. In response, we

have further revised the manuscript to more carefully moderate our structural conclusions, particularly in relation to the HDX-MS data and AlphaFold-based modelling, ensuring that the interpretation remains appropriately supported by the available evidence. We have also clarified the scope of the study and tempered statements where appropriate to avoid overstating the mechanistic or biological implications. Detailed point to point response can be found below. We hope that the reviewer will agree that the revised manuscript now provides a balanced and sufficiently supported study suitable for publication.

Major comments
1. Missing controls in the intron retention experiments (Figure 6) The intron retention analysis is now central to the manuscript's biological claims. However, the figure does not clearly show knockdown efficiency for USP5 and/or SDE2 within the same experimental context. Without these controls, it is difficult to directly attribute the observed phenotype to the intended perturbation. These controls are essential and should be included alongside the RT-PCR data.

We thank the reviewer for this important comment. In response, we have now included Western blot validation of USP5 and SDE2 knockdown efficiency alongside the intron retention analyses. These additional controls have been incorporated into the supporting information as new **Supporting Figure 19** and confirm that the observed splicing phenotypes correlate with effective depletion of the targeted proteins. We believe these data strengthen the interpretation of the experiments and more directly support the link between USP5/SDE2 depletion and the reported intron retention events.

2. Limited functional validation of biological relevance
While the addition of intron retention data is appreciated, the manuscript still relies largely on a single molecular readout (without accompanying controls) to support the biological significance of USP5-mediated SDE2 cleavage. Given the broader roles of SDE2 in splicing, DNA repair, and replication stress, this represents a relatively narrow validation of biological impact. At minimum, the authors should better contextualise this limitation; ideally, an additional proof-of-concept experiment demonstrating functional consequences at the cellular level (e.g. under stress conditions) would substantially strengthen the manuscript.

We thank the reviewer for this important point and agree that additional functional validation would further strengthen the biological context of USP5-mediated SDE2 processing.

[**Editorial note: third party material, and unpublished, confidential data redacted**]

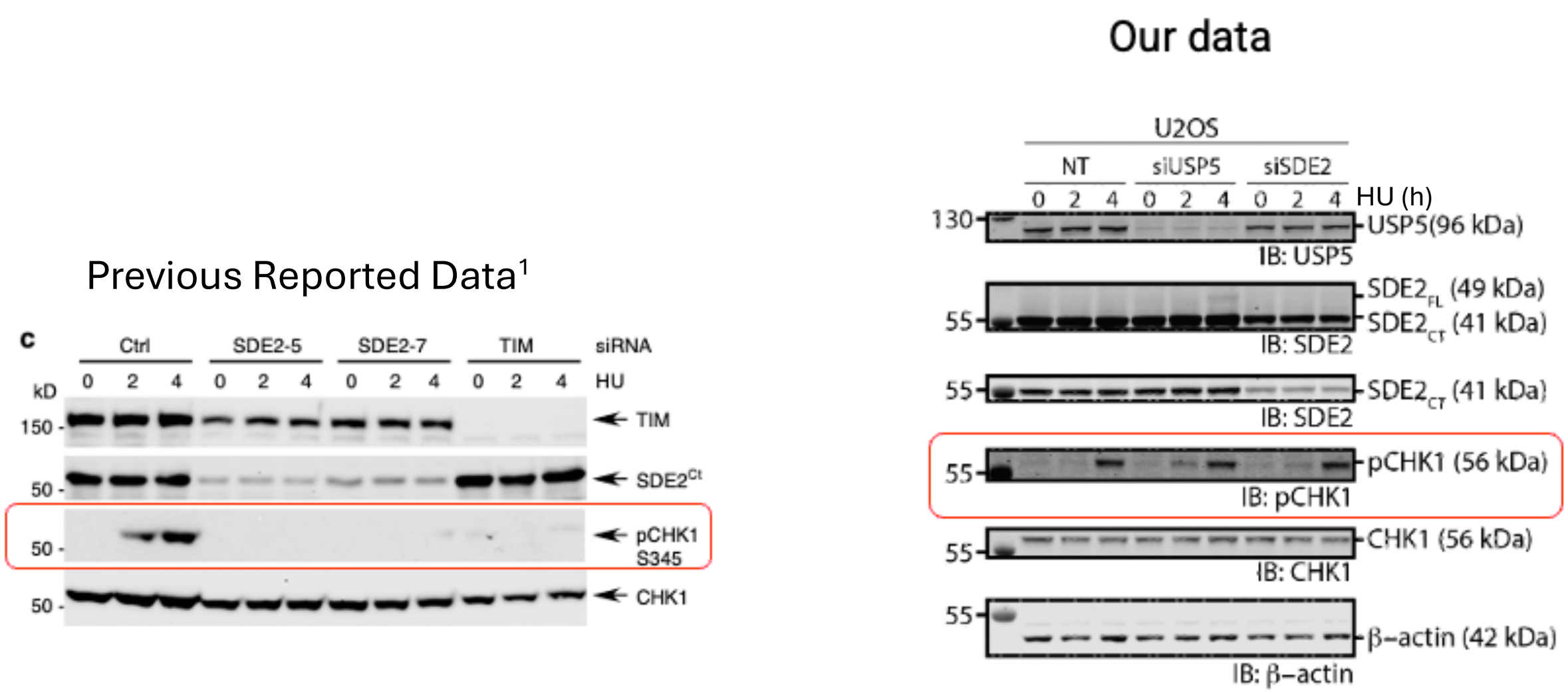

***Figure 1**: (Left) Western blot analysis of pCHK1 levels upon siRNA depletion of SDE2 or TIM under HU treatment. The blot was extracted from previous reported data In U2OS cells[1]. (Right) Western blot analysis that we have performed under the same condictiones that highlights the different phenotype we have been observing. The red rectangles highlight the pCHK1 blots.*

Despite extensive optimisation, multiple biological replicates, and careful benchmarking against the published methodology, the effects we observed are not consistent with the phenotype previously reported (i.e. a full reduction in CHK phosphorylation under SDE2 knockdown). The experiment was repeated in HEK293T cells with similar results. In light of potential (real or perceived) conflicts of interest, we have not been in a position to contact the corresponding authors directly to clarify the experimental conditions. Given the inconsistency of these results, we felt that inclusion of these data in the manuscript would not provide a sufficiently robust or interpretable addition to the study.

Importantly, although SDE2 has been linked to DNA repair and replication stress pathways, the only direct connection currently reported between SDE2 cleavage and its biological function is in the context of splicing regulation, making intron retention the most appropriate experimental readout for this study. In this context, by establishing a direct link between USP5 catalysed SDE2 cleavage and mRNA splicing, we have provided experimental validation for what has thus far remained a proposed mechanistic model. While the current manuscript primarily focuses on splicing-related consequences of impaired SDE2 processing, we do not exclude broader roles for USP5-mediated SDE2 cleavage in replication stress or genome maintenance pathways. Rather, we consider these important directions for future investigation that will require further dedicated mechanistic studies.

To better reflect this, we have tempered the discussion throughout the manuscript and more explicitly acknowledged the current limitations regarding functional validation at the cellular level.

3. Clarity and interpretation of gel-based data
Figures presenting intron retention and related assays show bands without clear annotation of what is being measured (e.g. RT-PCR products vs immunoblotting). While this may be inferred by a specialist reader, clearer labelling within figure panels and legends is necessary to ensure readability and transparency.

We thank the reviewer for this helpful comment and agree that clearer annotation and presentation of the gel-based data can improve interpretability. In response, we have revised the relevant figures to provide clearer labelling of bands, including explicit indication of the measured species and assay type where appropriate. We have also updated the figure legends to improve readability and facilitate interpretation of the RT-PCR and DNA agarose gel data.

4. Structural interpretation remains somewhat overstated
The inclusion of HDX-MS data is a valuable addition; however, the conclusions drawn from these experiments should be moderated. The coverage of USP5 is incomplete (85% is considered low for HDX-MS), including regions proximal to the catalytic site, which limits the strength of the mechanistic claims regarding binding mode and substrate mimicry. These limitations should be more explicitly acknowledged, and the interpretation tempered accordingly.

We thank the reviewer for this thoughtful comment. While the HDX-MS dataset does not provide complete sequence coverage of USP5, we note that the achieved coverage (~85%) remains within an acceptable range for this type of analysis. We have now explicitly stated the sequence coverage in the main text, directly referenced a sequence coverage map (Supplementary Figure 17), and clarified the lack of coverage of small fragments within the catalytic site .

Importantly, our interpretation is not based solely on the HDX-MS data, but is also supported by the direct comparison of HDX changes observed upon ubiquitin binding together with the accompanying biophysical analyses. These orthogonal datasets collectively support the proposal that $SDE2_{UBL}$ engages USP5 in a similar binding mode to ubiquitin.

To better reflect the limitations of the dataset, we have revised the text throughout to emphasise that the structural interpretation represents a proposed model of interaction rather than a definitive mechanistic description.

Reviewer #2 (Remarks to the Author):

The authors should be commended for their thorough effort in addressing all the reviewer comments and for addressing my concerns. In particular, the inclusion of functional assays demonstrating splicing impairment in several candidate proteins further strengthened the paper and underscored the importance of USP5-mediated processing of SDE2.

I have one minor comment: for the AlphaFold 3 modelling, please include the corresponding PAE plots in Supplementary Figures 4a, b and in Supplementary Figure 10.

We thank Reviewer #2 for their positive assessment of our revised manuscript and for recognising the importance of the additional functional assays linking USP5-mediated SDE2 processing to splicing regulation.

In response to the reviewer's final comment, we have now included the corresponding PAE plots for the AlphaFold3 models in the revised Supplementary Information. These have been added to **Supplementary Figure 4 as panel C and D, and Supplementary Figure 10 as panel B**.

Reviewer #3 (Remarks to the Author):

The manuscript is well-written, and this revised version shows significant improvements in cohesion and scientific rigor. The follow-up experiments and the subsequent refinements to the data effectively support the authors' original claims.

This work is significant to the field, as it opens new avenues for studying DUBs in novel regulatory contexts. Furthermore, the methodology is sound and the procedures are described with sufficient detail to allow for reproducibility. Therefore, I recommend this revised manuscript for publication

We thank Reviewer #2 for their positive and encouraging assessment of our revised manuscript. We greatly appreciate their recognition of the improvements in cohesion and scientific rigor, as well as their support for the additional experiments and analyses included during revision.

We are also grateful for the reviewer's positive evaluation of the significance of this work and its potential to open new avenues for studying DUBs in novel regulatory contexts. Finally, we appreciate their recommendation for publication and their acknowledgment that the methodology and experimental procedures are described with sufficient detail to ensure reproducibility.

References

1. Rageul, J., Park, J.J., Zeng, P.P. et al. SDE2 integrates into the TIMELESS-TIPIN complex to protect stalled replication forks. Nat Commun 11, 5495 (2020). https://doi.org/10.1038/s41467-020-19162-5
